# Organocatalytic Stetter Cyclization of Pentoses for the Synthesis of Polyhydroxylated Cyclopentanone Scaffolds

**DOI:** 10.1021/acs.joc.5c01792

**Published:** 2025-10-01

**Authors:** Christoph Suster, Nicolas Kratena, Kyryl Bocharov, Christian Stanetty

**Affiliations:** 27259 Institute for Applied Synthetic Chemistry TU Wien, Getreidemarkt 9, Vienna 1060, Austria

## Abstract

An organocatalytic approach for the carbocyclization of aldopentoses is disclosed. After initial activation, with the introduction of the intramolecular Michael acceptor in the substrate, the NHC-catalyzed Stetter reaction can be performed, giving rise to cyclopentanone scaffolds bearing multiple chiral centers and protected hydroxy groups. An optional controlled one-pot elimination provides the corresponding cyclopentenones as well. Due to the commercial availability of d- and l-pentoses all possible stereochemical permutations of the products can be prepared conveniently and affordably.

## Graphical abstract

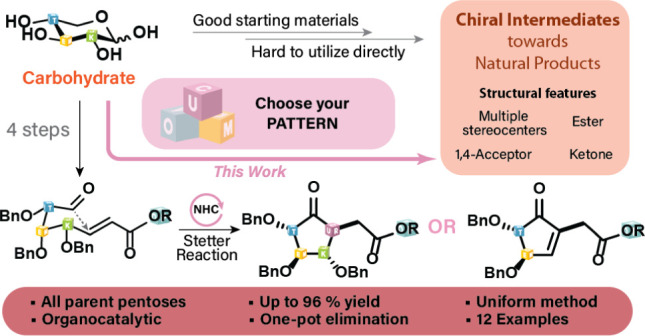

Monosaccharides are abundantly available from natural sources as single enantiomers and can thus be used as building blocks for the asymmetric synthesis of complex structures (chiral pool strategy). The deployment of abundant carbohydrates like d-glucose or l-arabinose as starting materials in total synthesis, however, remains underrepresented when compared to typical monoterpene precursors such as carvone or pinene.^1^ This is surprising, given that sugars exhibit multiple well-defined stereocenters, which could conceivably be transposed to complex natural products^2,3^ containing multiple hydroxy groups on the skeleton. The reason for this discrepancy likely stems from the expansive body of literature on the transformation of monoterpenes into useful chiral building blocks.^4–8^ Despite singular reports already demonstrating the usefulness of carbohydrates in total synthesis,^9–13^ the development of useful chiral building blocks accessible from aldoses is, to date, still lacking due to difficulties in transferring synthetic methods into the realm of sugar chemistry. The chiral polyhydroxylated carbohydrate backbone exerts high demands in terms of functional group tolerance and regioselectivity, often leading to unforeseen complications when applying otherwise well-established methods. In continuation of our previous efforts to expand the scope of useful transformations of the sugar scaffold based on organocatalytic activation with *N*-heterocyclic carbenes (NHC),^14^ we wanted to explore the intramolecular Stetter cyclization of functionalized pentoses.^15–20^ This would allow access to cyclopentanones with multiple chiral centers, which would be suited for the preparation of certain natural products (Scheme 1A). The reactivity we were aiming for had been shown in a singular report (Scheme 1B) from Matsuda,^21^ who used 4 equivalents of SmI_2_ to trigger C-1 radical formation in hexoses and subsequent cyclization toward C-5 of the carbohydrate. Intrigued by the results of this pioneering study, we set out to achieve a similar carbocyclization^22–29^ of appropriate substrates by means of Umpolung via NHC catalysis.^30^

**Scheme 1:**
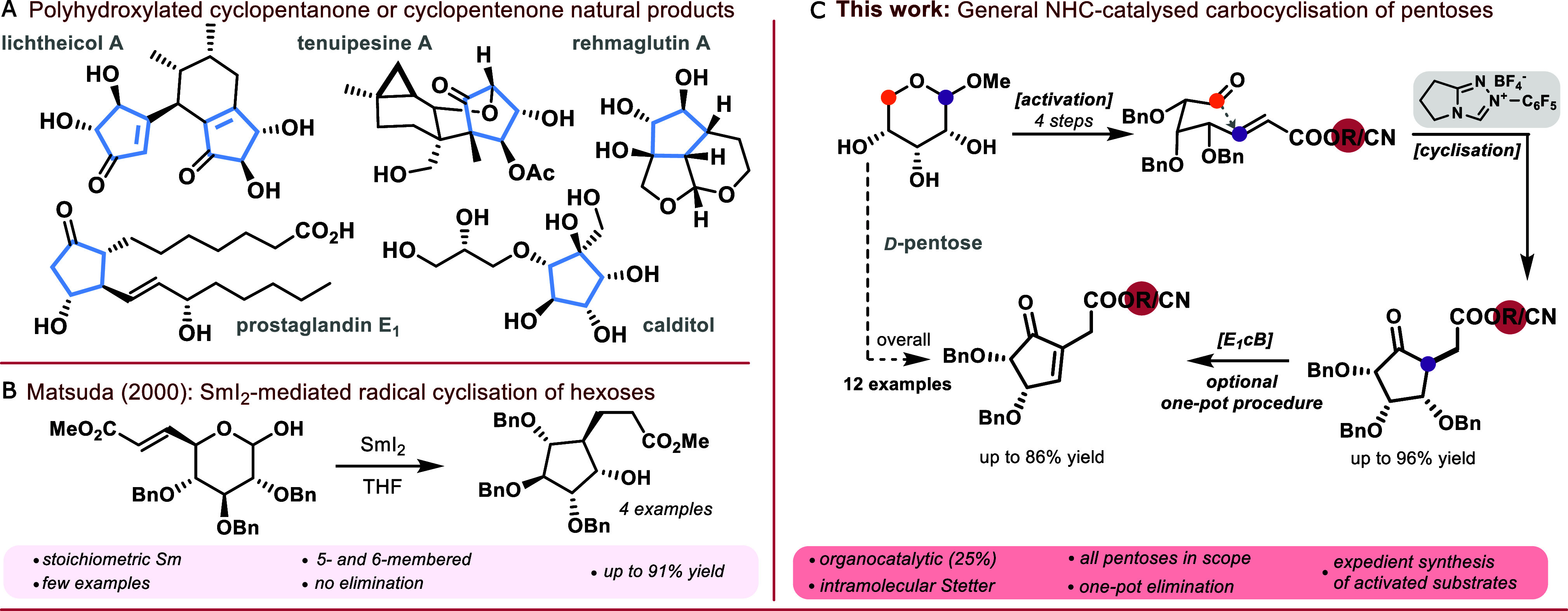
A: Examples of Highly Oxidized Cyclopentanes in Nature. B: Reductive Cyclization of Benzylated Hexose Derivatives by Matsuda.^21^ C: General Overview of the NHC-Catalyzed Stetter Reaction of Aldo-Pentose Derivatives for the Synthesis of Trihydroxy- and Dihydroxycyclopentanones

Envisioning an utmost general solution, we aimed for a cyclization precursor that is accessible via an easy and general synthetic sequence, which is not influenced by the stereogenic pattern provided by the starting sugar. Among other candidate concepts, a 4-step synthetic approach from methyl glycosides consisting of: 1. Global protection of hydroxy groups; 2. Acidic hydrolysis of the C-1 acetal; 3. Wittig reaction to introduce the activated olefin; and 4. Dess-Martin oxidation, became the method of choice (see Scheme 2, top). The protocols for these 4 steps are well established in the literature,^22,24,31,32^ very robust, and were successfully applied to all d-pentoses (see Supporting Information). The result is a fully protected linear aldehyde, such as **1a**, with the carbonyl functionality residing on the former C-5 of the starting pentose. We decided to focus on benzyl protecting groups, as they promised good stability during the transformation while having orthogonal cleaving conditions after cyclization. Following the works of the Rovis group, we expected little influence from the substituents.^33^ Starting from methyl *xylo*-pyranoside and applying our 4-step protocol provides aldehyde **1a** in 4 steps and 59% yield (2 chromatographic purifications) at decagram scale. With this substrate in hand, optimization of the reaction conditions could be performed. At first, we screened for a catalyst with the right reactivity profile; thus, five (**A**–**E**) commonly used NHC catalysts were employed. In this case, the reaction was highly dependent on the catalyst, with only the pentafluorinated triazole catalyst **A** efficiently catalyzing this transformation toward carbocycle **2a** with, e.g., Et_3_N activation (see Table 1, entries 1–5) at concentrations below the catalyst.

**Table 1:**
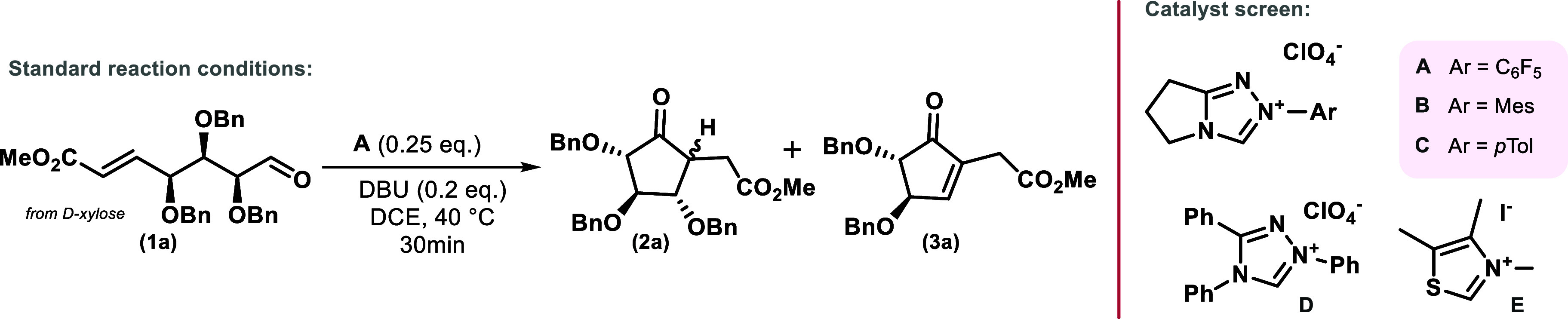
Optimization of the Cyclization Step via Catalyst, Base, and Solvent Screening

#	Solvent	NHC	Temp.	Base	Yield (2a) (%)^a^	Yield (3a) (%)^a^	Recovery (1a) (%)^a^
1	DCE	A	40 °C	Et_3_N	87	0	4
2	DCE	B	40 °C	Et_3_N	2	0	73
3	DCE	C	40 °C	Et_3_N	2	0	72
4	DCE	D	40 °C	Et_3_N	0	0	92
5	DCE	E	40 °C	Et_3_N	3	0	97
6	DCE	A	40 °C	-	0	0	>99
**7**	**DCE**	**A**	**40 °C**	**DBU**	**94**	**0**	**0**
8	DCE	A	40 °C	DIPEA	93	0	0
9	DCE	A	40 °C	2,6-lutidine	3	0	97
10	DCE	A	40 °C	Et_3_N^b^	32	39	0
11	DCE	B	40 °C	DBU	6	0	20
12	DCE	E	40 °C	DBU	<5	0	87
13	DCE	A^c^	40 °C	DBU^c^	55	0	30
14	DCE^d^	A	40 °C	DBU	91	0	5
15^e^	DMF	A	40 °C	DBU	58	0	28
16^e^	DMSO	A	40 °C	DBU	67	0	36
17^e^	THF	A	40 °C	DBU	87	0	20
18	PhMe	A	40 °C	DBU	91	0	0

a Yields were determined by calibrated HPLC-UV after 30 min.

b 1.0 equiv. instead of 0.2 equiv. of base was used.

c 0.1 equiv of precatalyst and 0.08 equiv. of base used.

d Nonanhydrous solvent used.

e Extended reaction time to 3 h.

**Scheme 2:**
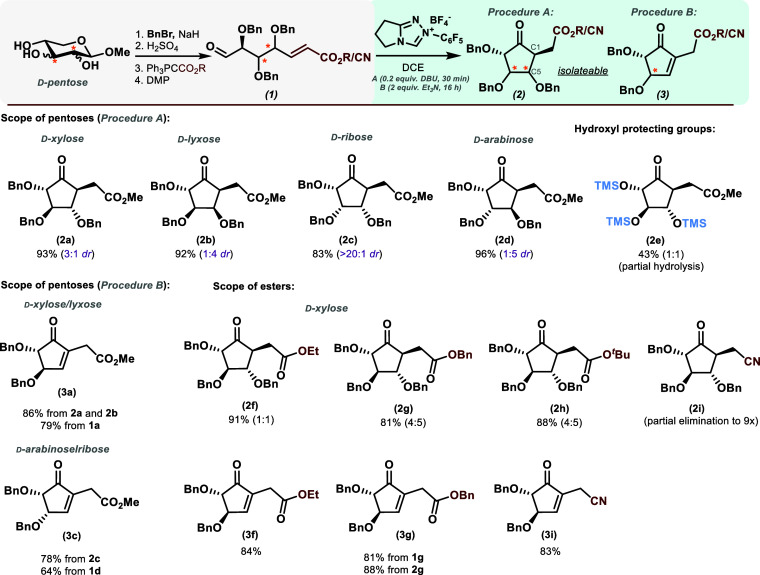
Scope for the Carbocyclization of Pentoses by NHC Catalysis After a Multistep Activation Protocol^a^

a All yields shown are isolated yields.

Next, the base screening was tackled, which resulted in a slight increase in yield compared to Et_3_N with bases DBU and DIPEA (entries 7 and 8), whereas 2,6-lutidine was not basic enough to enable the reaction (entry 9). Using equimolar quantities of base (entry 10) led to predominant elimination of the C-2 benzyloxy group via an E1cB mechanism. We then revisited catalysts **B** and **E** using commonly used DBU as a stronger base^34^ to rule out insufficient carbene formation of these precatalyst salts, still yielding no conversion to the targeted carbocycle (entries 11, 12). With an optimized pair of NHC precatalyst and base at hand, we tried to lower the precatalyst loading (see Supporting Information for further details). However, even at 10 mol %, the reaction remained incomplete (entry 13). Regarding the choice of solvents, the reaction can be carried out in solvents of different polarity (entries 14–18). Generally, DCE and toluene gave excellent yields (entries 7 and 18), whereas more polar solvents showed incomplete conversion even with extended reaction times (entries 15–17). Notably, even with water-saturated DCE, the reaction performed equally well (entry 14).

With optimized conditions (entry 7) available, we turned our attention to eliminating product **3a** to allow for optional access to this product directly by means of controlled elimination. Based on the conditions showing incomplete elimination using 1.0 equiv. of Et_3_N (entry 13), we were pleased to find that a clean transformation was achieved overnight using 2.0 equiv. of base and slightly increasing the temperature to 50 °C. The elimination can also be achieved within minutes using DBU, but Et_3_N generally showed much cleaner reactions and better yields in this protocol. With the modified procedure, the cyclopentenone structure **3a** is accessible directly from its parent aldehyde **1a** (via a one-pot procedure) or from the respective carbocycles **2a** or **2b** through base-mediated elimination in a subsequent step.

With this, we set out to explore the scope of the whole transformation by applying it to all d-pentoses (see Scheme 2). Gratifyingly, both the precursor synthesis and the cyclization protocol proceeded smoothly for all d-pentoses, with yields ranging from 83 to 96% for a complete series of methyl esters. We observed that the *syn*-configuration between C1 and C5 was favored (4:1) for the *lyxo*-product, while all other stereochemical patterns gave good to excellent selectivity toward the *anti*-product. Notably, for the *ribo*-configured carbocycle **2c,** we obtained the antiproduct as a single isomer, which we attribute to the inherent instability of the *syn*-configuration in the transition state, having 4 bulky residues oriented toward the same face above the plane. The obtained diastereomeric ratios follow our predictions from a simple transition state model (see Supporting Information), where the Michael acceptor always arranges quasi-equatorially in a 5-membered chairlike TS. Interestingly, we observed no change in diastereoselectivity depending on the *E/Z* configuration of the cyclization precursor, as *E/Z* mixtures gave comparable reaction outcomes (see Supporting Information). Specifically, we observed an inherent tendency of the *xylo*-configured cyclopentanone **2a** to equilibrate at C1 toward an ∼1:1 *syn/anti* mixture upon, for example, extended (10 min) treatment with 0.5 M HCl. We did not observe this behavior for other configurations.

Expanding the scope with respect to the Stetter acceptor, to our delight, a variety of ester functionalities were well tolerated by this method. On the *xylo*-scaffold rather small methyl and ethyl esters are equally well tolerated as bulky tert-butyl and benzyl esters. Further, we could show that an α,β-unsaturated nitrile undergoes carbocyclization within seconds; however, in this particular case, we were unsuccessful in preventing partial follow-up elimination even with reduced amounts of base. We also tested per-OTMS protection in the substrate with our cyclization protocol to obtain carbocycle **2e**, where cyclization proceeds smoothly, but partial hydrolysis of the OTMS group is an issue (access to OTMS-protected precursors via a different procedure^35^, see Supporting Information). We demonstrated the flexibility of the method by obtaining the follow-up elimination products of the methyl ester series (**3a** and **3c**) cleanly in excellent yield, in a one-pot and multistep fashion from both possible parent compounds. Also, eliminated ethyl and benzyl esters (**3f** and **3g**), as well as the nitrile compound **3i**, are accessible from their parent cyclopentanones.

Interestingly, our efforts to extend the methodology to the larger analogous hexose structures have been unsuccessful—likely due to the lower reactivity of hexose-derived precursors with α,β-unsaturated ester groups to form the six-membered ring system, in consistency with reports on simpler cyclizations.^36–38^ However, the included cyclization of the nitrile-based Michael acceptor **1i,** with its outstanding reactivity, presents a potential avenue to enable analogous six-membered rings in future studies.

Summing up, we have developed a robust and general strategy for the synthesis of highly functionalized cyclopentanones from naturally occurring pentoses using an organocatalytic NHC-mediated intramolecular Stetter reaction. The method tolerates a variety of functional groups and stereochemical configurations with consistent selectivity and high efficiency across all tested substrates. The ability to selectively eliminate the C5 benzyloxy group postcyclization provides a direct route to the corresponding cyclopentenones, expanding the synthetic versatility of the protocol. This approach enables the construction of enantiomerically pure, densely functionalized carbocycles from inexpensive and commercially available sugars. The resulting products feature well-defined stereochemical arrays and are amenable to further chemistry toward natural product synthesis and medicinal chemistry.

We believe that the method represents a significant step forward in exploiting the chiral pool of carbohydrates for complex molecule construction.

## Supplementary Material



## Data Availability

The data underlying this study are available in the published article and its Supporting Information.

## Notes

General information, synthetic procedures for precursor synthesis, analytical data of all compounds are provided as well as ^1^H and ^1^C NMR images (PDF)

## Notes

Dedicated to Professor Peter Stanetty on the occasion of his 80th birthday.

## References

[1] Scifinder.com Total Synthesis AND Terpenes” 1047 unique publications. “Total Synthesis AND Carbohydrates” 540 unique publication hits.

[2] Ferrier R. J., Middleton S. (1993). The conversion of carbohydrate derivatives into functionalized
cyclohexanes and cyclopentanes. Chem. Rev..

[3] Shing, T. K. M. Carbocyclization of Carbohydrates to Hydroxylated Cycloalka(e)nes TCIMAIL 2018 178

[4] Fernandes R. A., Khatun G. N., Moharana S. (2024). Traversing the chiral
pool potential
of carvone in total synthesis of natural products. Tetrahedron.

[5] Lusi R. F., Perea M. A., Sarpong R. (2022). C–C Bond Cleavage of α-Pinene
Derivatives Prepared from Carvone as a General Strategy for Complex
Molecule Synthesis. Acc. Chem. Res..

[6] Selka A., Abidli A., Schiavo L., Jeanmart L., Hanquet G., Lubell W. D. (2025). Recent Advances
in Sustainable Total Synthesis and
Chiral Pool Strategies with Emphasis on (−)-Sclareol in Natural
Products Synthesis. Eur. J. Org. Chem..

[7] Brill Z. G., Condakes M. L., Ting C. P., Maimone T. J. (2017). Navigating the Chiral
Pool in the Total Synthesis of Complex Terpene Natural Products. Chem. Rev..

[8] Casiraghi G., Zanardi F., Rassu G., Spanu P. (1995). Stereoselective Approaches
to Bioactive Carbohydrates and Alkaloids-With a Focus on Recent Syntheses
Drawing from the Chiral Pool. Chem. Rev..

[9] Konrad D. B., Rühmann K.-P., Ando H., Hetzler B. E., Strassner N., Houk K. N., Matsuura B. S., Trauner D. (2022). A concise synthesis
of tetrodotoxin. Science.

[10] Yu S., Liu Y., Shang C., Du Y., Liu J. (2021). A carbohydrate-based
approach for the total synthesis of sawaranospirolide C. Tetrahedron Lett..

[11] Nicolaou K. C., Yang Z., Shi G.-Q., Gunzner J. L., Agrios K. A., Gärtner P. (1998). Total synthesis of brevetoxin A. Nature.

[12] Suman P., Raju B. C. (2014). Carbohydrate-based first stereoselective total synthesis
of bioactive cytospolide P. Org. Biomol. Chem..

[13] Hubschwerlen C. (1986). A convenient
synthesis of L-(S)-glyceraldehyde acetonide from L-ascorbic acid. Synthesis.

[14] Draskovits M., Kalaus H., Stanetty C., Mihovilovic M. D. (2019). Intercepted
dehomologation of aldoses by N-heterocyclic carbene catalysis - a
novel transformation in carbohydrate chemistry. Chem. Commun..

[15] Gobert J., Glomb M. A. (2009). Degradation of Glucose:
Reinvestigation of Reactive
α-Dicarbonyl Compounds. J. Agric. Food
Chem..

[16] Zhang J., Xing C., Tiwari B., Chi Y. R. (2013). Catalytic Activation
of Carbohydrates as Formaldehyde Equivalents for Stetter Reaction
with Enones. J. Am. Chem. Soc..

[17] Wendeborn S., Mondière R., Keller I., Nussbaumer H. (2012). Organocatalytic
Conversion of Ribose and Other Protected Carbohydrate Derivatives
into 2-Deoxy-lactones. Synlett.

[18] Cramer D. L., Bera S., Studer A. (2016). Exploring
Cooperative Effects in
Oxidative NHC Catalysis: Regioselective Acylation of Carbohydrates. Chem. - Eur. J..

[19] Behera P. C., Ramarao J., Suresh S. (2025). Design and Development of Carbene-Catalyzed
Intramolecular Vinylogous Stetter Reaction to Access Phenanthrol Derivatives. J. Org. Chem..

[20] Ding Y., Long X., Zhang J., Qu C., Wang P., Yang X., Puno P.-T., Deng J. (2025). Asymmetric
total synthesis
of penicilfuranone A through an NHC-catalyzed umpolung strategy. Chem. Sci..

[21] Kan T., Nara S., Ozawa T., Shirahama H., Matsuda F. (2000). SmI2-Induced Conversion of Carbohydrates
into Carbocycles. Angew. Chem. Int. Ed..

[22] Banachowicz P., Buda S. (2019). Gram-scale carbasugar
synthesis via intramolecular seleno-Michael/aldol
reaction. RSC Adv..

[23] Banachowicz P., Buda S. (2025). General strategy for the synthesis of unsaturated carbasugars via
a diastereoselective seleno-Michael/aldol reaction. RSC Adv..

[24] Banachowicz P., Mlynarski J., Buda S. (2018). Intramolecular Tandem Seleno-Michael/Aldol
Reaction: A Simple Route to Hydroxy Cyclo-1-ene-1-carboxylate Esters. J. Org. Chem..

[25] Hyldtoft L., Madsen R. (2000). Carbohydrate Carbocyclization
by a Novel Zinc-Mediated
Domino Reaction and Ring-Closing Olefin Metathesis. J. Am. Chem. Soc..

[26] Shing T. K. M., So K. H., Kwok W. S. (2009). Carbocyclization
of Carbohydrates:
Diastereoselective Synthesis of (+)-Gabosine F, (−)-Gabosine
O, and (+)-4-epi-Gabosine O. Org. Lett..

[27] Stockton K.
P., Greatrex B. W., Taylor D. K. (2014). Synthesis of allo- and epi-Inositol
via the NHC-Catalyzed Carbocyclization of Carbohydrate-Derived Dialdehydes. J. Org. Chem..

[28] Marco-Contelles J., de Opazo E. (2002). Synthesis of Enantiomerically
Pure, Highly Functionalized,
Medium-Sized Carbocycles from Carbohydrates: Formal Total Synthesis
of (+)-Calystegine B2. J. Org. Chem..

[29] Liu S.-L., Shi X.-X., Xu Y.-L., Xu W., Dong J. (2009). Asymmetric
syntheses of (−)-methyl shikimate and (−)-5a-carba-β-d-gulopyranose
from d-arabinose via Mukaiyama-type intramolecular aldolization. Tetrahedron: asymmetry.

[30] Hopkinson M. N., Richter C., Schedler M., Glorius F. (2014). An overview of N-heterocyclic
carbenes. Nature.

[31] Kireev A. S., Breithaupt A. T., Collins W., Nadein O. N., Kornienko A. (2005). Enantiodivergent
Formal Synthesis of (+)- and (−)-Cyclophellitol from d-Xylose
Based on the Latent Symmetry Concept. J. Org.
Chem..

[32] Bennett J. J., Murphy P. V. (2024). Flow Chemistry for Synthesis of 2-(C-Glycosyl)­acetates
from Pyranoses via Tandem Wittig and Michael Reactions. Org. Process Res. Dev..

[33] Reynolds N. T., Rovis T. (2005). The effect of pre-existing
stereocenters in the intramolecular asymmetric
Stetter reaction. Tetrahedron.

[34] Mattson A. E., Scheidt K. A. (2004). Catalytic Additions of Acylsilanes to Imines: An Acyl
Anion Strategy for the Direct Synthesis of α-Amino Ketones. Org. Lett..

[35] Wang M. H., Barsoum D., Schwamb C. B., Cohen D. T., Goess B. C., Riedrich M., Chan A., Maki B. E., Mishra R. K., Scheidt K. A. (2017). Catalytic, Enantioselective
β-Protonation through
a Cooperative Activation Strategy. J. Org. Chem..

[36] Dolhem F., Smiljanic N., Lièvre C., Demailly G. (2006). A straightforward synthesis
of glyco-2,7- and 2,8-dienes. Tetrahedron.

[37] Kerr M. S., Read de Alaniz J., Rovis T. (2002). A Highly Enantioselective Catalytic
Intramolecular Stetter Reaction. J. Am. Chem.
Soc..

[38] Kerr M. S., Rovis T. (2003). Effect of the Michael acceptor in the asymmetric intramolecular Stetter
reaction. Synlett.

