# Supplementary material for: Organocatalytic Stetter Cyclization of Pentoses for the Synthesis of Polyhydroxylated Cyclopentanone Scaffolds
Source: J Org Chem. 2025 Oct 1;90(40):14328–32. doi: 10.1021/acs.joc.5c01792 (PMC12519462; doi:10.1021/acs.joc.5c01792)
Supplement: Supplementary file 1 [file jo5c01792_si_001.pdf]

Organocatalytic Stetter Cyclization of Pentoses for the Synthesis of Polyhydroxylated Cyclopentanone Scaffolds

Christoph Suster, Nicolas Kratena, Kyryl Bocharov, Christian Stanetty*

Institute for Applied Synthetic Chemistry, TU Wien, Getreidemarkt 9, 1060 Vienna, Austria
*christian.stanetty@tuwien.ac.at

Supplementary Information

Inhaltsverzeichnis

A. General Information 4
B. Synthesis of Cyclisation Precursors 6
B.1. Xylose derived compounds 6
B.1.1. Synthesis of Methyl 2,3,4-tri-*O*-benzyl-β-D-xylopyranoside (4a) 6
B.1.2. Synthesis of 2,3,4-Tri-*O*-benzyl-D-xylopyranose (5a) 7
B.1.3. Synthesis of Methyl (4S,5R,6R)-4,5,6-tris(benzyloxy)-7-hydroxyhept-2-enoate (6a) 8
B.1.4. Synthesis of Methyl (4S, 5R, 6S, E)-4,5,6-tris(benzyloxy)-7-oxo-hept-2-enoate (1a) 10
B.1.5. Synthesis of Ethyl (4S,5R,6R)-4,5,6-tris(benzyloxy)-7-hydroxyhept-2-enoate (6f) 11
B.1.6. Synthesis of Ethyl (4S, 5R, 6S, E)-4,5,6-tris(benzyloxy)-7-oxo-hept-2-enoate (1f) 12
B.1.7. Synthesis of Benzyl (4S,5R,6R)-4,5,6-tris(benzyloxy)-7-hydroxyhept-2-enoate (6g) 13
B.1.8. Synthesis of Benzyl (4S, 5R, 6S, E)-4,5,6-tris(benzyloxy)-7-oxo-hept-2-enoate (1g) 14
B.1.9. Synthesis of Tert-butyl (4S,5R,6R)-4,5,6-tris(benzyloxy)-7-hydroxyhept-2-enoate (6h) 15
B.1.10. Synthesis of Tert. butyl (4S, 5R, 6S, E)-4,5,6-tris(benzyloxy)-7-oxo-hept-2-enoate (1h) 16
B.1.11. Synthesis of (4S,5R,6R,E)-4,5,6-tris(benzyloxy)-7-hydroxyhept-2-enenitrile (6i) 17
B.1.12. Synthesis of (4S,5R,6S,E)-4,5,6-tris(benzyloxy)-7-oxo-hept-2-enenitrile (1i) 18
B.1.13. Synthesis of Methyl (4S,5R,6R,E)-4,5,6,7-tetrahydroxyhept-2-enoate (4e) 19
B.1.14. Synthesis of Methyl (4S,5R,6S,E)-7-oxo-4,5,6-tris(trimethylsiloxy)hex-2-enoate (1e) 20
B.2. Lyxose Derived Compounds 21
B.2.1. Methyl D-lyxopyranoside (4b) 21
B.2.2. Synthesis of Methyl 2,3,4-tri-*O*-benzyl-α-D-lyxopyranoside (5b) 22
B.2.3. Synthesis of 2,3,4-Tri-*O*-benzyl-D-lyxopyranose (6b) 23
B.2.4. Synthesis of Methyl (4R,5R,6R)-4,5,6-tris(benzyloxy)-7-hydroxyhept-2-enoate (7b) 24
B.2.5. Synthesis of Methyl (4R,5R,6R, Z)-4,5,6-tris(benzyloxy)-7-oxo-hept-2-enoate (1b) 25
B.3. Ribose Derived Compounds 26
B.3.1. Synthesis of Methyl 2,3,4-tri-*O*-benzyl-β-D-ribopyranoside (4c) 26
B.3.2. Synthesis of 2,3,4-tri-*O*-benzyl-D-ribopyranose (5c) 27
B.3.3. Synthesis of Methyl (4S,5S,6R)-4,5,6-tris(benzyloxy)-7-hydroxyhept-2-enoate (6c) 28
B.3.4. Synthesis of Methyl (4S,5S,6R, E)-4,5,6-tris(benzyloxy)-7-oxo-hept-2-enoate (1c) 29
B.4. Arabinose Derived Compounds 30
B.4.1. Synthesis of Methyl 2,3,4-tri-*O*-benzyl-β-D-arabinopyranoside (4d) 30
B.4.2. Synthesis of Methyl (4S, 5S, 6S)-4,5,6-tris(benzyloxy)-7-hydroxyhept-2-enoate (6d) 31
B.4.3. Synthesis of Methyl (4S, 5S, 6S)-4,5,6-tris(benzyloxy)-7-oxo-hept-2-enoate (1d) 32
C. Cyclisations and Eliminations 33
C.1. General Procedures 33
C.1.1. General Procedure for Carbocylisation 33

C.1.2. General Procedure Elimination 33
C.1.3. One Pot Cylisation and Elimination 34
C.2. Xylose derived Carbocylcles 35
C.2.1. Methyl [(3S,4R,5S)-3,4,5-tris(benzyloxy)-2-oxocyclopentyl]acetate (2a) 35
C.2.2. Methyl [(3R,4S)-3,4-bis(benzyloxy)-5-oxocyclopent-1-en-1-yl]acetate (xylo / lyxo) (3a) 35
C.2.3. Ethyl [(3S,4R,5S)-3,4,5-tris(benzyloxy)-2-oxocyclopentyl]acetate (2f) 36
C.2.4. Ethyl [(3R,4S)-3,4-bis(benzyloxy)-5-oxocyclopent-1-en-1-yl]acetate (3f) 36
C.2.5. Benzyl [(3S,4R,5S)-3,4,5-tris(benzyloxy)-2-oxocyclopentyl]acetate (2g) 37
C.2.6. Benzyl [(3R,4S)-3,4-bis(benzyloxy)-5-oxocyclopent-1-en-1-yl]acetate (3g) 37
C.2.7. Tert. butyl [(3S,4R,5S)-3,4,5-tris(benzyloxy)-2-oxocyclopentyl]acetate (2h) 37
C.2.8. [(3R,4S)-3,4-bis(benzyloxy)-5-oxocyclopent-1-en-1-yl]acetonitrile (3i) 38
C.2.9. Methyl [(3S,4R,5S)-2-oxo-3,4,5-tris(trimethylsiloxy)cyclopentyl]acetate (2e) 38
C.3. Other Stereoconfigurations 39
C.3.1. Methyl [(3S,4R,5R)-3,4,5-tris(benzyloxy)-2-oxocyclopentyl]acetate (*Lyxo*) (2b) 39
C.3.2. Methyl [(1S,3S,4S,5S)-3,4,5-tris(benzyloxy)-2-oxocyclopentyl]acetate (*Ribo*) (2c) 40
C.3.3. Methyl [(3S,4S,5R)-3,4,5-tris(benzyloxy)-2-oxocyclopentyl]acetate (Arabino) (2d) 40
C.3.4. Methyl [(3S,4S)-3,4-bis(benzyloxy)-5-oxocyclopent-1-en-1-yl]acetate (Ribo / Arabino) (3c). 40
C.4. Prediction of syn/anti ratio between C1 and C5 41
D. Spectra 43
D.1. Xylose Derived Compounds 43
D.1.1. Methyl 2,3,4-Tri-*O*-benzyl-β-D-xylopyranoside (4a) 43
D.1.2. 2,3,4-tri-*O*-benzyl-β-D-xylopyranose (5a) 46
D.1.3. Methyl (4S,5R,6R,E)-4,5,6-tris(benzyloxy)-7-hydroxyhept-2-enoate (6a-e) 49
D.1.4. Methyl (4S,5R,6R,Z)-4,5,6-tris(benzyloxy)-7-hydroxyhept-2-enoate (6a-z) 51
D.1.5. Methyl (4S, 5R, 6S, E)-4,5,6-tris(benzyloxy)-7-oxohept-2-enoate (1a) 53
D.1.6. Ethyl (4S,5R,6R)-4,5,6-tris(benzyloxy)-7-hydroxyhept-2-enoate (6f) 55
D.1.7. Ethyl (4S, 5R, 6S, E)-4,5,6-tris(benzyloxy)-7-oxohept-2-enoate (1f-e) 58
D.1.8. Ethyl (4S, 5R, 6S, Z)-4,5,6-tris(benzyloxy)-7-oxohept-2-enoate (1f-z) 61
D.1.9. Benzyl (4S,5R,6R,E)-4,5,6-tris(benzyloxy)-7-hydroxyhept-2-enoate (6g-e) 64
D.1.10. Benzyl (4S,5R,6R,Z)-4,5,6-tris(benzyloxy)-7-hydroxyhept-2-enoate (6g-z) 67
D.1.11. Benzyl (4S, 5R, 6S, E)-4,5,6-tris(benzyloxy)-7-oxo-hept-2-enoate (1g) 70
D.1.12. Tert-butyl (4S,5R,6R,E)-4,5,6-tris(benzyloxy)-7-hydroxyhept-2-enoate (6h-e) 73
D.1.13. Tert-butyl (4S,5R,6R,Z)-4,5,6-tris(benzyloxy)-7-hydroxyhept-2-enoate (6h-z) 76
D.1.14. Tert. butyl (4S, 5R, 6S, E)-4,5,6-tris(benzyloxy)-7-oxohept-2-enoate (1h) 79
D.1.15. (4S,5R,6R)-4,5,6-tris(benzyloxy)-7-hydroxyhept-2-enenitrile (6i) 82
D.1.16. (4S,5R,6S)-4,5,6-tris(benzyloxy)-7-oxo-hept-2-enenitrile (1i) 84
D.1.17. Methyl (4S,5R,6R,E)-4,5,6,7-tetrahydroxyhept-2-enoate (4e) 86

D.1.18. Methyl (4S,5R,6S,E)-7-oxo-4,5,6-tris(trimethylsiloxy)hex-2-enoate (1e) 89
D.2. Lyxose derived Compounds 93
D.2.1. Methyl D-lyxopyranoside (4b) 93
D.2.2. Methyl 2,3,4-Tri-*O*-benzyl-α-D-lyxopyranoside (5b) 95
D.2.3. 2,3,4-Tri-*O*-benzyl-D-lyxopyranose (6b) 98
D.2.4. Methyl (4R,5R,6R, E)-4,5,6-tris(benzyloxy)-7-hydroxyhept-2-enoate (7b-e)....................... 101
D.2.5. Methyl (4R,5R,6R, Z)-4,5,6-tris(benzyloxy)-7-hydroxyhept-2-enoate (7b-z) 104
D.2.6. Methyl (4R,5R,6R, Z)-4,5,6-tris(benzyloxy)-7-oxo-hept-2-enoate (1b)............................... 107
D.3. Ribose derived Compounds 110
D.3.1. Methyl 2,3,4-tri-*O*-benzyl-β-D-ribopyranoside (4c) 110
D.3.2. 2,3,4-tri-*O*-benzyl-D-ribopyranose (5c) 113
D.3.3. Methyl (4S,5S,6R, E)-4,5,6-tris(benzyloxy)-7-hydroxyhept-2-enoate (6c-e)........................ 116
D.3.4. Methyl (4S,5S,6R, Z)-4,5,6-tris(benzyloxy)-7-hydroxyhept-2-enoate (6c-z) 119
D.3.5. Methyl (4S, 5S, 6S, E)-4,5,6-tris(benzyloxy)-7-oxohept-2-enoate (1c)................................ 122
D.4. Arabinose derived Compounds.. 125
D.4.1. Methyl 2,3,4-tri-*O*-benzyl-β-D-arabinopyranoside (4d) ....**Fehler! Textmarke nicht definiert.**
D.4.2. Methyl (4S, 5S, 6S)-4,5,6-tris(benzyloxy)-7-hydroxyhept-2-enoate (6d)...... **Fehler! Textmarke nicht definiert.**
D.4.3. Methyl (4S, 5S, 6S, E)-4,5,6-tris(benzyloxy)-7-oxo-hept-2-enoate (1d)........ **Fehler! Textmarke nicht definiert.**
D.5. Carbocycles ....**Fehler! Textmarke nicht definiert.**
D.5.1. Xylose derived Carbocycles...**Fehler! Textmarke nicht definiert.**
D.5.2. Other Stereoconfigurations ....**Fehler! Textmarke nicht definiert.**
E. LC-MS based Condition Screening.......................................**Fehler! Textmarke nicht definiert.**
E.1. Calibration ....**Fehler! Textmarke nicht definiert.**
E.2. Screenings..**Fehler! Textmarke nicht definiert.**
E.2.1. Catalyst screening...**Fehler! Textmarke nicht definiert.**
E.2.2. Other Screenings ....**Fehler! Textmarke nicht definiert.**
E.3. Influence of E/Z on diastereoselectivity and equilibration of diastereomeres........................... 174
F. References 174

A. General Information

Reagents and Solvents

All chemicals were directly used from commercial vendors without further purification. Water free solvents were obtained from a PureSolv EN 1-4 Enclosed solvent drying plant, or from commercial sources with "water-free" specification stored under argon and over molecular sieve.

TLC Analysis:

TLC for monitoring reactions and analysing fractions from column chromatography was performed on silica gel 60 F254-plates or HPTLC plates (silica gel 60 with concentration zone 20 × 2.5 cm). Visualisation was done using UV light (254 nm) followed by staining with an ethanolic anisaldehyde solution (180 mL EtOH, 10 mL anisaldehyde, 10 mL H_2SO_4 conc., 2 mL AcOH).

Column Chromatography

Column separation was performed on self-packed glass columns using silica gel from Merck (40-63 µm). MPLC separation was done on a Büchi Sephacore Flash (MPLC)-System consisting of two Büchi Pump Modules C-605, Büchi Pump Manager C-615, Büchi UV Photometer C-635 and a Büchi Fraction Collector C-660, or a Büchi Pure C850 FlashPrep-System with UV and ELS detector.
All indicated eluents were directly used from commercial sources.

NMR

NMR spectra were recorded on an Avance UltraShield 400 spectrometer (400 MHz machine) or a Bruker Avance III HD 600 spectrometer equipped with a prodigy N_2-cryo probe head (600 MHz machine). Spectra were calibrated to the solvent residual signal. Coupling constants (*J*) are given in Hz and chemical shifts (δ) in ppm. Assignments are based on COSY, HSQC and HMBC spectra and follow IUPAC nomenclature. For clarification of the numbering of carbocycles, see *Fehler! Verweisquelle konnte nicht gefunden werden.*.

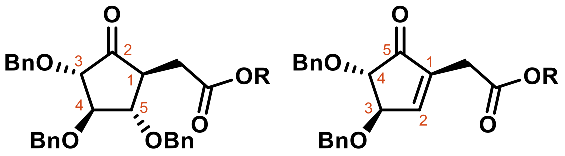

Figure S1. *IUPAC numbering of carbocycles disclosed in this article.*

In NMR-Codes of mixtures, the integrals of the minor compounds are scaled to 1:1 with the major compounds. The exact ratio is always indicated above, and in the NMR depiction (Chapter D)

Melting points:

Melting points were measured on a Büchi Melting Point B-545 system, with 40%/90% threshold detection and a heating rate of 0.5 °C/min

Optical rotation

Optical rotation was measured on an Anton Paar MCP 500 at the specified conditions, [α]D values are given in 10^{-1} deg cm^2 g^{-1}.

HPLC-MS

HPLC-MS analysis was performed on a Shimadzu LC-MS system equipped with a LC-40DXR solvent delivery module, a DGU-405 degassing unit, a CTO-40C column oven, an SPD-M40 Photodiode array detector, an ELSD-LTIII evaporative light scattering detector and an LCMS-2050 mass spectrometer. Method gradients were between acetonitrile and water with 0.1% formic acid. Reaction control and reaction optimisations were done on the columns indicated in the relevant sections.

HRMS (ESI+) m/z:

HR-MS analysis was performed using HTC PAL system auto sampler, an Agilent 1100/1200 HPLC and Agilent 6230 AJS ESI-TOF mass spectrometer. Data evaluation was performed using Agilent MassHunter

Qualitative Analysis B.07.00. Identification was based on peaks obtained from extracted ion chromatograms (extraction width ± 20 ppm).

Catalyst:

All catalysts were synthesized following literature known procedures.

Bicyclic triazolium catalysts (A), (B) and (C) were synthesised via a procedure by Rovis et al.[1], additionally, penta-fluoro aryl substituted catalyst (A) was also synthesised via an alternative procedure by Gravel et al.[2]

B. Synthesis of Cyclisation Precursors

B.1. Xylose derived compounds

B.1.1. Synthesis of Methyl 2,3,4-tri-O-benzyl-β-D-xylopyranoside (4a)

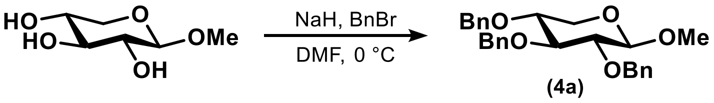

Commercially available Methyl-β-D-xylopyranoside (12.0 g, 70.2 mmol, 1.00 equiv.) was transferred into a 1000 mL three-neck flask equipped with a dropping funnel and a mechanical stirrer. The flask was set under argon atmosphere and 250 mL dry DMF were added via transfer cannula. The solution was cooled to 0 °C with an ice-bath. Upon reaching the desired temperature, NaH as 60% dispersion in paraffine oil (16.8 g, 421 mmol, 6.00 equiv.) was added under vigorous stirring, in three equal portions over 20 minutes. The solution was stirred for 30 min at 0 °C, leading to solidification of the mixture due to salt formation. Next, benzyl bromide (51.1 mL, 421 mmol, 6.00 equiv.) was added dropwise over 90 minutes. Upon complete addition, the ice bath was removed, and the mixture was allowed to warm to rt. The reaction was monitored via TLC (LP:EtOAc 4:1 and $CHCl_3$:MeOH:H_2O 14:7:1; anisaldehyde stain). After 3 h, TLC indicated complete consumption of starting material, and formation of a single product, therefore the reaction was again cooled to 0 °C with an ice bath, before 40 mL of MeOH were added slowly to quench the excess reagent. The mixture was stirred for further 15 min, before 400 mL EtOAc were added, and phases were separated, and the organic layer was washed 6 times with 100 mL H_2O each. The combined aqueous layers were once reextracted with 100 mL EtOAc, and the combined organic layers were dried over Na_2SO_4, filtered and evaporated (twice co-evaporated from toluene) to give the crude product as a colourless oil.
Cooling the crude material to approximately -10 °C, and addition of ice-cold hexane (~ 150 mL), initiated crystallisation of target material as white needles. The formed crystals were filtered off and washed with small amounts of cold hexane. The mother liquor was evaporated, and the crystallisation process was repeated two times, to give 22.4 g (73%) of target material (4a) in excellent purity (in three equally pure crystal fractions).

^{1}H NMR (600 MHz, Chloroform-d) δ 7.41 – 7.28 (m, 15H, Ar-H), 4.92 – 4.85 (m, 3H, C2-BnCH_2a, C3-BnCH_2ab), 4.76 (d, J = 11.6 Hz, 1H, C4-BnCH_2a), 4.73 (d, J = 11.1 Hz, 1H, C2-BnCH_2b), 4.65 (d, J = 11.6 Hz, 1H, C4-BnCH_2b), 4.28 (d, J = 7.6 Hz, 1H, H1), 3.97 (dd, J = 11.6, 5.1 Hz, 1H, H5a), 3.67 – 3.60 (m, 1H, H4), 3.60 (t, J = 8.7 Hz, 1H, H3), 3.56 (s, 3H, OCH_3), 3.38 (dd, J = 8.8, 7.5 Hz, 1H, H2), 3.24 (dd, J = 11.7, 9.7 Hz, 1H, H5b).

^{13}C{^{1}H} NMR (151 MHz, Chloroform-d) δ 138.8 (ArCH), 138.7 (ArCH), 138.3 (ArCH), 128.6 (2 × ArCH), 128.5 (4 × ArCH), 128.1 (2 × ArCH), 128.1 (2 × ArCH), 127.98 (ArCH4), 127.96 (2 × ArCH), 127.74 (ArCH4), 127.72 (ArCH4), 105.4 (C1), 83.8 (C3), 82.1 (C2), 78.0 (C4), 75.7 (BnCH_2(C4)), 75.0 (BnCH_2(C2)), 73.5 (BnCH_2(C3)), 64.0 (C5), 57.1 (OCH_3).

HRMS (ESI+) m/z: **[M + K]**$^+$ Calcd for $C_{27}H_{30}O_5K^+$: 473.1730; found 473.1736

$[\alpha_D^{20}]$ = +1.1° (c 1.0)

m.p. (hexane) 64.1 – 66.0 °C

Spectral data in accordance with literature[3]

B.1.2. Synthesis of 2,3,4-Tri-O-benzyl-D-xylopyranose (5a)

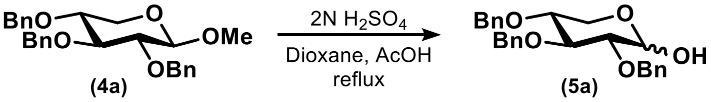

Methyl 2,3,4-tri-*O*-benzyl-β-D-xylopyranoside (4a) (11.8 g, 27.0 mmol, 1.00 equiv.) was suspended in 200 mL of a 1:1:1 mixture of acetic acid, dioxane and 1M H_2SO_4. The mixture was refluxed overnight, when after 15 h TLC (LP:EtOAc 3:1) indicated full consumption of starting material and formation of 2 new polar spots. The reaction was allowed to cool to rt, and the reaction mixture was 3 times extracted with EtOAc (100 mL each). Then, the combined organic layers were neutralised with sat. $NaHCO_3$ solution, dried over Na_2SO_4, filtered and evaporated, to give crude material as a colorless oil. Addition of ice-cold hexane (100 mL) gave a biphasic mixture, which was emulisifed by sonication. From this emulsion, white crystals started growing, upon cooling with an ice-bath. The crystallisation was completed at -18 °C over night. Two more crystalline fractions with equal purity were obtained by evaporating the mother liquor and adding new hexane. Overall, 8.1 g of pure target material (5a) were obtained as an anomeric mixture (α : β = 2:1).

^{1}H NMR (600 MHz, Chloroform-d) δ 7.38 – 7.27 (m, 30H, ArH), 5.12 (d, *J* = 3.5 Hz, 1H, A1), 4.92 – 4.87 (m, 1H, $BnCH_2a$-B2), 4.88 – 4.83 (m, 4H, $BnCH_2ab$-A3, $BnCH_2ab$-B3), 4.79 – 4.76 (m, 2H, $BnCH_2a$-A2, CH_2b-B2), 4.72 (dd, *J* = 11.7, 1.5 Hz, 2H, $BnCH_2a$-A4, $BnCH_2a$-B4), 4.69 – 4.66 (m, 2H, B1, $BnCH_2b$-A2), 4.64 (d, *J* = 11.7 Hz, 1H, $BnCH_2b$-A4), 4.63 (d, *J* = 11.6 Hz, 1H, $BnCH_2b$-B4), 3.98 – 3.93 (m, 1H, B5a), 3.87 (t, *J* = 8.7 Hz, 1H, A3), 3.80 (dd, *J* = 11.3, 10.1 Hz, 1H, A5a), 3.67 (dd, *J* = 11.2, 5.3 Hz, 1H, A5b), 3.65 – 3.58 (m, 2H, B3,B4), 3.55 (ddd, *J* = 10.3, 8.5, 5.3 Hz, 1H, A4), 3.49 (dd, *J* = 8.9, 3.5 Hz, 1H, A2), 3.35 – 3.25 (m, 2H, B2, B5b).

$^{13}C\{^{1}H\}$ NMR (151 MHz, Chloroform-d) δ 138.8 (ArC-(A)), 138.6 (ArC-(B)), 138.4 (ArC-(B)), 138.3 (ArC-(A)), 138.2 (ArC-(B)), 137.9 (ArC-(A)), 128.7 (2x ArCH-(A)), 128.6 (4 × ArCH-(B)), 128.59 (2x ArCH-(A)), 128.57 (2x ArCH-(B)), 128.50 (4x ArCH-(A, 4x ArCH-(B))), 128.23 (2x ArCH-(B)), 128.19 (2x ArCH-(A)), 128.16 (2x ArCH-(B)), 128.14 (2x ArCH-(A)), 128.11 (2x ArCH-(B)), 128.03 (ArCH-(B)), 127.97 (ArCH-(A)), 127.95 (ArCH-(A)), 127.92 (2 × ArCH-(A), ArCH-(B)), 127.84 (ArCH-(B)), 127.82 (ArCH-(A)), 97.9 (B1), 91.6 (A1), 83.3 (B3), 82.4 (B2), 80.6 (A3), 79.6 (A2), 77.6 (B4), 77.6 (A4), 75.7 ($BnCH_2$-A3), 75.6 ($BnCH_2$-B3), 75.0 ($BnCH_2$-B2), 73.6 ($BnCH_2$-A2), 73.40 ($BnCH_2$-B4), 73.35 ($BnCH_2$-A4), 63.9 (B5), 60.5 (A5).

HRMS (ESI+) m/z: $[M + K]^+$ Calcd for $C_{26}H_{28}O_5K$: 459.1573; found 459.1575

$[\alpha_D^{20}] = +15.7$ ° (c 1.0)

m.p. (hexane) 110-118 °C (non sharp melting point, due to anomeric mixture)

Spectral data in accordance with literature[3]

B.1.3. Synthesis of Methyl (4S,5R,6R)-4,5,6-tris(benzyloxy)-7-hydroxyhept-2-enoate (6a)

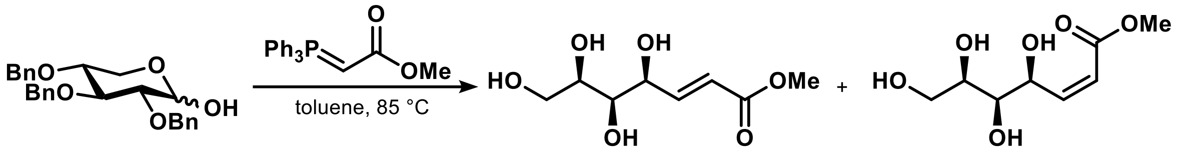

(5a) (6a-e) (6a-z)

Following a modified literature procedure[4]: Starting material (5a) (3.00 g, 7.13 mmol, 1.00 equiv.) was transferred into a 250 mL three-neck flask equipped with a condenser and a thermometer. Next, 100 mL of toluene were added, and the mixture was warmed to 85 °C (internal temperature). Upon reaching the desired temperature, Wittig reagent (7.30 g, 21.4 mmol, 3.00 equiv.) was added in one portion. The reaction was stirred at 85 °C and monitored via LC-MS (C8 column, H_2O:ACN 50% - 95% gradient). After 12, most of the starting material was converted into the desired material, but the reaction progress had stopped; therefore, another portion of reagent (2.43 g, 7.13 mmol, 1.00 equiv.) was added, which drove the reaction to completion within the next 5 h. The reaction was then cooled to rt, before volatiles were removed under reduced pressure. The residual oil was treated with an ice-cold mixture of hexane : Et_2O, until precipitation of triphenylphosphine oxide stopped. The white precipitate was filtered off, and the mother liquor was again evaporated to give crude material. Purification *via* column chromatography (MPLC, 180 g SiO_2, 60 mL/min, LP:EtOAc 6:1 – 3:1) gave pure (6a-z) and a mixture of (6a-z) and (6a-e). Overall, 2.81 g (83%) of target compound were obtained (E:Z 4:1).
A small analytical sample was further purified via column chromatography, to obtain both isomers separately as colorless oil:

<u>Analytical data (6a-e):</u>

^{1}H NMR (600 MHz, Chloroform-*d*) δ 7.32 – 7.23 (m, 15H, ArH), 6.92 (dd, *J* = 15.8, 5.8 Hz, 1H, H3), 6.03 (dd, *J* = 15.8, 1.3 Hz, 1H, H2), 4.65 (s, 2H, Bn-CH_2), 4.57 – 4.54 (m, 3H, Bn-CH_2a, Bn-CH_2), 4.35 (d, *J* = 11.6 Hz, 1H, Bn-CH_2b), 4.23 – 4.19 (m, 1H, H4), 3.70 (s, 3H, OCH_3), 3.69 – 3.66 (m, 1H, H7a), 3.66 – 3.63 (m, 1H, H5), 3.57 (q, *J* = 4.6 Hz, 1H, H6), 3.53 – 3.46 (m, 1H, H7b).

^{13}C{^{1}H} NMR (151 MHz, Chloroform-*d*) δ 166.49 (C1=O), 145.2 (C3), 138.7 (Ar-C1), 137.9 (Ar-C1), 137.4 (Ar-C1), 128.70 (2 × ArCH), 128.67 (2 × ArCH), 128.61 (2 × ArCH), 128.59 (2 × ArCH), 128.18 (2 × ArCH), 128.15 (ArC4), 128.1 (2 × ArC, ArC4), 128.0 (ArC4), 122.9 (C2), 80.8 (C5), 79.4 (C6), 78.3 (C4), 74.9 ($BnCH_2$), 73.0 ($BnCH_2$), 72.0 ($BnCH_2$), 61.5 (C7), 51.8 (OCH_3).

Spectral data in accordance with literature[4]

HRMS (ESI+) m/z: $[M + Na]^+$ Calcd for $C_{29}H_{32}O_6Na$ 499.2096; found 499.2112

$[\alpha_D^{20}]$ = +33.7 ° (c 1.0, $CHCl_3$)

<u>Analytical data (6a-z):</u>

^{1}H NMR (600 MHz, Chloroform-*d*) δ 7.51 – 7.03 (m, 15H, ArH), 6.35 (dd, *J* = 11.7, 8.2 Hz, 1H, H3), 5.78 (dd, *J* = 11.7, 1.2 Hz, 1H, H2), 5.17 – 5.10 (m, 1H, H4), 4.73 (d, *J* = 11.5 Hz, 2H, 2x $BnCH_2$a), 4.60 (d, *J* = 11.5 Hz, 1H, $BnCH_2$b), 4.56 (app. t, *J* = 12.2 Hz 2H, $BnCH_2$a, $BnCH_2$b), 4.32 (d, *J* = 11.8 Hz, 1H, $BnCH_2$b), 3.79 (s, 2H, H5, H6), 3.63 (s, 4H, OCH_3, H7a), 3.48 – 3.42 (m, 1H, H7b), 2.26 – 2.19 (m, 1H, -OH).

^{13}C{^{1}H} NMR (151 MHz, Chloroform-*d*) δ 166.3 (C1=O), 149.4 (C3), 138.7 (ArC1), 138.3 (ArC1), 137.6 (ArC1), 128.7 (2 × ArCH), 128.5 (2 × ArCH), 128.5 (4 × ArCH), 128.4 (2 × ArCH), 128.1 (ArC4), 128.0 (2 × ArCH), 127.9 (ArC4), 127.8 (ArC4), 121.7 (C2), 81.5 (C5/C6), 80.4 (C5/C6), 75.2 (Bn-CH_2), 74.1 (C4), 73.4 (Bn-CH_2), 71.7 (Bn-CH_2), 61.7 (C7), 51.7 (OCH_3).

HRMS (ESI+) m/z: $[M + Na]^+$ Calcd for $C_{29}H_{32}O_6Na$ 499.2096; found 499.2103

Additional Information: The step involving the all-equatorial xylo-configuration proved unreliable. Despite considerable efforts, column separation of the starting material (5a) from the target compounds was unachievable across several solvent systems. Thus, it is essential to ensure complete consumption of the starting material prior to final purification. Extended reaction times, however, promote a subsequent Oxa-Michael addition that leads to C-glycoside formation, thereby consuming the product.

Furthermore, we observed significant variability in C-glycoside formation between attempts. Our results indicate a strong dependency on reagent quality—specifically, residual base content appears to catalyse the unwanted Oxa-Michael addition. In instances where reagents yielded elevated amounts of C-glycosides, pretreatment with Amberlyst15 in toluene was beneficial. Additionally, strict temperature control is critical, as higher temperatures tend to favor C-glycoside formation.

B.1.4. Synthesis of Methyl (4S, 5R, 6S, E)-4,5,6-tris(benzyloxy)-7-oxo-hept-2-enoate (1a)

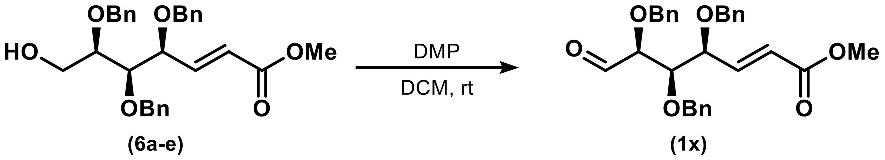

Starting material (6a-e) (1.6 g, 3.36 mmol, 1.00 equiv.) was dissolved in 10 mL of DCM, to which Dess-Martin periodinane (DMP) (1.76 g, 4.03 mmol, 1.20 equiv.) was added in one portion. The mixture turned cloudy immediately. After 5 min of stirring at rt, TLC (LP:EtOAc 2:1, anisaldehyde stain) indicated full consumption of starting material, and formation of two more apolar spots.
Next, 10 mL of sat. $NaHCO_3$ solution were added, and the mixture was vigorously stirred for 20 min. The mixture was filtered through a plug of cotton, to remove white precipitate (presumably IBX). bThe phases were then separated, and the aqueous phase was 3 times extracted with 30 mL of DCM each. The combined organic layers were dried over Na_2SO_4, filtered and evaporated to give crude material, which was directly submitted to column chromatography (MPLC, 90 g SiO2, 60 mL/min, LP:EtOAc 4:1 – 1:1), to give 1.36 g (85%) of pure target material (1a) as a colorless oil.

^{1}H NMR (400 MHz, Chloroform-*d*) δ 9.66 (d, *J* = 0.8 Hz, 1H, CHO), 7.38 – 7.27 (m, 13H, ArH), 7.25 – 7.20 (m, 2H, ArH), 6.89 (dd, *J* = 15.8, 6.1 Hz, 1H, H3), 6.03 (dd, *J* = 15.8, 1.4 Hz, 1H, H2), 4.73 (d, *J* = 11.8 Hz, 1H, $BnCH_2a$), 4.62 (d, *J* = 11.8 Hz, 1H, $BnCH_2a$), 4.57 (d, *J* = 11.8 Hz, 1H, $BnCH_2b$), 4.50 (dd, *J* = 11.6, 1.4 Hz, 2H, $BnCH_2a$, $BnCH_2b$), 4.40 (d, *J* = 11.4 Hz, 1H, Bn-CH_2b), 4.28 (ddd, *J* = 6.0, 4.6, 1.4 Hz, 1H, H4), 3.89 (dd, *J* = 4.4, 0.8 Hz, 1H, H6), 3.83 (t, *J* = 4.5 Hz, 1H, H5), 3.75 (s, 3H, OCH_3).
$^{13}C\{^1H\}$ NMR (101 MHz, Chloroform-*d*) δ 201.3 (CHO), 166.3 (COOMe), 144.7 (C3), 137.24 (ArC1), 137.18 (ArC1), 137.0 (ArC1), 128.7 (2 × ArCH), 128.58 (2 × ArCH), 128.55 (4 × ArCH), 128.4 (2 × ArCH), 128.3 (2 × ArC, ArC4), 128.2 (ArC4), 128.1 (ArC4), 123.3 (C2), 81.9 (C5), 80.9 (C6), 77.5 (C4), 74.4 ($BnCH_2$), 73.5 ($BnCH_2$), 72.2 ($BnCH_2$), 51.8 (OCH_3).
HRMS (ESI+) m/z: $[M + Na]^+$ Calcd for $C_{29}H_{30}O_6Na$: 497.1934; found 497.1936
Spectral data in accordance with literature[4]

B.1.5. Synthesis of Ethyl (4S,5R,6R)-4,5,6-tris(benzyloxy)-7-hydroxyhept-2-enoate (6f)

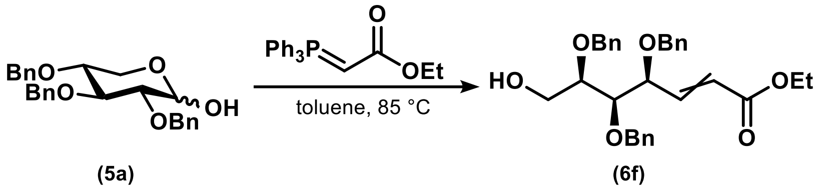

Starting material (5a) (1.51 g, 3.60 mmol, 1.00 equiv.) was transferred into a three-neck flask equipped with a thermometer and a reflux condenser. First, 40 mL of toluene, then before Wittig reagent (3.68 g, 10.8 mmol, 3.00 equiv.) was added. The flask was placed into a preheated oilbath at 95 °C, and the reaction was stirred for 18 h at that temperature. After that time, TLC (LP:EtOAc 4:1) indicated full consumption of starting material. LC-MS (C8 column, H_2O:ACN 50% - 95% gradient) further showed, that only insignificant amounts of Oxa-Michael byproducts were formed. The reaction was allowed to cool to rt before the toluene was removed under reduced pressure. The residue was subjected to column chromatography (MPLC, dry loaded onto 10 g Celite, 90 g SiO2, cyclohexane:EtOAc 4:1 isocratic) to give 962 mg (54%) of target compound (6f) as E/Z mixture (4:1) according to NMR as colorless oil.

^{1}H NMR (600 MHz, Chloroform-*d*) δ 7.31 – 7.23 (m, 30H, Ar-H), 6.92 (dd, *J* = 15.8, 5.9 Hz, 1H, Z-H3), 6.32 (dd, *J* = 11.7, 8.2 Hz, 1H, E-H3), 6.02 (dd, *J* = 15.8, 1.4 Hz, 1H, Z-H2), 5.76 (dd, *J* = 11.7, 1.2 Hz, 1H, E-H2), 5.13 (d, *J* = 8.1 Hz, 1H, Z-H4), 4.71 (d, *J* = 11.2 Hz, 1H, 2 × Z-BnCH$_2$a), 4.65 (s, 2H, E-BnCH$_2$), 4.59 (d, *J* = 12.7 Hz, 1H, E-BnCH$_2$a), 4.56 (s, 3H, E-BnCH$_2$), 4.54 (d, *J* = 11.5 Hz, 1H, 2x Z-BnCH$_2$b), 4.35 (d, *J* = 11.7 Hz, 1H, E-BnCH$_2$b), 4.30 (d, *J* = 11.8 Hz, 1H, Z-BnCH$_2$b), 4.22 – 4.20 (m, 1H, Z-H4), 4.16 (qd, *J* = 7.1, 2.3 Hz, 2H, E-OEt-CH$_2$), 4.08 (q, *J* = 7.1 Hz, 2H, Z-OEt-CH$_2$), 3.78 (t, *J* = 2.5 Hz, 2H, Z-H5, Z-H6), 3.69 – 3.60 (m, 3H, E-H5, E-H7a, Z-H7a), 3.57 (q, *J* = 4.5 Hz, 1H, E-H6), 3.49 (dd, *J* = 11.8, 4.3 Hz, 1H, E-H7b), 3.44 (dd, *J* = 8.9, 3.4 Hz, 1H, Z-H7b), 1.26 (t, *J* = 7.1 Hz, 3H, E-OEt-CH$_3$), 1.20 (t, *J* = 7.1 Hz, 3H, Z-OEt-CH$_3$). *Integrals of Z-compound scaled to match E-compound

^{13}C{^{1}H} NMR (151 MHz, Chloroform-*d*) δ 166.1 (E-C=O), 165.9 (Z-C=O), 149.0 (Z3), 144.8 (E3), 138.7 (Z-ArC1), 138.3 (E-ArC1, E-ArC1), 137.9 (E-ArC1), 137.6 (Z-ArC1), 137.5 (E-ArC1), 128.71 (ArCH), 128.69 (2 × ArCH), 128.64 (2 × ArCH), 128.58 (2 × ArCH), 128.57 (2 × ArCH), 128.52 (ArCH), 128.51 (ArCH), 128.4 (ArCH), 128.2 (2 × ArCH), 128.11 (4 × ArCH), 128.07 (ArCH), 128.05 (ArCH), 127.94 (2 × ArCH), 127.90 (ArCH), 127.80 (ArCH), 123.4 (E2), 122.2 (E2), 81.5 (Z5/6), 80.8 (E5), 80.4 (Z5/6), 79.5 (E6), 78.4 (E4), 75.3 (Z-BnCH$_2$), 74.9 (E-BnCH$_2$), 74.0 (Z4), 73.4 (Z-BnCH$_2$), 73.0 (E-BnCH$_2$), 71.9 (E-BnCH$_2$), 71.7 (Z-BnCH$_2$), 61.7 (Z7), 61.5 (E7), 60.7 (E-OEt-CH$_2$), 60.6 (Z-OEt-CH$_2$), 14.40 (E-OEt-CH$_3$), 14.3 (Z-OEt-CH$_3$).

HRMS (ESI+) m/z: $[M + Na]^+$ Calcd for $C_{30}H_{34}O_6Na^+$: 513.2259 found 513.2267

Spectral data for both isomers in accordance with literature[5,6]

B.1.6. Synthesis of Ethyl (4S, 5R, 6S, E)-4,5,6-tris(benzyloxy)-7-oxo-hept-2-enoate (1f)

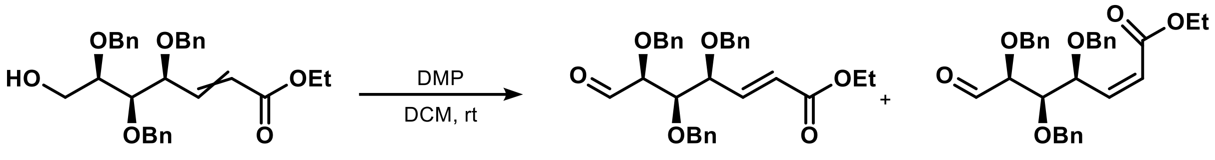

An E/Z mixture of (6f) (E:Z = 4:1) (340 mg, 0.658 mmol, 1.00 equiv.) was dissolved in 10 ml of DCM, before Dess-Martin-Periodinane (DMP) (374 mg, 0.856 mmol, 1.30 equiv.) was added in one portion. The mixture was stirred at rt for 20 min, when TLC (LP:EtOAc 4:1, anisaldehyde stain) indicated complete consumption of starting material, and formation of a new more apolar spot. 10 mL of sat. $NaHCO_3$ solution was added to the reaction, and then stirring was continued for a further 20 min. The mixture was filtered through a plug of cotton to remove the white precipitate (presumably IBX) before phases were separated, and the aqueous phase was 3 times extracted with 20 mL of DCM each. The combined organic layers were dried over Na_2SO_4, filtered and evaporated to give crude material, that was directly submitted to column chromatography (MPLC, 45 g SiO2, 60 mL/min, LP:EtOAc 4:1 – 1:1), to give 231 mg of pure E compound (1f-e), and 75 mg of Z enriched E/Z mixture (15:85) (6f-z) both as colorless oil. Combined yield: 306 mg (95%)

Analytical data for E-compound (1f-e)
^{1}H NMR (600 MHz, Chloroform-*d*) δ 9.65 (d, *J* = 0.6 Hz, 1H, CHO), 7.39 – 7.18 (m, 15H, ArH), 6.89 (dd, *J* = 15.8, 6.2 Hz, 1H, H3), 6.01 (dd, *J* = 15.8, 1.4 Hz, 1H, H2), 4.73 (d, *J* = 11.8 Hz, 1H, $BnCH_2$a), 4.62 (d, *J* = 11.8 Hz, 1H, $BnCH_2$a), 4.57 (d, *J* = 11.8 Hz, 1H, $BnCH_2$b), 4.50 (d, *J* = 11.6 Hz, 2H, $BnCH_2$a, $BnCH_2$b), 4.39 (d, *J* = 11.4 Hz, 1H, $BnCH_2$b), 4.28 (ddd, *J* = 6.0, 4.6, 1.3 Hz, 1H, H4), 4.20 (qd, *J* = 7.1, 1.8 Hz, 2H, OEt-CH_2), 3.88 (dd, *J* = 4.5, 0.7 Hz, 1H, H6), 3.82 (t, *J* = 4.5 Hz, 1H, H5), 1.30 (t, *J* = 7.1 Hz, 3H, OEt-CH_3).
^{13}C{^{1}H} NMR (151 MHz, Chloroform-*d*) δ 201.4 (CHO), 165.9 (**C**OOEt), 144.3 (C2), 137.3 (ArC1), 137.2 (ArC1), 137.1 (ArC1), 128.70 (2 × ArCH), 128.60 (2 × ArCH), 128.56 (2 × ArCH), 128.55 (2 × ArCH), 128.5 (2 × ArCH), 128.4 (ArC4), 128.34 (2 × ArCH), 128.25 (ArC4), 128.0 (ArC4), 123.8 (C3), 81.9 (C6), 80.9 (C5), 77.6 (C4), 74.4 ($BnCH_2$), 73.5 ($BnCH_2$), 72.1 ($BnCH_2$), 60.7 (OEt-CH_2), 14.4 (OEt-CH_3).
HRMS (ESI+) m/z: $[M + Na]^+$ Calcd for $C_{30}H_{32}O_6Na$: 511.2102 found 511.2118

Analytical data for Z-compound (1f-z)
^{1}H NMR (600 MHz, Chloroform-*d*) δ 9.72 (d, *J* = 0.7 Hz, 1H, CHO), 7.37 – 7.21 (m, 15H, ArH), 6.32 (dd, *J* = 11.8, 8.3 Hz, 1H, H3), 5.81 (d, *J* = 1.3 Hz, 1H, H2), 5.35 (ddd, *J* = 8.3, 3.0, 1.3 Hz, 1H, $BnCH_2$), 4.74 (d, *J* = 12.0 Hz, 1H, $BnCH_2$a), 4.63 (d, *J* = 12.0 Hz, 1H, $BnCH_2$b), 4.60 (d, *J* = 11.9 Hz, 1H, $BnCH_2$a), 4.56 (d, *J* = 11.7 Hz, 1H, $BnCH_2$b), 4.45 (d, *J* = 2.8 Hz, 2H, $BnCH_2$), 4.11 (q, *J* = 7.1 Hz, 2H, OEt-CH_2), 4.03 (dd, *J* = 5.6, 3.1 Hz, 1H, H6), 3.99 – 3.93 (m, 1H, H5), 1.23 (t, *J* = 7.1 Hz, 3H, OEt-CH_3).
^{13}C{^{1}H} NMR (151 MHz, Chloroform-*d*) δ 200.5 (CHO), 165.7 (**C**OOEt), 147.7 (C2), 137.6 (Ar-C1), 137.5 (2 × ArC1), 128.58 (4 × ArCH), 128.55 (2 × ArCH), 128.44 (2 × ArCH), 128.36 (2 × ArCH), 128.2 (2 × ArCH), 128.13 (ArC4), 128.07 (ArC4), 127.9 (ArC4), 122.4 (C3), 81.5 (C5), 81.2 (C6), 74.2 ($BnCH_2$), 73.8 (C4), 73.0 ($BnCH_2$), 72.2 ($BnCH_2$), 60.5 (OEt-CH_2), 14.3 (OEt-CH_3).
*NMR Signals extracted from a mixture of (1f-e) and (1f-z) (15:85)
Spectral data for both isomers in accordance with literature[6]

B.1.7. Synthesis of Benzyl (4S,5R,6R)-4,5,6-tris(benzyloxy)-7-hydroxyhept-2-enoate (6g)

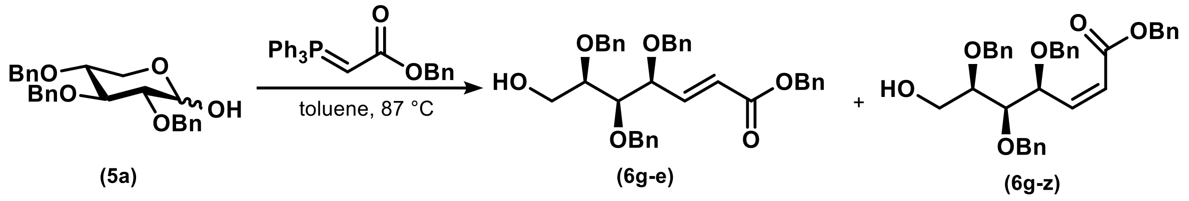

Starting material (5a) (750 mg, 1.78 mmol, 1.00 equiv.) was transferred into a three-neck flask equipped with a thermometer and a reflux-condenser. Next, the solid was dissolved in 30 mL of toluene, before Wittig ylide (2.31 g, 5.35 mmol, 3.00 equiv.) was added, and the flask was put into a preheated oil-bath (95 °C). The reaction was stirred at 87 °C internal temperature and was monitored via LC-MS (C8, column, H_2O:ACN 50% - 95%). After 16 hrs, about 50% of the starting material was converted into mostly target material, but also substantial amounts of Oxa-michael byproduct was observed (~10%). To drive the reaction to completion, another portion of Wittig reagent (770 mg, 1.78 mmol, 1.00 equiv.) was added, and the reaction was upheld over night. After 38 hrs, most of the starting material about 80% of starting material were converted, but the ratio between target material and Oxa-Michael byproduct was trending towards by-product. Therefore, the reaction was stopped, by allowing it to cool to rt. Next, toluene was evaporated under reduced pressure, and the residue was submitted to column chromatography (24 g 40µm SiO_2, 32 ml/min, hexane: EtOAc 6:1 – 2:1), to give pure 105 mg pure Z-compound (6g-e) in the leading fractions, and 418 mg of an inseparable mixture of E-compound (6g-e) and starting material (5a) (9:1), both as colorless oils (Overall yield 49%)

Analytics of (6g-e) (from mixture with 5a):
^{1}H NMR (600 MHz, Chloroform-*d*) δ 7.38 – 7.23 (m, 20H, ArH), 7.00 (dd, *J* = 15.8, 5.9 Hz, 1H, H3), 6.10 (dd, *J* = 15.8, 1.4 Hz, 1H, H2), 5.21 – 5.11 (m, 2H, OBn-CH_2), 4.66 (d, *J* = 2.0 Hz, 2H, BnCH_2), 4.60 – 4.54 (m, 3H, BnCH_2, BnCH_2a), 4.37 (d, *J* = 11.7 Hz, 1H, BnCH_2b), 4.26 – 4.22 (m, 1H, H4), 3.70 – 3.65 (m, 2H, H5, H7a), 3.61 – 3.57 (m, 1H, H6), 3.54 – 3.49 (m, 1H, H7b), 2.09 (t, *J* = 6.1 Hz, 1H, OH).
^{13}C{^{1}H} NMR (151 MHz, Chloroform-*d*) δ 165.7 (COOBn), 145.5 (C3), 138.1 (ArC1), 137.7 (ArC1), 137.3 (ArC1), 135.8 (COOBn-ArC1), 128.6 (2 × ArCH), 128.51 (2 × ArCH), 128.48 (2 × ArCH), 128.42 (2 × ArCH), 128.41 (2 × ArCH), 128.28 (2 × ArCH), 128.27 (ArC4), 128.02 (2 × ArCH), 127.96 (ArC4), 127.94 (2 × ArC, ArC4), 127.8 (ArC4), 122.8 (C2), 80.6 (C5), 79.3 (C6), 78.3 (C4), 74.7 (BnCH_2), 72.9 (BnCH_2), 71.9 (BnCH_2), 66.4 (COOBn-CH_2), 61.32 (C7).
HRMS (ESI+) m/z: [M + Na]$^+$ Calcd for $C_{35}H_{36}O_6Na$: 575.2404; found 575.2413

Analytics of (6g-z):
^{1}H NMR (600 MHz, Chloroform-*d*) δ 7.42 – 7.18 (m, 20H, ArH), 6.38 (dd, *J* = 11.7, 8.2 Hz, 1H, H3), 5.82 (dd, *J* = 11.7, 1.1 Hz, 1H, H2), 5.18 (d, *J* = 7.9 Hz, 1H, H4), 5.10 (s, 2H, COOBn-CH_2), 4.75 (d, *J* = 11.5 Hz, 1H, BnCH_2a), 4.73 (d, *J* = 11.4 Hz, 1H, BnCH_2a), 4.62 (d, *J* = 11.5 Hz, 1H, BnCH_2b), 4.58 (d, *J* = 12.3 Hz, 1H, BnCH_2a), 4.56 (d, *J* = 11.7 Hz, 1H, BnCH_2b), 4.33 (d, *J* = 11.8 Hz, 1H, BnCH_2b), 3.85 – 3.67 (m, 2H, H5, H6), 3.66 – 3.60 (m, 1H, H7a), 3.48 – 3.37 (m, 1H, H7b), 2.21 (s, 1H, OH).
^{13}C{^{1}H} NMR (151 MHz, Chloroform-*d*) δ 165.65 (COOBn), 149.78 (C3), 138.65 (ArC1), 138.22 (ArC1), 137.60 (ArC1), 135.81 (COOBn-ArC1), 128.75 (4 × ArCH), 128.54 (2 × ArCH), 128.53 (2 × ArCH), 128.51 (4 × ArCH), 128.38 (ArC4, 2 × ArCH), 128.07 (ArC4), 128.06 (2 × ArCH), 127.92 (ArC4), 127.79 (ArC4), 121.70 (C2), 81.34 (C5), 80.39 (C6), 75.18 (BnCH_2), 74.03 (C4), 73.38 (BnCH_2), 71.75 (BnCH_2), 66.41 (COOBn-CH_2), 61.76 (C7).
HRMS (ESI+) m/z: [M + Na]$^+$ Calcd for [$C_{35}H_{36}O_6$]Na^+: 575.2404 found 575.2412

B.1.8. Synthesis of Benzyl (4S, 5R, 6S, E)-4,5,6-tris(benzyloxy)-7-oxo-hept-2-enoate (1g)

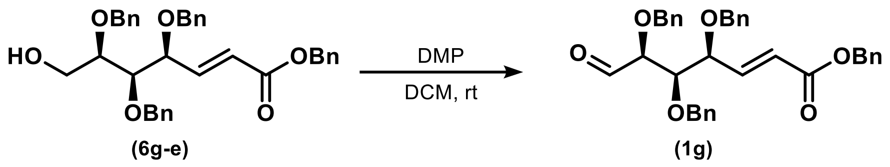

Starting material (6g-e) (110 mg, 0.179 mmol, 1.00 equiv., 90% purity), was dissolved in 2 mL of DCM, before DMP (94 mg, 0.215 mmol, 1.20 equiv.). The reaction was stirred at rt, when TLC (LP:EtOAc 4:1, anis aldehyde stain) confirmed completion of the reaction. The reaction mixture was filtered to remove the white precipitate (rinsed with DCM), next all volatiles were removed under reduced pressure, and the residue was directly submitted to column chromatography (12 g SiO2, hexane: EtOAc 6:1 – 3:1, 18 mL/min) to give 65 mg of pure target material (1g) as colorless oil (66% yield).

^{1}H NMR (600 MHz, Chloroform-*d*) δ 9.62 (s, 1H, CHO), 7.40 – 7.14 (m, 20H, ArH), 6.90 (dd, *J* = 15.8, 6.1 Hz, 1H, H3), 6.03 (dd, *J* = 15.9, 1.4 Hz, 1H, H2), 5.18 (d, *J* = 12.4 Hz, 1H, COOBn-CH_2a), 5.15 (d, *J* = 12.4 Hz, 1H, COOBn-CH_2b), 4.70 (d, *J* = 11.8 Hz, 1H, BnCH_2a), 4.58 (d, *J* = 11.8 Hz, 1H, BnCH_2a), 4.54 (d, *J* = 11.8 Hz, 1H, BnCH_2b), 4.46 (d, *J* = 11.5 Hz, 2H, BnCH_2a, BnCH_2b), 4.36 (d, *J* = 11.4 Hz, 1H, BnCH_2b), 4.26 (ddd, *J* = 5.9, 4.5, 1.3 Hz, 1H, H4), 3.86 (d, *J* = 4.5 Hz, 1H, H6), 3.78 (t, *J* = 4.4 Hz, 1H, H5).
^{13}C{^{1}H} NMR (151 MHz, Chloroform-*d*) δ 201.24 (CHO), 165.67 (COOBn), 145.08 (C3), 137.18 (ArC1), 137.16 (ArC1), 137.02 (ArC1), 135.98 (COOBn-ArC1), 128.71 (2 × ArCH), 128.69 (2 × ArCH), 128.58 (2 × ArC, ArC1), 128.55 (4 × ArCH), 128.41 (2 × ArC, ArC4), 128.39 (ArC4), 128.36 (2 × ArCH), 128.2 (ArC4), 128.1 (ArC4), 123.3 (C2), 81.7 (C6), 80.8 (C5), 77.5 (C4), 74.5 (BnCH_2), 73.5 (BnCH_2), 72.2 (BnCH_2), 66.5 (COOBn-CH_2).
HRMS (ESI+) m/z: $[M + Na]^+$ Calcd for $C_{35}H_{34}O_7Na$: 589.2202; found 589.2212 *(This compound ionised as its corresponding acid during several HRMS runs, while NMR gave no indications for over-oxidation of the aldehyde moiety before and after the HRMS measurements; Further, carbocyclisation proceeded smoothly with the batch the HRMS measurements were taken from)*

B.1.9. Synthesis of Tert-butyl (4S,5R,6R)-4,5,6-tris(benzyloxy)-7-hydroxyhept-2-enoate (6h)

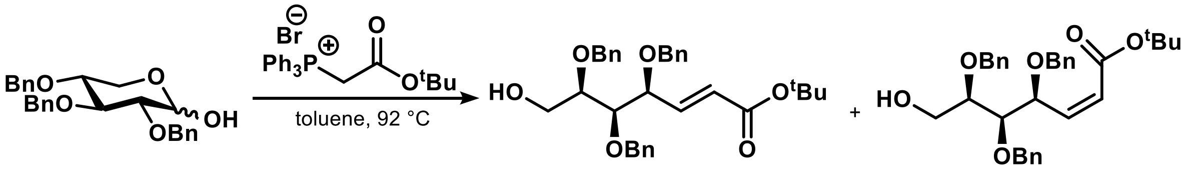

Wittig salt (1.66 g, 3.55 mmol, 3.00 equiv.) was dissolved in 15 mL of $CHCl_3$ and was twice extracted with 10 mL of 2N NaOH. The combined aqueous layers were re-extracted with 10 mL of $CHCl_3$. The combined organic layers were dried over Mg_2SO_4, filtered and evaporated directly in a three-neck flask.
The residual goo was dissolved in 15 mL of toluene, and sugar benzyl-xylose (5a) (498 mg, 1.18 mmol, 1.00 equiv.) was added immediately. The flask was equipped with a reflux condenser and a thermometer before it was put into a preheated oil bath. The reaction was stirred at 92 °C internal temperature for 22 hrs, when TLC (LP:EtOAc 4:1) and LC-MS (C8 column, 50% - 95% ACN) showed complete consumption of starting material, and predominant formation of target material (6h), with small amounts (<10%) of Oxa-Michael side products. The reaction was allowed to cool to rt, before toluene was removed under reduced pressure. The residual oil, was submitted to column chromatography (MPLC, dry loading in 5 g celite, 20 g SiO_2, cyclohexane : EtOAc 4:1 isocratic) to give 276 mg of pure E isomer, and 57 mg of pure Z isomer as colorless oils (combined yield 54%).
Analytical data (6h-e):
^{1}H NMR (600 MHz, Chloroform-*d*) δ 7.36 – 7.27 (m, 15H, ArH), 6.88 (dd, *J* = 15.8, 6.2 Hz, 1H, H3), 5.99 (dd, *J* = 15.8, 1.3 Hz, 1H, H2), 4.70 (s, 2H, $BnCH_2$), 4.62 (d, *J* = 11.8 Hz, 1H, $BnCH_2$a), 4.61 (s, 2H, $BnCH_2$), 4.39 (d, *J* = 11.7 Hz, 1H, $BnCH_2$b), 4.25 (ddd, *J* = 6.2, 4.9, 1.3 Hz, 1H, H4), 3.73 – 3.67 (m, 2H, H7a, H5), 3.64 – 3.61 (m, 1H, H6), 3.53 (dd, *J* = 11.9, 4.4 Hz, 1H, H7b), 1.50 (s, 8H, tBu-CH_3).
^{13}C{^{1}H} NMR (151 MHz, Chloroform-*d*) δ 165.4 (COOtBu), 143.5 (C3), 138.3 (ArC1), 138.0 (ArC1), 137.6 (ArC1), 128.67 (2 × ArCH), 128.62 (2 × ArCH), 128.59 (2 × ArCH), 128.56 (2 × ArCH), 128.2 (2 × ArCH), 128.09 (2 × ArC, ArC4), 128.07 (ArC4), 127.9 (ArC4), 125.4 (C2), 81.0 (C5), 79.5 (C6), 78.5 (C4), 74.9 ($BnCH_2$), 73.1 ($BnCH_2$), 71.9 ($BnCH_2$), 61.5 (OtBu-C), 28.3 (3 × OtBu-CH_3).
HRMS (ESI+) m/z: $[M + K]^+$ Calcd for $C_{33}H_{36}O_6K$: 557.2311; found 557.2317
Analytical data (6h-z):
^{1}H NMR (600 MHz, Chloroform-*d*) δ 7.38 – 7.27 (m, 15H, ArH), 6.30 (dd, *J* = 11.8, 8.2 Hz, 1H, H3), 5.75 (dd, *J* = 11.8, 1.2 Hz, 1H, H2), 5.16 (d, *J* = 8.1 Hz, 1H, H4), 4.78 (d, *J* = 11.5 Hz, 2H, $BnCH_2$a), 4.77 (d, *J* = 11.4 Hz, 2H, $BnCH_2$a), 4.66 – 4.59 (m, 3H, 2 × $BnCH_2$b, $BnCH_2$a), 4.36 (d, *J* = 11.8 Hz, 1H, $BnCH_2$b), 3.85 – 3.81 (m, 2H, H5, H6), 3.67 (dd, *J* = 12.8, 2.2 Hz, 1H, H7a), 3.50 – 3.46 (m, 1H, H7b), 1.46 (s, 9H, OtBu-CH_3).
^{13}C{^{1}H} NMR (151 MHz, Chloroform-*d*) δ 165.4 (COOtBu), 147.6 (C3), 138.7 (ArC1), 138.3 (ArC1), 137.6 (ArC1), 128.7 (2 × ArCH), 128.6 (2 × ArCH), 128.5 (2 × ArCH), 128.5 (2 × ArCH), 128.4 (2 × ArCH), 128.1 (2 × ArCH), 128.0 (ArC4), 127.8 (ArC4), 127.7 (ArC4), 124.0 (C2), 81.5 (C5/6), 80.5 (C5/6), 75.2 ($BnCH_2$), 73.5 (C4), 73.4 ($BnCH_2$), 71.5 ($BnCH_2$), 61.7 (OtBu-C), 28.2 (3 × OtBu-CH_3).
HRMS (ESI+) m/z: $[M + K]^+$ Calcd for $C_{32}H_{36}O_6K$: 557.2311; found 557.2319

B.1.10. Synthesis of Tert. butyl (4S, 5R, 6S, E)-4,5,6-tris(benzyloxy)-7-oxo-hept-2-enoate (1h)

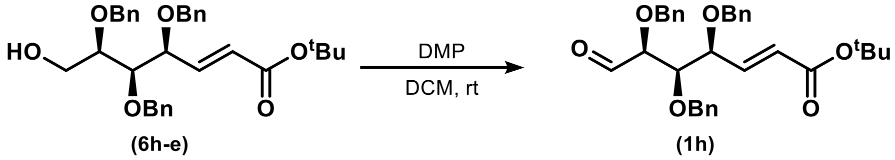

Starting material (6h-e) (70.0 mg, 0.128 mmol, 1.00 equiv.), as pure E-compound was dissolved in DCM (3 mL). DMP (76.7 mg, 0.175 mmol, 1.30 equiv.) was added in one portion. The mixture was stirred at rt for 10 min, before TLC (LP:EtOAc 4:1) showed completion of the reaction. 5 mL of sat. $NaHCO_3$ solution was added to the reaction, and then stirring was continued for a further 20 min. The mixture was filtered through a plug of cotton to remove the white precipitate before phases were separated, and the aqueous phase was 3 times extracted with 20 mL of DCM each. The combined organic layers were dried over Na_2SO_4, filtered and evaporated to give crude material, that was directly submitted to column chromatography (MPLC, 12 g SiO2, 18 mL/min, LP:EtOAc 4:1 – 2:1), to give 49 mg (70%) of pure E compound (1h) as colorless oil.

^{1}H NMR (600 MHz, Chloroform-*d*) δ 9.64 (d, J = 0.6 Hz, 1H, A), 6.80 (dd, J = 15.8, 6.5 Hz, 1H, H3), 5.93 (dd, J = 15.8, 1.3 Hz, 1H, H2), 4.72 (d, J = 11.8 Hz, 1H, $BnCH_2$a), 4.63 (d, J = 11.8 Hz, 1H, $BnCH_2$a), 4.57 (d, J = 11.8 Hz, 1H, $BnCH_2$b), 4.51 (dd, J = 11.5, 1.8 Hz, 2H, $BnCH_2$a, $BnCH_2$b), 4.38 (d, J = 11.4 Hz, 1H, $BnCH_2$b), 4.27 (ddd, J = 6.2, 4.6, 1.3 Hz, 1H, H4), 3.88 (dd, J = 4.5, 0.5 Hz, 1H, H5), 3.81 (t, J = 4.5 Hz, 1H, H6), 1.50 (s, 9H, OtBu-CH_3).

^{13}C{^{1}H} NMR (151 MHz, Chloroform-*d*) δ 201.4 (CHO), 165.2 (COOtBu), 143.1 (C3), 137.4 (ArC1), 137.3 (ArC1), 137.1 (ArC1), 128.7 (2 × ArCH), 128.60 (2 × ArCH), 128.56 (2 × ArCH), 128.53 (2 × ArCH), 128.44 (2 × ArCH), 128.38 (ArC4), 128.3 (2 × ArCH), 128.2 (ArC4), 128.0 (ArC4), 125.8 (C2), 81.9 (C6), 81.1 (C5), 80.8 (OtBu-C), 77.8 (C4), 74.4 ($BnCH_2$), 73.5 ($BnCH_2$), 72.1 ($BnCH_2$), 28.3 (3 × OtBu-CH_3).

HRMS (ESI+) m/z: $[M + Na]^+$ Calcd for $C_{32}H_{36}O_6Na$ 539.2404; found 539.2410

B.1.11. Synthesis of (4S,5R,6R,E)-4,5,6-tris(benzyloxy)-7-hydroxyhept-2-enenitrile (6i)

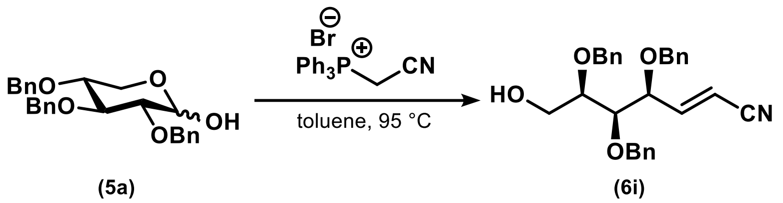

Wittig salt (1.00 g, 2.97 mmol, 2.50 equiv.) was dissolved in 20 mL of $CHCl_3$, and was twice extracted with 10 mL of 2N NaOH solution. The aqueous layers were once reextracted with fresh $CHCl_3$ (10 mL), before the combined organic layers were evaporated under reduced pressure. The residual oil was suspended in 30 mL of dry toluene, before tri-benzyl-xylose (5a) (500 mg, 1.19 mmol, 1.00 equiv.) was added to the suspension. The mixture was heated to 95 ° internal temperature, and was stirred at that temperature for 3 h, when TLC (LP:EtOAc 2:1, anis aldehyde stain) showed full consumption of starting material. The reaction was allowed to cool to rt, before volatiles were removed under reduced pressure.

The residual oil was submitted to column chromatography (MPLC, 45 g SiO2, 60 mL/min, LP:EtOAc, dryloading 4 g Celite, 4:1 – 2:1) to give 312 mg primary alcohol as colorless oil, as E/Z mixture(0.4:1).

^{1}H NMR (600 MHz, Chloroform-*d*) δ 7.49 – 7.27 (m, 30H, ArH), 6.64 (dd, *J* = 16.4, 4.9 Hz, 1H, E3), 6.54 (dd, *J* = 11.2, 8.7 Hz, 1H, Z3), 5.55 (dd, *J* = 16.4, 1.7 Hz, 1H, E2), 5.40 (dd, *J* = 11.2, 0.8 Hz, 1H, Z_2), 4.72 – 4.52 (m, 10H, 5 × E-BnCH$_2$, 5 × Z-BnCH$_2$), 4.46 – 4.42 (m, 2H, E-BnCH$_2$b, Z-BnCH$_2$b), 4.19 (td, *J* = 5.0, 1.7 Hz, 1H, E4), 3.77 – 3.67 (m, 4H, Z5, Z6, Z7a, E7a), 3.65 (t, *J* = 5.2 Hz, 1H, E5), 3.56 (ddd, *J* = 18.3, 8.6, 3.2 Hz, 3H, Z7b, E7b, E6).

$^{13}C\{^{1}H\}$ NMR (151 MHz, Chloroform-*d*) δ 151.9 (E3), 151.8 (Z3), 138.1 (Z-ArC1), 137.9 (E-ArC1), 137.7 – 137.5 (Z-ArC1), 137.6 (E-ArC1), 137.1 (Z-ArC1), 137.0 (E-ArC1), 128.9, 128.77, 128.75, 128.73, 128.68, 128.63, 128.62, 128.43, 128.39, 128.31, 128.28, 128.25, 128.22, 128.20, 128.1, 128.0 (15 × Z-ArCH, 15 × E-ArCH), 117.1 (E-CN), 115.5 (Z-CN), 102.0 (Z2), 101.1 (E2), 80.1 (Z6), 79.8 (E5), 79.0 (Z5), 78.8 (E6), 78.4 (E4), 77.0 (Z4), 74.9 (E-BnCH$_2$), 74.6 (Z-BnCH$_2$), 73.2 (Z-BnCH$_2$), 73.0 (E-BnCH$_2$), 72.6 (E-BnCH$_2$), 72.2 (Z-BnCH$_2$), 61.6 (Z7), 61.3 (E7).

HRMS (ESI+) m/z: (of E/Z mixture): $[M + Na]^+$ Calcd for $C_{28}H_{29}NO_4Na$ 466.1994; found 466.2002

B.1.12. Synthesis of (4S,5R,6S,E)-4,5,6-tris(benzyloxy)-7-oxo-hept-2-enenitrile (1i)

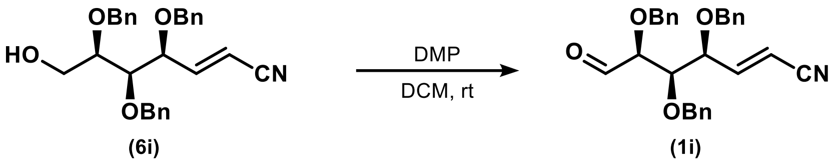

Starting material (6i) (260 mg, 0.557 mmol, 1.00 equiv.) as E/Z mixture (0.4:1), was dissolved in 5 mL of DCM, before DMP (317 mg, 0.724 mmol, 1.30 equiv.) was added in one portion, leading to formation of precipitate immediately after addition.

The reaction showed completeness after 15 min (TLC, LP:EtOAc 4:1), therefore 15 mL of saturated Na-HCO_3 solution was added, and the mixture was vigorously stirred for 30 min. The organic layer was once washed with water, and the combined aqueous layers was then reextracted with 25 mL of DCM, before the combined organic layers were dried over Na_2SO_4, filtered and evaporated.

Finally, column chromatography (30 g SiO_2, LP:EtOAc 8:1 – 4:1, 60 mL/min) afforded 220 mg of pure target product (1i) as an E/Z mixture (0.4:1) as colorless oil.

^{1}H NMR (600 MHz, Chloroform-*d*) δ 9.67 (s, 1H, E7), 9.63 (d, *J* = 1.0 Hz, 1H, Z7), 7.40 – 7.27 (m, 30H, 30 × ArH), 6.60 – 6.50 (m, 1H, E3, Z3), 5.50 (dd, *J* = 16.4, 1.7 Hz, 1H, E2), 5.38 (dd, *J* = 11.2, 0.8 Hz, 1H, Z2), 4.78 – 4.72 (m, 2H, E-BnCH$_2$a, Z-BnCH$_2$a), 4.69 – 4.63 (m, 2H, Z4, E-BnCH$_2$a), 4.59 (dd, *J* = 11.9, 3.0 Hz, 3H, Z-BnCH$_2$b, E-BnCH$_2$a, E-BnCH$_2$b), 4.56 – 4.41 (m, 6H, 6 × BnCH$_2$), 4.19 (td, *J* = 4.7, 1.6 Hz, 1H, E4), 3.97 (dd, *J* = 4.6, 0.9 Hz, 1H, Z6), 3.90 (d, *J* = 4.5 Hz, 1H, E6), 3.87 (t, *J* = 4.4 Hz, 1H, Z5), 3.78 (t, *J* = 4.3 Hz, 1H, E5).

^{13}C{^{1}H} NMR (151 MHz, Chloroform-*d*) δ 201.2 (Z7), 201.1 (E7), 151.6 (Z3), 151.4 (E3), 136.9 (2 × Z-ArC1), 136.8 (Z-ArC1), 136.8 (E-ArC1), 136.8 (E-ArC1), 136.6 (E-ArC1), 128.9, 128.7, 128.7, 128.7, 128.62, 128.60, 128.57, 128.54, 128.50, 128.45, 128.44, 128.43, 128.37, 128.35, 128.33, 128.26 (30 × ArCH), 116.9 (E-CN), 115.3 (Z-CN), 102.1 (Z2), 101.5 (E2), 81.4 (Z6), 81.2 (E6), 80.4 (Z5), 80.1 (E5), 77.3 (E4), 76.3 (Z4), 74.3 (E-BnCH$_2$), 74.1 (Z-BnCH$_2$), 73.5 (E-BnCH$_2$, Z-BnCH$_2$), 72.8 (E-BnCH$_2$), 72.6 (Z-BnCH$_2$).

HRMS (ESI+) m/z: (of E/Z mixture): $[M + Na]^+$ Calcd for $C_{28}H_{27}NO_4Na$ 464.1837; found 464.1840

B.1.13. Synthesis of Methyl (4S,5R,6R,E)-4,5,6,7-tetrahydroxyhept-2-enoate (4e)

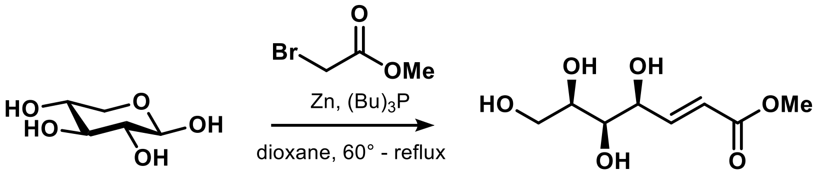

(4e)

Following a modified literature procedure[7] Zinc (654 mg, 10 mmol, 2.00 equiv.) was transferred into a 3-neck flask equipped with a condenser, septum and drying tube. The apparatus was thoroughly dried via heat gun heating and flushing with argon. before dry dioxane (20 mL) was added. The suspension was heated to 60 °C. Next, bromoacetate (1.53 g, 10.0 mmol, 2.00 equiv.) and tributyl phosphine (2.63 mL, 10.0 mmol, 2.00 equiv.) was added. The mixture was stirred at 60 °C for 5 min before D-xylose (730 mg, 5.00 mmol, 1.00 equiv.) was added. The reaction was stirred under reflux conditions for 60 min, when TLC ($CHCl_3$:MeOH:H_2O 14:7:1) indicated complete conversion into a single product. The reaction was allowed to cool to rt, was filtered and evaporated, to give a brownish syrup. Column chromatography (90 g SiO_2, 60 mL/min, DCM:MeOH 85:15 isocratic) to give 590 mg (57%) of target material (4e) as pure E compound as pale yellow oil.

^{1}H NMR (600 MHz, Deuterium Oxide) δ 7.00 (dd, *J* = 15.8, 5.2 Hz, 1H, H3), 6.14 (dd, *J* = 15.8, 1.6 Hz, 1H, H2), 4.45 (td, *J* = 5.3, 1.6 Hz, 1H, H4), 3.78 – 3.72 (m, 5H, H6, OCH_3), 3.69 – 3.57 (m, 4H, H5, H7ab).

^{13}C{^{1}H} NMR (151 MHz, Deuterium Oxide) δ 168.88 (COOMe), 147.66 (C3), 121.35 (C2), 72.77 (C5), 71.41 (C6), 71.29 (C4), 62.52 (C7), 52.17 (OCH_3).

B.1.14. Synthesis of Methyl (4S,5R,6S,E)-7-oxo-4,5,6-tris(trimethylsiloxy)hex-2-enoate (1e)

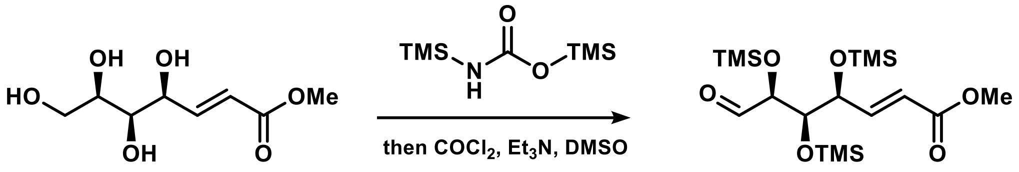

(4e) (1e)

Following a modified literature procedure[7] starting material (4e) (100 mg, 0.485 mmol, 1.00 equiv.) was dissolved in 2 mL of dry NMP and set under argon atmosphere. Next, N,O-bis-(trimethylsilyl)carbamate (559 mg; 2.67 mmol, 5.50 equiv.) was added in one portion and the mixture was stirred at rt until all solids had dissolved (~ 10 min). Then, 1M TBAF solution in THF (4 µL, 5 µmol, 0.01 equiv.) was added via hamilton syringe and the reaction was stirred at rt over night, when TLC (LP:EtOAc 1:1) indicated formation of a single new spot. The reaction was quenched with 1 mL of dry MeOH and was subsequently evaporated under reduced pressure. The resulting syrup was taken up in hexane (6 mL) and 3 times washed with water (10 mL each). The organic layer was dried over Na_2SO_4, filtered and evaporated. The resulting oil was analysed via ^{1}H-NMR and H-Si-HMBC spectra to ensure complete silylation.

^{1}H NMR (600 MHz, Chloroform-*d*) δ 7.16 (dd, *J* = 15.7, 4.6 Hz, 1H, H3), 5.97 (dd, *J* = 15.7, 1.8 Hz, 1H, H2), 4.34 (ddd, *J* = 6.2, 4.6, 1.8 Hz, 1H, H4), 3.73 (s, 3H, OCH_3), 3.70 (ddd, *J* = 6.7, 5.2, 3.7 Hz, 2H, H6), 3.64 – 3.60 (m, 2H, H5, H7a), 3.48 (dd, *J* = 10.2, 6.7 Hz, 1H, H7b), 0.13 (s, 9H, OTMS), 0.12 (s, 9H, OTMS), 0.09 (s, 9H, OTMS), 0.09 (s, 9H, OTMS).

Next a solution of oxalyl chloride (130 µL, 1.45 mmol, 3.00 equiv.) was dissolved in dry DCM. The mixture was cooled to -78 °C, before dry DMSO (207 µL, 2.91 mmol, 6.00 equiv.) was added dropwise under argon atmosphere. The solution was stirred at -78 °C for 15 min. Then the per-silylated compound from the previous step was dissolved in 5 mL DCM, and was slowly dropped into the cooled reaction mixture. (Dropping speed was adjusted to always stay below -75 °C) After 45 min, TLC (mini-workup by addition of Et_3N) showed complete consumption of starting material, therefor Et_3N (600 µL, 4.30 mmol, 9.0 equiv.) was added quickly to the reaction mixture, raising the temperature to -60 °C. The reaction was completed by stirring at -60 °C for 20 min and then allowing the mixture to warm to rt.
Finally, sat. NH_4Cl solution was added, phases were separated, and the organic layer was washed with water once. Drying over Na_2SO_4, filtration and evaporation under reduced pressure gave crude target material as mixture between target aldehyde and starting material.
Column chromatography (20 g SiO_2, LP:EtOAc 8:1, isocratic flow) gave 80 mg (40%) of target product (1e) in excellent purity as colorless oil.

^{1}H NMR (600 MHz, Chloroform-*d*) δ 9.67 (d, *J* = 0.6 Hz, 1H, H7), 7.09 (dd, *J* = 15.7, 4.8 Hz, 1H, H3), 5.98 (dd, *J* = 15.7, 1.8 Hz, 1H, H2), 4.41 (td, *J* = 4.6, 1.8 Hz, 1H, H4), 3.96 (d, *J* = 5.2 Hz, 1H, H6), 3.88 (dd, *J* = 5.3, 4.1 Hz, 1H, H5), 3.75 (s, 3H, OCH_3), 0.12 (s, 9H, OTMS), 0.12 (s, 9H, OTMS), 0.09 (s, 9H, OTMS).
$^{13}C\{^1H\}$ NMR (151 MHz, Chloroform-*d*) δ 201.2 (C7), 166.8 (COOMe), 148.2 (C3), 121.1 (C2), 78.1 (C6), 77.16 ($CDCl_3$, C5), 71.8 (C4), 51.7 (OCH_3), 0.3 (OTMS), 0.2 (OTMS), 0.1 (OTMS).
HRMS (ESI+) m/z: $[M + Na]^+$ Calcd for $C_{17}H_{36}O_6Si_3Na$ 444.1712; found 443.1721

B.2. Lyxose Derived Compounds

B.2.1. Methyl D-lyxopyranoside (4b)

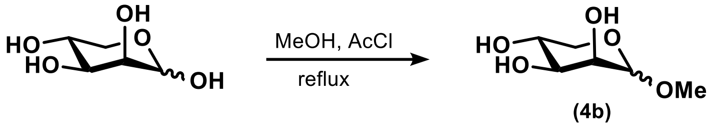

(4b)

D-Lyxose (2.00 g, 13.2 mmol, 1.00 equiv.) was dissolved in 40 mL of dry MeOH, and set under argon atmosphere. The solution was heated to reflux temperature, before acetyl chloride (1.9 ml, 26 mmol, 2.0 equiv.) was added to the solution in one portion. The reaction was refluxed over night, when TLC ($CHCl_3$:MeOH:H_2O 14:7:1) showed formation a one major product, and minute amounts of presumably other isomers. The mixture was cooled to rt, and solid $NaHCO_3$ was added to neutralise the reaction mixture. The mixture was then evaporated to dryness and the residue was directly submitted to column chromatography (dryload 10g Celite, DCM:MeOH 10% - 20%, 90 g SiO_2). This procedure yielded 1.94 g (89%) of target material (4b) as mixture of anomers (α:β 87:13).

^{1}H NMR (400 MHz, Methanol-d_4) δ 4.58 (d, J = 2.7 Hz, 1H, A1), 4.54 – 4.52 (m, 0.15H, B1), 3.98 (dd, J = 12.0, 3.3 Hz, 0.15H, B5a), 3.89 (t, J = 2.6 Hz, 0.15H, B2), 3.84 – 3.75 (m, 2H, A2, A4, B3), 3.68 (dd, J = 11.0, 5.3 Hz, 2.15H, A3, A5a), 3.65 – 3.61 (m, 0.15H, B4), 3.48 (s, 0.45H, B-OCH_3), 3.44 (d, J = 9.7 Hz, 1H, A5b), 3.41 (s, 3H, A-OCH_3), 3.30 (dd, J = 12.0, 5.9 Hz, 0.15H, B5b).

^{13}C{^{1}H} NMR (101 MHz, Methanol-d_4) δ 103.1 (A1), 102.8 (B1), 73.8 (B4), 72.7 (A2), 71.6 (A4), 69.8 (B3), 69.1 (B2), 68.5 (A3), 64.0 (A5), 63.3 (B5), 56.6 (B-OCH_3), 55.5 (A-OCH_3).

HRMS (ESI+) m/z: $[M + Na]^+$ Calcd for $C_6H_{12}O_5Na$: 187.0577; found 187.0580

$[\alpha_D^{20}]$ = +55 ° (c = 1.0, MeOH)

Spectral data in accordance with literature[10]

B.2.2. Synthesis of Methyl 2,3,4-tri-O-benzyl-α-D-lyxopyranoside (5b)

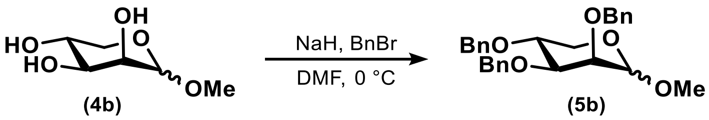

Starting material (4b) as anomeric mixture (1.94 g, 11.8 mmol, 1.00 equiv.) was dissolved in dry DMF (20 mL), and was cooled to 0 °C with an ice-bath. Next, sodium hydride (60% dispersion in paraffine) (3.15 g, 70.9 mmol, 6.00 equiv.) was added in 3 equal portions over 15 min while vigorously stirring the mixture. Upon complete addition, the reaction was stirred at 0 °C for further 10 min, before benzyl bromide (8.5 mL, 71 mmol, 6.0 equiv.) was dropped in over 10 min. Then, the ice-bath was removed, and the mixture was allowed to warm to rt. The reaction was monitored via TLC (LP:EtOAc 4:1, $CHCl_3$:MeOH:H_2O 14:7:1). After 6 hrs, TLC indicated complete consumption of starting material, and formation of two new spots. Therefore, the mixture was again cooled to 0 °C, and 10 mL of MeOH were slowly added to quench excess of reagent. Then the reaction mixture was distributed between EtOAc and water (100 mL each), the organic layer was washed with 20 mL of water 5 times, and once with brine. The organic layer was dried over Na_2SO_4, filtered and evaporated to give crude material.
Finally, column chromatography (45 g SiO_2, hexane, EtOAc 4:1, isocratic) gave 3.98 g (77%) of pure target material (5b) as colorless oil.

^{1}H NMR (400 MHz, Chloroform-*d*) δ 7.45 – 7.16 (m, 15H, A), 4.80 – 4.60 (m, 7H, 3 × $BnCH_2$, H1), 3.94 (td, *J* = 8.7, 5.0 Hz, 1H, H4), 3.84 – 3.74 (m, 3H, H2, H3, H5a), 3.53 (dd, *J* = 11.1, 9.1 Hz, 1H, H5b), 3.35 (s, 2H, OCH_3).
^{13}C{^{1}H} NMR (101 MHz, Chloroform-*d*) δ 138.8 (ArC1), 138.7 (ArC1), 138.53 (ArC1), 128.49 (2 × ArCH), 128.47 (2 × ArCH), 128.46 (2 × ArCH), 128.1 (2 × ArCH), 127.83 (2 × ArCH), 127.76 (ArC4), 127.75 (ArC4), 127.72 (2 × ArCH), 127.6 (ArC4), 100.1 (H1), 79.1 (C3), 75.6 (C2), 74.9 (C4), 73.3, 73.2, 72.8 (3 × $BnCH_2$), 61.6 (C5), 55.2 (OCH_3).
HRMS (ESI+) m/z: $[M + Na]^+$ Calcd for $C_{27}H_{30}O_5Na$ 457.1991; found: 457.1996
Spectral data in accordance with literature[11]

B.2.3. Synthesis of 2,3,4-Tri-O-benzyl-D-lyxopyranose (6b)

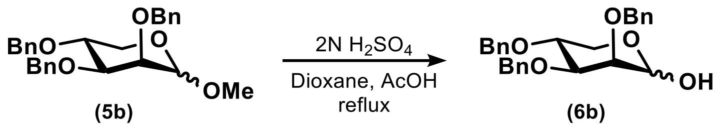

Starting material (5b) (2.50 g, 5.75 mmol, 1.00 equiv.) was taken up in 50 mL of a 1:1:1 mixture of dioxane, acetic acid and 2N H_2SO_4. The mixture was refluxed overnight, when TLC (LP:EtOAc 4:1) indicated full consumption of starting material, and formation of two new spots. The reaction was allowed to cool to rt, before it was distributed between diethyl ether and water (100 mL each). Then, the organic layer was neutralised with sat. $NaHCO_3$ solution (pH of aqueous phase >7). The organic layer was dried over Na_2SO_4, filtered and evaporated to give crude material. Finally, purification via column chromatography (45g SiO2, LP:EtOAc 6:1 – 4:1, 60 mL/min) gave 1.91 g (79%) of target material (6b) as an equilibrating mixture of anomeres as colorless oil.

1H NMR (400 MHz, Chloroform-d) δ 7.46 – 7.10 (m, 30H, ArH), 5.16 (br, 1H, B1), 5.12 (d, *J* = 4.3 Hz, 1H, A1), 4.78 – 4.53 (m, 11H, 11 × $BnCH_2$), 4.47 (d, *J* = 12.1 Hz, 1H, $BnCH_2$b), 4.06 (d, *J* = 11.2 Hz, 1H, B5a), 3.93 – 3.87 (m, 2H, B3, A3), 3.87 – 3.76 (m, 4H, B2, A4, A5a, A5b), 3.71 (dd, *J* = 4.3, 3.0 Hz, 1H, A2), 3.63 – 3.54 (m, 2H, B5b, B4).

$^{13}C\{^1H\}$ NMR (101 MHz, Chloroform-*d*) δ 138.7 (A-ArC1), 138.50 (A-ArC1), 138.48 (A-ArC1), 138.1 (B-ArC1), 137.9 (B-ArC1), 137.5 (B-ArC1), 128.7, 128.6, 128.53, 128.49, 128.3, 128.11, 128.08, 128.02, 128.00, 127.93, 127.85, 127.8, 127.7 (15 × A-ArCH, 15 × B-ArCH), 94.0 (A1), 93.1 (B1), 77.7 (A3), 76.8 (B3), 76.6 (A2), 75.0 (A4), 74.6 (B4), 74.3 (B-$BnCH_2$), 73.3 (A-$BnCH_2$), 73.09 (B2), 73.07 (B-$BnCH_2$), 72.7 (A-$BnCH_2$), 71.61 (A-$BnCH_2$), 71.57 (B-$BnCH_2$), 62.3 (A5), 57.7 (B5).

HRMS (ESI+) m/z: $[M + Na]^+$ Calcd for $C_{26}H_{28}O_5Na$: 443.1834; found 443.1845

B.2.4. Synthesis of Methyl (4R,5R,6R)-4,5,6-tris(benzyloxy)-7-hydroxyhept-2-enoate (7b)

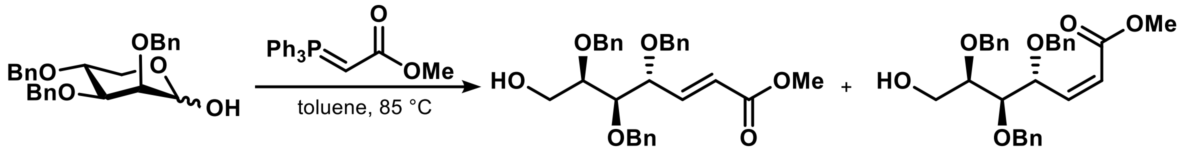

(6b) (7b-e) (7b-z)

Starting material (6b) (900 mg, 2.14 mmol, 1.00 equiv.), was taken up in toluene (25 mL). Next, Wittig reagent (2.19 g, 6.42 mmol, 3.00 equiv.) was added while the reaction was warming to 90 °C internal temperature. The reaction was stirred at this temperature for 22 hrs, before HPLC-MS (C8 column, 50% - 95% ACN). The reaction mixture was allowed to warm to rt, and then all volatiles were removed under reduced pressure. The residue was directly submitted to column chromatography (dry load 7 g celite, 45 g SiO_2, LP:EtOAc 15% - 33%, 60 mL/min). This procedure yielded 427 mg of pure target Z-isomer (7b-z), and 400 mg of a E/Z-mixture 95:5 (7b-e) (80% yield), both as colorless oil.

Analytics (7b-e)

^{1}H NMR (600 MHz, Chloroform-*d*) δ 7.33 – 7.21 (m, 15H, ArH), 7.02 (dd, *J* = 15.8, 6.5 Hz, 1H, H3), 6.09 (dd, *J* = 15.8, 1.2 Hz, 1H, H2), 4.62 (d, *J* = 11.3 Hz, 1H, $BnCH_2$a), 4.54 – 4.50 (m, 4H, 2 × $BnCH_2$), 4.24 (d, *J* = 11.5 Hz, 1H, $BnCH_2$b), 4.23 – 4.19 (m, 1H, H4), 3.72 – 3.68 (m, 4H, OCH_3, H5), 3.68 – 3.63 (m, 2H, H6, H7a), 3.60 – 3.55 (m, 1H, H7b).

^{13}C{^{1}H} NMR (151 MHz, Chloroform-*d*) δ 166.49 (COOMe), 145.9 (C3), 138.3 (ArC1), 137.8 (ArC1), 137.7 (ArC1), 128.64, 128.60, 128.59, 128.56, 128.11, 128.10, 127.99, 127.96, 127.88 (15 × ArCH), 123.6 (C2), 81.4 (C5), 79.2 (C6), 78.6 (C4), 74.6 ($BnCH_2$), 73.2 ($BnCH_2$), 71.5 ($BnCH_2$), 61.9 (C7), 51.8 (OCH_3).

HRMS (ESI+) m/z: $[M + Na]^+$ Calcd for $C_{29}H_{32}O_6Na$ 499.2096; found 499.2098

Analytics (7b-z)

^{1}H NMR (600 MHz, Chloroform-*d*) δ 7.31 – 7.20 (m, 15H, 15 × ArH), 6.37 (dd, *J* = 11.7, 9.1 Hz, 1H, H3), 5.93 (dd, *J* = 11.8, 1.1 Hz, 1H, H2), 5.41 (ddd, *J* = 9.1, 3.7, 1.1 Hz, 1H, H4), 4.75 (d, *J* = 11.5 Hz, 1H, $BnCH_2$a), 4.63 (d, *J* = 11.5 Hz, 1H, $BnCH_2$b), 4.58 (d, *J* = 11.0 Hz, 1H, $BnCH_2$a), 4.54 (d, *J* = 11.8 Hz, 1H, $BnCH_2$a), 4.47 (d, *J* = 11.6 Hz, 1H, $BnCH_2$b), 4.43 (d, *J* = 11.7 Hz, 1H, $BnCH_2$b), 3.85 (dd, *J* = 6.0, 3.7 Hz, 1H, H5), 3.73 (td, *J* = 7.6, 3.9 Hz, 1H, H7a), 3.70 – 3.67 (m, 1H, H7b), 3.67 (s, 3H, OCH_3), 3.59 (app. q, *J* = 5.1 Hz, 1H, H6), 2.28 (dd, *J* = 7.7, 5.3 Hz, 1H, OH).

^{13}C{^{1}H} NMR (151 MHz, Chloroform-*d*) δ 166.46 (COOMe), 147.03 (C3), 138.57 (ArC1), 138.49 (ArC1), 138.16 (ArC1), 128.51, 128.47, 128.4, 128.2, 128.0, 127.82, 127.80 (M), 127.76 (15 × ArCH), 122.0 (C2), 81.1 (C5), 80.2 (C6), 74.6 (C4), 73.8 ($BnCH_2$), 73.1 ($BnCH_2$), 71.7 ($BnCH_2$), 61.9 (C7), 51.6 (OCH_3).

HRMS (ESI+) m/z: $[M + Na]^+$ Calcd for $C_{29}H_{32}O_6Na$ 499.2096; found 499.2100

B.2.5. Synthesis of Methyl (4R,5R,6R, Z)-4,5,6-tris(benzyloxy)-7-oxo-hept-2-enoate (1b)

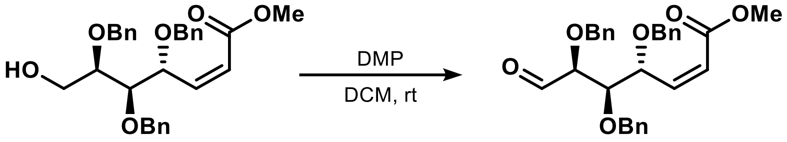

(7b-z) (1b)

Pure Z-configured starting material (7b-z) (401 mg, 0.841 mmol, 1.00 equiv.) was dissolved in 10 mL of DCM. While stirring at rt, DMP (442 mg, 1.01 mmol, 1.20 equiv.) was added in one portion, leading to an immediate formation of white precipitate. The reaction was stirred for 10 min, when TLC (LP:EtOAc 4:1) showed complete consumption of starting material and formation of a new spot. To quench the reaction, sat. $NaHCO_3$ solution (10 mL) was added to the reaction mixture. After vigorously stirring for 15 min, layers were separated, and the organic layer was filtered through a plug of cotton, to remove the white precipitate. Then the organic layer was washed with water twice, dried over Na_2SO_4, filtered and evaporated to give crude material. Finally, column chromatography (12 g, SiO_2, hexane:EtOAc 4:1 isocratic, 60 mL/min) gave 284 mg (71%) of pure target material (1b) as colorless oil.

^{1}H NMR (600 MHz, Chloroform-*d*) δ 9.78 – 9.53 (m, 1H, CHO), 7.52 – 7.05 (m, 15H, ArH), 6.27 (dd, *J* = 11.7, 8.7 Hz, 1H, H3), 5.92 (dd, *J* = 11.7, 1.2 Hz, 1H, H2), 5.45 (dd, *J* = 8.7, 3.6 Hz, 1H, H4), 4.64 (d, *J* = 11.7 Hz, 1H, $BnCH_2$a), 4.61 (d, *J* = 12.0 Hz, 1H, $BnCH_2$a), 4.57 (d, *J* = 11.7 Hz, 1H, $BnCH_2$b), 4.52 (d, *J* = 7.8 Hz, 1H, $BnCH_2$a), 4.50 (d, *J* = 7.9 Hz, 1H, $BnCH_2$b), 4.38 (d, *J* = 11.8 Hz, 1H, $BnCH_2$b), 3.96 (d, *J* = 3.8 Hz, 2H, H5, H6), 3.69 (s, 3H, OCH_3).

^{13}C{^{1}H} NMR (151 MHz, Chloroform-*d*) δ 201.4 (CHO), 166.4 (COOMe), 147.7 (C3), 138.0 (ArC1), 137.9 (ArC1), 137.4 (ArC1), 122.0 (C2), 83.3 (C5/C6), 81.3 (C5/C6), 74.4 (C4), 73.3 ($BnCH_2$), 73.2 ($BnCH_2$), 71.8 ($BnCH_2$), 51.6 (OCH_3).

HRMS (ESI+) m/z: $[M + Na]^+$ Calcd for $C_{29}H_{30}O_6Na$ 497.1935; found 497.1933

B.3. Ribose Derived Compounds

B.3.1. Synthesis of Methyl 2,3,4-tri-*O*-benzyl-β-D-ribopyranoside (4c)

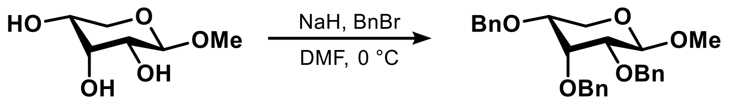

(4c)

Ribo pyranoside (2.16 g, 13.0 mmol, 1.00 equiv.) was dissolved in 60 mL dry DMF. The solution was cooled to 0 °C with an ice-bath. Then NaH (3.13 g, 78.2 mmol, 6.00 equiv., 60% dispersion in paraffin) was added in 3 equal portions over 10 min. The reaction was stirred for 30 min at 0 °C, before benzyl bromide (9.50 mL, 78.2 mmol, 6.00 equiv.) was added dropwise over 15 min. After completion of the addition, the ice bath was removed, and the reaction was stirred at rt overnight. The next morning, TLC (LP:EtOAc 8:1 and $CHCl_3$: MeOH, water 14:6:1, anis aldehyde stain) showed complete consumption of starting material, and formation of a single new spot. Therefore, excess of reagent was quenched by addition of 10 mL of MeOH under ice-bath cooling.

The reaction mixture was then distributed between water (120 mL) and EtOAc (150 mL). The aqueous layer was 4 times extracted with EtOAc (220 mL total), until TLC showed complete extrtraction. The combined organic layers were dried over Na_2SO_4, filtered and evaporated to give cure material. Colum chromatography (MPLC, 180 g, 60 mL/min, LP:EtOAc 5% - 20%) afforded 4.7 g (83%) of target material (4c) in excellent purity as colorless oil.

^{1}H NMR (600 MHz, Chloroform-*d*) δ 7.38 – 7.35 (m, 2H, 2x ArH), 7.32 – 7.20 (m, 13H, 13x ArH), 4.81 – 4.74 (m, 3H, $BnCH_2$a-O2, $BnCH_2$ab-O3), 4.69 (d, J = 7.0 Hz, 1H, H1), 4.60 (d, J = 12.2 Hz, 1H, $BnCH_2$b-O2), 4.53 (d, J = 12.0 Hz, 1H, $BnCH_2$a-O4), 4.49 (d, J = 12.1 Hz, 1H, $BnCH_2$b-O4), 4.01 (td, J = 2.7, 1.1 Hz, 1H, H3), 3.81 (dd, J = 11.0, 9.4 Hz, 1H, H5a), 3.74 (ddd, J = 11.0, 4.7, 1.1 Hz, 1H, H5b), 3.49 (ddd, J = 9.4, 4.7, 2.5 Hz, 1H, H4), 3.46 (s, 3H, OCH_3), 3.21 (dd, J = 7.1, 2.8 Hz, 1H, H2).

^{13}C{^{1}H} NMR (151 MHz, Chloroform-*d*) δ 139.13 (PhC1-O3), 138.87 (PhC1-O2), 138.30 (PhC1-O4), 128.53 (2 × ArCH), 128.42 (2 × ArCH), 128.30 (2 × ArCH), 127.95 (2 × ArCH), 127.82 (ArC4), 127.75 (2xArCH), 127.65 (2 × ArCH), 127.58 (ArC4), 127.49 (ArC4), 102.10 (C1), 78.29 (C2), 75.55 (C3), 75.31 (C4), 73.94 ($BnCH_2$-O3), 72.97 ($BnCH_2$-O2), 71.54 ($BnCH_2$-O4), 62.23 (C5), 56.81 (OCH_3).

Optical Rotation: $[\alpha]_D^{20}$ -25.3° (c 1.0, $CHCl_3$)

HRMS (ESI+) m/z: $[M + Na]^+$ Calcd for $C_{27}H_{30}O_5Na$ 457.1991; found: 457.1997

Spectral data in accordance with literature[8]

B.3.2. Synthesis of 2,3,4-tri-*O*-benzyl-D-ribopyranose (5c)

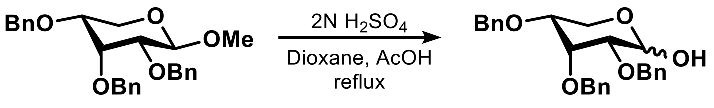

Starting material (4c) (4.60 g, 10.6 mmol, 1.00 equiv.) was suspended in 100 mL of a 1:1:1 mixture of dioxane, acetic acid and 2N sulfuric acid. The reaction was refluxed over night. After 14 hrs, TLC (LP:EtOAc 4:1) showed complete consumption of starting material and formation of a smearing spot. The reaction was allowed to cool to rt, diluted with 200 mL EtOAc and then neutralised with sat. $NaHCO_3$ solution (pH 8 reached). The organic layer was washed with brine, dried over Na_2SO_4, filtered and evaporated to give crude material. Purification via column chromatography (180 g SiO2, 60 mL/min, dry load 10g celite, LP:EtOAc 4:1 isocratic) gave target material (5c) as colorless oil in excellent purity as anomeric mixture (α:β 3:2).

^{1}H NMR (400 MHz, Chloroform-*d*) δ 7.44 – 7.27 (m, 30H, ArH), 5.47 (d, *J* = 10.4 Hz, 1H, OH-A1), 5.19 – 5.11 (m, 2H, A1, B1), 4.90 (d, *J* = 11.5 Hz, 1H, $BnCH_2$a-A3), 4.86 – 4.80 (m, 3H, $BnCH_2$b-A3, $BnCH_2$ab-B), 4.74 (d, *J* = 12.0 Hz, 1H, $BnCH_2$a-B2), 4.70 – 4.64 (m, 2H, $BnCH_2$a-A2, $BnCH_2$b-B2), 4.59 – 4.51 (m, 5H, $BnCH_2$b-A2, $BnCH_2$ab-A4, $BnCH_2$ab-B), 4.31 – 4.25 (m, 1H, A3), 4.14 (td, *J* = 2.6, 1.1 Hz, 1H, B3), 4.02 (t, *J* = 11.0 Hz, 1H, A5a), 3.91 (dd, *J* = 10.9, 10.0 Hz, 1H, B5a), 3.82 (ddd, *J* = 10.9, 5.0, 1.2 Hz, 1H, B5b), 3.67 (ddd, *J* = 11.2, 5.2, 1.1 Hz, 1H, A5b), 3.55 (ddd, *J* = 10.0, 5.0, 2.4 Hz, 1H, B4), 3.50 (ddd, *J* = 10.8, 5.2, 2.4 Hz, 1H, A4), 3.36 (dd, *J* = 3.6, 2.5 Hz, 1H, A2), 3.19 (dd, *J* = 7.3, 2.7 Hz, 1H, B2).
^{13}C{^{1}H} NMR (101 MHz, Chloroform-*d*) δ 139.0 (PhC1-B), 138.3 (PhC1-B), 138.1 (PhC1-B), 137.9 (PhC1-A), 137.8 (PhC1-A), 137.7 (PhC1-A), 128.5 (ArCH), 128.5 (ArCH), 128.5 (ArCH), 128.4 (ArCH), 128.4 (ArCH), 128.2 (ArCH), 128.2 (ArCH), 127.9 (ArCH), 127.9 (ArCH), 127.8 (ArCH), 127.7 (ArCH), 127.7 (ArCH), 127.5 (ArCH), 127.5 (ArCH), 127.4 (ArCH), 94.8 (B1), 92.0 (A1), 79.5 (B2), 76.7 (A3), 75.3 (B4), 75.3 ($BnCH_2$-A3), 74.7 (B3), 74.5 (A4), 74.4 (A2), 74.0 ($BnCH_2$-B3/B4), 72.4 ($BnCH_2$-B2), 71.4 ($BnCH_2$-B3/B4), 71.3 ($BnCH_2$-A4), 70.6 ($BnCH_2$-A2), 62.4 (B5), 56.2 (A5).
Optical Rotation: $[\alpha]_D^{20}$ +2.6° (c 1.0, $CHCl_3$)
HRMS (ESI+) m/z: $[M + Na]^+$ Calcd for $C_{26}H_{28}O_5Na$ 443.1832; found: 443.1833

B.3.3. Synthesis of Methyl (4S,5S,6R)-4,5,6-tris(benzyloxy)-7-hydroxyhept-2-enoate (6c)

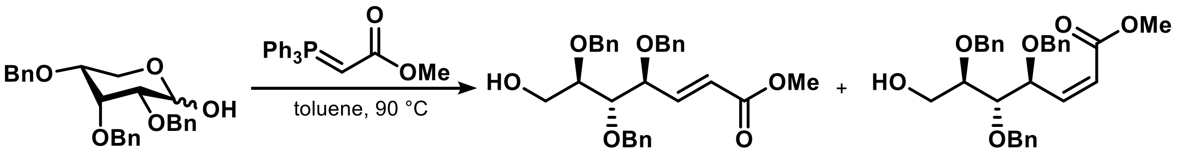

(5c) (6c-e) (6c-z)

Starting material (5c) (960 mg, 2.28 mmol, 1.00 equiv.) was transferred into a three-neck flask, equipped with a thermometer and a reflux condenser. Then, toluene (35 mL) and Wittig reagent (2.33 g, 6.86 mmol, 3.00 equiv.) were added, and the mixture was heated to 90 °C internal temperature. The reaction was stirred at that temperature o/n. After 17 h of reaction time TLC (LP:EtOAc 4:1, anis aldehyde stain) indicated completion of the reaction. The mixture was allowed to cool to rt, before volatiles were removed under reduced pressure. The residual material was directly submitted to column chromatography (MPLC, dry-loading 7 g celite, 90 g SiO2, LP:EtOAc 5:1 – 3:1, 60 mL/min) afforded 566 mg of pure E material (6c-e) as colorless oil and 250 mg of pure Z material (6c-z) as pale yellow oil. Overall yield: 78%

Analytical data (6c-e):

^{1}H NMR (400 MHz, Chloroform-*d*) δ 7.32 – 7.13 (m, 15H, 15x Ar-H), 6.86 (dd, *J* = 15.8, 6.6 Hz, 1H, H3), 5.86 (dd, *J* = 15.8, 1.3 Hz, 1H, H2), 4.69 (d, *J* = 11.3 Hz, 1H, C5-Bn-CH_2a), 4.59 (d, *J* = 11.3 Hz, 1H, C5-Bn-CH_2b), 4.57 – 4.48 (m, 2H, C4-BnCH_2a, C6-BnCH_2a), 4.41 (d, *J* = 11.6 Hz, 1H, C6-BnCH_2b), 4.35 (d, *J* = 11.9 Hz, 1H, C4-BnCH_2b), 4.18 (ddd, *J* = 6.6, 3.7, 1.3 Hz, 1H, H4), 3.79 (dd, *J* = 6.7, 3.7 Hz, 1H, H5), 3.68 (dd, *J* = 4.1, 1.6 Hz, 2H, H7a, H7b), 3.66 (s, 3H, OCH_3), 3.47 (dt, *J* = 6.7, 4.1 Hz, 1H, H6).

^{13}C{^{1}H} NMR (151 MHz, Chloroform-*d*) δ 166.4 (C=O), 145.1 (d, *J* = 2.8 Hz(C3)), 137.9 (C1-Bn(C5)), 137.8 (2x C1-Bn(C4, C6)), 128.6 (2x Ar-CH), 128.5 (2x Ar-CH), 128.5 (2x Ar-CH), 128.4 (2x Ar-CH), 128.12 (2x Ar-CH), 128.1 (Ar-C4), 128.0 (Ar-C4), 128.0 (Ar-C4), 127.9 (2x Ar-CH), 123.6 (C4), 80.8 (C5), 78.8 (C4), 78.3 (d, *J* = 1.9 Hz(C6)), 74.3 (BnCH_2(C5)), 71.9 (BnCH_2(C6)), 71.6 (BnCH_2(C4)), 61.0 (C7), 51.77 (OCH_3).

Optical Rotation: $[\alpha]_D^{20}$+25.4° (c 1.0, $CHCl_3$)

HRMS (ESI+) m/z: $[M + Na]^+$ Calcd for $C_{29}H_{32}O_5Na^+$ 499.2097; found: 499.2093

Analytical data (6c-z):

^{1}H NMR (600 MHz, Chloroform-*d*) δ 7.32 – 7.19 (m, 15H, Ar), 6.24 (dd, *J* = 11.8, 8.4 Hz, 1H, H3), 5.85 (dd, *J* = 11.8, 1.4 Hz, 1H, H2), 5.43 (ddd, *J* = 8.5, 3.0, 1.4 Hz, 1H, H4), 4.77 (d, *J* = 11.5 Hz, 1H, BnCH_2a), 4.64 (d, *J* = 11.5 Hz, 1H, BnCH_2b), 4.56 (d, *J* = 11.9 Hz, 1H, BnCH_2a), 4.51 – 4.46 (m, 3H, BnCH_2b, BnCH_2), 3.81 (dd, *J* = 6.6, 3.0 Hz, 1H, H5), 3.75 (dd, *J* = 11.8, 4.7 Hz, 1H, H7a), 3.70 (dd, *J* = 11.8, 3.7 Hz, 1H, H7b), 3.64 (ddd, *J* = 6.6, 4.7, 3.6 Hz, 1H, H6), 3.60 (s, 3H, OCH_3).

^{13}C{^{1}H} NMR (151 MHz, Chloroform-*d*) δ 166.35 (COOMe), 148.29 (C3), 138.38 (2x Ar-C1), 138.21 (Ar-C1), 128.52 (2 × ArCH), 128.46 (2 × ArCH), 128.44 (2 × ArCH), 128.26 (2 × ArCH), 127.92 (2 × ArCH), 127.82 (Ar-C4), 127.80 (Ar-C4), 127.78 (2 × ArCH), 127.70 (ArC4), 121.33 (C2), 81.66 (C5), 78.59 (C6), 75.40 (C4), 73.23 (C5-BnCH_2), 71.99 (C4/C6-BnCH_2), 71.97 (C4/C6-BnCH_2), 61.34 (C7), 51.51 (OCH_3).

Optical Rotation: $[\alpha]_D^{20}$+38.0° (c 1.0, $CHCl_3$)

HRMS (ESI+) m/z: $[M + Na]^+$ Calcd for $C_{29}H_{32}O_6Na^+$ 499.2097; found: 499.2094

Spectral of both isomers in accordance with literature[9]

B.3.4. Synthesis of Methyl (4S,5S,6R, E)-4,5,6-tris(benzyloxy)-7-oxo-hept-2-enoate (1c)

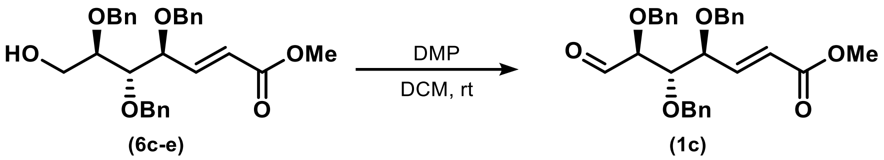

Starting material (6c-e) (356 mg, 0.747 mmol, 1.00 equiv.) was dissolved in 10 mL DCM, and DMP (425 mg, 0.971 mmol, 1.30 equiv.) were added in one portion. The reaction was stirred at rt for 10 min, before TLC (LP : EtOAc 4:1) indicated complete consumption of starting material and formation of a new more apolar spot. The reaction was quenched by addition of 10 mL of sat. $NaHCO_3$ solution and vigorous stirring for 15 min. Next, layers were separated, and the organic layer was filtered through a pad of cotton to remove the white precipitate that formed during reaction. The organic layer was then washed with water (2 × 20 mL), and the combined organic layers were once reextracted with DCM (25 mL). After drying over Na_2SO_4, filtration end evaporation of the organic layers, crude product was obtained. Finally, purification via column chromatography (24 g SiO_2, 30 mL/min, LP : EtOAc 6:1 – 4:1) gave 280 mg (79%) of target material (1c) as colorless oil, in excellent purity.

1H NMR (400 MHz, Chloroform-d) δ 9.50 (d, J = 1.1 Hz, 1H, CHO), 7.38 – 7.23 (m, 15H, 15 × Ar-H), 6.92 (dd, J = 15.8, 6.2 Hz, 1H, H3), 6.05 (dd, J = 15.8, 1.3 Hz, 1H, H2), 4.73 (d, J = 12.0 Hz, 1H, C6-$BnCH_2$a), 4.67 (d, J = 12.0 Hz, 1H, C6-$BnCH_2$b), 4.59 – 4.53 (m, 2H, C5-$BnCH_2$a, C4-$BnCH_2$a), 4.50 (d, J = 11.6 Hz, 1H, C5-$BnCH_2$b), 4.44 (d, J = 11.4 Hz, 1H, C4-$BnCH_2$b), 4.32 (ddd, J = 7.8, 6.2, 1.3 Hz, 1H, H4), 4.03 (dd, J = 2.7, 1.1 Hz, 1H, H6), 3.81 (dd, J = 8.0, 2.7 Hz, 1H, H5), 3.75 (s, 3H, OCH_3).

$^{13}C\{^1H\}$ NMR (101 MHz, Chloroform-d) δ 201.3 (CHO), 166.4 (COOMe), 145.6 (C3), 137.3 (ArC1), 137.2 (2 × ArC1), 128.7 (2 × ArCH), 128.6 (2 × ArCH), 128.5 (2 × ArCH), 128.3 (2 × ArCH), 128.21 (ArC4), 128.15 (2 × ArCH), 128.12 (ArC4), 128.0 (2 × ArCH, ArC4), 123.6 (C2), 83.0 (C5), 82.2 (C6), 76.7 (C4), 73.4 ($BnCH_2$), 73.1 ($BnCH_2$), 72.0 ($BnCH_2$).

HRMS (ESI+) m/z: $[M + Na]^+$ Calcd for $C_{29}H_{30}O_6Na^+$ 497.1935; found 497.1941

Spectral of both isomers in accordance with literature[9]

B.4. Arabinose Derived Compounds

B.4.1. Synthesis of Methyl 2,3,4-tri-*O*-benzyl-β-D-arabinopyranoside (4d)

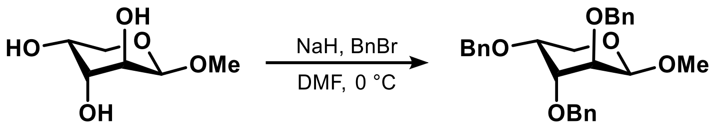

Methyl-β-D-arabinopyranoside (2.0 g, 12.2 mmol, 1.00 equiv.) was transferred into a 250 mL three-neck flask equipped with a dropping funnel. The flask was set under argon atmosphere and 40 mL dry DMF were added. The solution was cooled to 0 °C with an ice-bath. Upon reaching the desired temperature, NaH as 60% dispersion in paraffine oil (2.93 g, 73 mmol, 6.00 equiv.) was added under vigorous stirring, in three equal portions over 20 minutes. The solution was stirred for 30 min at 0 °C, leading to solidification of the mixture due to salt formation. Next, benzyl bromide (8.9 mL, 73 mmol, 6.0 equiv.) was added dropwise over 30 minutes. Upon complete addition, the ice bath was removed, and the mixture was allowed to warm to rt. The reaction was monitored via TLC (LP:EtOAc 4:1 and $CHCl_3$:MeOH:H_2O 14:7:1; anis aldehyde stain). After 3 h, TLC indicated complete consumption of starting material, and formation of a single product, therefore the reaction was again cooled to 0 °C with an ice bath, before 40 mL of MeOH were added slowly to quench the excess reagent. The mixture was stirred for further 15 min, before 40 mL EtOAc were added, and phases were separated and the organic layer was washed 4 times with 10 mL H_2O each. The combined aqueous layers were once reextracted with 40 mL EtOAc, and the combined organic layers were dried over Na_2SO_4, filtered and evaporated (twice co-evaporated from toluene) to give the crude product as a colourless oil.

Cooling the crude material to approximately -10 °C, and addition of ice-cold hexane (~ 30 mL), initiated crystallisation of target material as white needles. The formed crystals were filtered off, and washed with small amounts of cold hexane. The mother liquor was evaporated, and the crystallisation process was repeated two times to give 4.8 g (90%) of target material (4d) in excellent purity (in three equally pure crystal fractions).

^{1}H NMR (600 MHz, Chloroform-*d*) δ 7.66 – 7.02 (m, 15H, ArH), 4.86 (d, *J* = 12.1 Hz, 1H, $BnCH_2$a), 4.76 (d, *J* = 11.9 Hz, 1H, $BnCH_2$a), 4.74 – 4.67 (m, 4H, $BnCH_2$, $BnCH_2$b, H1), 4.65 (d, *J* = 11.9 Hz, 1H, $BnCH_2$b), 4.01 (dd, *J* = 9.7, 3.5 Hz, 1H, H2), 3.88 (dd, *J* = 9.7, 3.2 Hz, 1H, H3), 3.75 (s, 1H, H4), 3.66 (dd, *J* = 12.4, 2.4 Hz, 1H, H5a), 3.60 (dd, *J* = 12.4, 1.2 Hz, 1H, H5b), 3.38 (s, 3H, OCH_3).

^{13}C{^{1}H} NMR (151 MHz, Chloroform-*d*) δ 138.90 (ArC1), 138.81 (ArC1), 138.50 (ArC1), 128.48 (2 × ArCH), 128.46 (4 × ArCH), 128.15 (2 × ArCH), 128.03 (2 × ArCH), 127.78 (ArC4), 127.76 (2 × ArCH), 127.75 (ArC4), 127.64 (ArC4), 99.50 (C1), 77.42 (C3), 76.53 (C2), 74.21 (C4), 73.86 ($BnCH_2$), 72.95 ($BnCH_2$), 71.92 ($BnCH_2$), 60.35 (C5), 55.63 (OCH_3).

Optical Rotation: $[\alpha]_D^{20}$-44.3° (c 1.0, $CHCl_3$)

HRMS (ESI+) m/z: $[M + Na]^+$ Calcd for $C_{27}H_{30}O_5Na^+$ 457.1991; found: 457.1994

Spectral data in accordance with literature[8]

B.4.2. Synthesis of Methyl (4S, 5S, 6S)-4,5,6-tris(benzyloxy)-7-hydroxyhept-2-enoate (6d)

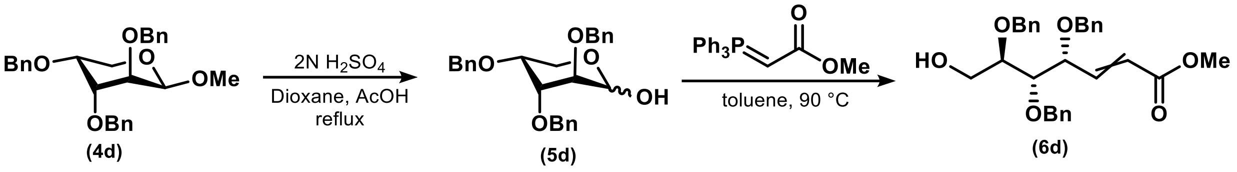

Starting material (4d) (4.62 g, 10.6 mmol, 1.00 equiv.) was taken up in 50 mL of a 1:1:1 mixture of dioxane, acetic acid and 2N H_2SO_4 and was refluxed for 6 h until TLC (LP:EtOAc 4:1) showed full consumption of starting material and formation of a more polar spot. The reaction was allowed to cool to rt, before it was diluted with Et_2O (100 mL). Layers were separated, and the organic layer was neutralised with sat. $NaHCO_3$ solution. The organic layers were dried over Na_2SO_4, filtered and evaporated to give 3.81 g of a white amorphous solid that was recrystallised from 115 mL of hexane. NMR indicated formation of reducing sugar (5d) together with an unknown side-product (~ 90% purity). The material was suspended in toluene (40 mL), Wittig reagent (9.25 g, 27.1 mmol, 3.00 equiv.) was added, and the reaction was stirred at 90 °C internal temperature overnight.
After 16 h, TLC (LP:EtOAc 4:1) and LC-MS (C8 column, 50-95% ACN) indicated complete consumption of starting material and formation of two new products. The reaction mixture was allowed to cool to rt, before all volatiles were removed under reduced pressure. The residue was purified via column chromatography (180 g SiO_2, 60 mL/min, hexane : EtOAc 5:1 – 3:1) to give 3.41 g of the target material (6d) as an E/Z 2:1 mixture as colorless oil.(67% over two steps).

^{1}H NMR (600 MHz, Chloroform-*d*) δ 7.37 – 7.26 (m, 30H, ArH), 6.99 (dd, *J* = 15.8, 6.4 Hz, 1H, E3), 6.33 (dd, *J* = 11.8, 8.7 Hz, 1H, Z3), 6.12 (dd, *J* = 15.8, 1.3 Hz, 1H, E2), 5.85 (dd, *J* = 11.8, 1.2 Hz, 1H, Z2), 5.33 (ddd, *J* = 8.7, 3.3, 1.2 Hz, 1H, Z4), 4.71 (d, *J* = 11.4 Hz, 1H, Z-CH_2a), 4.64 (dd, *J* = 12.2, 5.1 Hz, 4H, 3 × E-BnCH_2a, Z-CH_2b), 4.56 (d, *J* = 11.7 Hz, 1H, Z-BnCH_2a), 4.51 (d, *J* = 11.5 Hz, 1H, Z-BnCH_2a), 4.47 (d, *J* = 11.3 Hz, 1H, E-BnCH_2a), 4.41 (d, *J* = 11.5 Hz, 1H, Z-BnCH_2b), 4.36 (d, *J* = 11.8 Hz, 1H, Z-BnCH_2b), 4.33 (d, *J* = 11.8 Hz, 1H, E-BnCH_2b), 4.29 (d, *J* = 11.3 Hz, 1H, E-BnCH_2b), 4.27 (ddd, *J* = 6.3, 3.6, 1.3 Hz, 1H, E4), 3.89 (dd, *J* = 12.0, 3.9 Hz, 1H, Z7a), 3.87 – 3.83 (m, 1H, Z7b, Z5), 3.83 – 3.80 (m, 1H, E7a), 3.80 – 3.78 (m, 1H, E7b), 3.78 – 3.72 (m, 5H, E5, E-OCH_3, Z6), 3.69 (d, *J* = 6.3 Hz, 4H, E6, Z-OCH_3).
^{13}C{^{1}H} NMR (151 MHz, Chloroform-*d*) δ 166.4 (E-COOMe), 166.3 (Z-COOMe), 148.7 (Z-C2), 146.0 (E-C2), 138.3 (Z-ArC1), 138.1 (Z-ArC1), 138.01 (Z-ArC1), 137.99 (E-ArC1), 137.71 (E-ArC1), 137.69 (E-ArC1), 128.61, 128.60, 128.54, 128.53, 128.44, 128.43, 128.37, 128.3, 128.1, 128.0, 127.94 (ArC), 127.88, 127.86, 127.83 (15 × Z-ArCH, 15 × E-ArCH), 123.1 (E-C3), 121.8 (Z-C3), 81.0 (Z-C6), 80.4 (E-C6), 79.3 (Z-C5), 78.8 (E-C5), 78.2 (E-C4), 75.07 (Z-C4), 75.05 (E-BnCH_2, Z-BnCH_2), 71.92 (E-BnCH_2), 71.89 (Z-BnCH_2), 71.7 (E-BnCH_2, Z-BnCH_2), 60.9 (Z-C7), 60.6 (E-C7), 51.8 (E-OCH_3), 51.6 (Z-OCH_3).
HRMS (ESI+) m/z: $[M + Na]^+$ Calcd for $C_{29}H_{32}O_6Na^+$ 499.2097; found 499.2101
Optical Rotation: $[\alpha]_D^{20}$ -32° (c 1.0, $CHCl_3$)

B.4.3. Synthesis of Methyl (4S, 5S, 6S)-4,5,6-tris(benzyloxy)-7-oxo-hept-2-enoate (1d)

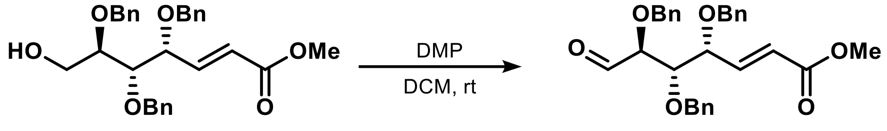

Starting material (6d) (1.41 g, 2.96 mmol, 1.00 equiv.) as a 2:1 E/Z Mixture was dissolved in 10 mL of DCM, before DMP (1.68 g, 3.85 mmol, 1.30 equiv.) was added in one portion. The reaction was stirred at rt and was monitored *via* TLC (LP:EtOAc 3:1). After 15 min, all starting material was consumed. Therefore, the reaction was treated with 15 mL of sat. $NaHCO_3$ solution, and was stirred vigorously for 20 min, before the mixture was diluted with 50 mL of DCM. Next, layers were separated, and the organic layer was filtered through a plug of cotton to remove any white precipitate. The organic layer was once washed with brine, before it was dried over Na_2SO_4, filtered and evaporated to give crude product as colorless oil.
Purification was done via column chromatography (90 g SiO2, 60 mL/min, hexane : EtOAc 4:1 isocratic), to give 740 mg of pure E-isomer (1d) and 470 mg a 3:2 mixture of Z/E Isomeres, both as colorless oils. (Overall yield 86%)
Analytical data:
^{1}H NMR (600 MHz, Chloroform-*d*) δ 9.63 (d, *J* = 1.5 Hz, 1H, CHO), 7.44 – 7.14 (m, 15H, ArH), 7.03 (dd, *J* = 15.9, 6.2 Hz, 1H, H3), 6.11 (dd, *J* = 15.9, 1.4 Hz, 1H, H2), 4.63 – 4.58 (m, 3H, $BnCH_2$, $BnCH_2$a), 4.56 (d, *J* = 11.7 Hz, 1H, $BnCH_2$a), 4.47 (d, *J* = 11.6 Hz, 1H, $BnCH_2$b), 4.36 (d, *J* = 11.8 Hz, 1H, $BnCH_2$b), 4.27 – 4.20 (m, 1H, H4), 4.05 (dd, *J* = 3.9, 1.5 Hz, 1H, H6), 3.91 (dd, *J* = 5.2, 3.9 Hz, 1H, H5), 3.74 (s, 3H, OCH_3).
^{13}C{^{1}H} NMR (151 MHz, Chloroform-*d*) δ 201.5 (CHO), 166.4 (COOMe), 145.1 (C3), 137.4 (2 × ArC1), 137.2 (ArC1), 128.60 (2 × ArCH), 128.58 (2 × ArCH), 128.55 (2 × ArCH), 128.3 (2 × ArCH), 128.2 (ArC4), 128.12 (ArC4), 128.09 (4 × ArCH), 128.07 (ArC4), 123.4 (C2), 83.3 (C5), 81.8 (C6), 78.0 (C4), 74.0 ($BnCH_2$), 73.1 ($BnCH_2$), 71.9 ($BnCH_2$), 51.8 (OCH_3).
HRMS (ESI+) m/z (of E/Z mixture): $[M + Na]^+$ Calcd for $C_{29}H_{30}O_6Na^+$ 497.1940; found 497.1942

C. Cyclisations and Eliminations

C.1. General Procedures

C.1.1. General Procedure for Carbocylisation

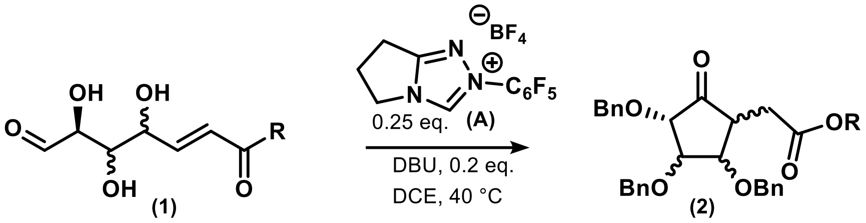

Starting aldehyde (1.00 equiv.) and pre-catalyst salt (**A**) (0.25 equiv.) were transferred into the reaction vessel. The vessel was charged with a stirring bar and 1,2-dichloroethane was added to form a 10% solution. The mixture was put into the sonicator for 10 s to disperse the insoluble catalyst salt. The reaction mixture was heated to 40 °C, before DBU (0.2 equiv.) was pipetted into the mixture, leading to orange colorisation within seconds. The reaction was monitored via TLC and LC-MS (C8 column, H_2O:ACN 50% - 95% gradient). After complete consumption of the starting material, the reaction mixture was immediately put on ice and was once extracted with ice-cold 1M HCl. The organic layer was then also washed with brine, before it was dried over Na_2SO_4, filtered and evaporated to give crude material.
For final purification the crude mixture was flashed through a plug of silica (hexane : EtOAc 1:1) to give target cyclised cyclopentane-one as mixture of diastereomeres

Additional information:

We observed a tendency to eliminate OBn with most cyclopentanones on silica and generally longer exposure to acids – it is therefore recommended to keep contact times during extraction and flash filtration to a minimum to obtain clean products. Further we observed that *xylo*-configured carbocycles show a high tendency to racemise at C1 to a ~1:1 mixture. However, the same precautions that stop elimination also prevent racemisation and allow isolation in an enantiomerically enriched form.
The reaction completes usually within minutes.

C.1.2. General Procedure Elimination

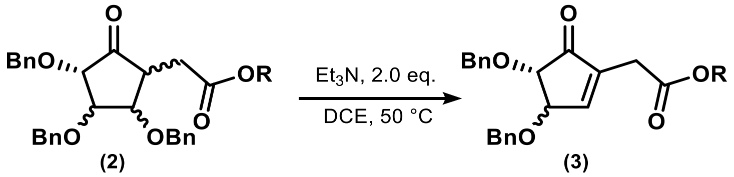

A diastereomeric mixture of cyclopentanone starting material (1.00 equiv.) was dissolved in 1,2-dichloroethane to form a 10% solution. The mixture was heated to 50 °C, before Et_3N (2.00 equiv.) was added in one portion. The mixture was stirred at 50 °C until all starting material was converted into a more polar spot on TLC (LP:EtOAc 3:1) (usually over night). After completion of the reaction, the mixture was extracted twice with 1M HCl, and was washed with brine. Afterwards the organic layer was dried over Na_2SO_4, filtered and evaporated to give crude product. Final purification via column chromatography gave target cyclopent-2-enone.

C.1.3. One Pot Cylisation and Elimination

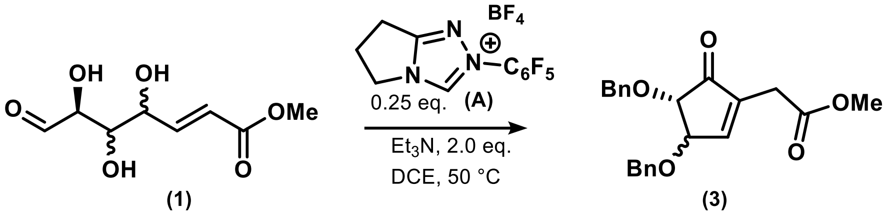

Starting aldehyde (1.00 equiv.) and pre-catalyst salt (A) (0.25 equiv.) were transferred into the reaction vessel. Next, 1,2-dichloroethane was added to form a 10% solution. The insoluble catalyst salt was dispersed by sonication for 10 s, then the reaction mixture was heated to 50 °C, and Et_3N (2.00 equiv.) was added in one portion. The reaction was monitored via TLC and LC-MS (C8, H_2O:ACN 50% - 95%). Upon completion, the reaction mixture was cooled to rt, before it was extracted with ice cold 1M HCl, and washed with brine. Crude material was obtained upon, drying over Na_2SO_4, filtration and evaporation, crude material was obtained almost pure. Finally, column chromatography gave target cyclopent-2-enone.

C.2. Xylose derived Carbocylcles

C.2.1. Methyl [(3S,4R,5S)-3,4,5-tris(benzyloxy)-2-oxocyclopentyl]acetate (2a)

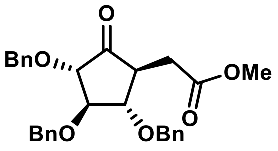

(2a)

Material synthesised following general procedure from (1a) (750 mg, 1.58 mmol, 1.00 equiv.)
Obtained 703 mg (93% yield) as diastereotopic mixture (anti : syn 3:1)

^{1}H NMR (600 MHz, Chloroform-*d*) δ 7.33 – 7.18 (m, 30H, ArH), 5.02 (d, *J* = 11.3 Hz, 1H, A-BnCH$_2$a), 4.86 (d, *J* = 11.9 Hz, 1H, B-BnCH$_2$a), 4.77 (d, *J* = 11.7 Hz, 1H, A-BnCH$_2$a), 4.75 (d, *J* = 11.6 Hz, 1H, A-BnCH$_2$a), 4.66 (d, *J* = 11.3 Hz, 1H, A-BnCH$_2$b), 4.68 – 4.61 (m, 2H, A-BnCH$_2$b, B-BnCH$_2$b), 4.56 (d, *J* = 11.7 Hz, 1H, A-BnCH$_2$b), 4.54 – 4.47 (m, 3H, 2 × B-BnCH$_2$a, B-BnCH$_2$b), 4.36 (d, *J* = 11.9 Hz, 1H, B-BnCH$_2$b), 4.19 (dd, *J* = 8.9, 0.9 Hz, 1H, A4), 4.08 (m, 2H, A3, B5), 4.03 (dt, *J* = 5.0, 1.2 Hz, 1H, B4/B3), 4.00 (dd, *J* = 4.8, 2.9 Hz, 1H, B4/B3), 3.96 (dd, *J* = 9.7, 7.5 Hz, 1H, A5), 3.53 (s, 3H, B-OCH$_3$), 3.50 (s, 3H, A-OCH$_3$), 3.18 (dddd, *J* = 8.5, 7.0, 5.0, 1.8 Hz, 1H, B1), 2.78 (dd, *J* = 17.6, 4.7 Hz, 1H, A-AcCH$_2$a), 2.65 (dd, *J* = 17.4, 5.1 Hz, 1H, B-AcCH$_2$a), 2.54 (dd, *J* = 17.3, 8.1 Hz, 1H, B-AcCH$_2$b), 2.46 (dd, *J* = 17.6, 4.1 Hz, 1H, A-AcCH$_2$b), 2.40 (dt, *J* = 9.2, 4.2 Hz, 1H, A1).

^{13}C{^{1}H} NMR (151 MHz, Chloroform-*d*) δ 211.9 (B2=O), 211.0 (A2=O), 172.7 (B-COOMe), 171.8 (A-COOMe), 138.2 (2 × A-ArC1), 137.7 (B-ArC1), 137.62 (B-ArC1), 137.59 (A-ArC1), 137.56 (B-ArC1), 128.63, 128.60 , 128.57, 128.55, 128.50, 128.41, 128.36, 128.18, 128.11, 128.09, 128.01, 127.94, 127.93, 127.88, 127.82 (15 × A-ArCH, 15 × B-ArCH), 85.6 (A5), 85.5 (A4), 83.3 (B4/B3), 82.7 (B4/B3), 78.7 (A3), 78.3 (B5), 73.5 (A-BnCH$_2$), 73.4 (A-BnCH$_2$), 73.0 (A-BnCH$_2$), 72.8 (B-BnCH$_2$), 72.1 (B-BnCH$_2$), 71.8 (B-BnCH$_2$), 52.1 (A-OCH$_3$), 52.0 (B-OCH$_3$), 49.9 (A1), 46.8 (B1), 31.4 (A-AcCH$_2$), 28.2 (B-AcCH$_2$).

*A = anti; B = syn

HRMS (ESI+) m/z: [M + Na]$^+$ Calcd for $C_{29}H_{30}O_6Na^+$ 497.1946; found 497.1950

C.2.2. Methyl [(3R,4S)-3,4-bis(benzyloxy)-5-oxocyclopent-1-en-1-yl]acetate (xylo / lyxo) (3a)

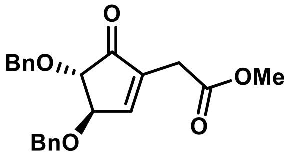

(3a)

Material synthesised following general elimination procedure from xylo-configured triol (2a) (40 mg, 0.084 mmol, 1.00 equiv.)
Obtained 26 mg (86% yield) as yellow oil.

^{1}H NMR (400 MHz, Chloroform-*d*) δ 7.38 – 7.17 (m, 10H, 10 × ArH, H2), 4.97 (d, *J* = 11.6 Hz, 1H, BnCH$_2$a), 4.71 (d, *J* = 11.6 Hz, 1H, BnCH$_2$b), 4.56 (s, 2H, BnCH$_2$), 4.54 (ddd, *J* = 4.0, 2.3, 1.7 Hz, 1H, H3), 4.08 (d, *J* = 2.5 Hz, 1H, H4), 3.63 (s, 3H, -OCH$_3$), 3.18 (q, *J* = 1.3 Hz, 2H, AcCH$_2$).

^{13}C{^{1}H} NMR (101 MHz, Chloroform-*d*) δ 202.1 (C5=O), 169.9 (COOMe), 153.6 (C2), 138.7 (ArC1), 137.4 (Ar-C1), 128.6 (2 × ArCH), 128.5 (2 × ArCH), 128.4 (2 × ArCH), 128.09 (ArC4), 128.07 (ArC4), 128.0 (2 × ArCH), 83.8 (C3), 80.7 (C4), 72.8 (BnCH$_2$), 72.2 (BnCH$_2$), 52.3 (OCH$_3$), 30.1 (AcCH$_2$).

HRMS (ESI+) m/z: [M + Na]$^+$ Calcd for $C_{22}H_{22}O_5Na^+$ 389.1370; found 389.1381

C.2.3. Ethyl [(3S,4R,5S)-3,4,5-tris(benzyloxy)-2-oxocyclopentyl]acetate (2f)

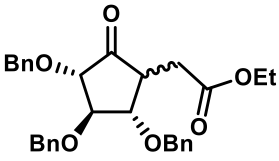

(2f)

Material synthesised following general procedure from (1f) (180 mg, 0.368 mmol, 1.00 equiv.)
Obtained 163 mg (91% yield) as diastereotopic mixture (1:1)

^{1}H NMR (600 MHz, Chloroform-*d*) δ 7.45 – 7.29 (m, 30H, ArH), 5.11 (d, *J* = 11.3 Hz, 1H, $BnCH_2a$), 4.95 (d, *J* = 11.8 Hz, 1H, $BnCH_2a$), 4.89 – 4.81 (m, 2H, 2 × $BnCH_2a$), 4.77 – 4.70 (m, 3H, 3 × $BnCH_2b$), 4.65 (d, *J* = 11.7 Hz, 1H, $BnCH_2b$), 4.63 – 4.55 (m, 3H, 2 × $BnCH_2a$, $BnCH_2b$), 4.46 (d, *J* = 12.0 Hz, 1H, $BnCH_2b$), 4.29 (d, *J* = 8.9 Hz, 1H, A4), 4.21 – 4.14 (m, 2H, B5), 4.14 – 4.00 (m, 7H, A-$OEtCH_2$, B-$OEtCH_2$, A3, B4, B3), 3.27 (q, *J* = 6.8 Hz, 1H, B1), 2.87 (dd, *J* = 17.6, 4.6 Hz, 1H, A-$AcCH_2a$), 2.72 (dd, *J* = 17.3, 5.1 Hz, 1H, B-$AcCH_2a$), 2.63 (dd, *J* = 17.3, 8.1 Hz, 1H, B-$AcCH_2b$), 2.56 – 2.51 (m, 1H, A-$AcCH_2b$), 2.48 (dt, *J* = 8.3, 3.9 Hz, 1H, A1), 1.23 – 1.17 (m, 6H, A-$OEtCH_3$, B-$OEtCH_3$).

^{13}C{^{1}H} NMR (151 MHz, Chloroform-*d*) δ 211.9 (B1=O), 211.0 (A1=O), 172.3 (B-COOEt), 171.4 (A-COOEt), 138.3 (2 × ArC1), 137.72 (ArC1), 137.70 (ArC1), 137.65 (ArC1), 137.61 (ArC1), 128.63, 128.60, 128.59, 128.55, 128.50, 128.40, 128.36, 128.2), 128.10, 128.08, 128.00, 127.95, 127.93, 127.87, 127.79 (15 × A-ArCH, 15 × B-ArCH), 85.7 (A5), 85.6 (A4), 83.4 (B4/B3), 82.8 (B4/B3), 78.8 (A3), 78.4 (B5), 73.6 ($BnCH_2$), 73.4 ($BnCH_2$), 73.0 ($BnCH_2$), 72.8 ($BnCH_2$), 72.1 ($BnCH_2$), 71.9 ($BnCH_2$), 61.1 (A/B-$OEtCH_2$), 60.9 (A/B-$OEtCH_2$), 50.0 (B1), 46.8 (A1), 31.70 (A-$AcCH_2$), 28.5 (B-$AcCH_2$), 14.3 (A/B-$OEtCH_3$), 14.2 (A/B-$OEtCH_3$).

*A = anti; B = syn

HRMS (ESI+) m/z: $[M + Na]^+$ Calcd for $C_{30}H_{32}O_6Na$ 511.2102; found 511.2113

C.2.4. Ethyl [(3R,4S)-3,4-bis(benzyloxy)-5-oxocyclopent-1-en-1-yl]acetate (3f)

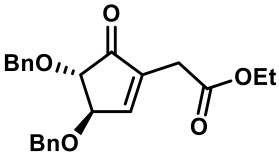

(3f)

Material synthesised following general elimination procedure from (2f) (30 mg, 0.368 mmol, 1.00 equiv.)
Obtained 19 mg (84% yield) as yellow oil

1H NMR (600 MHz, Chloroform-d) δ 7.45 – 7.29 (m, 11H, H3, 10 × ArH), 5.04 (d, J = 11.5 Hz, 1H, $BnCH_2a$), 4.79 (d, J = 11.5 Hz, 1H, $BnCH_2b$), 4.63 (s, 2H, $BnCH_2$), 4.63 – 4.59 (m, 1H, H4), 4.18 – 4.14 (m, 3H, OEt-CH_2, H5), 3.28 – 3.18 (m, 2H, $AcCH_2$), 1.26 (t, *J* = 7.1 Hz, 3H, $OEtCH_3$)

^{13}C{^{1}H} NMR (151 MHz, Chloroform-*d*) δ 202.3 (C1=O), 169.6 (COOEt), 153.6 (H3), 139.0 (ArC1), 137.5 (ArC1), 128.7 (2 × ArCH), 128.6 (2 × ArCH), 128.5 (2 × ArCH), 128.21 (ArC4), 128.19 (ArC4), 128.1 (2 × ArCH), 84.0 (H5), 80.8 (H4), 72.9 ($BnCH_2$), 72.3 ($BnCH_2$), 61.4 ($OEtCH_2$), 30.4 ($AcCH_2$), 14.3 ($OEtCH_3$).

HRMS (ESI+) m/z: $[M + Na]^+$ Calcd for $C_{23}H_{24}O_5Na$ 403.1516; found 403.1525

C.2.5. Benzyl [(3S,4R,5S)-3,4,5-tris(benzyloxy)-2-oxocyclopentyl]acetate (2g)

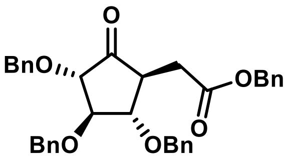

(2g)

Material synthesised following the general procedure from (1g) (40 mg, 0.073 mmol, 1.00 equiv.)
Obtained 36 mg (90% purity, 81% yield) as diastereotopic mixture (0.85:1)

^{1}H NMR (600 MHz, Chloroform-*d*) δ 7.63 – 7.09 (m, 40H, ArH), 5.09 – 4.98 (m, 5H, A-COOOBn-CH$_2$, B-COOOBn-CH$_2$, BnCH$_2$a), 4.93 (d, *J* = 11.9 Hz, 1H, BnCH$_2$a), 4.81 (dd, *J* = 11.6, 6.3 Hz, 2H, BnCH$_2$), 4.73 – 4.67 (m, 2H, BnCH$_2$), 4.61 (d, *J* = 11.7 Hz, 2H, BnCH$_2$), 4.57 – 4.51 (m, 2H, BnCH$_2$), 4.40 (d, *J* = 11.9 Hz, 2H, BnCH$_2$), 4.20 (d, *J* = 8.8 Hz, 1H, A3), 4.17 (dd, *J* = 7.6, 3.1 Hz, 2H, B5, A5), 4.10 (d, *J* = 5.0 Hz, 1H, B4/B3), 4.07 – 4.02 (m, 2H, A4, B4/B3), 3.30 – 3.23 (m, 1H, B1), 2.90 (dd, *J* = 17.7, 4.7 Hz, 1H, A-AcCH$_2$a), 2.78 (dd, *J* = 17.4, 5.0 Hz, 1H, B-AcCH$_2$a), 2.68 (dd, *J* = 17.4, 8.2 Hz, 1H, B-AcCH$_2$b), 2.57 (dd, *J* = 17.7, 4.2 Hz, 1H, A-AcCH$_2$b), 2.49 (dt, *J* = 9.2, 4.3 Hz, 1H, A1).
^{13}C{^{1}H} NMR (151 MHz, Chloroform-*d*) δ 211.8 (B2=O), 210.9 (A2=O), 172.1 (B-COOBn), 171.1 (A-COOBn), 138.21 (ArC1), 138.19 (ArC1), 137.7 (ArC1), 137.63 (2 × ArC1), 137.58 (ArC1), 135.9 (COOB-nArC1), 135.4 (COOBnArC1), 128.8, 128.7, 128.62, 128.59, 128.57, 128.53, 128.50, 128.4, 128.3, 128.13, 128.08, 128.06, 128.0, 127.92, 127.90, 127.86, 127.8 (20 × A-ArCH, 20 × B-ArCH), 85.60 (A3), 85.58 (A4), 83.3 (B4/B3), 82.7 (B4/B3), 78.8 (A5), 78.4 (B5), 73.5 (BnCH$_2$), 73.4 (BnCH$_2$), 73.0 (BnCH$_2$), 72.8 (BnCH$_2$), 72.1 (BnCH$_2$), 71.9 (BnCH$_2$), 67.0 (COOBnCH$_2$), 66.7 (COOBnCH$_2$), 50.0 (A1), 46.8 (B1), 31.6 (A-AcCH$_2$), 28.5 (B-AcCH$_2$).
*A = anti; B = syn
HRMS (ESI+) m/z: [M + Na]$^+$ Calcd for $C_{35}H_{34}O_6Na$ 573.2248, found 573.2262

C.2.6. Benzyl [(3R,4S)-3,4-bis(benzyloxy)-5-oxocyclopent-1-en-1-yl]acetate (3g)

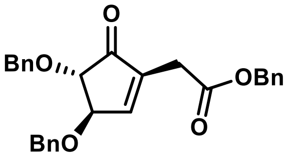

(3g)

Material synthesised following general one-pot procedure from (1g) (40 mg, 0.073 mmol, 1.00 equiv.): Obtained 26 mg (81% yield)
Material synthesised from (2g) following general elimination procedure (20 mg, 0.036 mmol, 1.00 equiv.): Obtained 14 mg (88%)

^{1}H NMR (600 MHz, Chloroform-*d*) δ 7.55 – 7.07 (m, 16H, 15 × ArH, H3), 5.14 (s, 2H, COOBn-CH$_2$), 5.03 (d, *J* = 11.5 Hz, 1H, BnCH$_2$a), 4.78 (d, *J* = 11.6 Hz, 1H, BnCH$_2$b), 4.61 (d, *J* = 1.9 Hz, 2H, BnCH$_2$), 4.60 – 4.59 (m, 2H, H4), 4.14 (d, *J* = 2.4 Hz, 1H, H5), 3.33 – 3.27 (m, 2H, AcCH$_2$).
^{13}C{^{1}H} NMR (151 MHz, Chloroform-*d*) δ 202.2 (C1=O), 169.4 (COOBn), 153.7 (C3), 138.8 (C2), 137.51 (ArC1), 137.50 (ArC1), 135.6 (COOBn-ArC1), 128.8 (2 × ArCH), 128.7 (2 × ArCH), 128.6 (2 × ArCH), 128.54 (ArC4), 128.50 (2 × ArCH), 128.4 (2 × ArCH), 128.20 (ArC4), 128.18 (ArC4), 128.1 (2 × ArCH), 83.9 (C5), 80.8 (C4), 72.9 (BnCH$_2$), 72.2 (BnCH$_2$), 67.1 (COOBn-CH$_2$), 30.4 (AcCH$_2$).
*A = anti; B = syn
HRMS (ESI+) m/z: [M + Na]$^+$ Calcd for $C_{28}H_{26}O_5Na$ 465.1672, found 465.1677

C.2.7. Tert. butyl [(3S,4R,5S)-3,4,5-tris(benzyloxy)-2-oxocyclopentyl]acetate (2h)

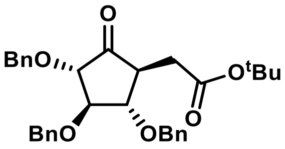

(2h)

Material synthesised following general procedure from (1h) (40 mg, 0.077 mmol, 1.00 equiv.)
Obtained 39 mg (90% purity, 88% yield) as diastereotopic mixture (0.8:1

^{1}H NMR (600 MHz, Chloroform-*d*) δ 7.36 – 7.18 (m, 30H, ArH), 5.03 (d, *J* = 11.3 Hz, 1H, A-BnCH$_2$a), 4.86 (d, *J* = 11.9 Hz, 1H, B-BnCH$_2$b), 4.76 (d, *J* = 11.5 Hz, 1H, B-BnCH$_2$a), 4.75 (d, *J* = 11.8 Hz, 1H, A-BnCH$_2$a), 4.67 (d, *J* = 11.3 Hz, 1H, A-BnCH$_2$b), 4.66 (d, *J* = 11.6 Hz, 1H, B-

BnCH$_2$b), 4.63 (d, *J* = 11.9 Hz, 1H, B-BnCH$_2$b), 4.59 (d, *J* = 11.8 Hz, 1H, A-BnCH$_2$b), 4.53 – 4.48 (m, 3H, B-BnCH$_2$a, A-BnCH$_2$ab), 4.39 (d, *J* = 11.9 Hz, 1H, B-BnCH$_2$b), 4.22 (d, *J* = 8.9 Hz, 1H, A3), 4.11 (dd, *J* = 7.2, 2.5 Hz, 1H, B5), 4.07 (dd, *J* = 8.9, 7.6 Hz, 1H, A4), 4.03 – 4.01 (m, 1H, B4/B3), 4.00 (m, 1H, B4/B3), 3.97 (dd, *J* = 9.5, 7.5 Hz, 1H, A5), 3.14 (tdd, *J* = 7.2, 5.2, 1.7 Hz, 1H, B1), 2.74 (dd, *J* = 17.5, 4.4 Hz, 1H, A-AcCH$_2$a), 2.57 (dd, *J* = 17.3, 5.2 Hz, 1H, B-AcCH$_2$a), 2.50 (dd, *J* = 17.3, 7.8 Hz, 1H, B-AcCH$_2$b), 2.38 (dd, *J* = 17.5, 4.1 Hz, 1H, A-AcCH$_2$b), 2.34 (dt, *J* = 8.9, 4.2 Hz, 1H, A1), 1.32 (s, 9H, B-O^{t}BuCH$_3$), 1.28 (s, 9H, A-O^{t}BuCH$_3$).

^{13}C{^{1}H} NMR (151 MHz, Chloroform-*d*) δ 212.0 (B2=O), 211.1 (A2=O), 171.6 (B-COOtBu), 170.4 (A-COOtBu), 138.30 , 138.27 , 137.78 , 137.76 , 137.73 , 137.66 (6 × ArC1), 128.60 , 128.57 , 128.54 , 128.48, 128.36, 128.32, 128.08 , 128.05 , 128.02 , 127.98 , 127.91 , 127.87 , 127.7 (15 × A-ArCH, 15 × B-ArCH), 85.7 (A4, A3), 83.4 (B4/B3), 82.8 (B4/B3), 81.6 (A-O^{t}BuC), 80.9 (B-O^{t}BuC), 78.8 (A5), 78.6 (B5), 73.5 (BnCH$_2$), 73.3 (BnCH$_2$), 73.0 (BnCH$_2$), 72.8 (BnCH$_2$), 72.1 (BnCH$_2$), 71.9 (BnCH$_2$), 50.2 (A1), 46.9 (B1), 32.9 (A-AcCH$_2$), 29.6 (B-AcCH$_2$), 28.2 (OtBuCH$_3$).

*A = anti; B = syn

HRMS (ESI+) m/z: [M + K]$^+$ Calcd for $C_{32}H_{36}O_6K^+$ 555.2143; found 555.2144

C.2.8. [(3R,4S)-3,4-bis(benzyloxy)-5-oxocyclopent-1-en-1-yl]acetonitrile (3i)

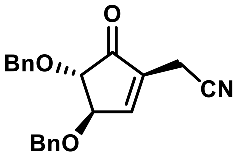

(3i)

Material synthesised following general one-pot procedure from (1i) (40 mg, 0.09 mmol, 1.00 equiv.): Obtained 25 mg (83% yield)
(Remark: Uneliminated triol not obtainable without significant amounts of elimination taking place)

^{1}H NMR (600 MHz, Chloroform-*d*) δ 7.47 (q, *J* = 1.7 Hz, 1H, H2), 7.43 – 7.29 (m, 10H, ArH), 5.01 (d, *J* = 11.5 Hz, 1H, BnCH$_2$a), 4.78 (d, *J* = 11.6 Hz, 1H, BnCH$_2$b), 4.65 – 4.57 (m, 3H, BnCH$_2$, H3), 4.15 (d, *J* = 2.3 Hz, 1H, H4), 3.31 (dt, *J* = 20.0, 2.0 Hz, 1H, AcCH$_2$a), 3.24 (dt, *J* = 20.0, 1.8 Hz, 1H, AcCH$_2$b).

^{13}C{^{1}H} NMR (151 MHz, Chloroform-*d*) δ 200.6 (C5=O), 153.9 (C2), 137.1 (ArC1), 136.0 (ArC1), 128.8 (2 × ArCH), 128.7 (2 × ArCH), 128.5 (2 × ArCH), 128.4 (ArC4), 128.4 (ArC4), 128.2 (2 × ArCH), 115.8 (CN), 83.9 (C4), 80.5 (C3), 73.1 (BnCH$_2$), 72.6 (BnCH$_2$), 14.7 (AcCH$_2$).

HRMS (ESI+) m/z: [M + Na]$^+$ Calcd for $C_{21}H_{19}NO_3Na$ 356.1257; found 356.1260

C.2.9. Methyl [(3S,4R,5S)-2-oxo-3,4,5-tris(trimethylsiloxy)cyclopentyl]acetate (2e)

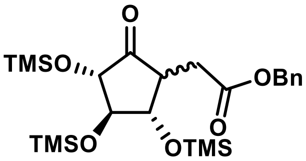

(2e)

Material synthesised following general procedure from (1e) (40 mg, 0.09 mmol, 1.00 equiv.): Obtained 24mg of material consisting of target product as syn/anti mixture together with other unidentified impurities after column chromatography. Presumeably different hydrolysed species.
1H NMR shows clearly that cyclisation occurred, but the substance could not be fully characterized from the obtained compound mixture.

^{1}H NMR (600 MHz, Chloroform-*d*) δ 4.61 (dd, *J* = 4.0, 1.8 Hz, 1H), 4.30 (d, *J* = 4.4 Hz, 1H), 4.25 (dt, *J* = 5.5, 1.9 Hz, 1H), 4.19 (dd, *J* = 9.7, 0.7 Hz, 1H), 3.93 (dt, *J* = 3.8, 1.7 Hz, 1H), 3.87 (dd, *J* = 9.6, 7.7 Hz, 1H), 3.73 (s, 1H), 3.70 (s, 1H), 3.68 (s, 1H), 3.67 (s, 2H), 3.67 (s, 3H), 3.17 – 3.12 (m, 1H), 2.95 (d, *J* = 4.3 Hz, 0H), 2.94 – 2.91 (m, 1H), 2.76 (dd, *J* = 16.5, 4.8 Hz, 1H), 2.66 (dd, *J* = 17.2, 3.7 Hz, 1H), 2.63 – 2.58 (m, 1H), 2.39 (dd, *J* = 16.5, 10.5 Hz, 1H), 2.25 (dtd, *J* = 9.5, 4.3, 0.7 Hz, 1H), 0.19 (s, 9H), 0.18 (s, 5H), 0.17 (s, 9H), 0.13 (s, 9H), 0.13 (s, 5H), 0.11 (s, 5H).

C.3. Other Stereoconfigurations

C.3.1. Methyl [(3S,4R,5R)-3,4,5-tris(benzyloxy)-2-oxocyclopentyl]acetate (*Lyxo*) (2b)

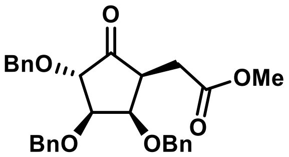

(2b)

Material synthesised following the general procedure from (1b) (210 mg, 0.443 mmol, 1.00 equiv.)
Obtained 194 mg (92% yield) as diastereotopic mixture (1:4)

^{1}H NMR (600 MHz, Chloroform-*d*) δ 7.34 (ddtd, *J* = 21.6, 13.7, 6.8, 4.0 Hz, 30H, ArH), 5.04 (d, *J* = 11.5 Hz, 1H, A-BnCH$_2$a), 4.95 (d, *J* = 11.5 Hz, 1H, A-BnCH$_2$a), 4.89 (d, *J* = 11.7 Hz, 1H, B-BnCH$_2$a), 4.78 (s, 2H, A-BnCH$_2$ab), 4.76 (d, *J* = 11.5 Hz, 1H, A-BnCH$_2$b), 4.68 (d, *J* = 11.7 Hz, 1H, B-BnCH$_2$b), 4.64 (d, *J* = 11.9 Hz, 1H, B-BnCH$_2$a), 4.59 (app. q, *J* = 12.5 Hz, 4H, B-BnCH$_2$a, 2 × B- BnCH$_2$b, A-BnCH$_2$b), 4.38 (t, *J* = 3.9 Hz, 1H, A5), 4.31 (d, *J* = 8.9 Hz, 1H, A3), 4.28 (dd, *J* = 6.1, 1.9 Hz, 1H, B3), 4.13 – 4.10 (m, 1H, A4), 4.08 (dd, *J* = 6.0, 4.8 Hz, 1H, B4), 4.04 – 4.00 (m, 1H, B5), 3.65 (s, 3H, B-OCH$_3$), 3.63 (s, 3H, A-OCH$_3$), 3.06 (ddt, *J* = 7.6, 5.6, 2.8 Hz, 1H, B1), 2.83 – 2.75 (m, 2H, A1, A-AcCH$_2$a), 2.65 (dd, *J* = 16.7, 5.1 Hz, 1H, B-AcCH$_2$a), 2.62 – 2.57 (m, 1H, A-AcCH$_2$b), 2.47 (dd, *J* = 16.7, 7.9 Hz, 1H, B-AcCH$_2$b).

^{13}C{^{1}H} NMR (151 MHz, Chloroform-*d*) δ 211.8 (A2=O), 211.5 (B2=O), 172.6 (A-COOMe), 171.8 (B-COOMe), 138.2 (A-ArC1), 138.1 (A-ArC1), 137.9 (B-ArC1), 137.80 (B-ArC1), 137.78 (A-ArC1), 137.6 (B-ArC1), 128.63, 128.59, 128.57, 128.56, 128.5, 128.3, 128.2, 128.14, 128.12, 128.07, 128.01, 128.00, 127.94, 127.91, 127.6 (15 × A-ArCH, 15 × B-ArCH), 83.4 (A4), 82.6 (A3), 81.9 (B3), 78.9 (B4), 78.0 (B5), 74.4 (A-BnCH$_2$), 74.1 (A5), 73.4 (A-BnCH$_2$), 72.8 (B-BnCH$_2$), 72.4 (A-BnCH$_2$), 72.0 (B-BnCH$_2$), 71.7 (B-BnCH$_2$), 52.1 (B-OCH$_3$), 52.0 (A-OCH$_3$), 49.3 (A1), 48.4 (B1), 32.4 (B-AcCH$_2$), 28.2 (A-AcCH$_2$).

HRMS (ESI+) m/z: $[M + Na]^+$ Calcd for $C_{29}H_{30}O_6Na^+$ 497.1946; found 497.1951

C.3.2. Methyl [(1S,3S,4S,5S)-3,4,5-tris(benzyloxy)-2-oxocyclopentyl]acetate (*Ribo*) (2c)

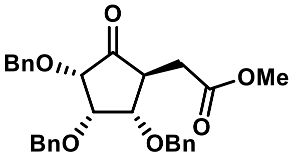

(2c)

Material synthesised following the general procedure from (1c) (104 mg, 0.22 mmol, 1.00 equiv.)
Obtained 83 mg (83% yield) as single isomer as yellow oil

^{1}H NMR (600 MHz, Chloroform-*d*) δ 7.41 – 7.27 (m, 15H, 15 × ArH), 4.97 (d, *J* = 12.1 Hz, 1H, $BnCH_2a$), 4.88 (d, *J* = 12.2 Hz, 1H, $BnCH_2a$), 4.79 (d, *J* = 12.2 Hz, 1H, $BnCH_2b$), 4.72 (d, *J* = 12.1 Hz, 1H, $BnCH_2b$), 4.63 (d, *J* = 11.8 Hz, 1H, $BnCH_2a$), 4.36 (d, *J* = 11.8 Hz, 1H, $BnCH_2b$), 4.33 (t, *J* = 3.6 Hz, 1H, H4), 4.16 (d, *J* = 4.0 Hz, 1H, H3), 3.98 (dd, *J* = 10.4, 3.2 Hz, 1H, H5), 3.54 (s, 3H, OCH_3), 2.92 (dd, *J* = 17.9, 4.7 Hz, 1H, $AcCH_2a$), 2.82 (dt, *J* = 10.3, 4.3 Hz, 1H, H1), 2.61 (dd, *J* = 17.9, 3.8 Hz, 1H, $AcCH_2b$).
$^{13}C\{^1H\}$ NMR (151 MHz, Chloroform-*d*) δ 211.1 (C2=O), 172.1 (COOMe), 138.4 (ArC1), 137.7 (ArC1), 137.6 (ArC1), 128.49 (2 × ArCH), 128.46 (2 × ArCH), 128.3 (2 × ArCH), 128.0 (2 × ArCH), 127.94 (ArC4), 127.86 (ArC4), 127.8 (2 × ArCH), 127.74 (2 × ArCH), 127.63 (ArC4), 83.96 (C3), 76.5 (C5), 73.7 (C4), 73.3 ($BnCH_2$), 73.2 ($BnCH_2$), 71.3 ($BnCH_2$), 51.8 (OCH_3), 47.0 (C1), 30.6 ($AcCH_2$).
HRMS (ESI+) m/z: $[M + Na]^+$ Calcd for $C_{29}H_{30}O_6Na^+$ 497.1946; found 497.1961

C.3.3. Methyl [(3S,4S,5R)-3,4,5-tris(benzyloxy)-2-oxocyclopentyl]acetate (Arabino) (2d)

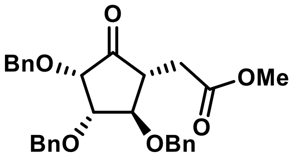

(2d)

Material synthesised following the general procedure from (1d) (610 mg, 1.29 mmol, 1.00 equiv.)
Obtained 587 mg (96% yield) as diastereotopic mixture (5:1 anti:syn)

^{1}H NMR (600 MHz, Chloroform-*d*) δ 7.70 – 6.95 (m, 30H, ArH), 4.85 (d, *J* = 12.0 Hz, 2H, A-$BnCH_2a$, B-$BnCH_2a$), 4.71 – 4.60 (m, 6H, 2 × A-$BnCH_2a$, A-$BnCH_2b$, B-$BnCH_2a$, 2 × B-$BnCH_2b$), 4.55 (dd, *J* = 14.4, 12.1 Hz, 2H, 2 × $BnCH_2b$), 4.47 (d, *J* = 11.8 Hz, 1H, B-CH_2a), 4.44 (dd, *J* = 4.5, 1.6 Hz, 1H, A3), 4.41 (d, *J* = 11.7 Hz, 1H, B-CH_2a), 4.23 (dd, *J* = 6.4, 1.6 Hz, 1H, B5), 4.18 (d, *J* = 4.8 Hz, 1H, B4), 4.07 (dd, *J* = 4.9, 1.7 Hz, 1H, B3), 4.05 (dt, *J* = 4.3, 1.7 Hz, 1H, A4), 3.89 (t, *J* = 2.1 Hz, 1H, A5), 3.68 (s, 3H, A-OCH_3), 3.61 (s, 3H, B-OCH_3), 3.05 (ddd, *J* = 10.3, 6.4, 4.0 Hz, 1H, B1), 2.85 (dd, *J* = 16.7, 4.5 Hz, 2H, A-$AcCH_2a$), 2.80 (ddt, *J* = 8.1, 4.3, 1.9 Hz, 1H, A1), 2.72 (dd, *J* = 17.1, 4.0 Hz, 1H, B-$AcCH_2a$), 2.61 (dd, *J* = 17.1, 10.1 Hz, 1H, B-$AcCH_2b$), 2.41 (dd, *J* = 16.7, 10.2 Hz, 2H, A-$AcCH_2b$).
$^{13}C\{^1H\}$ NMR (151 MHz, Chloroform-*d*) δ 212.6 (B2=O), 211.8 (A2=O), 172.6 (B-COOMe), 172.5 (A-COOMe), 137.9 (B-ArC1), 137.8 (A-ArC1), 137.7 (A-ArC1), 137.64 (B-ArC1), 137.56 (B-ArC1), 137.5 (A-ArC1), 128.63, 128.60, 128.58, 128.57, 128.42, 128.29, 128.17, 128.09, 128.06, 128.02, 128.00, 127.92, 127.86 (30 × ArCH), 81.8 (A3), 81.0 (B4), 79.5 (A5), 78.4 (A4), 76.4 (B5), 75.5 (B3), 73.0 (B-$BnCH_2$), 72.8 (B-$BnCH_2$), 72.7 (A-$BnCH_2$), 72.6 (B-$BnCH_2$), 72.4 (A-$BnCH_2$), 71.8 (A-$BnCH_2$), 52.0 (A-OCH_3), 51.9 (B-OCH_3), 46.9 (A1), 45.6 (B1), 33.9 (A-$AcCH_2$), 29.1 (B-$AcCH_2$).
HRMS (ESI+) m/z: $[M + Na]^+$ Calcd for $C_{29}H_{30}O_6Na^+$ 497.1946; found 497.1955

C.3.4. Methyl [(3S,4S)-3,4-bis(benzyloxy)-5-oxocyclopent-1-en-1-yl]acetate (Ribo / Arabino) (3c)

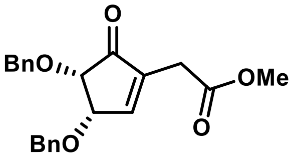

(3c)

Material synthesised following general one-pot procedure from arabino aldehyde (1d) (100 mg, 0.211 mmol, 1.00 equiv.) to give 61 mg of target material (64% yield)
Material synthesized following general elimination procedure from ribo triol (2r) (20 mg, 0.055 mmol, 1.00 equiv.) to give 12 mg of target material 78%

yield. (Benzylalcohol co-eluted with target material. Yields given are corrected for the benzylalcohol amount according to NMR)

^{1}H NMR (600 MHz, Chloroform-*d*) δ 7.34 – 7.18 (m, 10H, 10 × ArH), 4.93 (d, *J* = 11.5 Hz, 1H, $BnCH_2a$), 4.67 (d, *J* = 11.5 Hz, 1H, $BnCH_2b$), 4.52 – 4.51 (m, 2H, $BnCH_2$), 4.51 – 4.48 (m, 1H, H3), 4.05 (d, *J* = 2.5 Hz, 1H, H4), 3.59 (s, 3H, OCH_3), 3.14 (q, *J* = 1.7 Hz, 2H, $AcCH_2$).

$^{13}C\{^{1}H\}$ NMR (151 MHz, Chloroform-*d*) δ 202.25 (C5=O), 169.99 (COOMe), 153.68 (C1), 138.80 (ArC1), 137.48 (d, *J* = 1.1 Hz(ArC1)), 128.67 (2 × ArCH), 128.61 (2 × ArCH), 128.49 (2 × ArCH), 128.20 (ArC4), 128.17 (ArC4), 128.09 (2 × ArCH), 83.95 (H4), 80.78 (H3), 72.89 ($BnCH_2$), 72.29 ($BnCH_2$), 52.36 (OCH_3), 30.17 ($AcCH_2$).

HRMS (ESI+) m/z: $[M + Na]^+$ Calcd for $C_{22}H_{22}O_5Na$ 389.1370; found: 389.1379

C.4. Prediction of syn/anti ratio between C1 and C5

Assignment of syn/anti isomers is based on ribo-configured carbocycle (2r), as this was obtained as a single isomer. From a structural standpoint, we expected the syn-isomer to be either unstable or unlikely to form, due to it having all 4 substituents facing the same direction. For this compound NMR gives a coupling contanst between H1 and H5 of the cycle of > 10 Hz, indicating a potential anti arrangement of these protons.

Comparing the NMRs of the other stereoconfigurations gave two distinct patterns of "signal types".

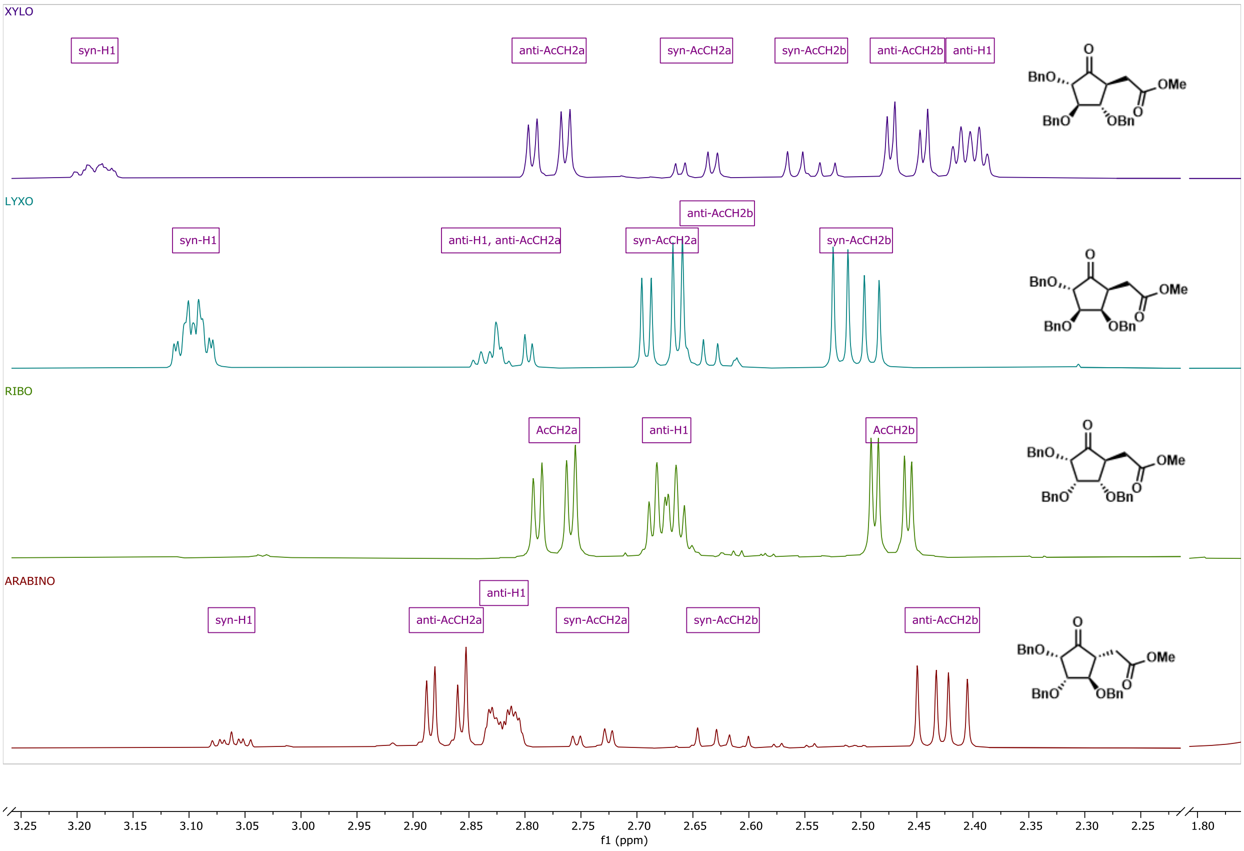

Figure S 2. *Stackplot of all stereopatterns of carbocycles. Showing two distinct sets of isomers in the spectrum.*

With the H1 of the presumed *anti*-arranged isomers always shifted highfield compared to the *syn* counterpart. Signal shape was recognizable throughout the whole series.

NOESY NMR of xylo-derived carbocycles (2a) revealed a clear correlation between H1 and H4 of the cycle only for one of the isomers. This correlation is highly likely to stem from an anti alignment of H1 and H5, bringing H1 and H4 to the same side of the plane. Thus, we identified the major isomers of 2x, 2r and 2a to be anti-arranged, while lyxo shows a preference for the syn configuration. Further, we reasoned about

the mechanistic origin of the observed selectivity. Using a Zimmerman Traxler like model of the transition the observed reaction outcome is always the one, where the amount of axially configured groups is minimised

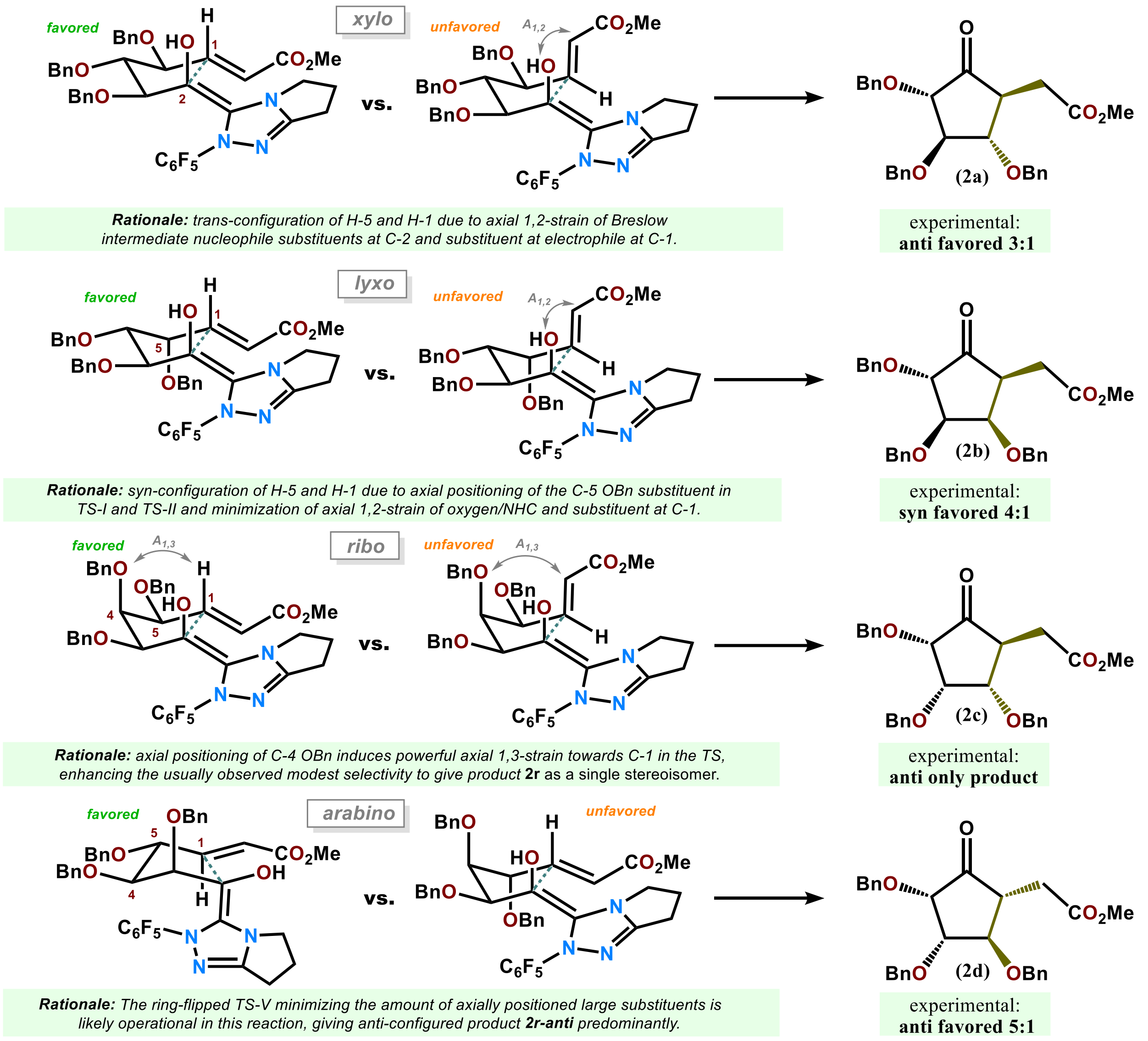

Figure S3. Transition state models for each stereo configuration and explanation of the observed selectivity.

D. Spectra

D.1. Xylose Derived Compounds

D.1.1. Methyl 2,3,4-Tri-*O*-benzyl-β-D-xylopyranoside (4a)

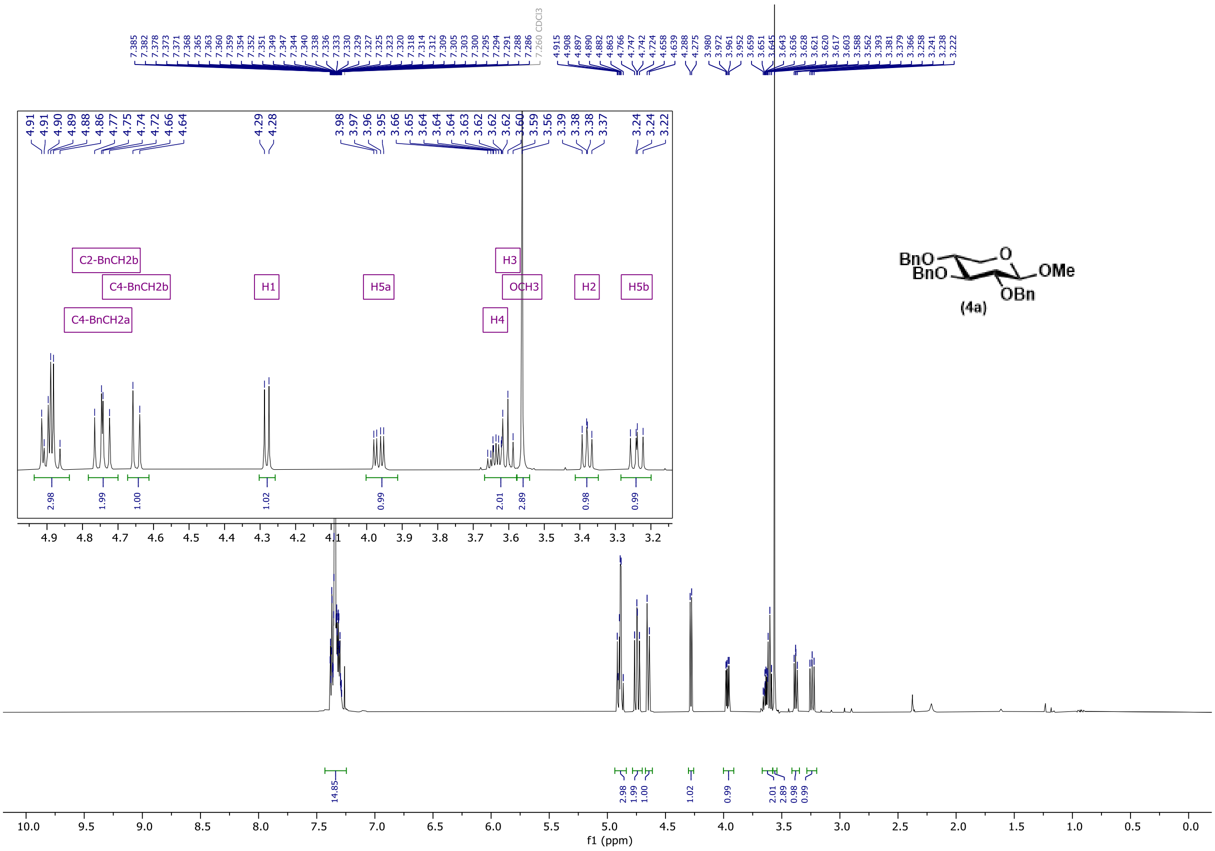

Figure S4. 600 MHz ^{1}H-NMR of (4a)

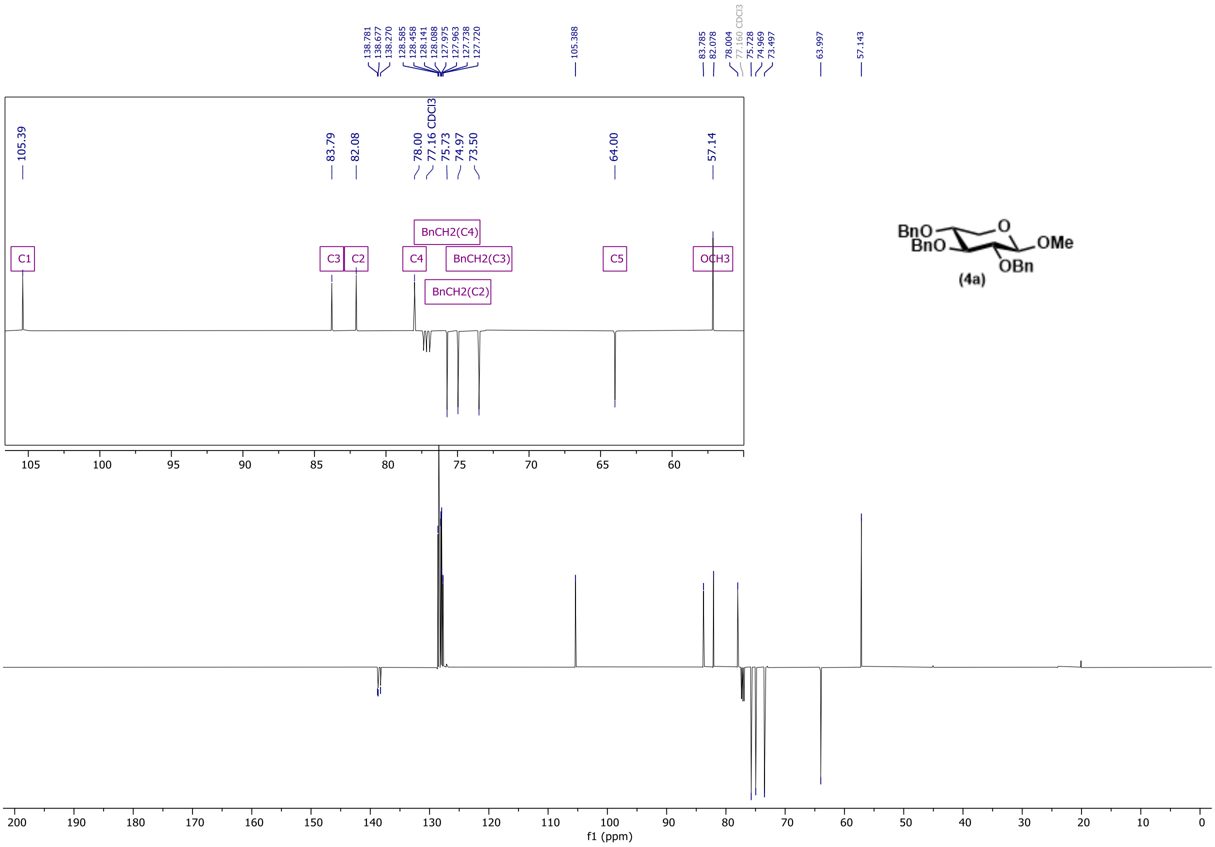

Figure S5. 151 MHz ^{13}C{^{1}H}-NMR (DEPTQ) of (4a)

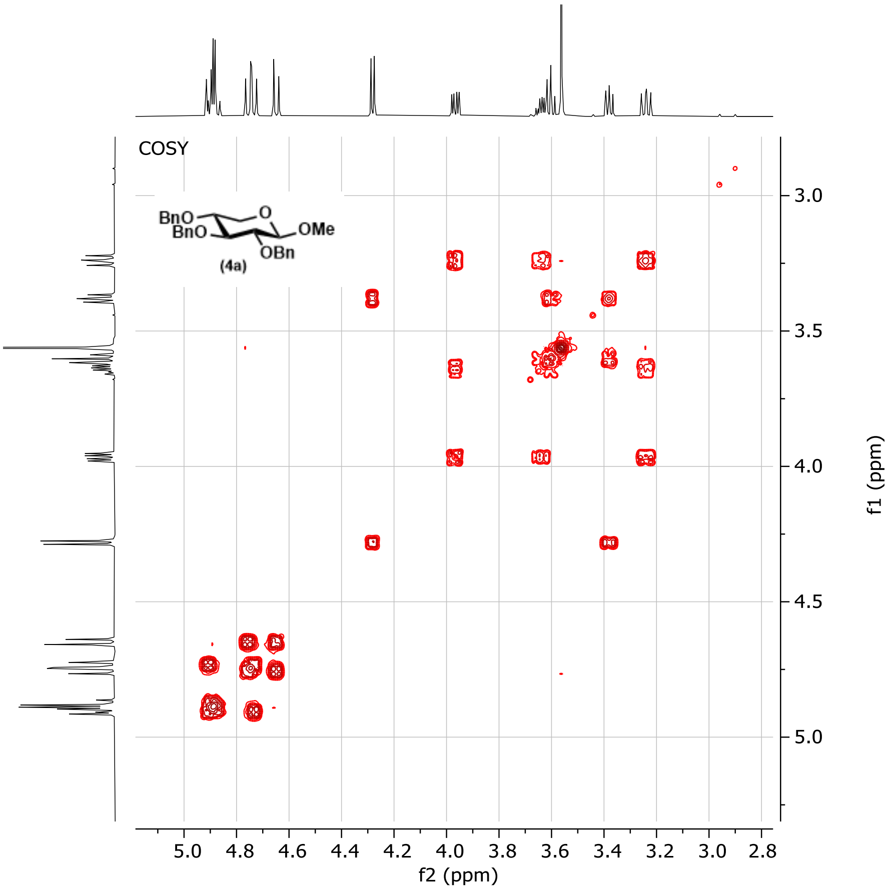

Figure S6. *COSY NMR of (4a)*

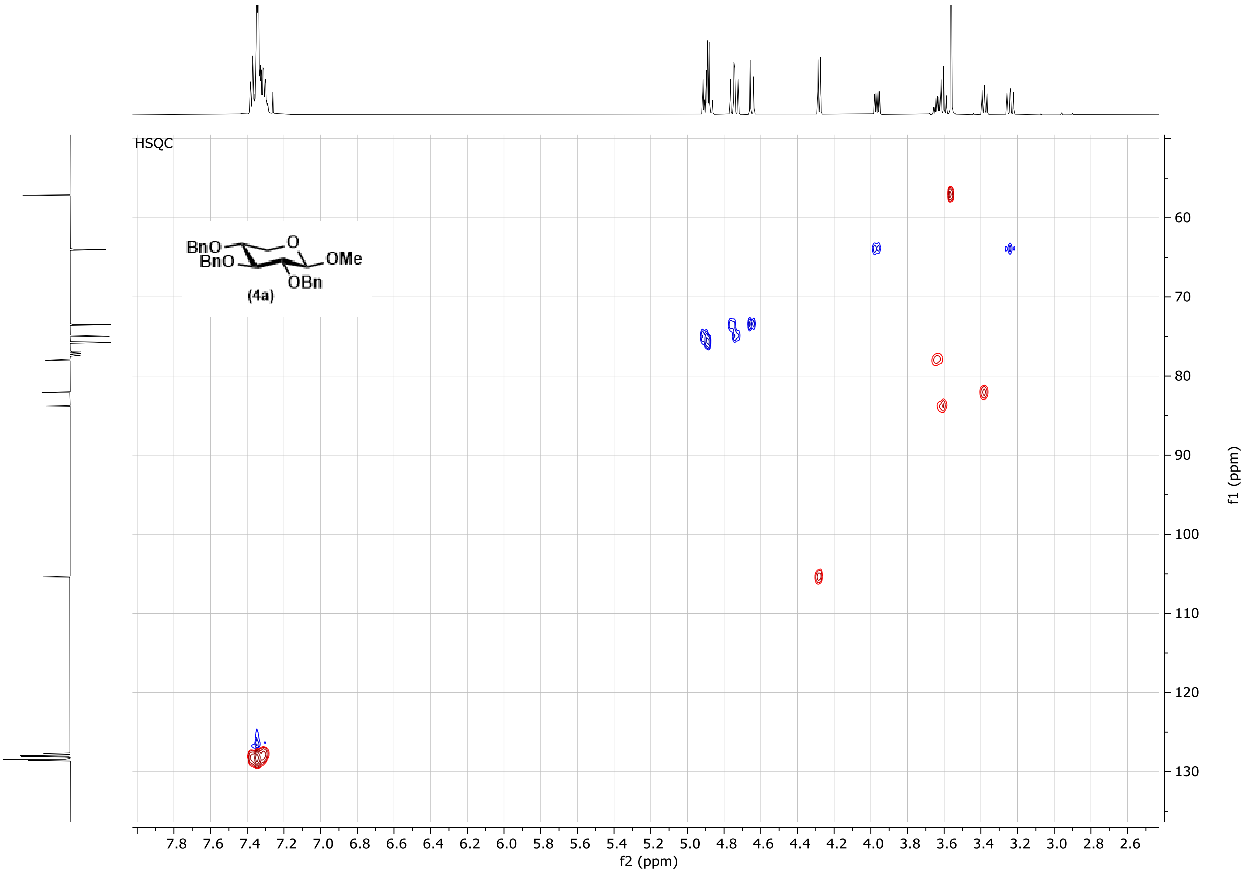

Figure S7. *HSQC NMR of (4a)*

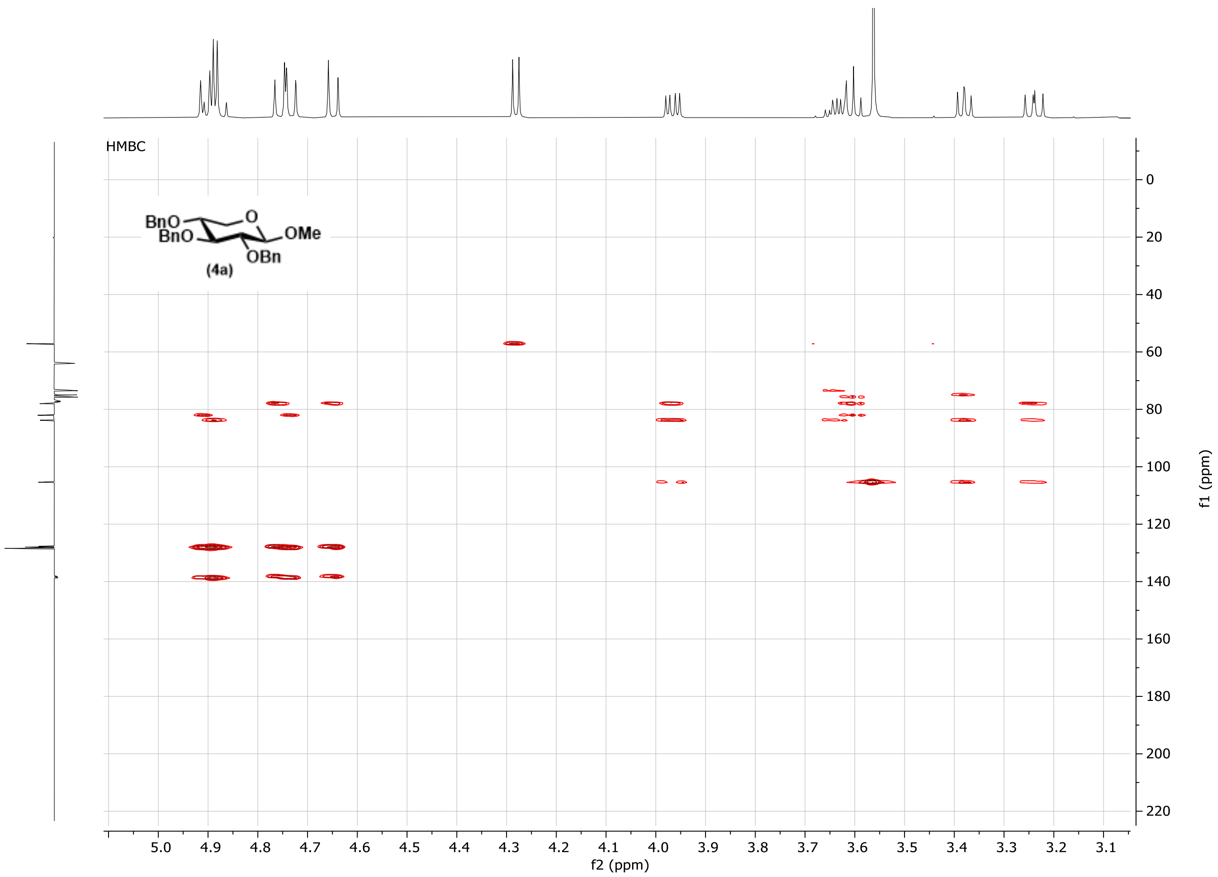

Figure S8. *HMBC NMR of (4a)*

D.1.2. 2,3,4-tri-*O*-benzyl-β-D-xylopyranose (5a)

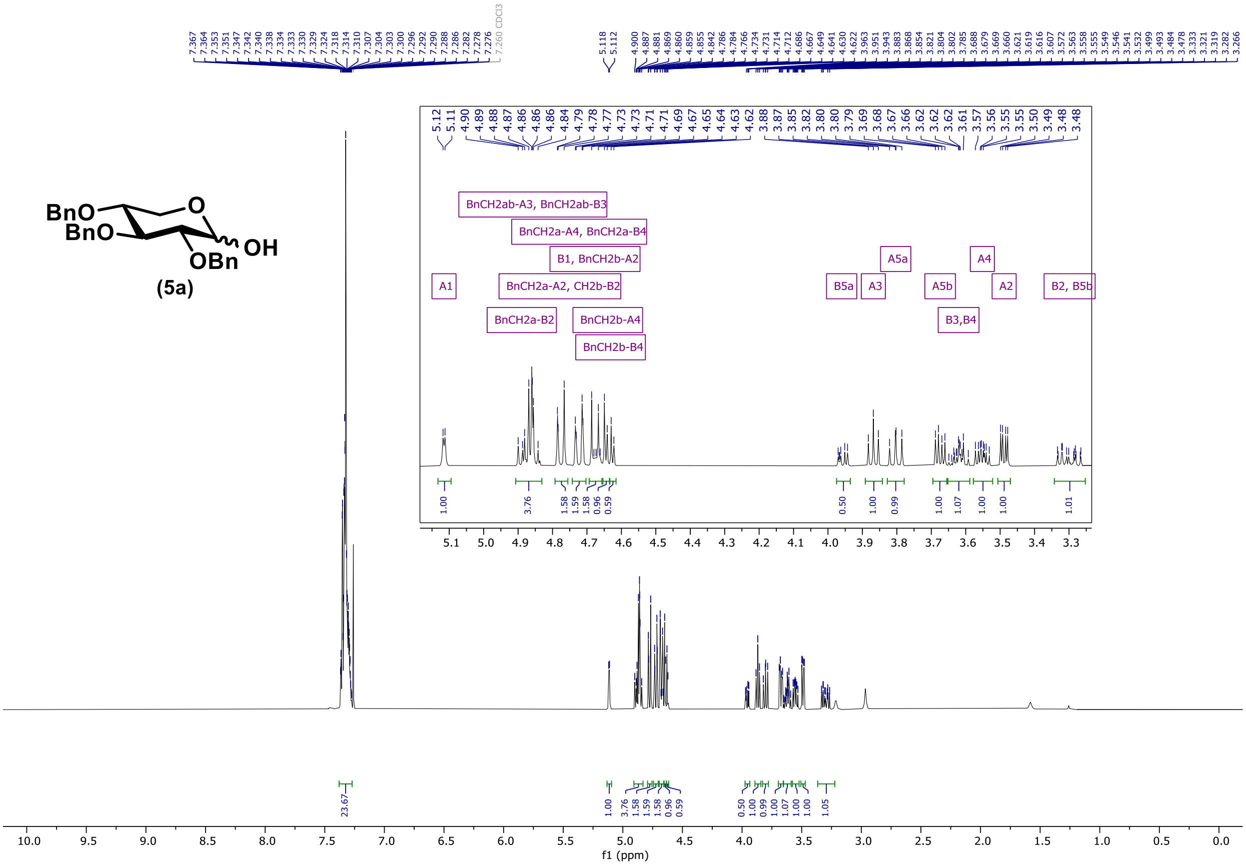

Figure S9. *600 MHz* ^{1}*H-NMR of (5a)*

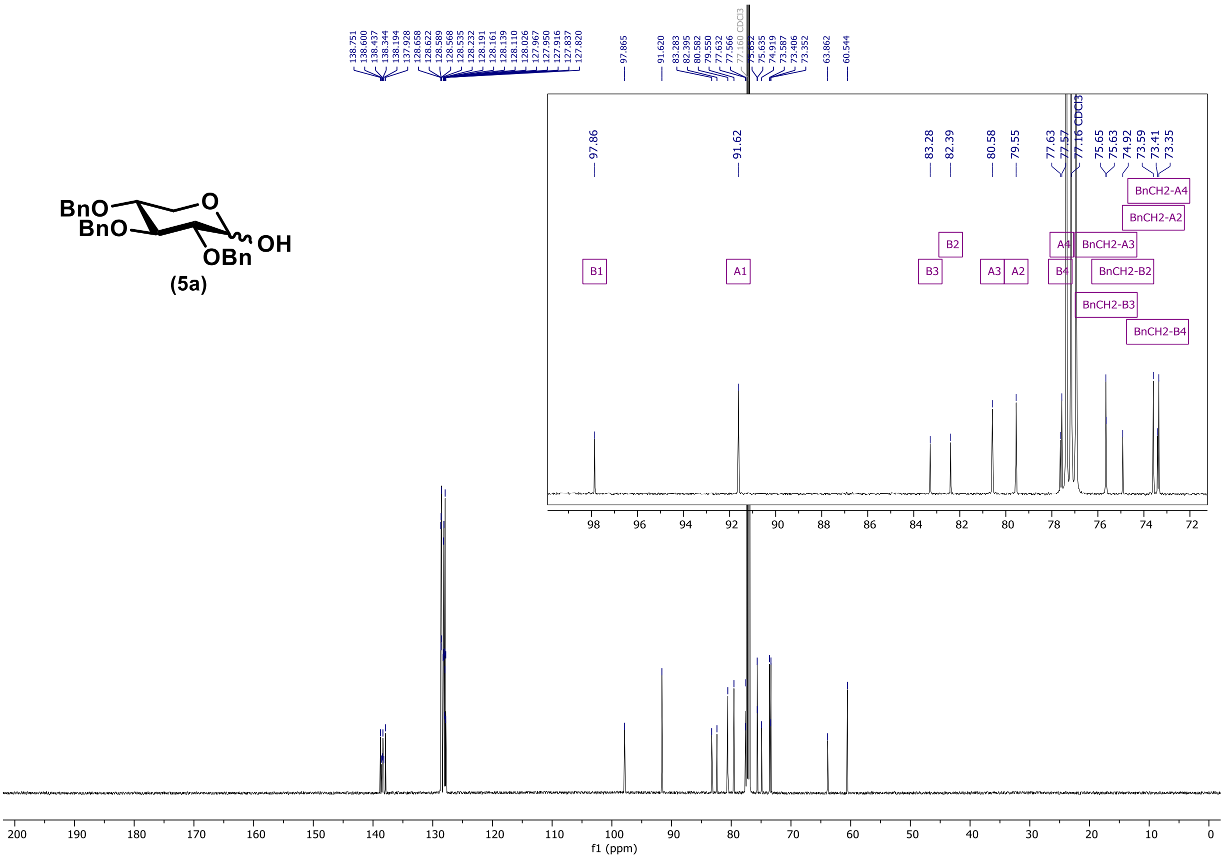

Figure S10. *151 MHz* ^{13}*C{*^{1}*H}-NMR of (5a)*

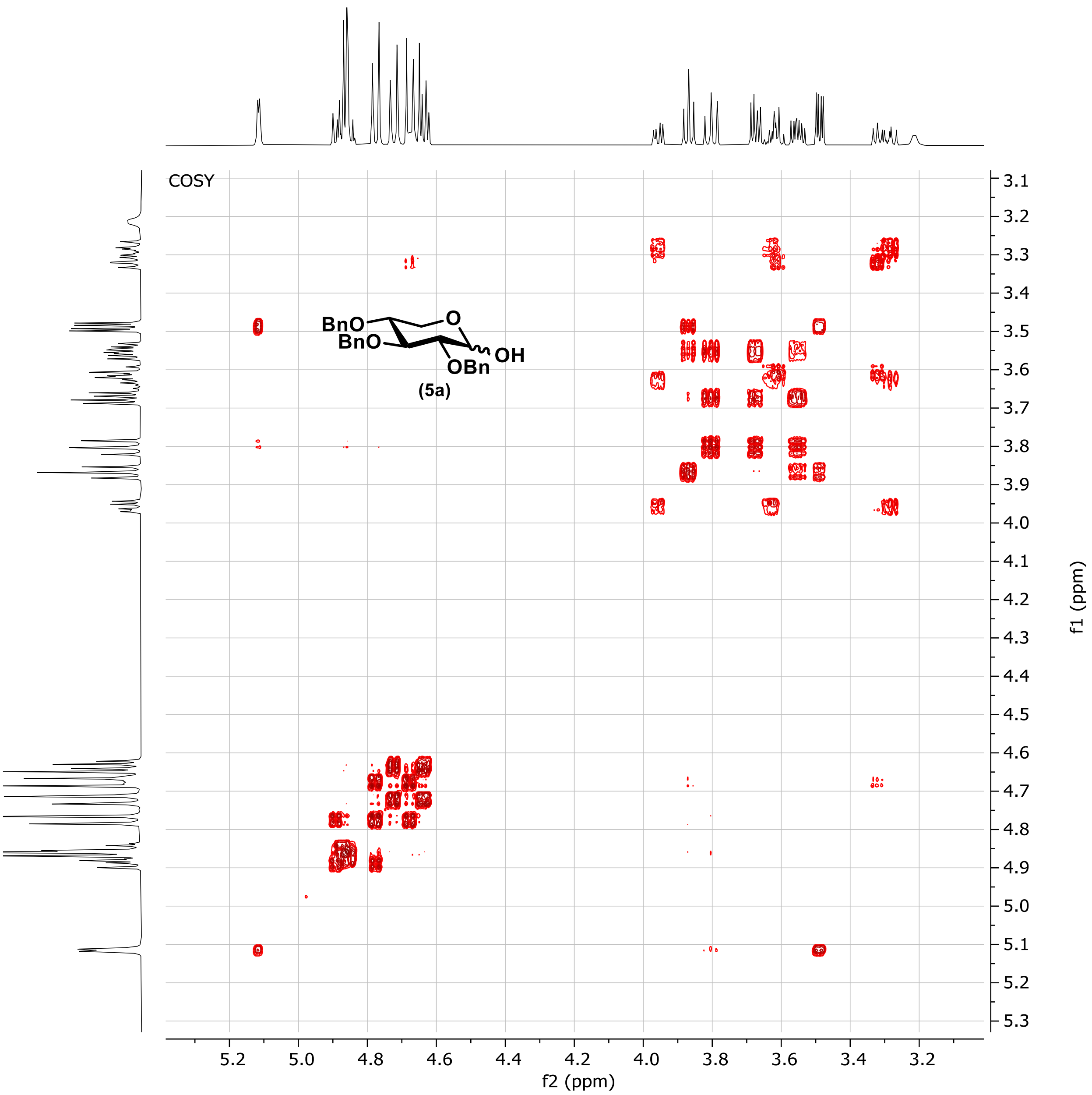

Figure S11. COSY NMR of (5a)

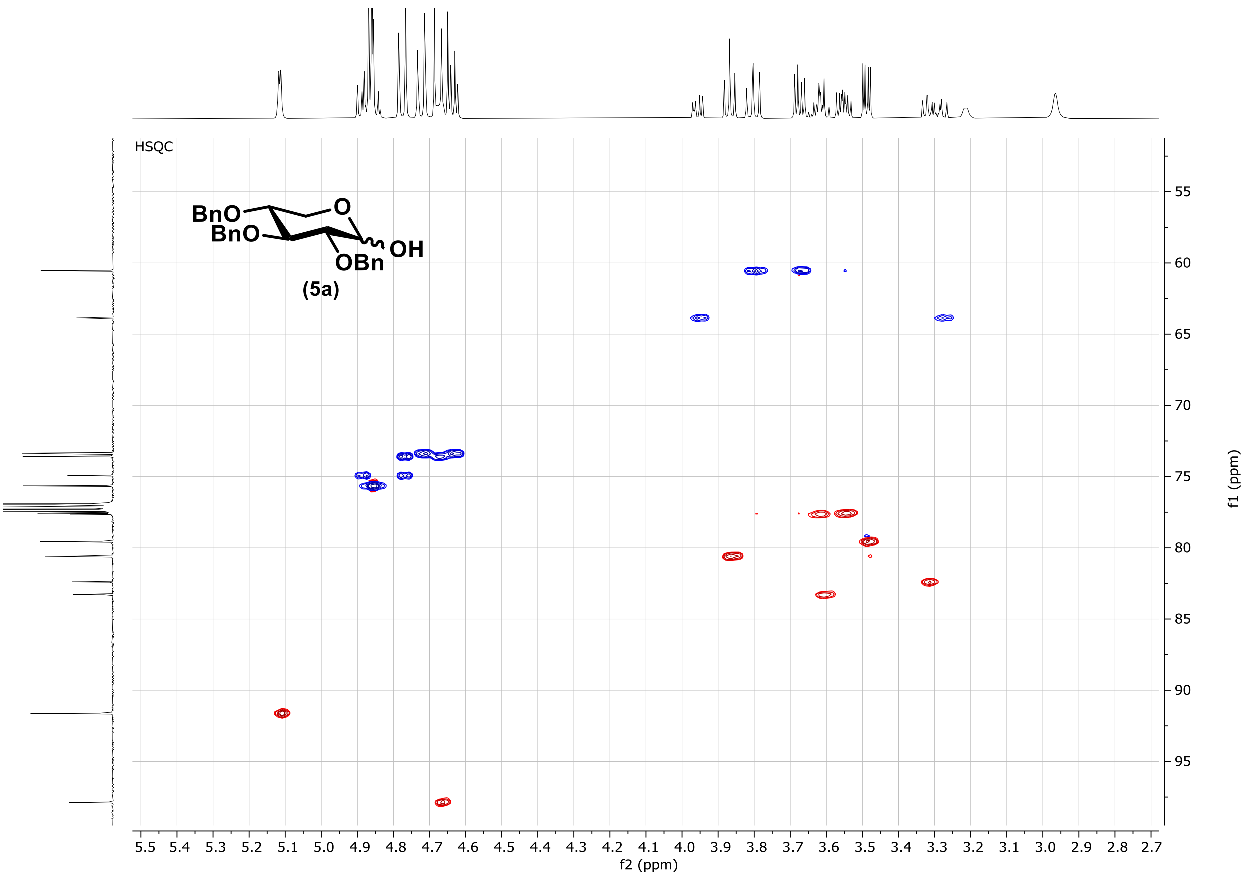

Figure S12. *HSQC NMR of (5a)*

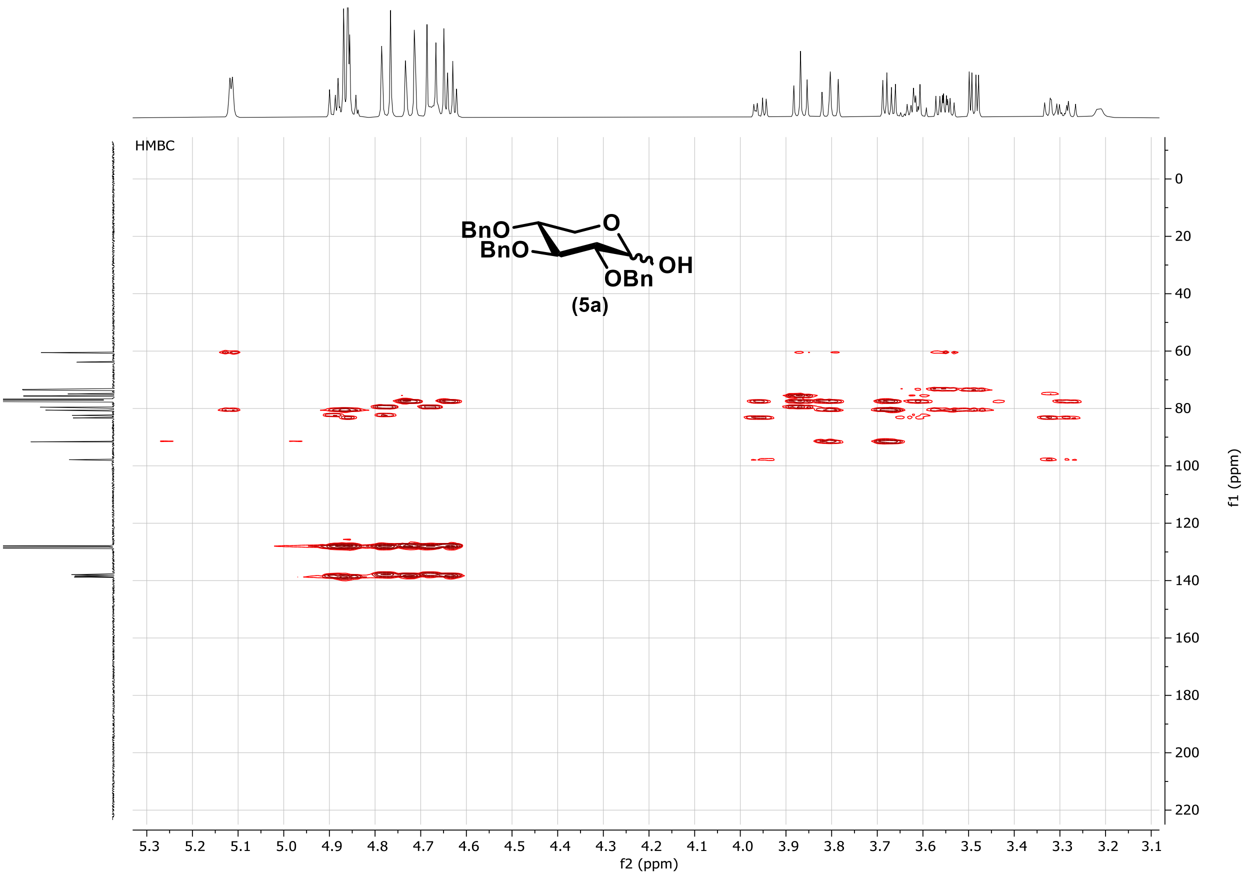

Figure S13. *HMBC NMR of (5a)*

D.1.3. Methyl (4S,5R,6R,E)-4,5,6-tris(benzyloxy)-7-hydroxyhept-2-enoate (6a-e)

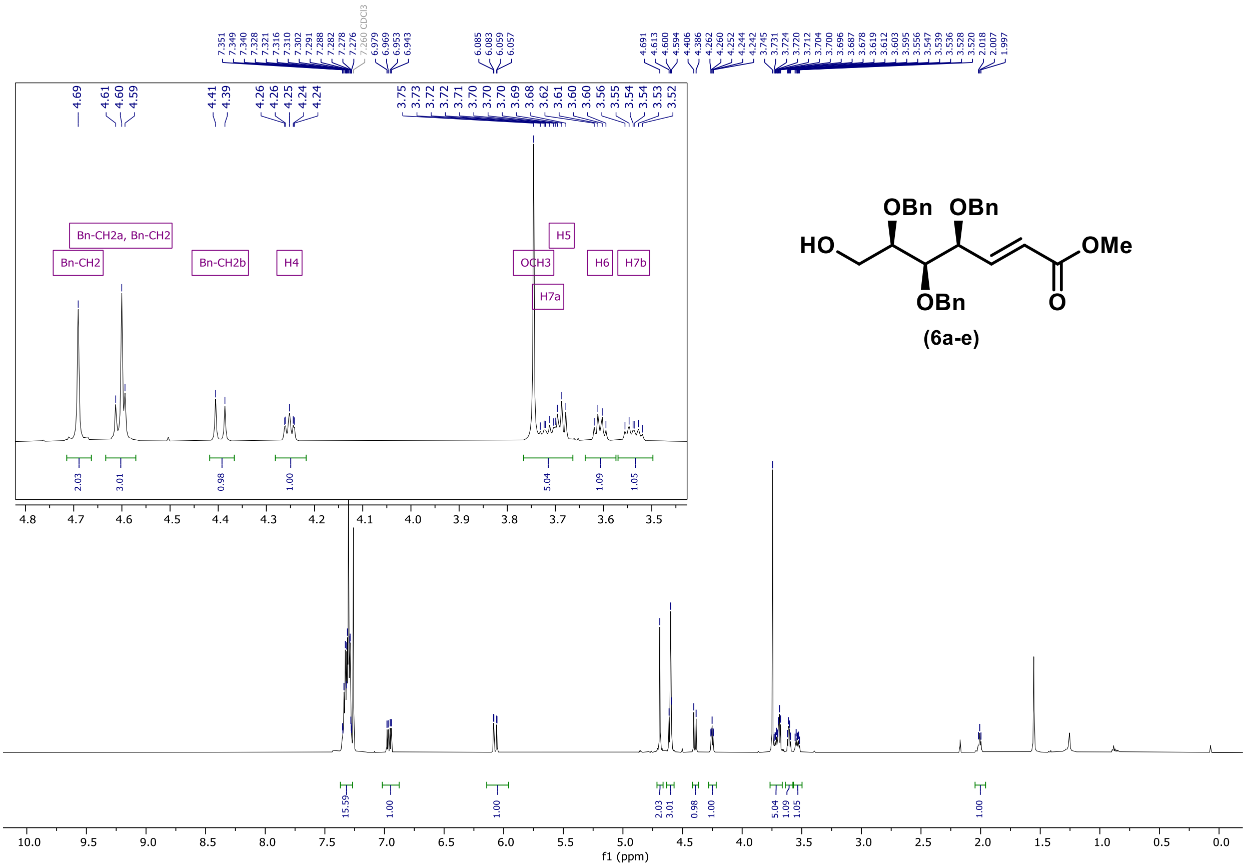

Figure S14. *600 MHz ¹H-NMR of (6a-e)*

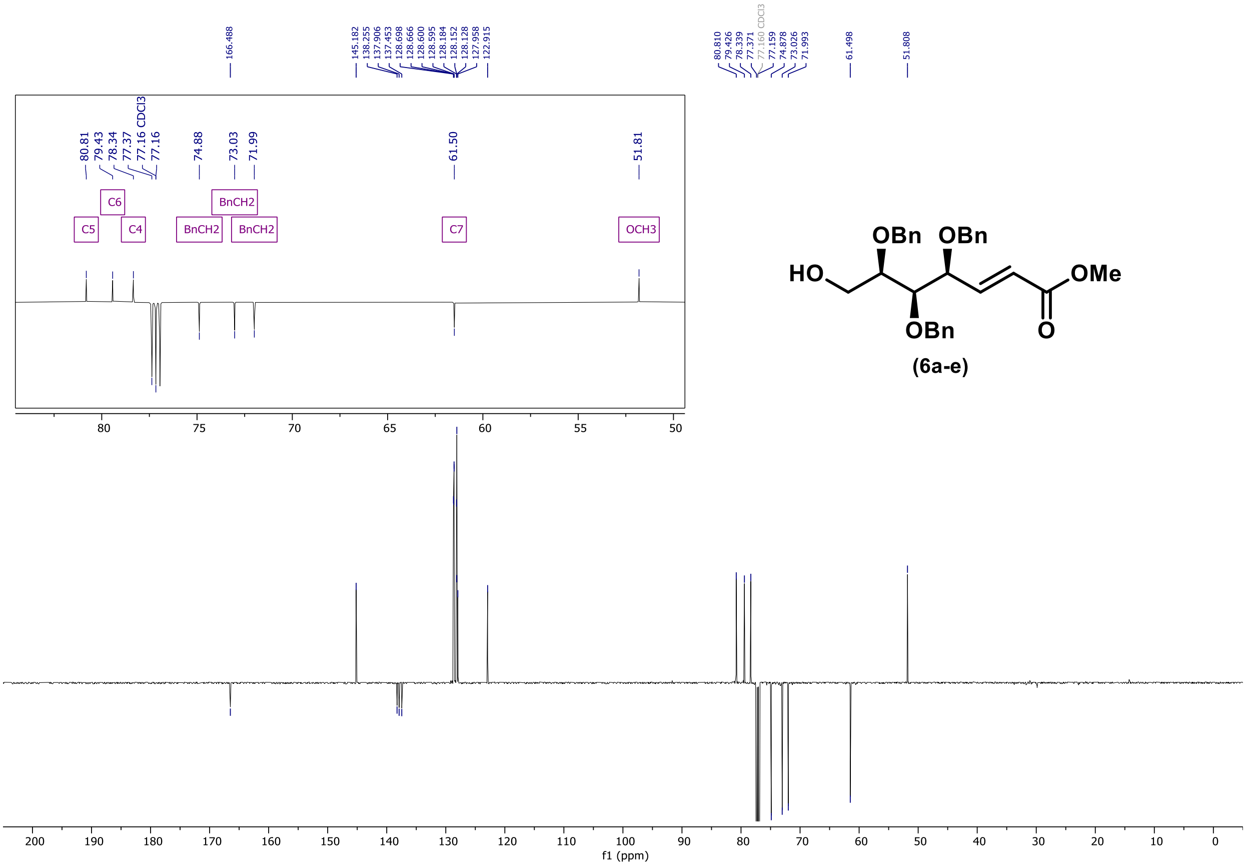

Figure S15. *151 MHz $^{13}C\{^{1}H\}$-NMR of (6a-e)*

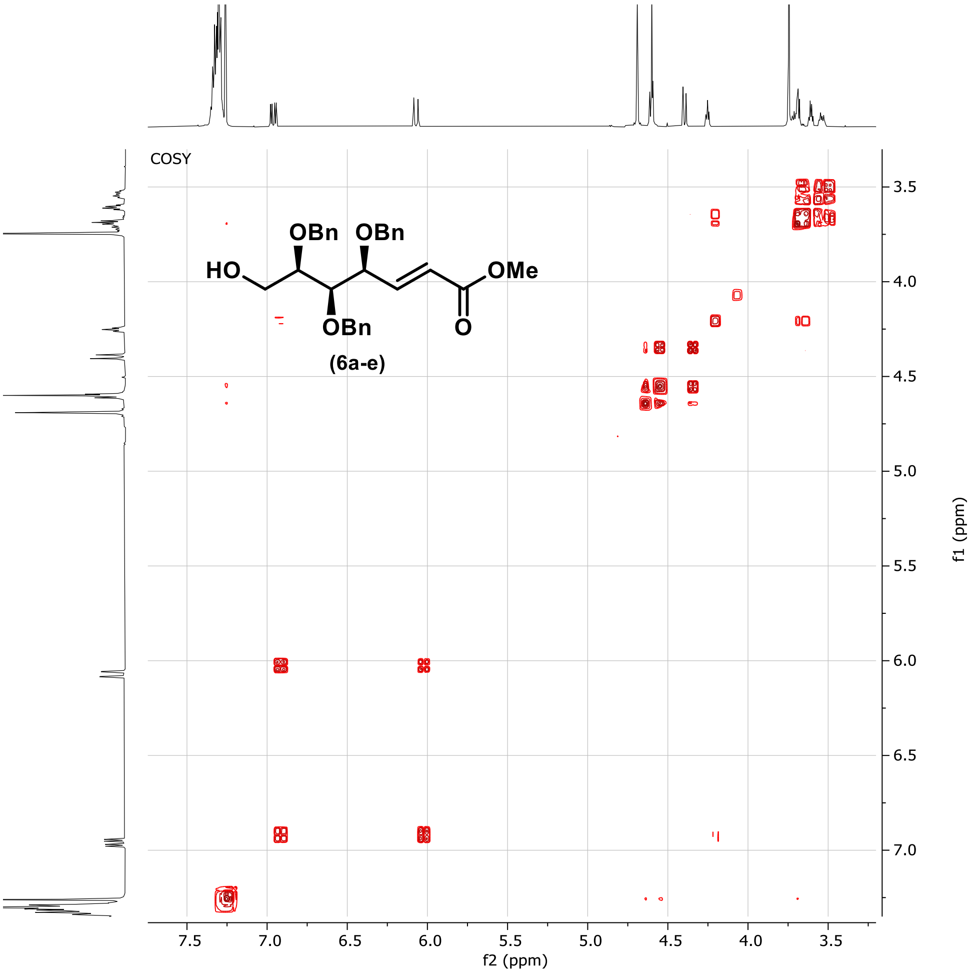

Figure S16. *COSY NMR of (6a-e)*

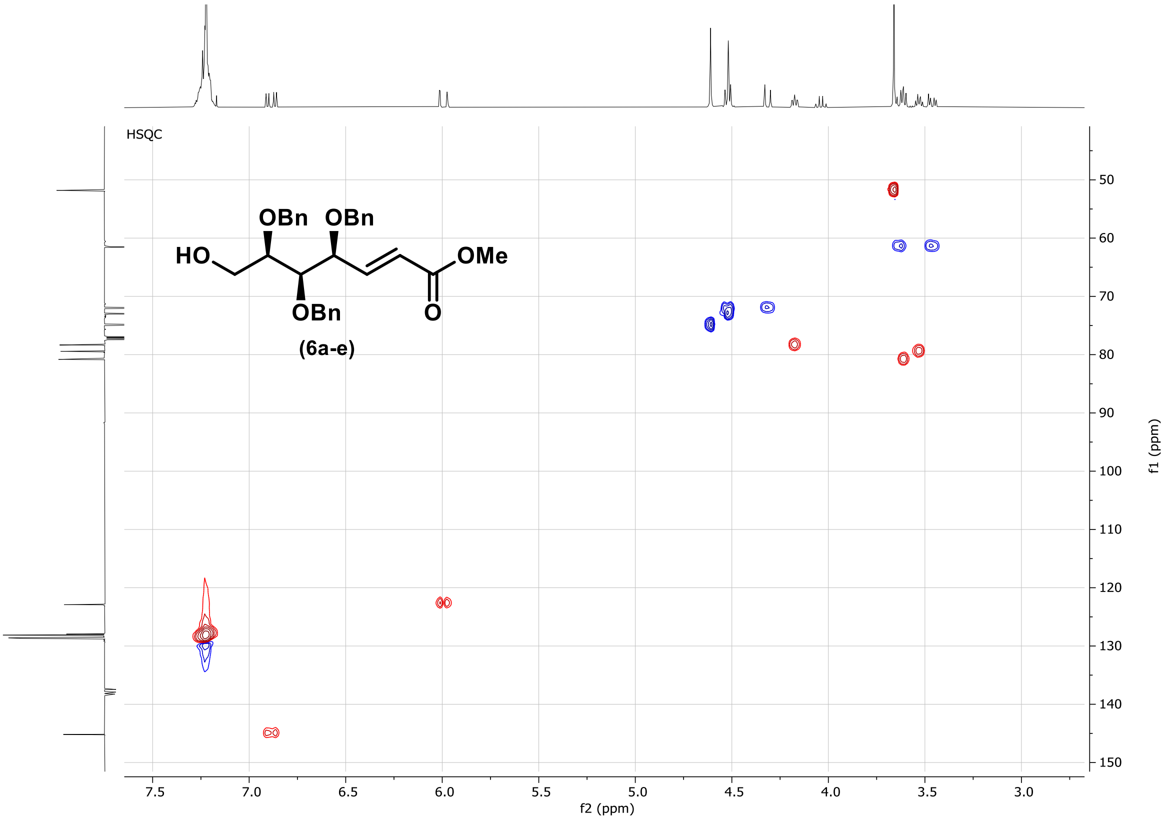

Figure S17. *HSQC NMR of (6a-e)*

D.1.4. Methyl (4S,5R,6R,Z)-4,5,6-tris(benzyloxy)-7-hydroxyhept-2-enoate (6a-z)

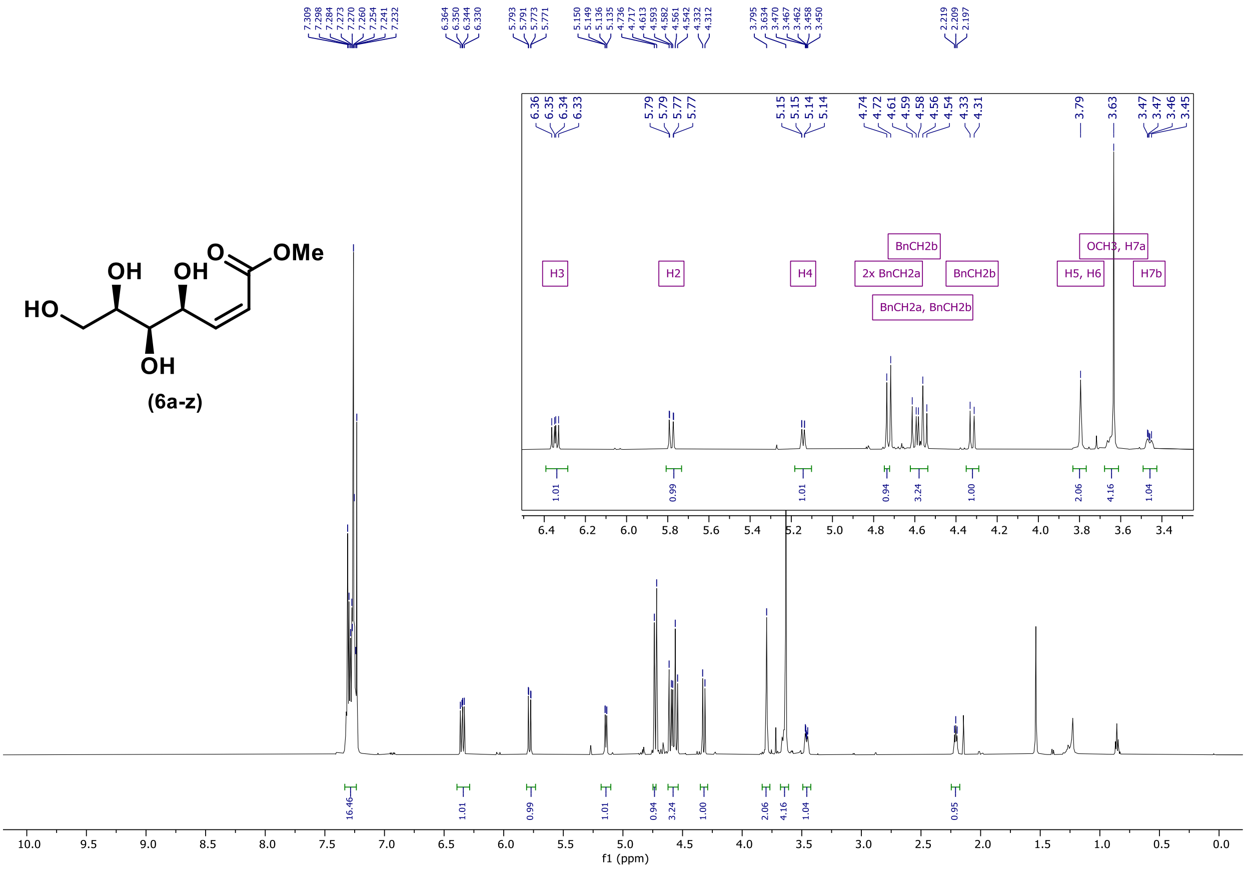

Figure S18. *600 MHz ^{1}H-NMR of (6a-z)*

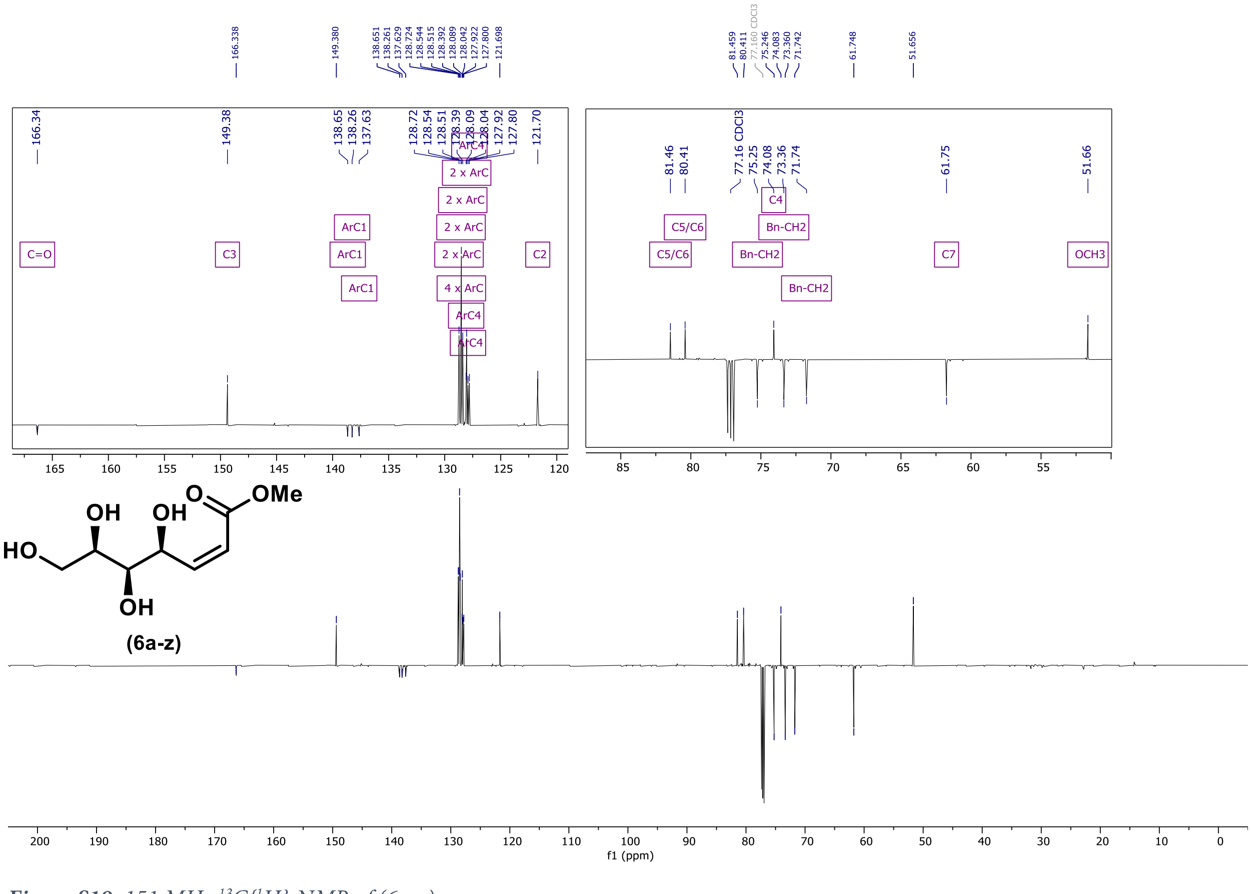

Figure S19. *151 MHz ^{13}C{^{1}H}-NMR of (6a-z)*

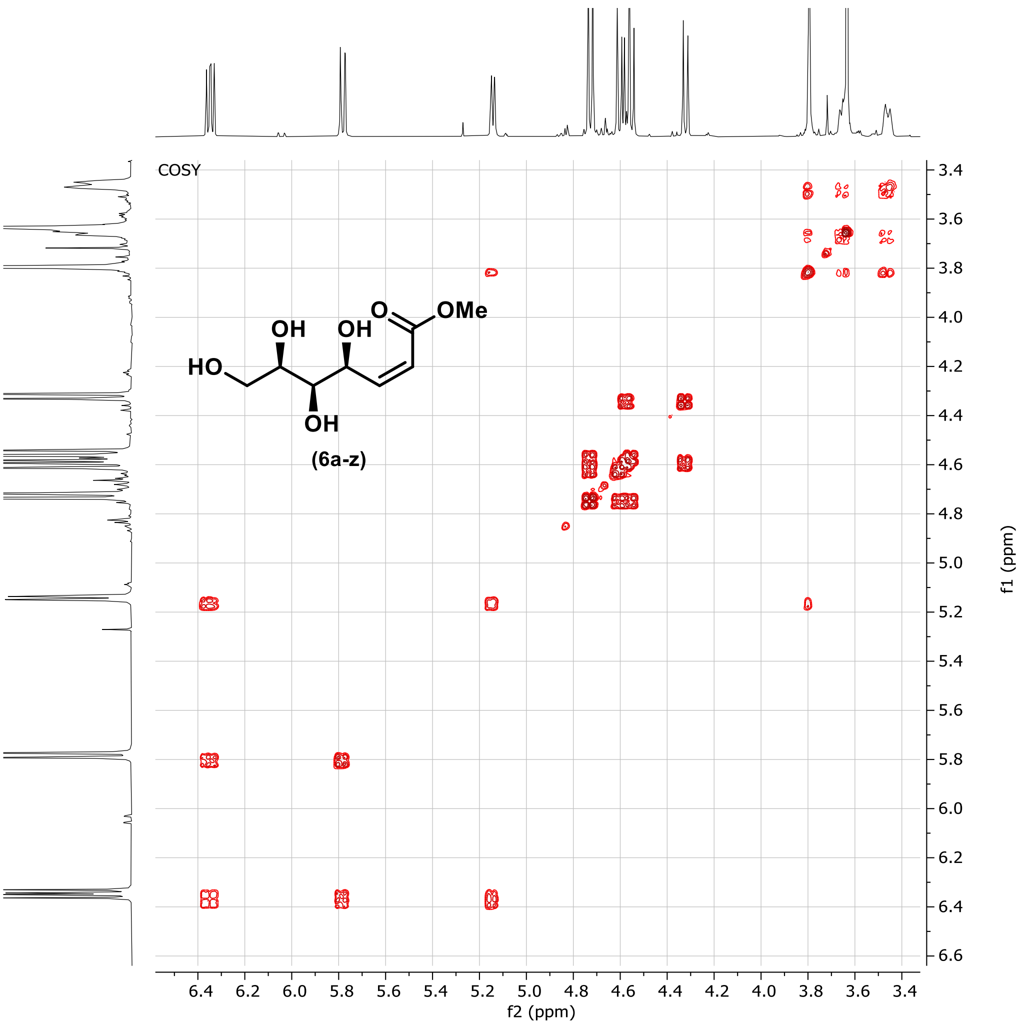

Figure S20. *COSY NMR of (6a-z)*

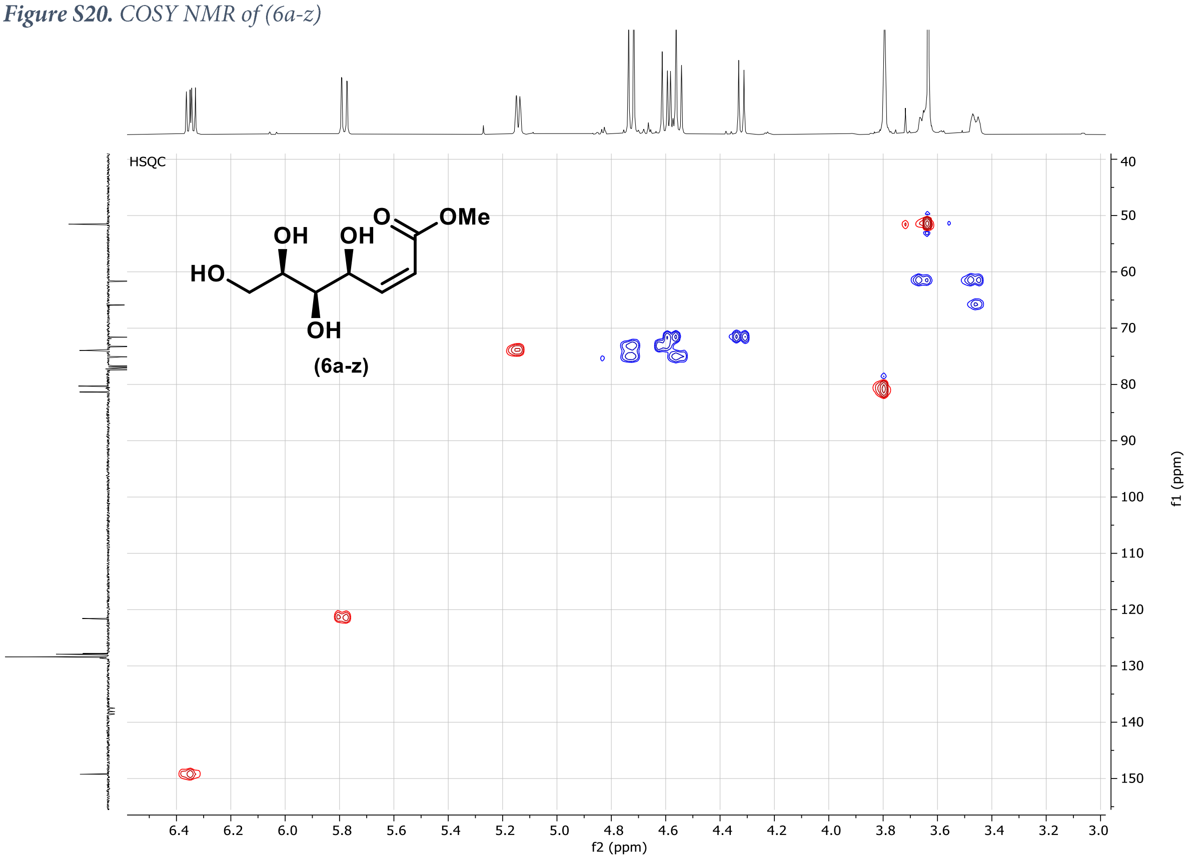

Figure S21. *HSQC NMR of (6a-z)*

D.1.5. Methyl (4S, 5R, 6S, E)-4,5,6-tris(benzyloxy)-7-oxohept-2-enoate (1a)

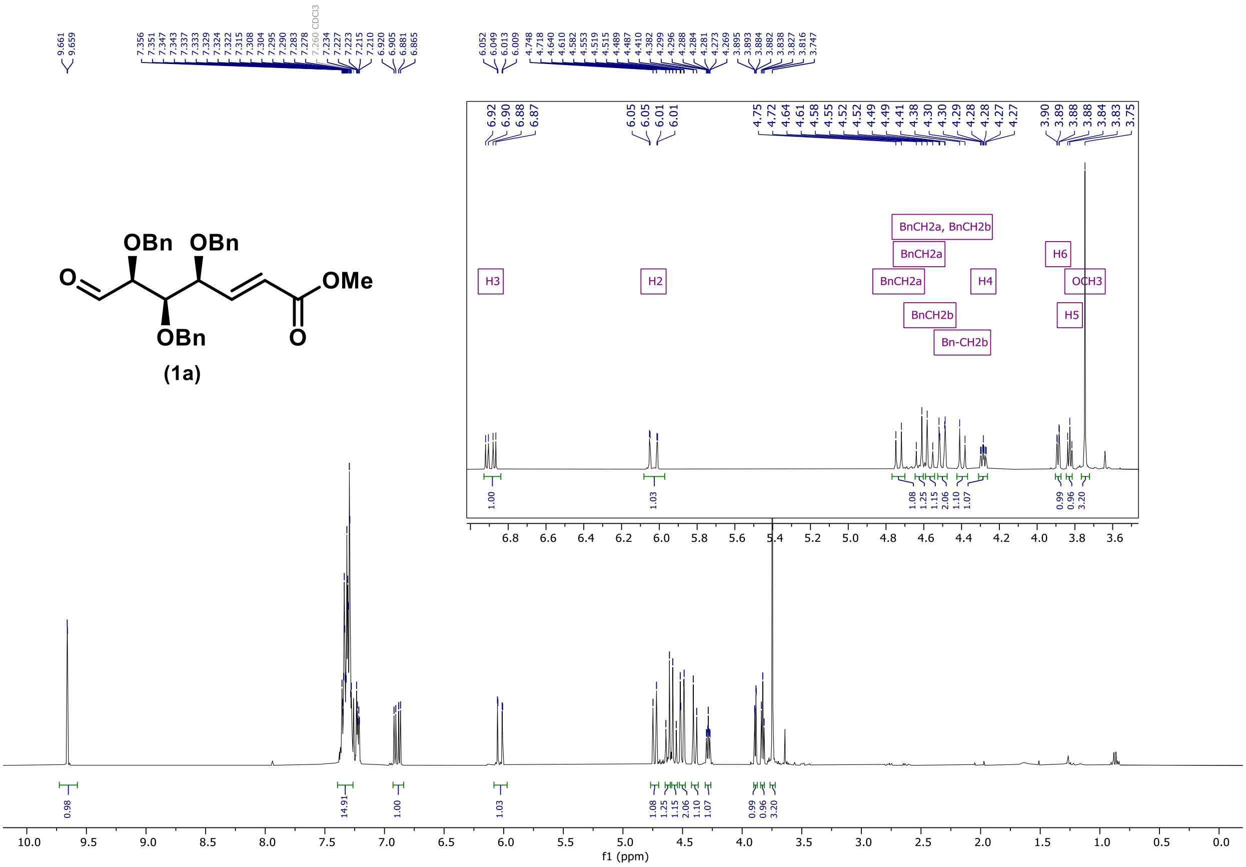

Figure S22. *400 MHz ^{1}H-NMR of (1a)*

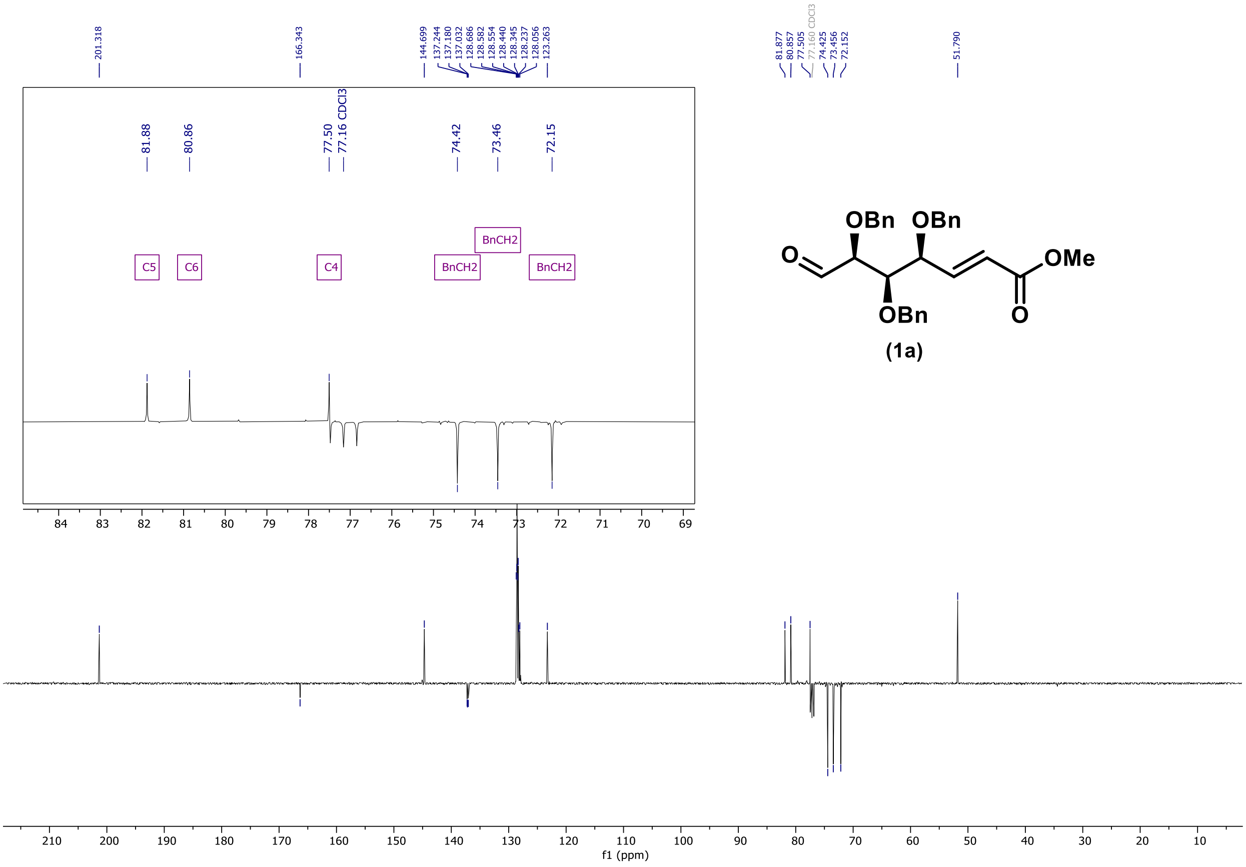

Figure S23. *101 MHz $^{13}C\{^{1}H\}$-NMR of (1a)*

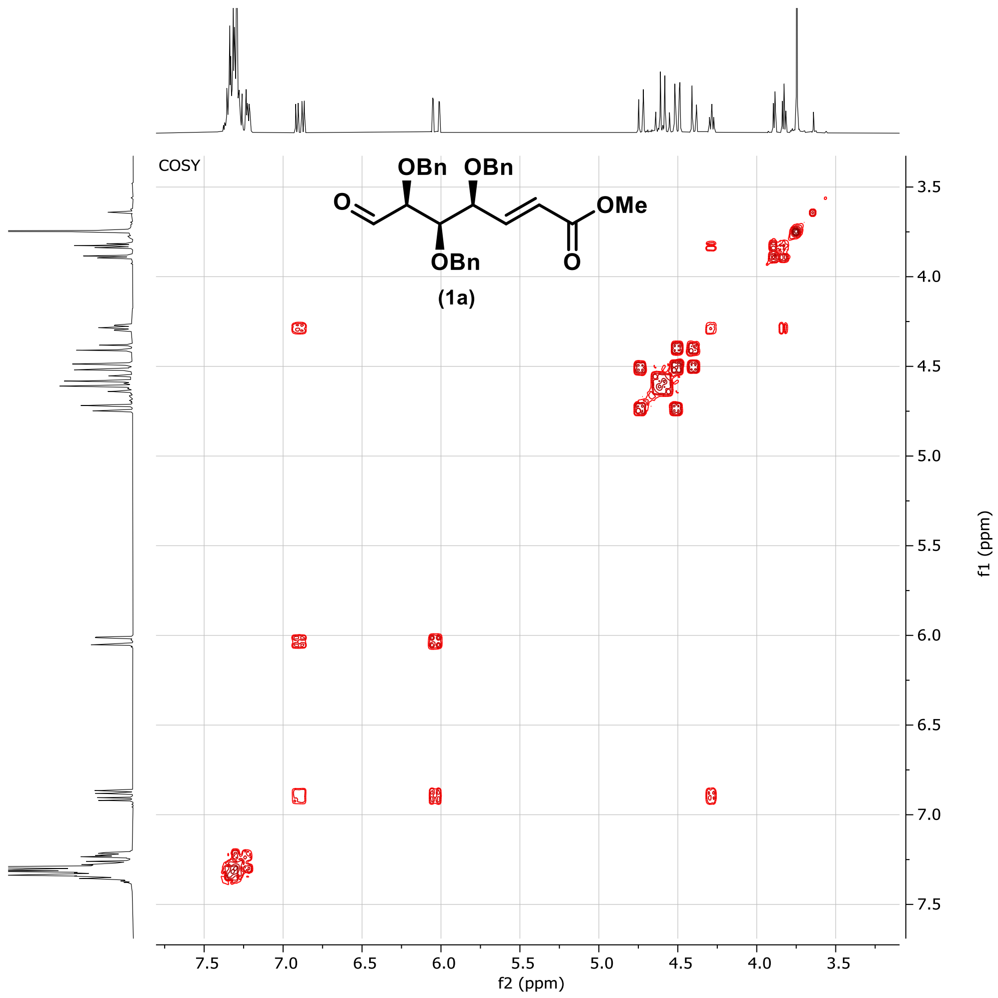

Figure S24. COSY NMR of (1a)

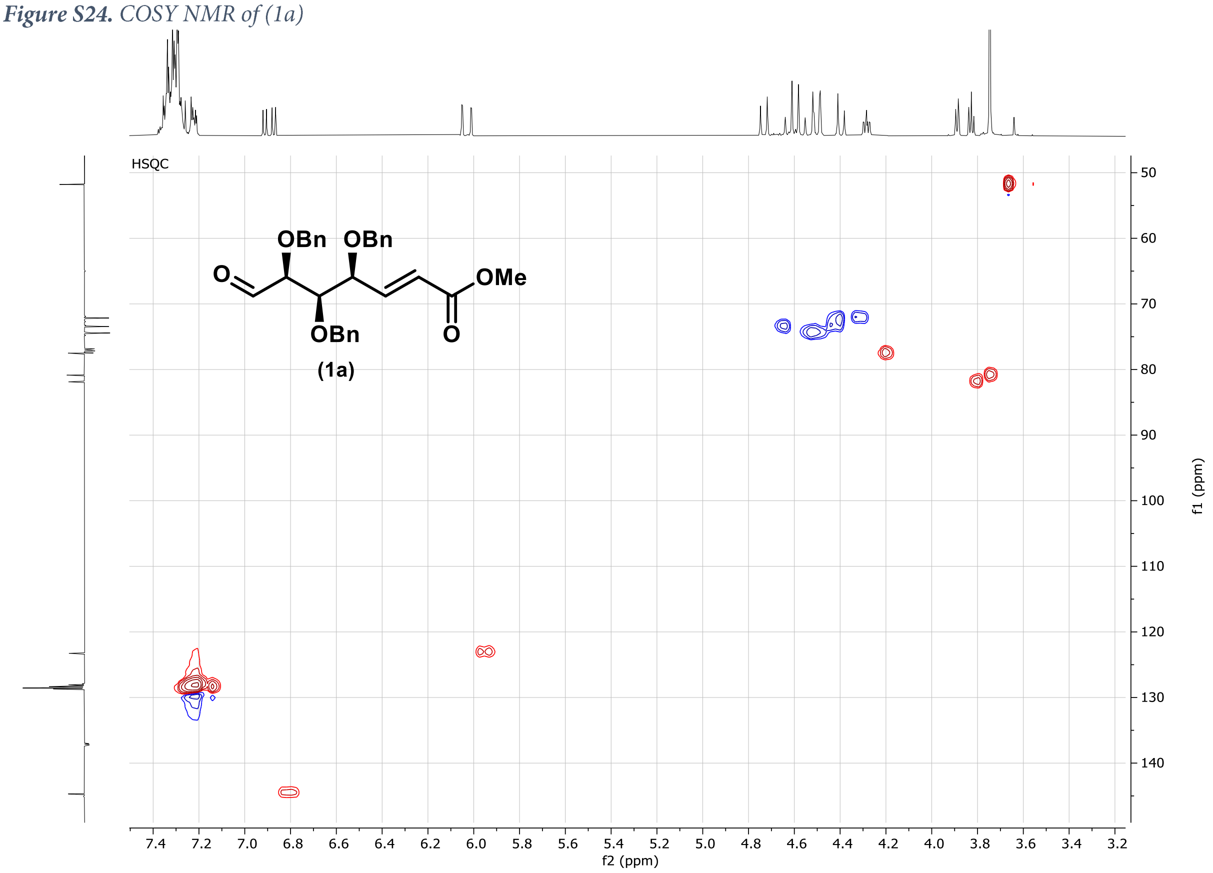

Figure S25. HSQC NMR of (1a)

D.1.6. Ethyl (4S,5R,6R)-4,5,6-tris(benzyloxy)-7-hydroxyhept-2-enoate (6f)

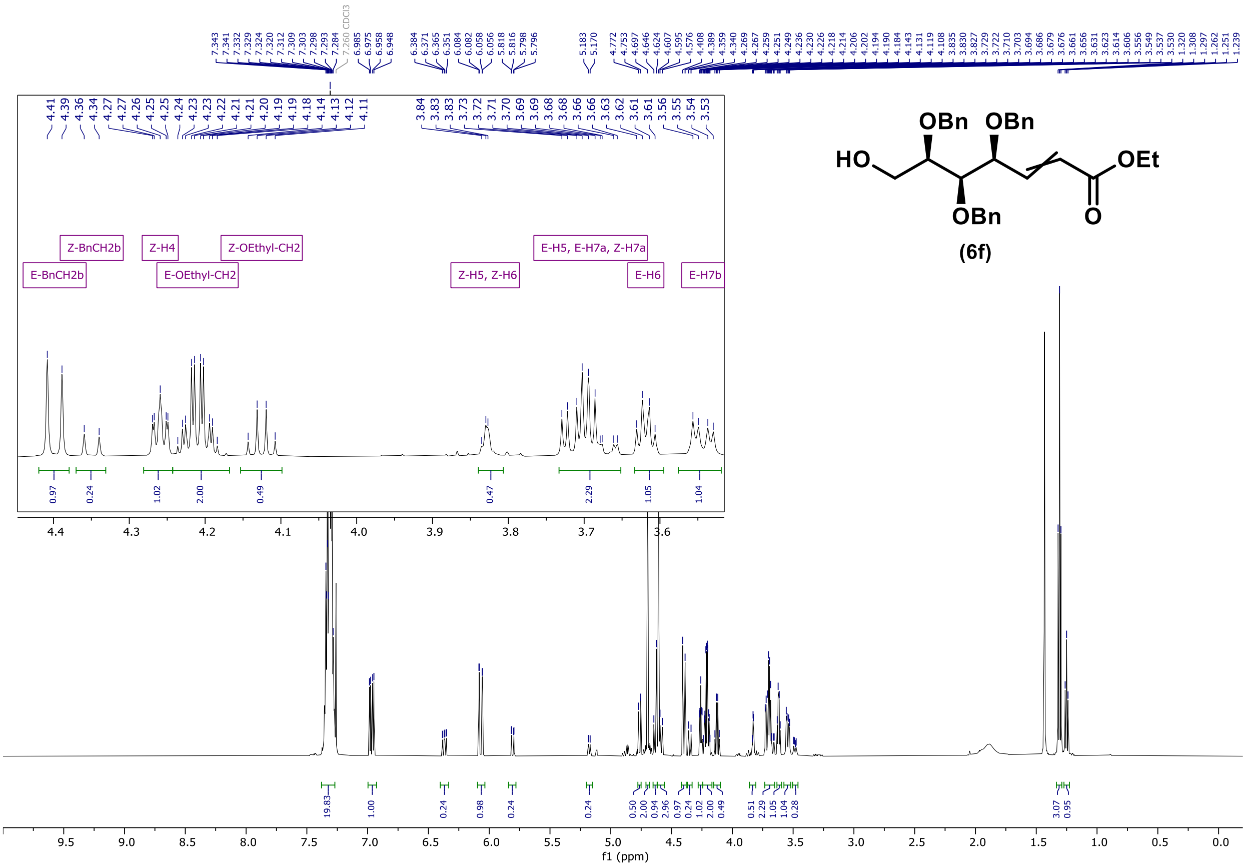

Figure S26. *600 MHz ^{1}H-NMR of (6f) as an E/Z Mixture*

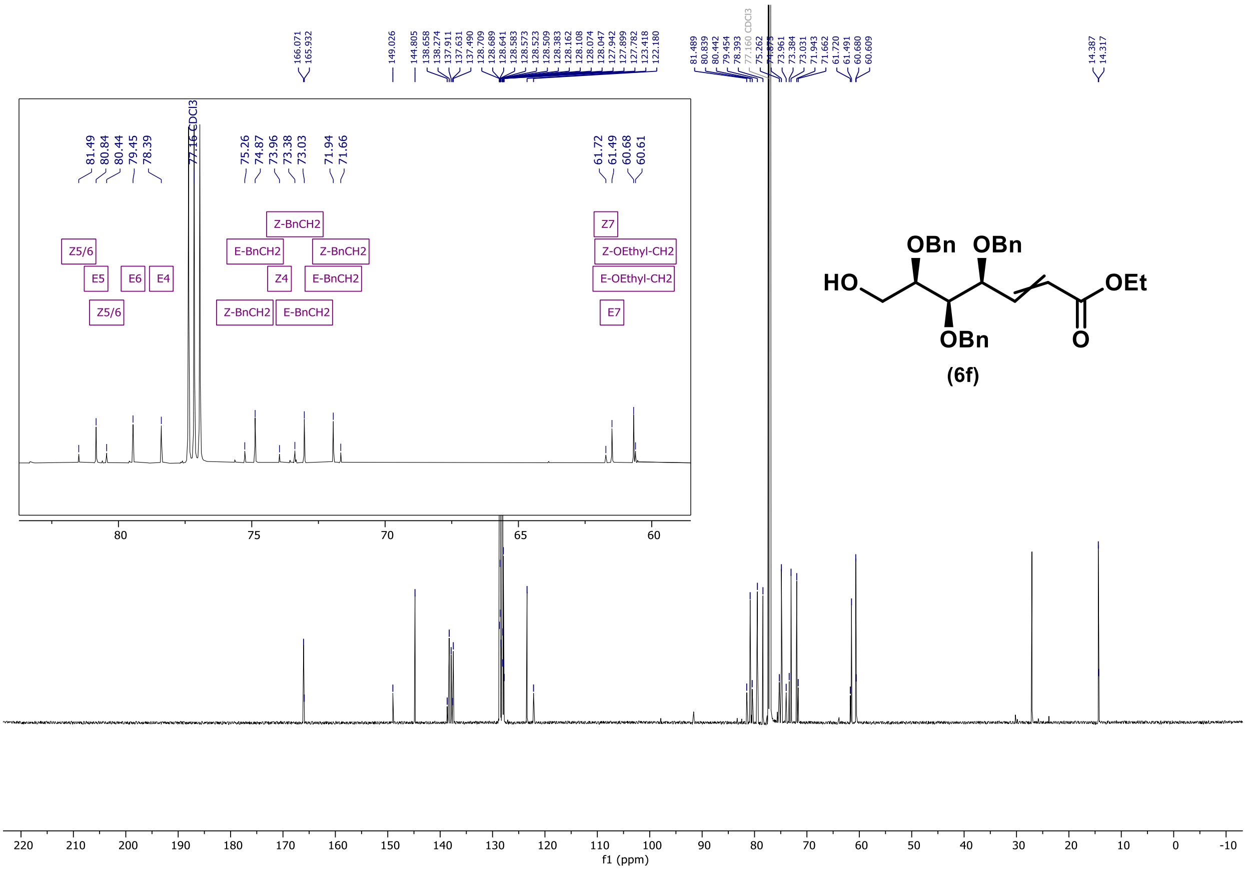

Figure S27. *151 MHz $^{13}C\{^{1}H\}$-NMR of (6f) as an E/Z Mixture*

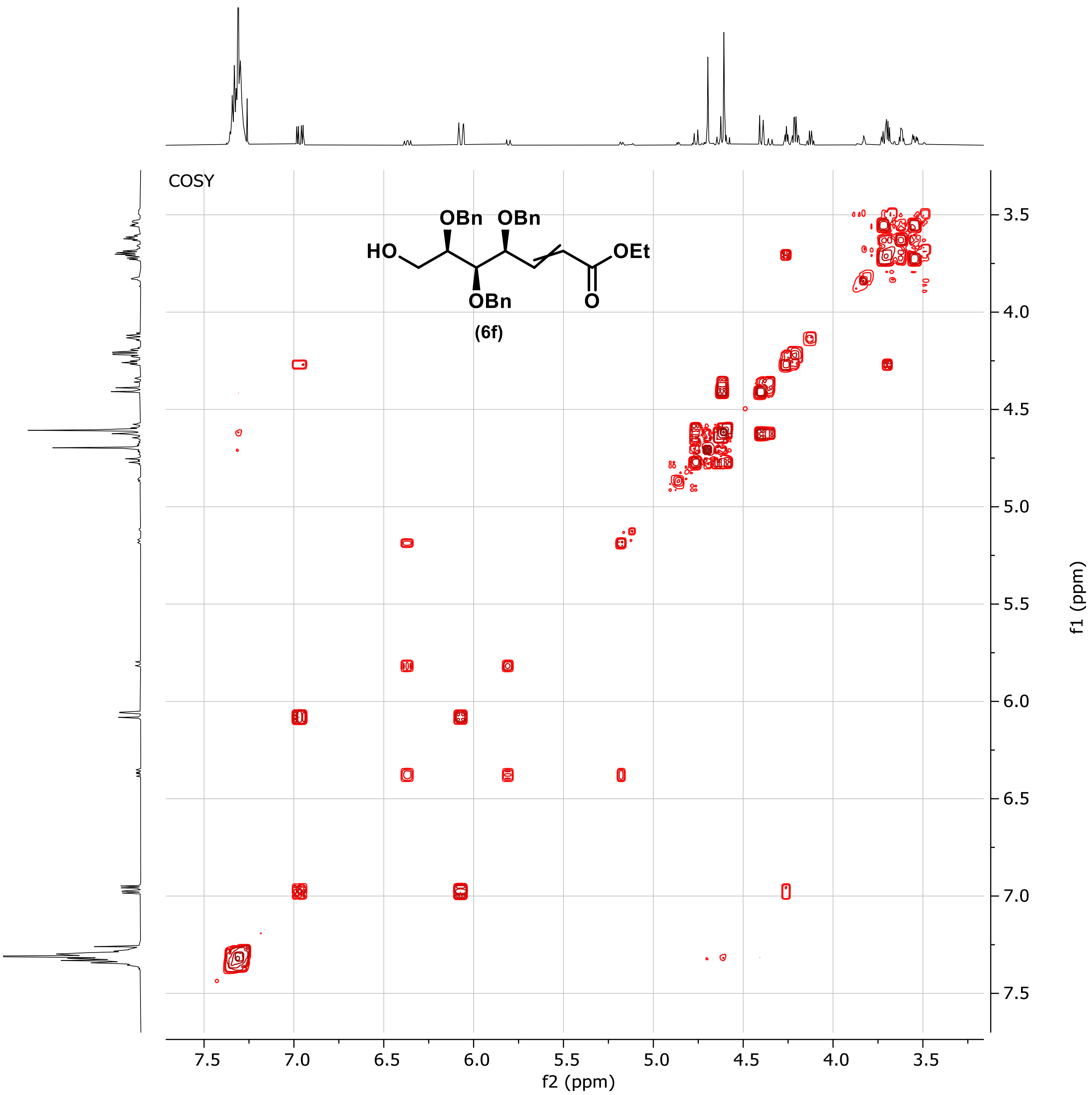

Figure S28. *COSY NMR of (6f) as an E/Z Mixture*

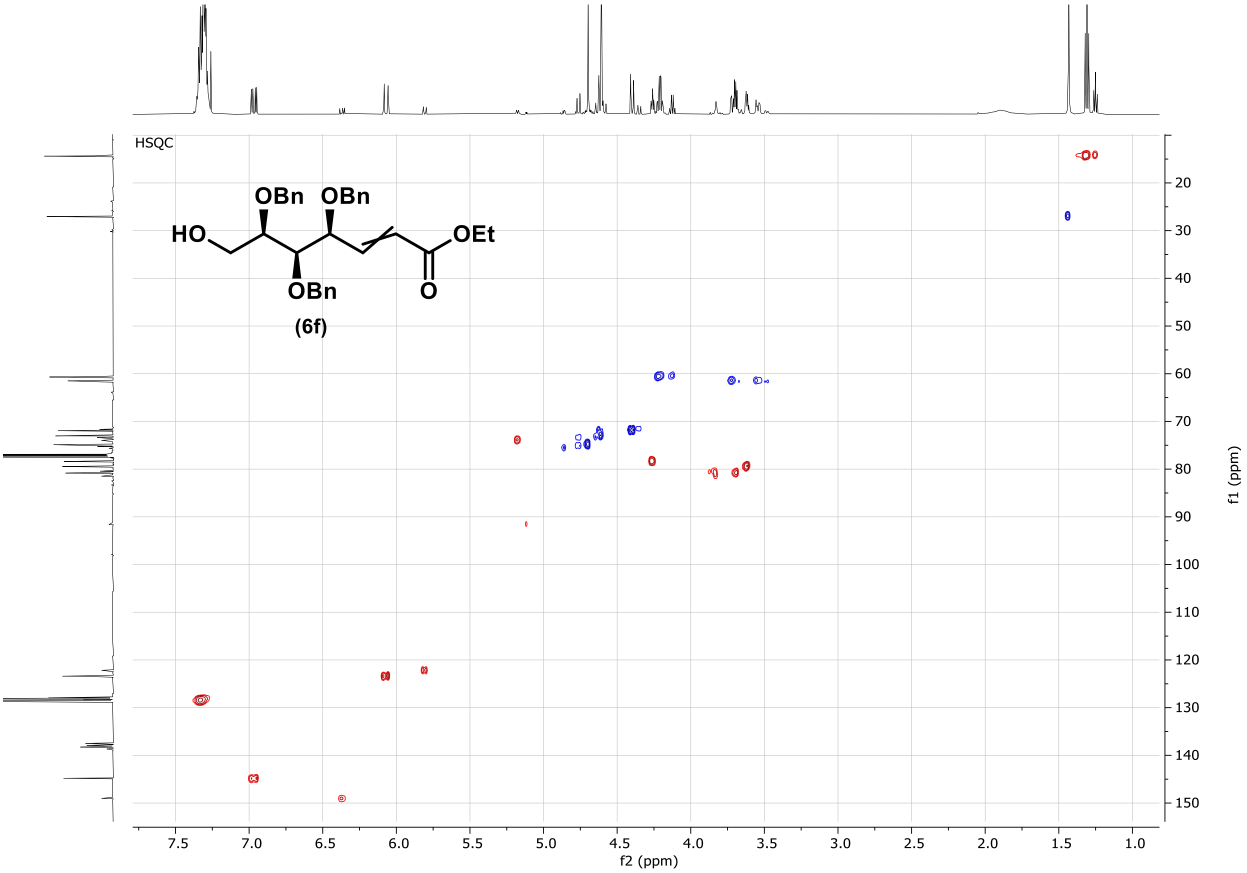

Figure S29. *HSQC NMR of (6f) as an E/Z Mixture*

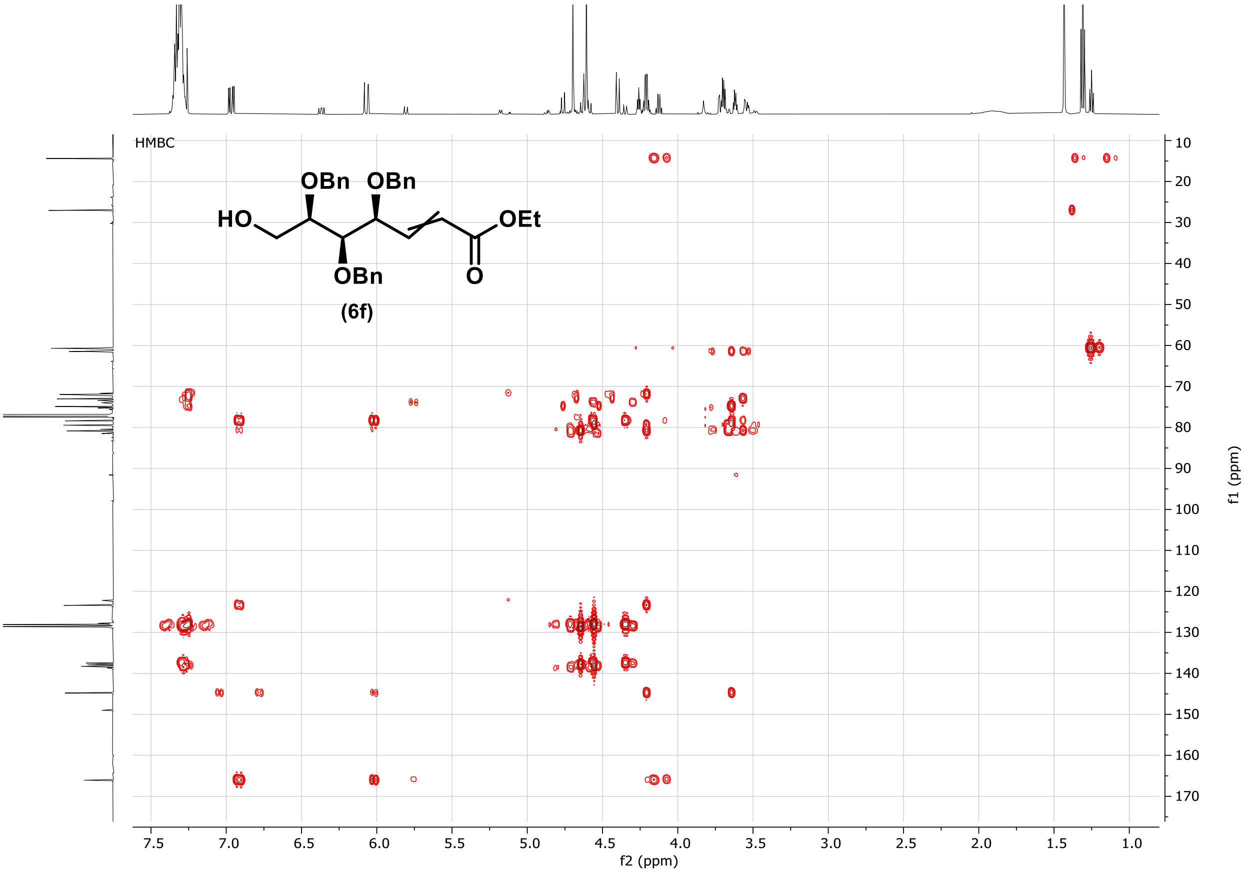

Figure S30. *HMBC NMR of (6f) as an E/Z Mixture*

D.1.7. Ethyl (4S, 5R, 6S, E)-4,5,6-tris(benzyloxy)-7-oxohept-2-enoate (1f-e)

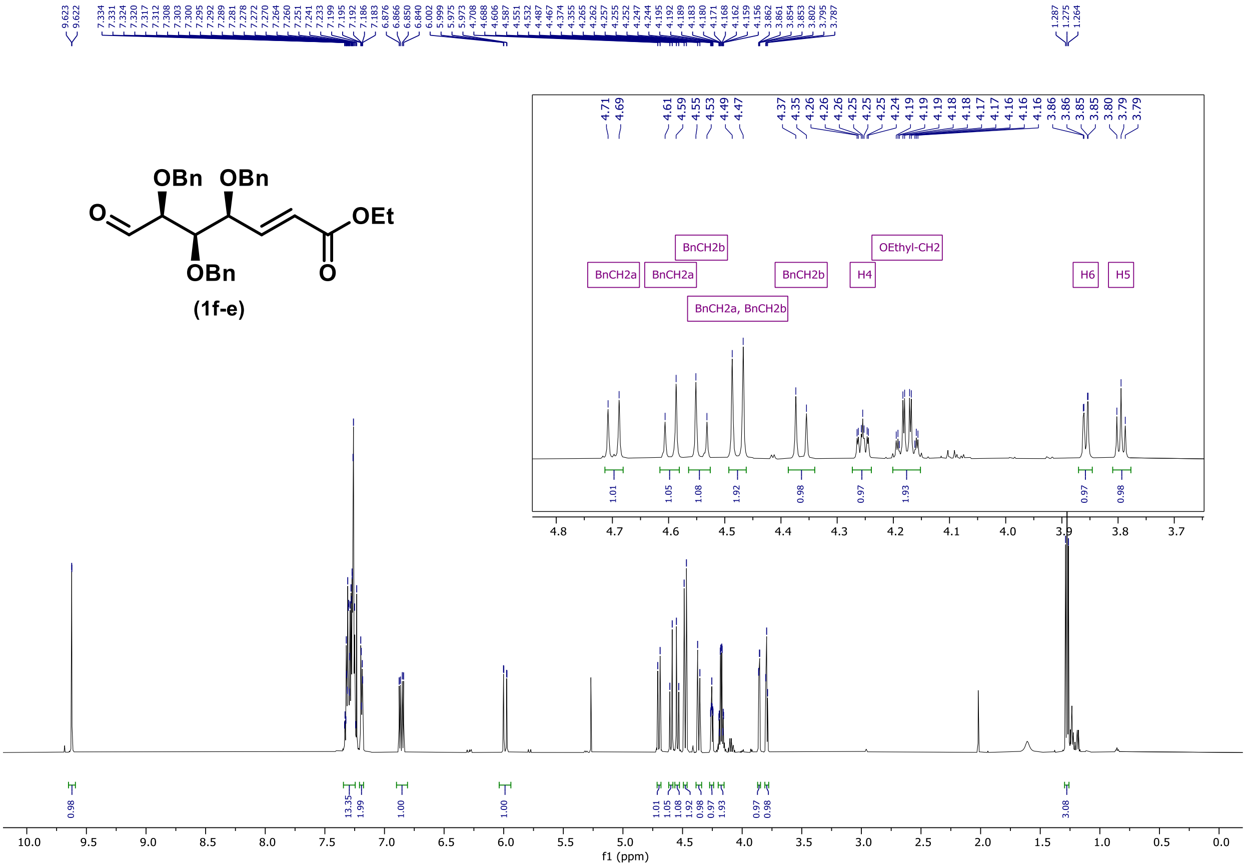

Figure S31. *600 MHz ^{1}H-NMR of (1f-e)*

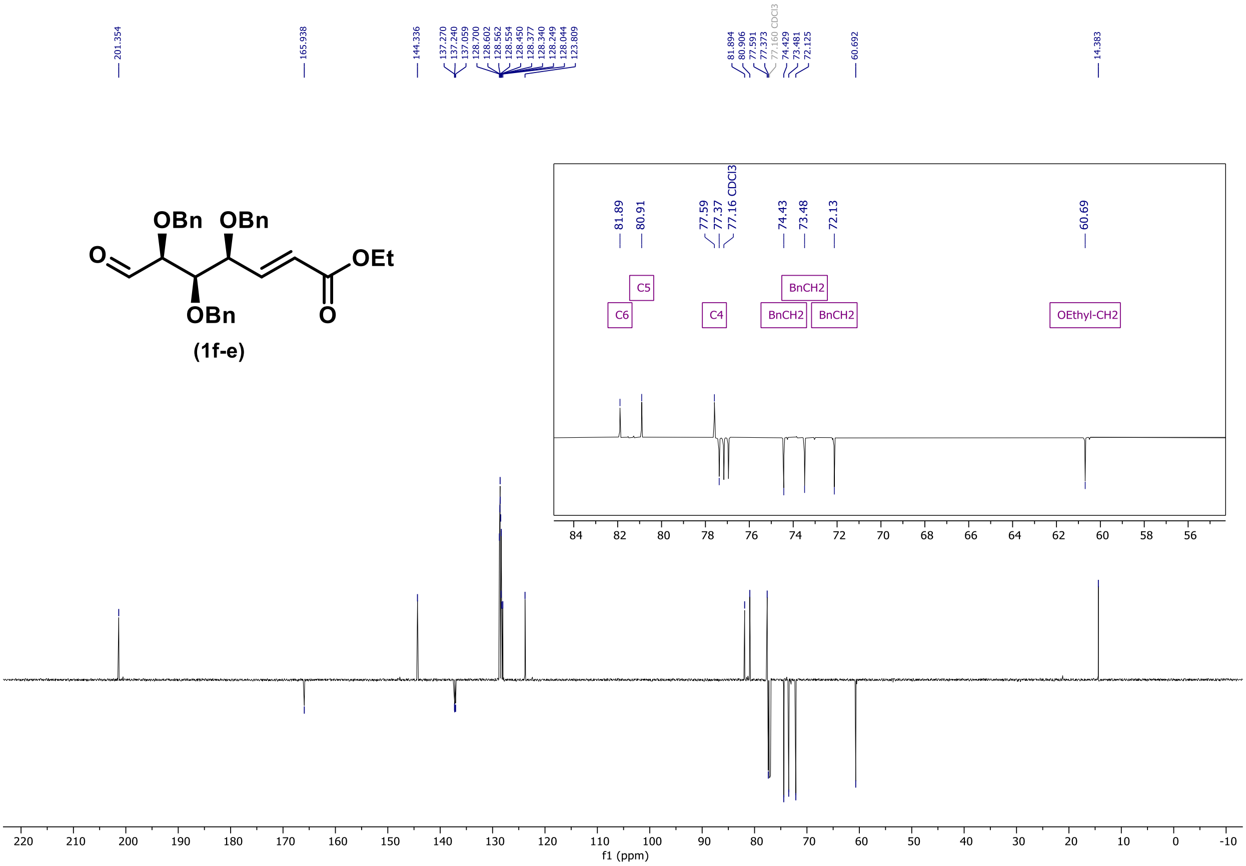

Figure S32. *151 MHz ^{13}C{^{1}H}-NMR of (1f-e)*

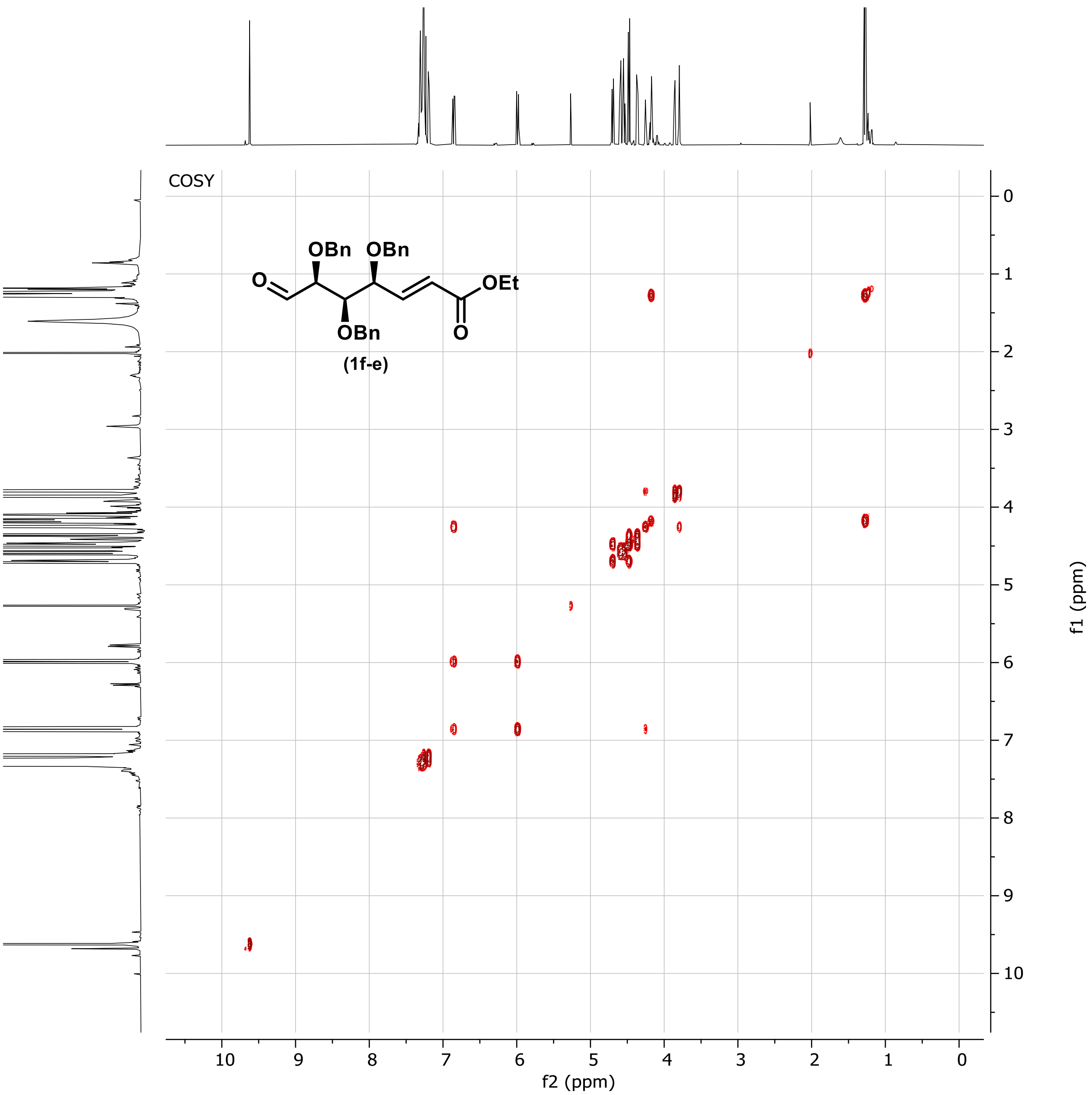

__Figure S33.__ COSY NMR of (1f-e)

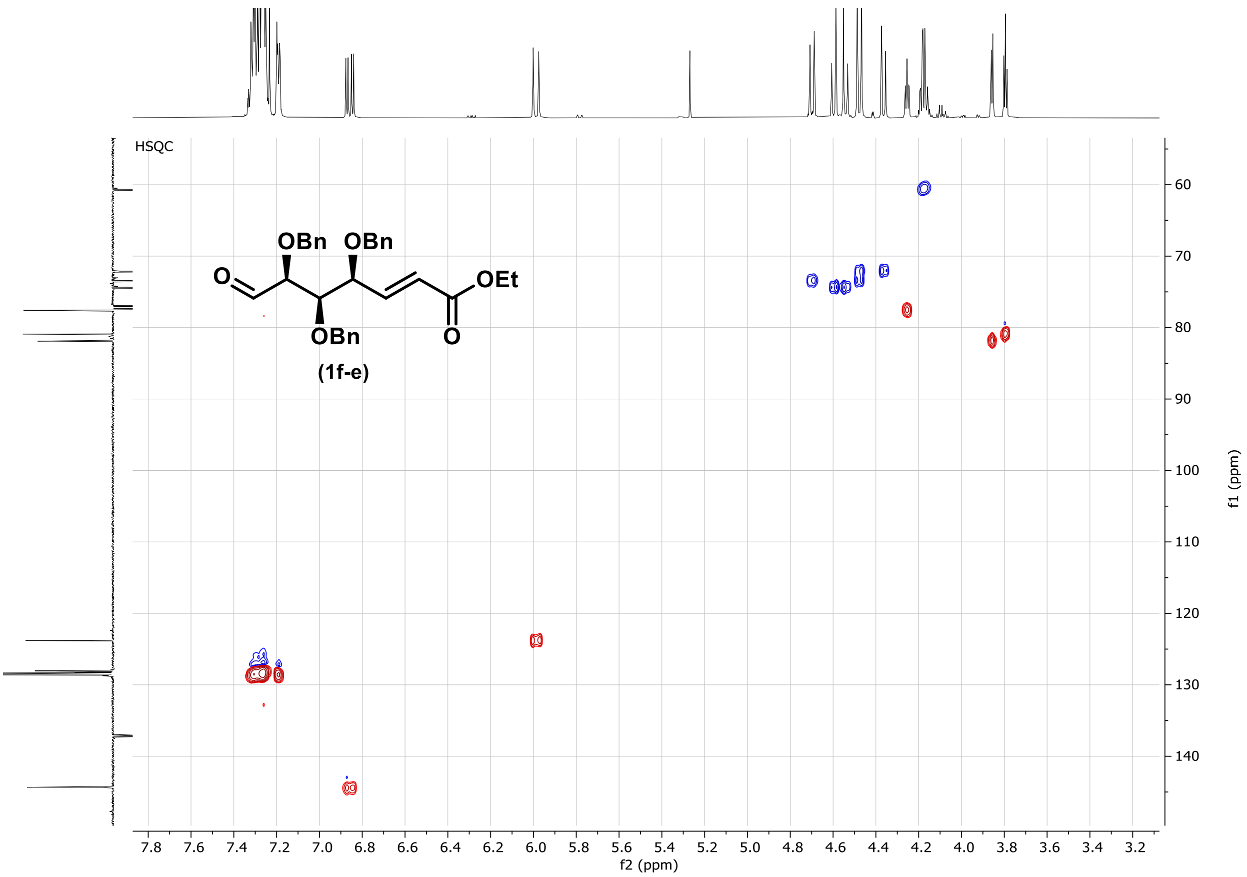

Figure S34. HSQC NMR of (1f-e)

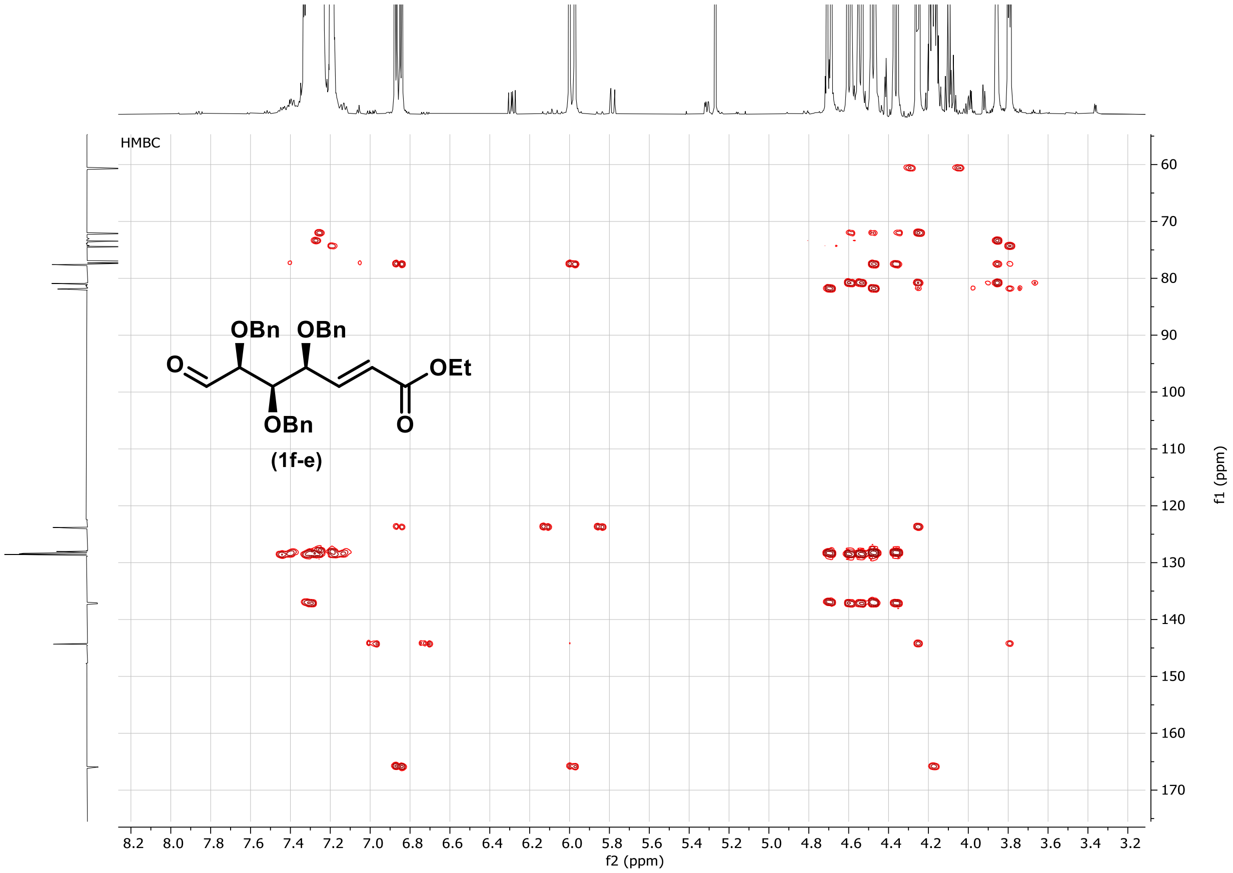

Figure S35. HMBC NMR of (1f-e)

D.1.8. Ethyl (4S, 5R, 6S, Z)-4,5,6-tris(benzyloxy)-7-oxohept-2-enoate (1f-z)

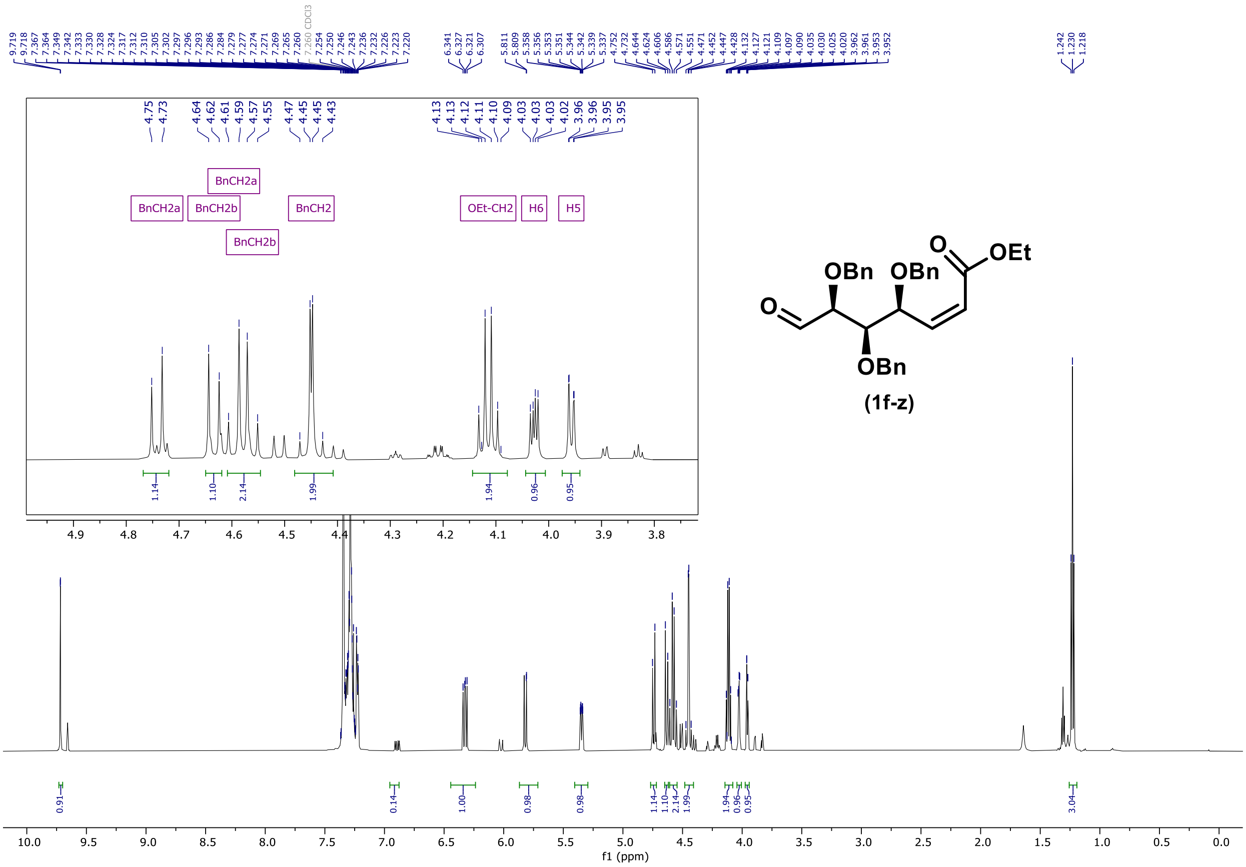

Figure S36. *600 MHz ^{1}H-NMR of (1f-z) in a E/Z mixture of 15:85*

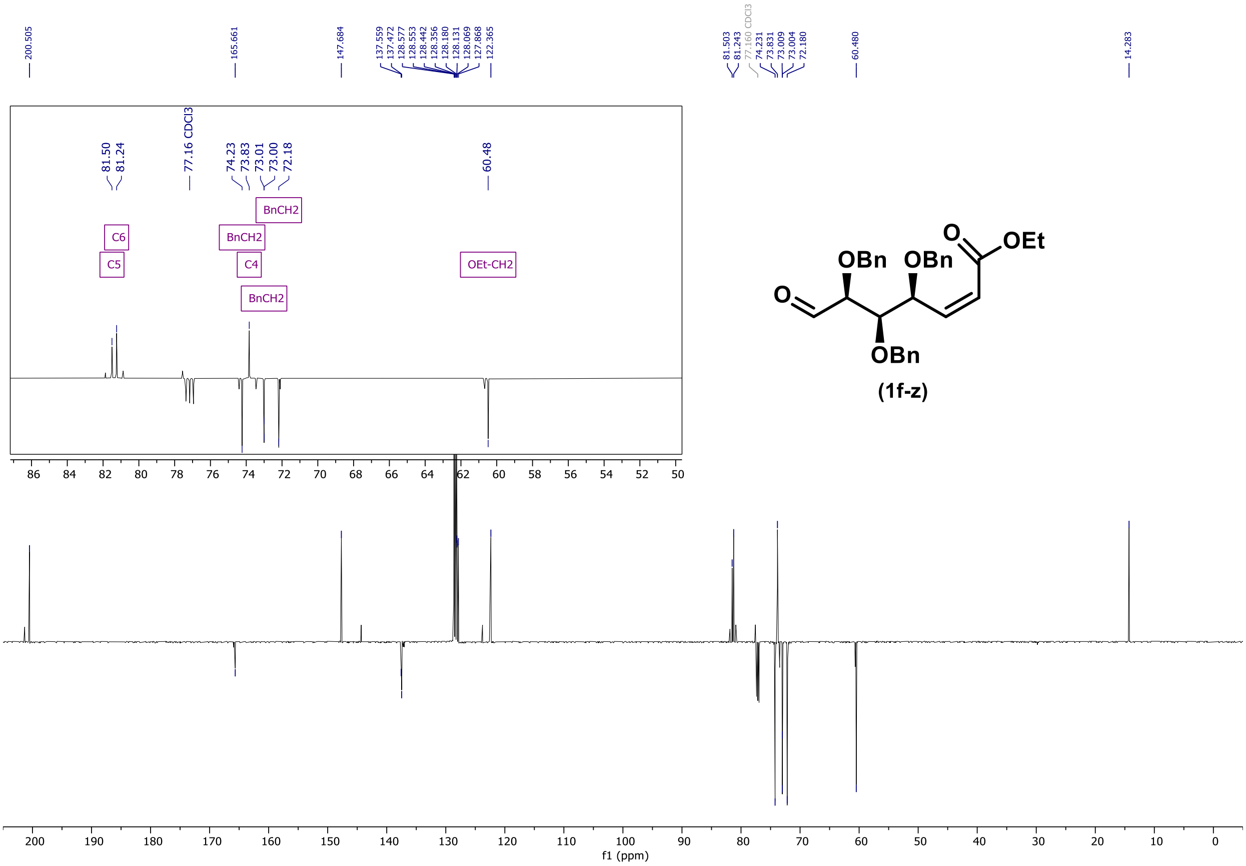

Figure S37. *151 MHz ^{13}C{^{1}H}-NMR of (1f-z) in a E/Z mixture of 15:85*

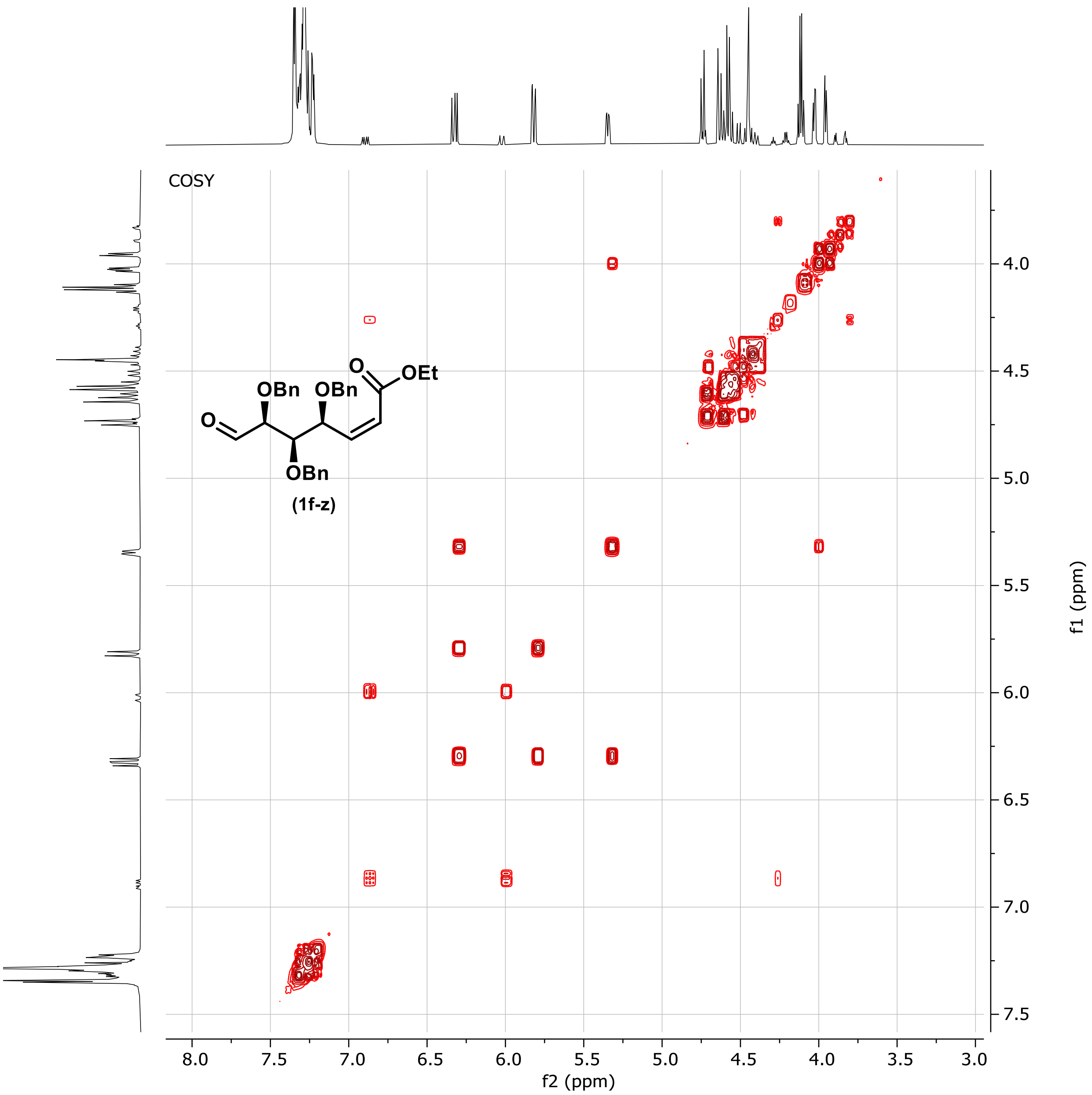

Figure S38. COSY NMR of (1f-z)

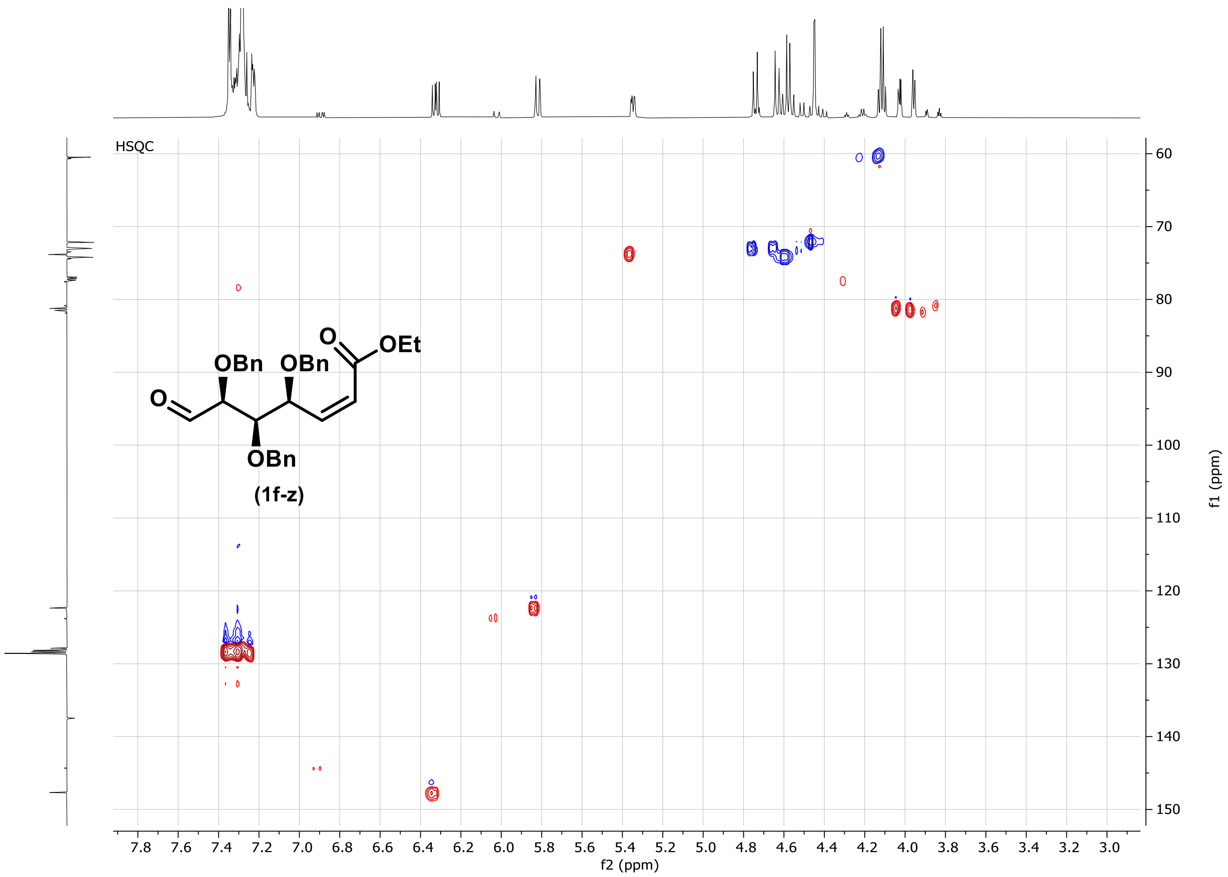

Figure S39. HSQC NMR of (1f-z)

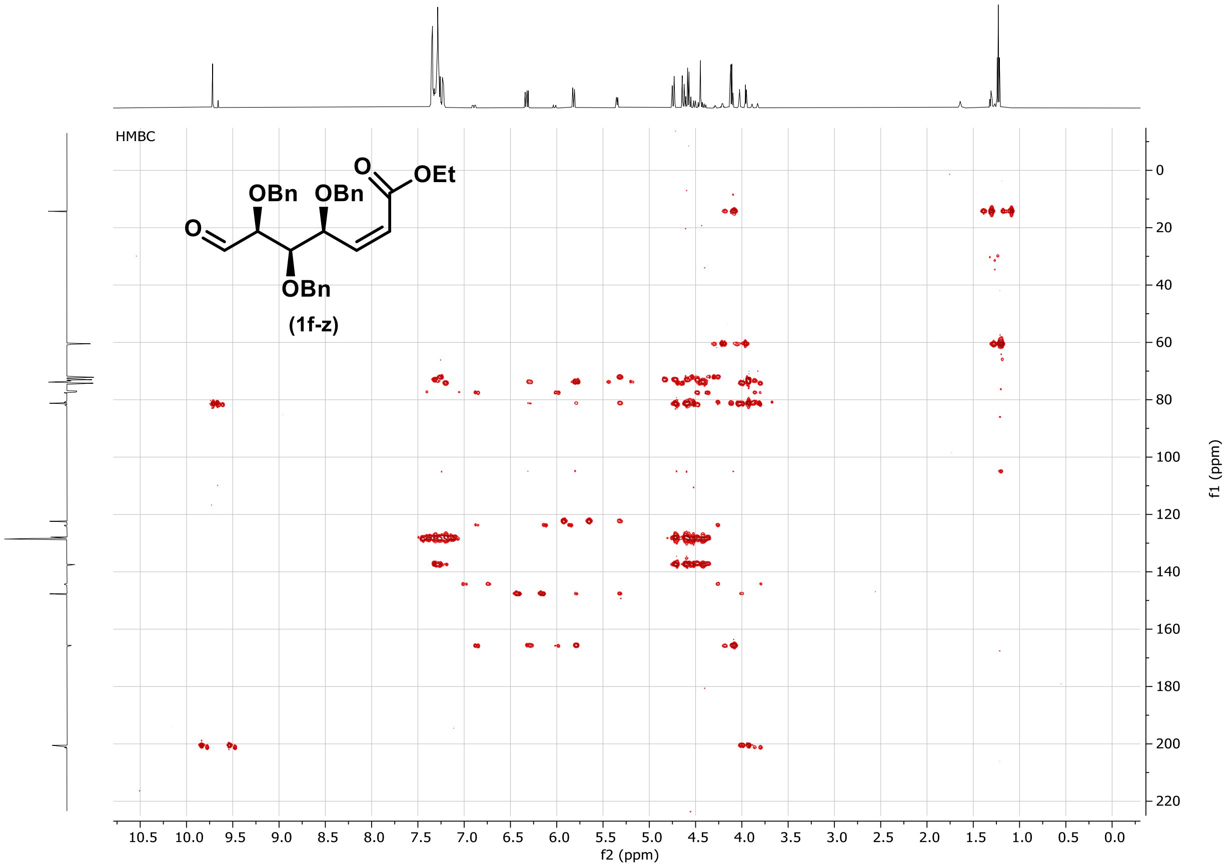

Figure S40. HMBC NMR of (1f-z)

D.1.9. Benzyl (4S,5R,6R,E)-4,5,6-tris(benzyloxy)-7-hydroxyhept-2-enoate (6g-e)

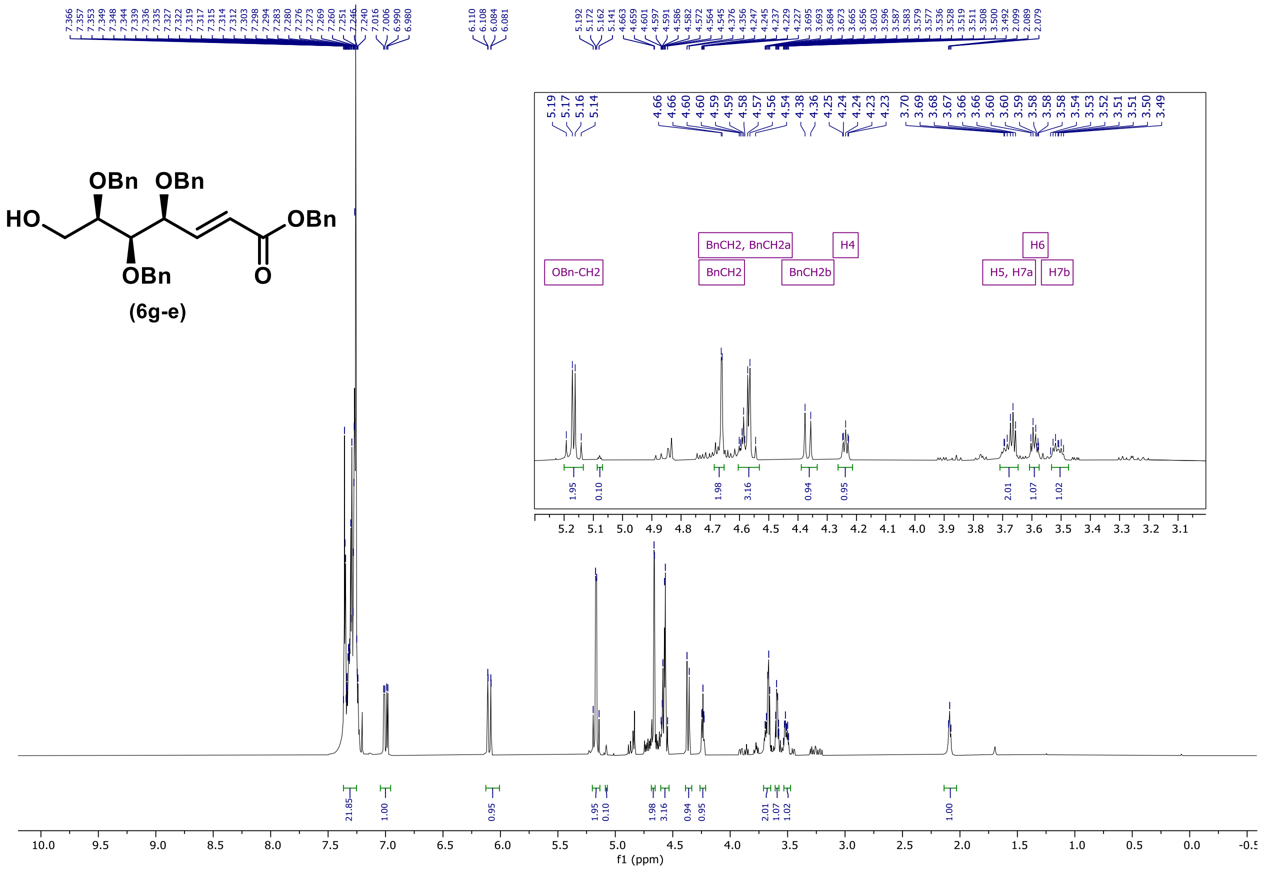

Figure S41. *600 MHz ^{1}H-NMR of (6g-e) as 9:1 mixture with (5a)*

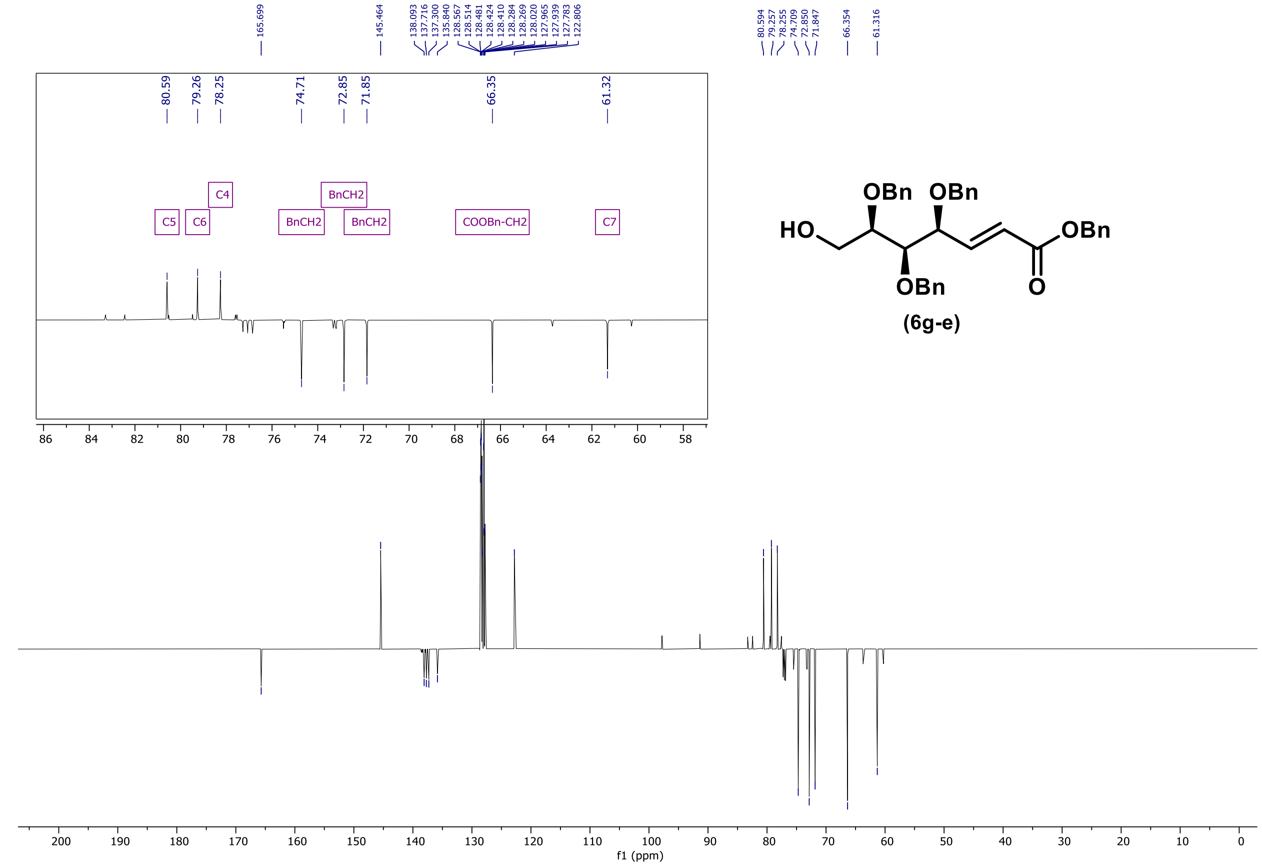

Figure S42. *151 MHz ^{13}C{^{1}H}-NMR of (6g-e) (5a)*

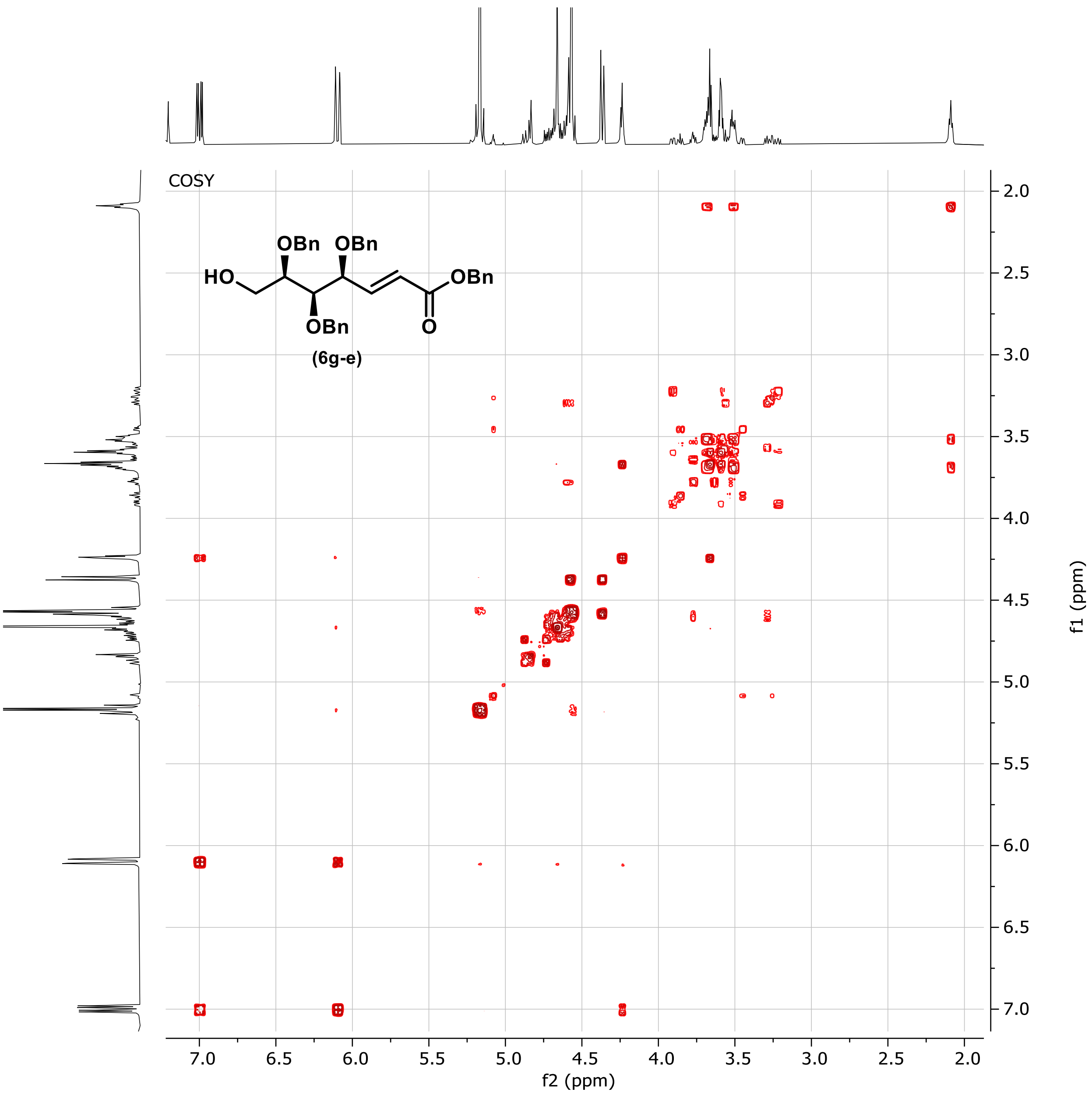

Figure S43. COSY NMR of (6g-e)

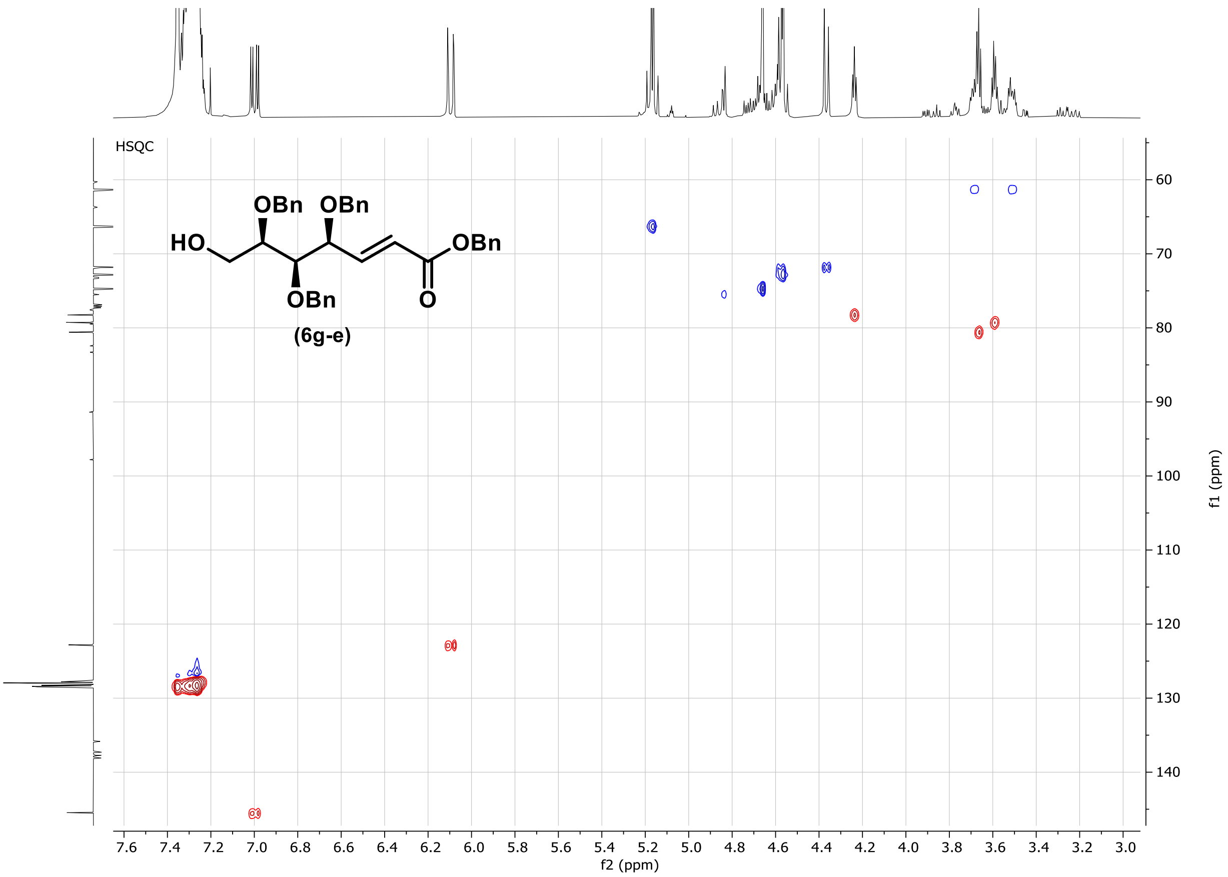

Figure S44. HSQC NMR of (6g-e)

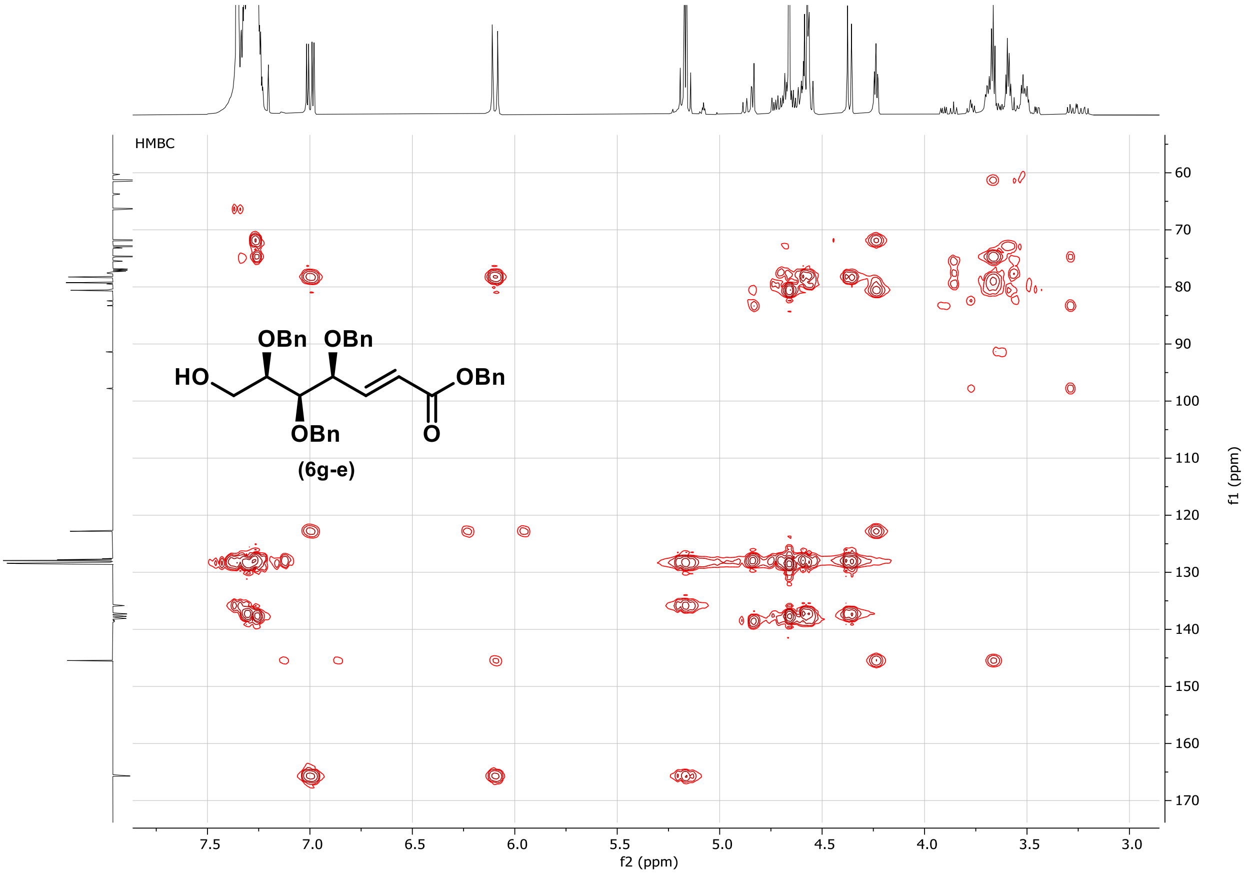

Figure S45. HMBC NMR of (6g-e)

D.1.10. Benzyl (4S,5R,6R,Z)-4,5,6-tris(benzyloxy)-7-hydroxyhept-2-enoate (6g-z)

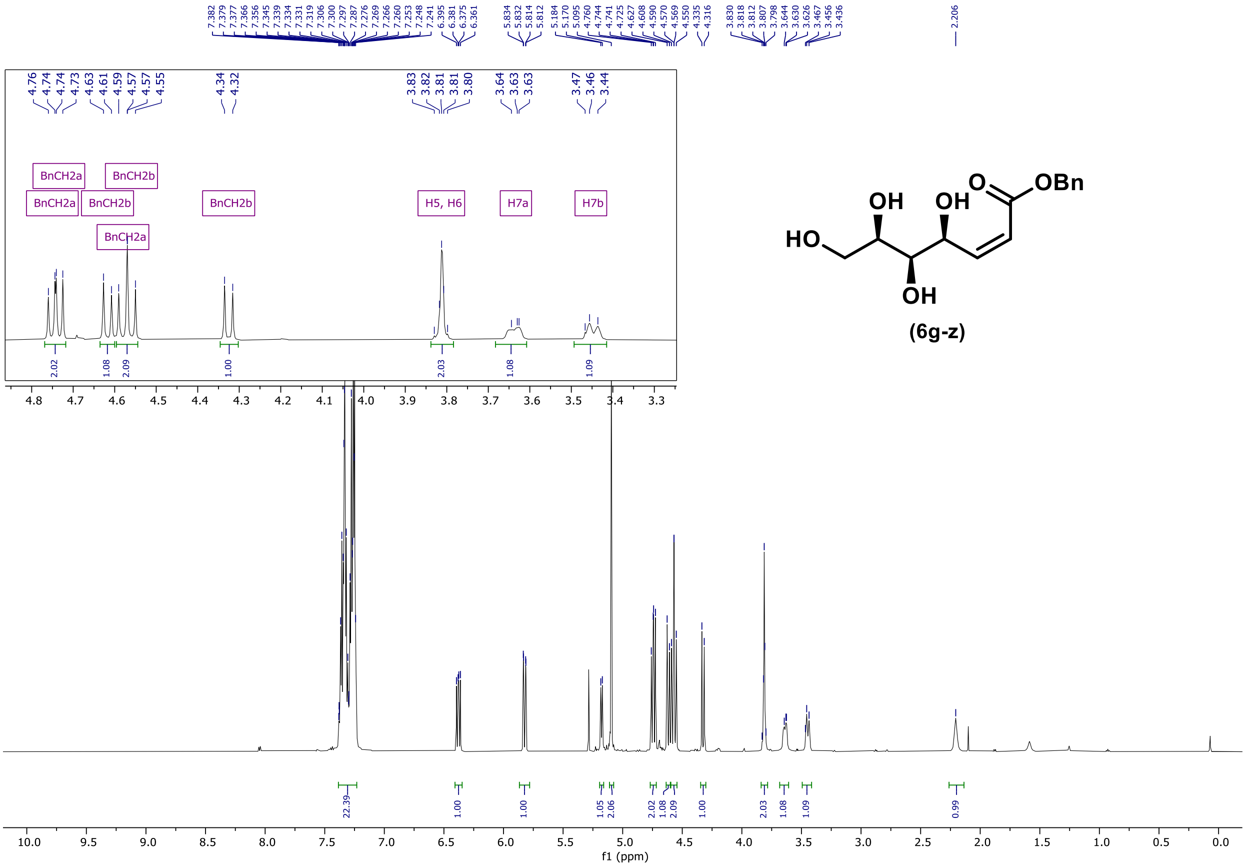

Figure S46. *600 MHz* ^{1}*H-NMR of (6g-z)*

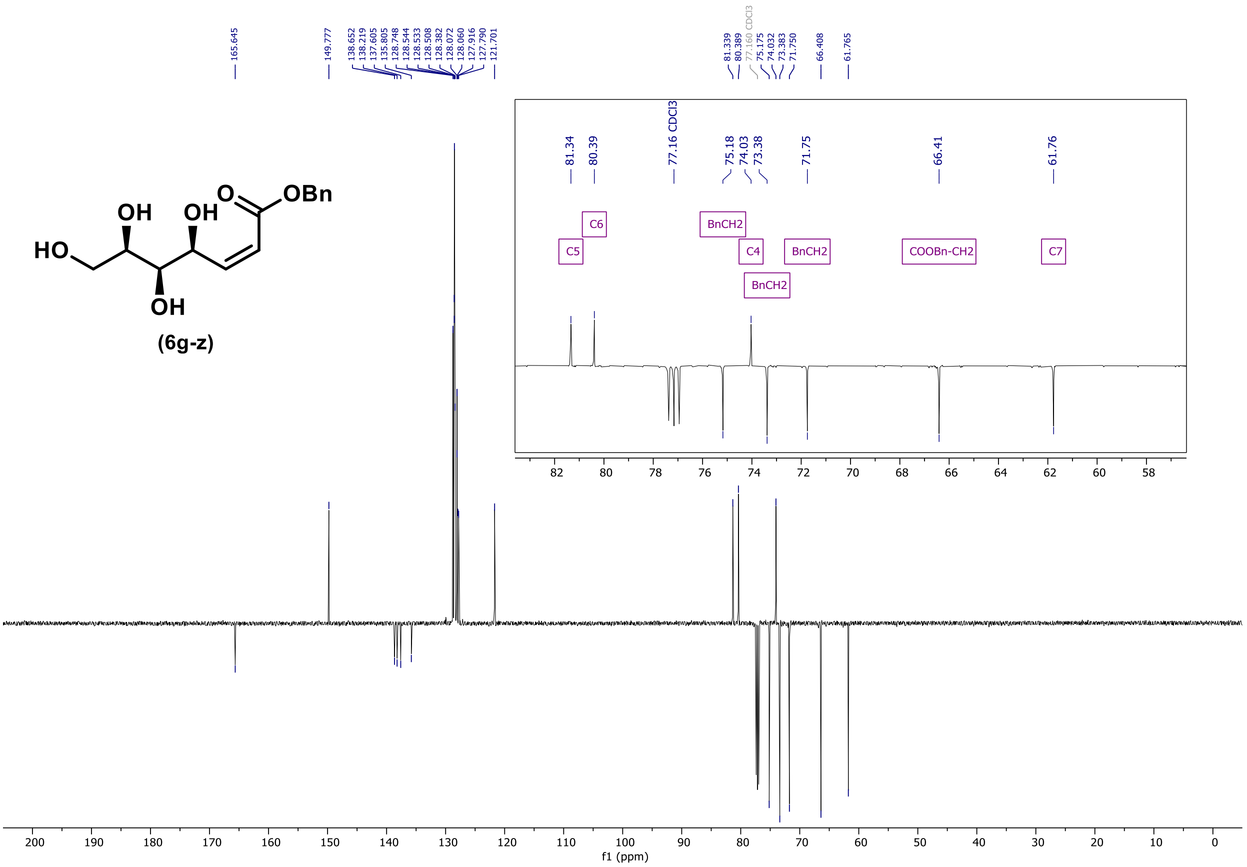

Figure S47. *151 MHz* $^{13}C\{^{1}H\}$*-NMR of (6g-z)*

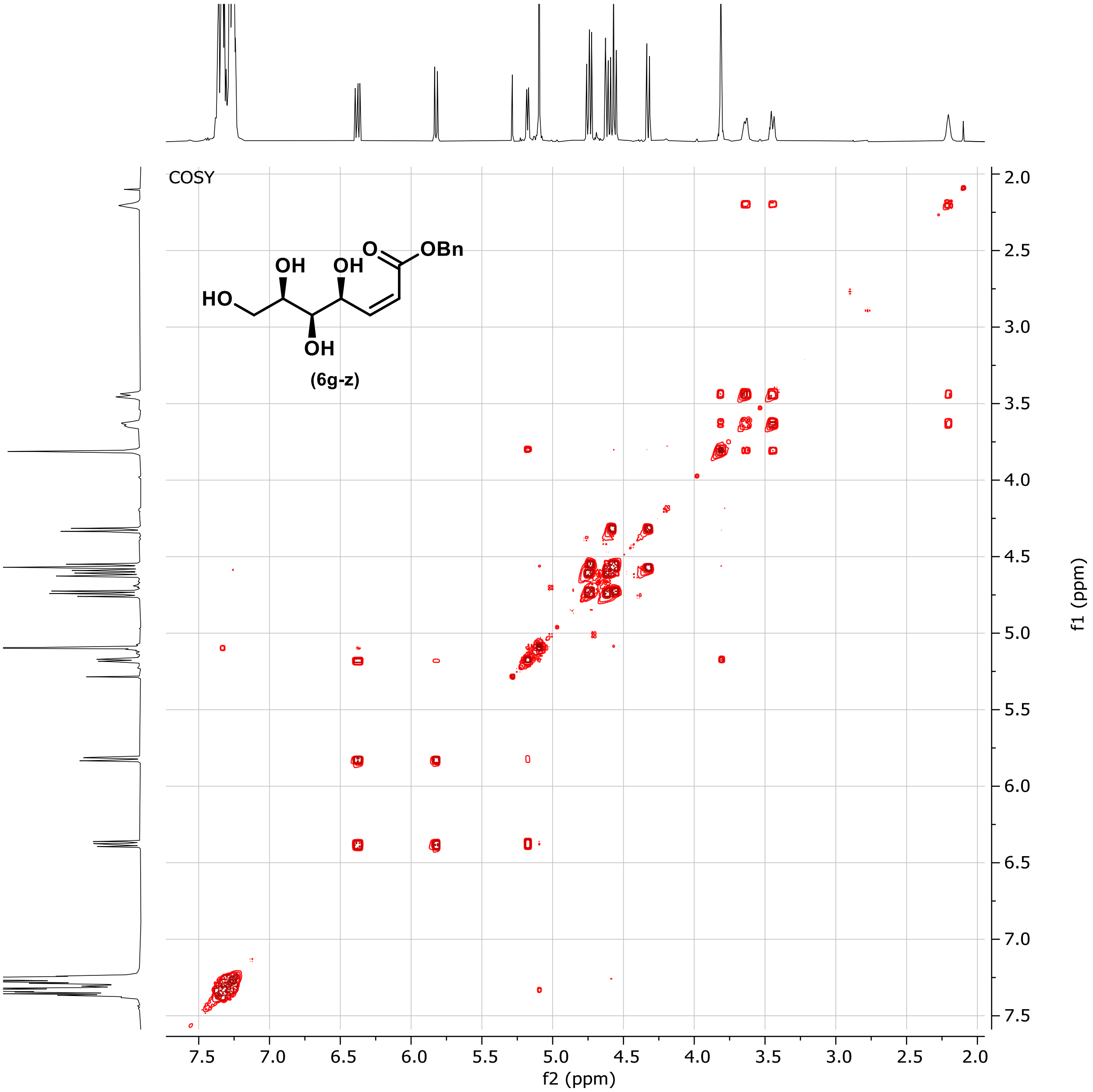

Figure S48. COSY NMR of (6g-z)

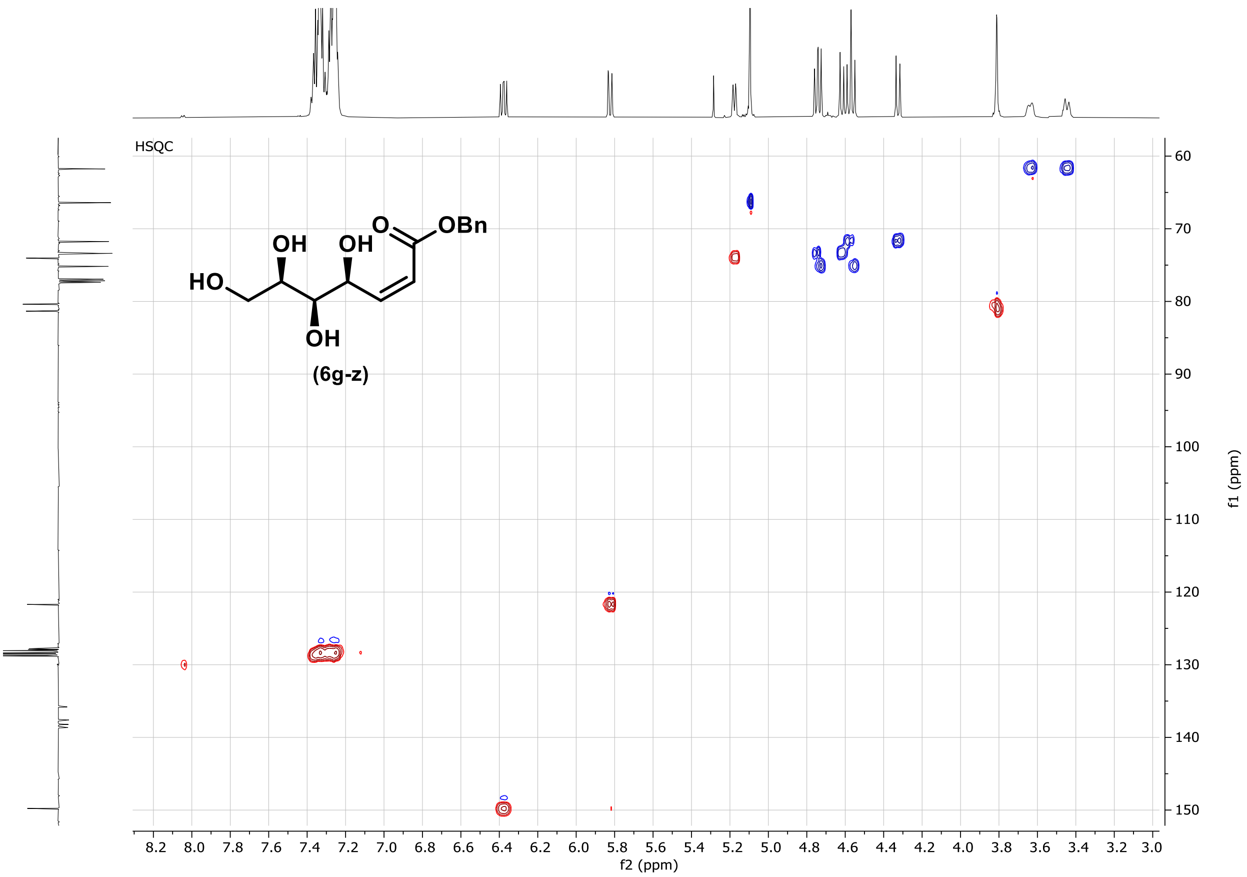

Figure S49. HSQC NMR of (6g-z)

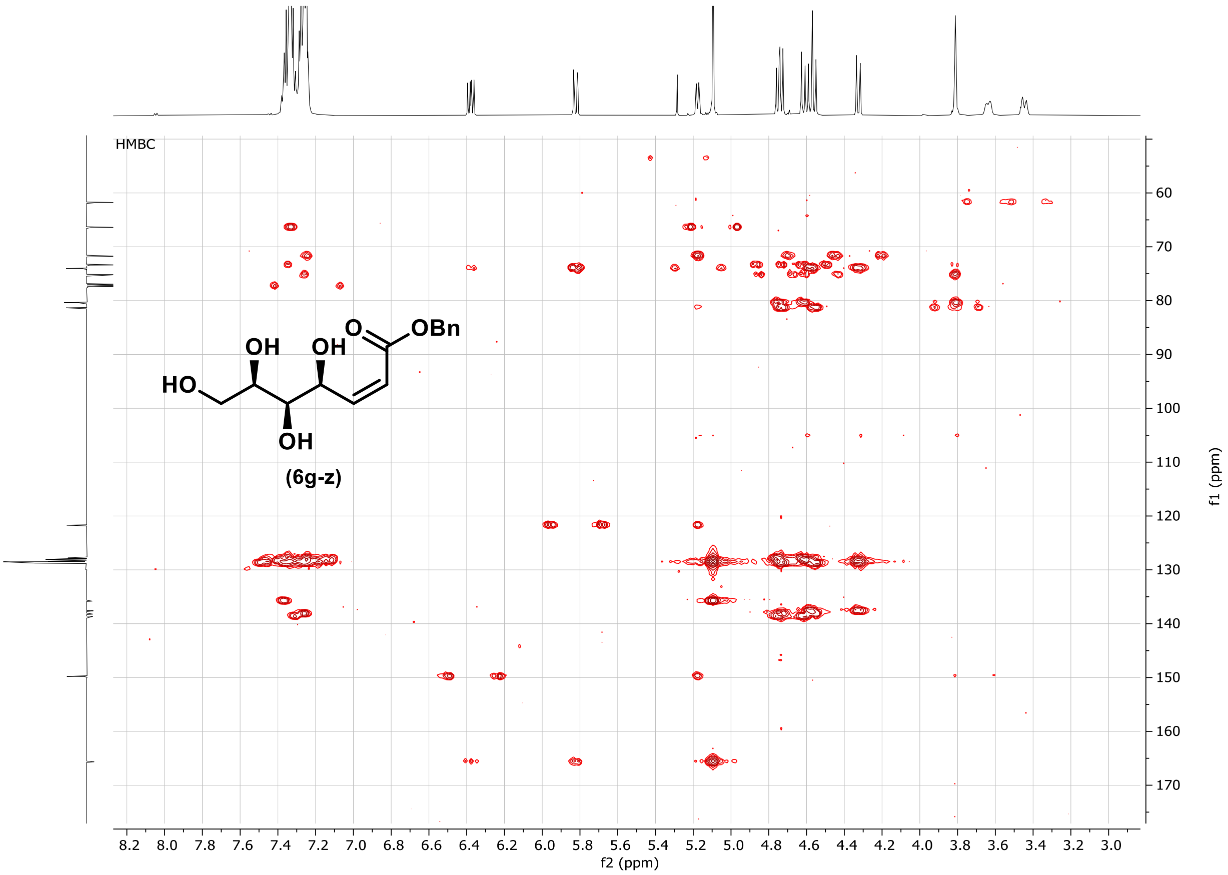

Figure S50. HMBC NMR of (6g-z)

D.1.11. Benzyl (4S, 5R, 6S, E)-4,5,6-tris(benzyloxy)-7-oxo-hept-2-enoate (1g)

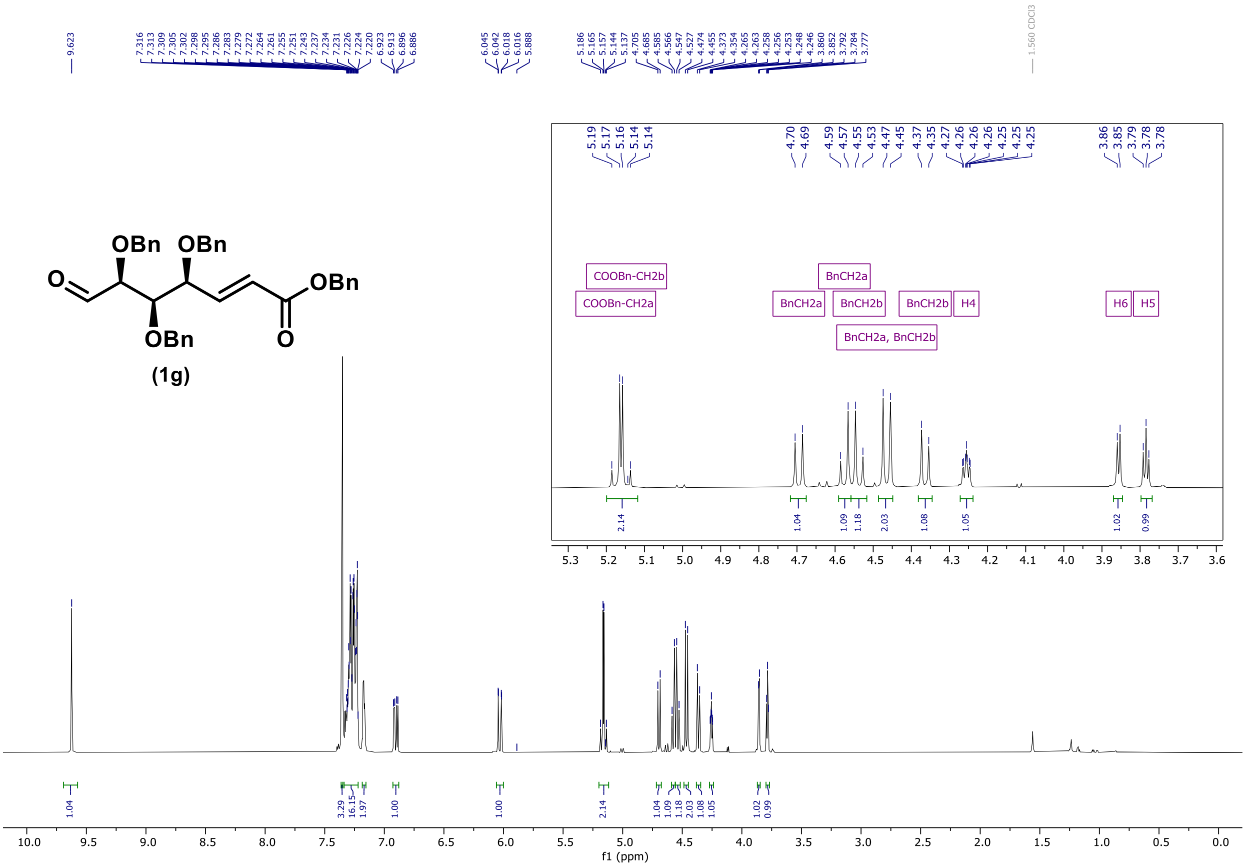

Figure S51. 600 MHz ^{1}H-NMR of (1g)

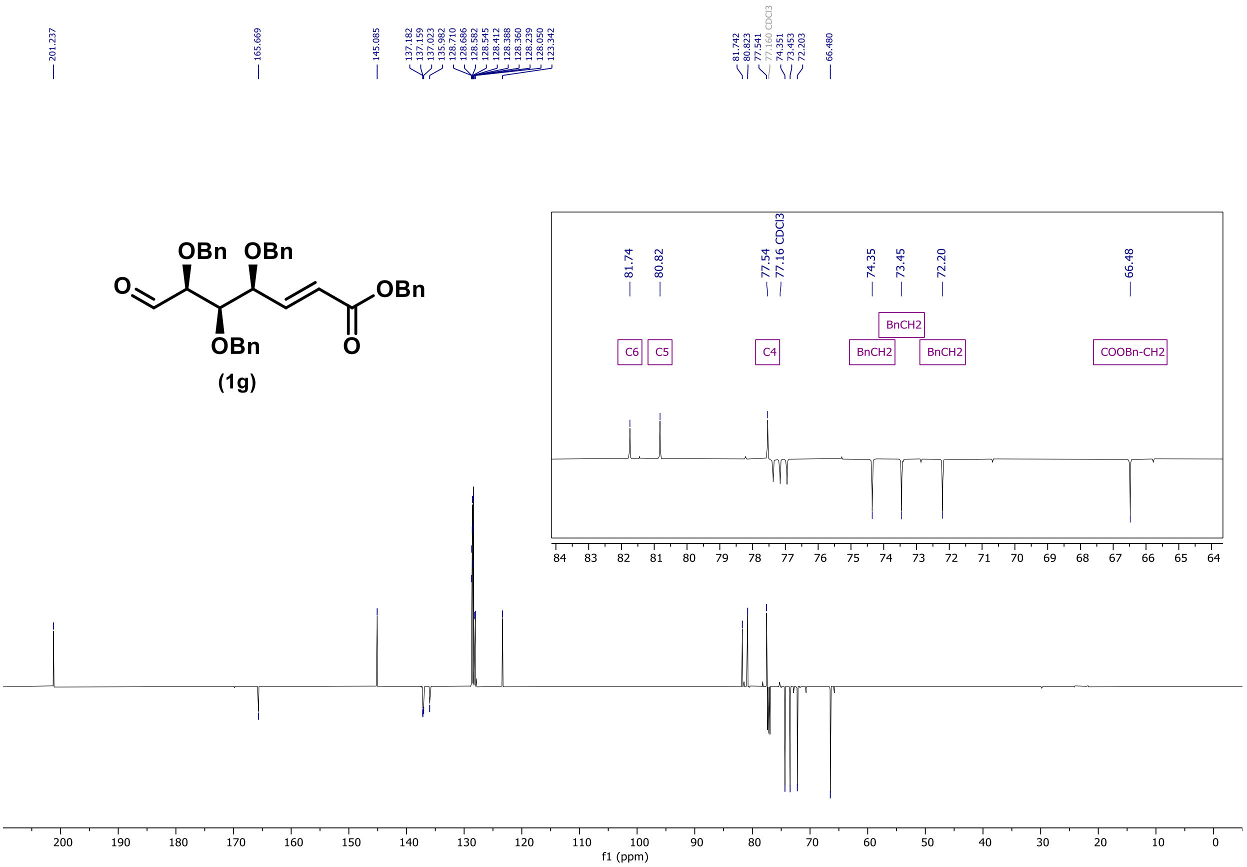

Figure S52. 151 MHz ^{13}C{^{1}H}-NMR of (1g)

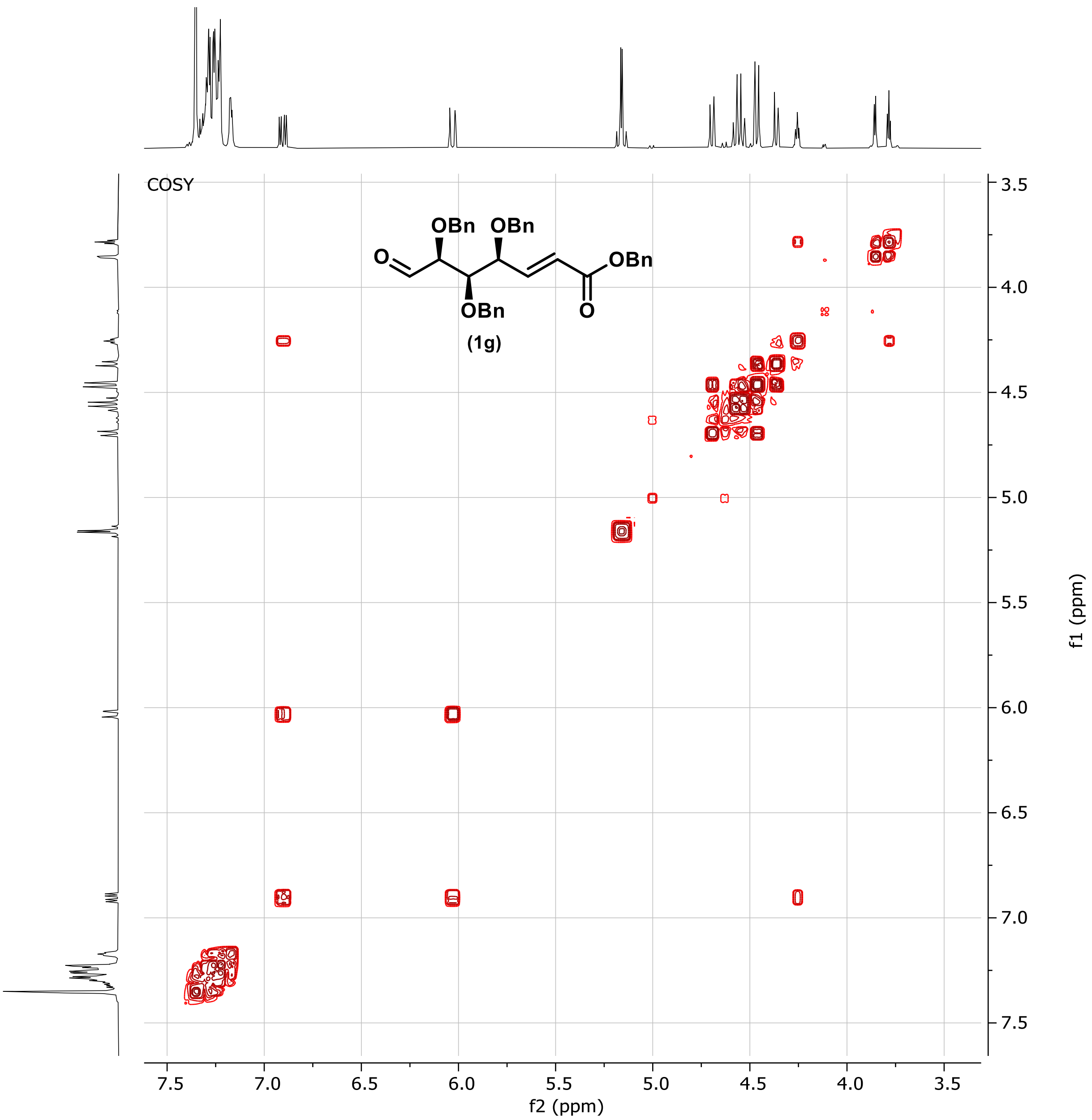

Figure S53. *COSY NMR of (1g)*

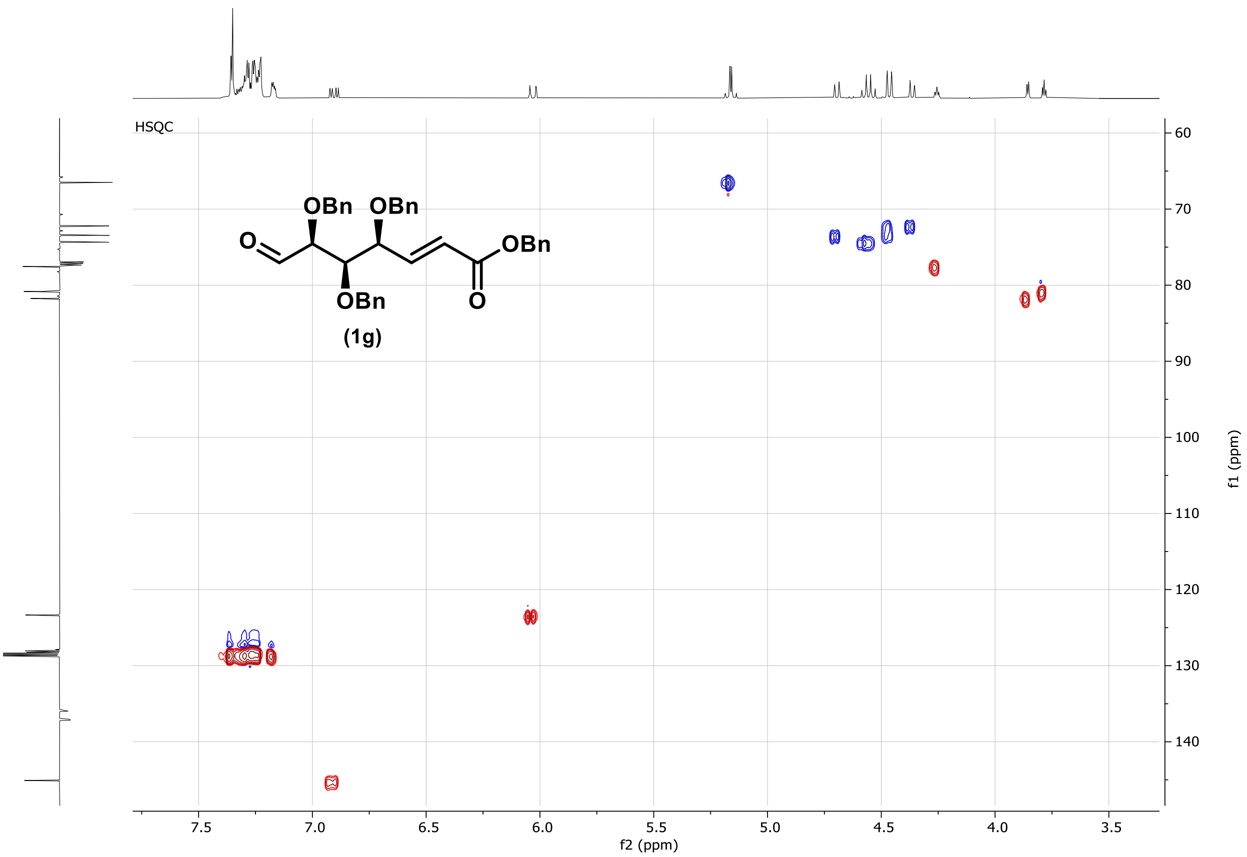

Figure S54. *HSQC NMR of (1g)*

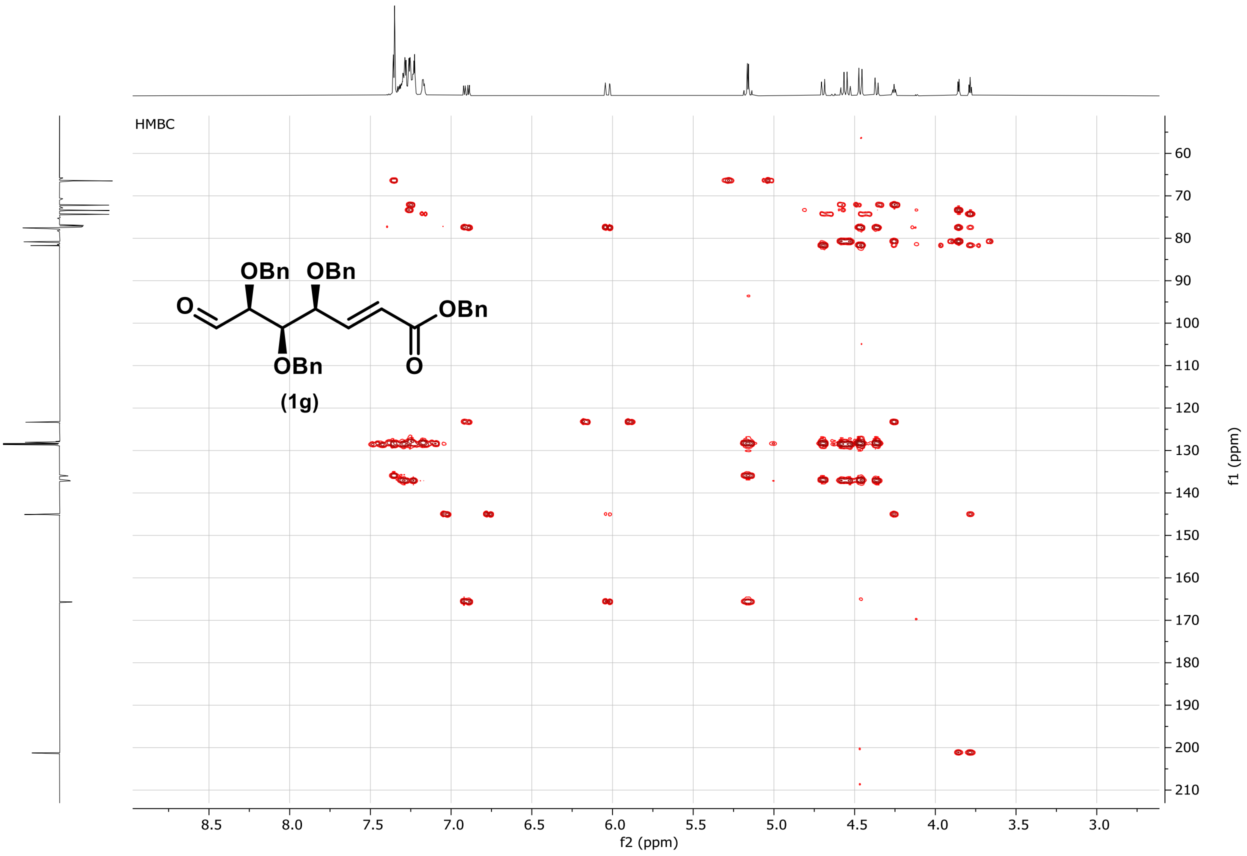

Figure S55. *HMBC NMR of (1g)*

D.1.12. Tert-butyl (4S,5R,6R,E)-4,5,6-tris(benzyloxy)-7-hydroxyhept-2-enoate (6h-e)

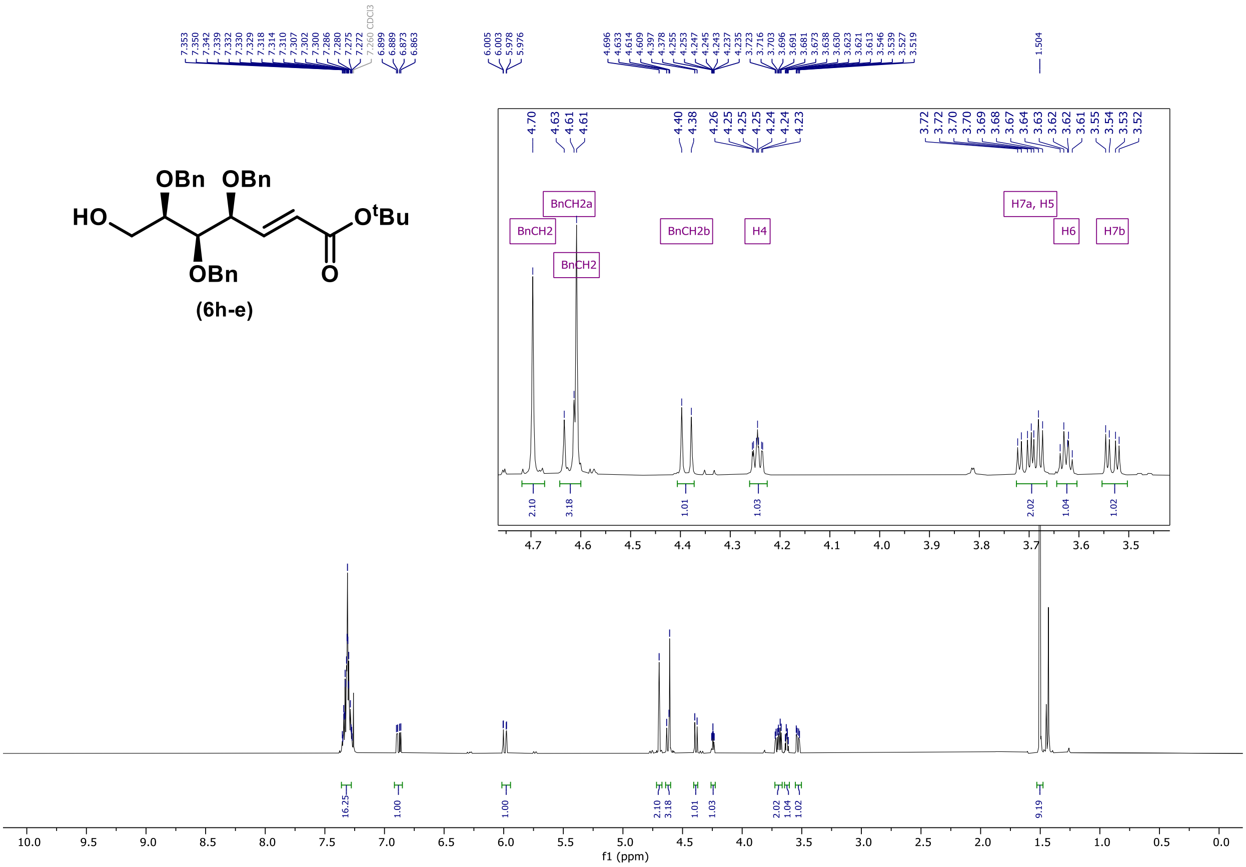

Figure S56. *600 MHz ^{1}H-NMR of (6h-e)*

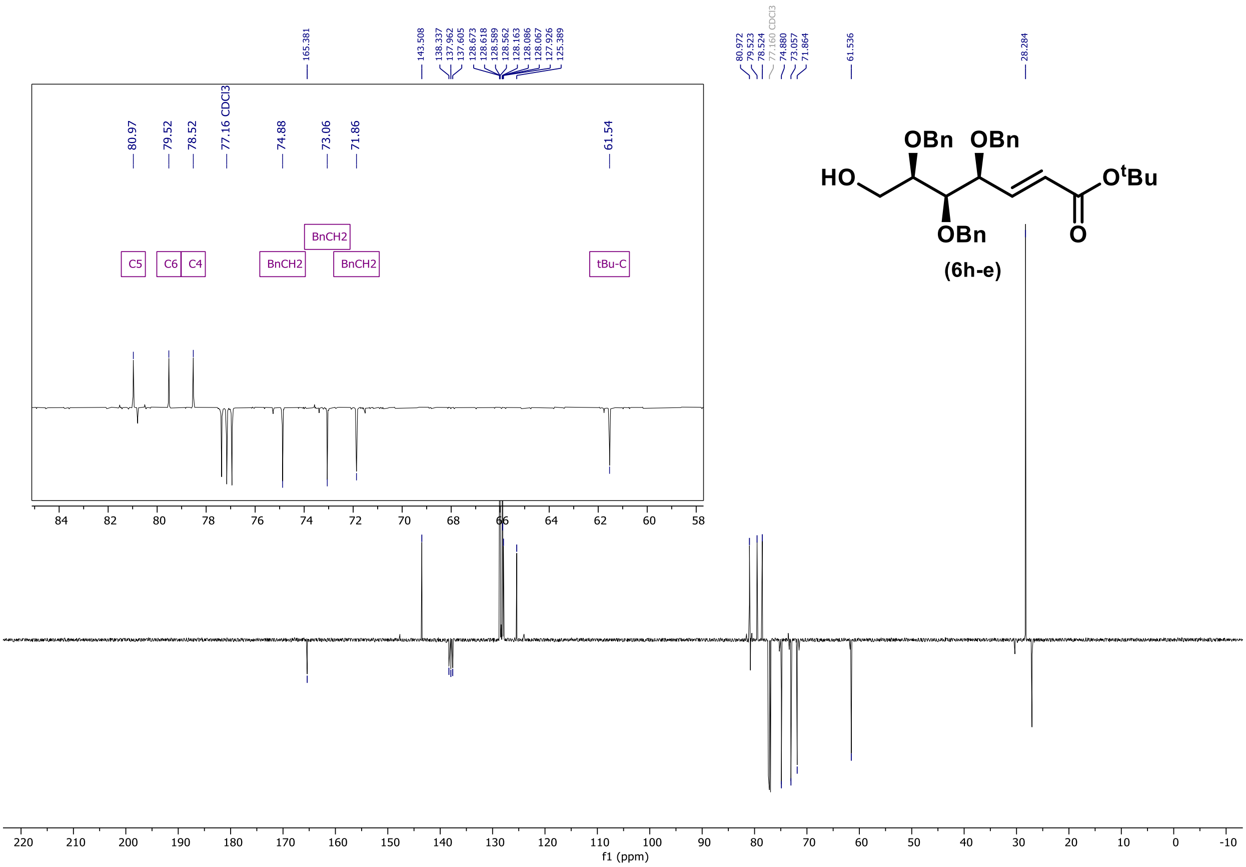

Figure S57. *151 MHz ^{13}C{^{1}H}-NMR of (6h-e)*

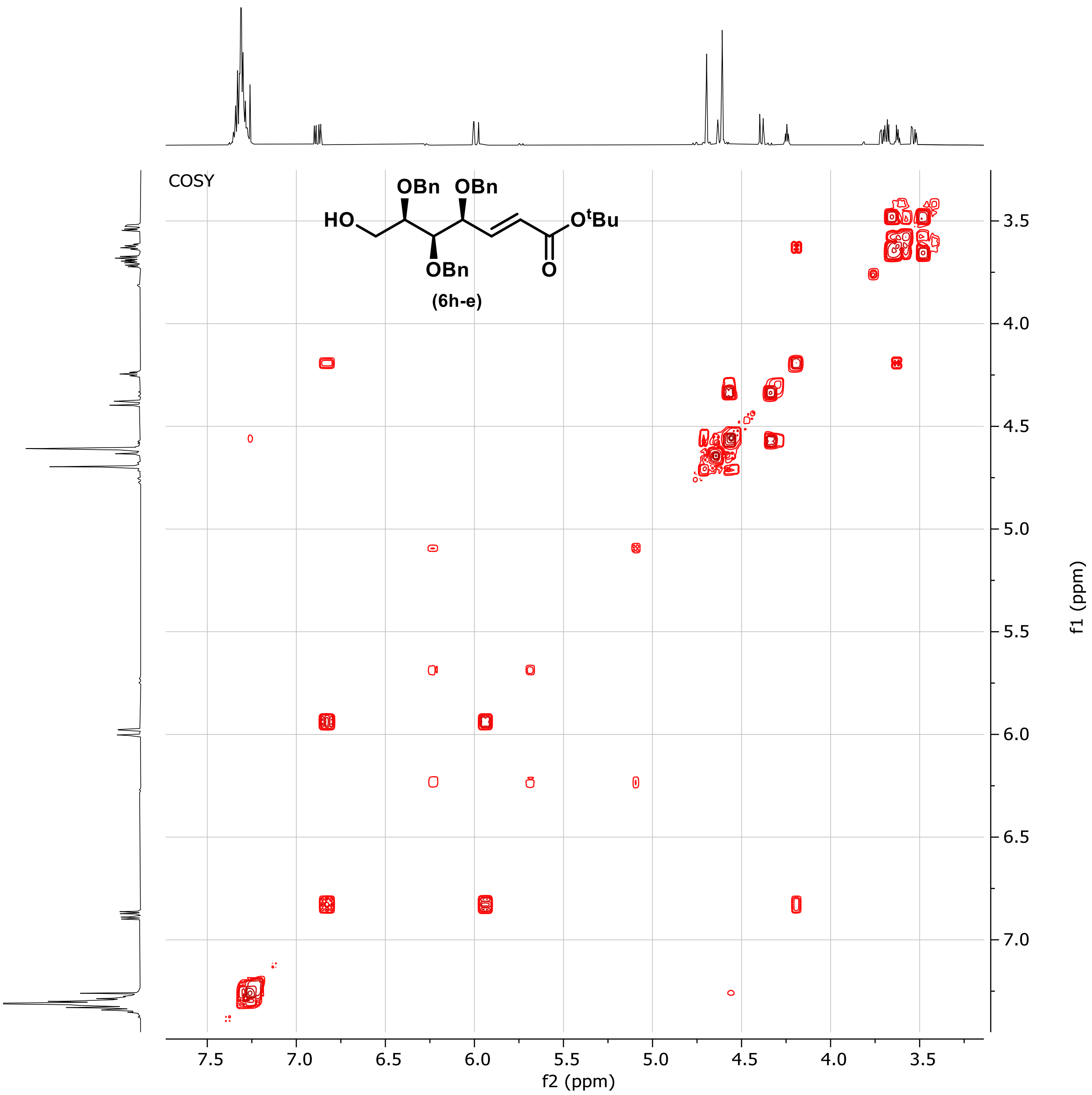

Figure S58. *COSY NMR of (6h-e)*

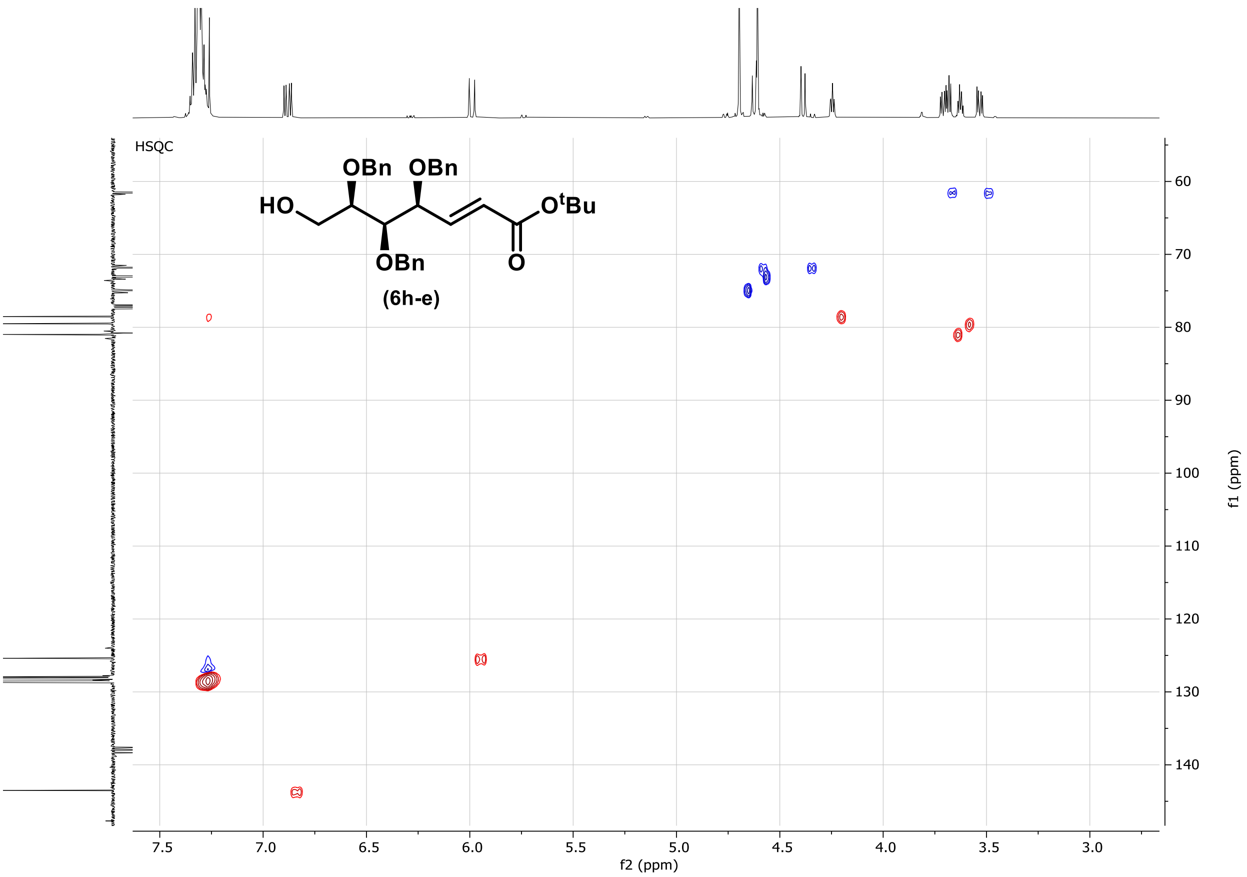

Figure S59. HSQC NMR of (6h-e)

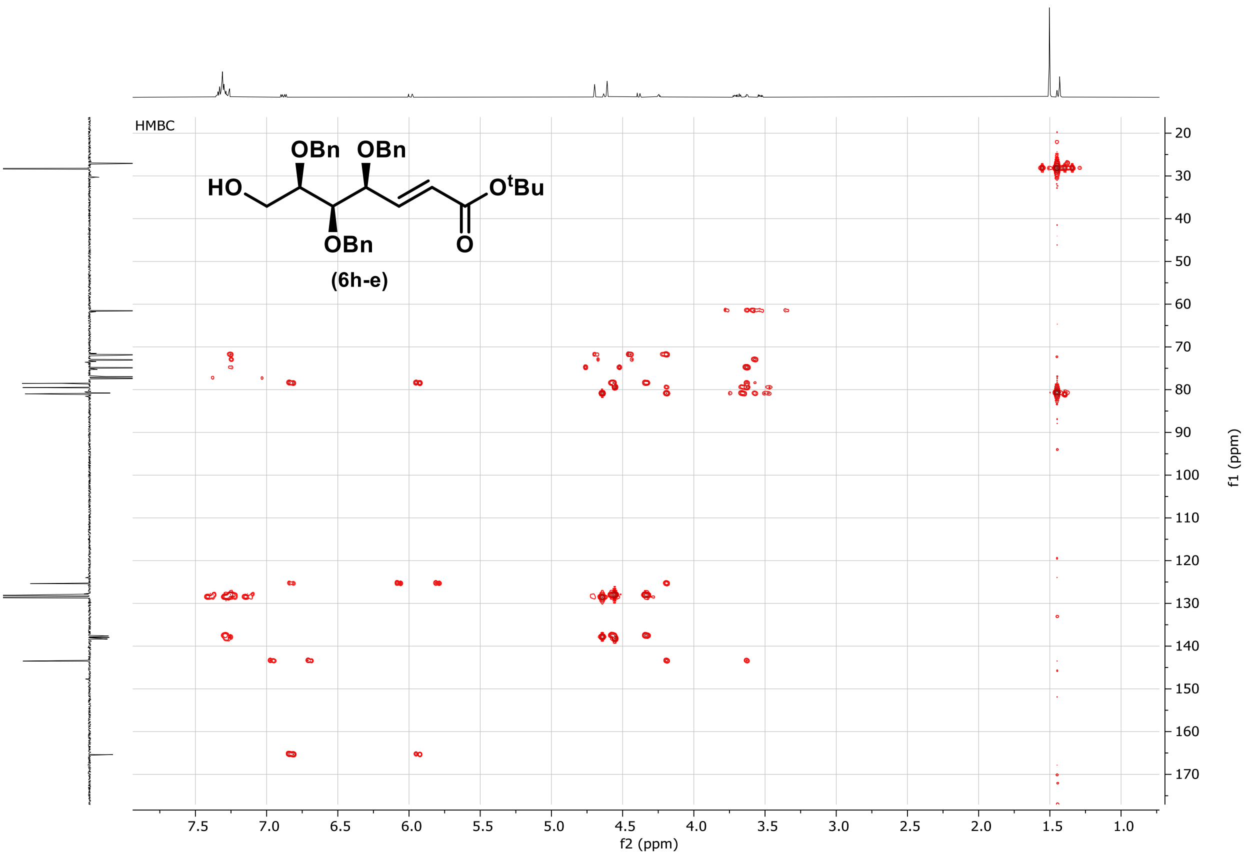

Figure S60. HMBC NMR of (6h-e)

D.1.13. Tert-butyl (4S,5R,6R,Z)-4,5,6-tris(benzyloxy)-7-hydroxyhept-2-enoate (6h-z)

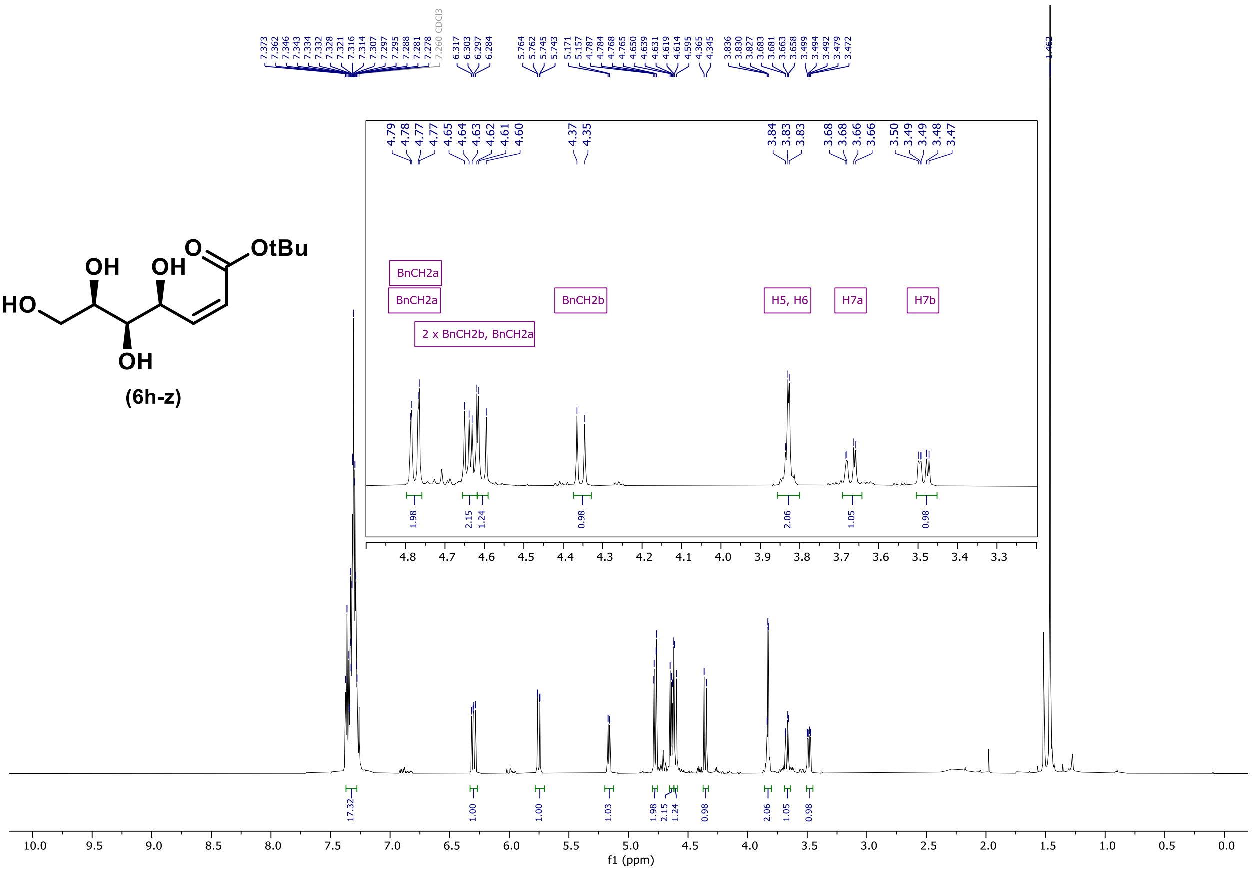

Figure S61. 600 MHz ^{1}H-NMR of (6h-z)

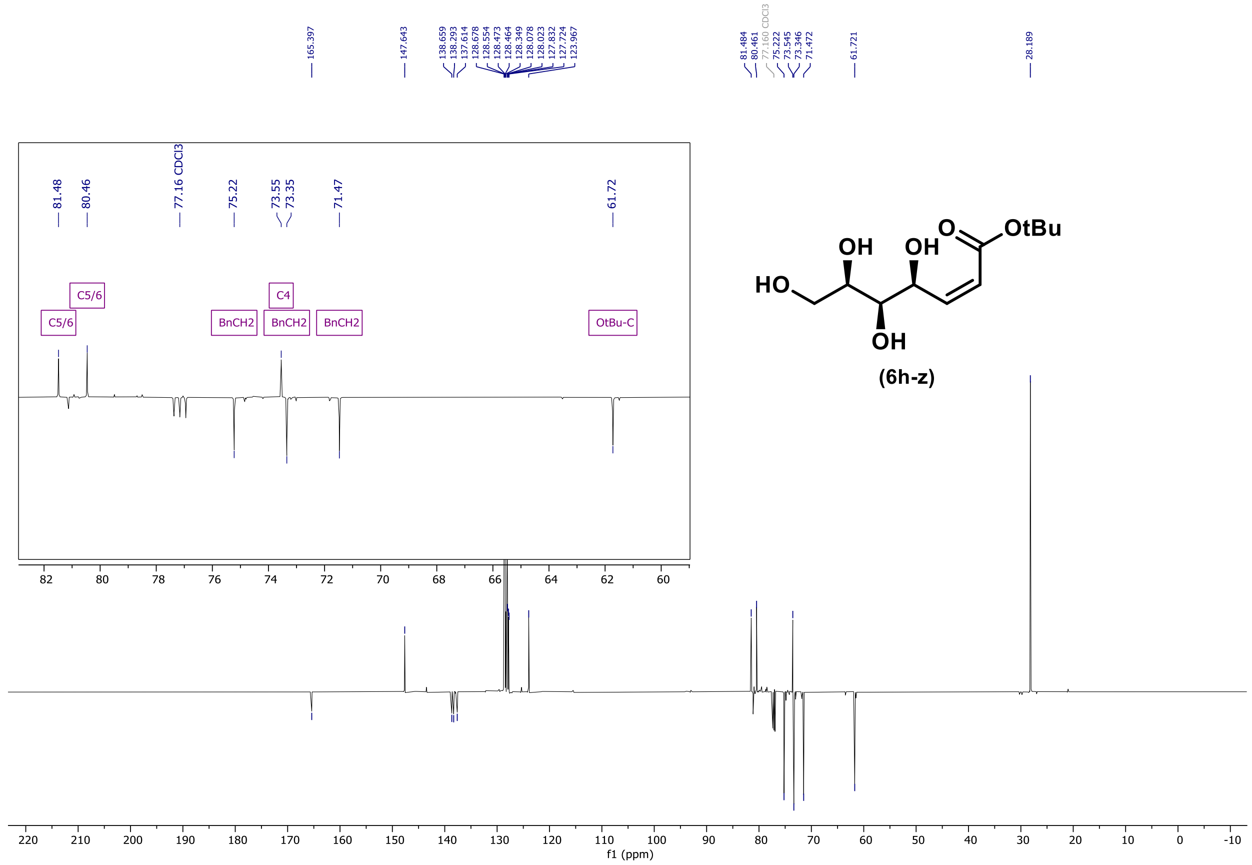

Figure S62. 151 MHz $^{13}C\{^{1}H\}$-NMR of (6h-z)

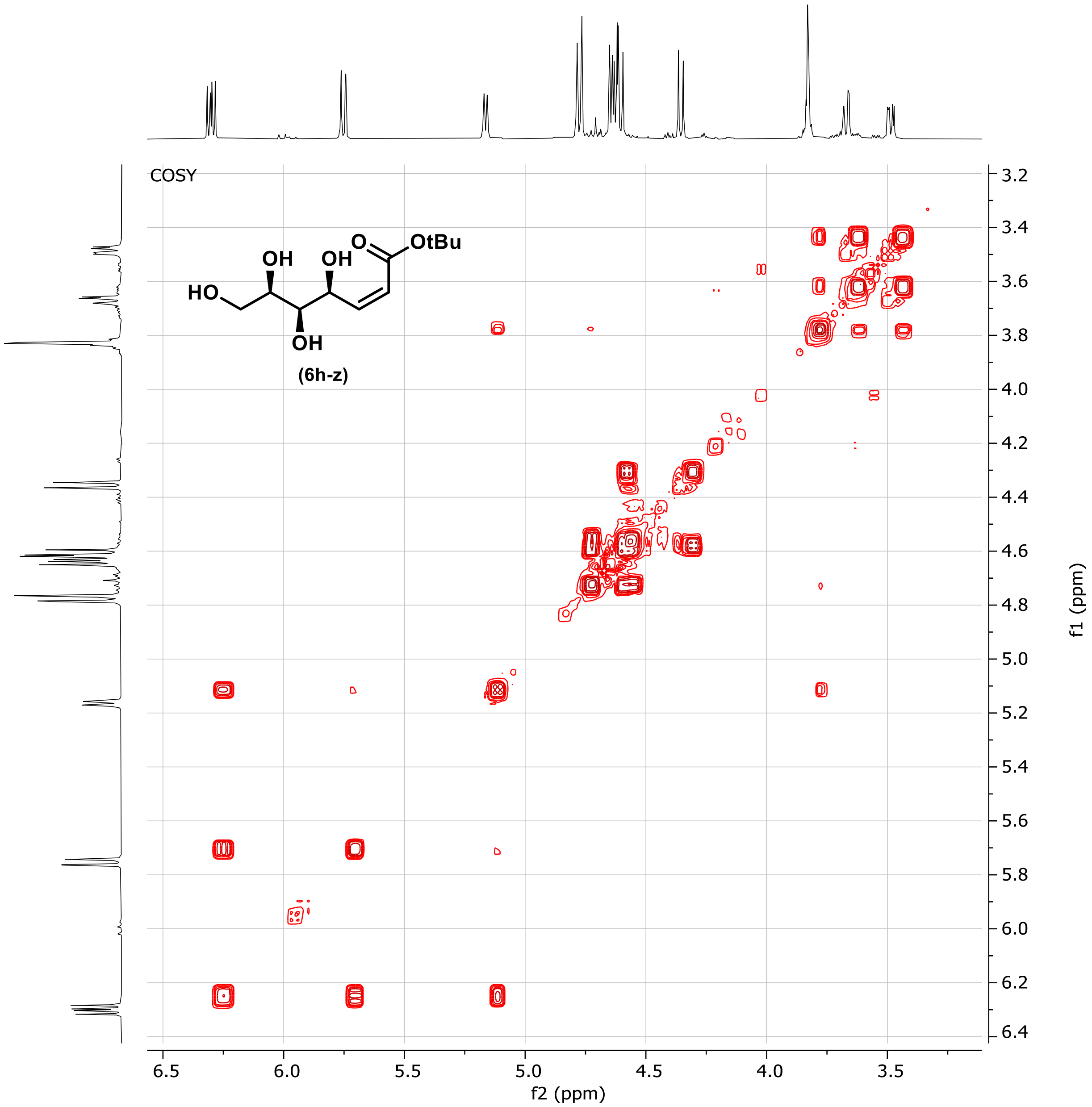

Figure S63. COSY NMR of (6h-z)

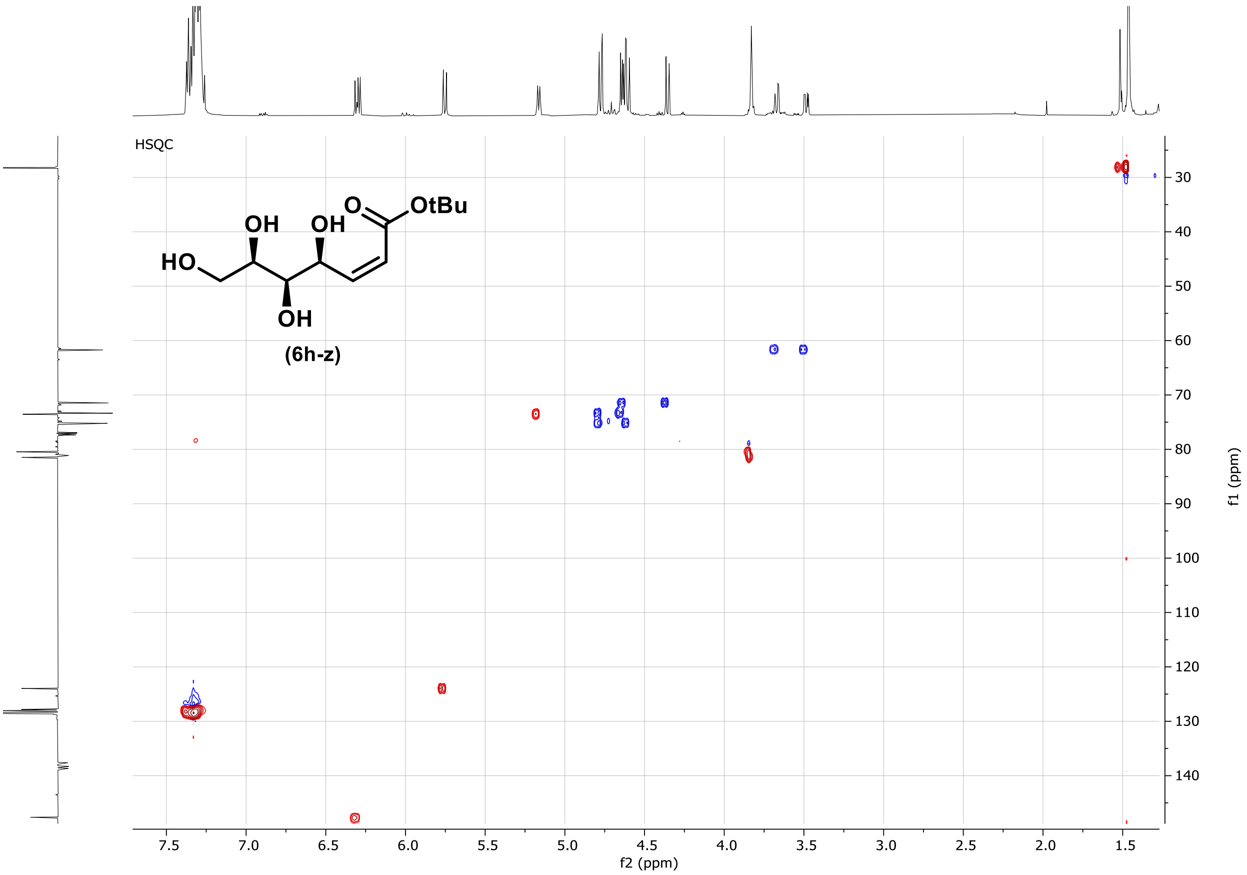

Figure S64. *HSQC NMR of (6h-z)*

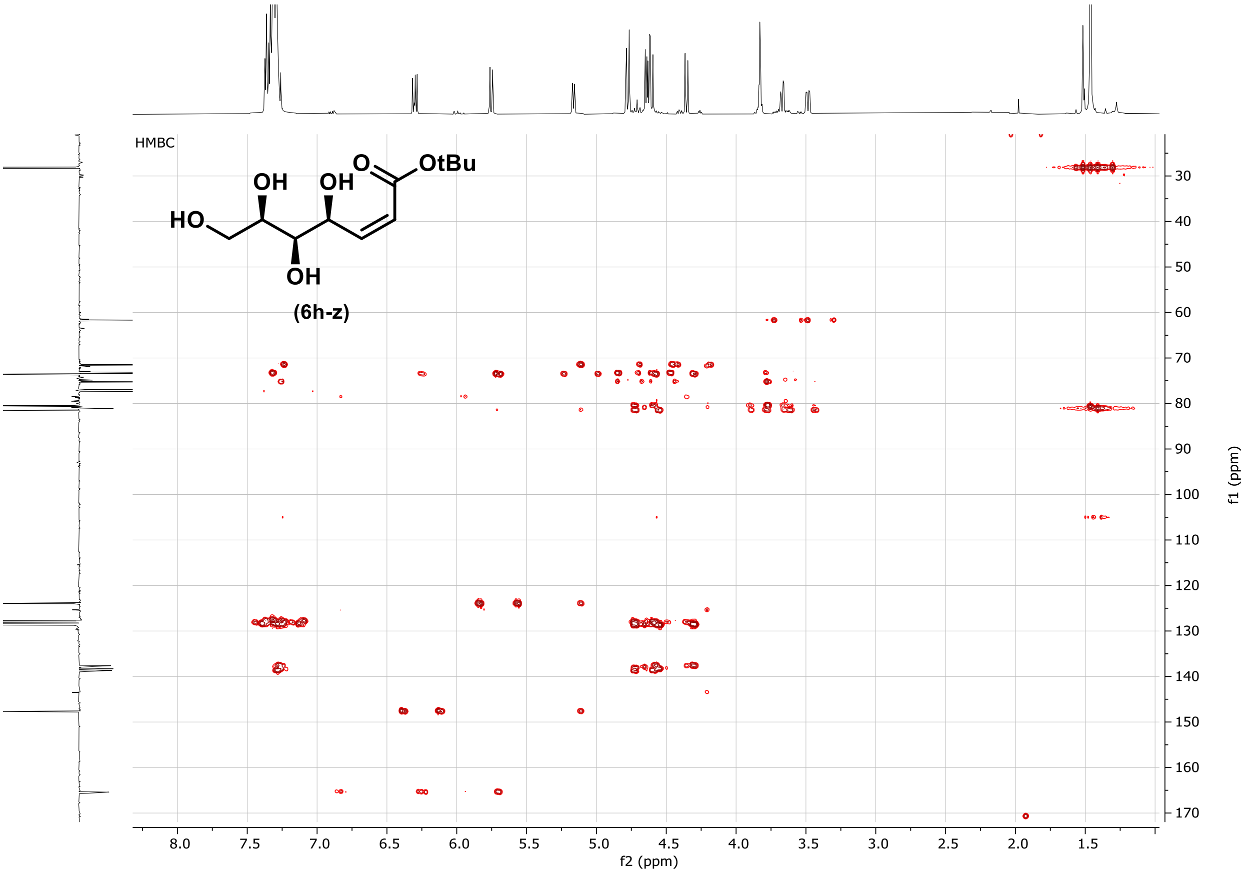

Figure S65. *HMBC NMR of (6h-z)*

D.1.14. Tert. butyl (4S, 5R, 6S, E)-4,5,6-tris(benzyloxy)-7-oxohept-2-enoate (1h)

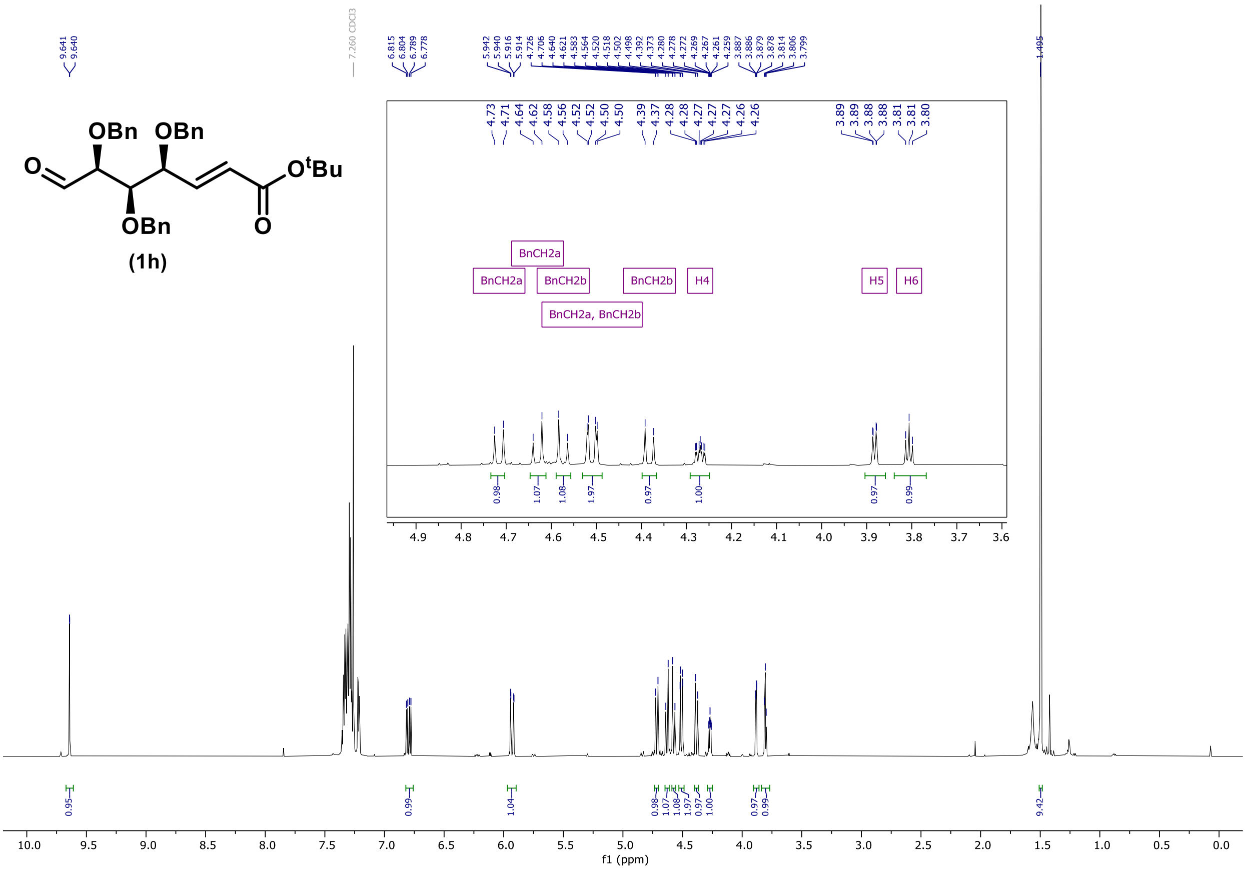

Figure S66. 600 MHz ^{1}H-NMR of (1h)

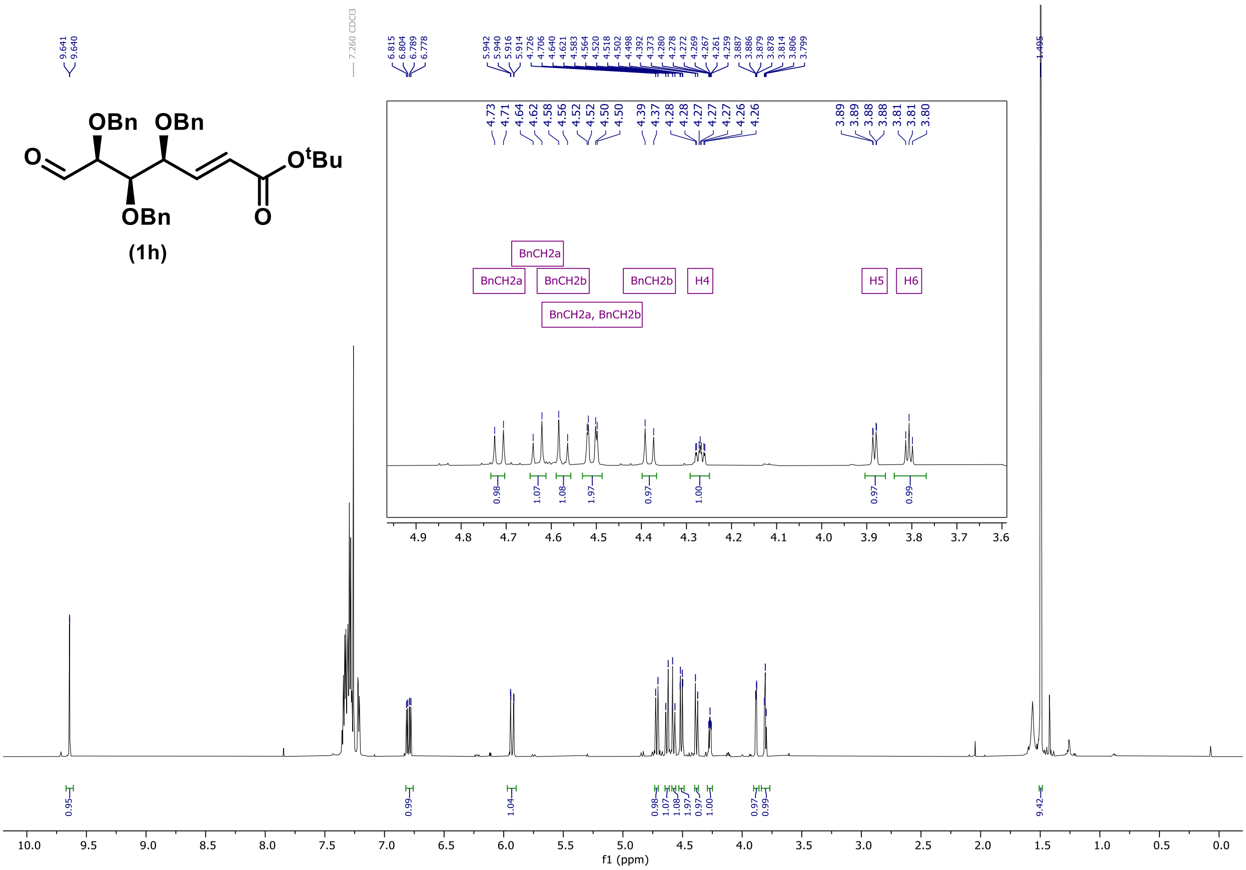

Figure S67. 151 MHz ^{13}C{^{1}H}-NMR of (1h)

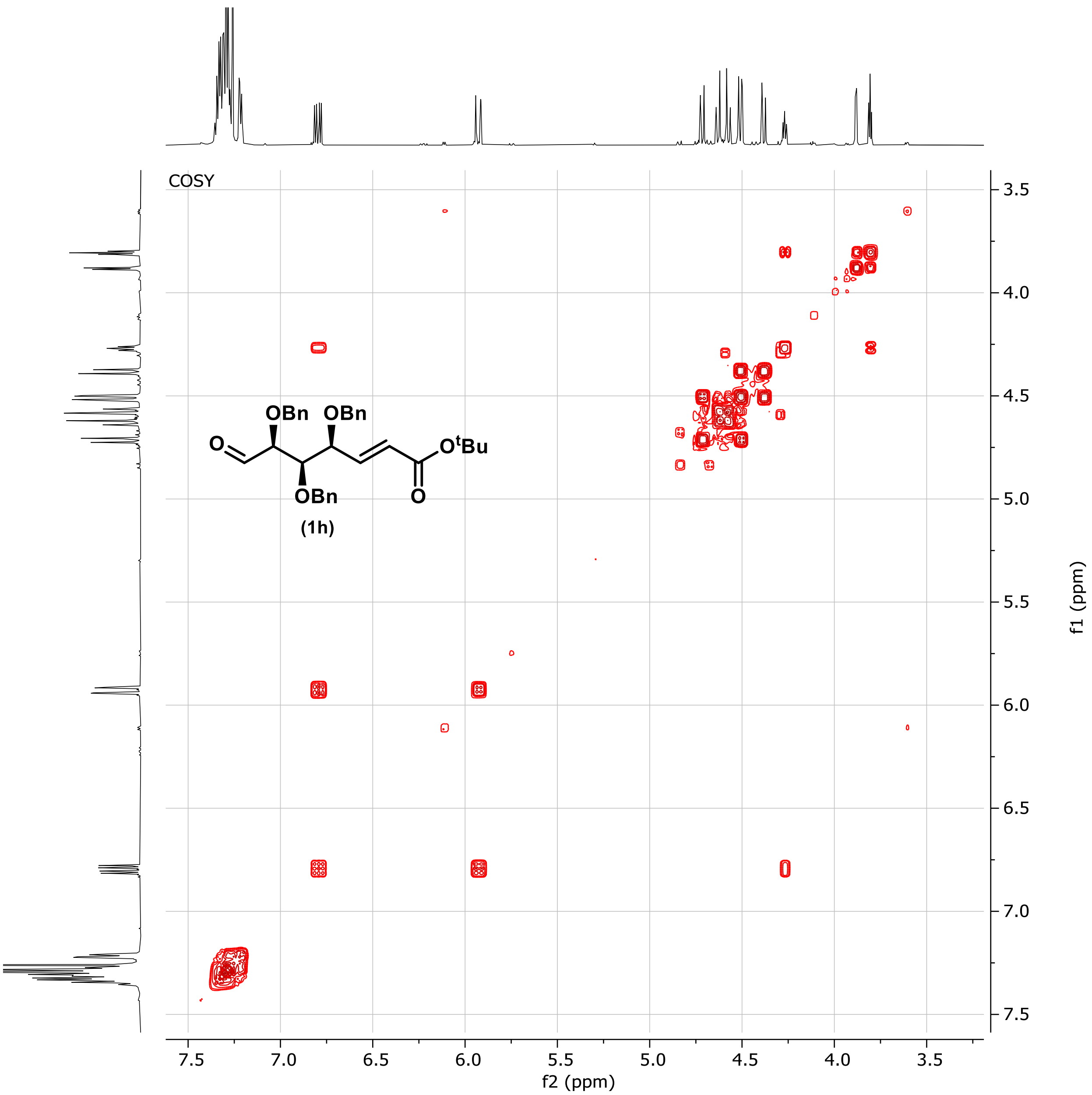

Figure S68. COSY NMR of (1h)

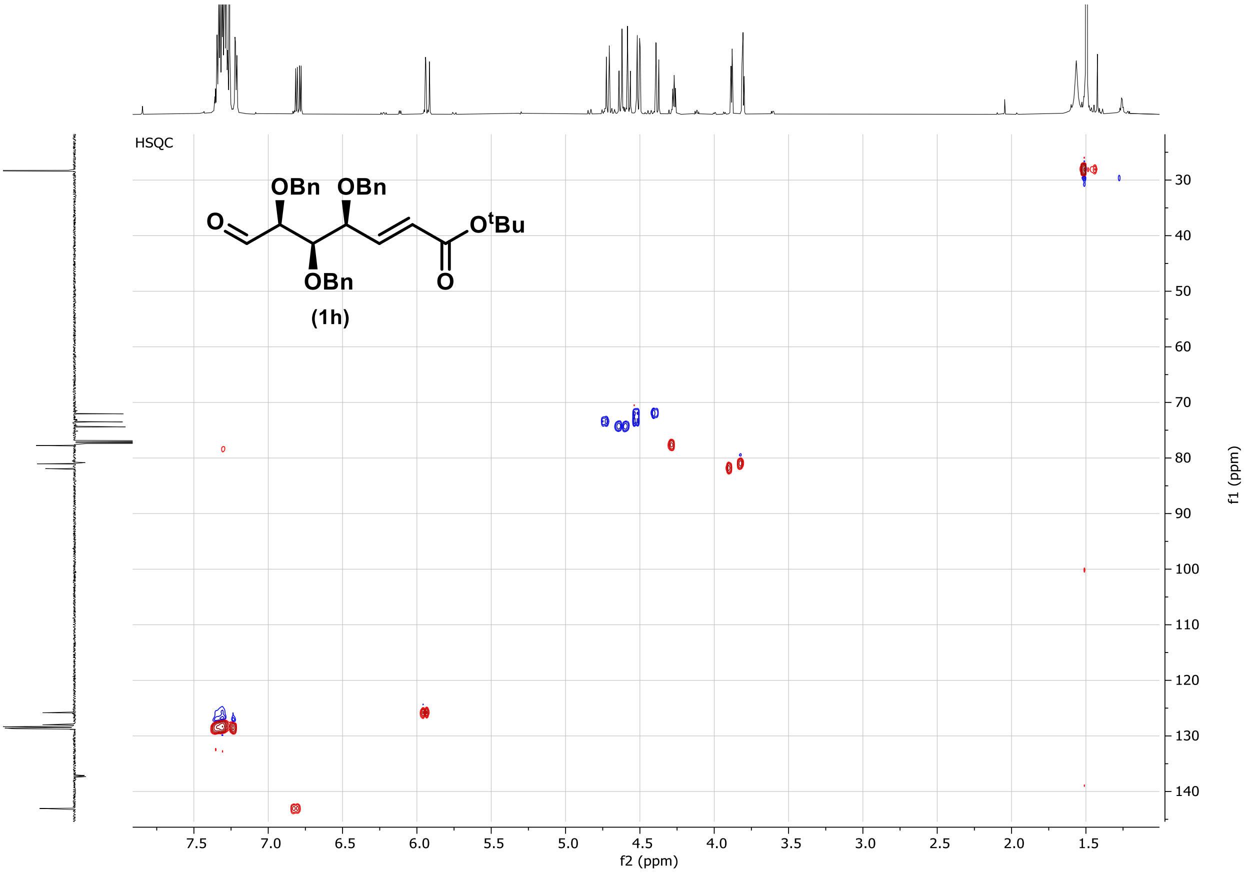

Figure S69. HSQC NMR of (1h)

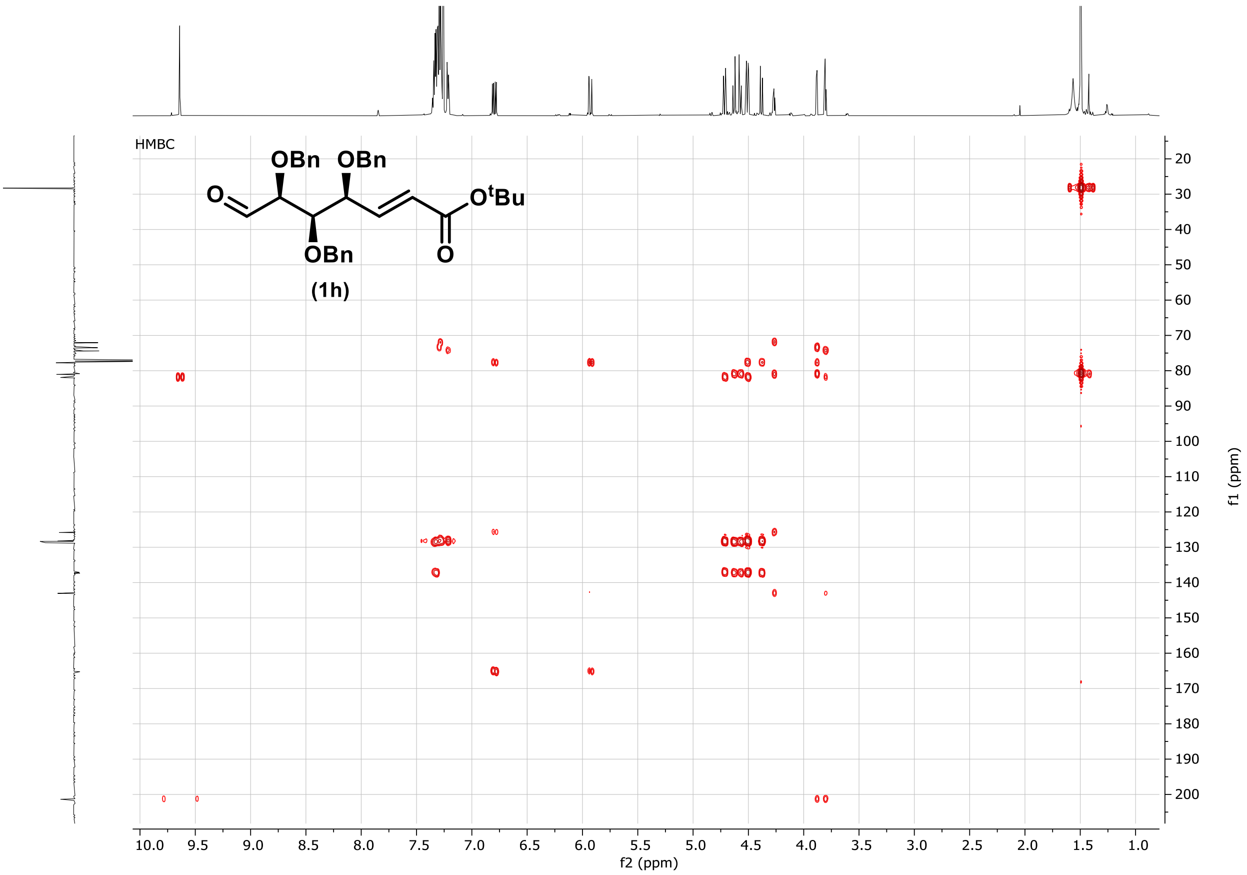

Figure S70. HMBC NMR of (1h)

D.1.15. (4S,5R,6R)-4,5,6-tris(benzyloxy)-7-hydroxyhept-2-enenitrile (6i)

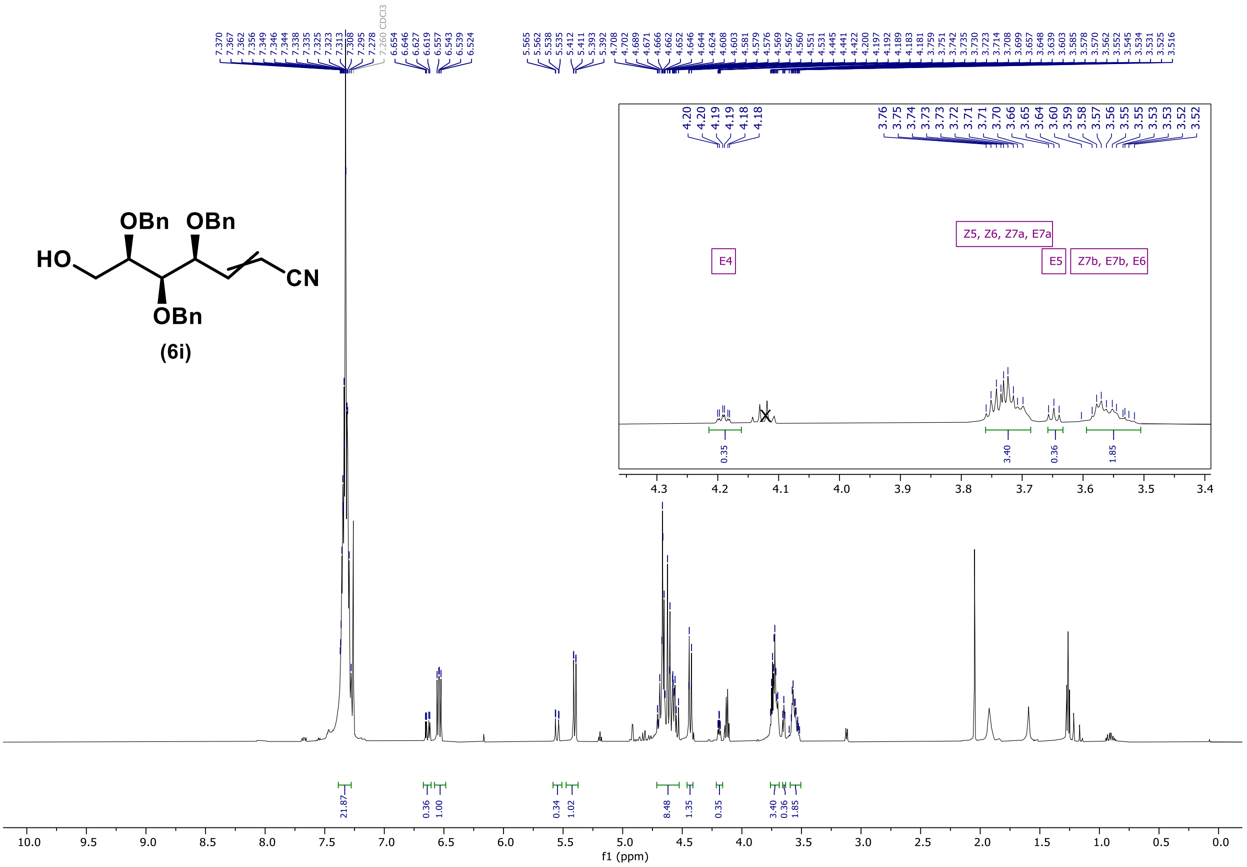

Figure S71. *600 MHz ^{1}H-NMR of (6i) as E/Z mixture (1:0.35)*

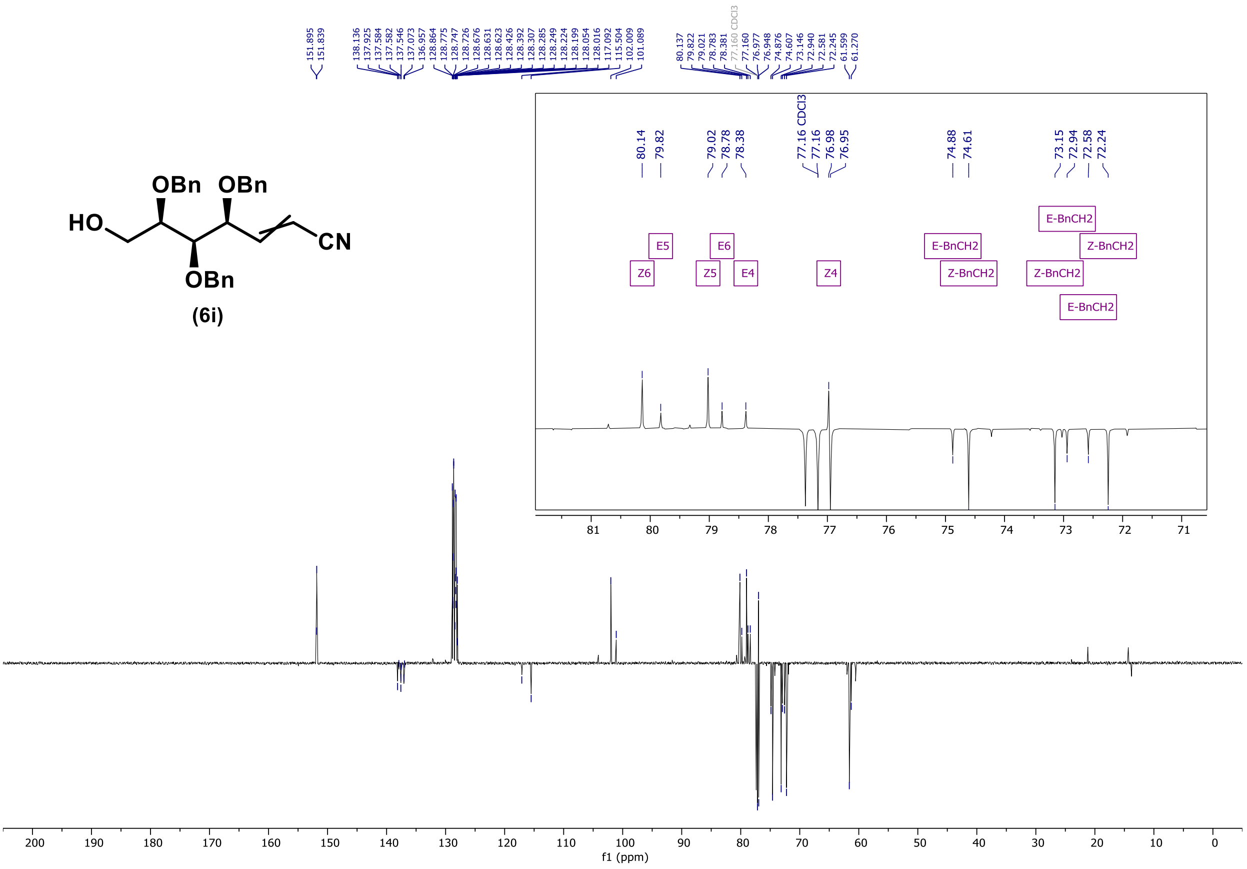

Figure 1. *151 MHz $^{13}C\{^{1}H\}$-NMR of (6i) as E/Z mixture (1:0.35)*

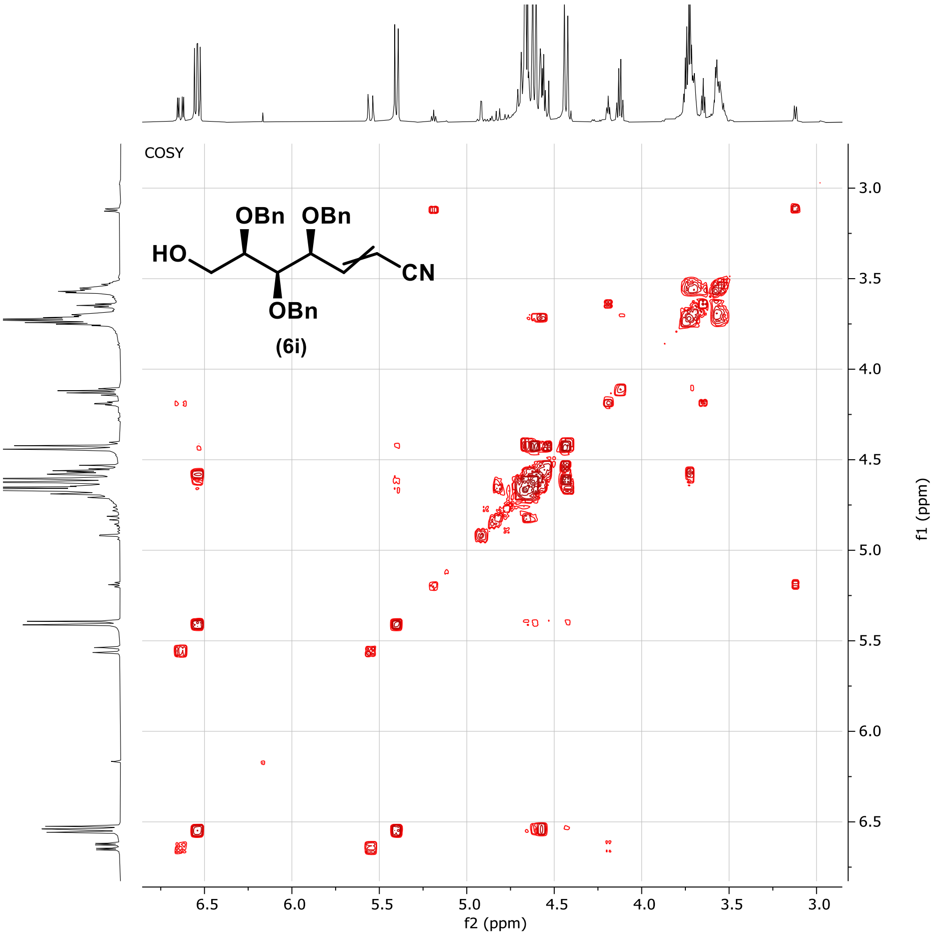

Figure S72. COSY NMR of (6i) as E/Z mixture (1:0.35)

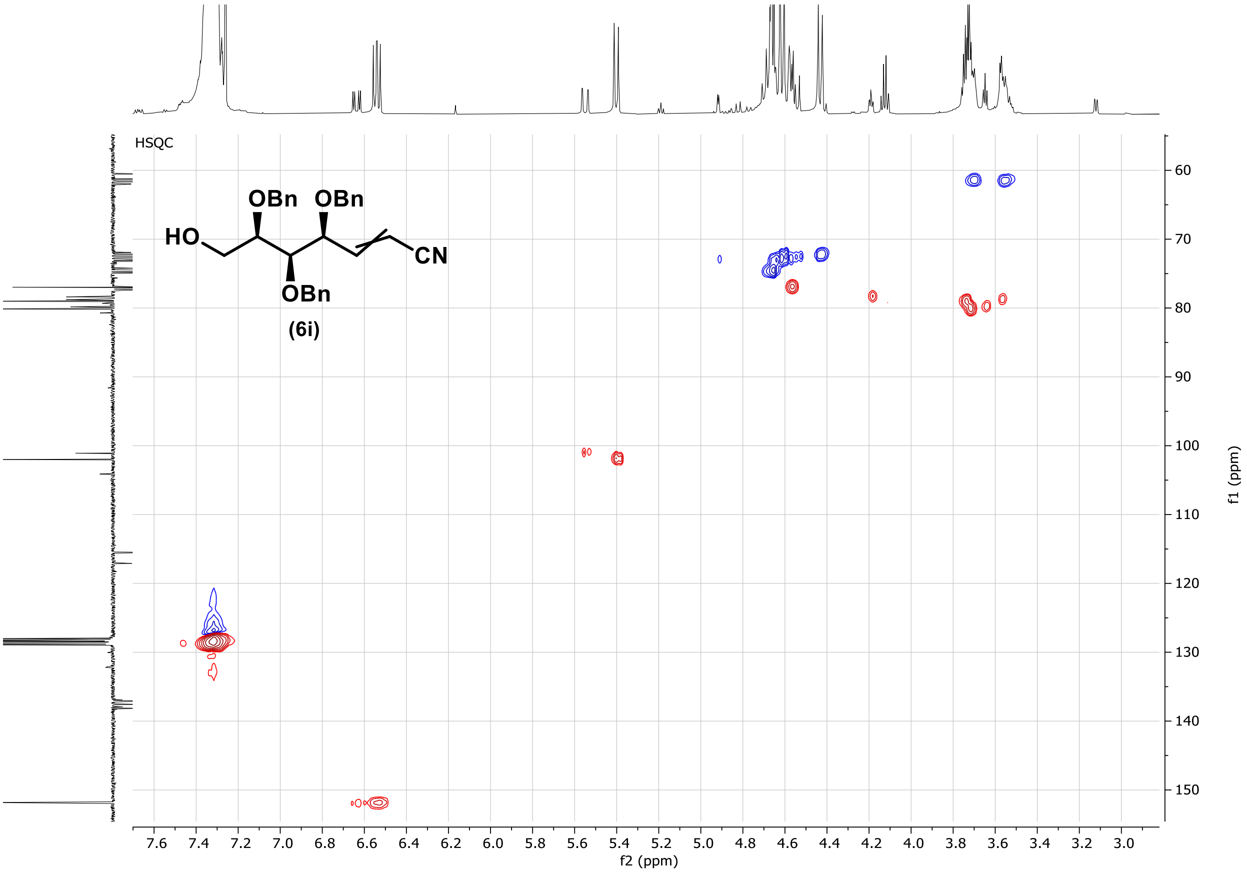

Figure S73. HSQC NMR of (6i) as E/Z mixture (1:0.35)

D.1.16. (4S,5R,6S)-4,5,6-tris(benzyloxy)-7-oxo-hept-2-enenitrile (1i)

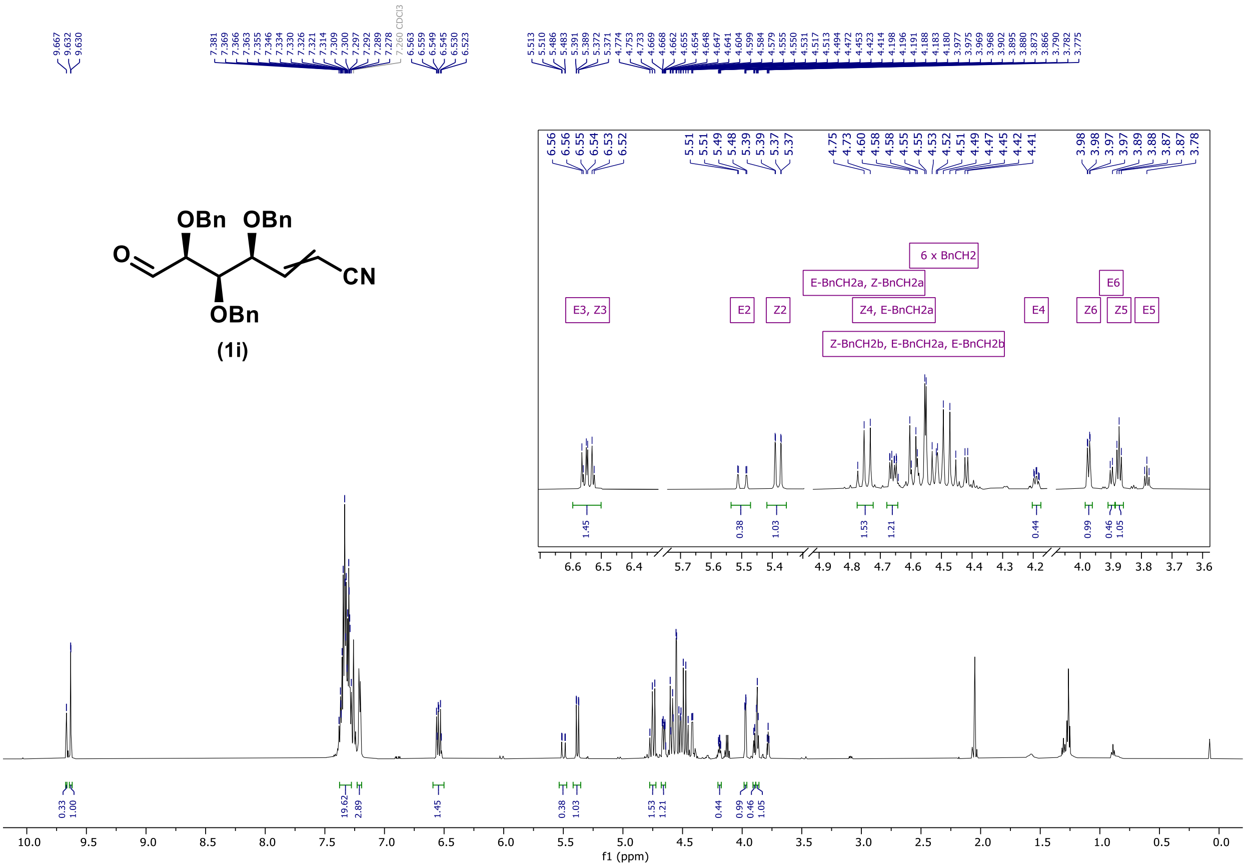

Figure S74. *600 MHz ^{1}H-NMR of (1i) E/Z mixture (1:0.35)*

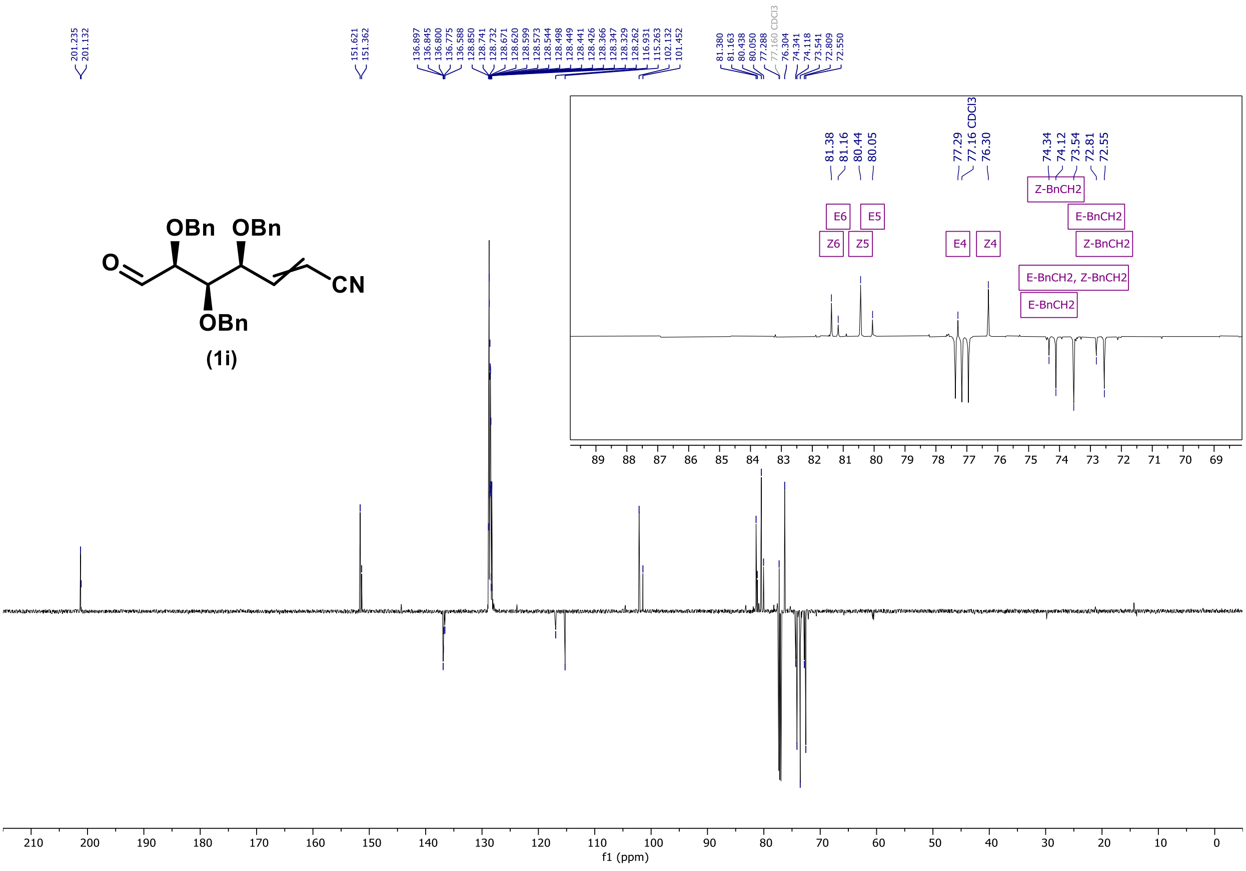

Figure S75. *151 MHz $^{13}C\{^1H\}$-NMR of (1i) as E/Z mixture (1:0.35)*

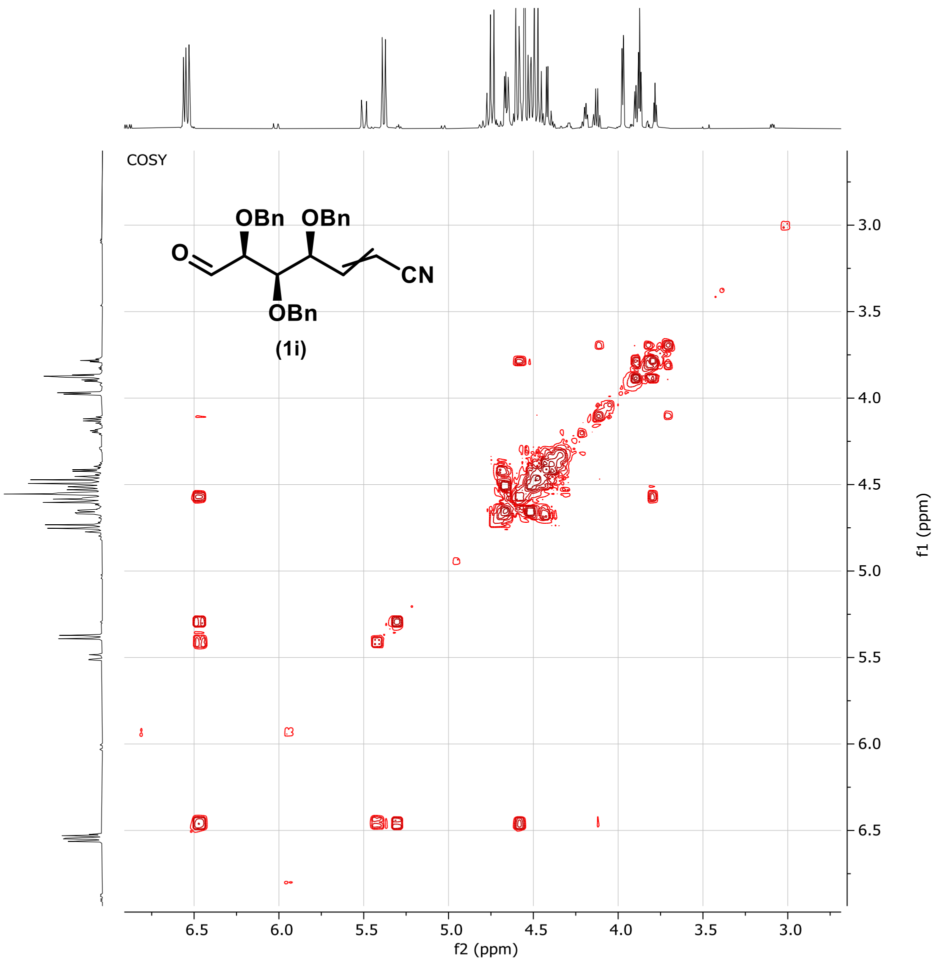

Figure S76. *COSY NMR of (1i) as E/Z mixture (1:0.35)*

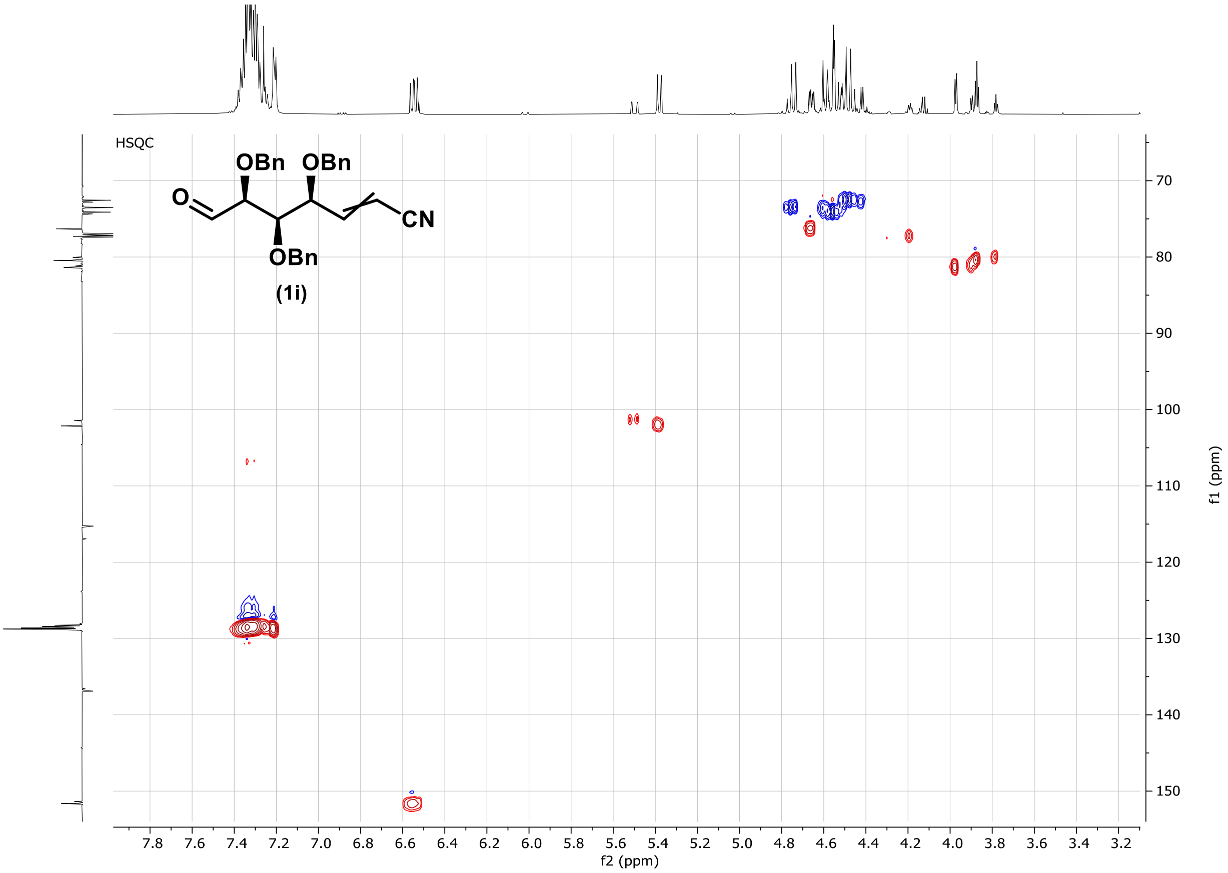

Figure S77. *HSQC NMR of (1i) as E/Z mixture (1:0.35)*

D.1.17. Methyl (4S,5R,6R,E)-4,5,6,7-tetrahydroxyhept-2-enoate (4e)

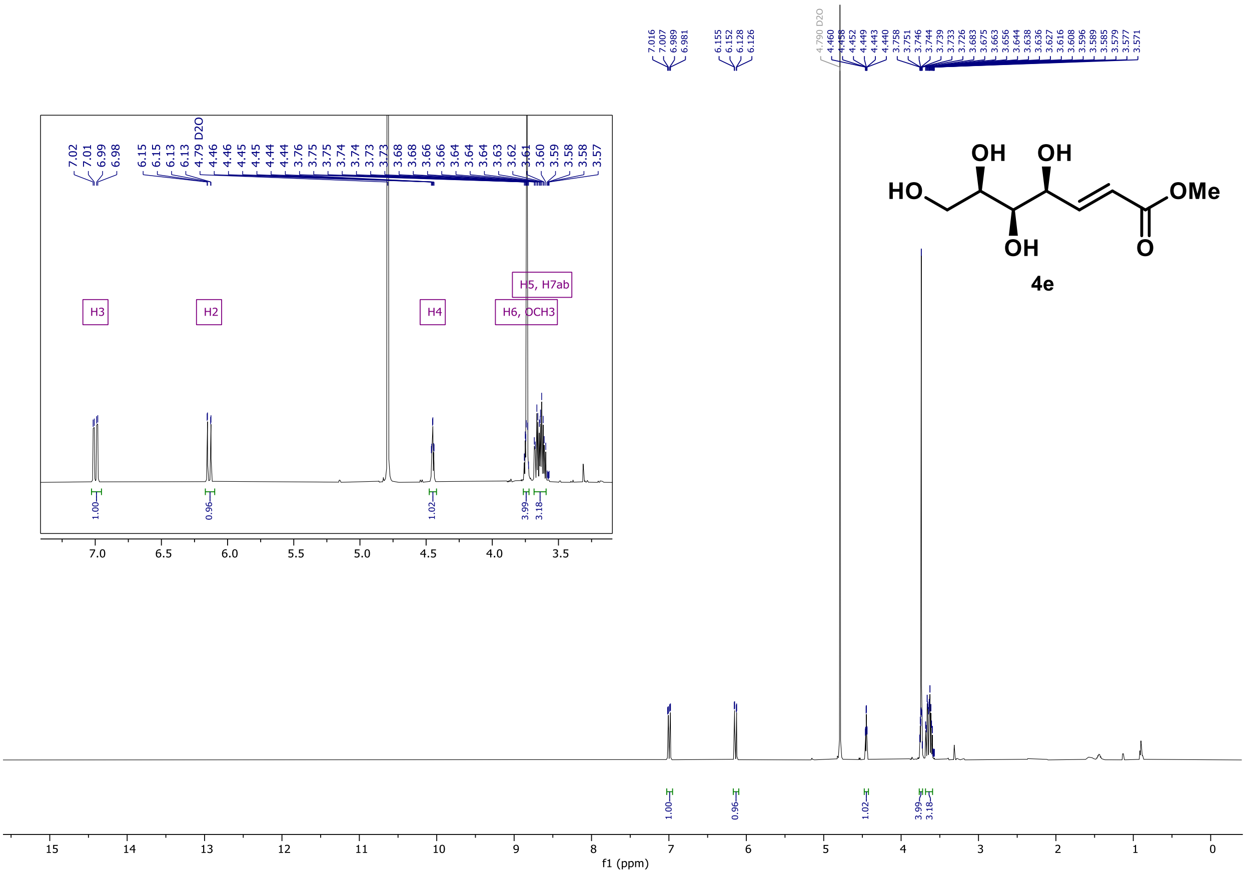

Figure S78. *600 MHz ^{1}H-NMR of (4e)*

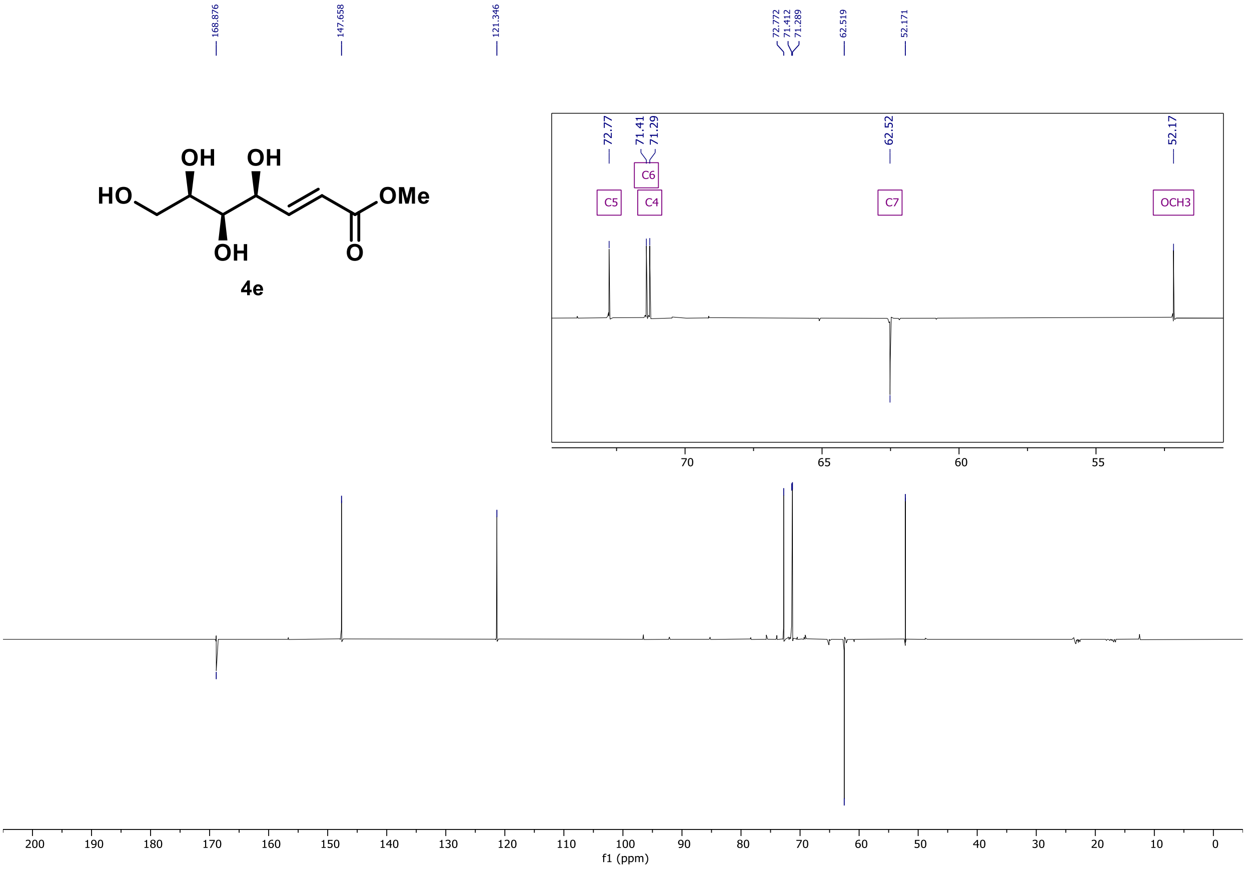

Figure S79. *151 MHz $^{13}C\{^1H\}$-NMR of (4e)*

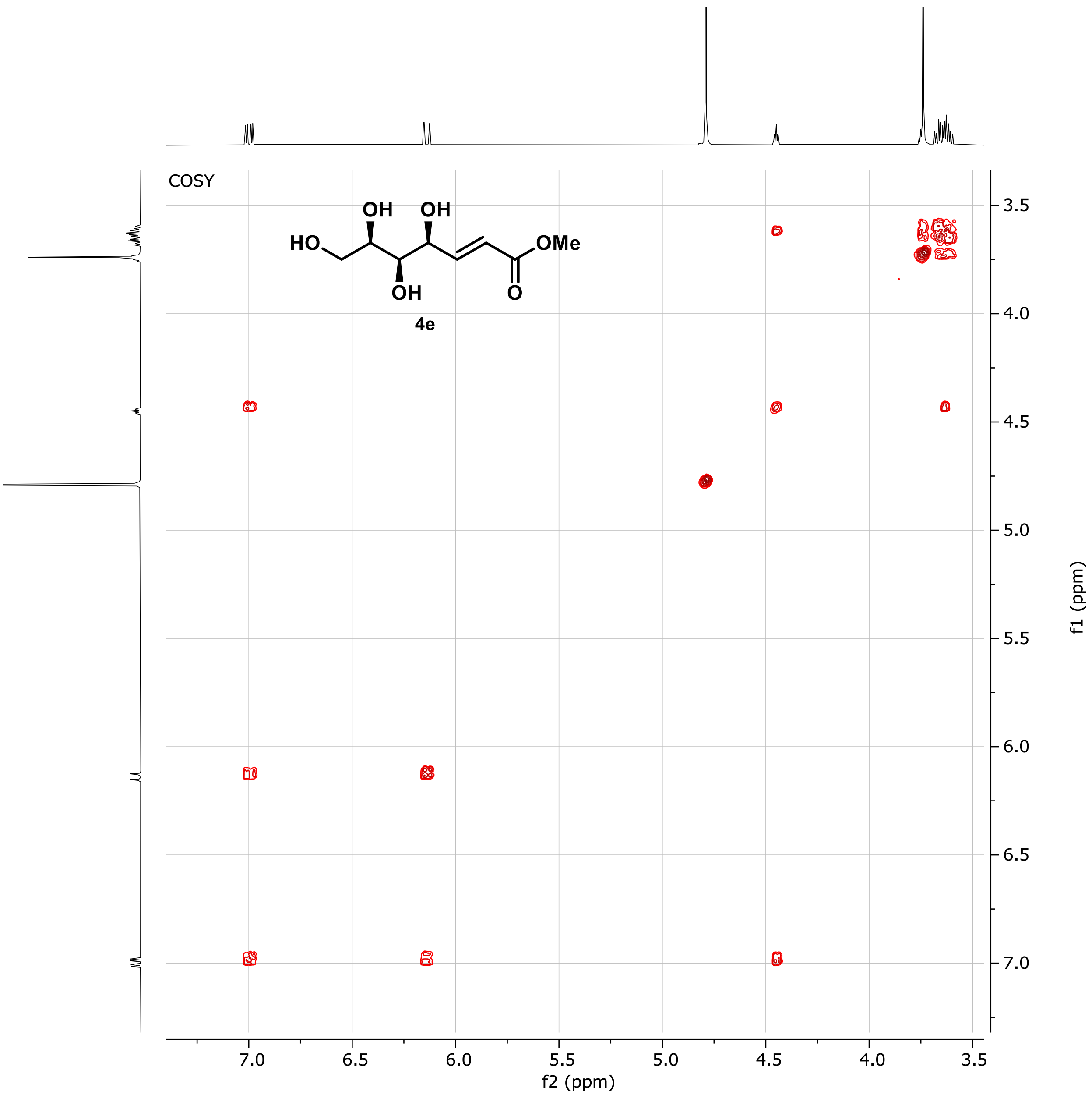

Figure S80. *COSY NMR of (4e)*

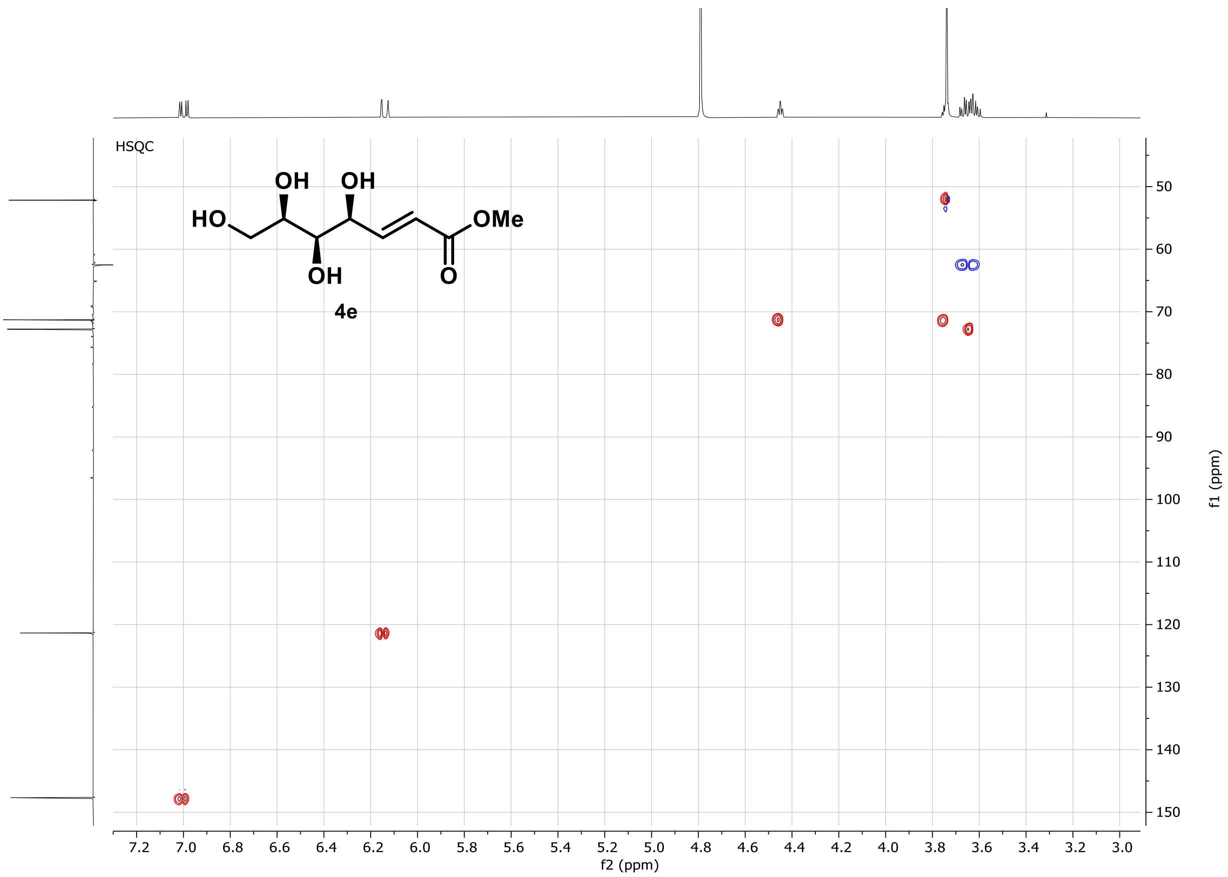

Figure S81. *HSQC NMR of (4e)*

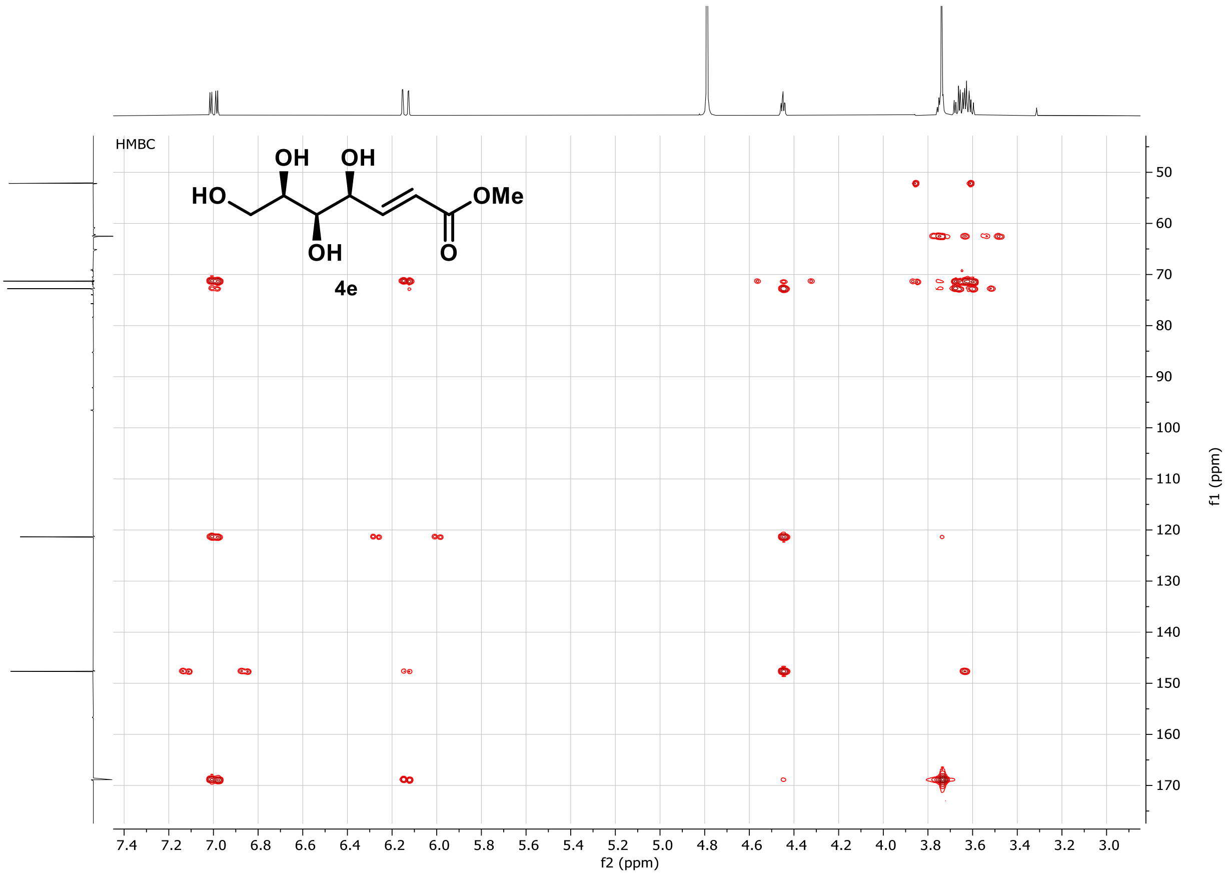

Figure S82. *HMBC NMR of (4e)*

D.1.18. Methyl (4S,5R,6S,E)-7-oxo-4,5,6-tris(trimethylsiloxy)hex-2-enoate (1e)

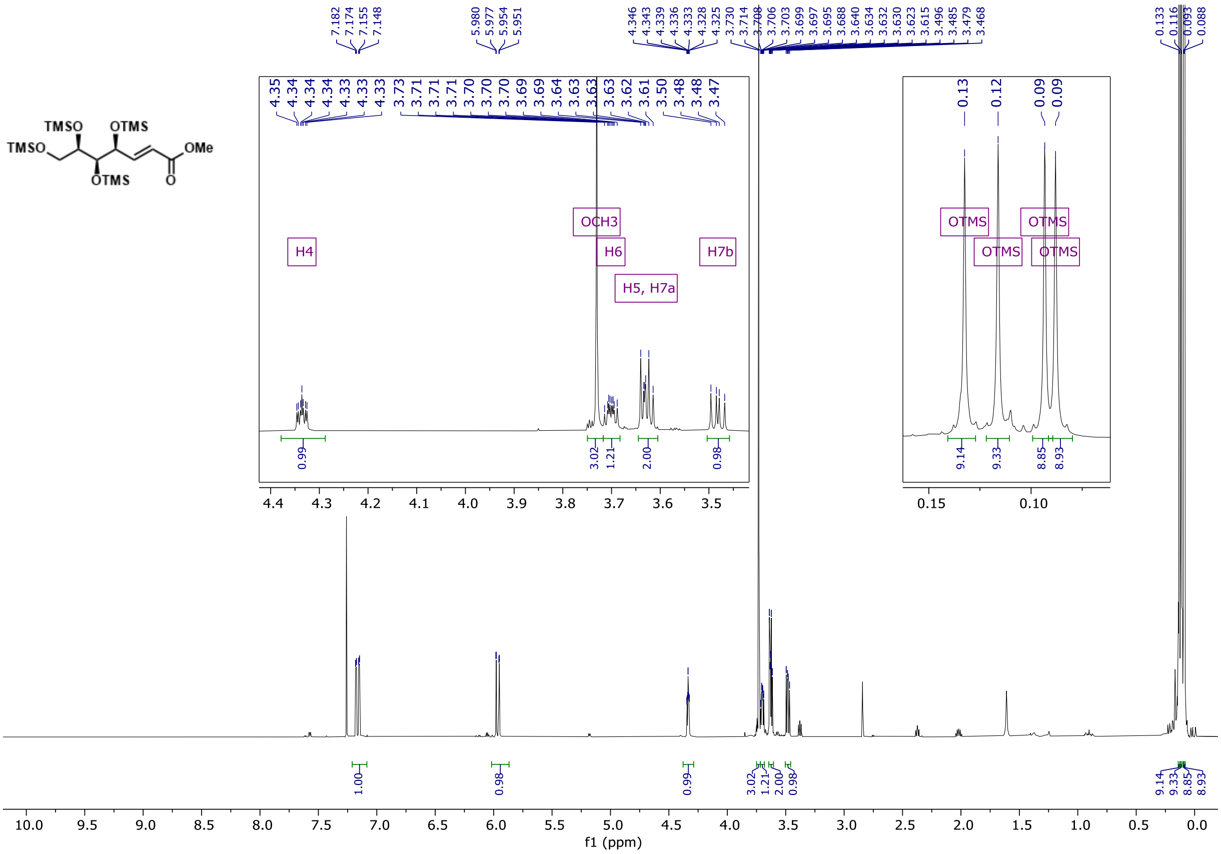

Figure S83. *600 MHz ^{1}H-NMR of intermediate per-silylated compound*

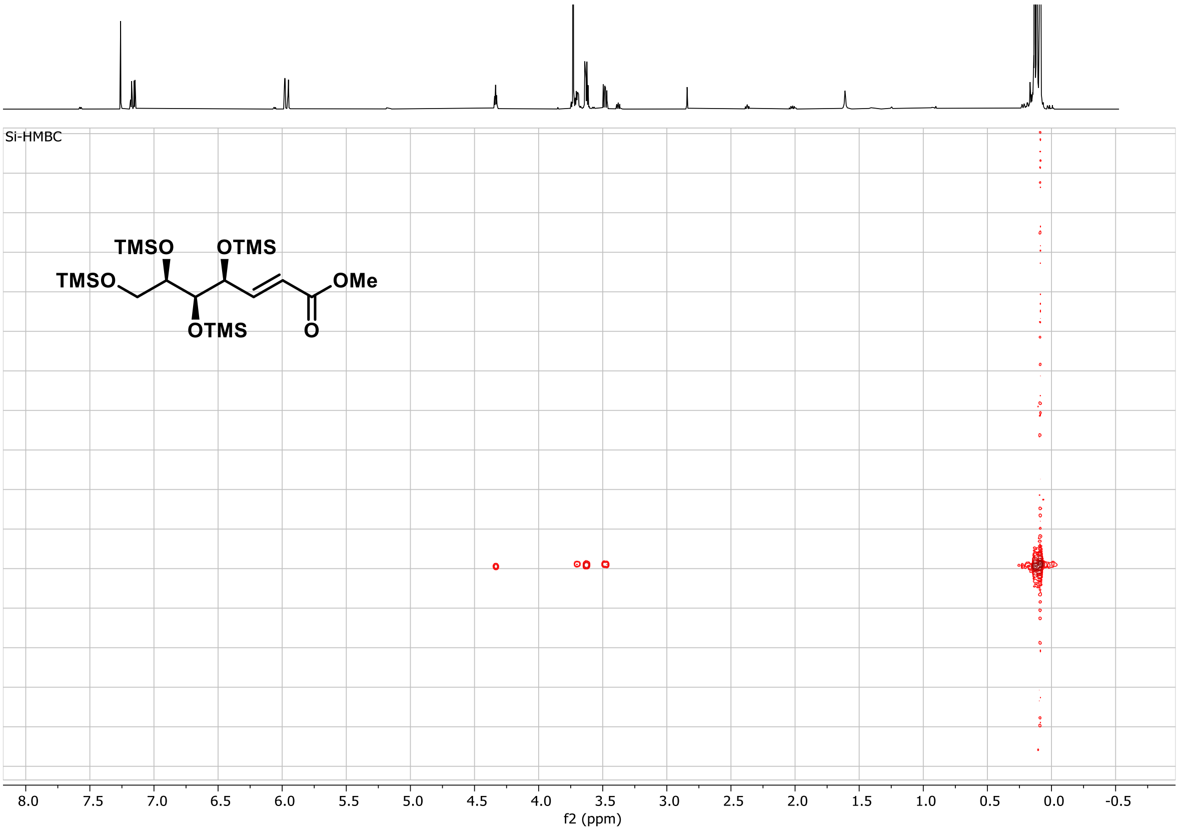

Figure 2. *Si-HMBC of intermediate per-silylated compound, showing four silyl groups*

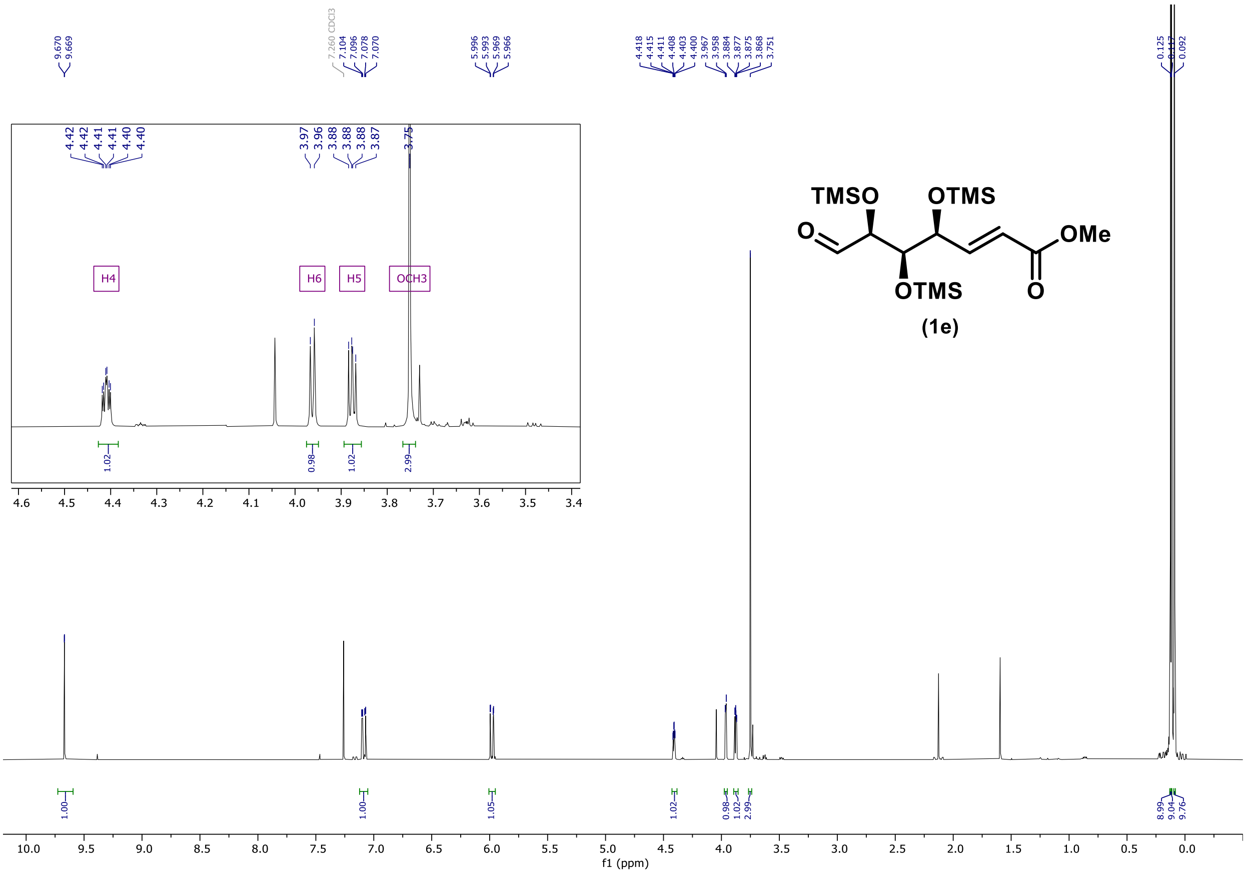

Figure S84. *600 MHz ¹H-NMR of (1e)*

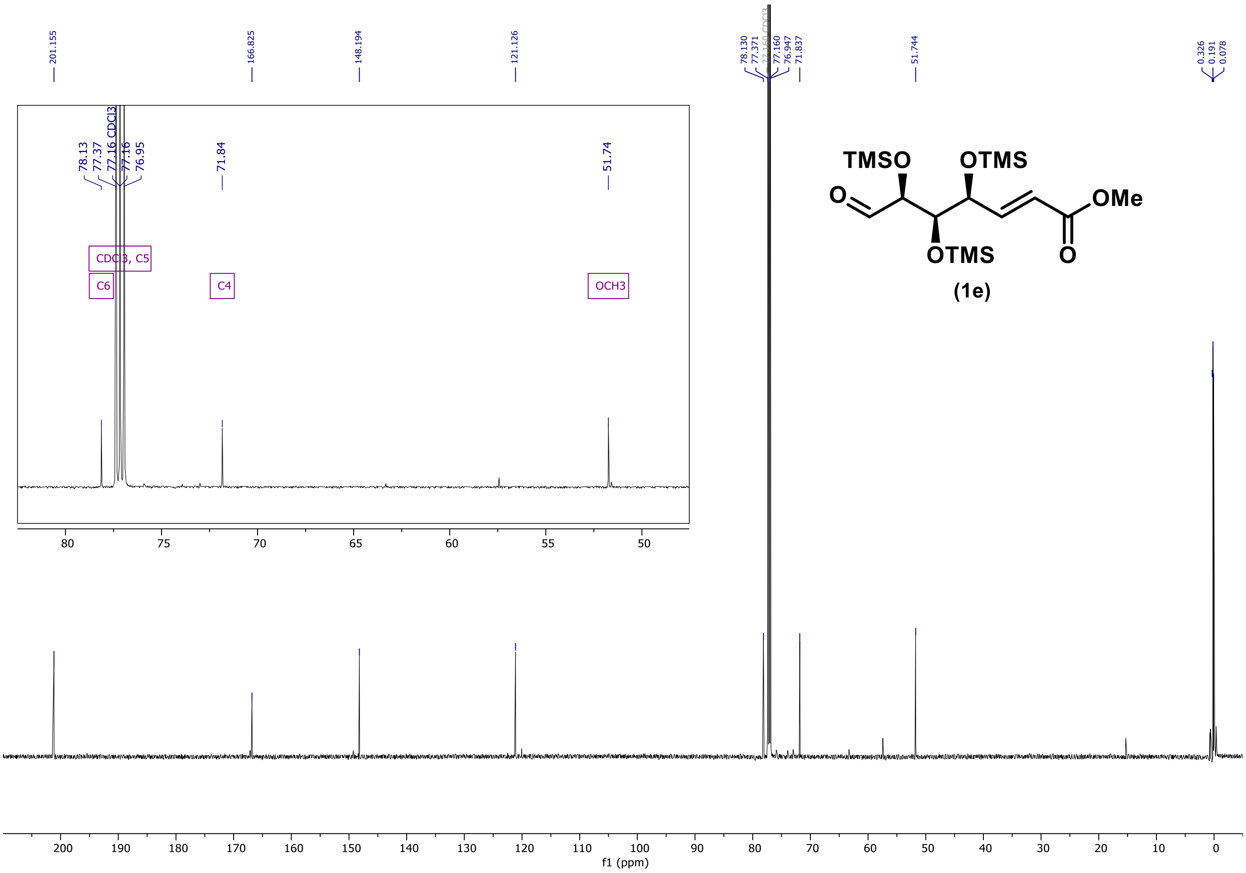

Figure S85. *151 MHz $^{13}C\{^{1}H\}$-NMR of (1e)*

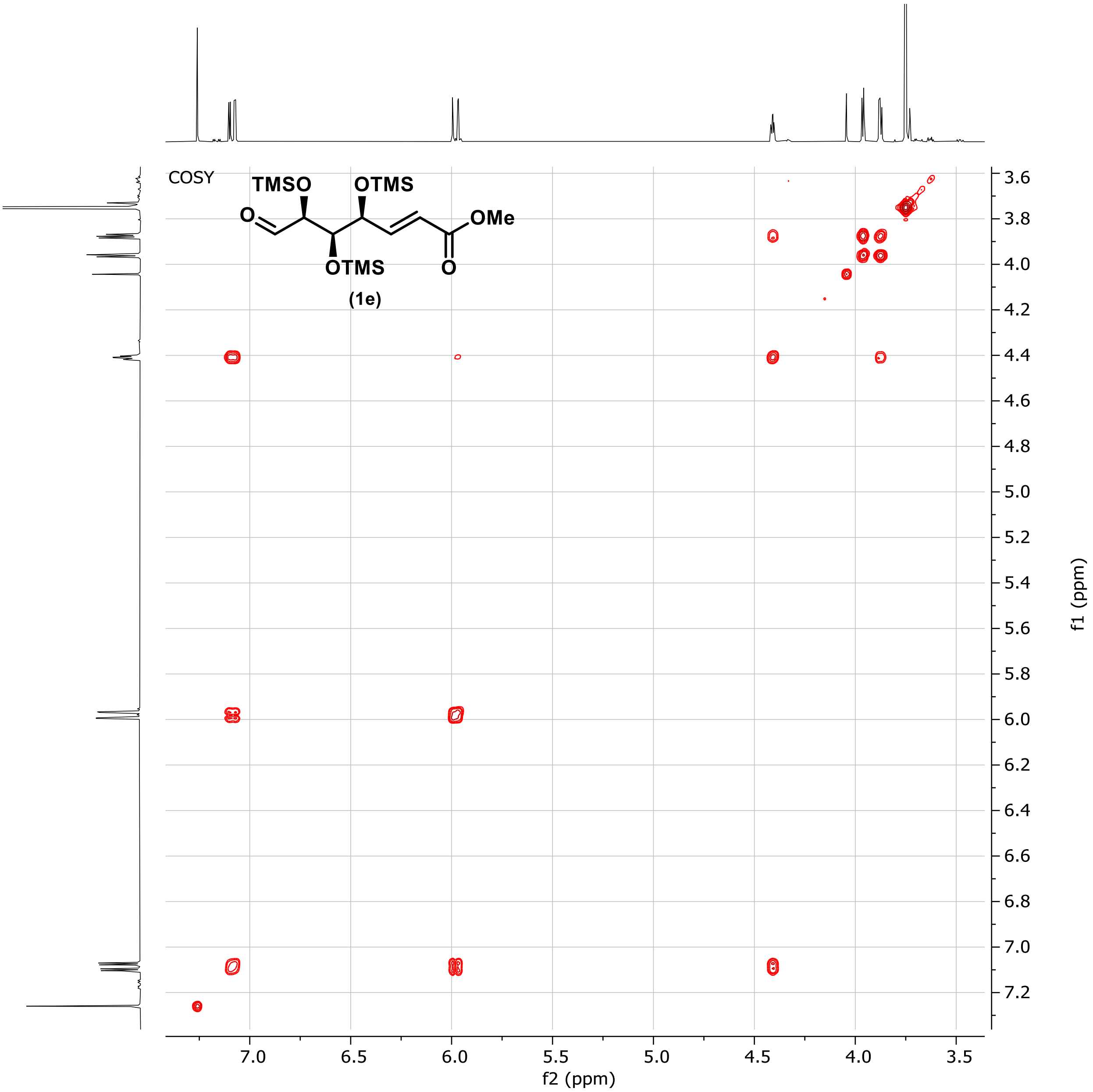

Figure S86. *COSY NMR of (1e)*

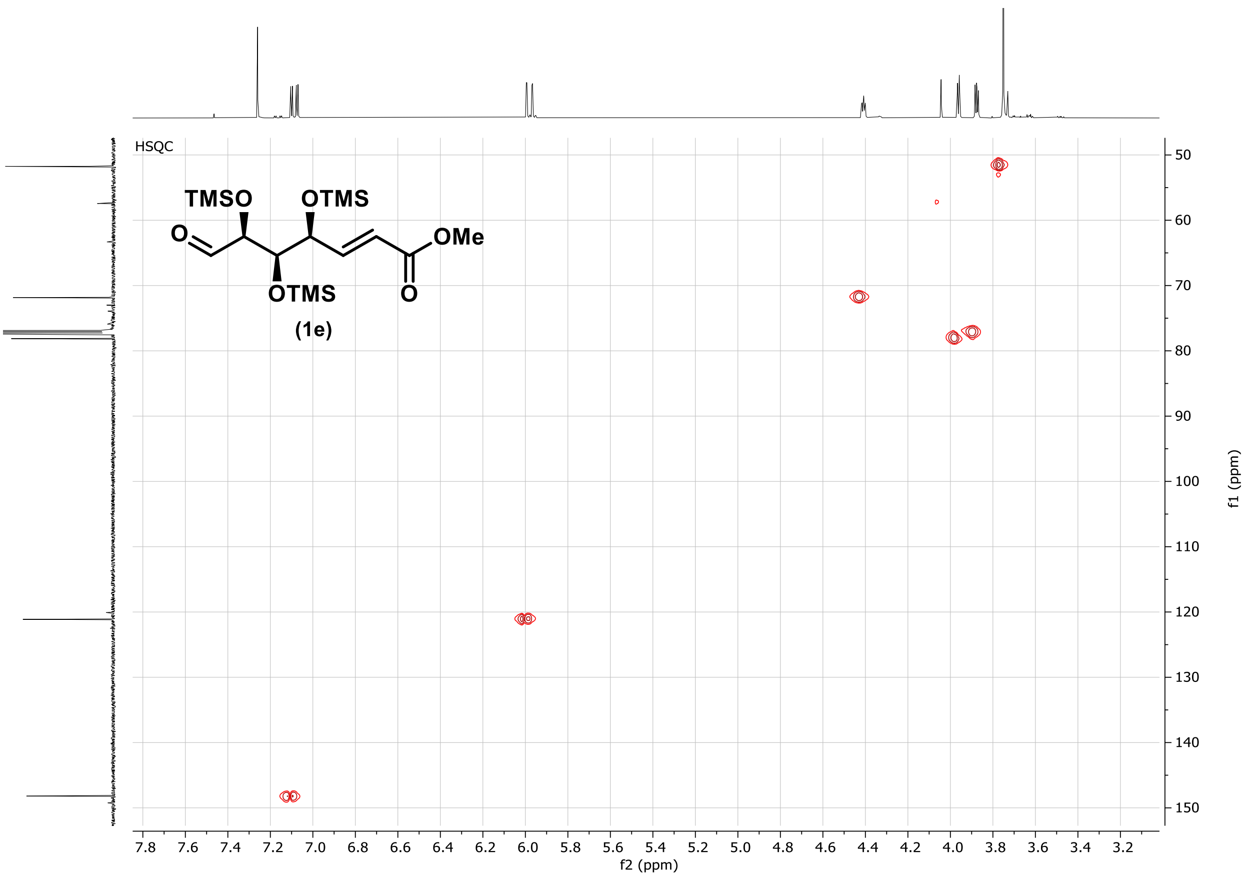

Figure S87. *HSQC NMR of (1e)*

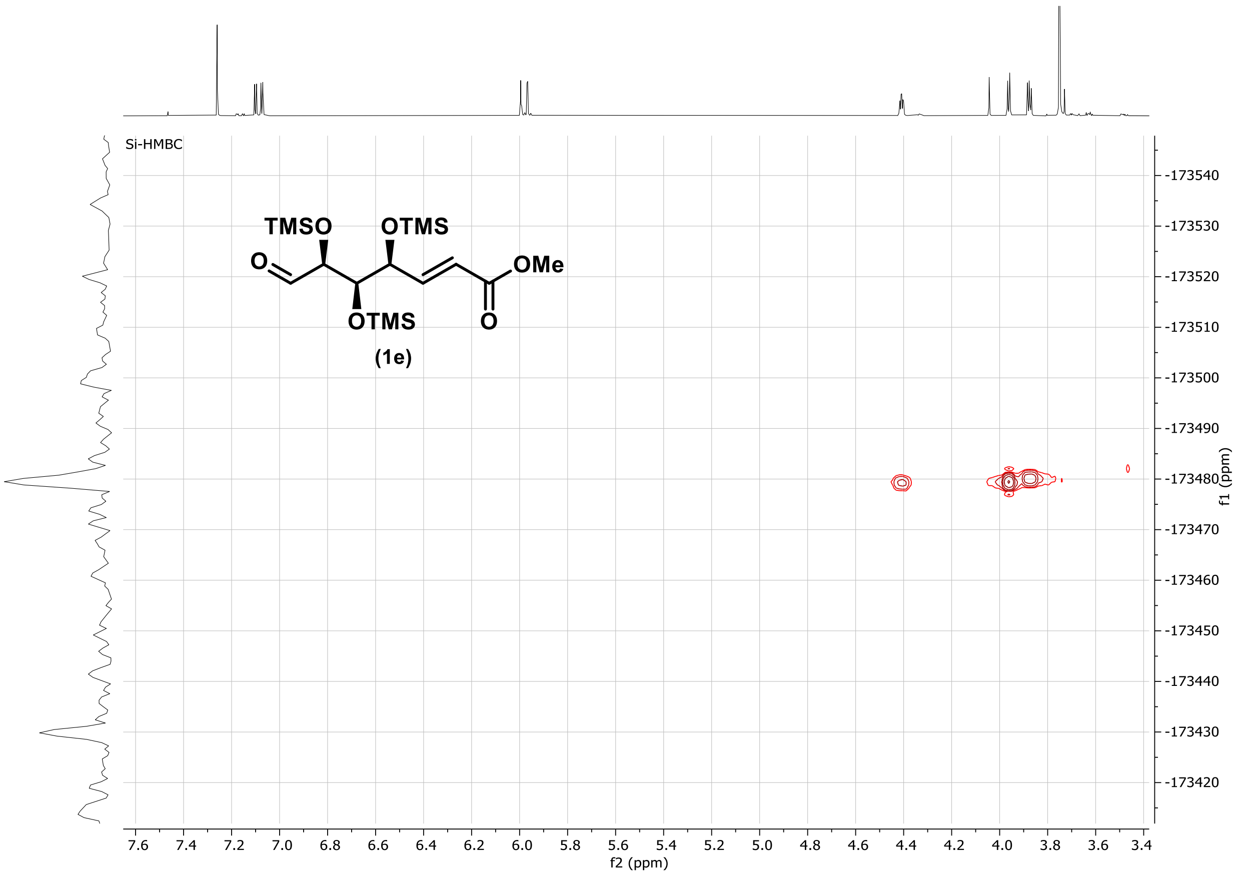

Figure S88. *Si-HMBC NMR of (1e) showing successful removal of one OTMS group*

D.2. Lyxose derived Compounds

D.2.1. Methyl D-lyxopyranoside (4b)

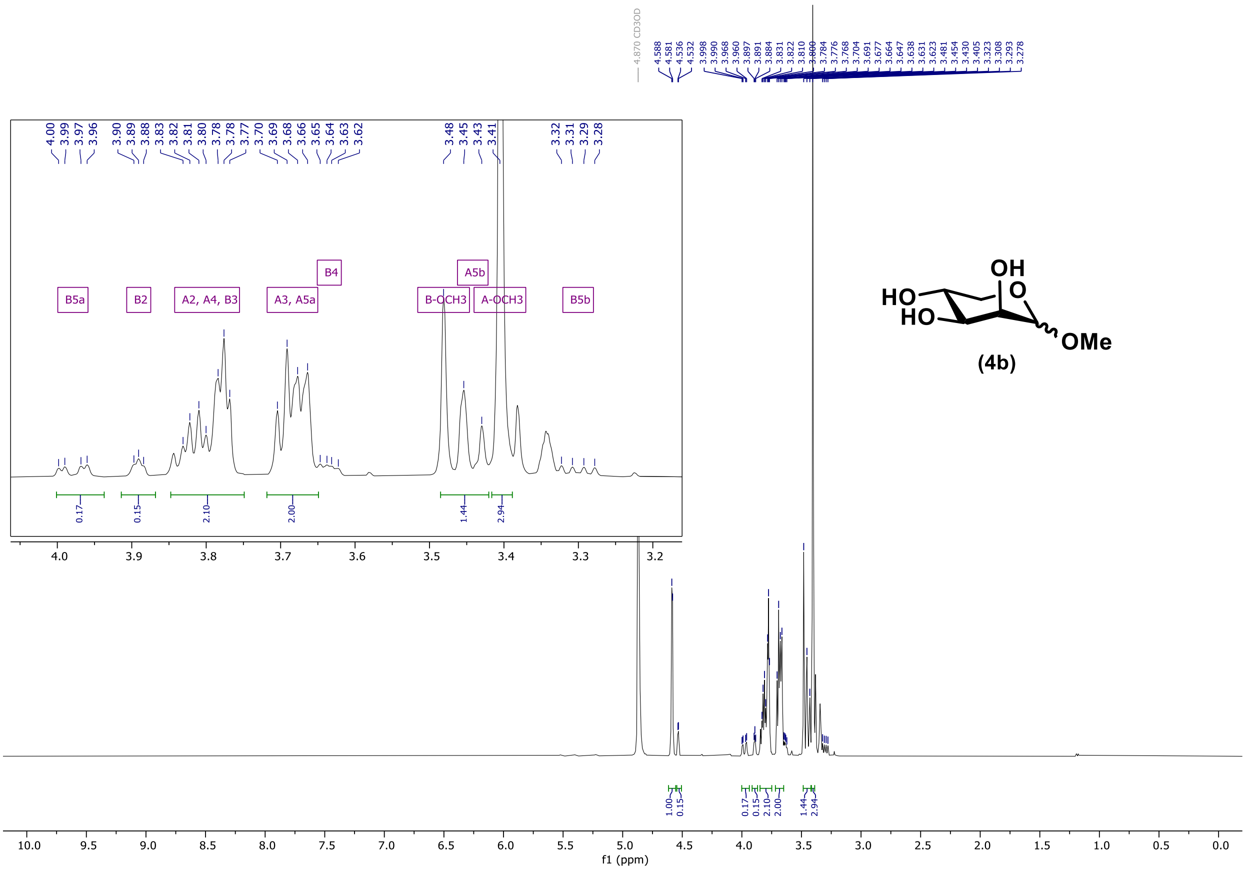

Figure S89. *400 MHz ^{1}H-NMR of (4b)*

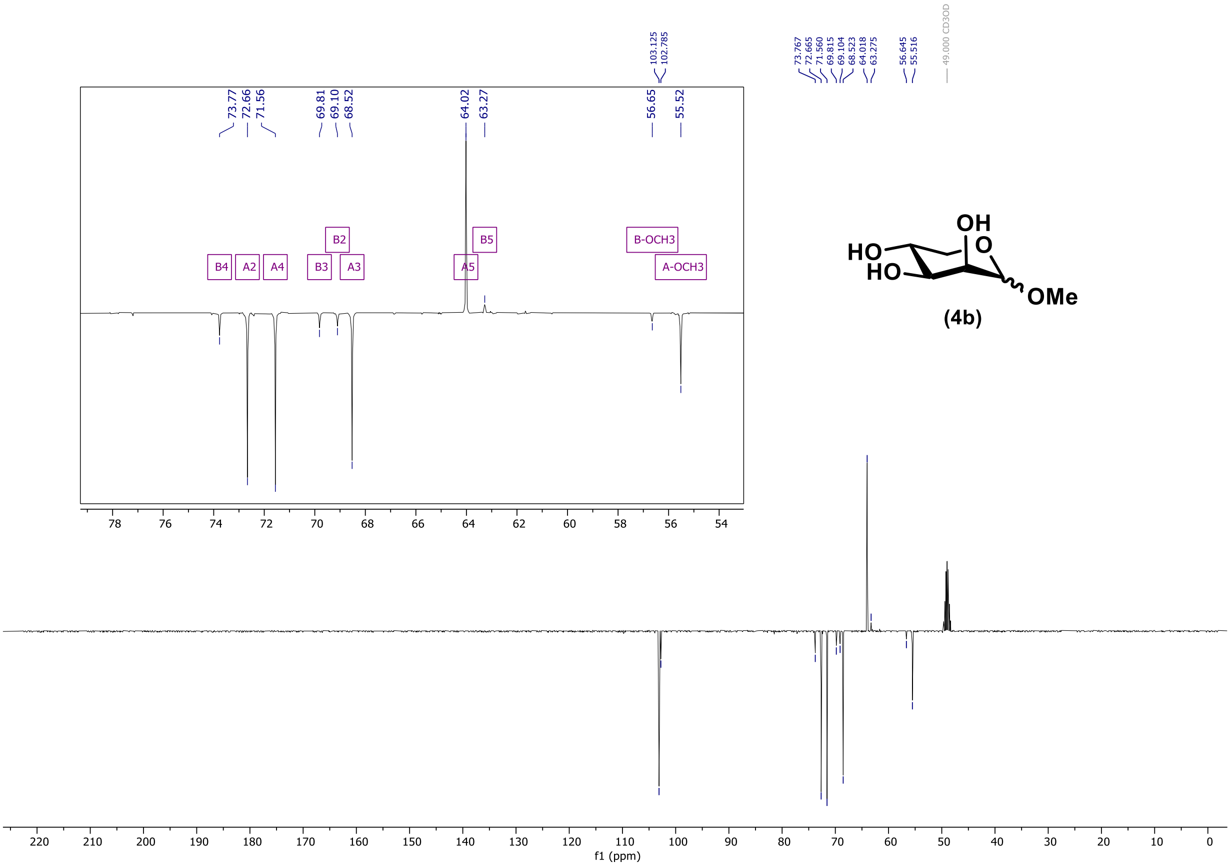

Figure S90. *101 MHz $^{13}C\{^1H\}$-NMR of (4b)*

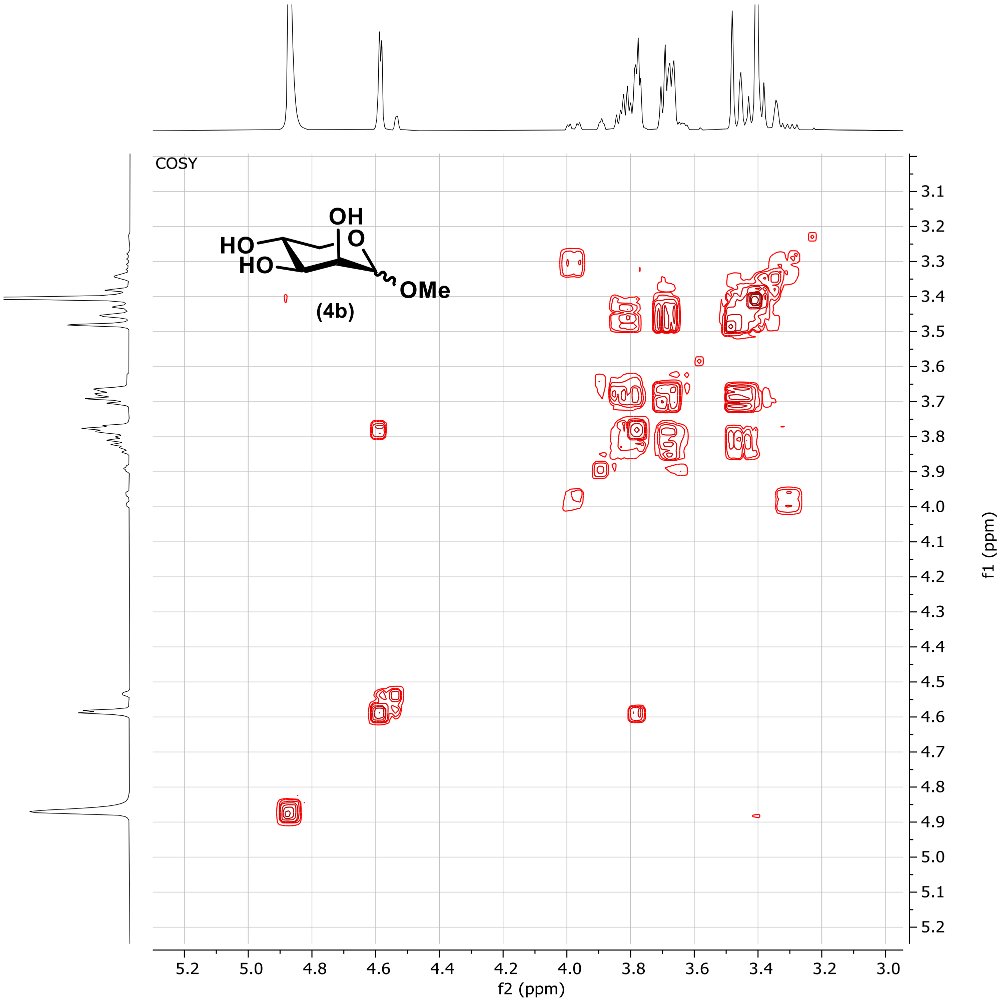

Figure S91. COSY NMR of (4b)

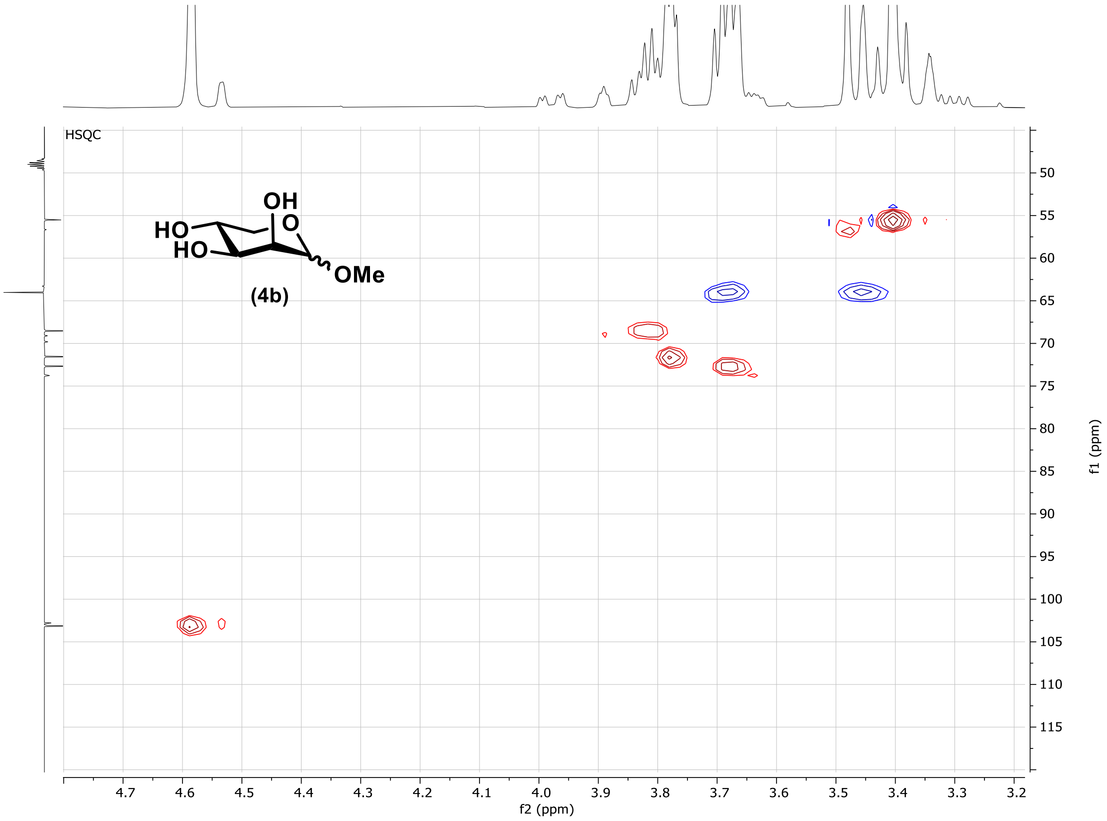

Figure S92. HSQC NMR of (4b)

D.2.2. Methyl 2,3,4-Tri-*O*-benzyl-α-D-lyxopyranoside (5b)

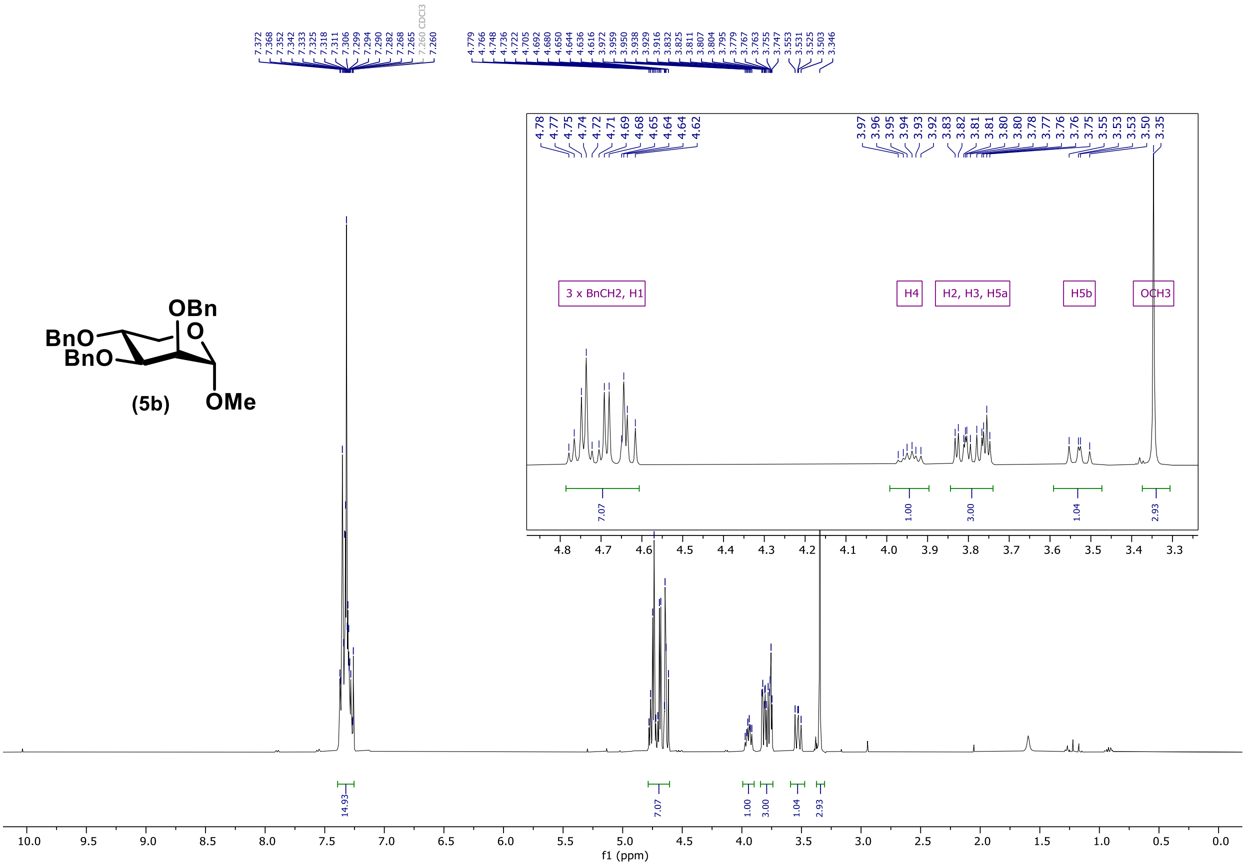

Figure S93. *400 MHz ^{1}H-NMR of (5b)*

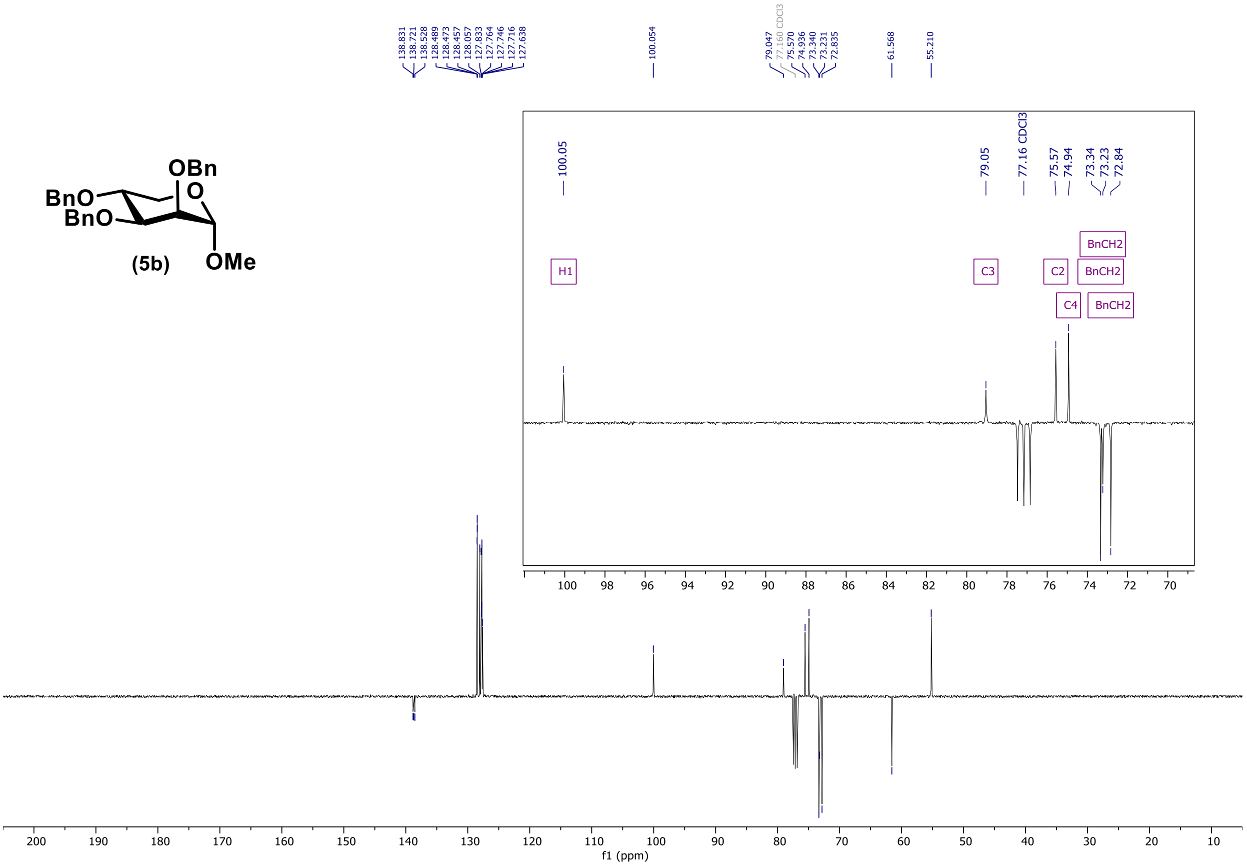

Figure S94. *101 MHz $^{13}C\{^1H\}$-NMR of (5b)*

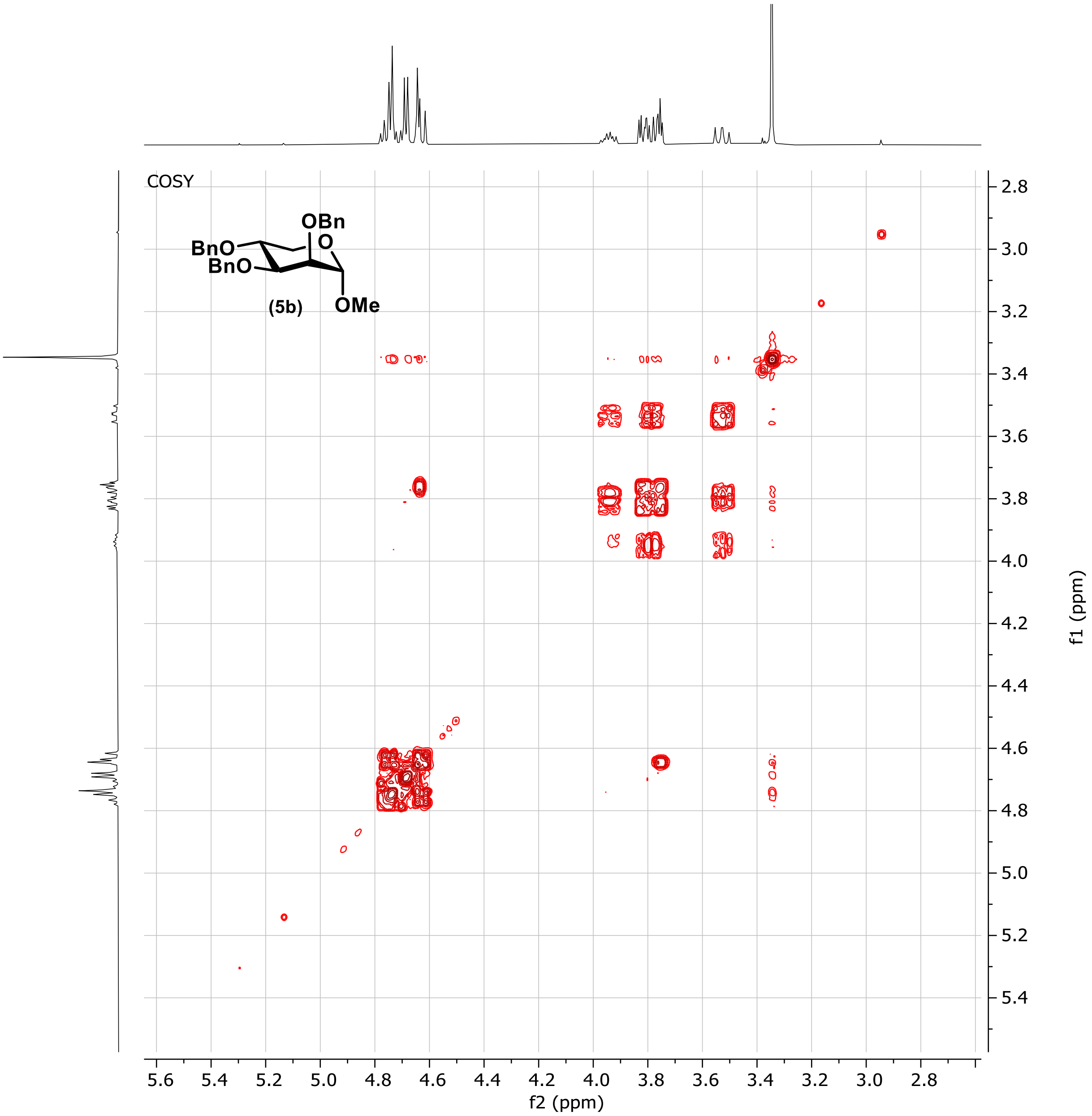

Figure S95. *COSY NMR of (5b)*

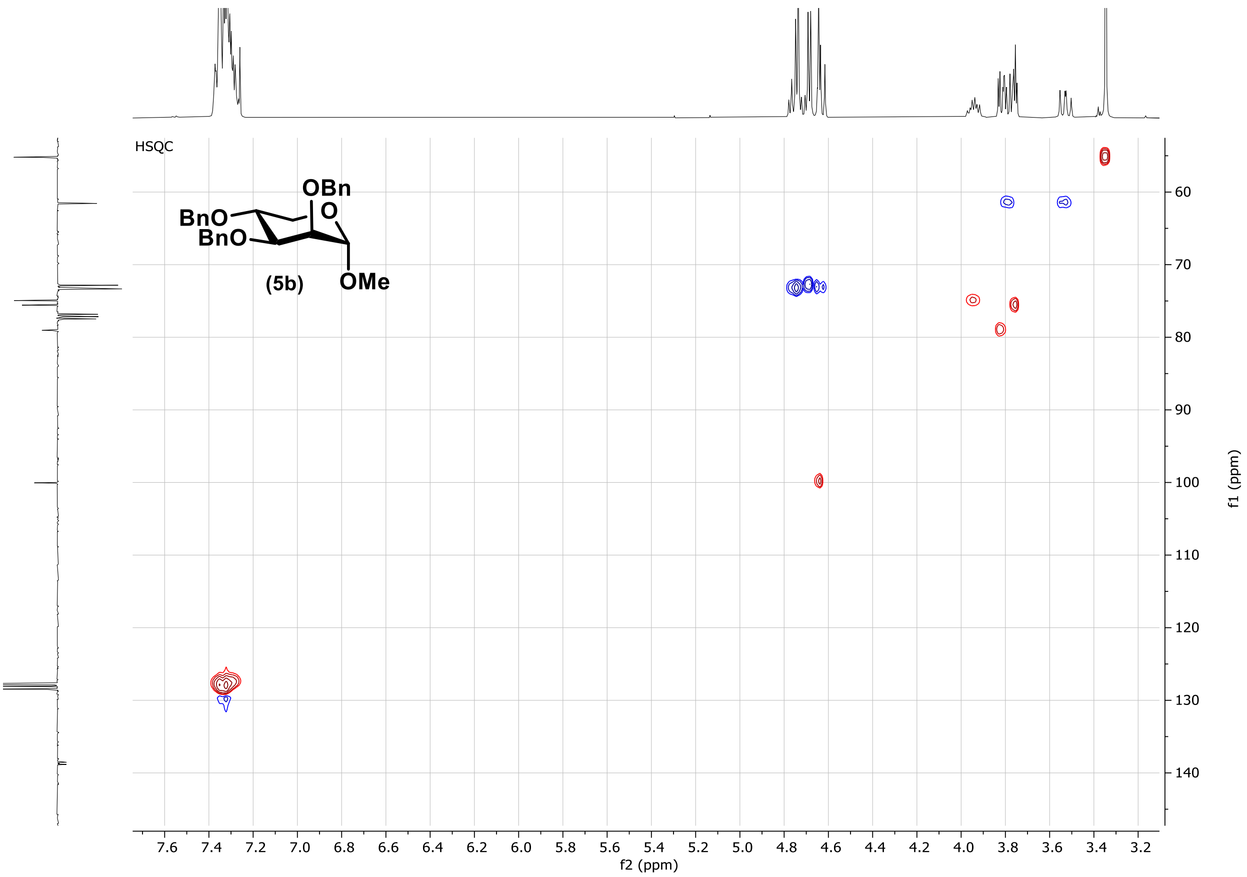

Figure S96. *HSQC NMR of (5b)*

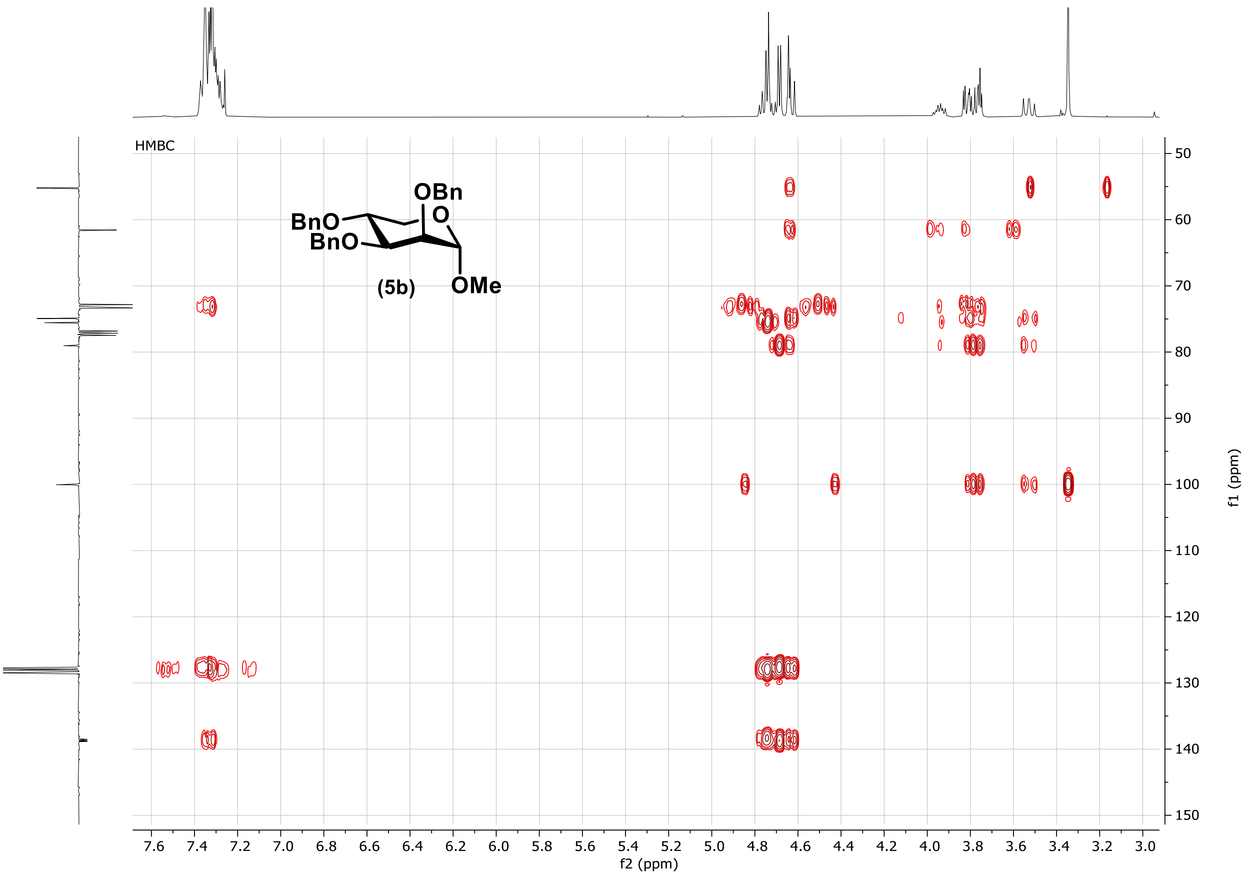

Figure S97. *HMBC NMR of (5b)*

D.2.3. 2,3,4-Tri-*O*-benzyl-D-lyxopyranose (6b)

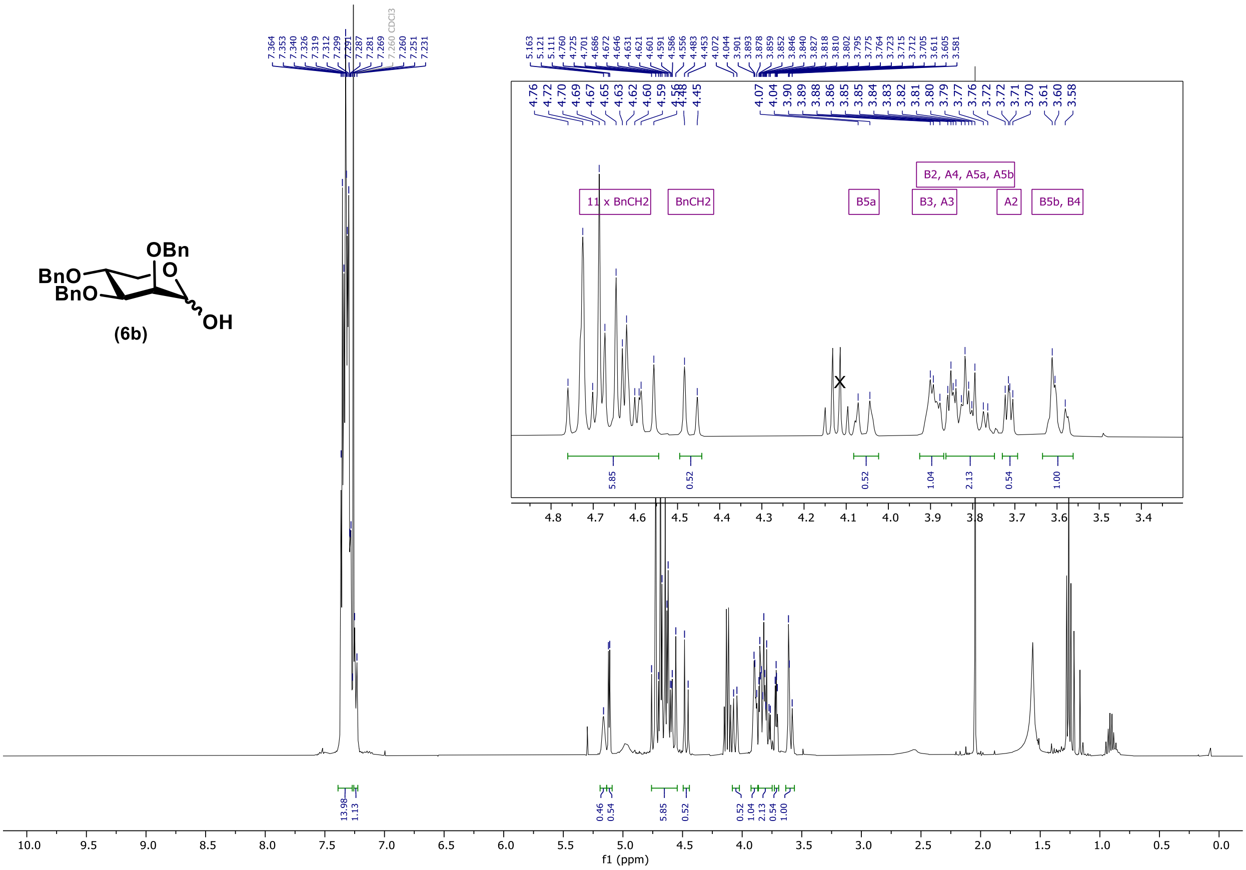

Figure S98. *600 MHz ^{1}H-NMR of (6b)*

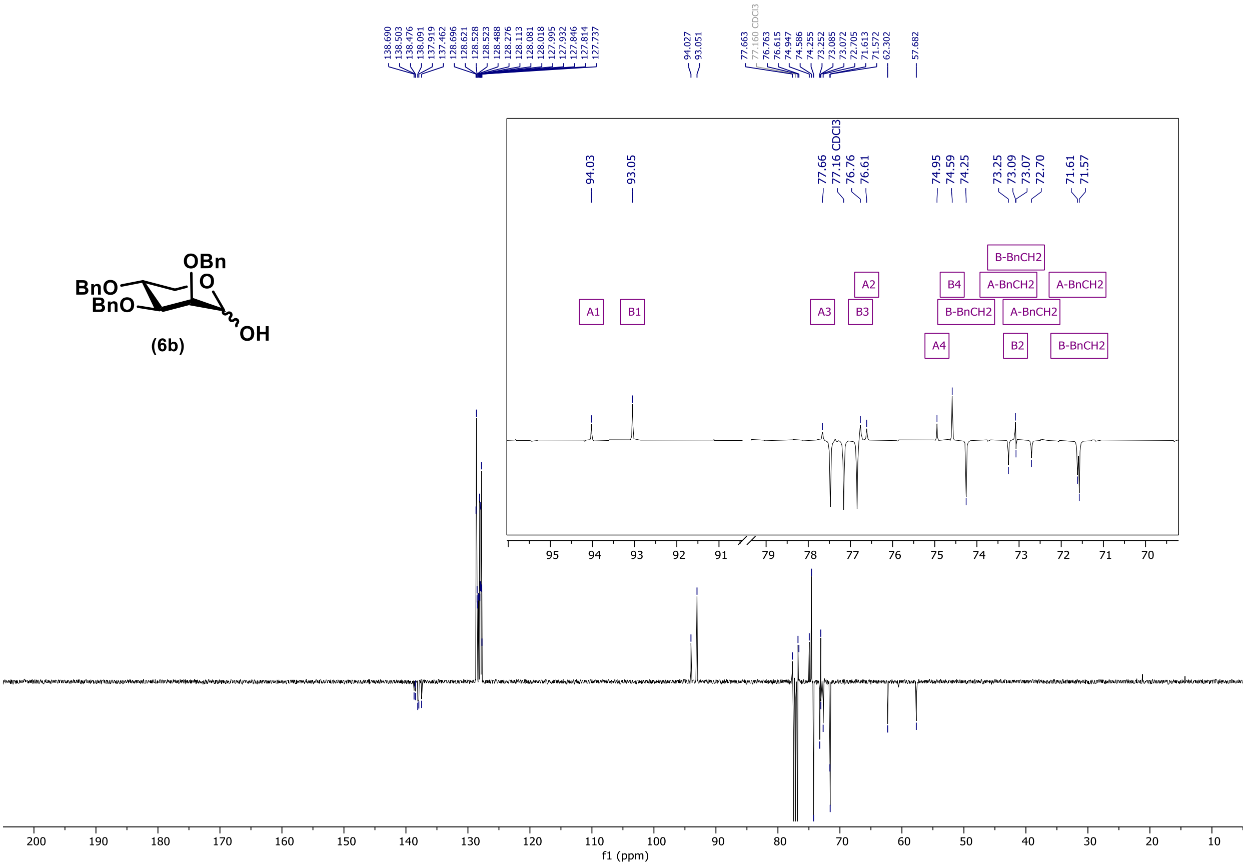

Figure S99. *151 MHz ^{13}C{^{1}H}-NMR of (6b)*

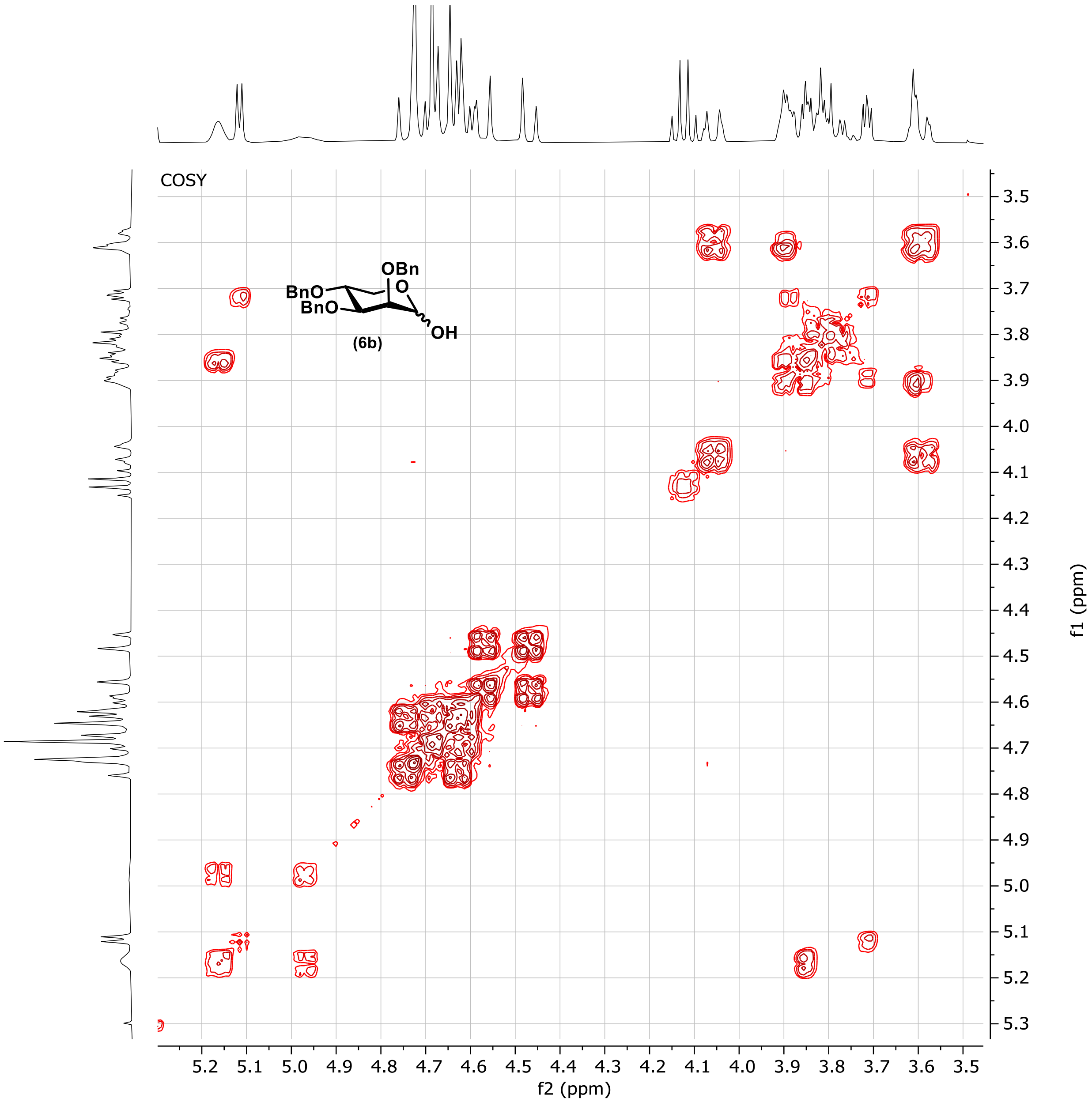

Figure S100. *COSY NMR of (6b)*

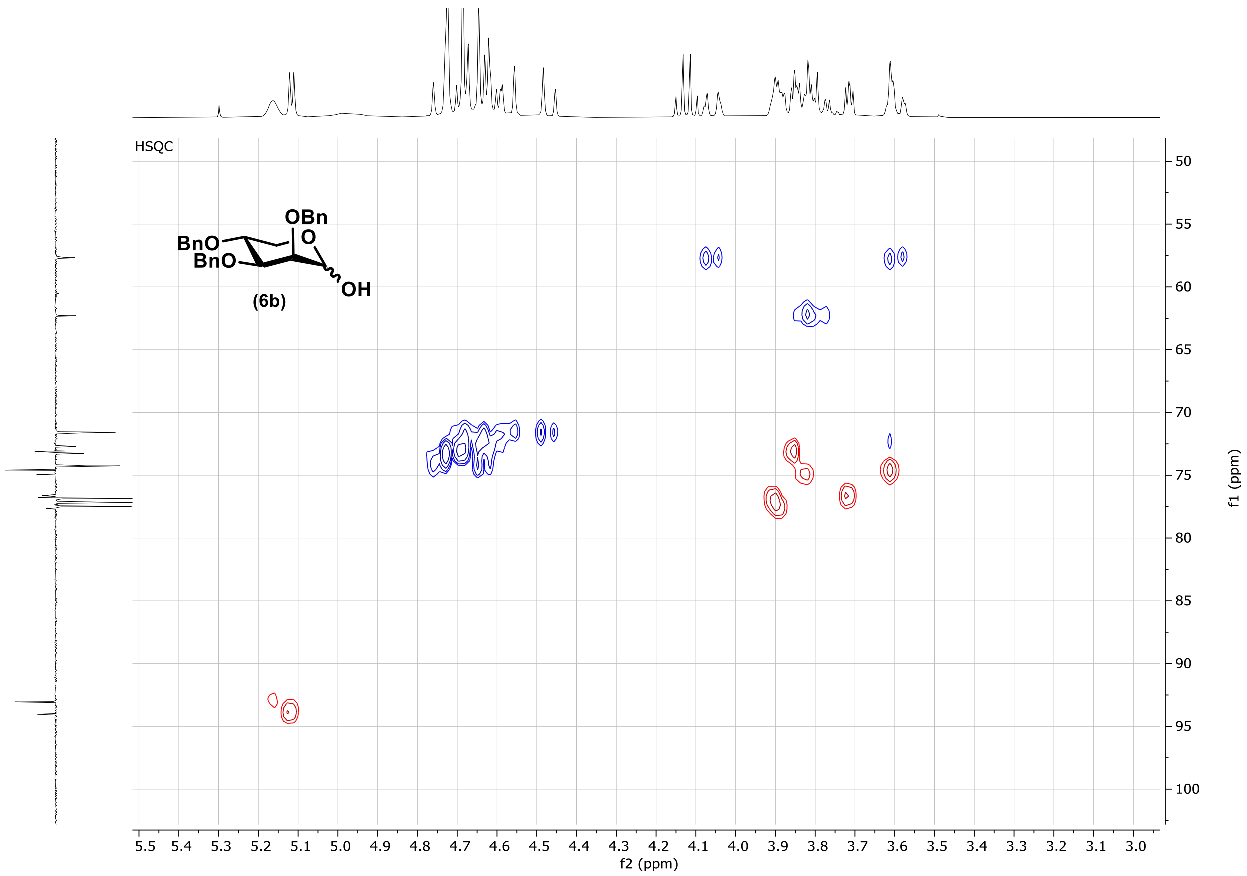

Figure S101. *HSQC NMR of (6b)*

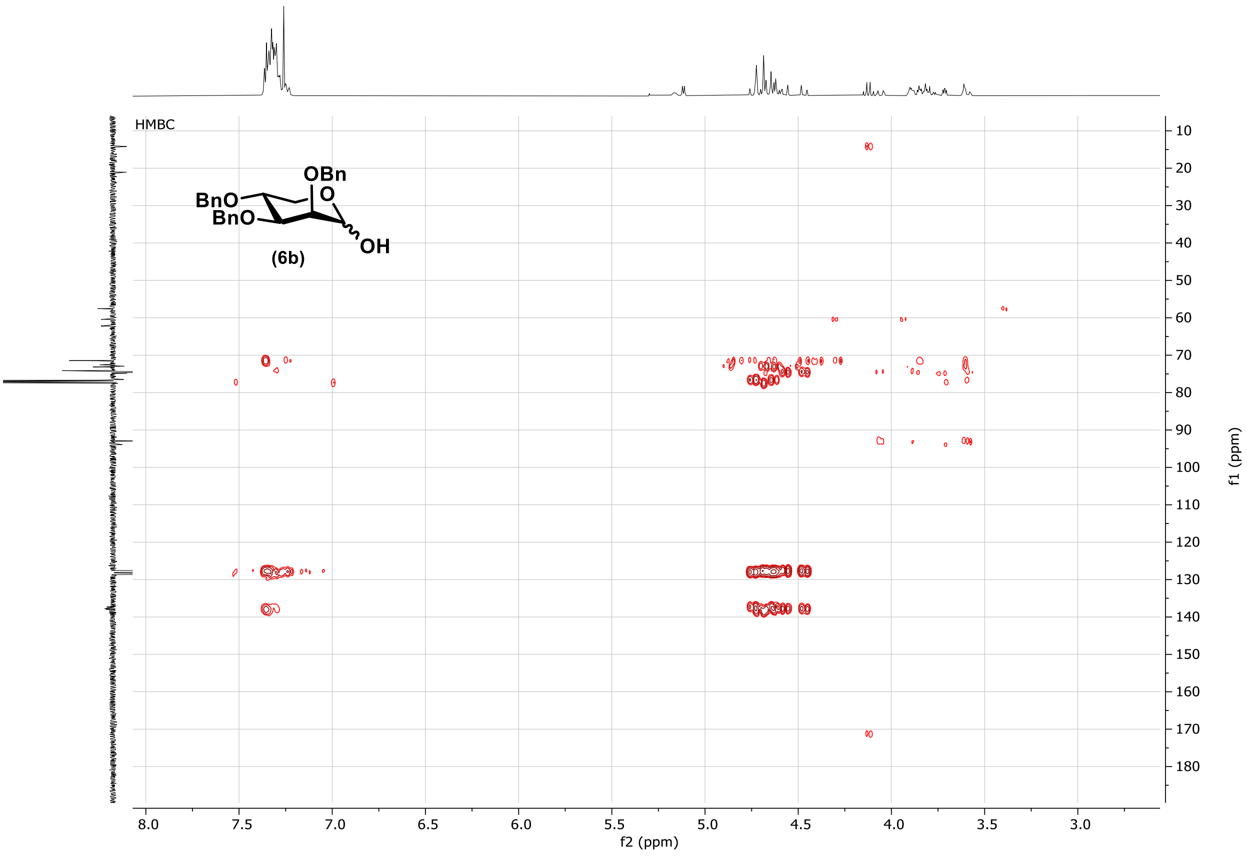

Figure S102. *HMBC NMR of (6b)*

D.2.4. Methyl (4R,5R,6R, E)-4,5,6-tris(benzyloxy)-7-hydroxyhept-2-enoate (7b-e)

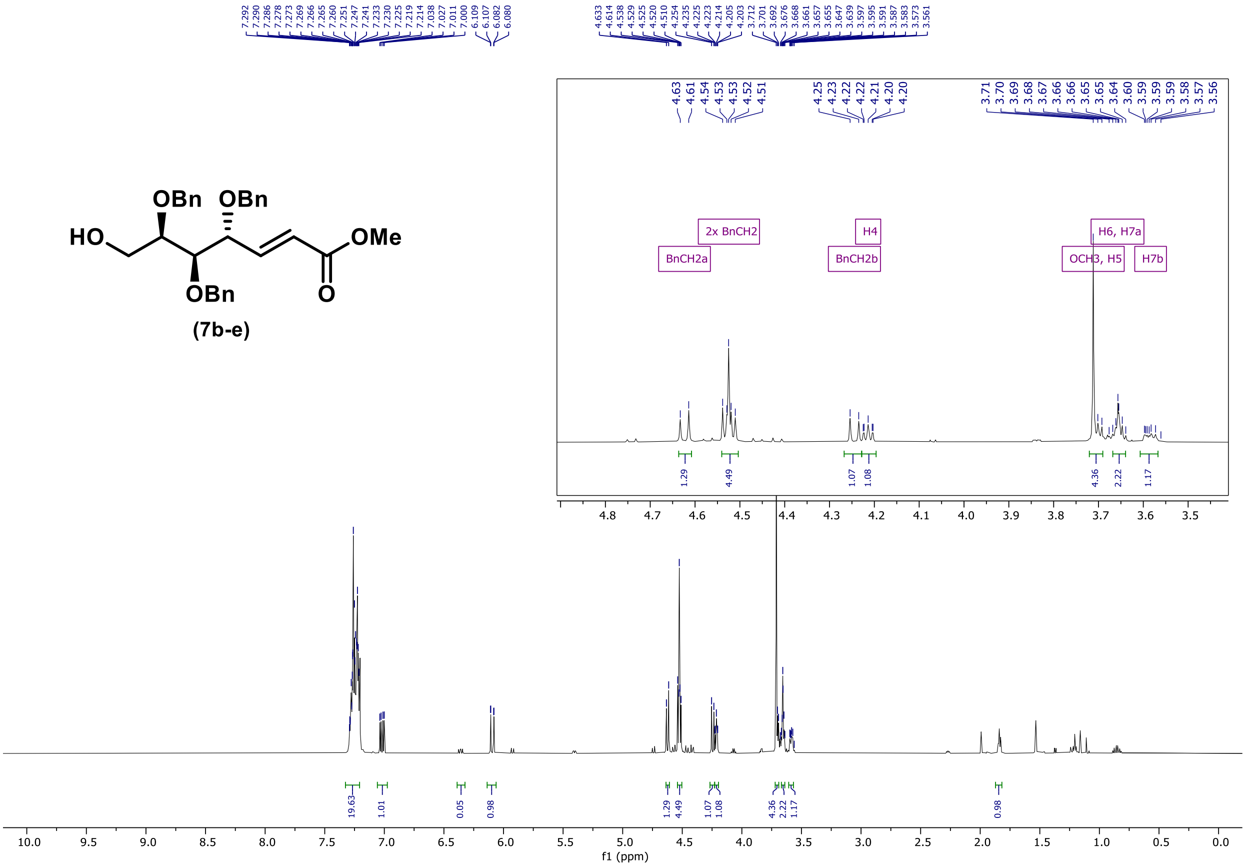

Figure S103. *600 MHz ¹H-NMR of (7b-e)*

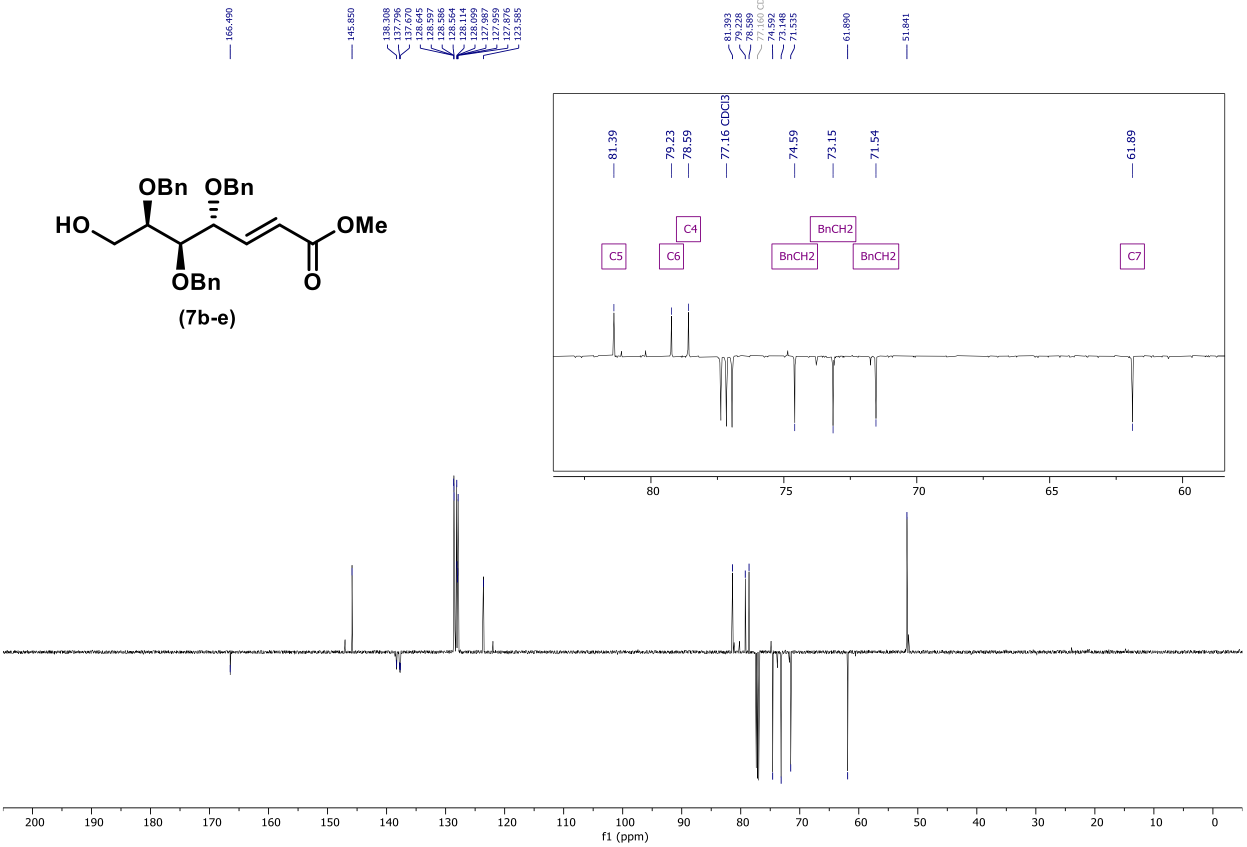

Figure S104. *151 MHz $^{13}C\{^{1}H\}$-NMR of (7b-e)*

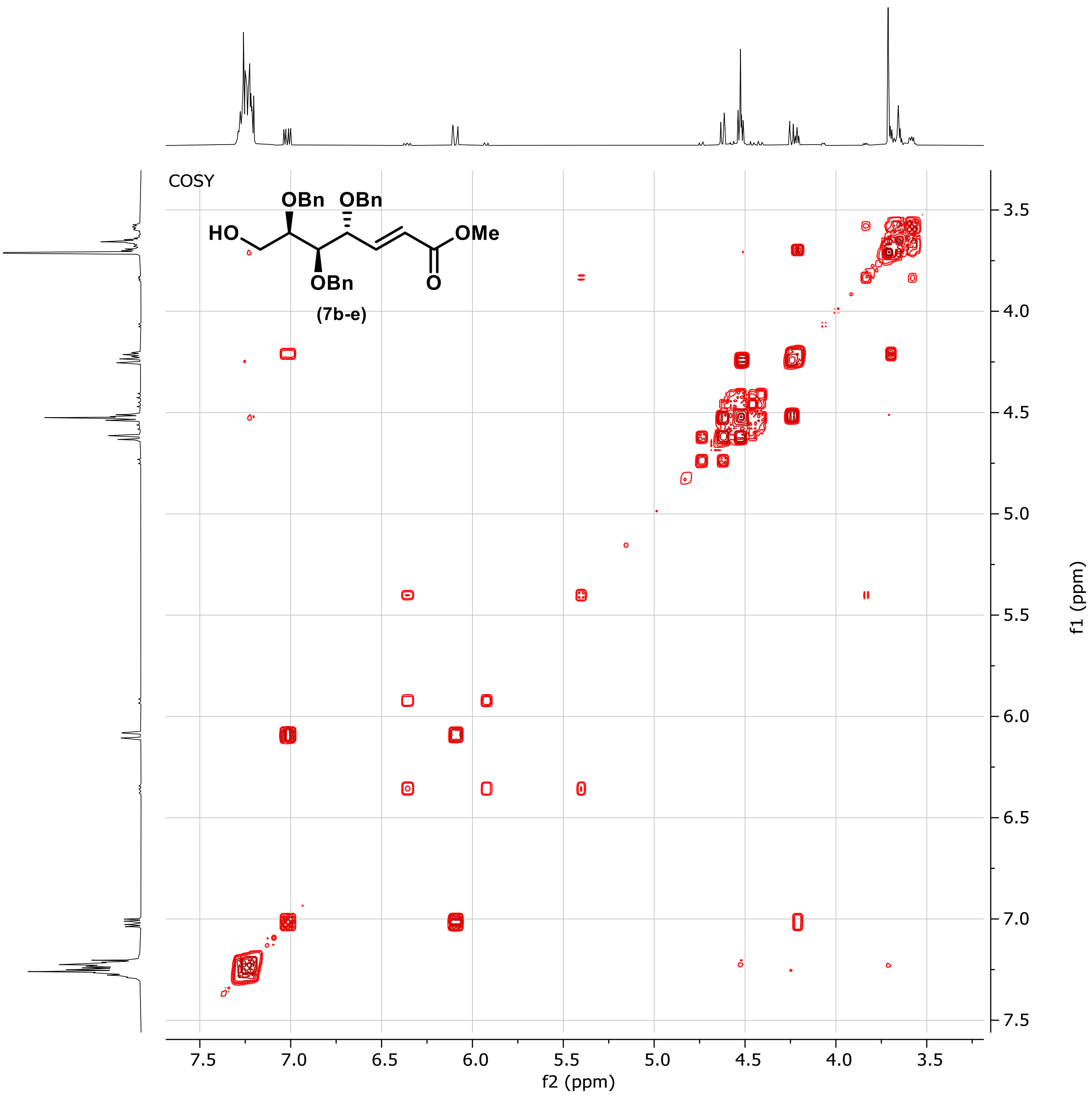

Figure S105. *COSY NMR of (7b-e)*

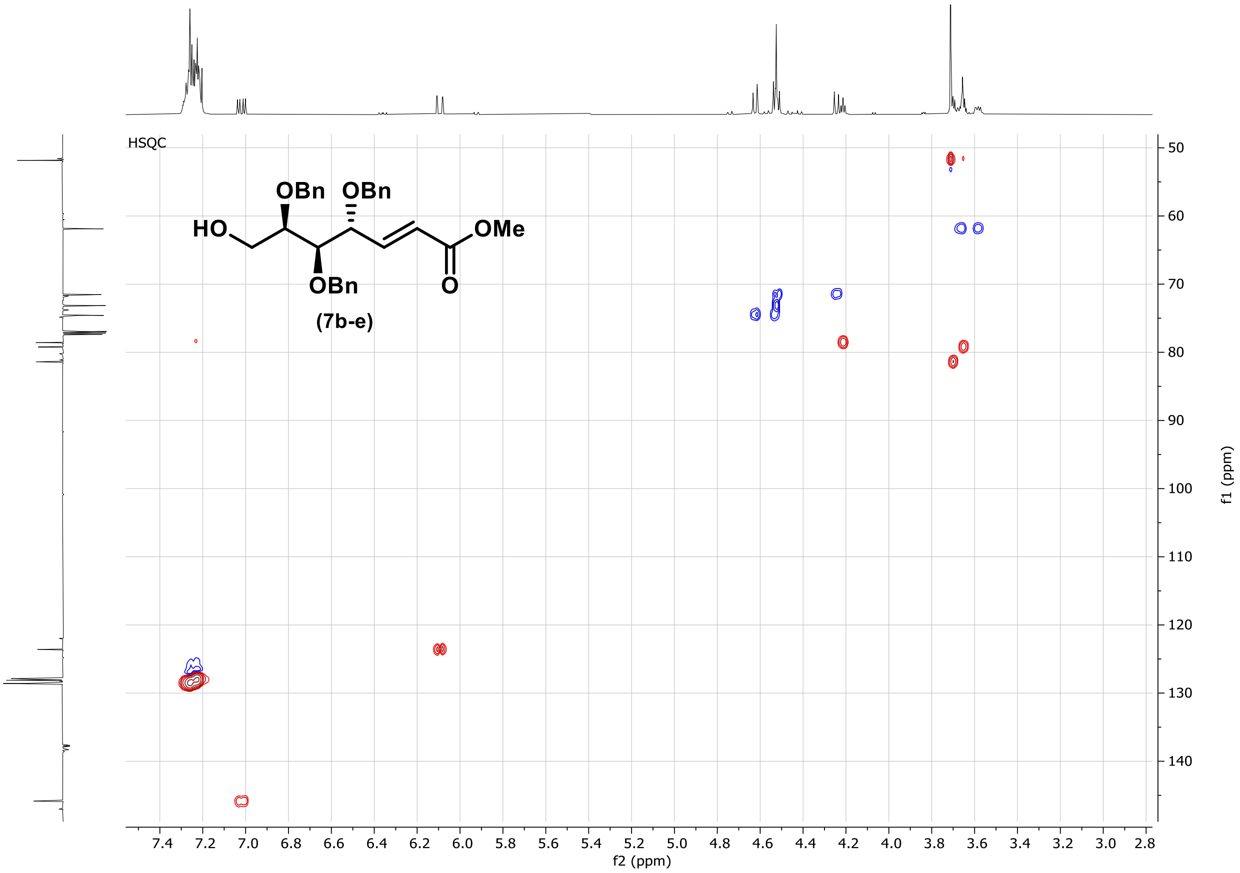

Figure S106. HSQC NMR of (7b-e)

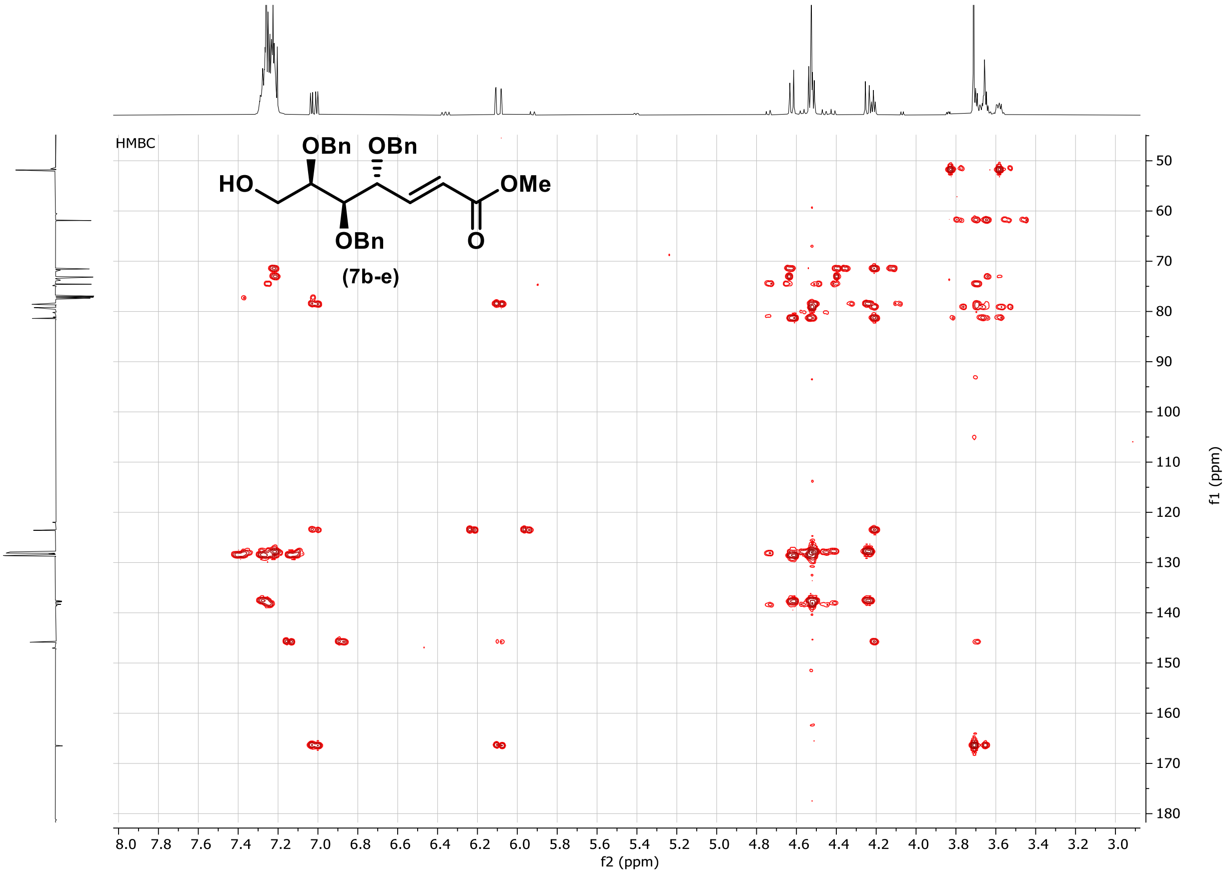

Figure S107. HMBC NMR of (7b-e)

D.2.5. Methyl (4R,5R,6R, Z)-4,5,6-tris(benzyloxy)-7-hydroxyhept-2-enoate (7b-z)

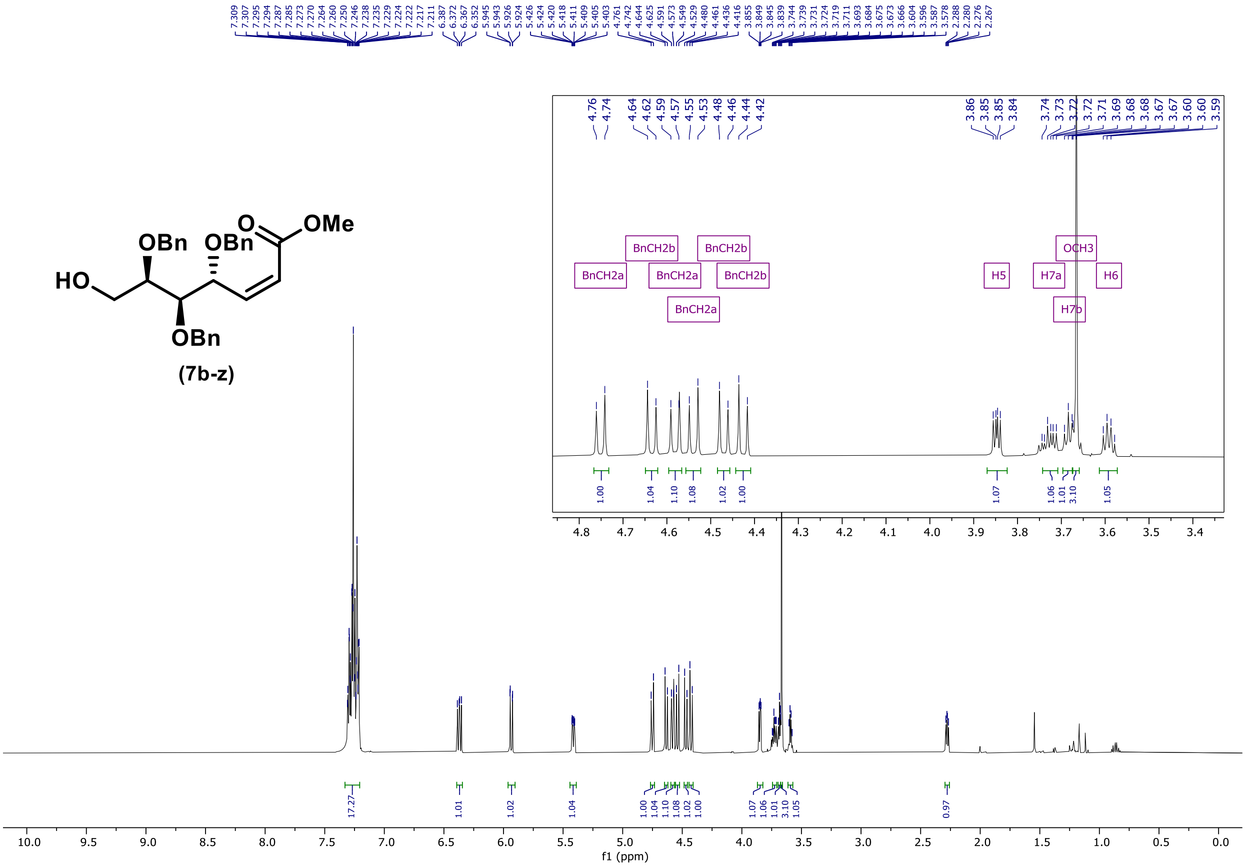

Figure S108. *400 MHz ^{1}H-NMR of (7b-z)*

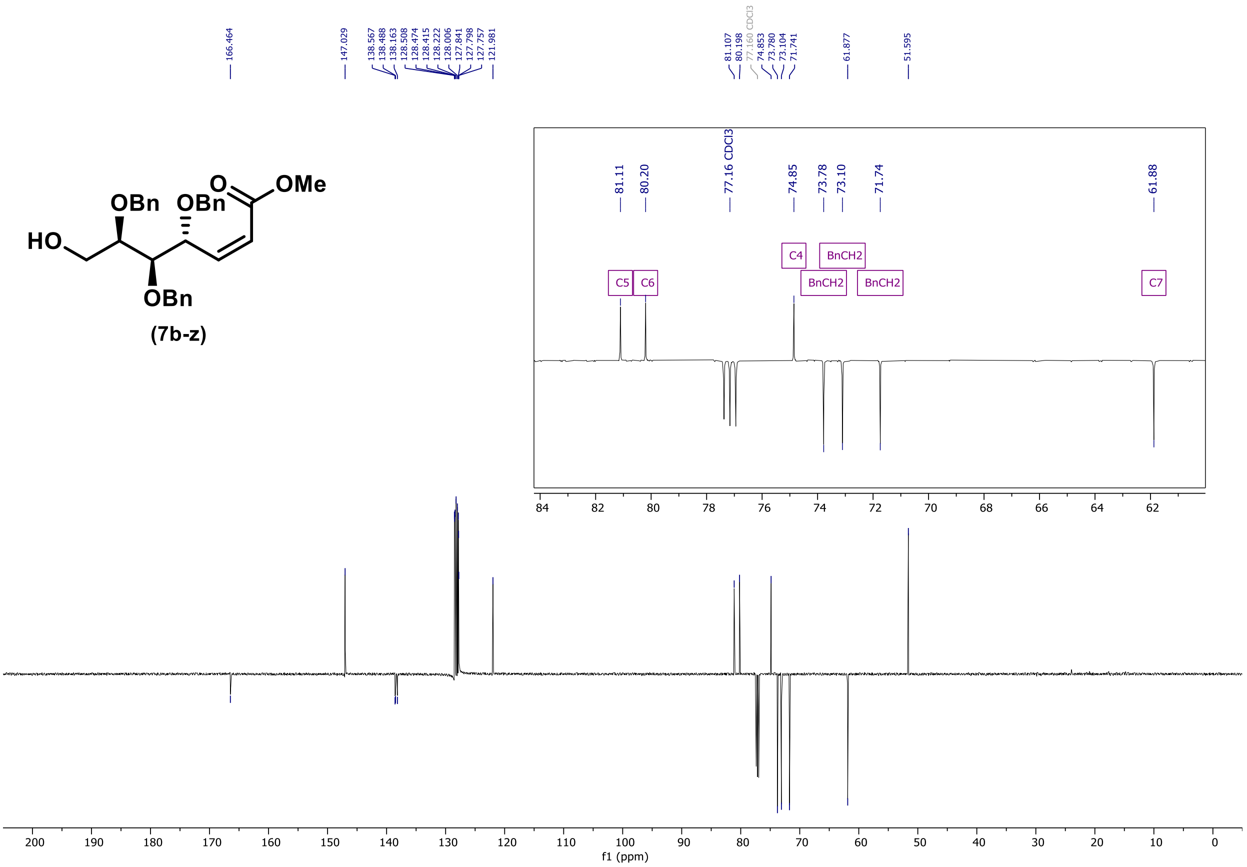

Figure S109. *101 MHz $^{13}C\{^1H\}$-NMR of (7b-z)*

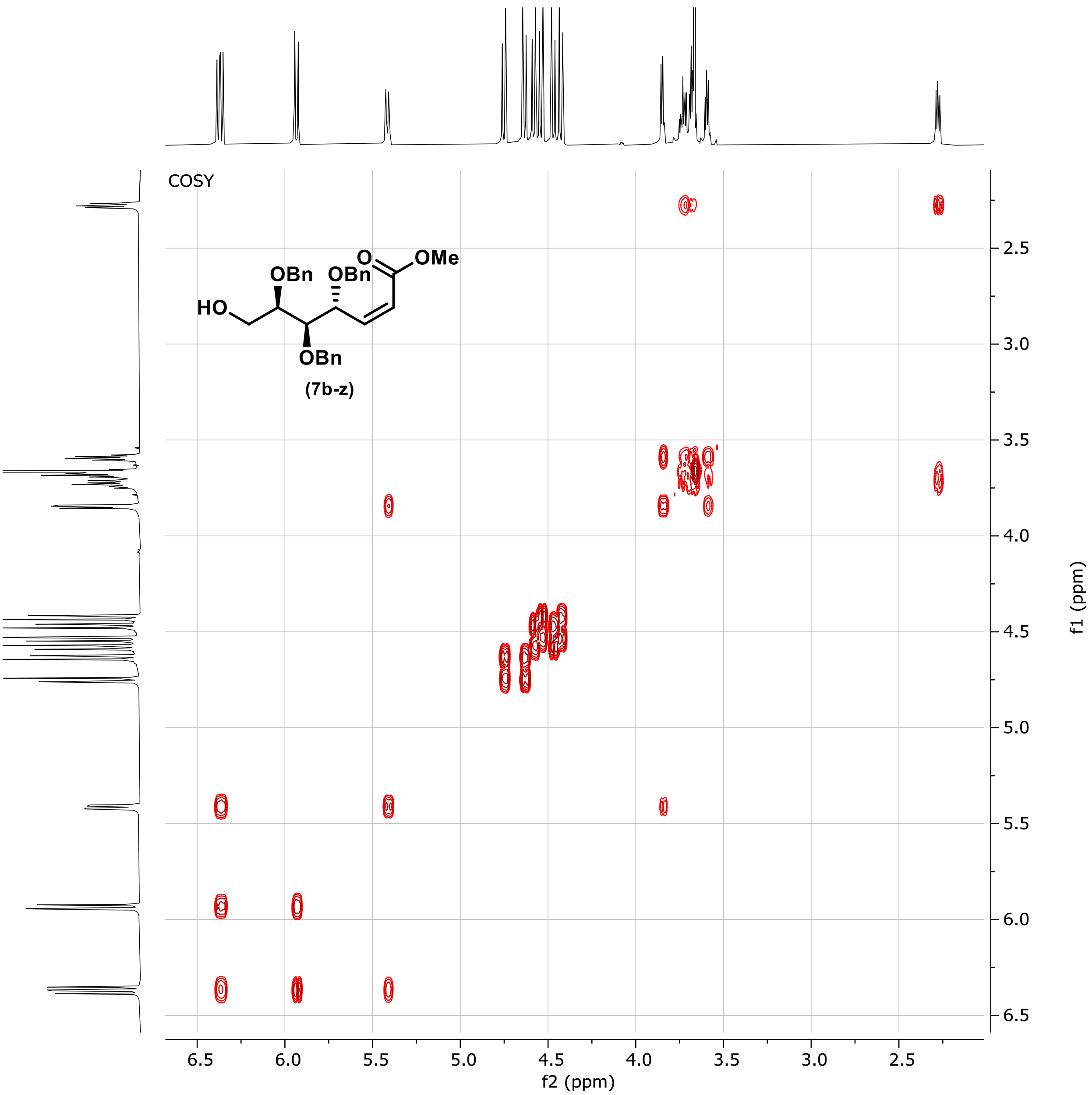

Figure S110. COSY NMR of (7b-z)

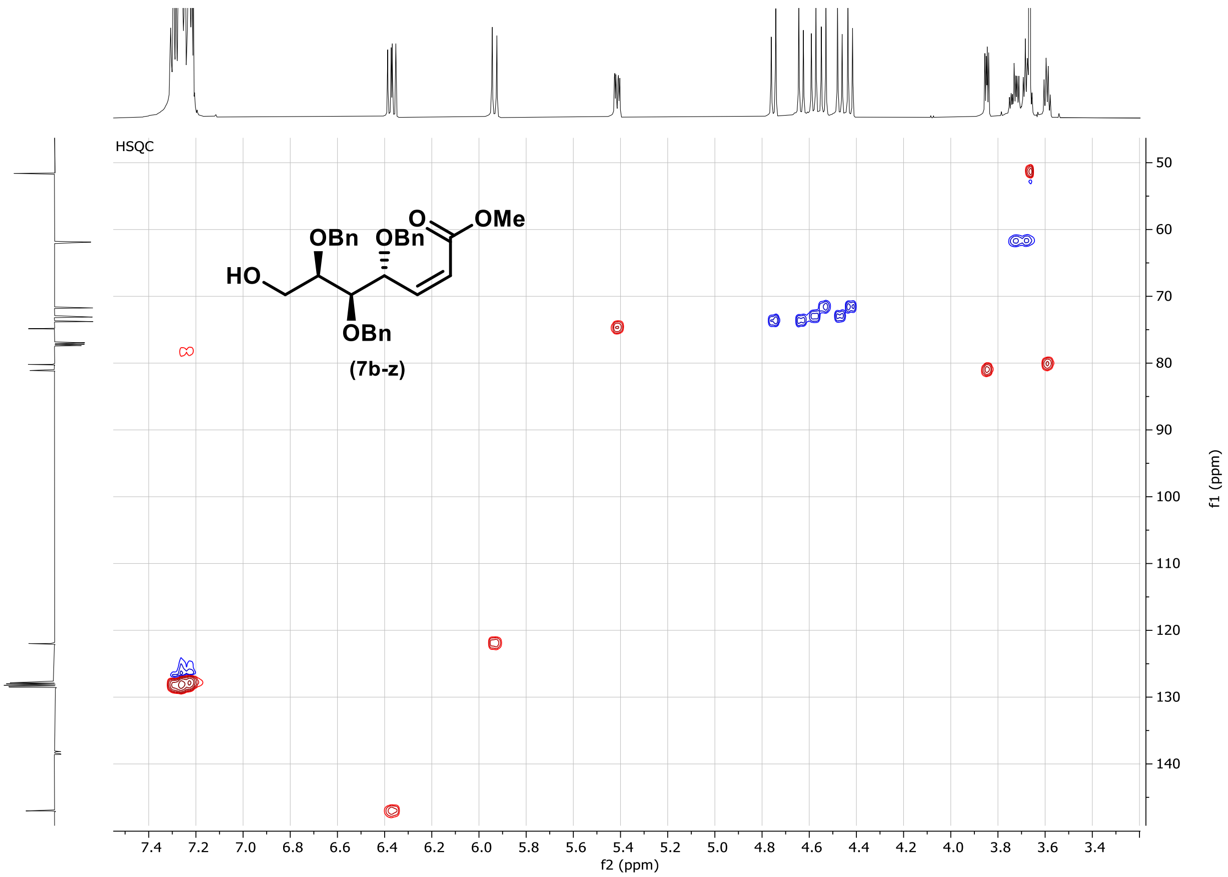

Figure S111. *HSQC NMR of (7b-z)*

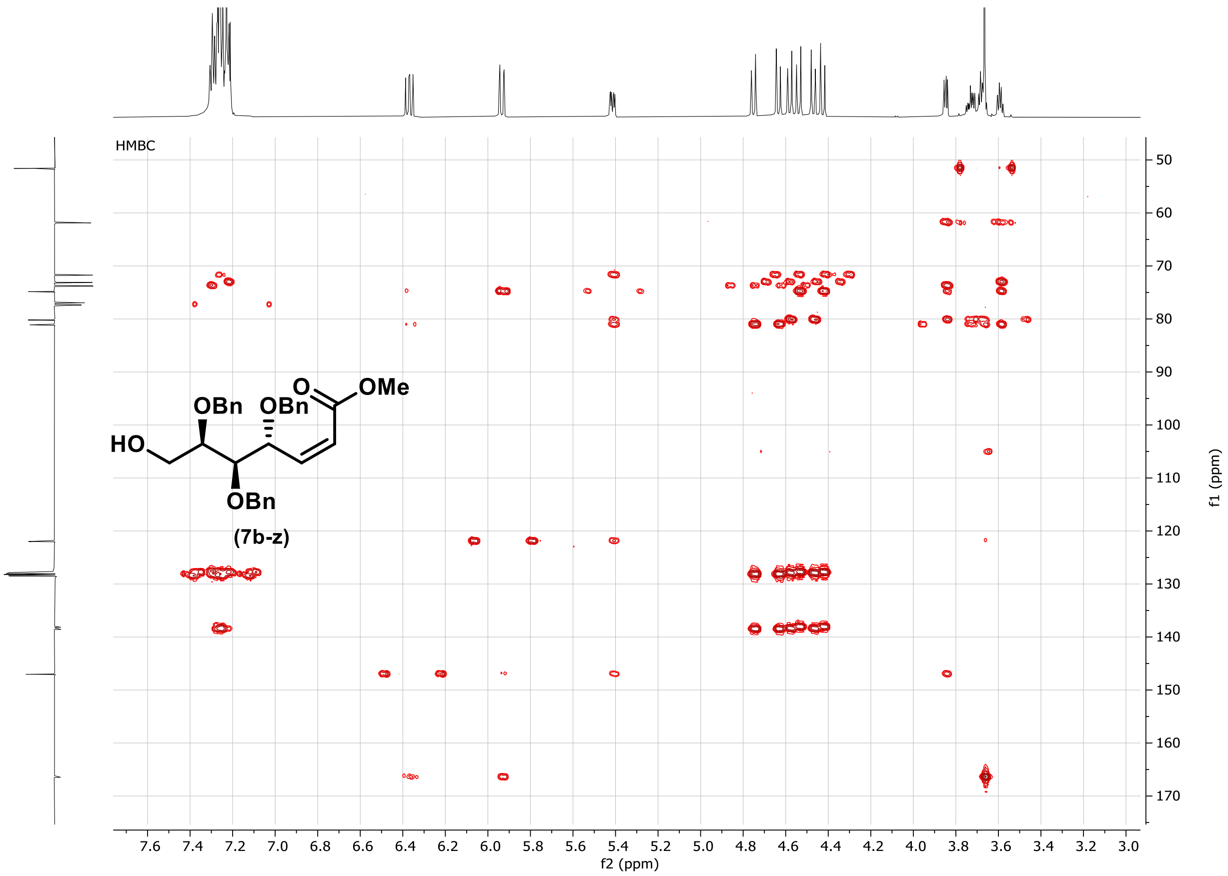

Figure S112. *HMBC NMR of (7b-z)*

D.2.6. Methyl (4R,5R,6R, Z)-4,5,6-tris(benzyloxy)-7-oxo-hept-2-enoate (1b)

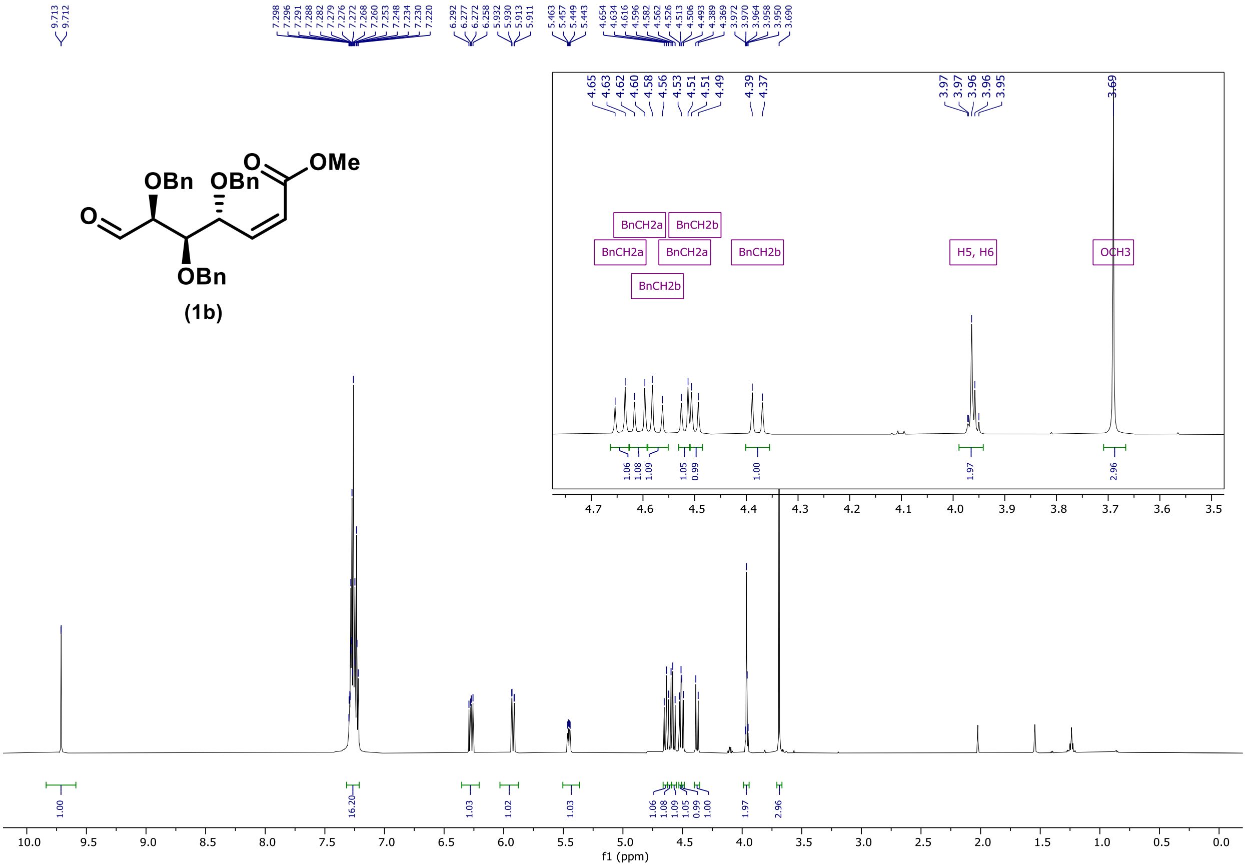

Figure S113. *400 MHz ^{1}H-NMR of (1b)*

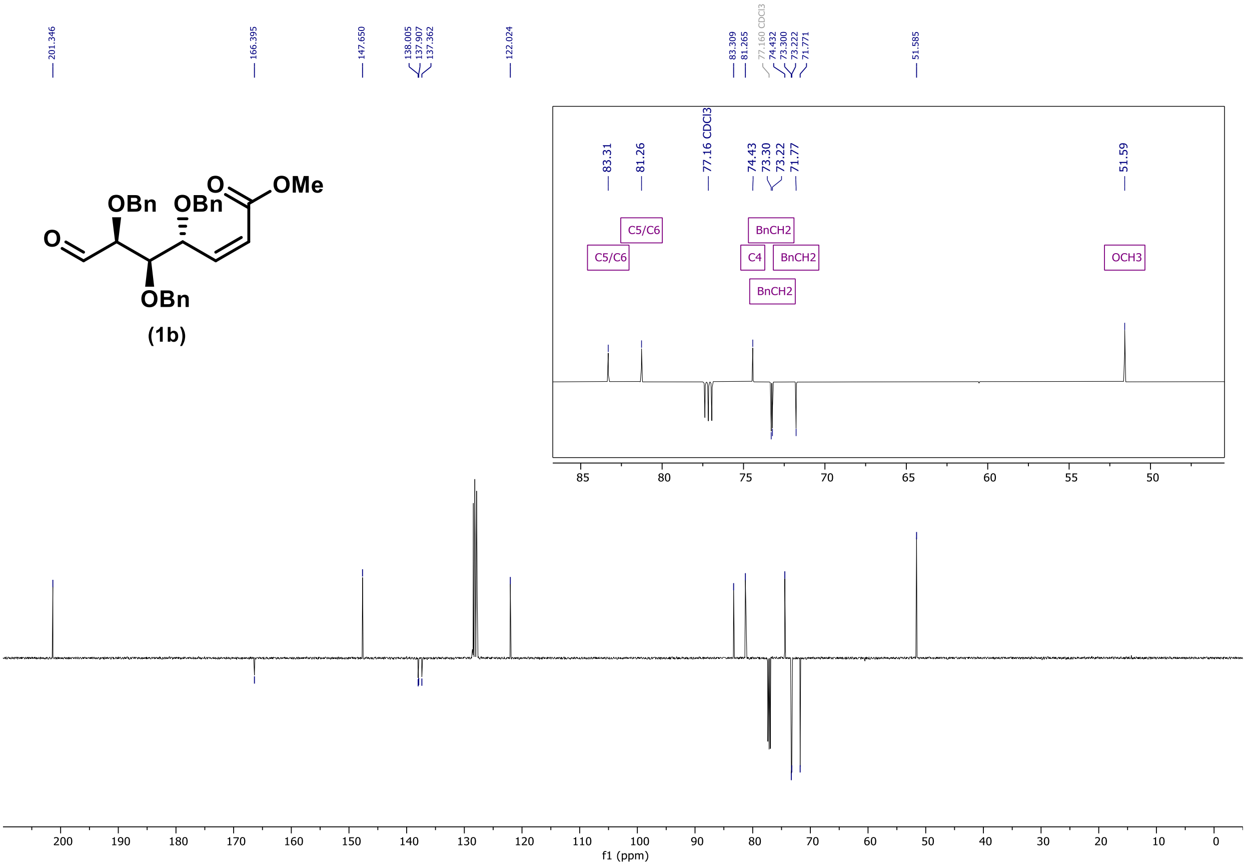

Figure S114. *101 MHz $^{13}C\{^1H\}$-NMR of (1b)*

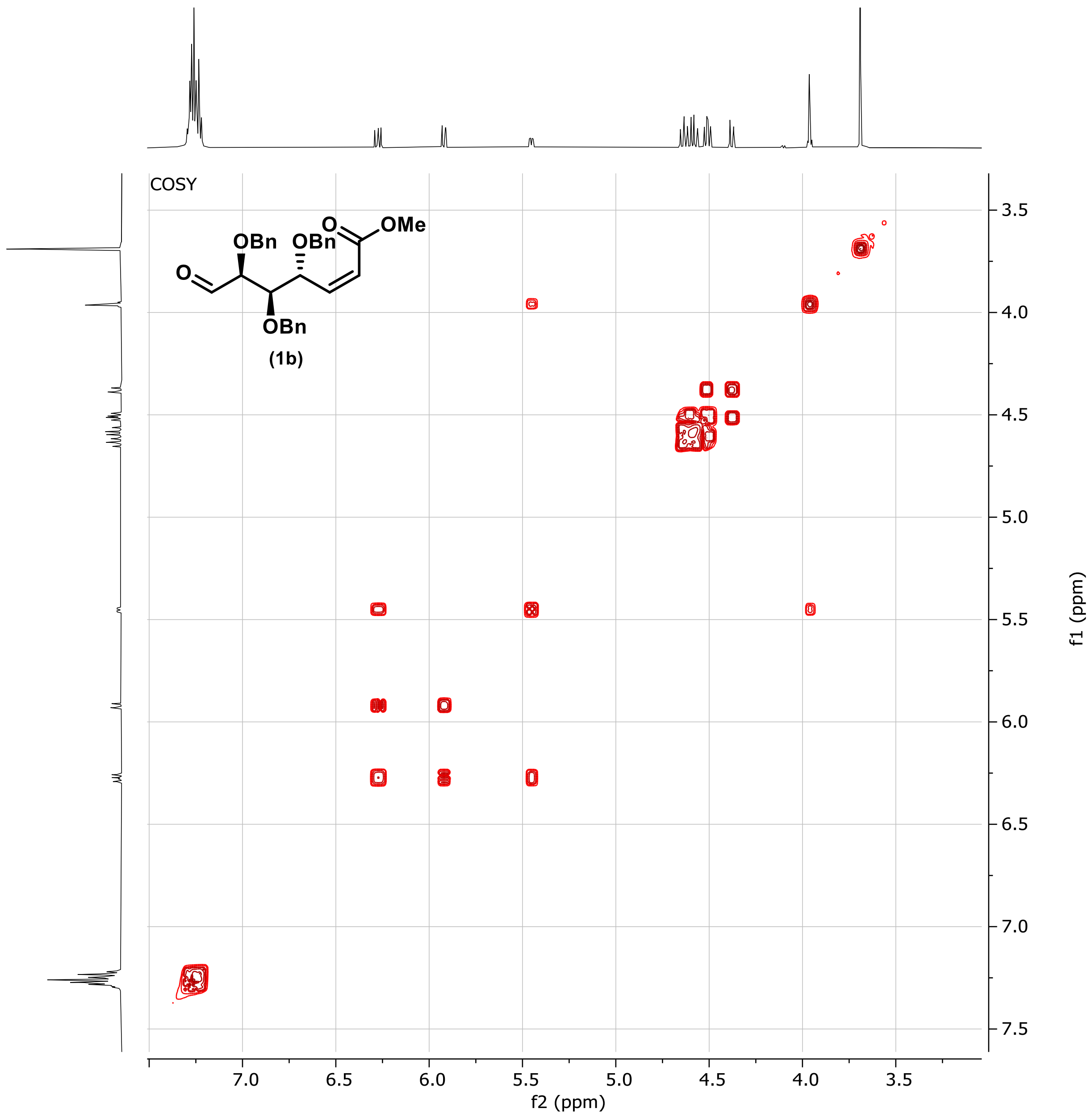

Figure S115. *COSY NMR of (1b)*

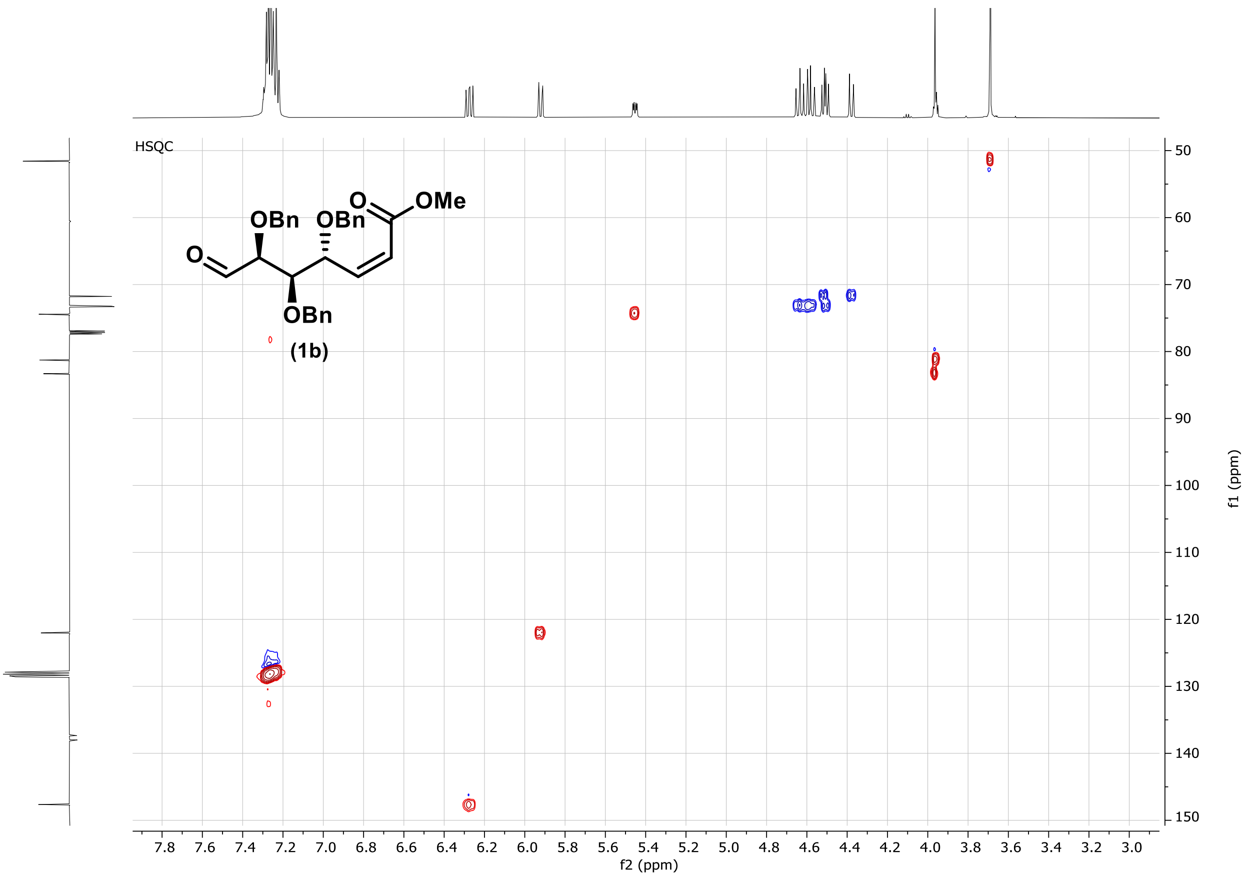

Figure S116. HSQC NMR of (1b)

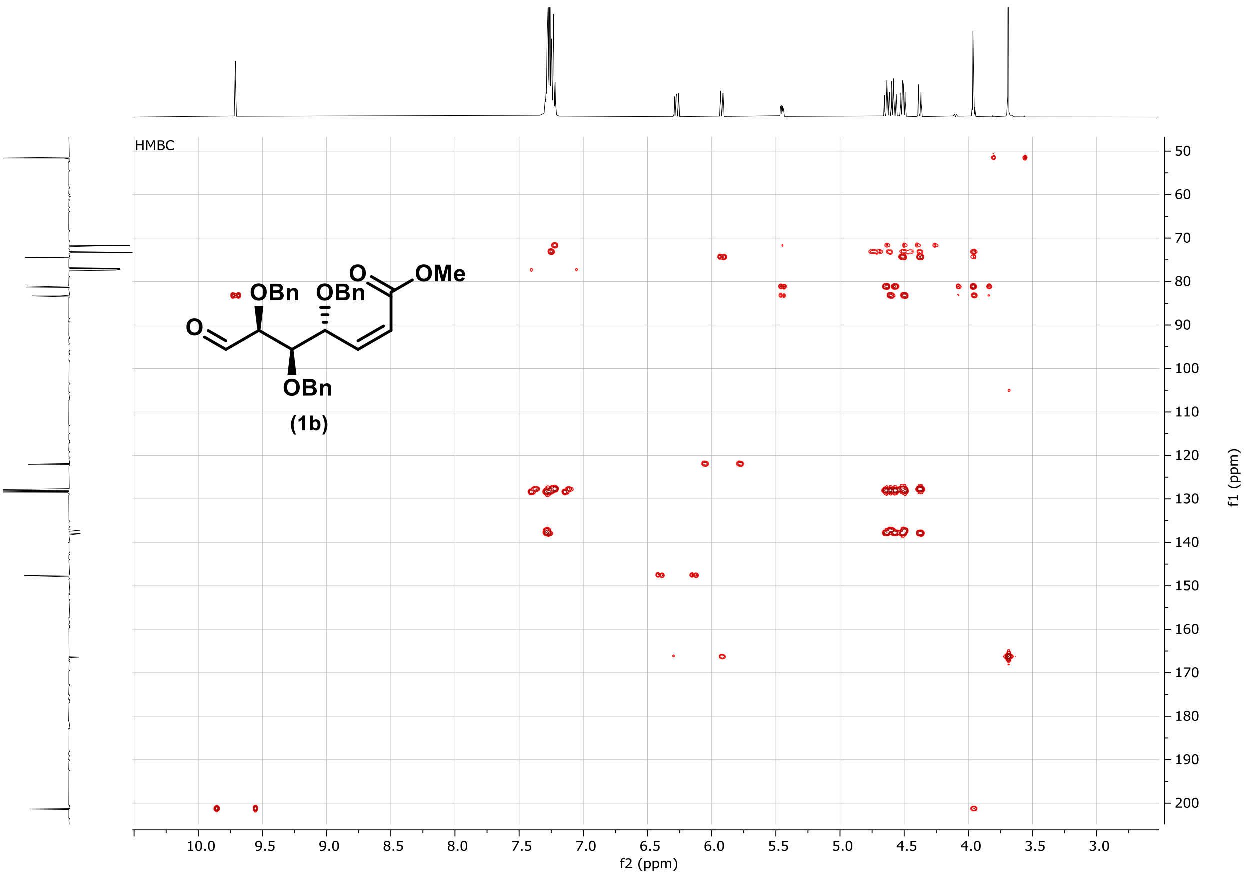

Figure S117. HMBC NMR of (1b)

D.3. Ribose derived Compounds

D.3.1. Methyl 2,3,4-tri-*O*-benzyl-β-D-ribopyranoside (4c)

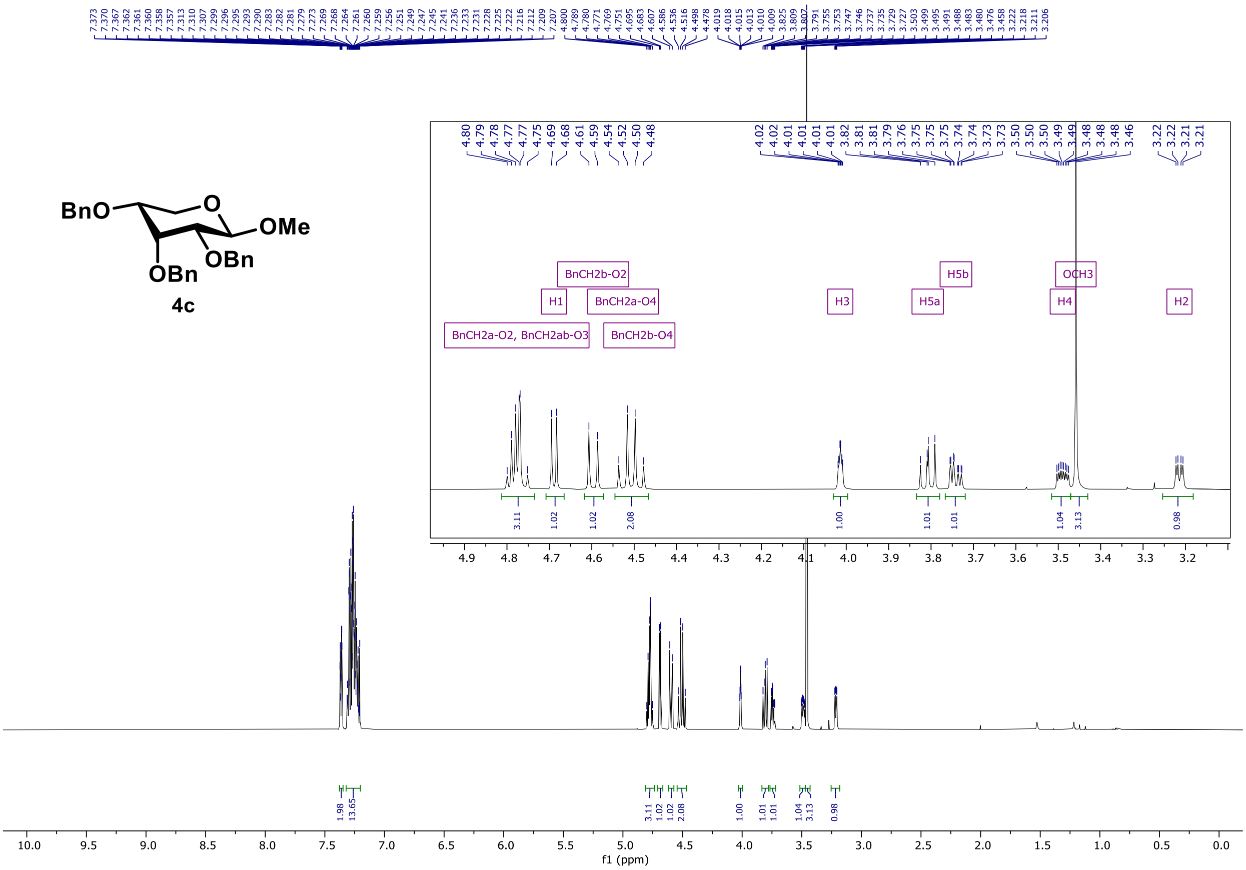

Figure S118. *600 MHz ^{1}H-NMR of (4c)*

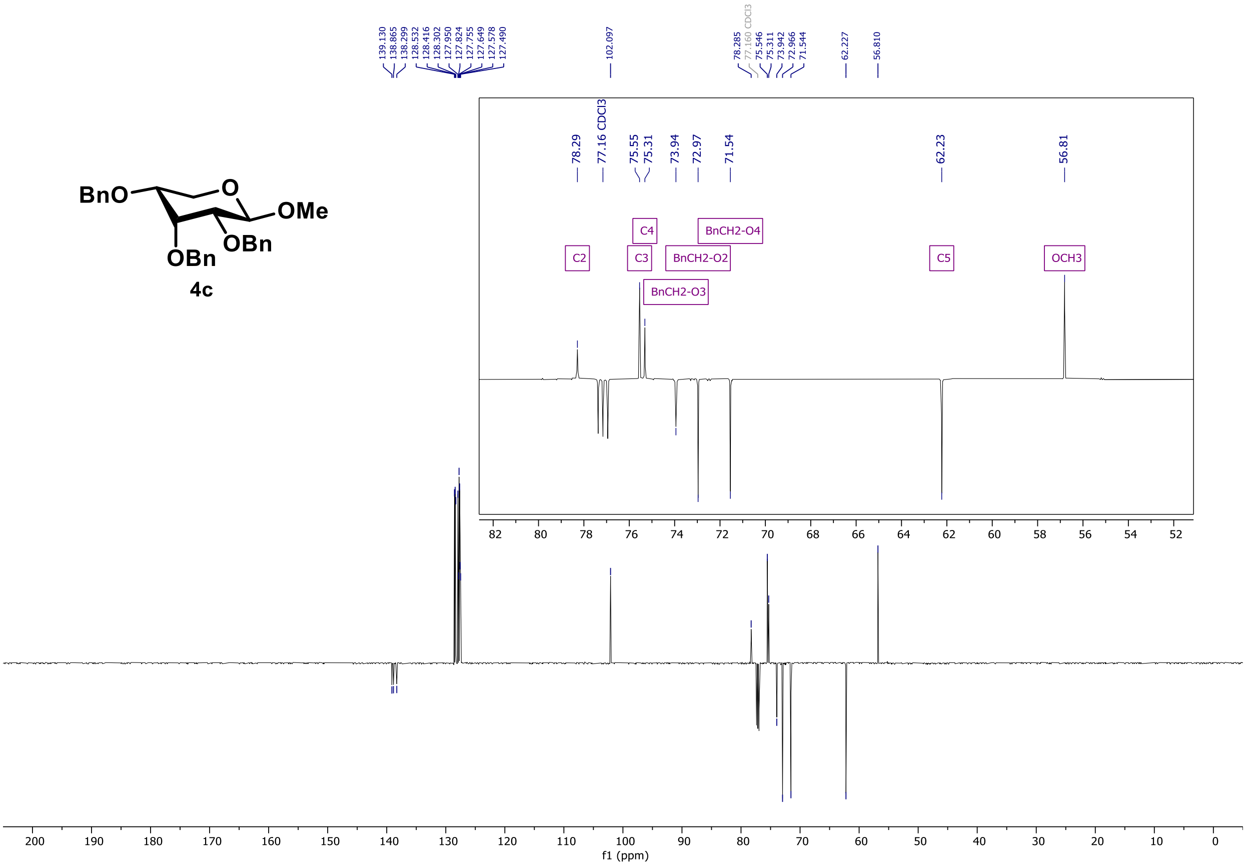

Figure S119. *151 MHz ^{13}C{^{1}H}-NMR of (4c)*

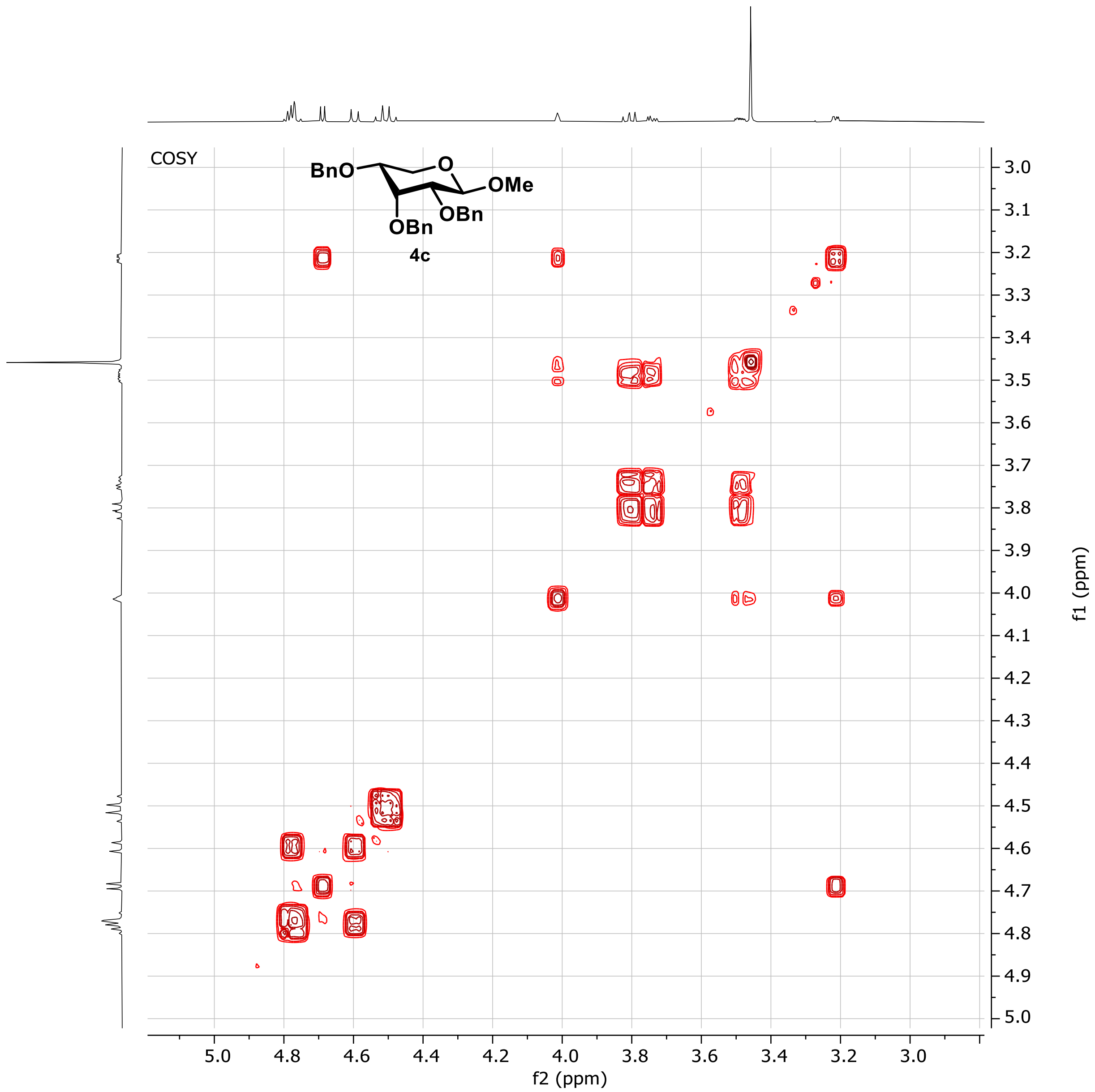

Figure S120. *COSY NMR of (4c)*

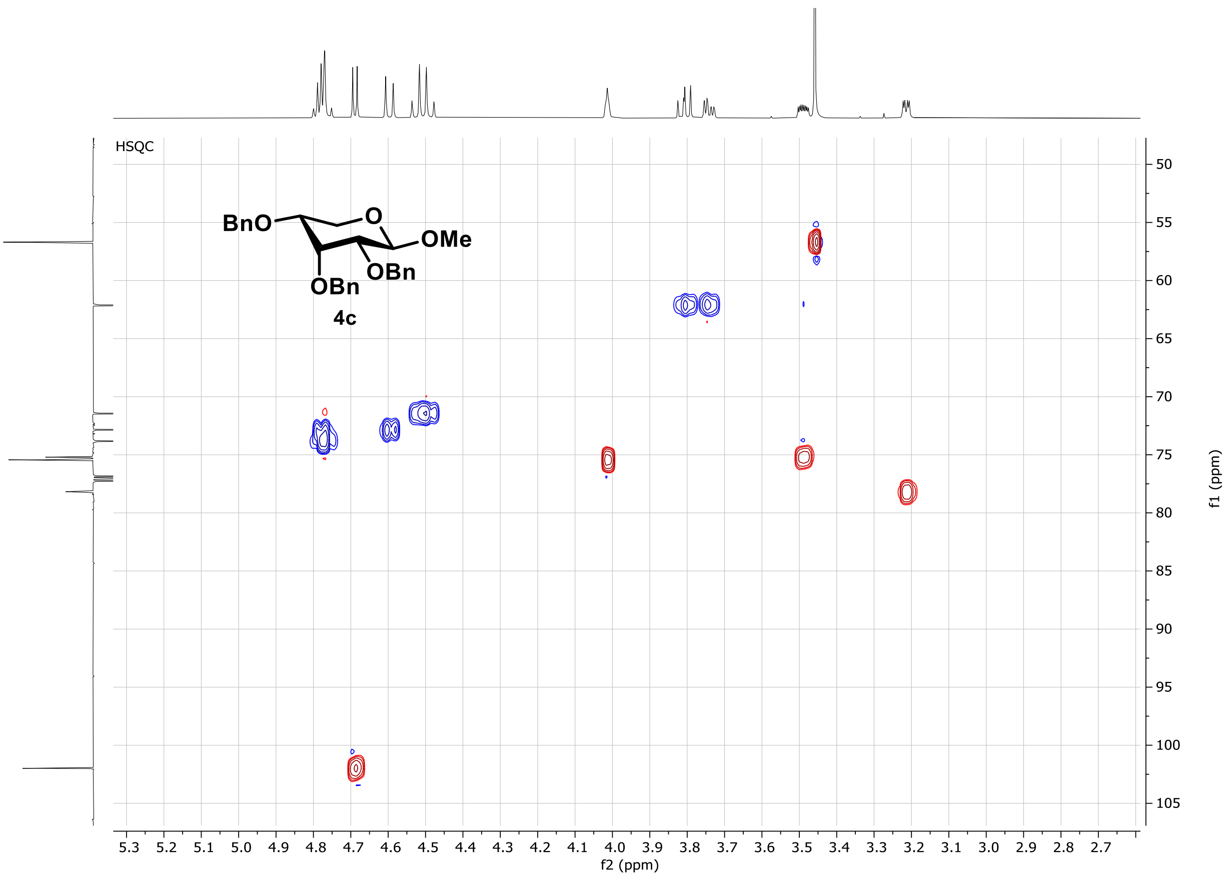

Figure S121. HSQC NMR of (4c)

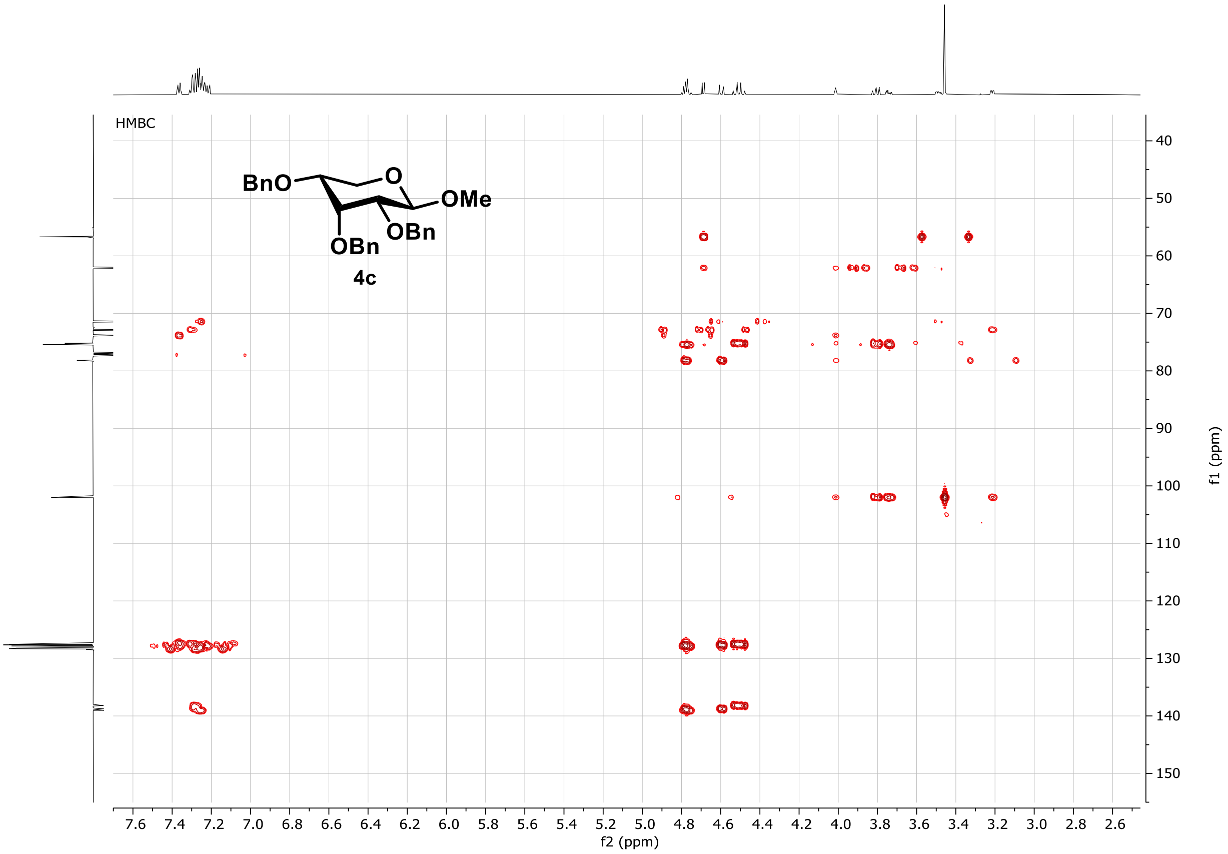

Figure S122. HMBC NMR of (4c)

D.3.2. 2,3,4-tri-*O*-benzyl-D-ribopyranose (5c)

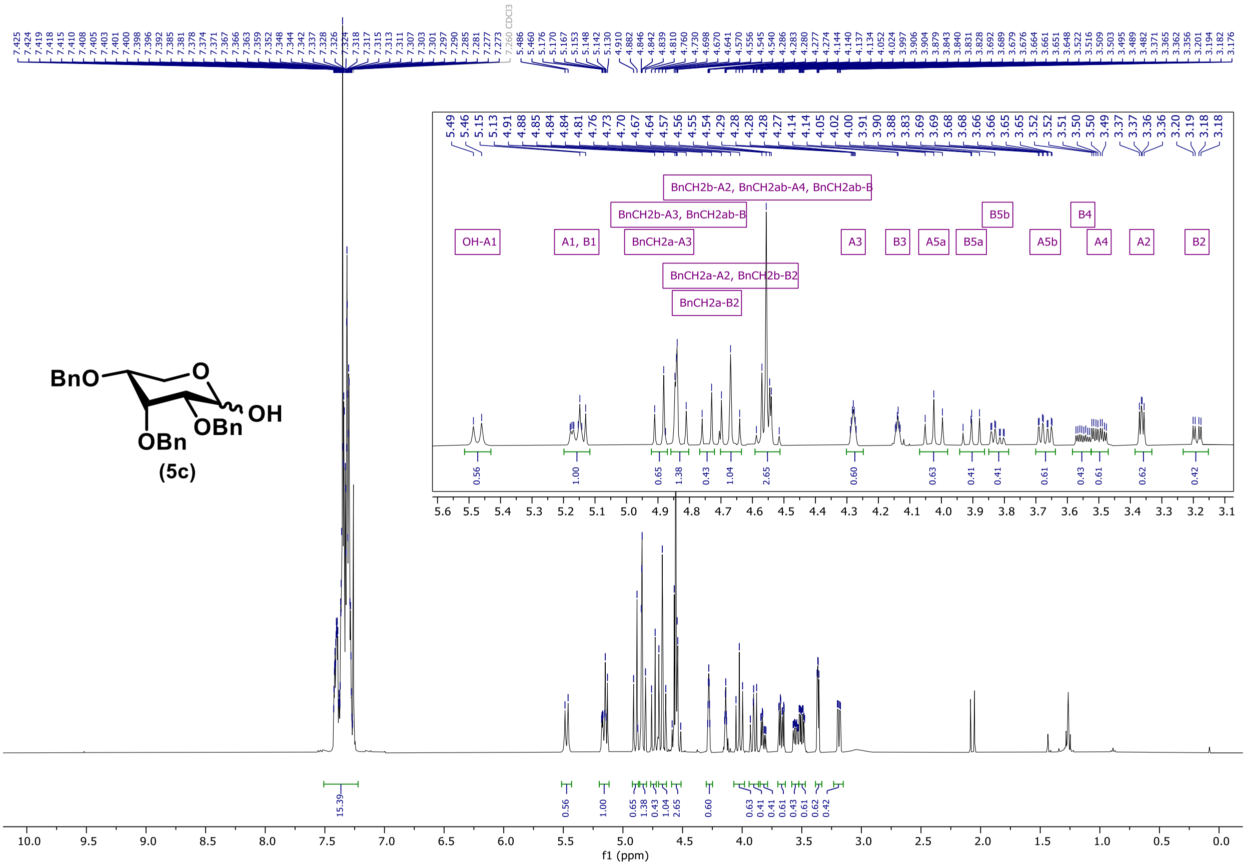

Figure S123. *600 MHz ^{1}H-NMR of (5c)*

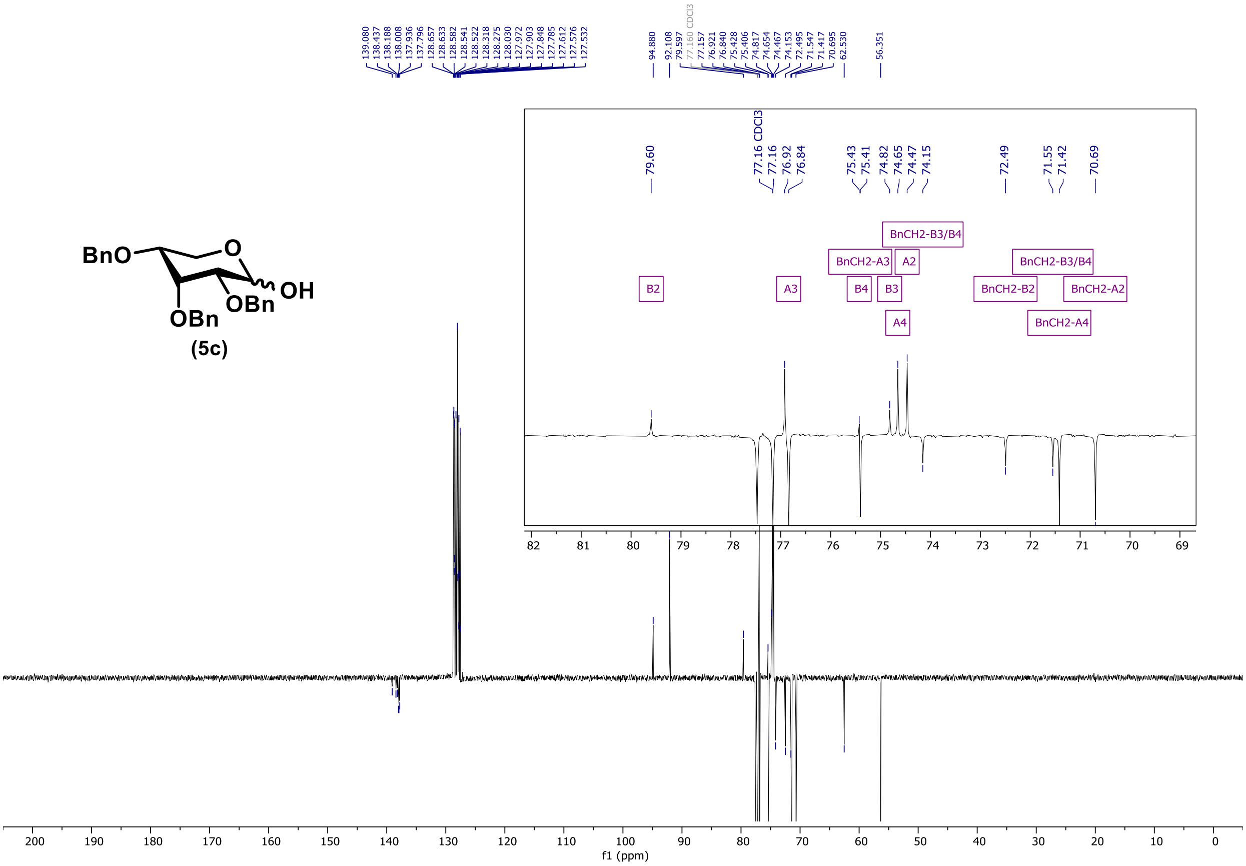

Figure S124. *151 MHz ^{13}C{^{1}H}-NMR of (5c)*

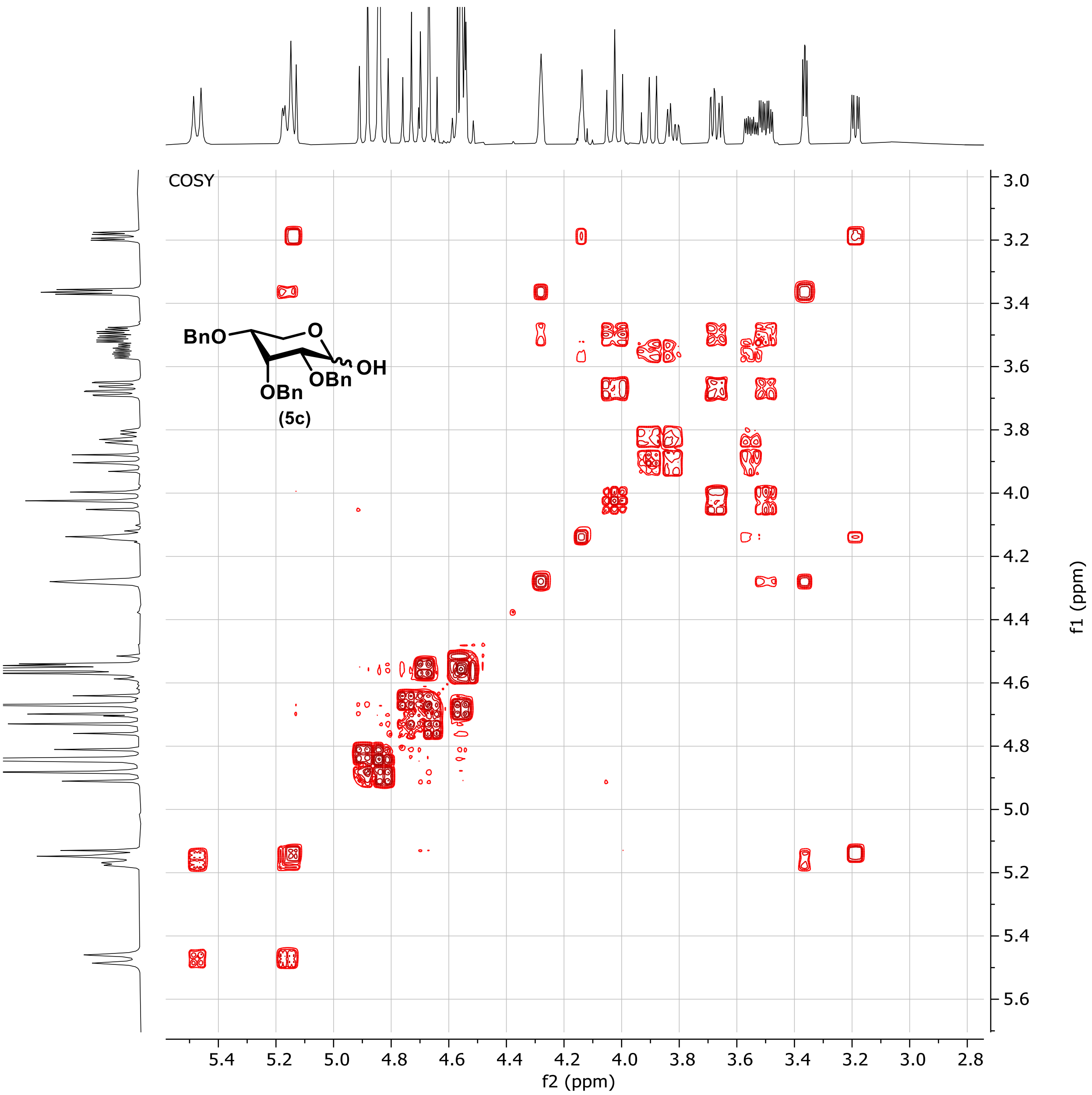

Figure S125. *COSY NMR of (5c)*

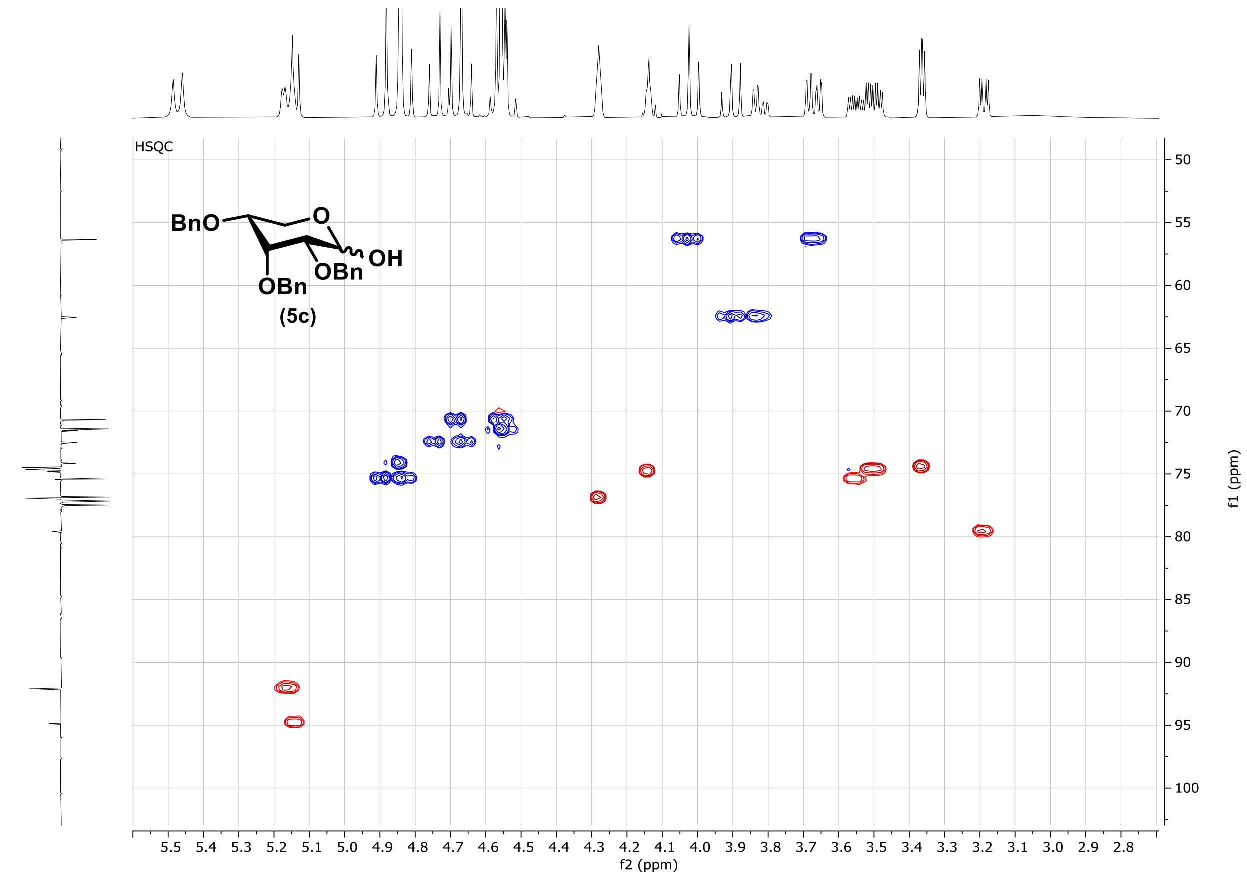

Figure S126. HSQC NMR of (5c)

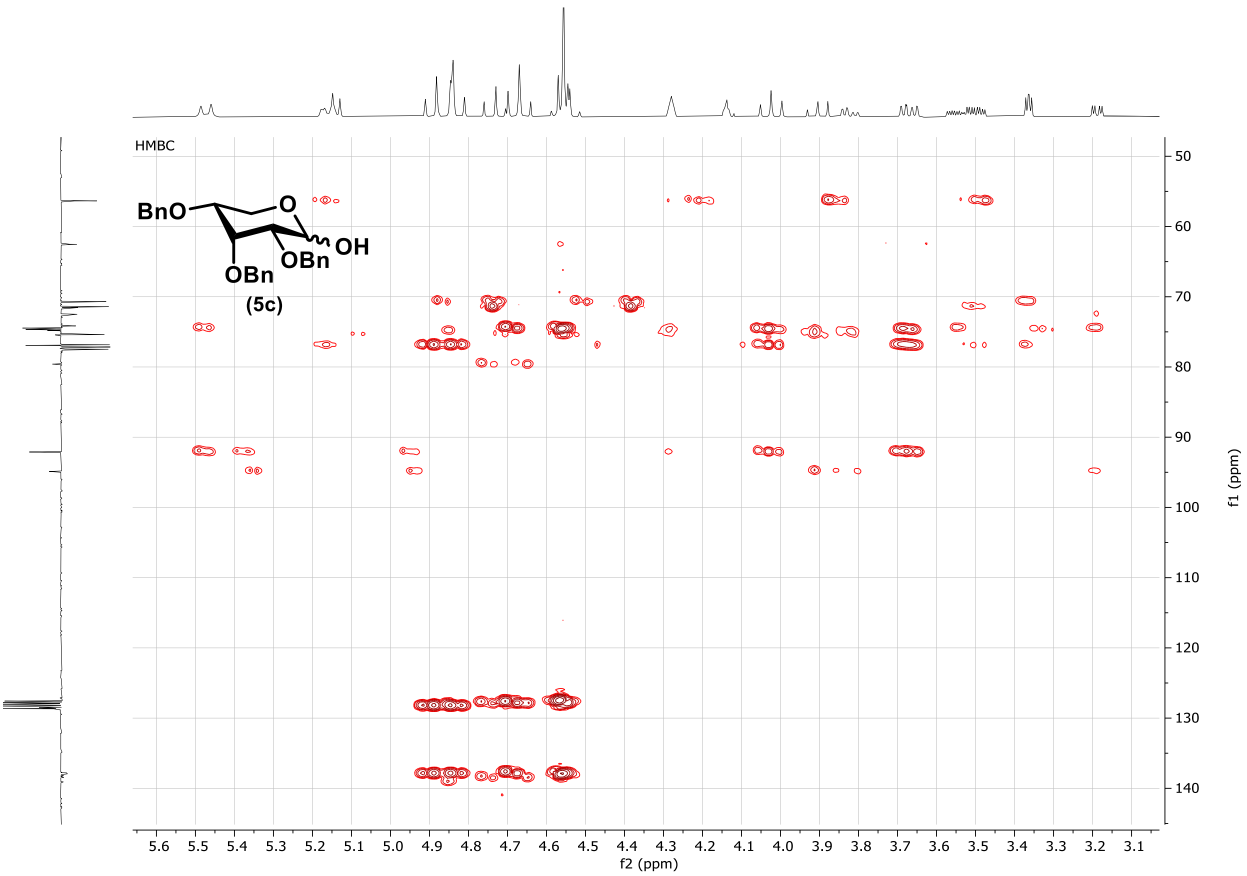

Figure S127. HMBC NMR of (5c)

D.3.3. Methyl (4S,5S,6R, E)-4,5,6-tris(benzyloxy)-7-hydroxyhept-2-enoate (6c-e)

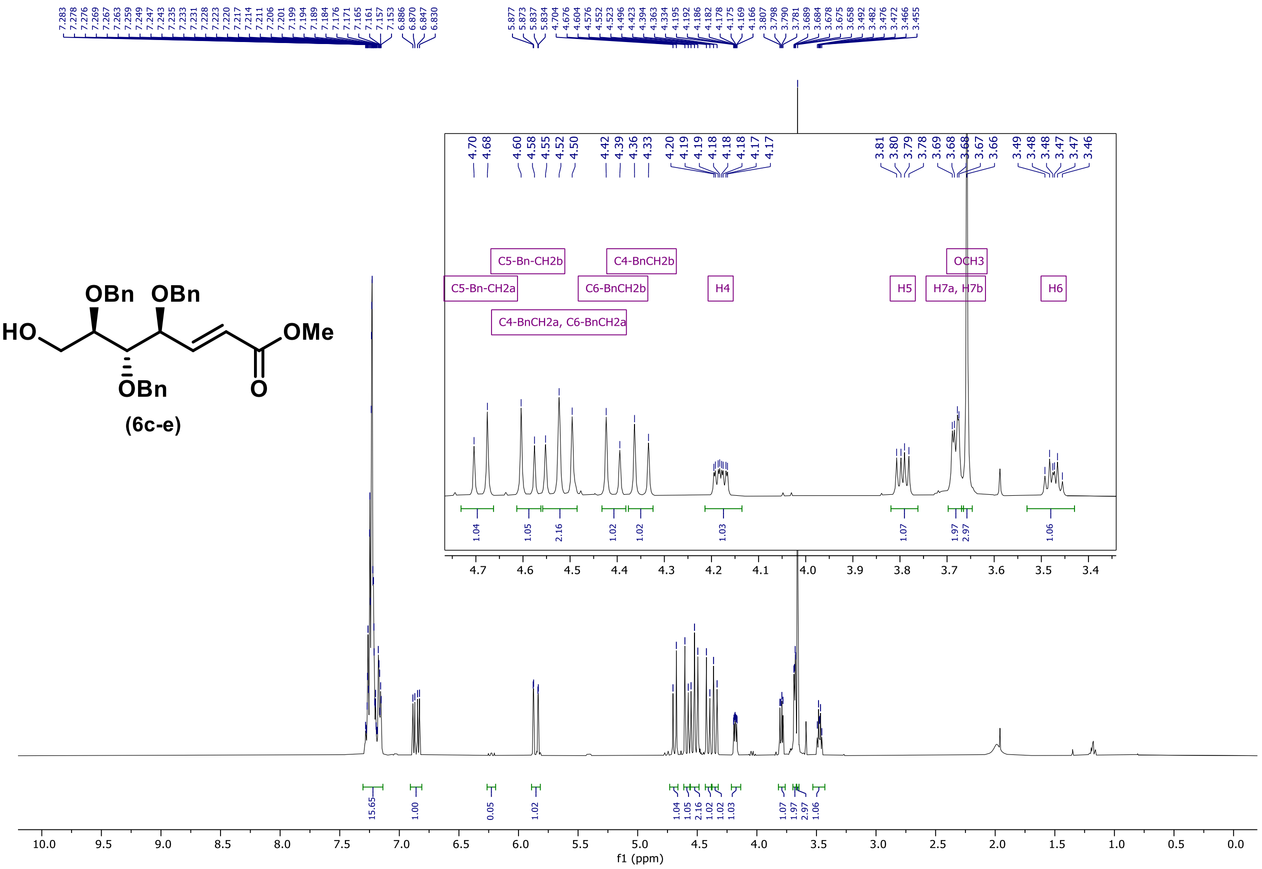

Figure S128. *600 MHz ^{1}H-NMR of (6c-e)*

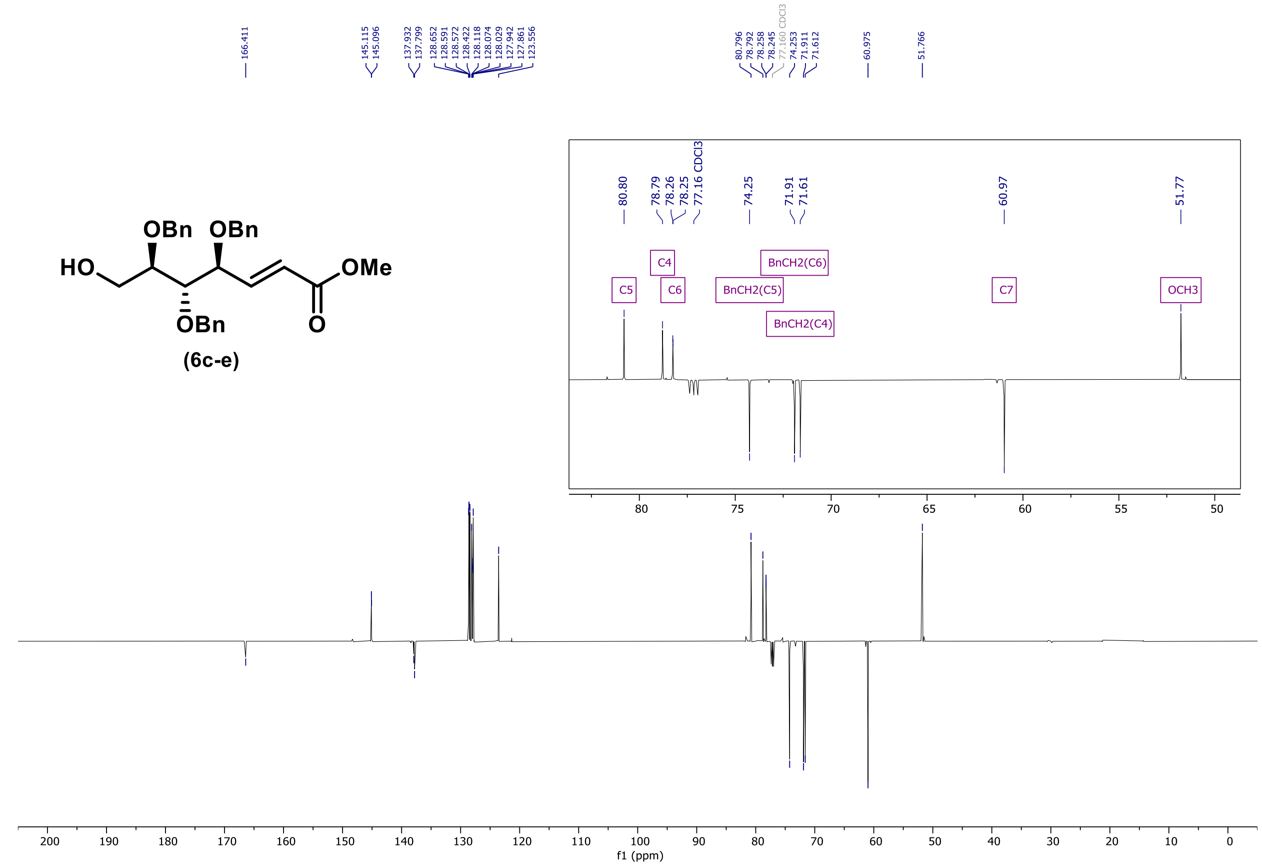

Figure S129. *151 MHz ^{13}C{^{1}H}-NMR of (6c-e)*

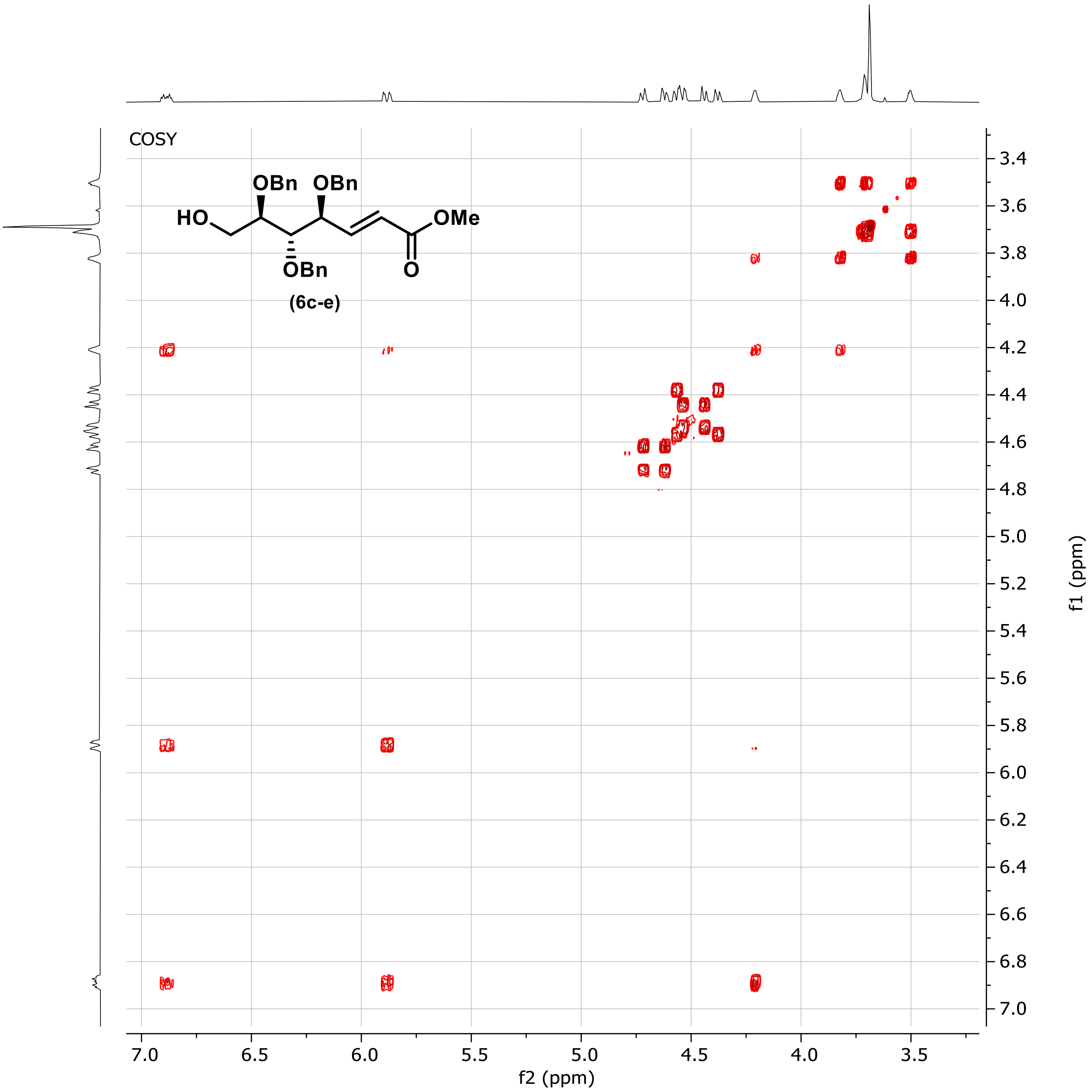

Figure S130. *COSY NMR of (6c-e)*

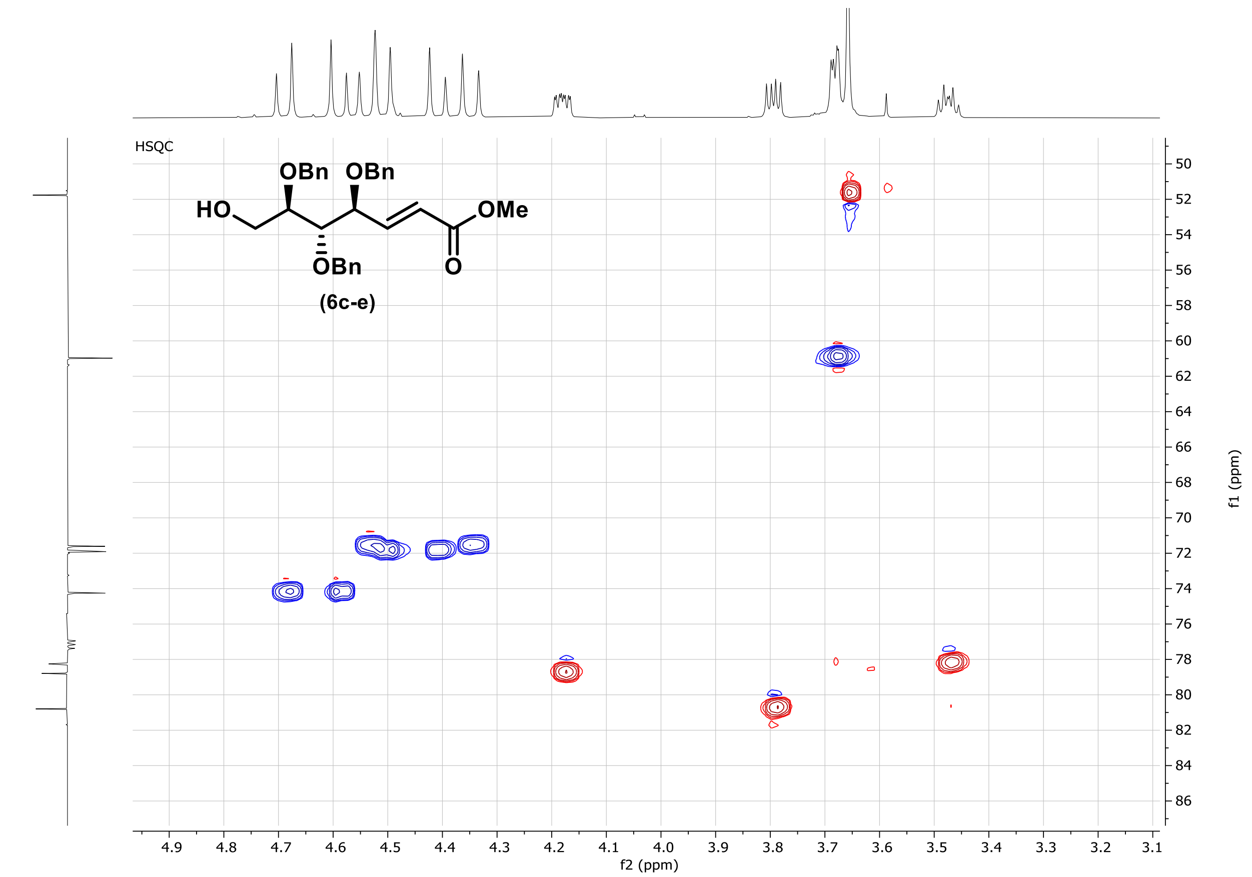

Figure S131. *HSQC NMR of (6c-e)*

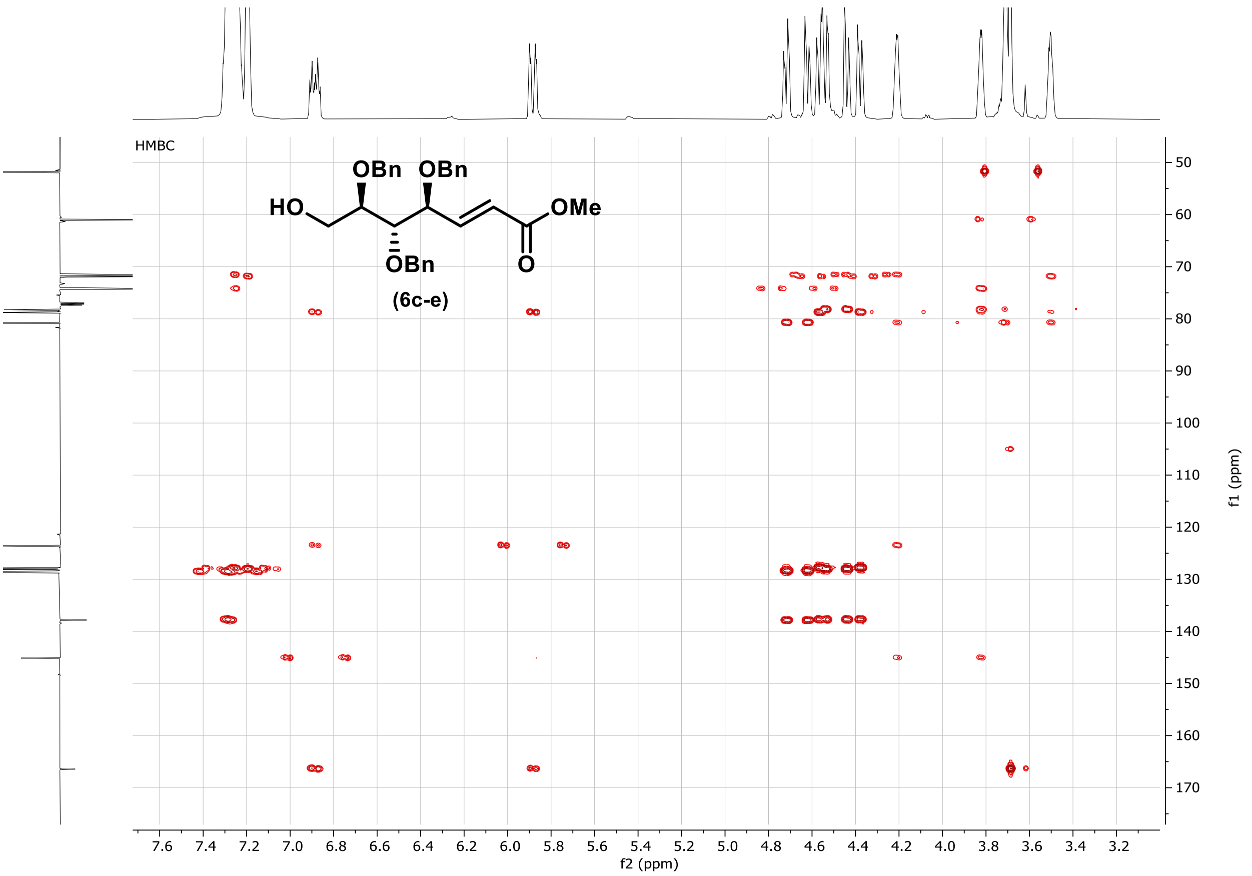

Figure S132. *HMBC NMR of (6c-e)*

D.3.4. Methyl (4S,5S,6R, Z)-4,5,6-tris(benzyloxy)-7-hydroxyhept-2-enoate (6c-z)

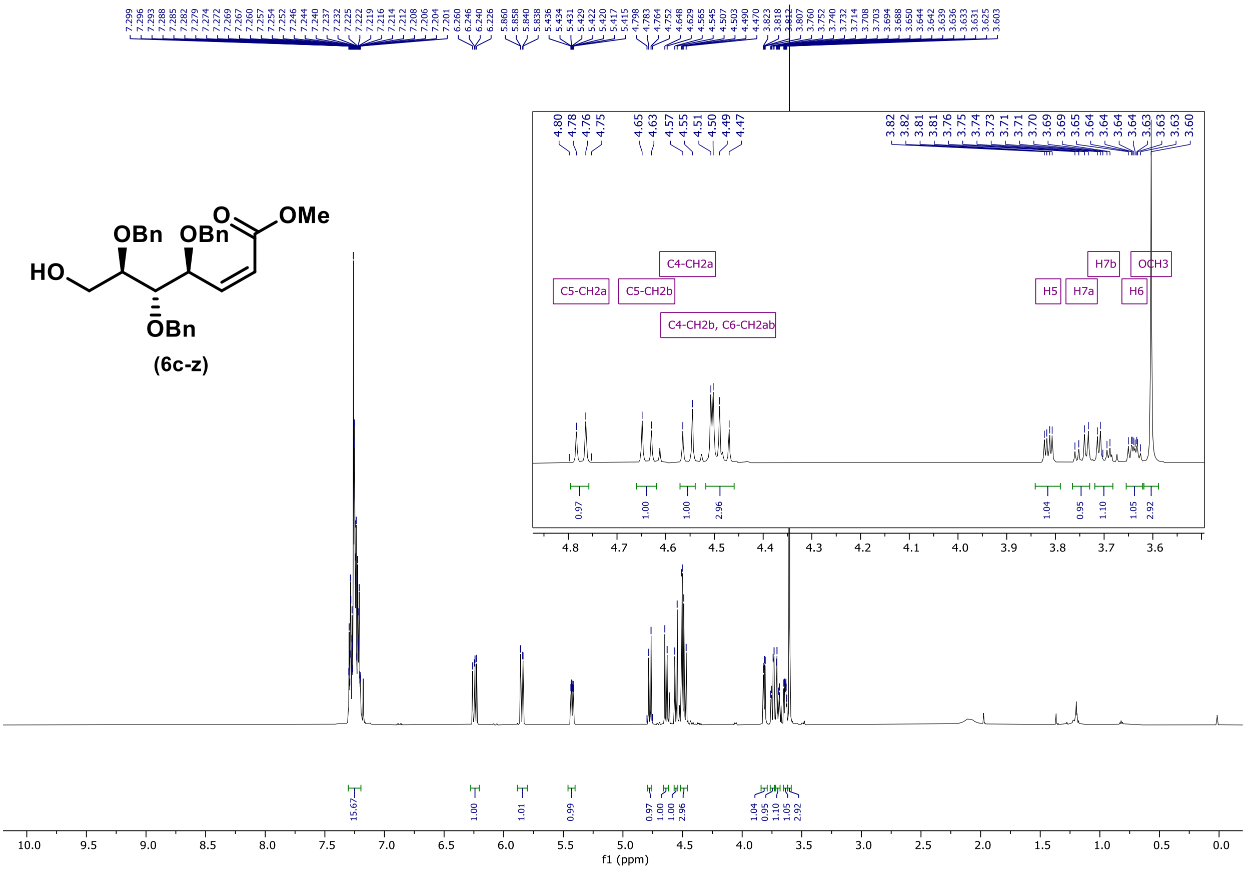

Figure S133. *600 MHz ¹H-NMR of (6c-z)*

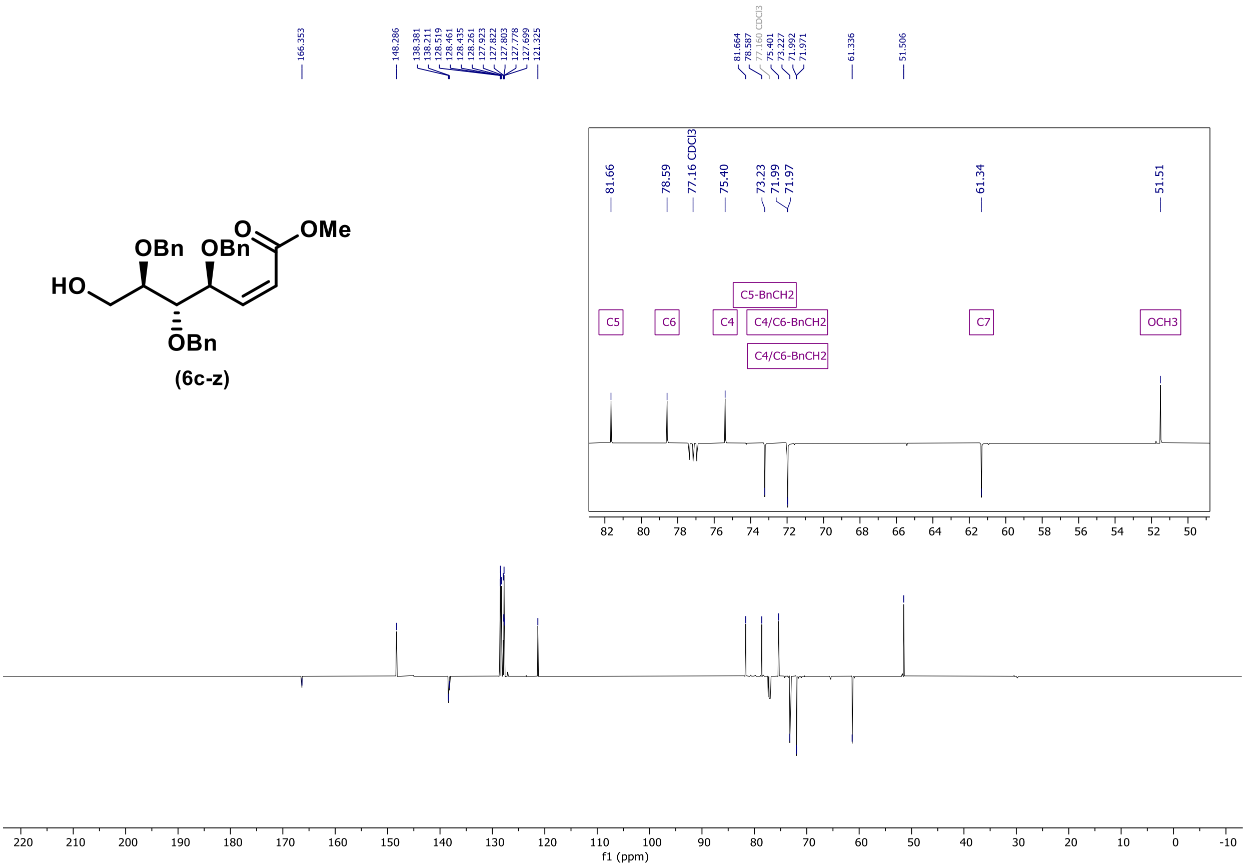

Figure S134. *151 MHz $^{13}C\{^{1}H\}$-NMR of (6c-z)*

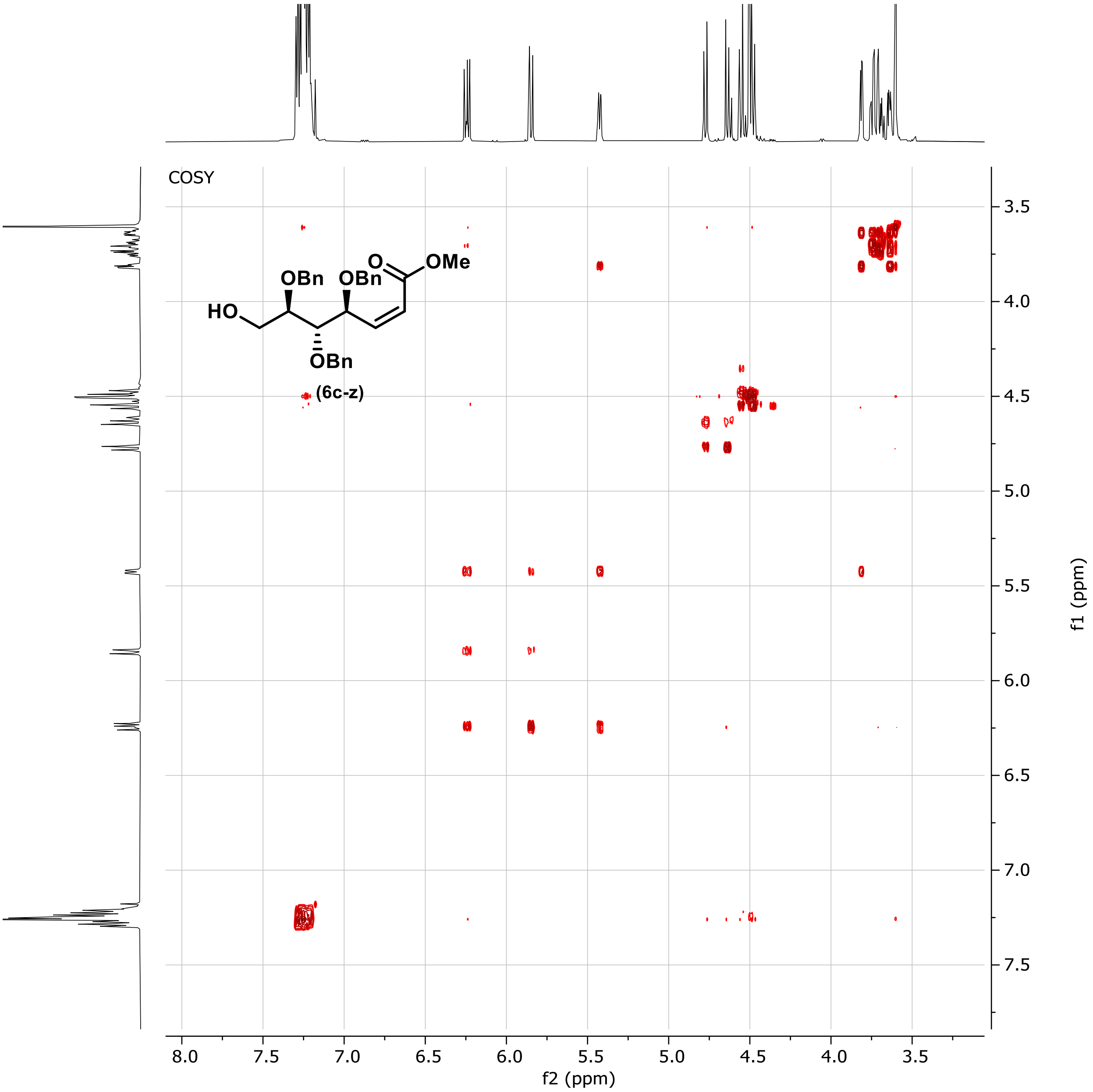

Figure S135. *COSY NMR of (6c-z)*

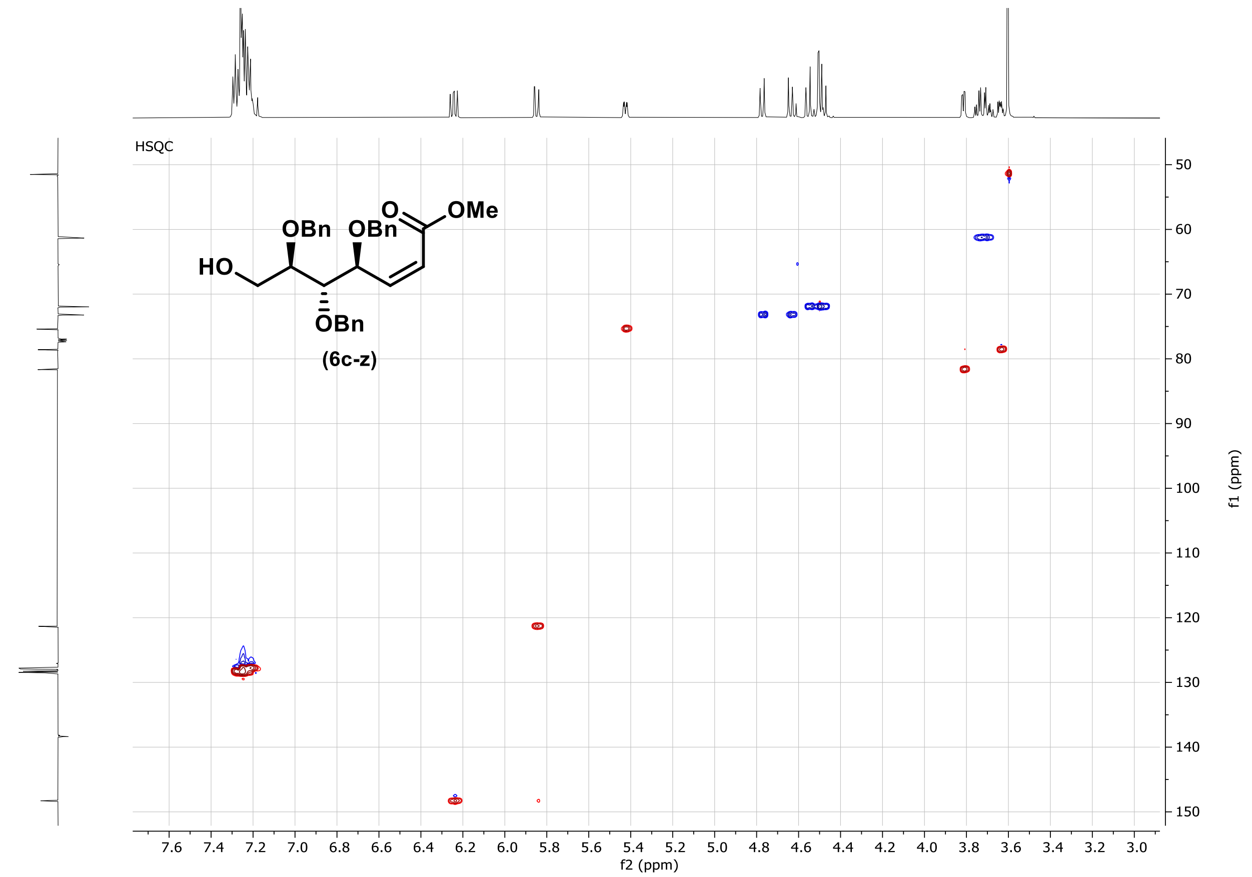

Figure S136. HSQC NMR of (6c-z)

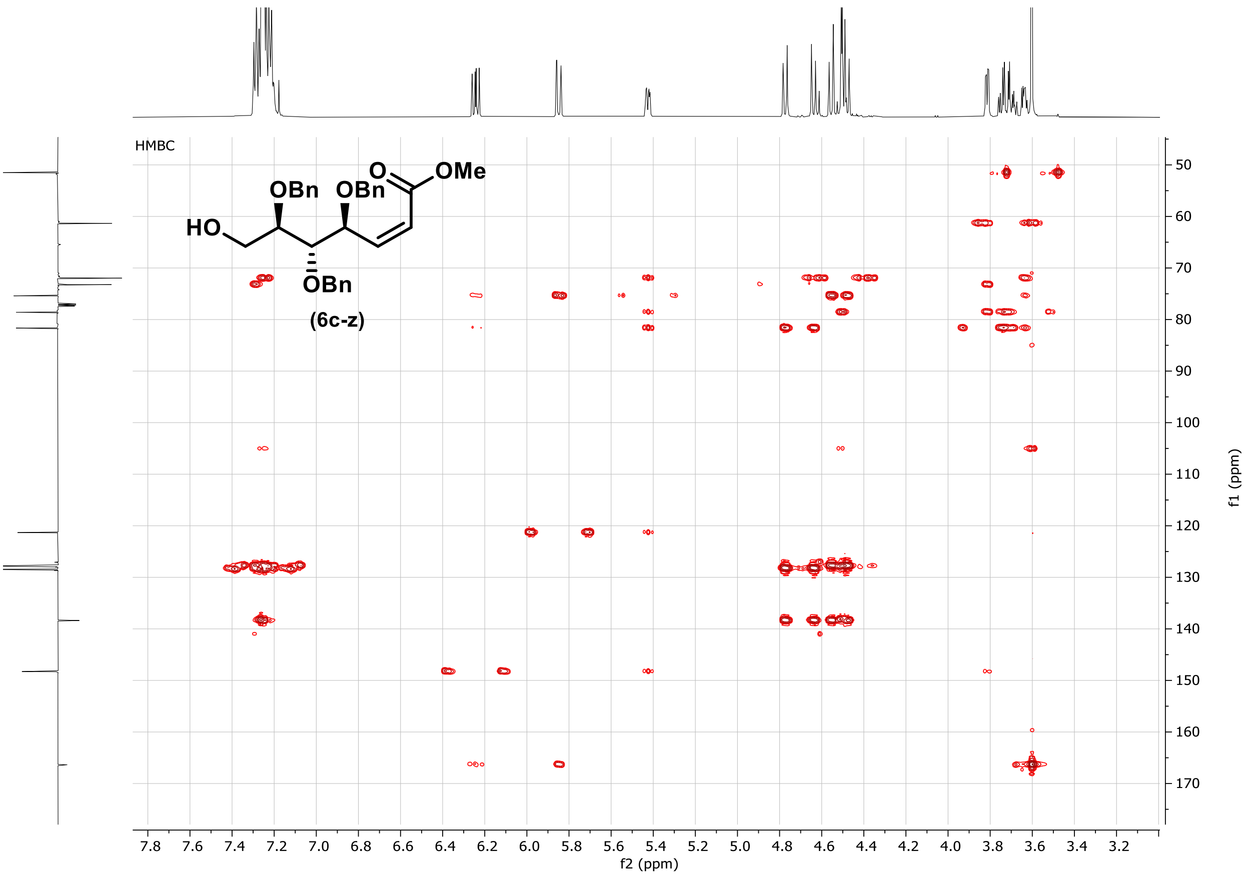

Figure S137. HMBC NMR of (6c-z)

D.3.5. Methyl (4S, 5S, 6S, E)-4,5,6-tris(benzyloxy)-7-oxohept-2-enoate (1c)

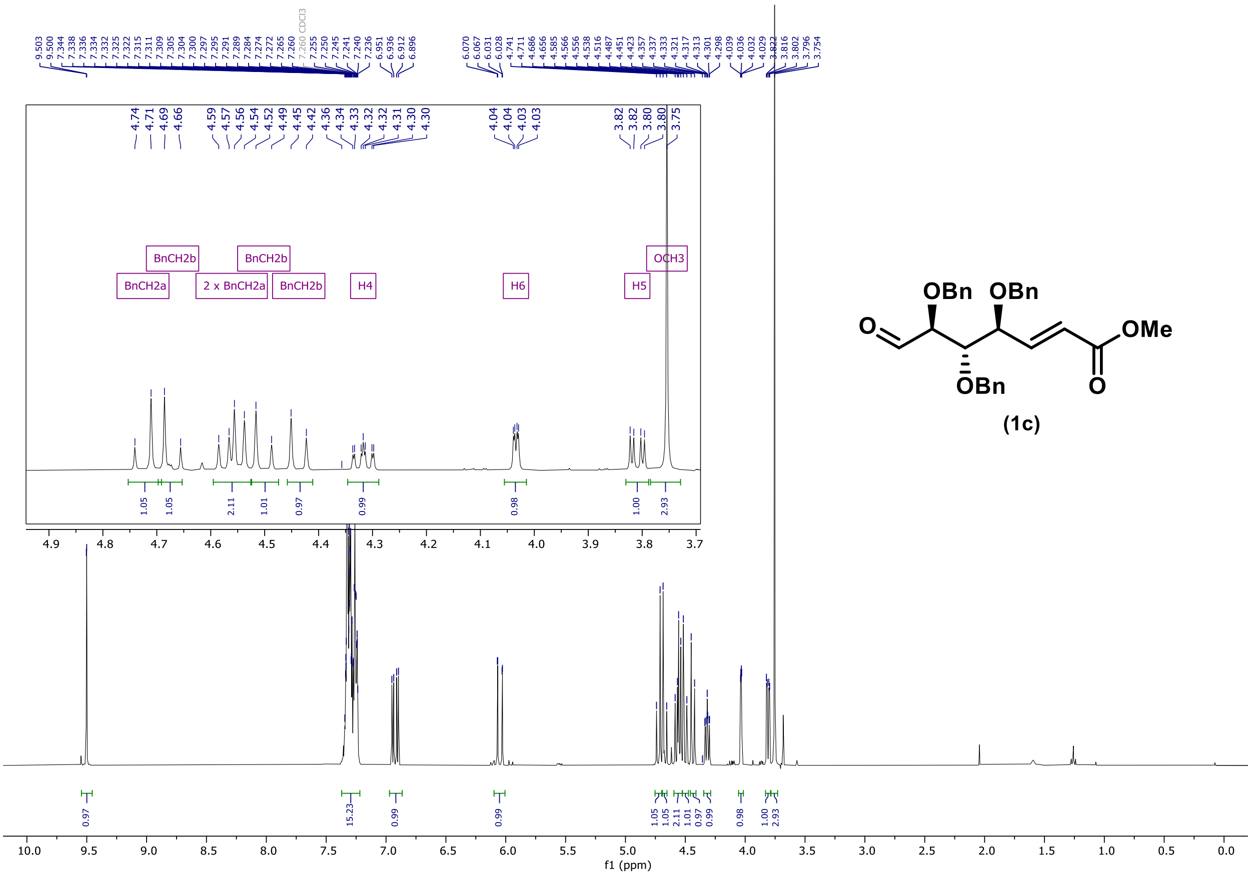

Figure S138. *600 MHz ^{1}H-NMR of (1c)*

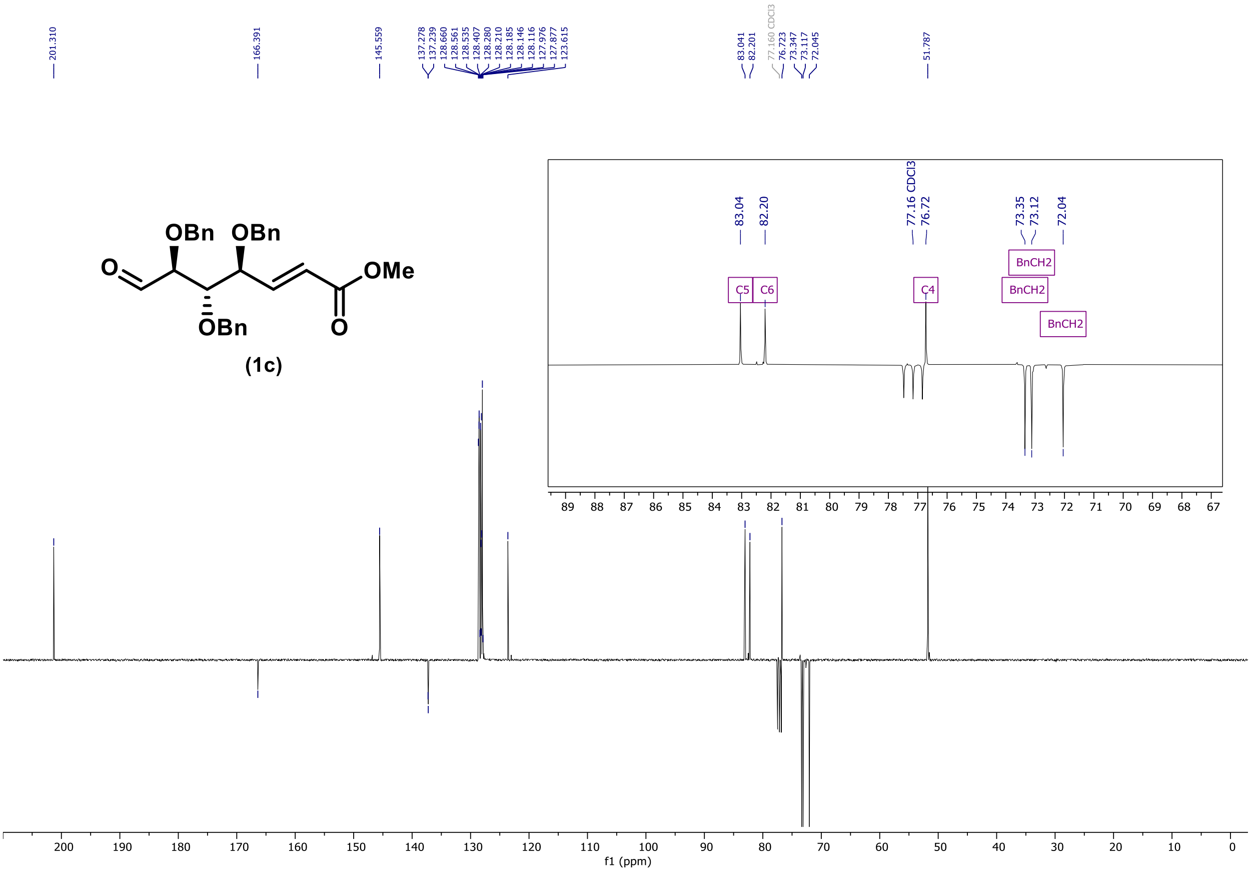

Figure S139. *151 MHz $^{13}C\{^{1}H\}$-NMR of (1c)*

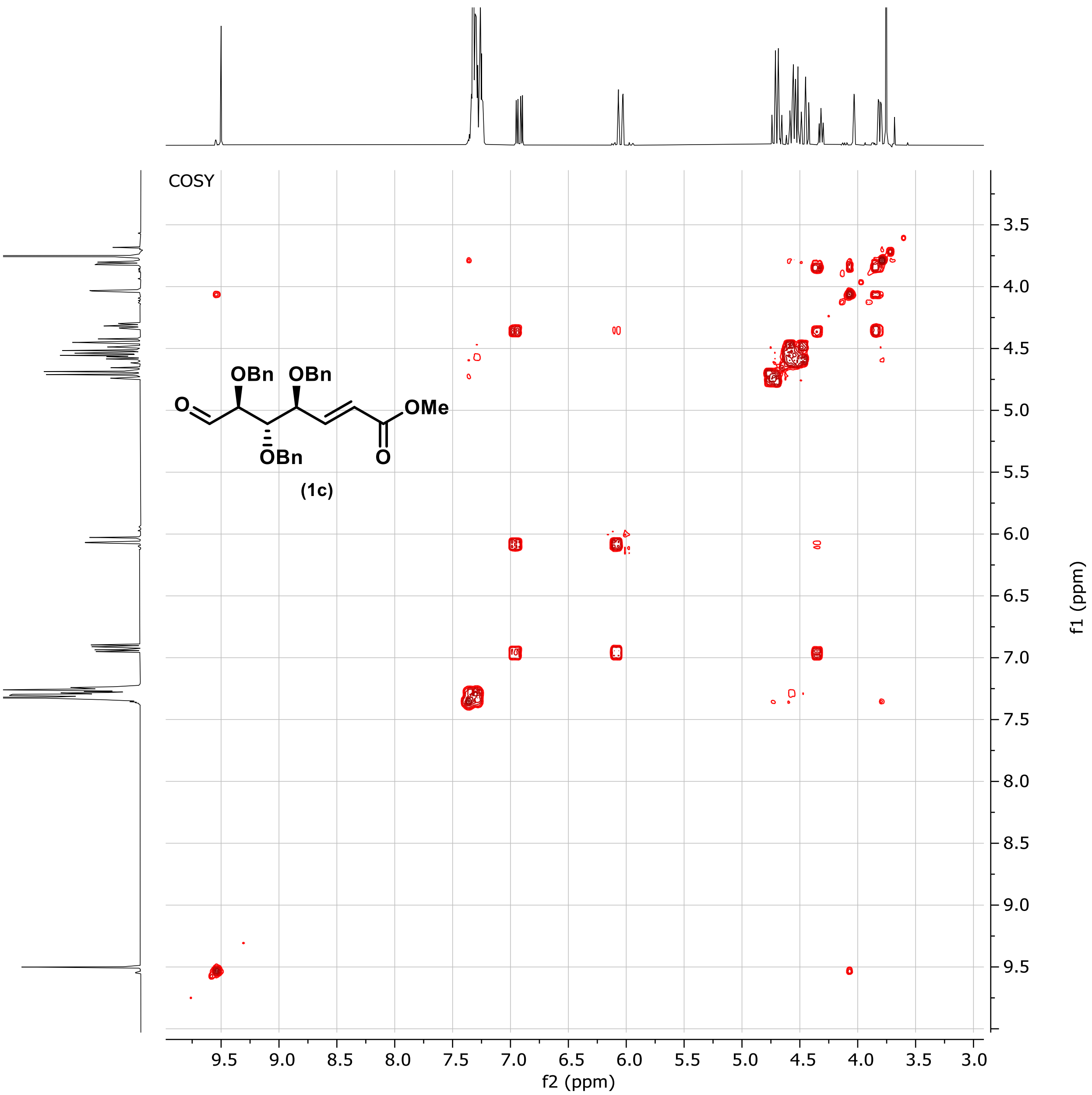

Figure S140. *COSY NMR of (1c)*

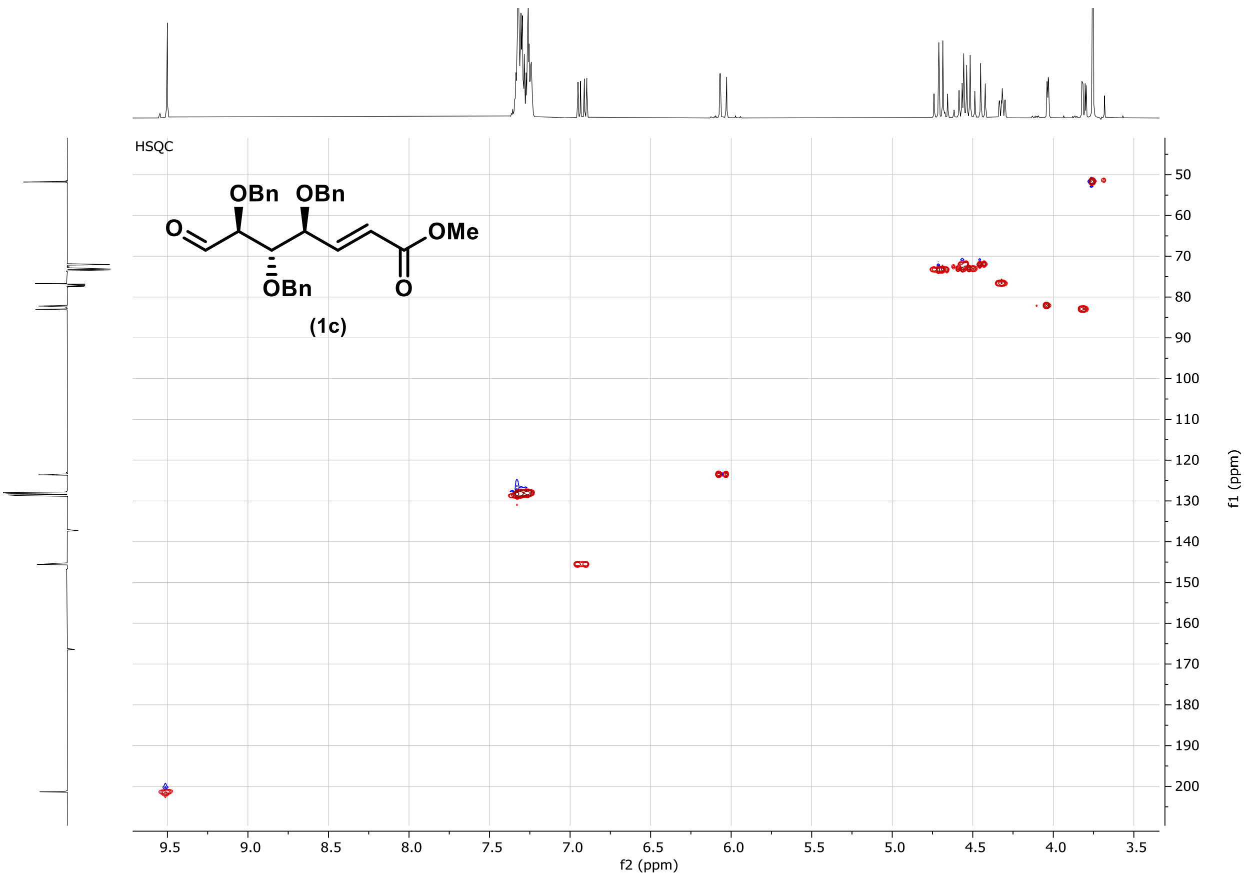

Figure S141. *HSQC NMR of (1c)*

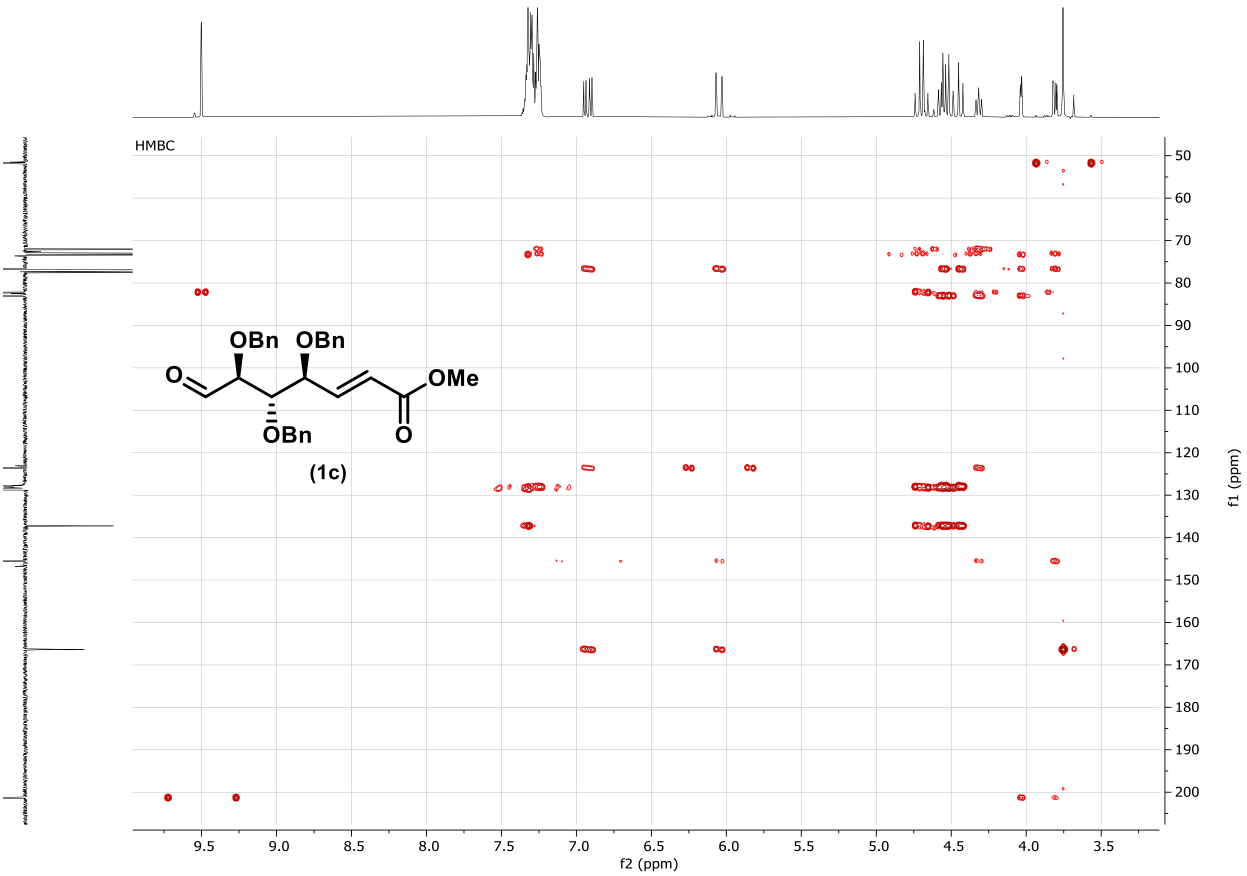

Figure S142. *HMBC NMR of (1c)*

D.4. Arabinose derived Compounds

D.4.1. Methyl 2,3,4-tri-*O*-benzyl-β-D-arabinopyranoside (1d)

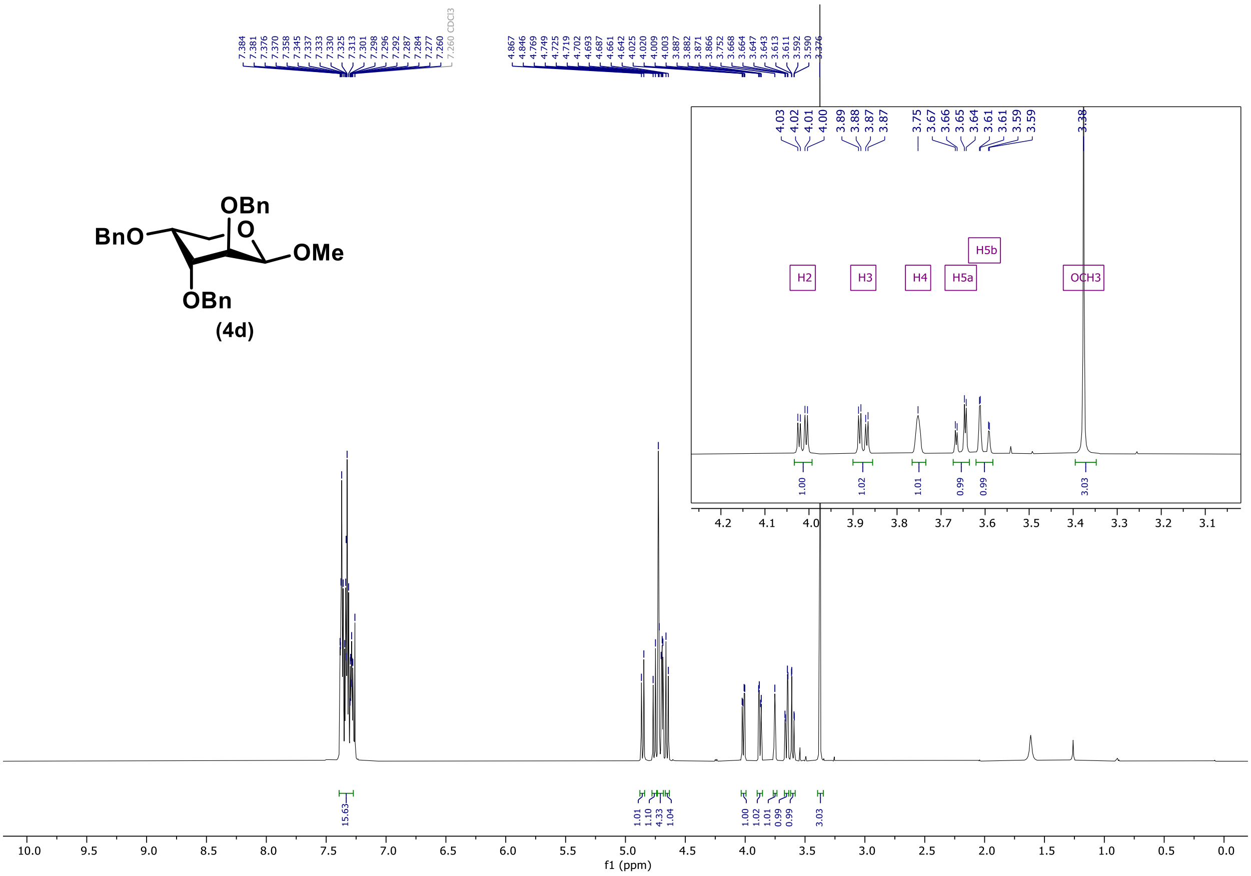

Figure S143. *600 MHz ^{1}H-NMR of (1d)*

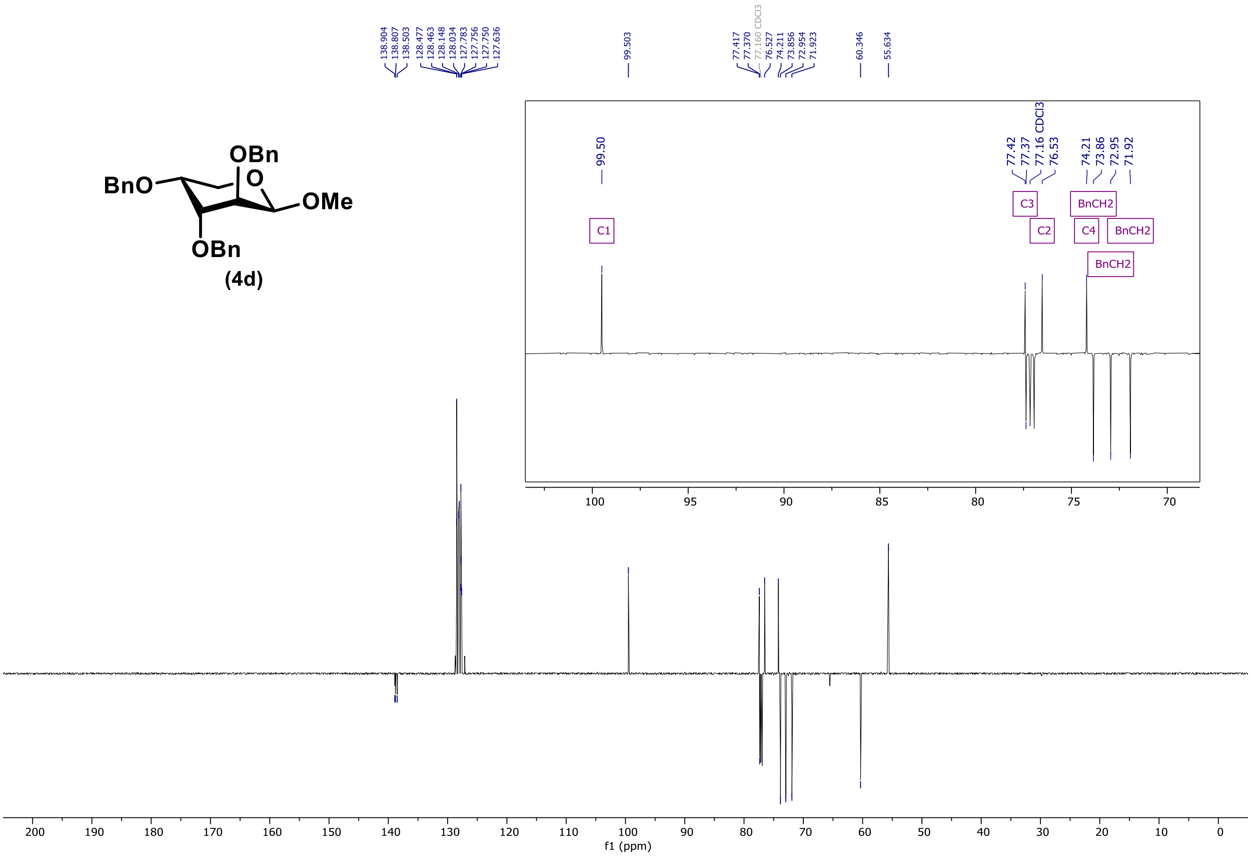

Figure S144. *151 MHz $^{13}C\{^{1}H\}$-NMR of (1d)*

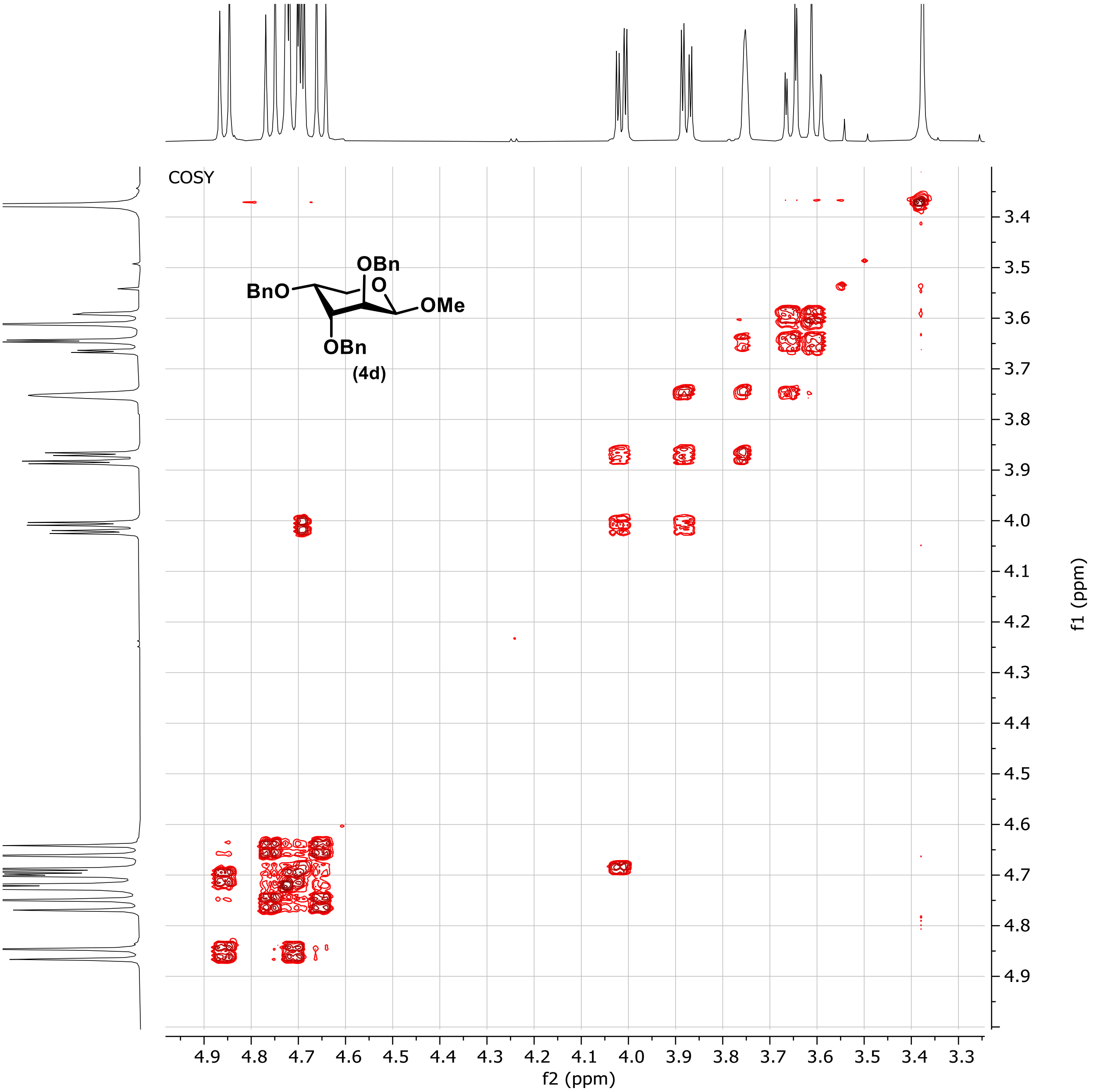

Figure S145. COSY NMR of (4d)

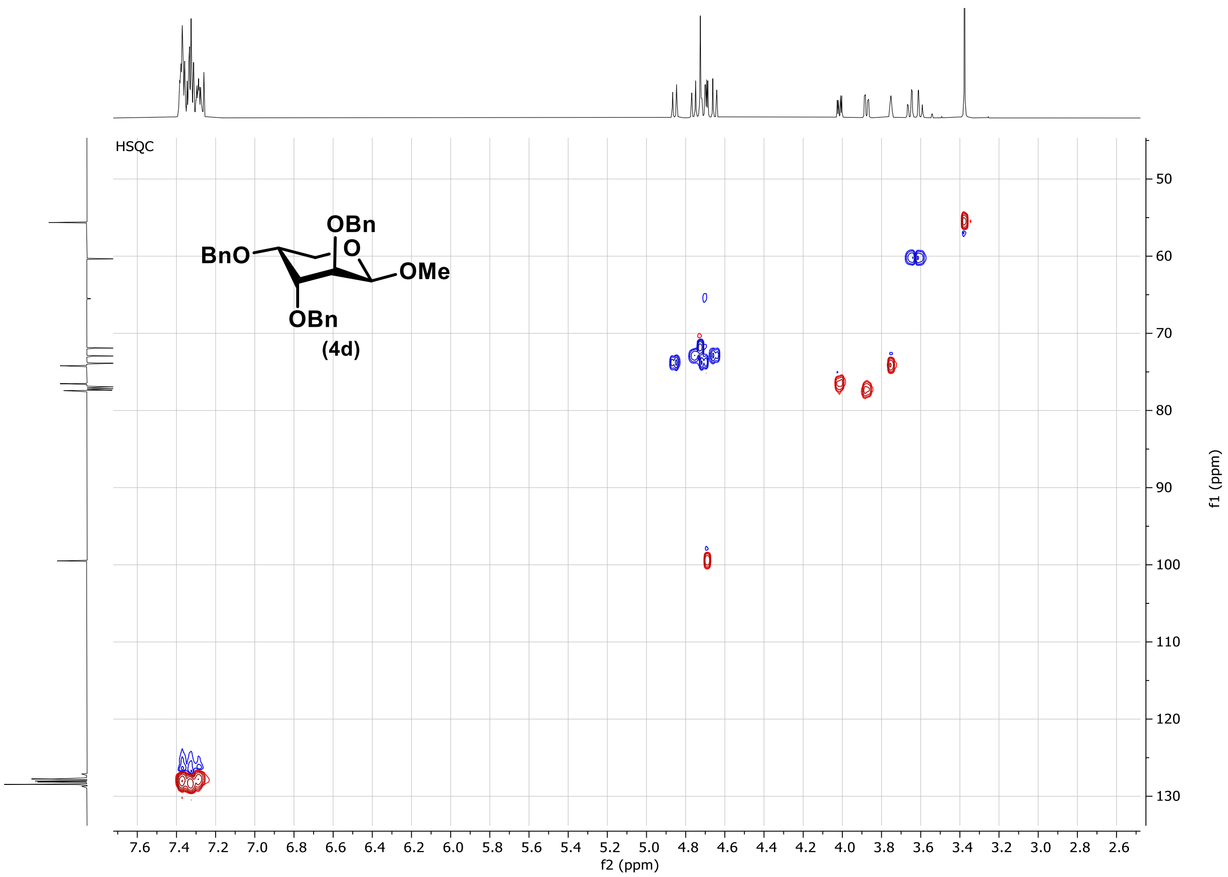

Figure S146. *HSQC NMR of (4d)*

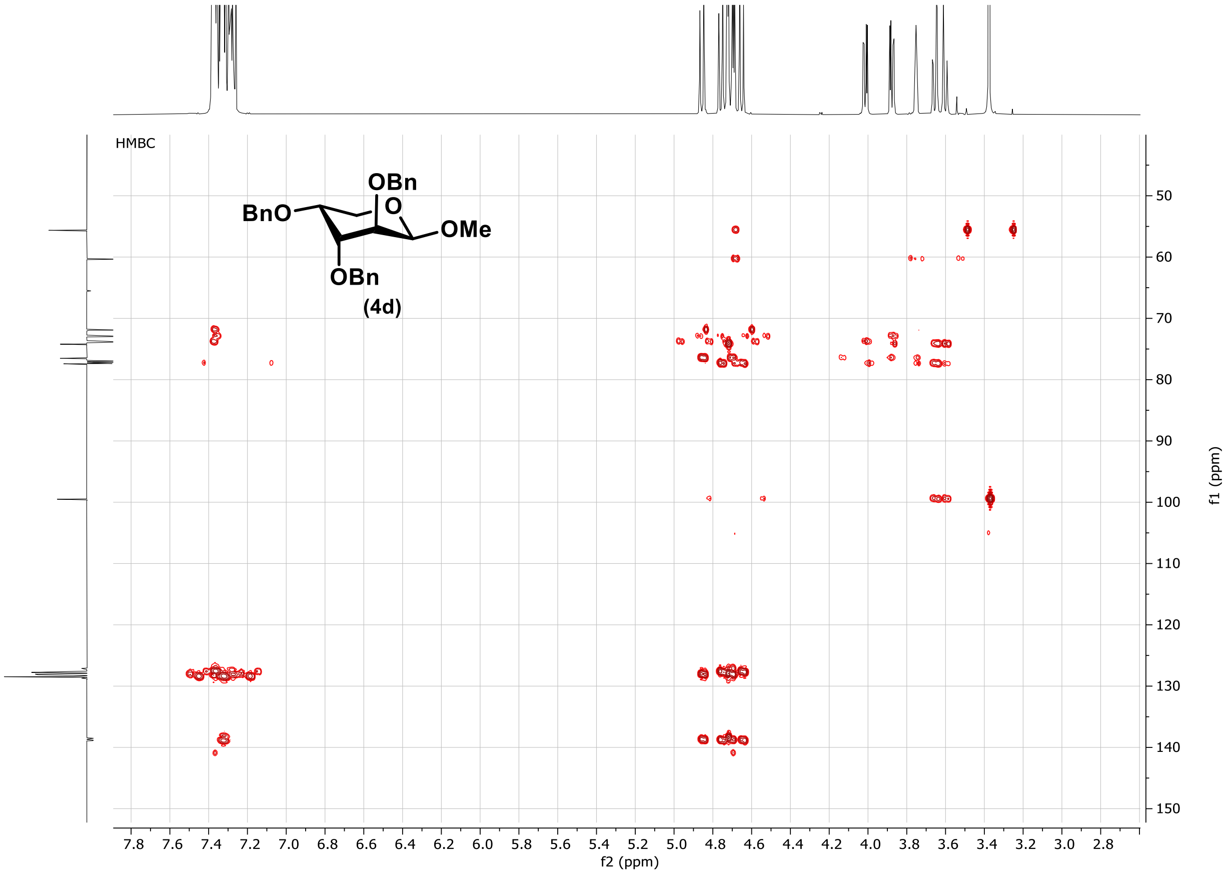

Figure S147. *HMBC NMR of (4d)*

D.4.2. Methyl (4S, 5S, 6S)-4,5,6-tris(benzyloxy)-7-hydroxyhept-2-enoate (S5a)

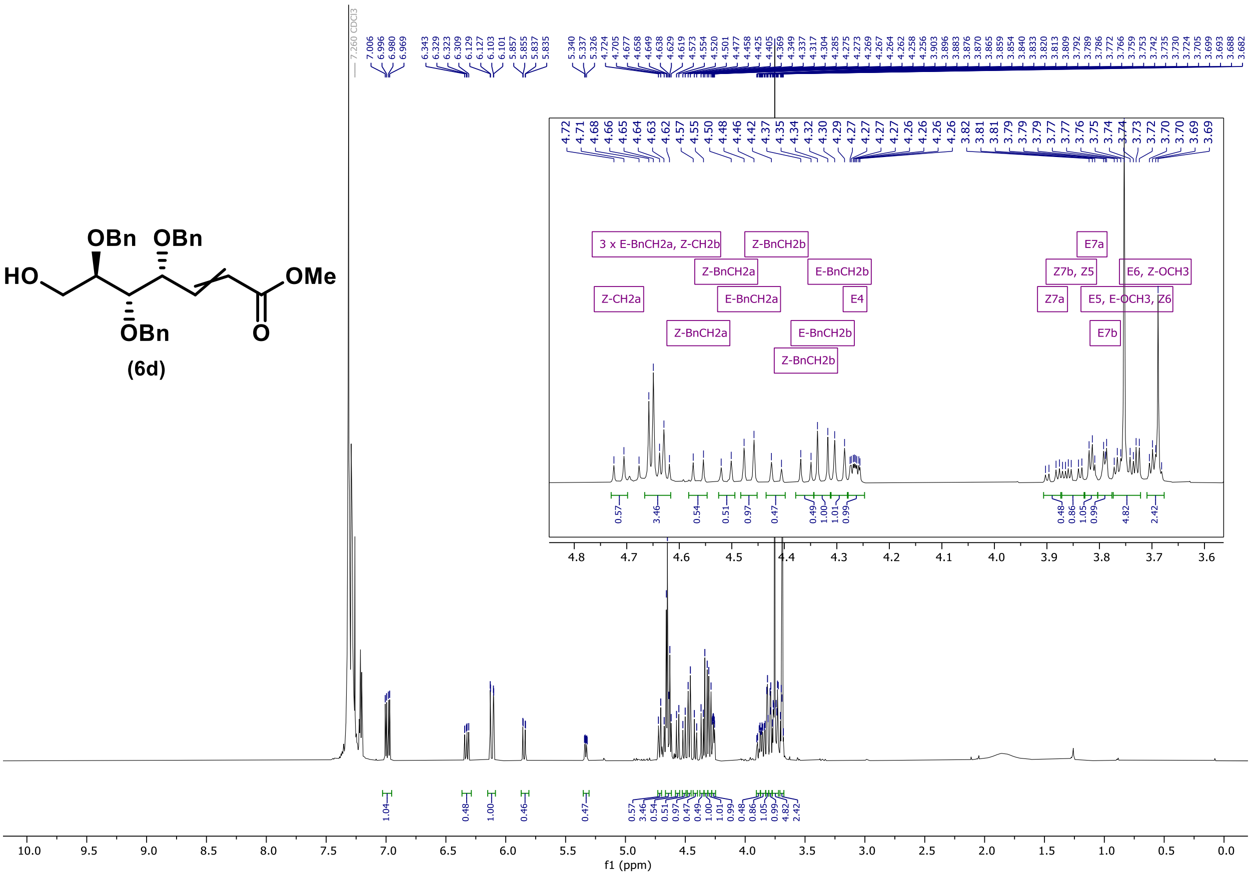

Figure S148. *600 MHz ^{1}H-NMR of (6d) as 2:1 E/Z mixture*

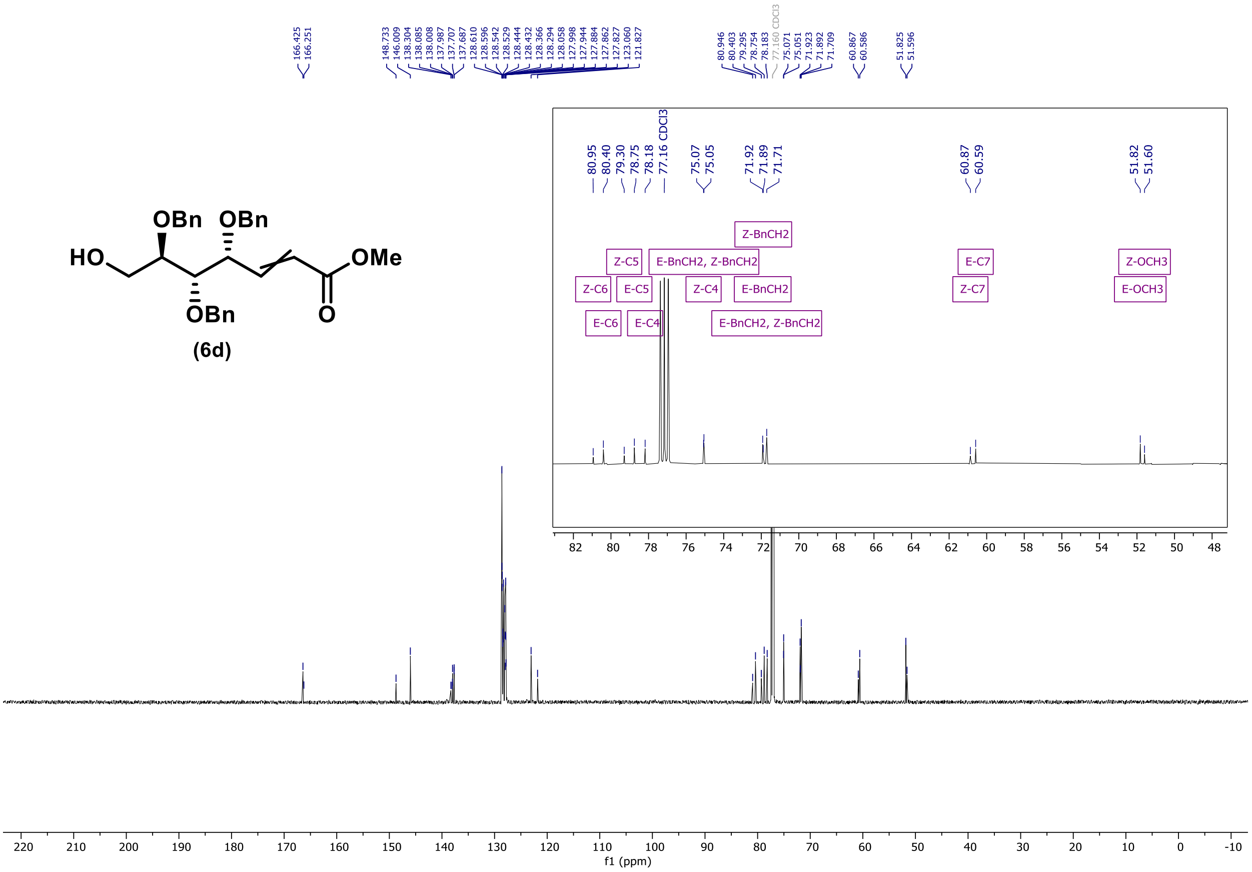

Figure S149. *151 MHz ^{13}C{^{1}H}-NMR of (6d) as 2:1 E/Z mixture*

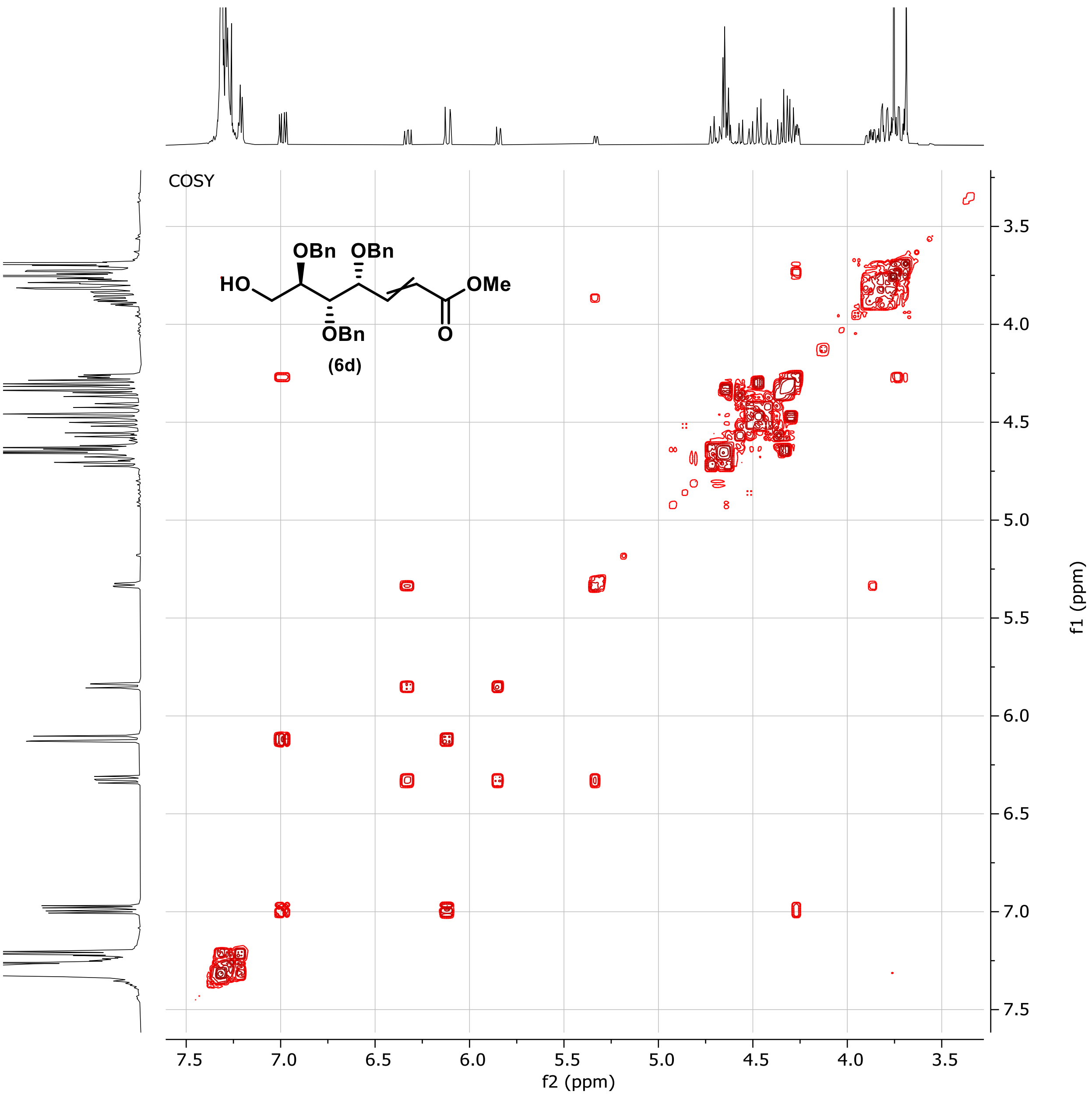

Figure S150. *COSY NMR of (6d) as 2:1 E/Z mixture*

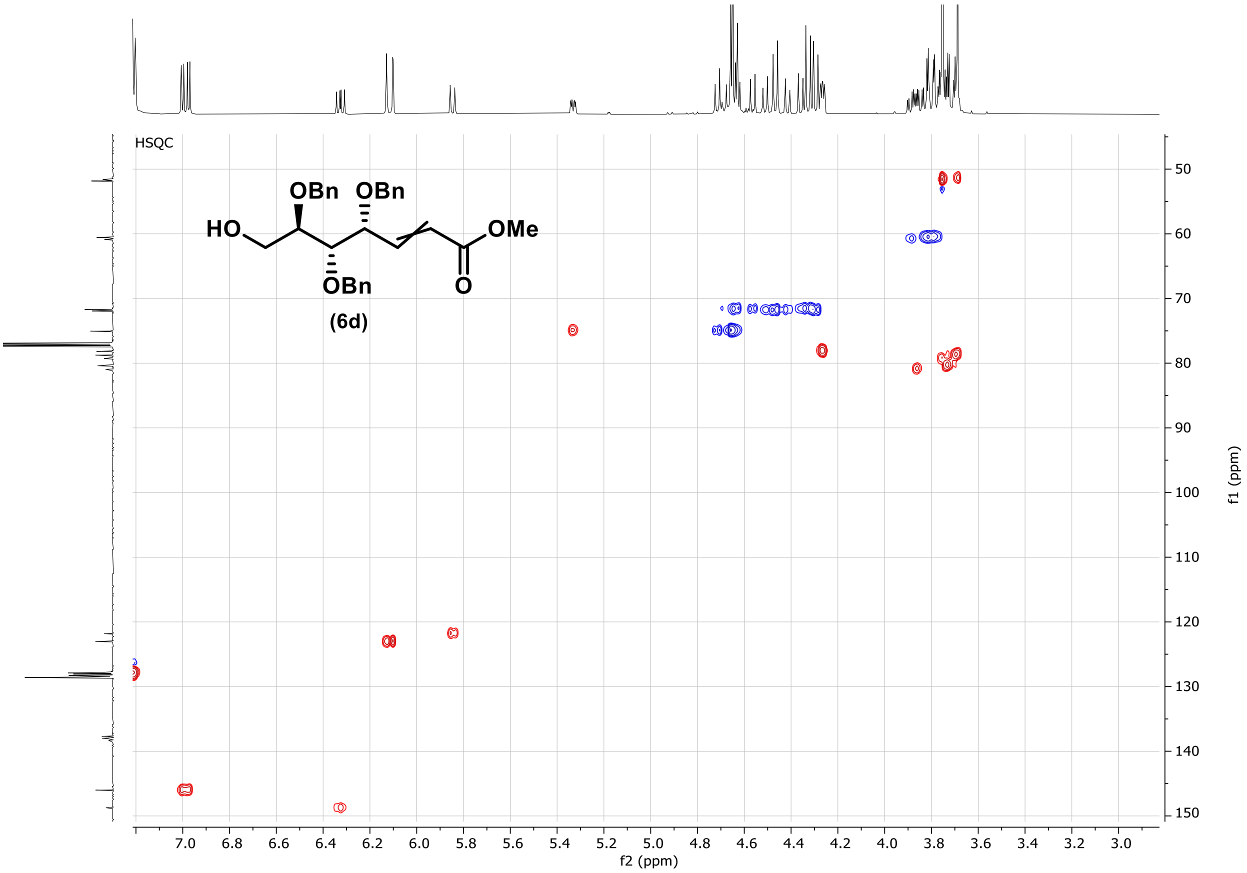

Figure S151. *HSQC NMR of (6d) as 2:1 E/Z mixture*

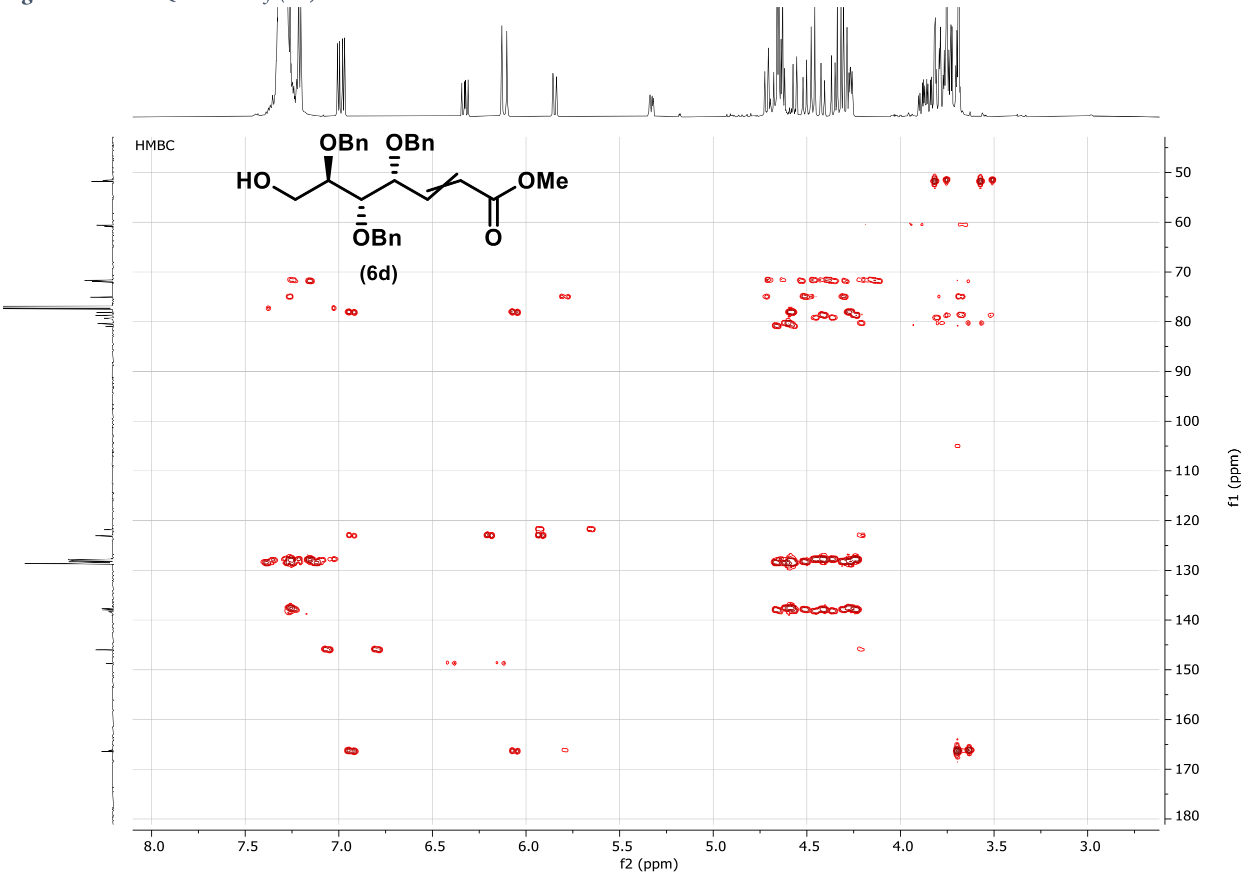

Figure S152. *HMBC NMR of (6d) as 2:1 E/Z mixture*

D.4.3. Methyl (4S, 5S, 6S, E)-4,5,6-tris(benzyloxy)-7-oxo-hept-2-enoate (1d)

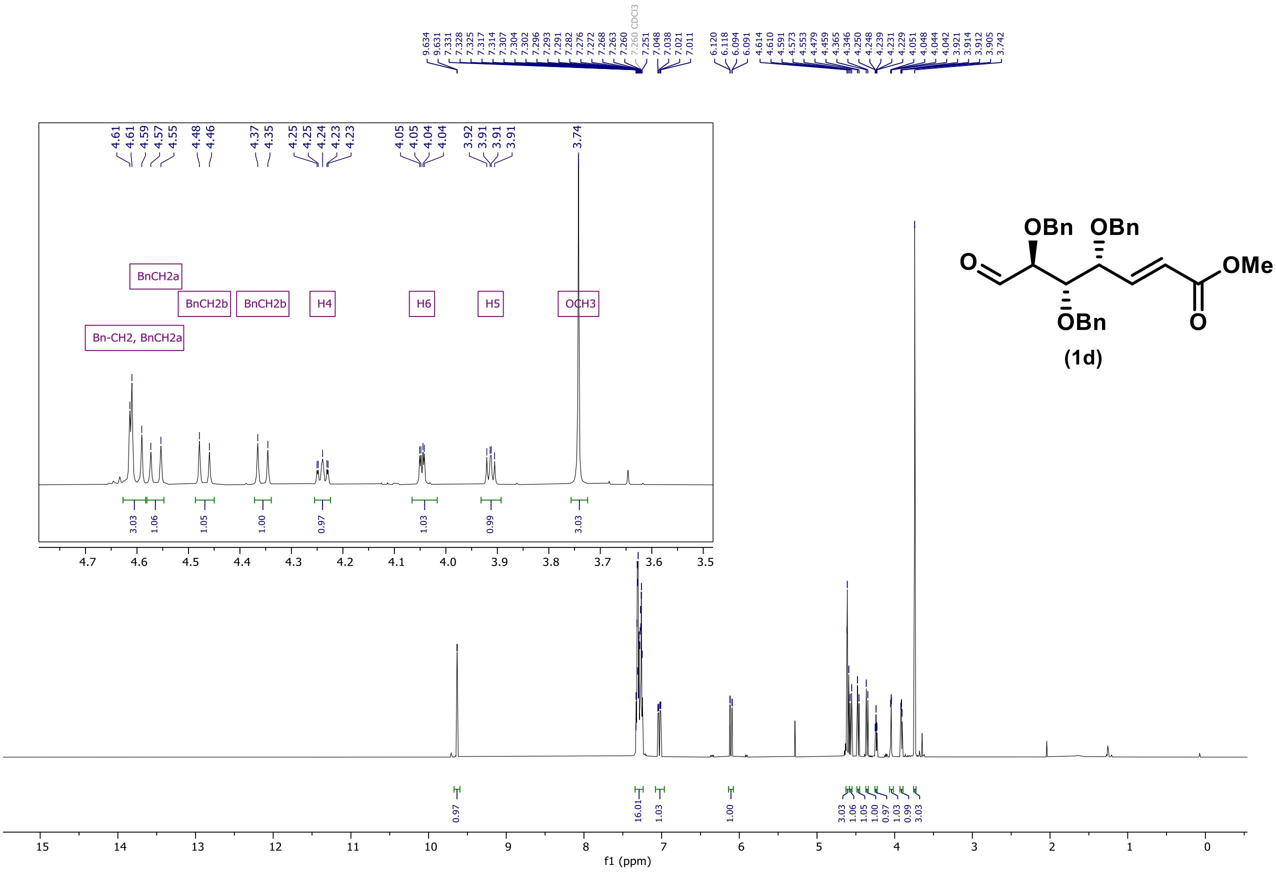

Figure S153. *600 MHz ^{1}H-NMR of (1d)*

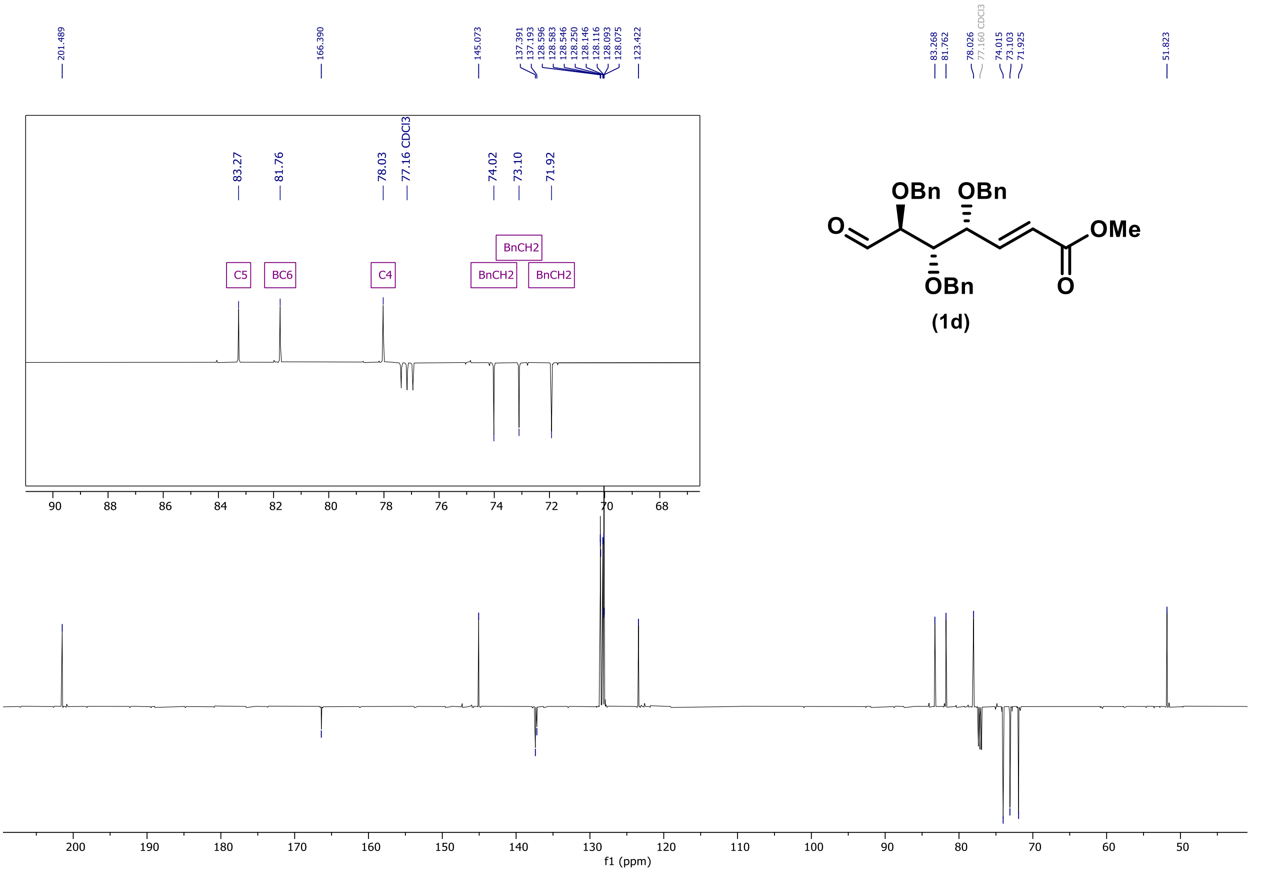

Figure S154. *151 MHz $^{13}C\{^{1}H\}$-NMR of (1d)*

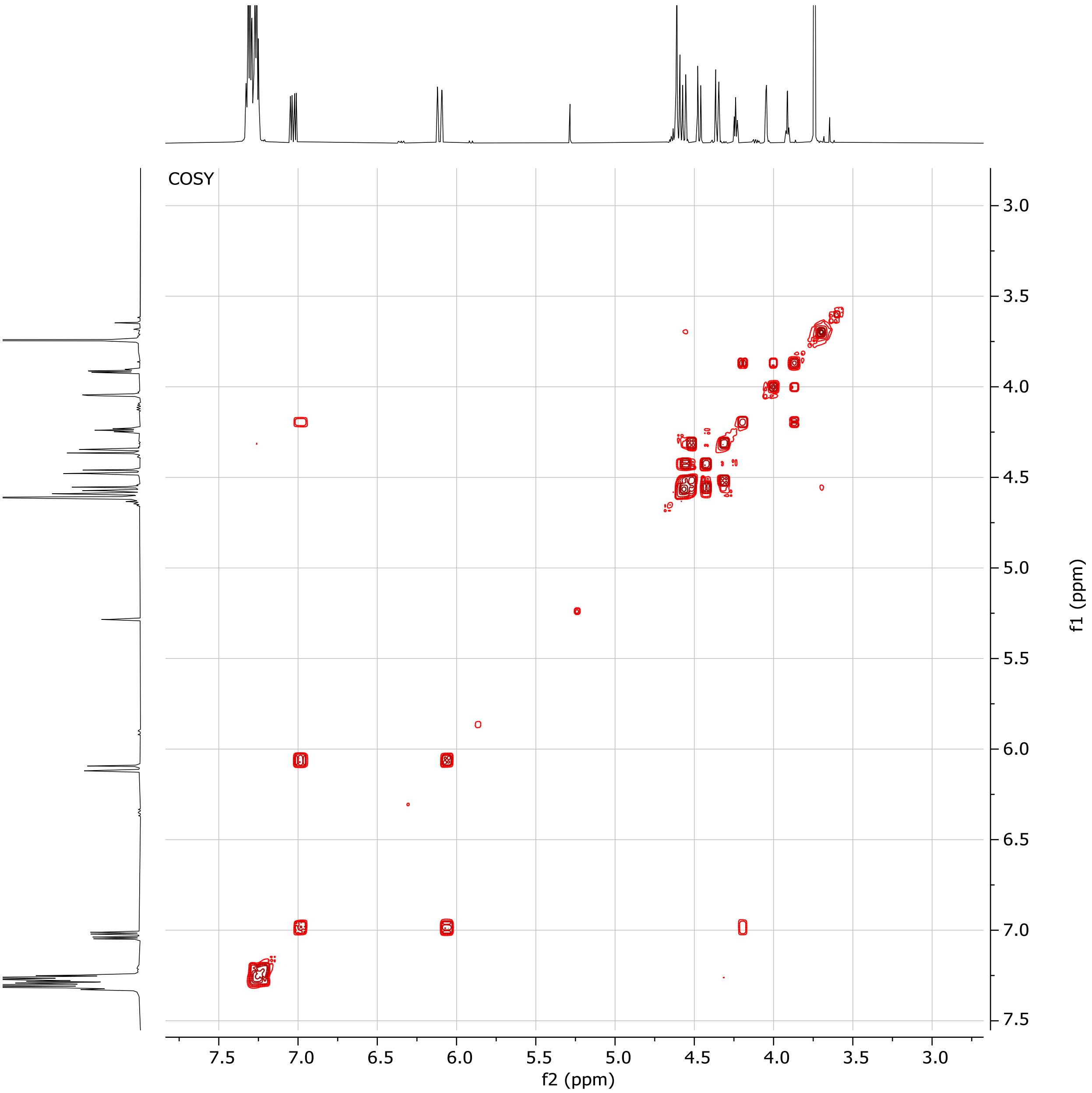

Figure S155. *COSY NMR of (1d)*

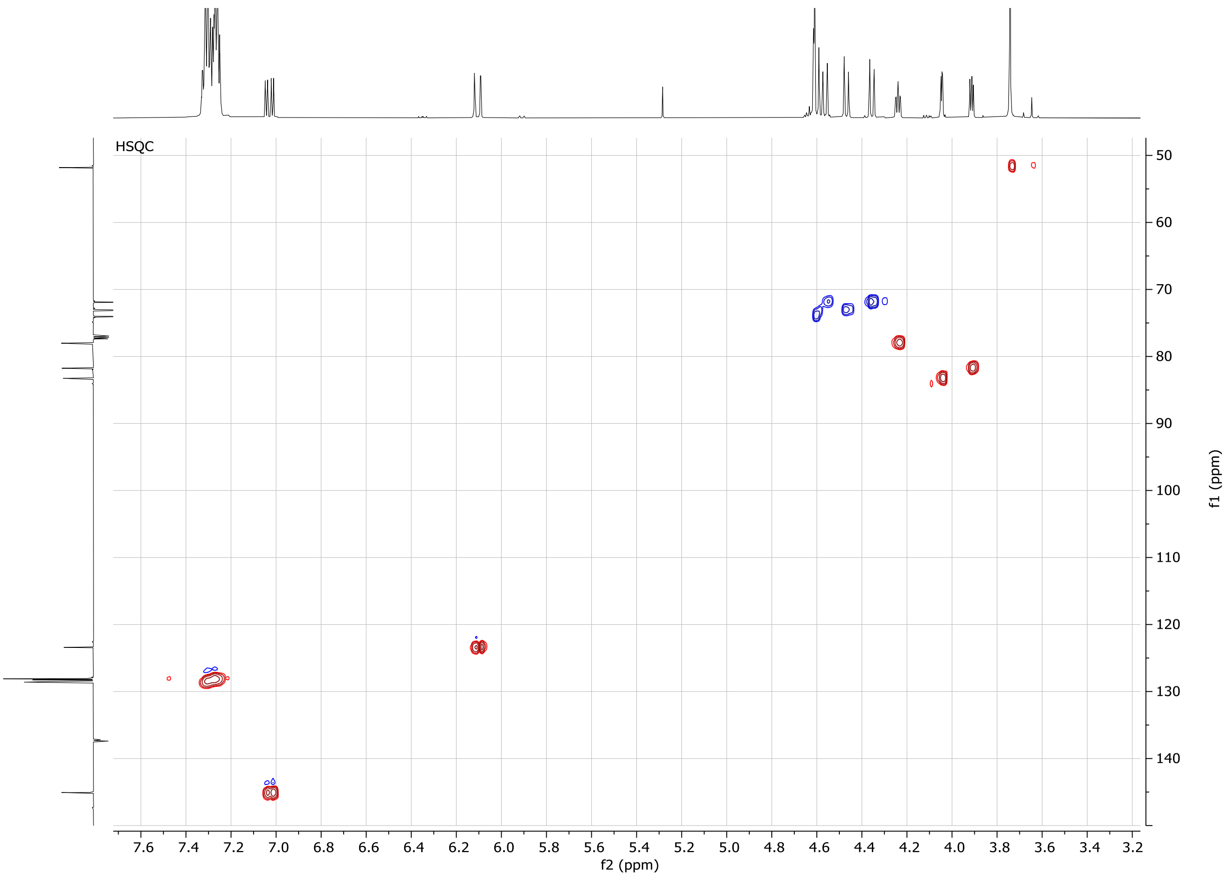

Figure S156. HSQC NMR of (1d)

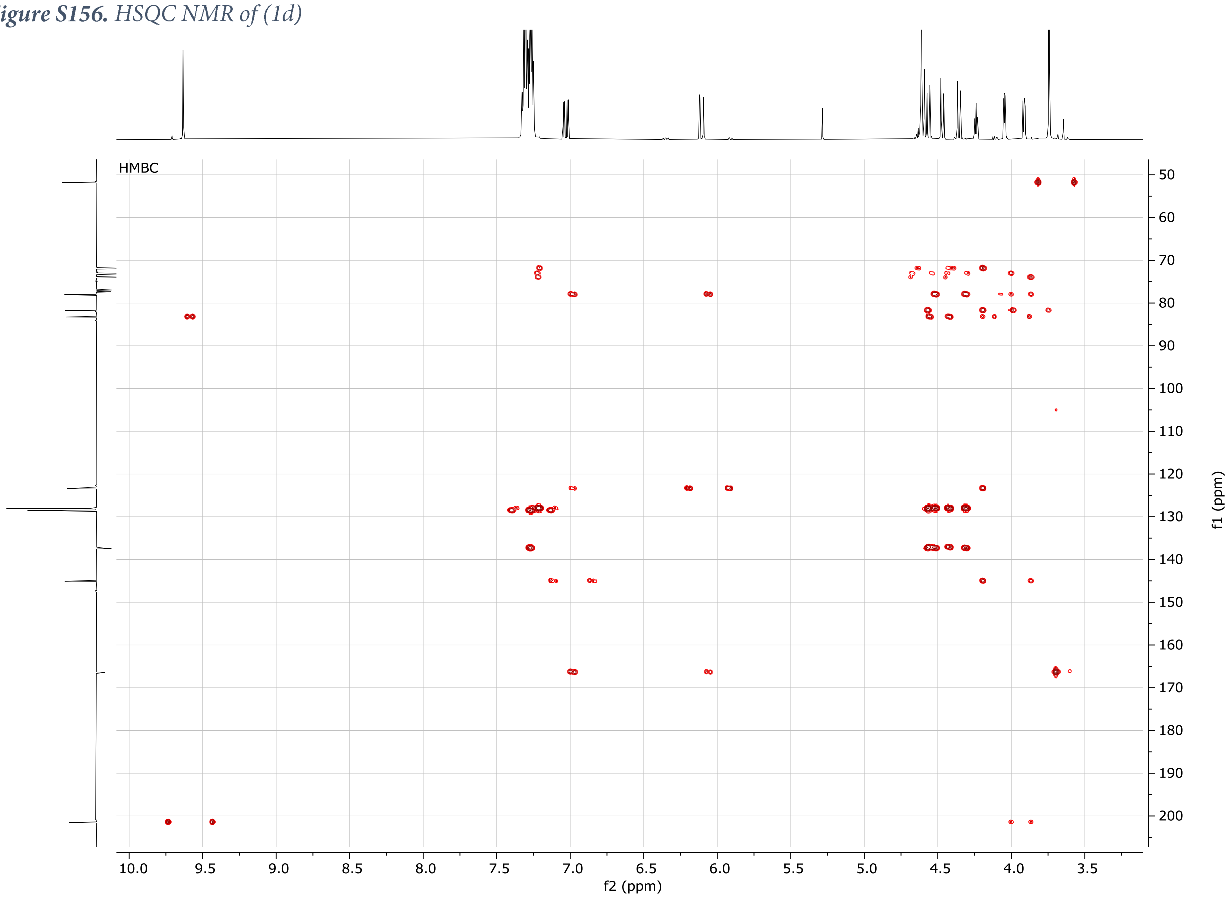

Figure S157. HMBC NMR of (1d)

D.5. Carbocycles

D.5.1. Xylose derived Carbocycles

D.5.1.1. Methyl [(3S,4R,5S)-3,4,5-tris(benzyloxy)-2-oxocyclopentyl]acetate (2a)

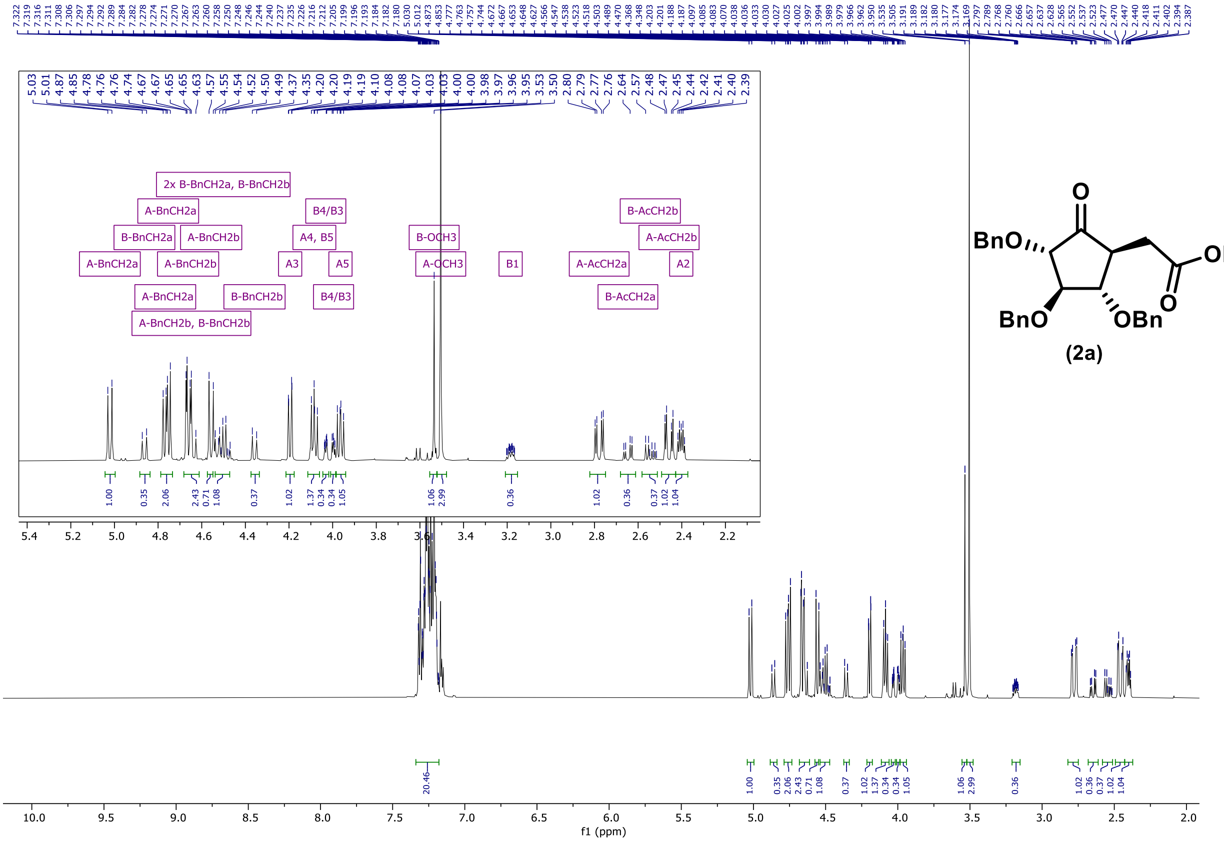

Figure S158. *600 MHz ^{1}H-NMR of (2a) as a anti:syn mixture 3:1*

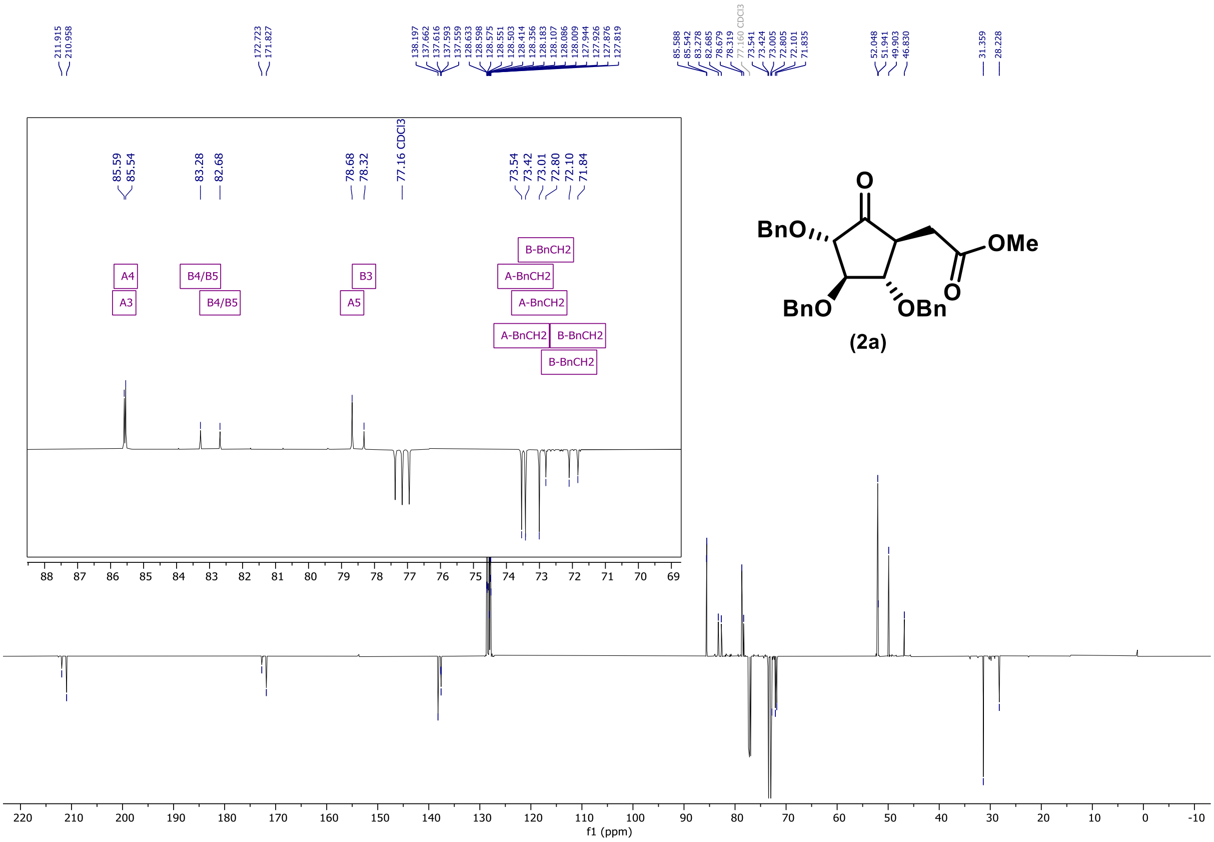

Figure S159. *151 MHz $^{13}C\{^1H\}$-NMR of (2a) as a anti:syn mixture 3:1*

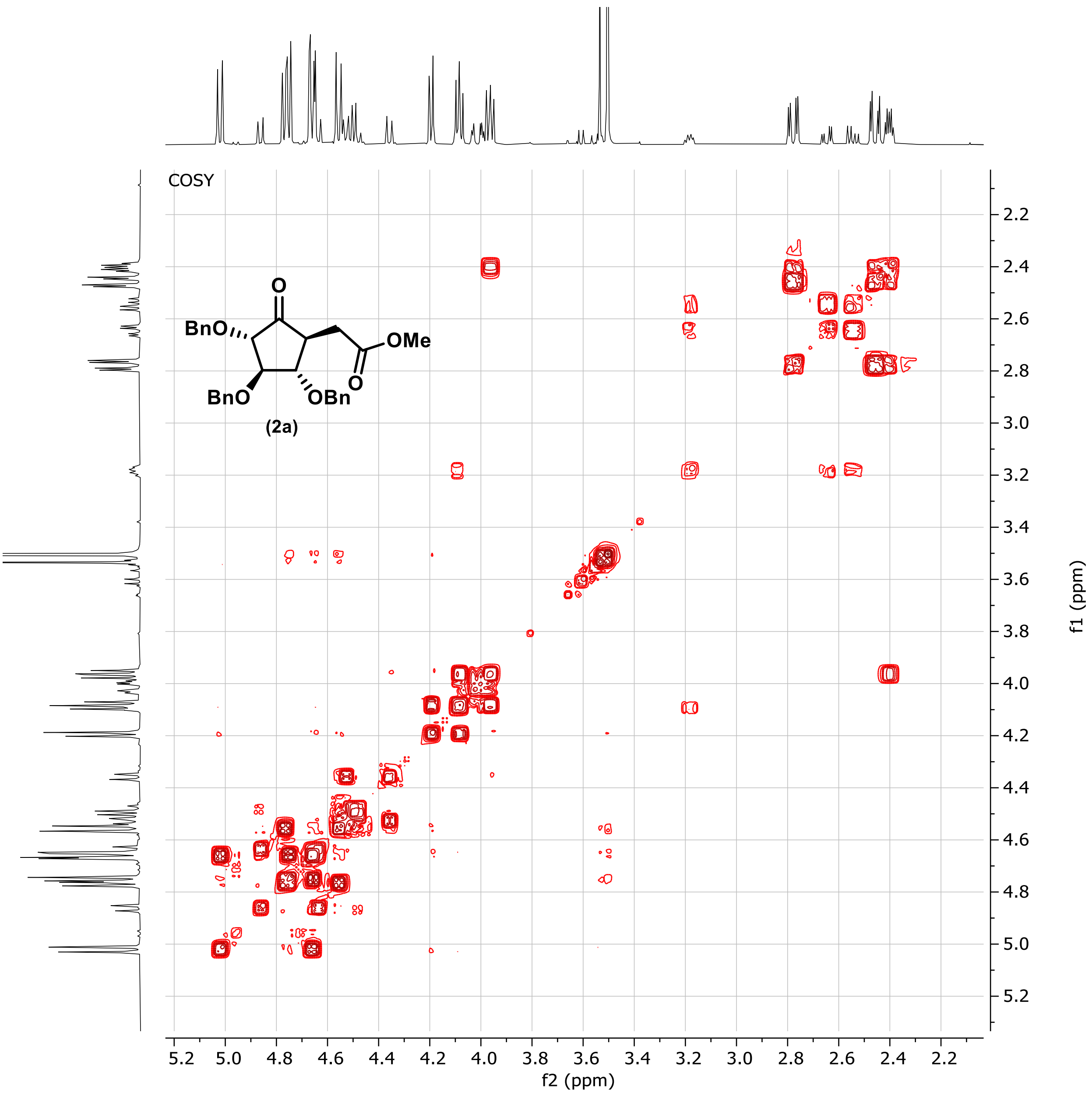

__Figure S160.__ COSY NMR of (2a)

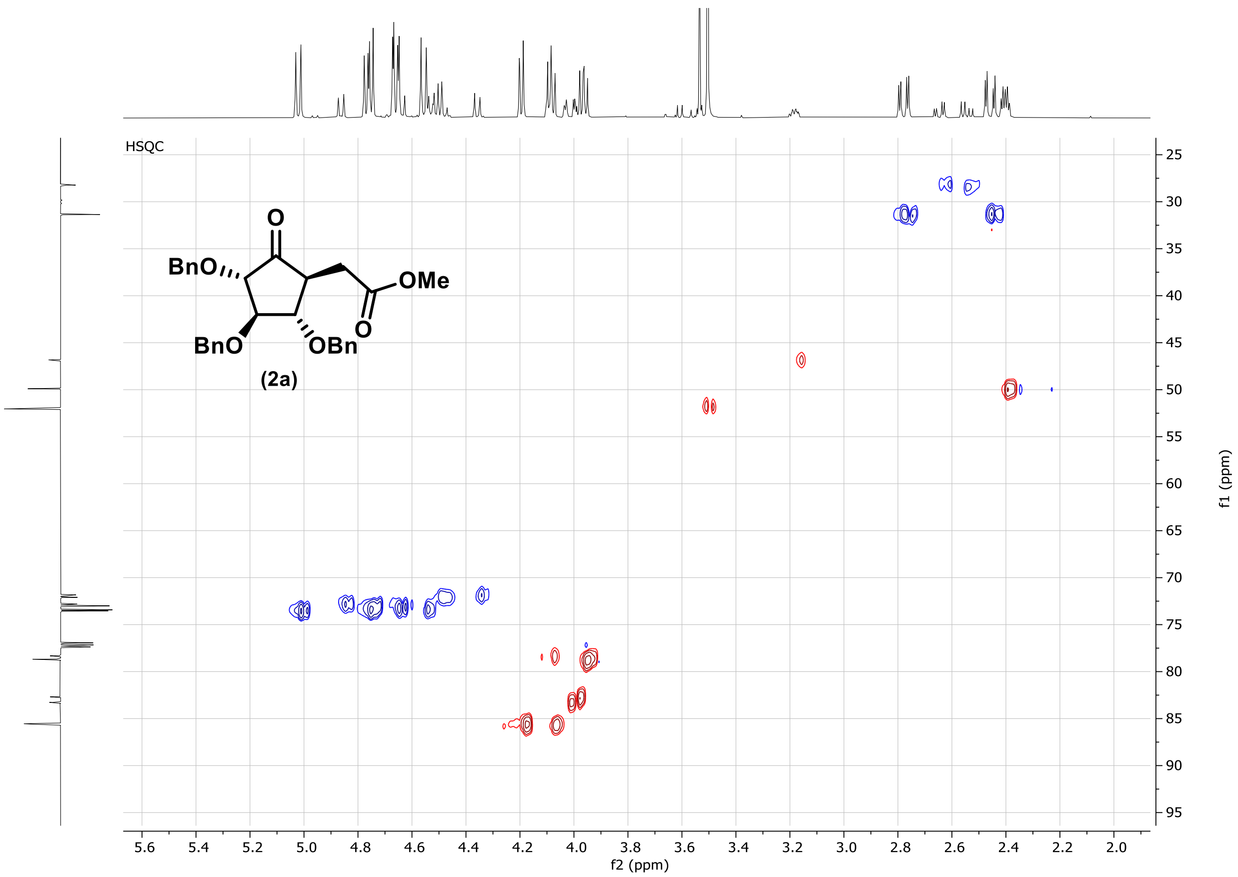

Figure S161. HSQC NMR of (2a)

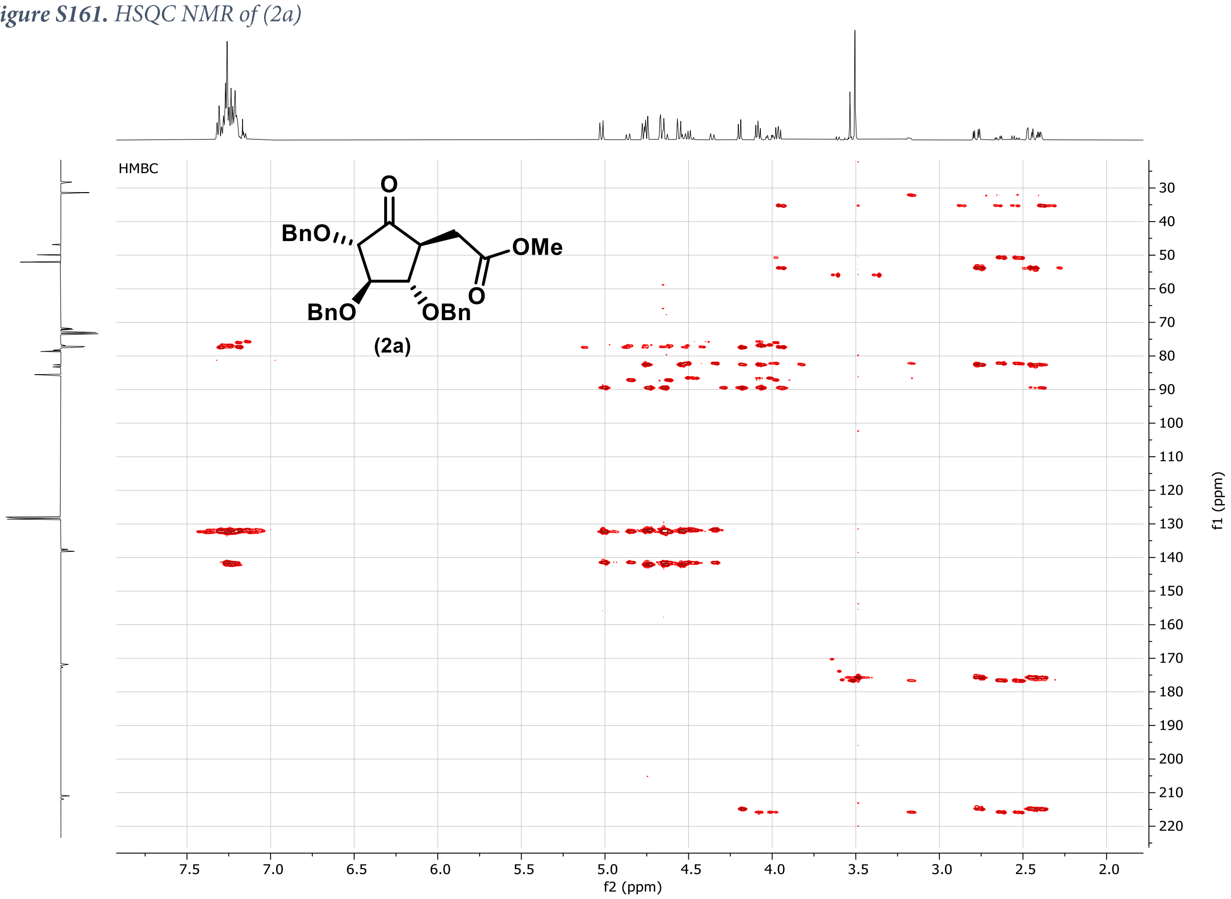

Figure S162. HMBC NMR of (2a)

D.5.1.2. Methyl [(3R,4S)-3,4-bis(benzyloxy)-5-oxocyclopent-1-en-1-yl]acetate (3a)

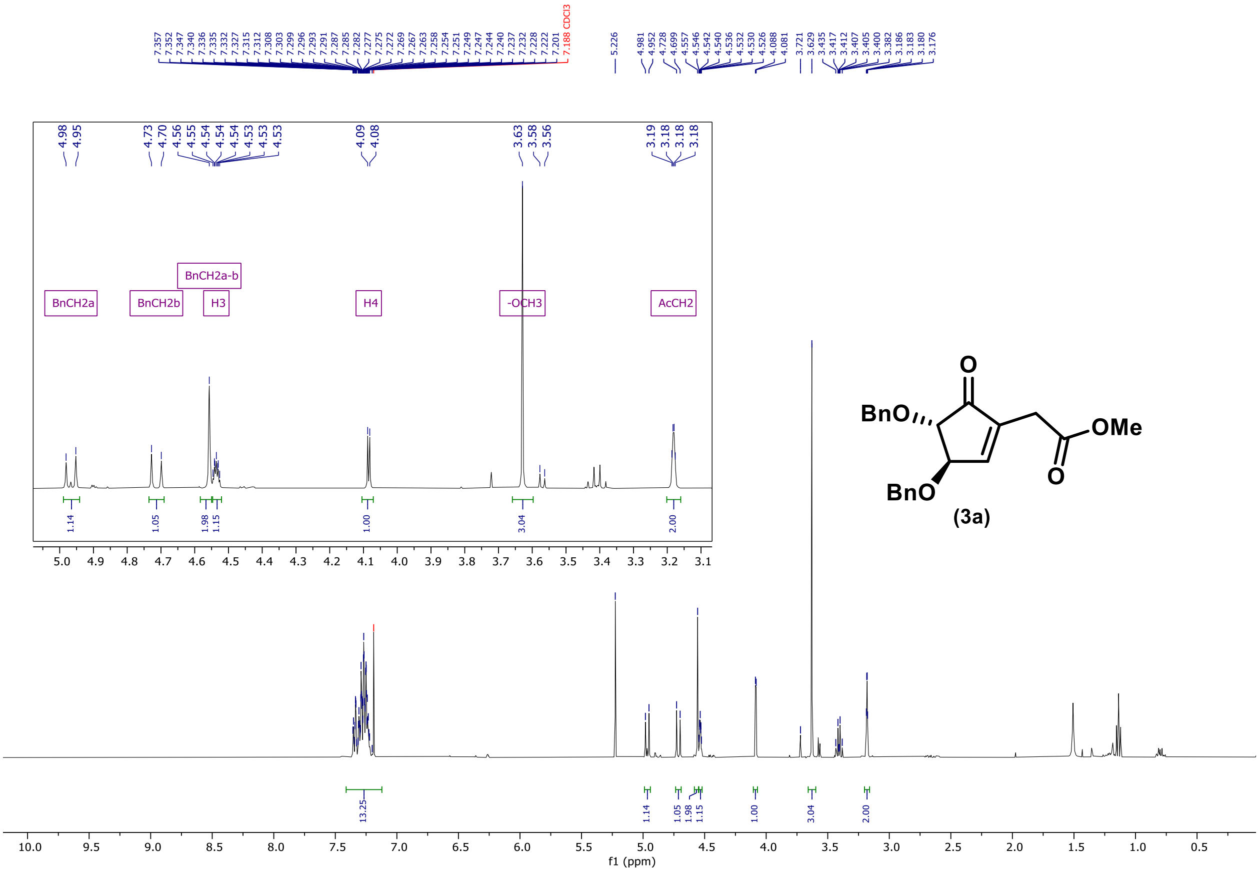

Figure S163. *400 MHz ¹H-NMR of (3a)*

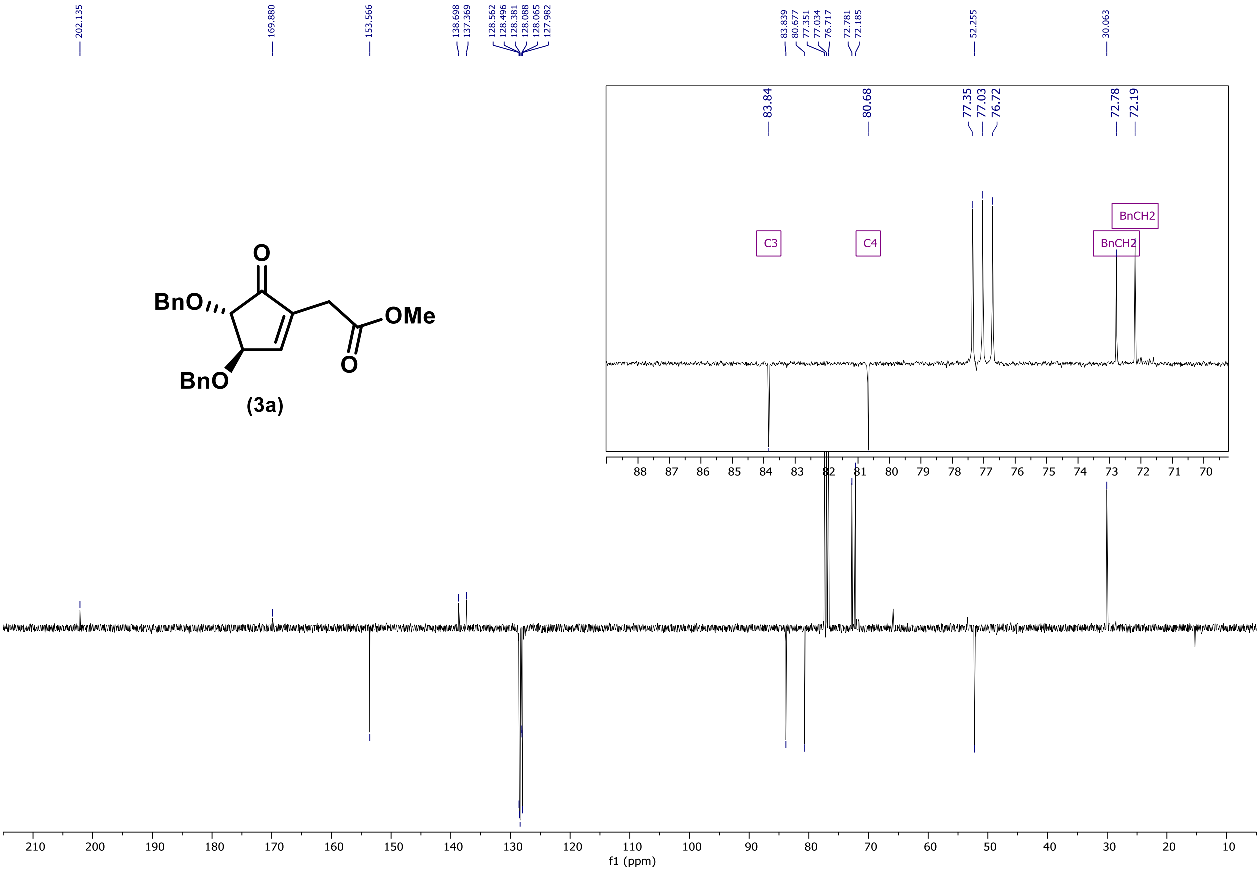

Figure S164. *101 MHz $^{13}C\{^{1}H\}$-NMR of (3xl)*

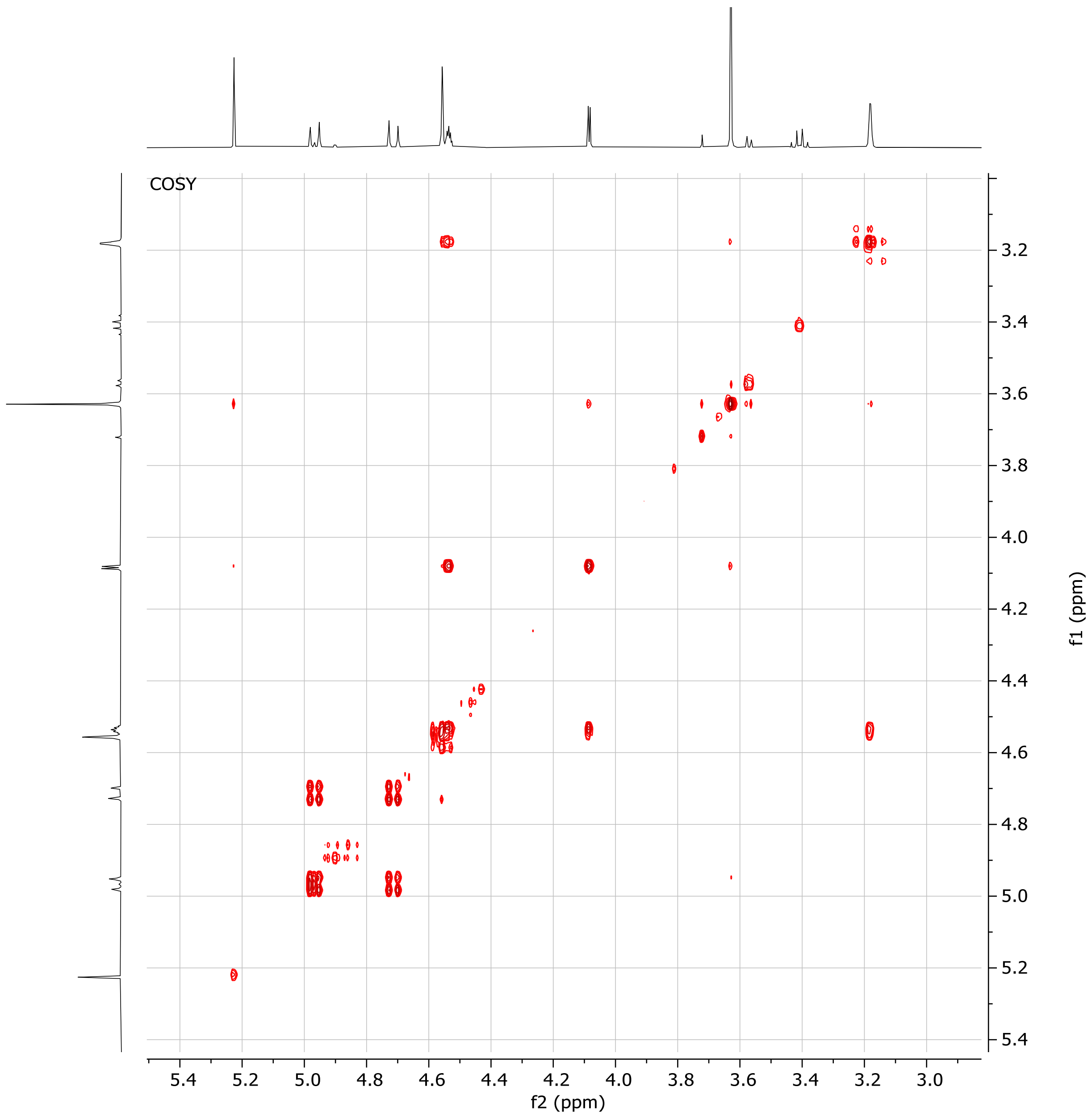

Figure S165. *COSY NMR of (6d)*

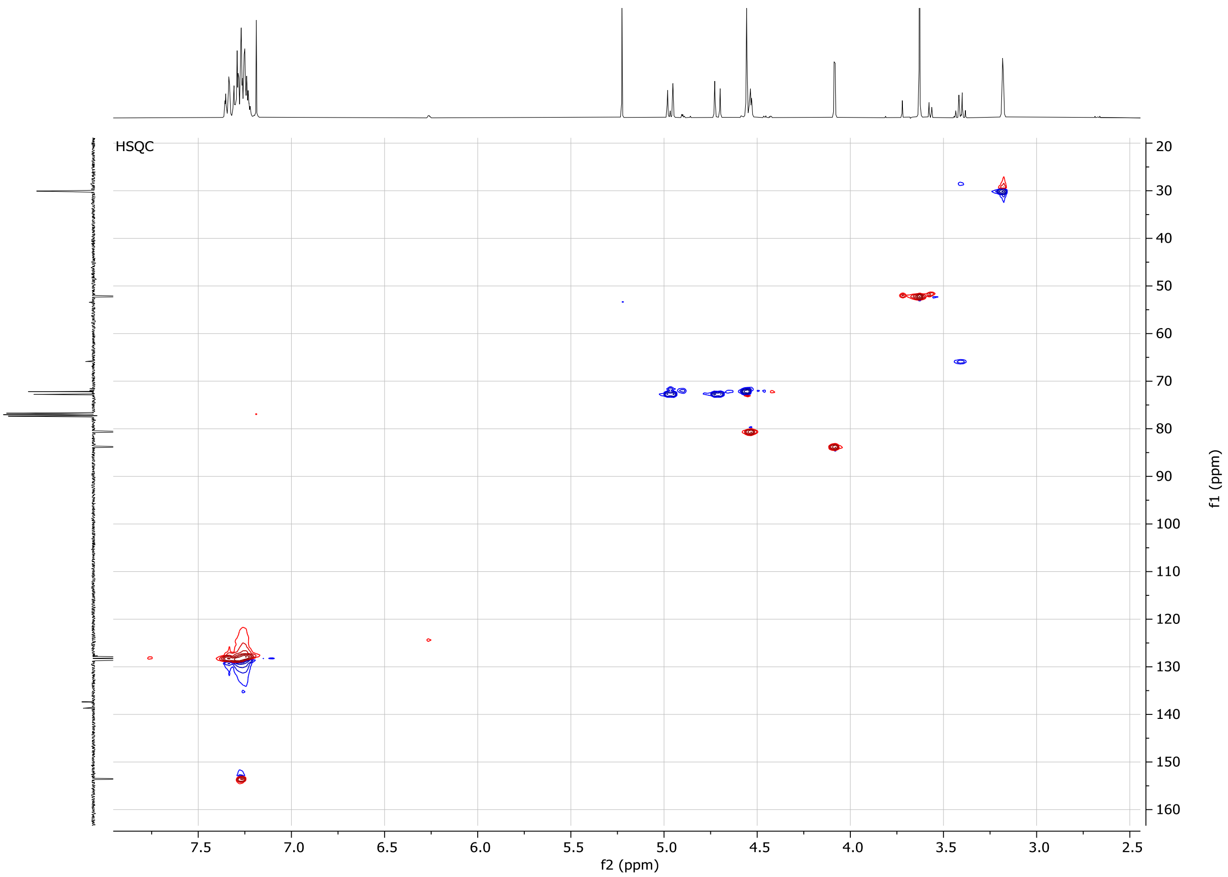

Figure S166. *HSQC NMR of (6d)*

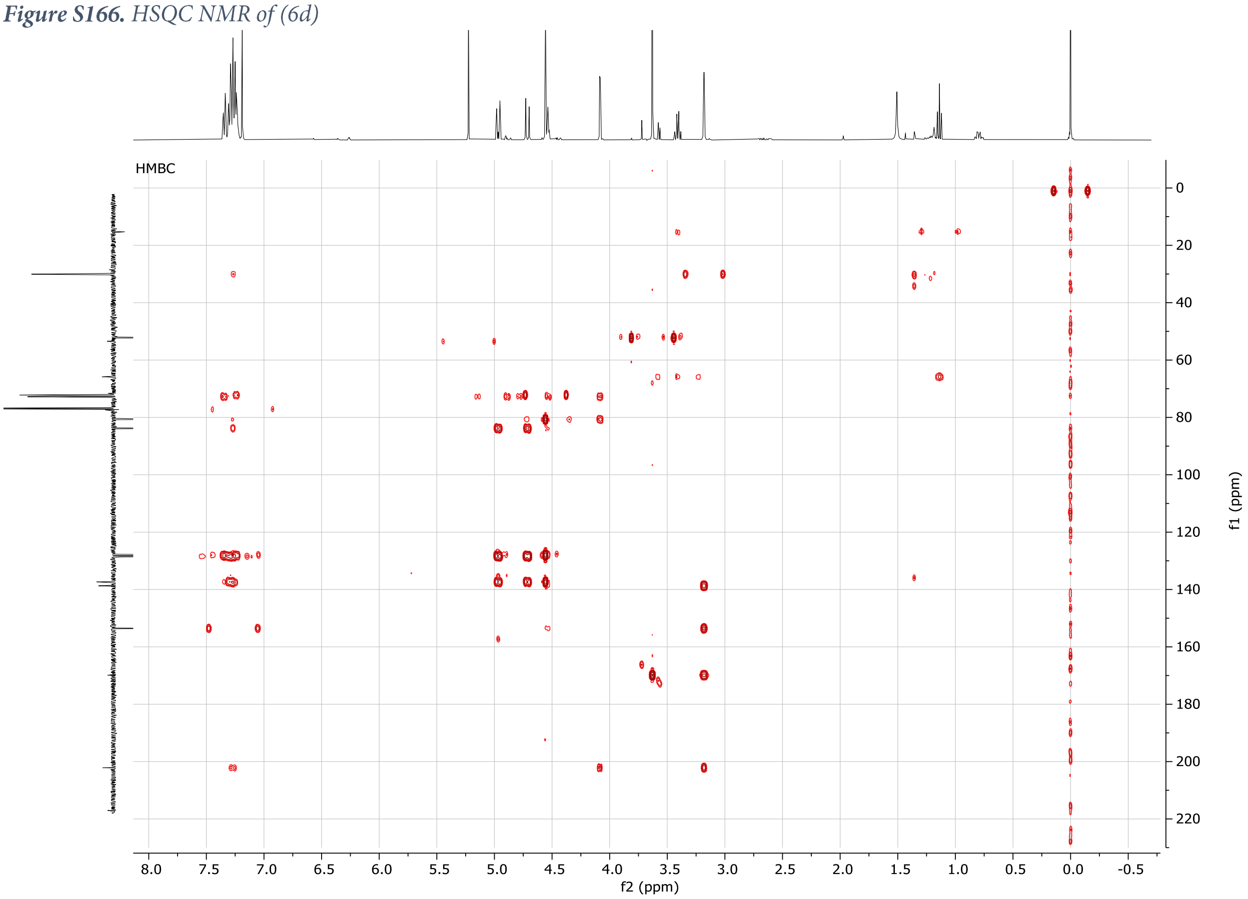

Figure S167. *HMBC NMR of (6d)*

D.5.1.3. Ethyl [(3S,4R,5S)-3,4,5-tris(benzyloxy)-2-oxocyclopentyl]acetate (2f)

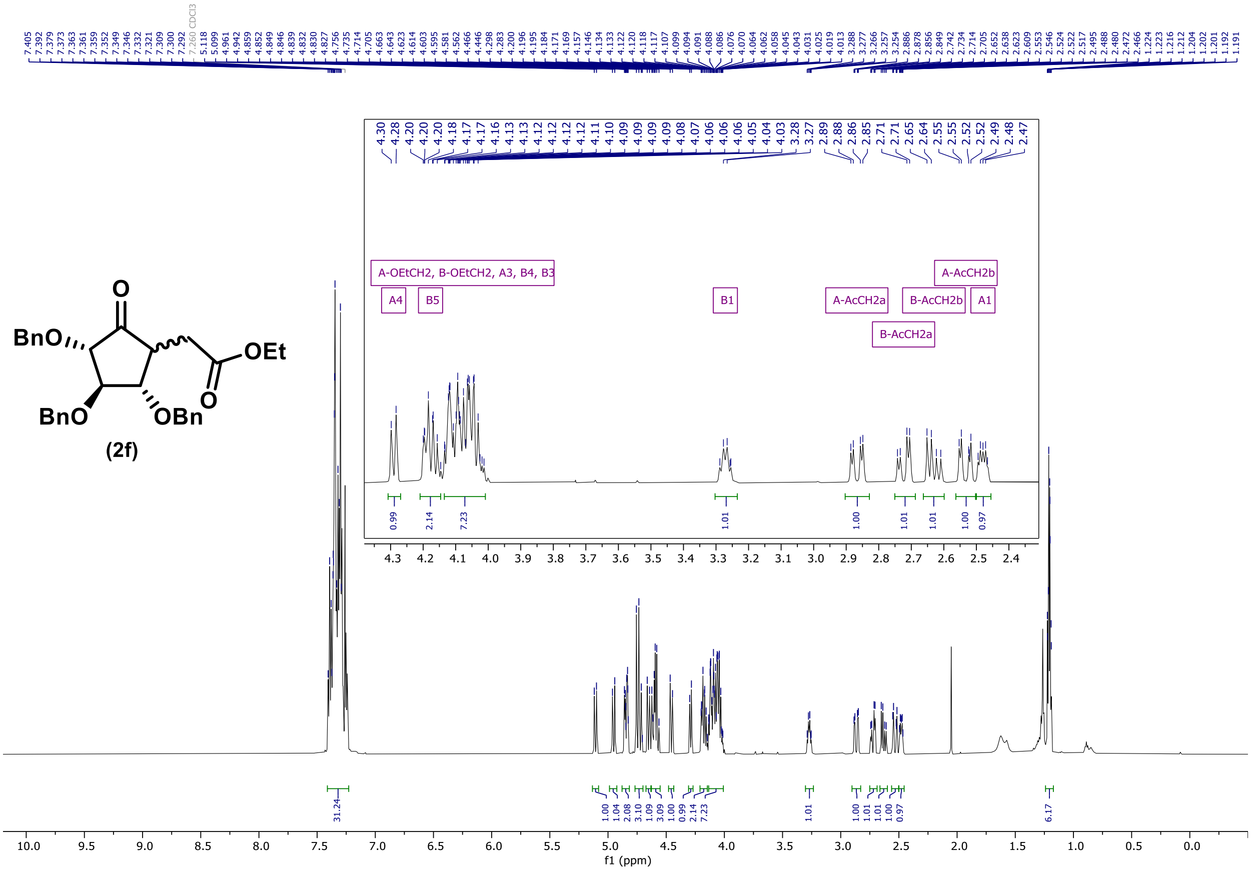

Figure S168. 600 MHz ^{1}H-NMR of (2f)

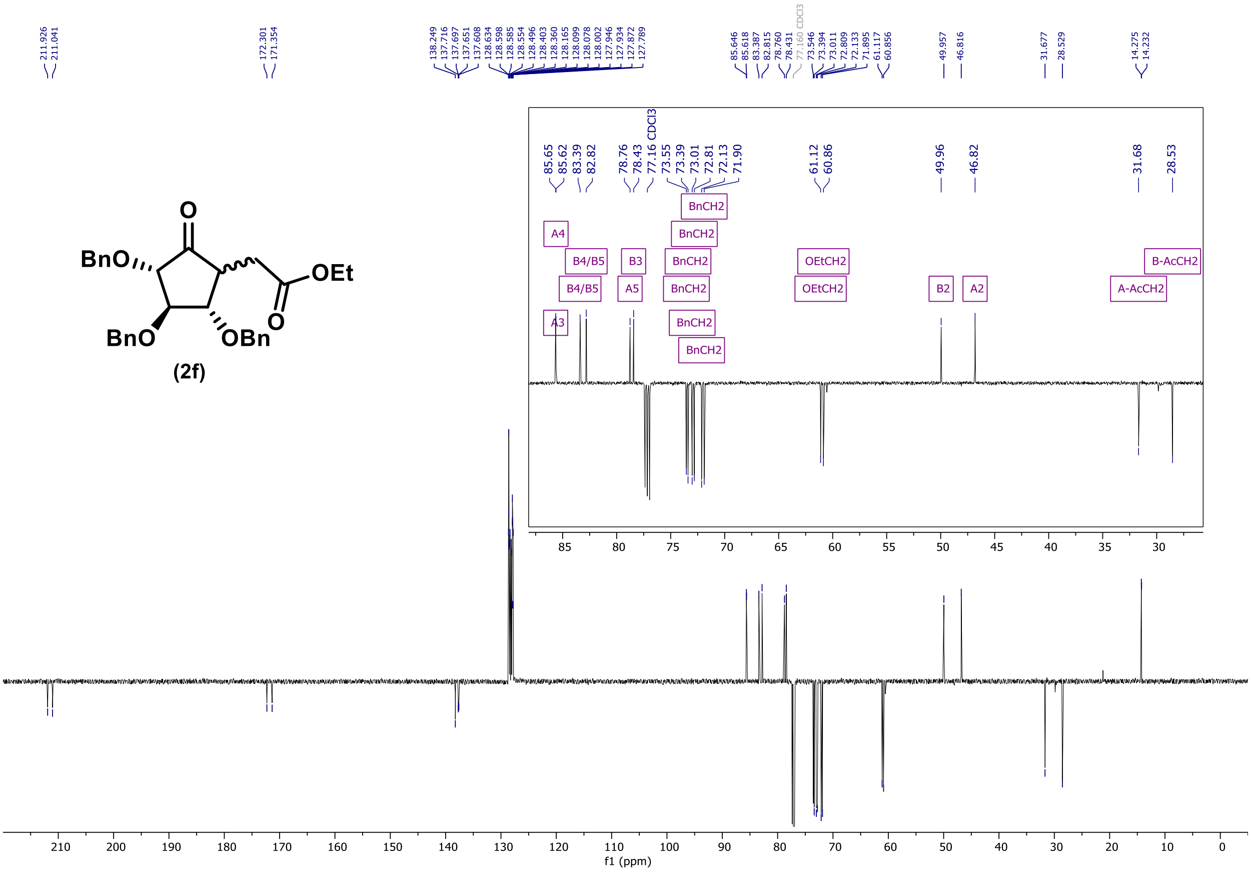

Figure S169. 151 MHz $^{13}C\{^1H\}$-NMR of (2f)

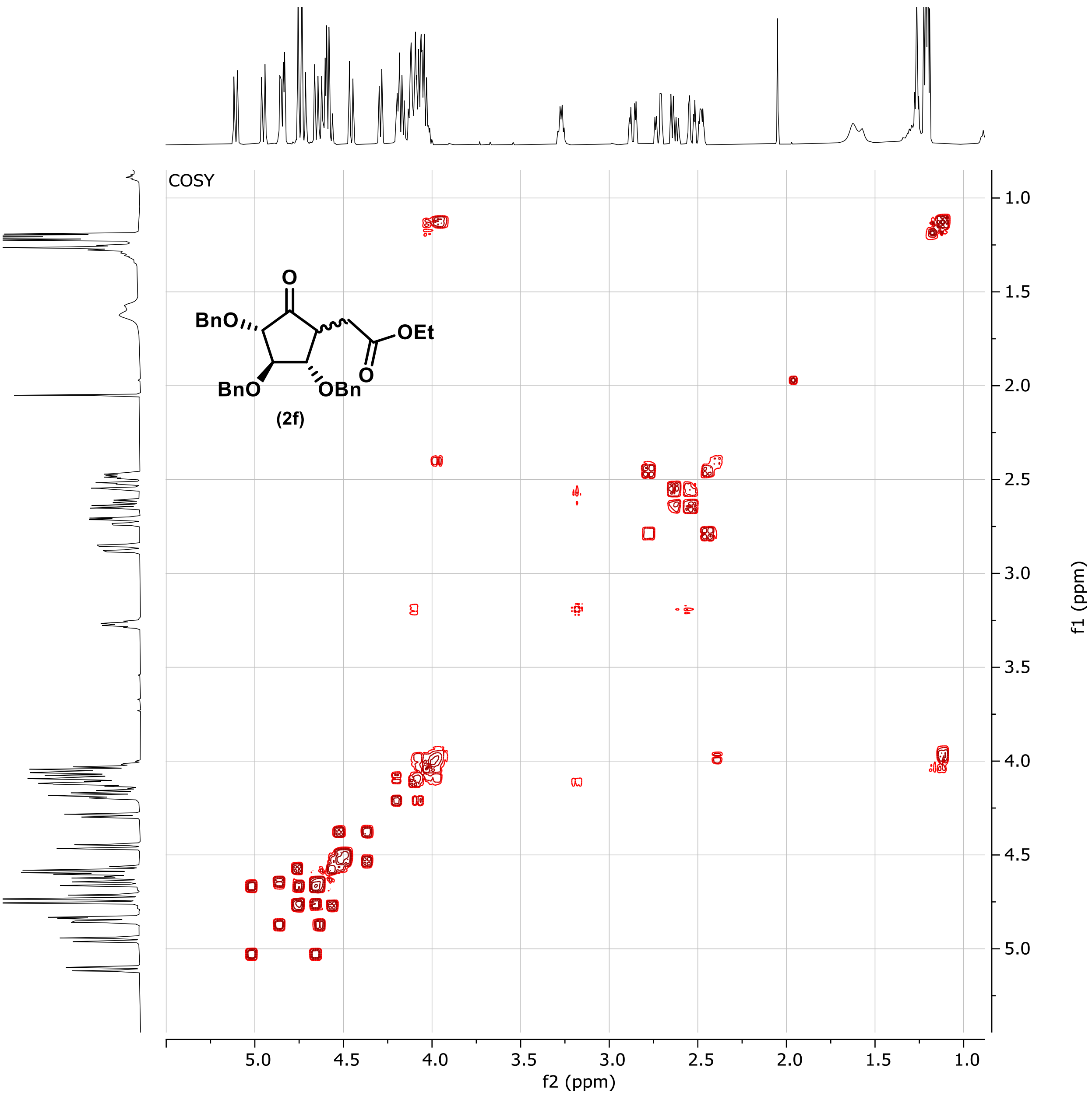

Figure S170. COSY NMR of (2f)

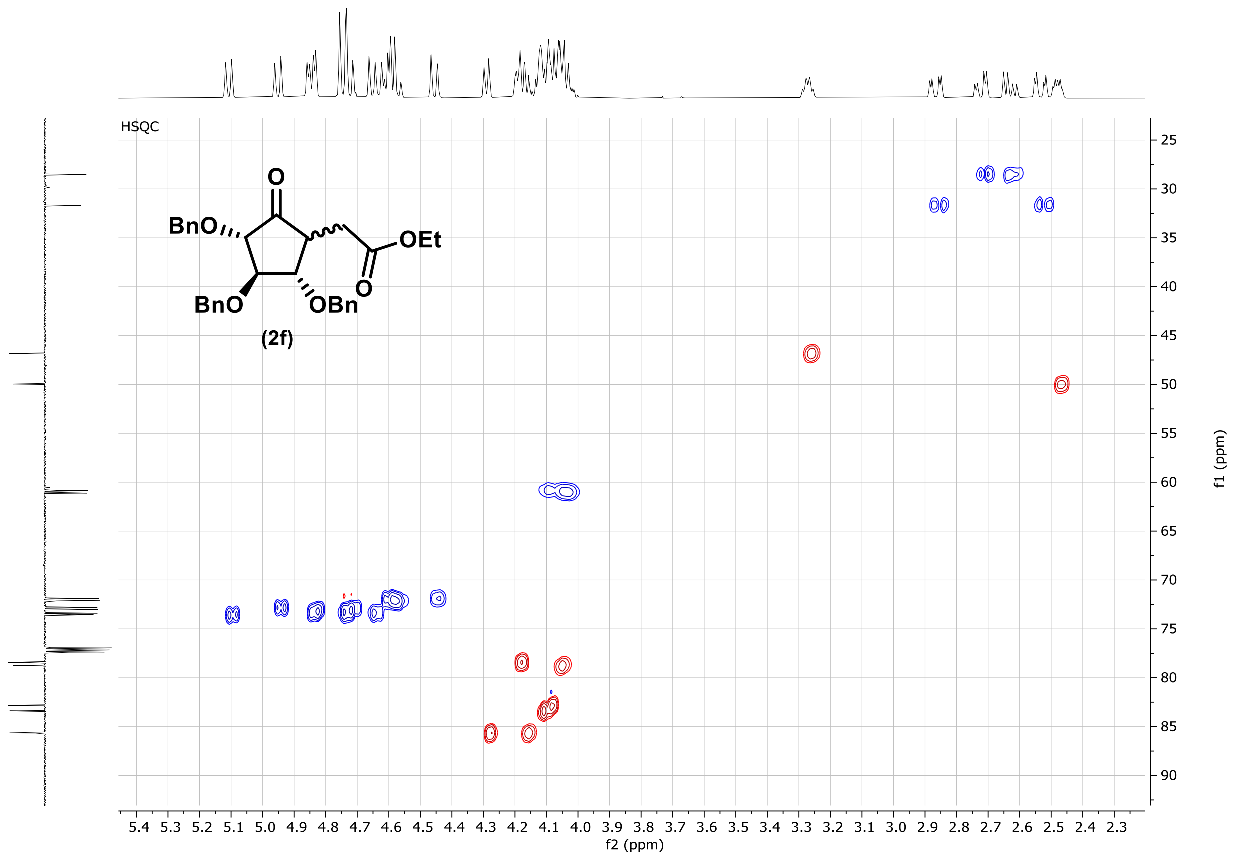

Figure S171. HSQC NMR of (2f)

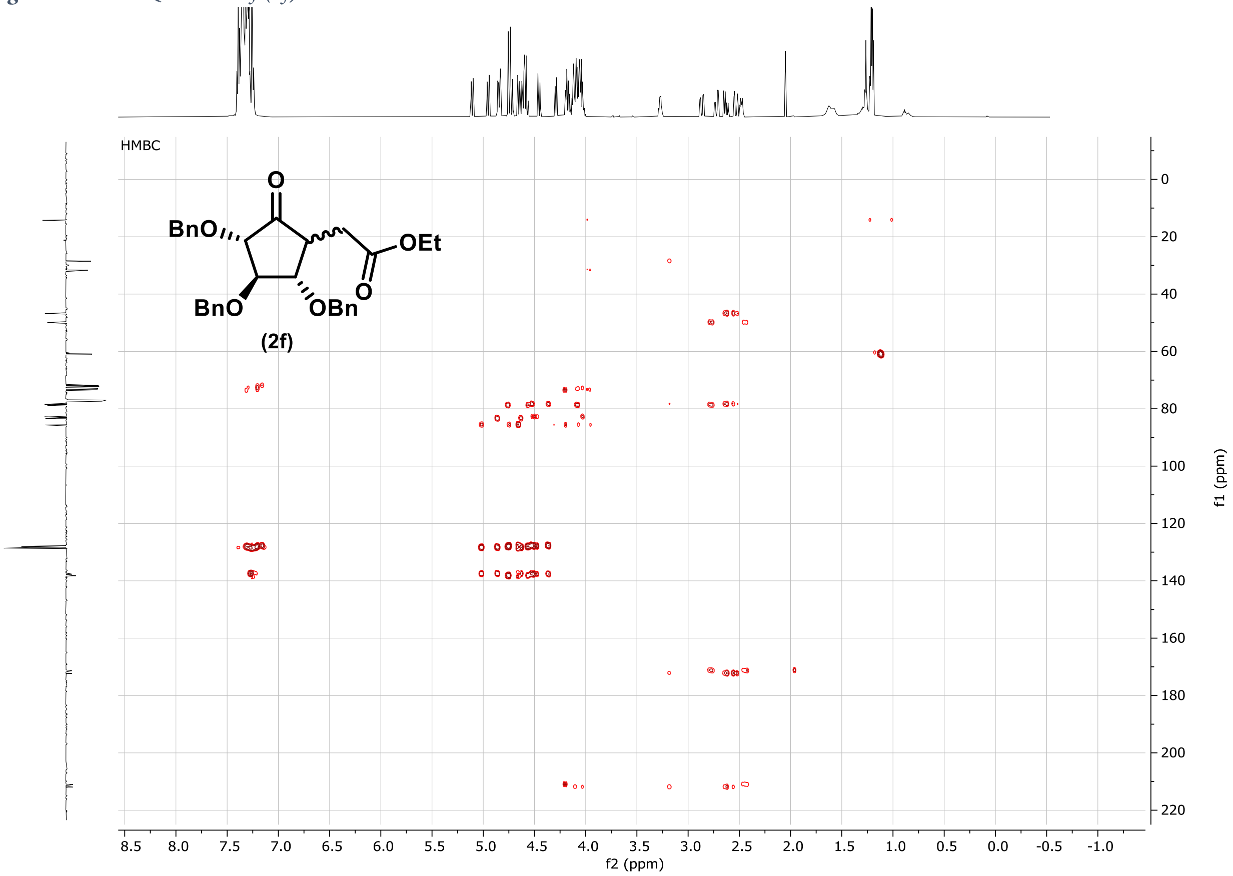

Figure S172. HMBC NMR of (2f)

D.5.1.4. Ethyl [(3R,4S)-3,4-bis(benzyloxy)-5-oxocyclopent-1-en-1-yl]acetate (3f)

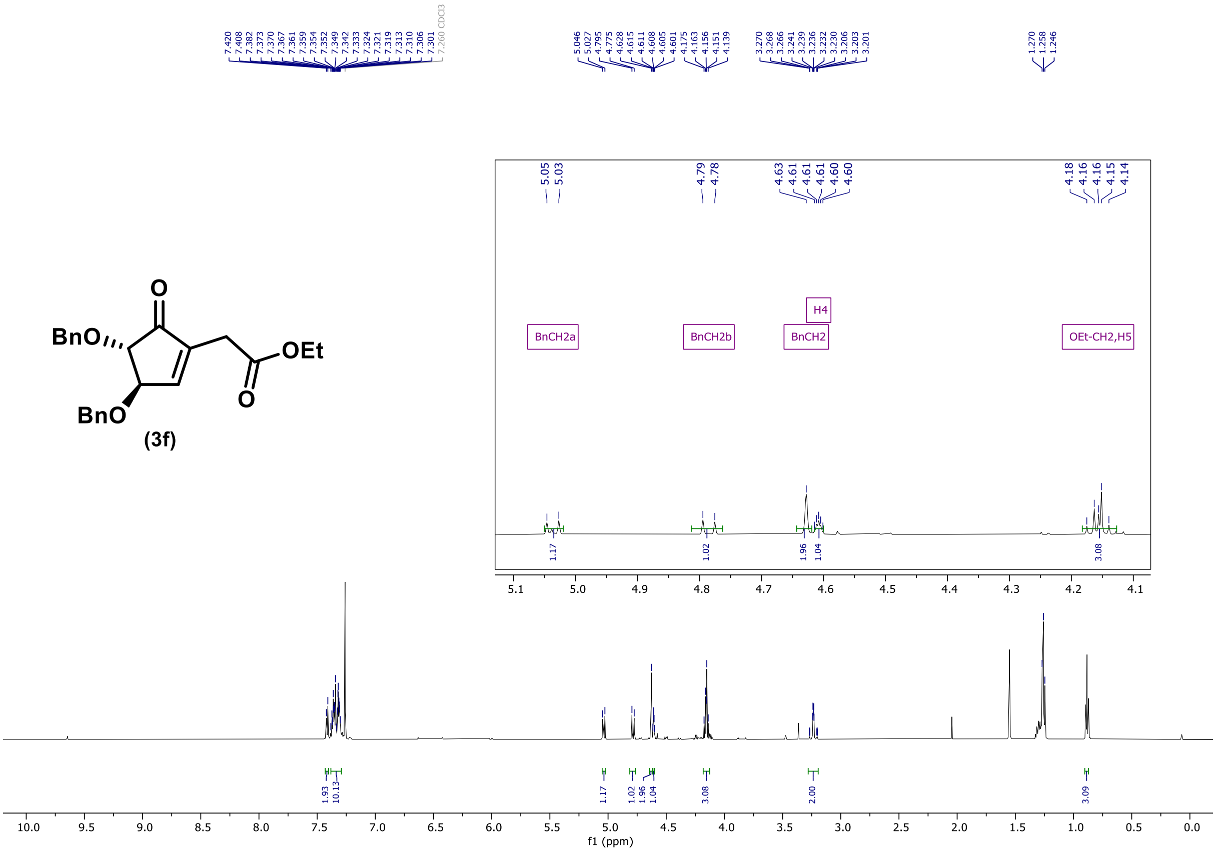

Figure S173. *600 MHz ^{1}H-NMR of (3f)*

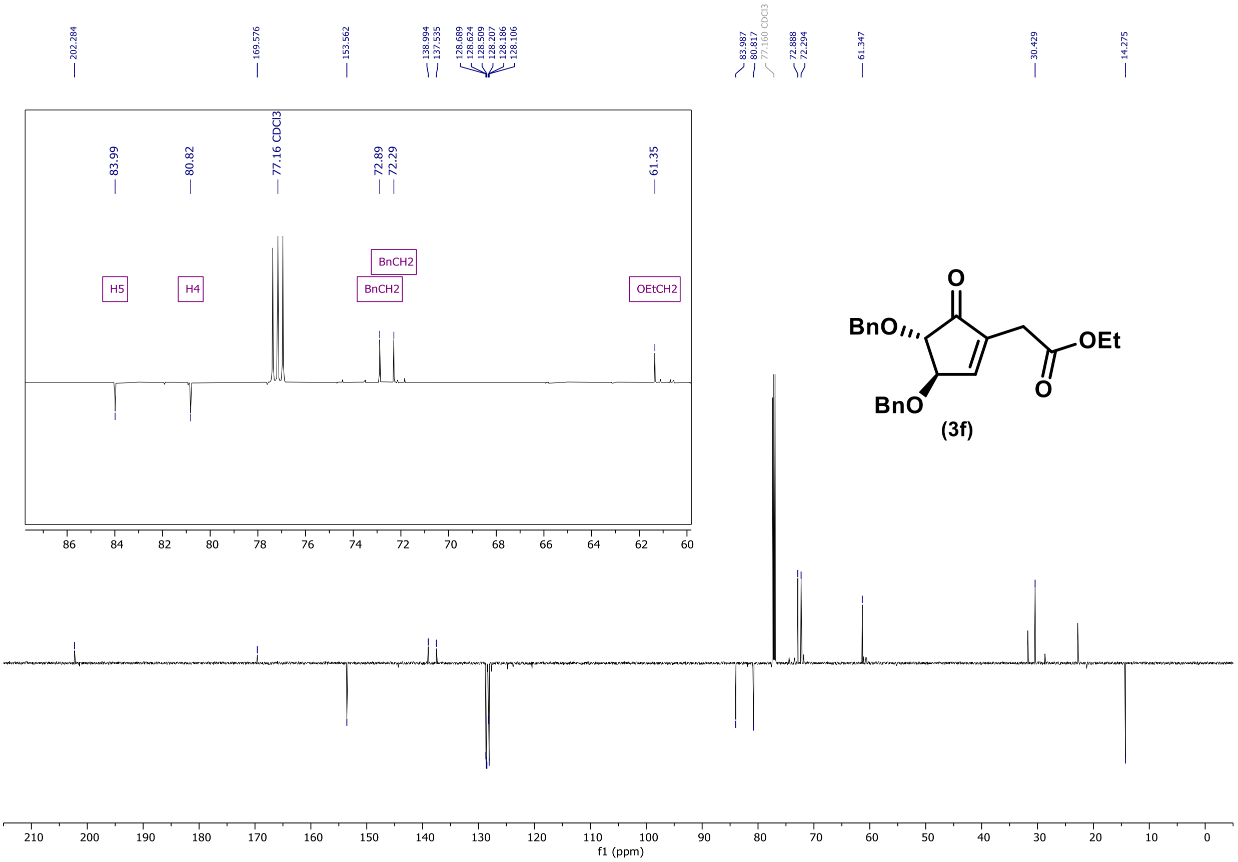

Figure S174. *151 MHz ^{13}C{^{1}H}-NMR of (3f)*

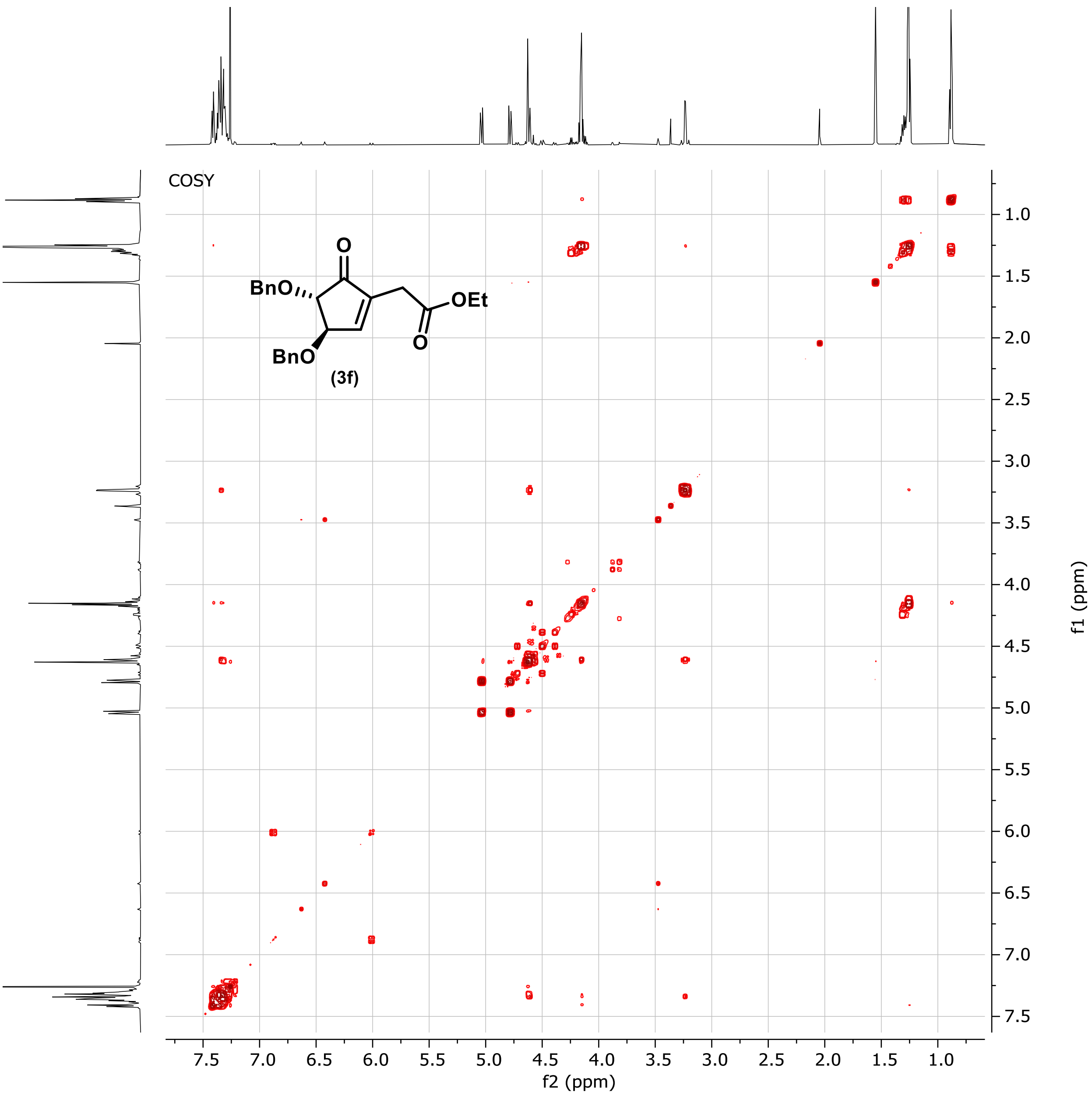

Figure S175. *COSY NMR of (3f)*

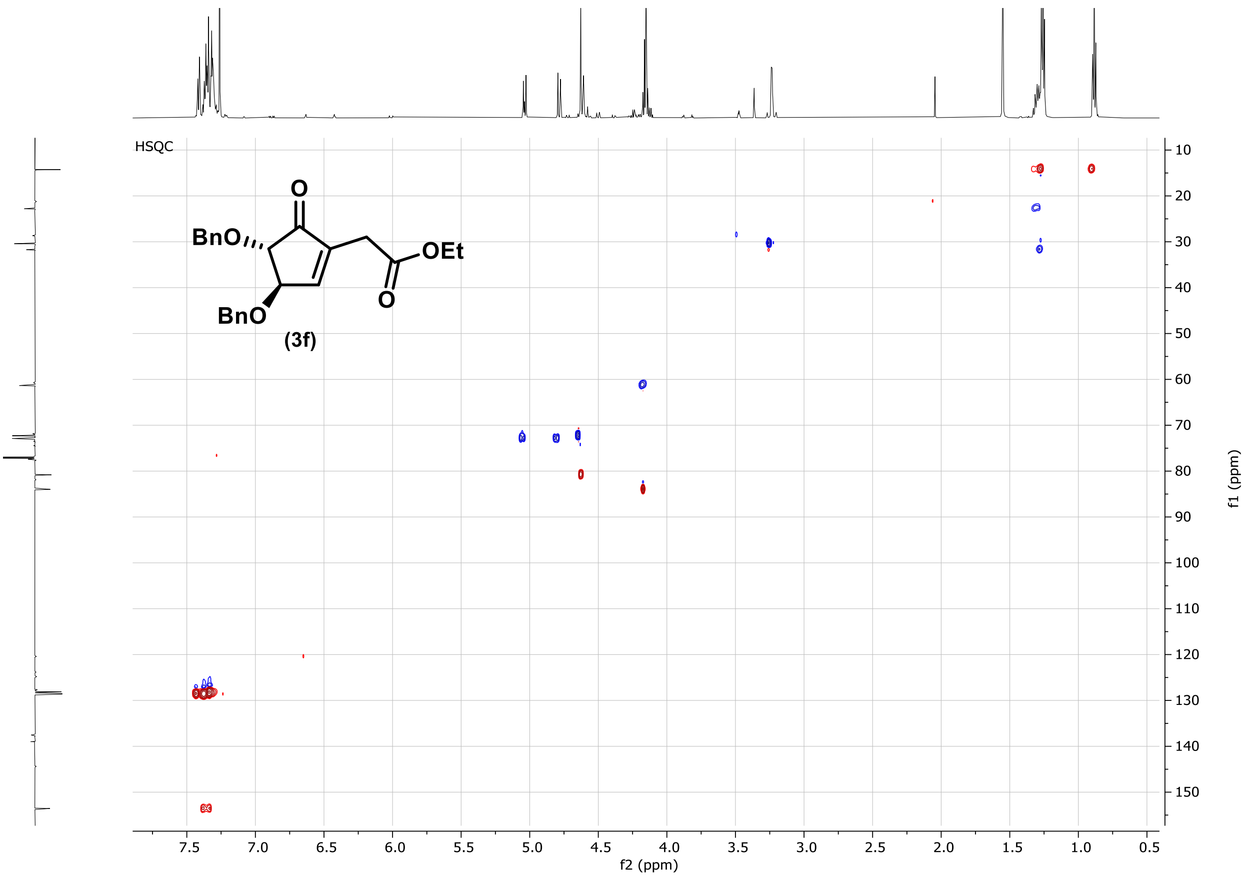

Figure S176. *HSQC NMR of (3f)*

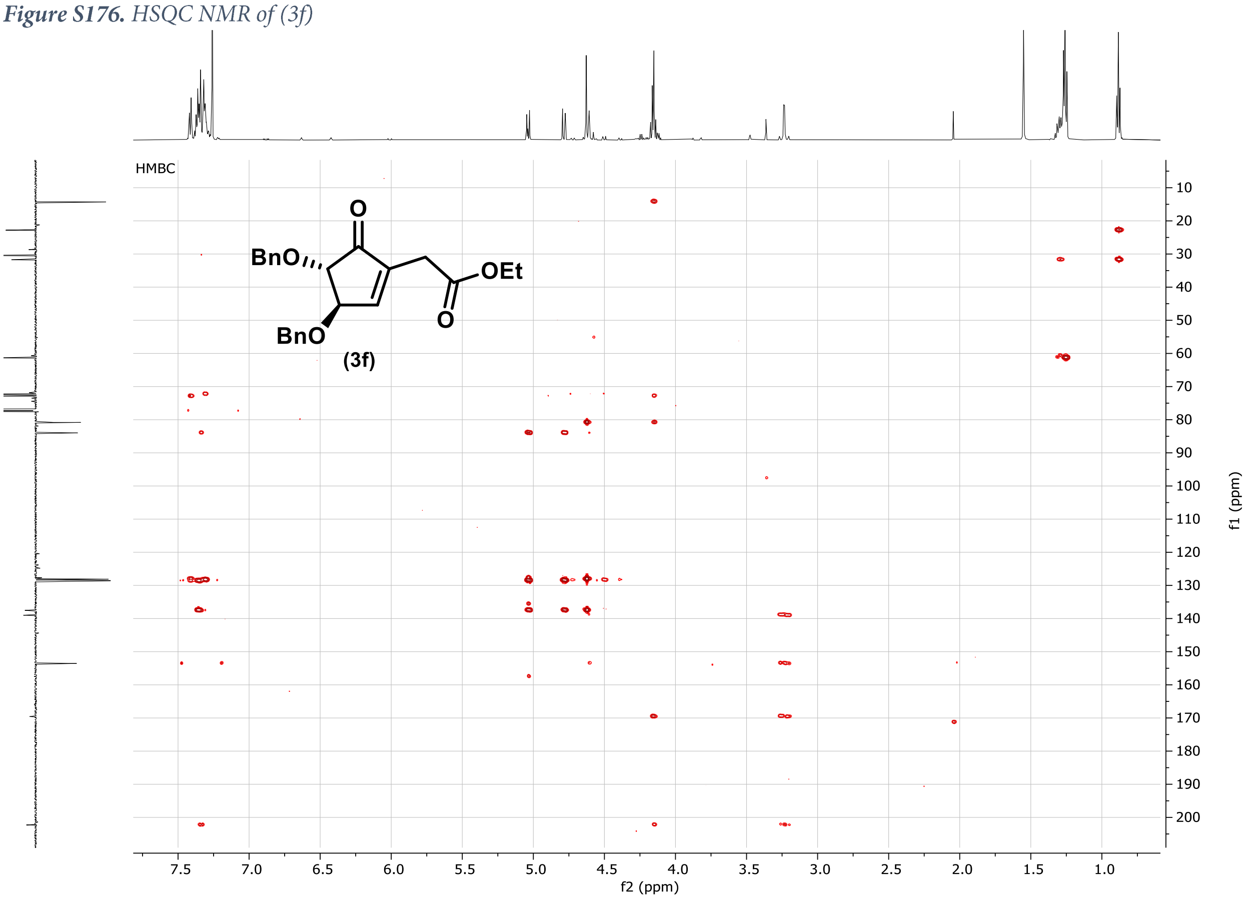

Figure S177. *HMBC NMR of (3f)*

D.5.1.5. Benzyl [(3S,4R,5S)-3,4,5-tris(benzyloxy)-2-oxocyclopentyl]acetate (2g)

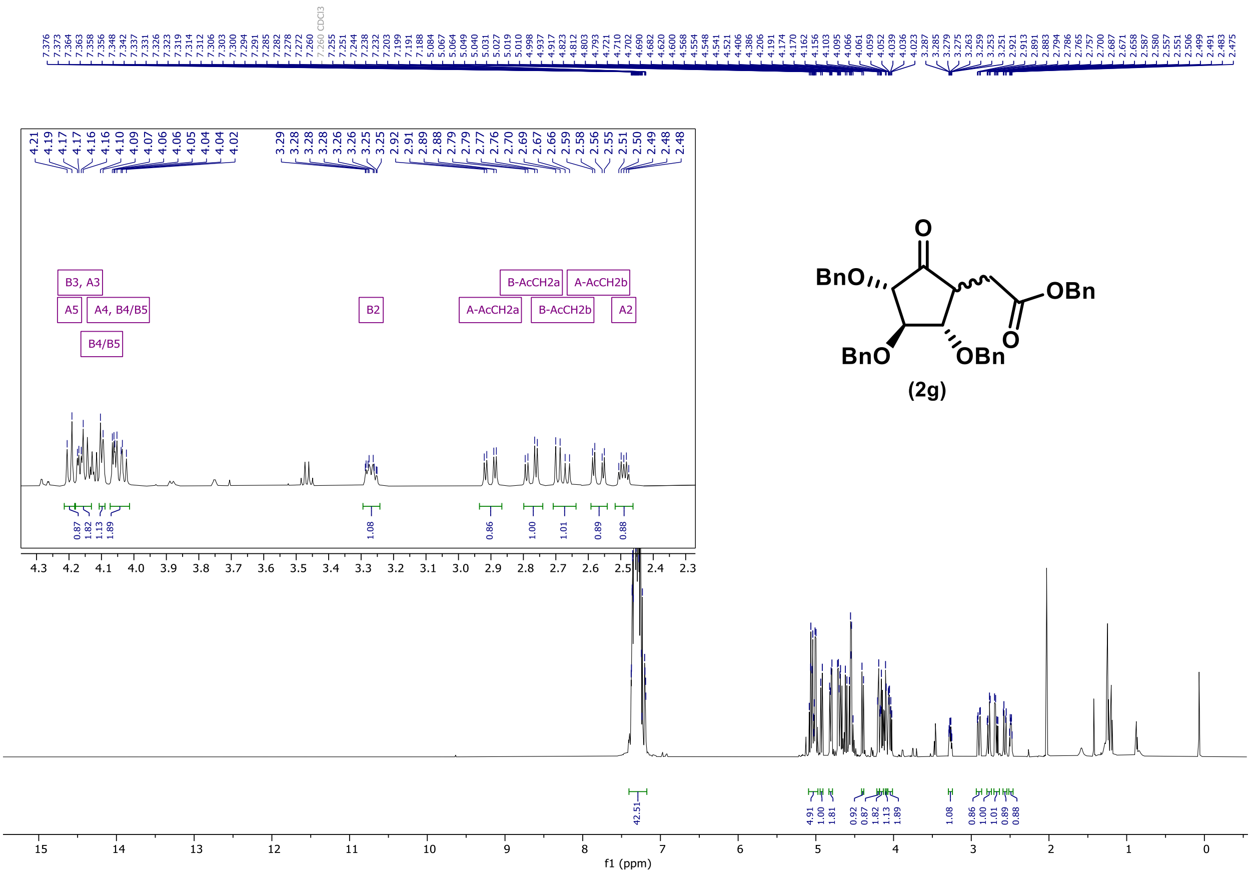

Figure S178. *600 MHz ^{1}H-NMR of (2g)*

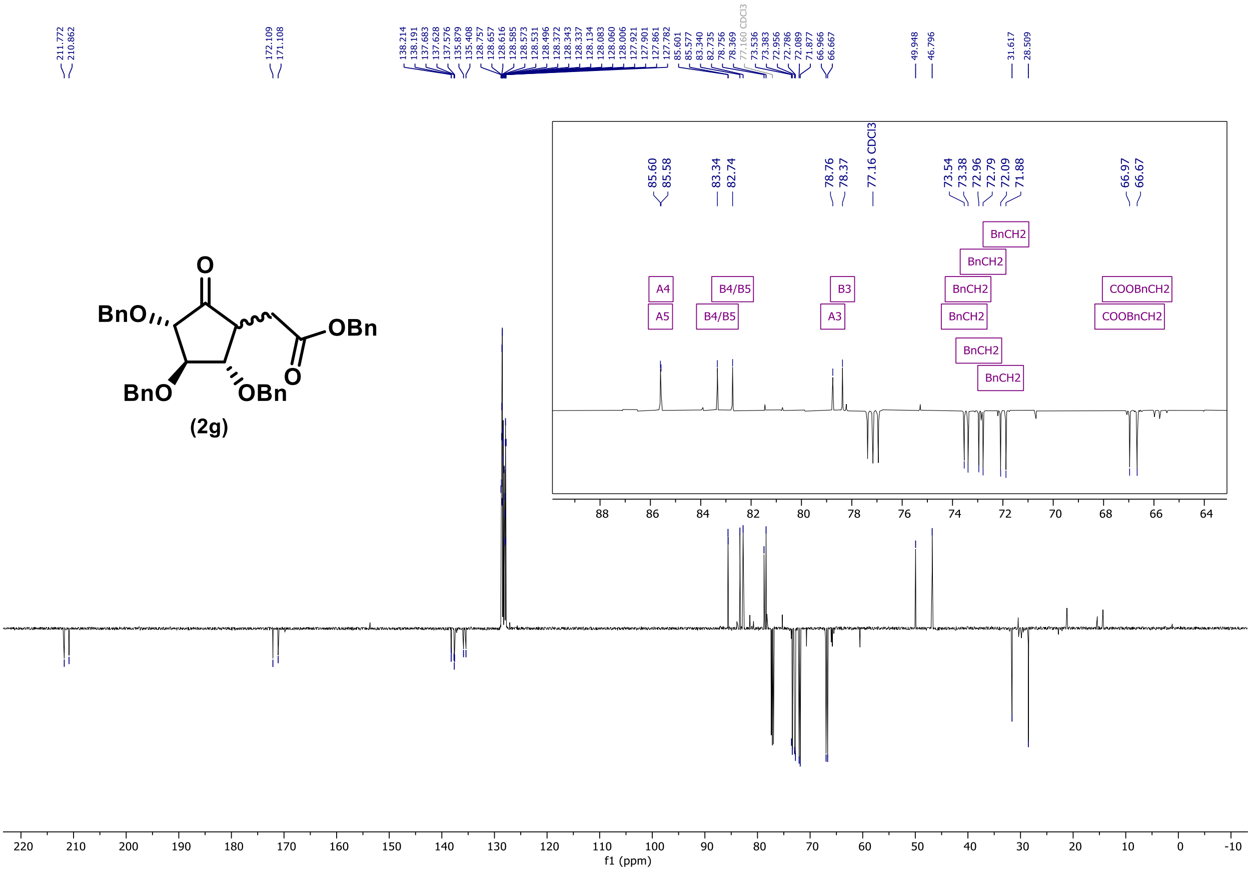

Figure S179. *151 MHz ^{13}C{^{1}H}-NMR of (2g)*

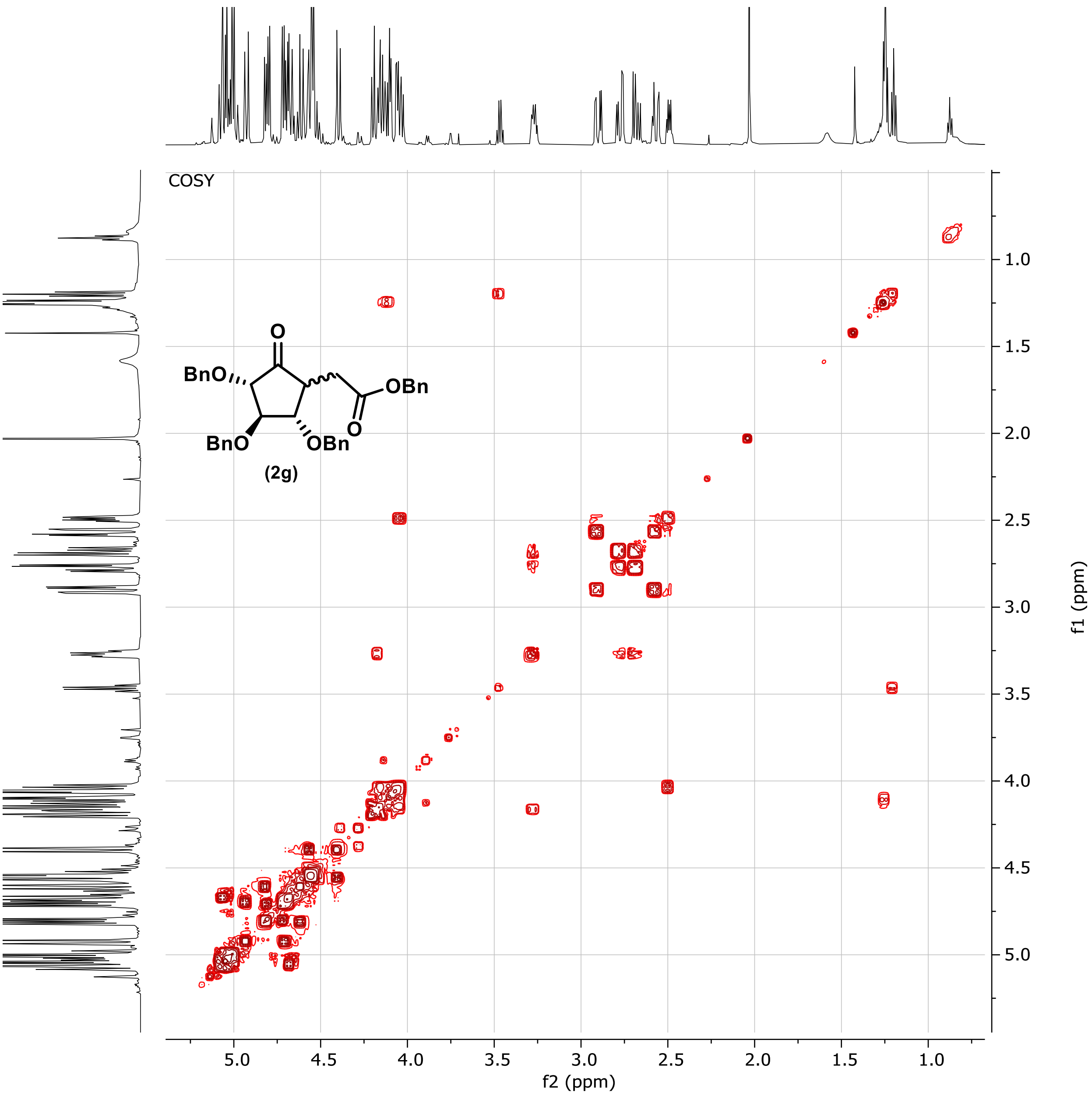

Figure S180. COSY NMR of (2g)

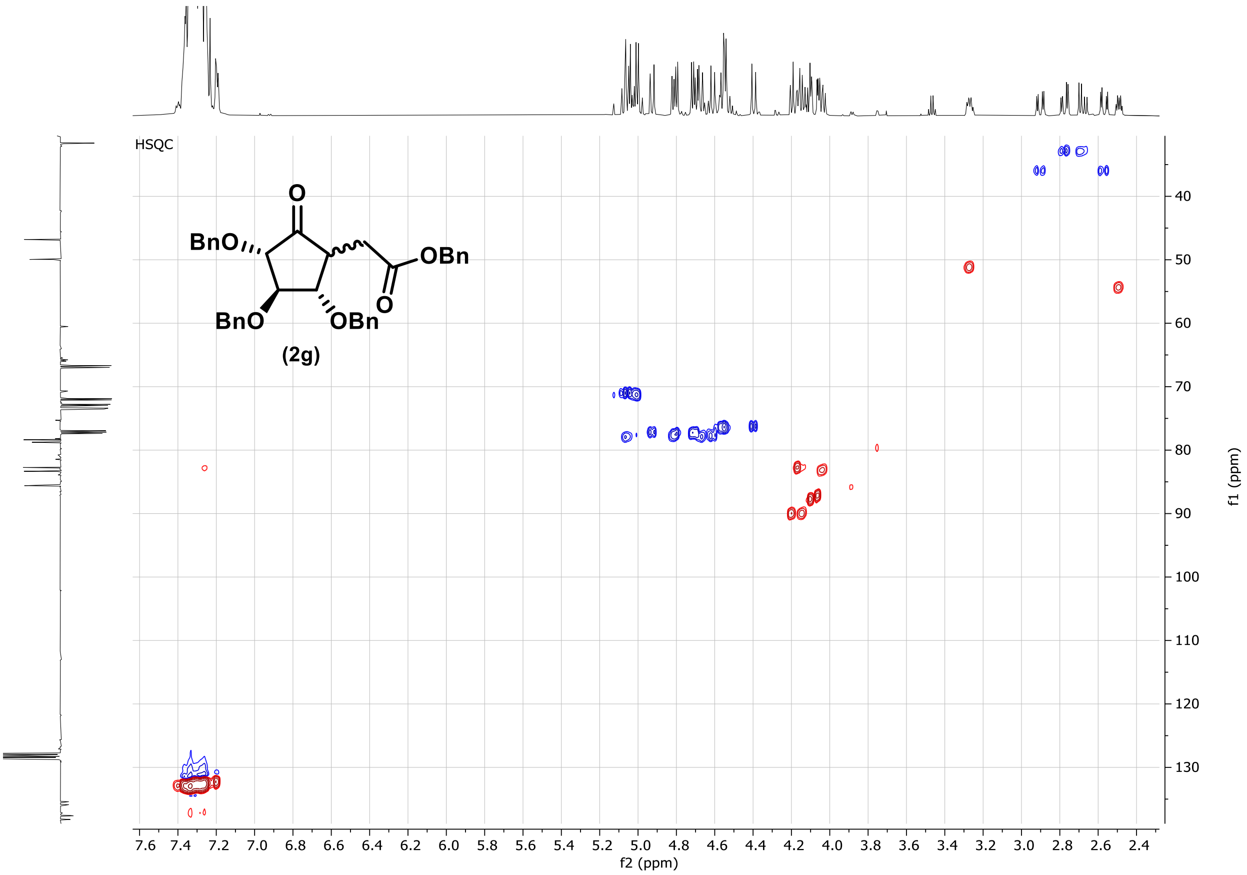

Figure S181. HSQC NMR of (2g)

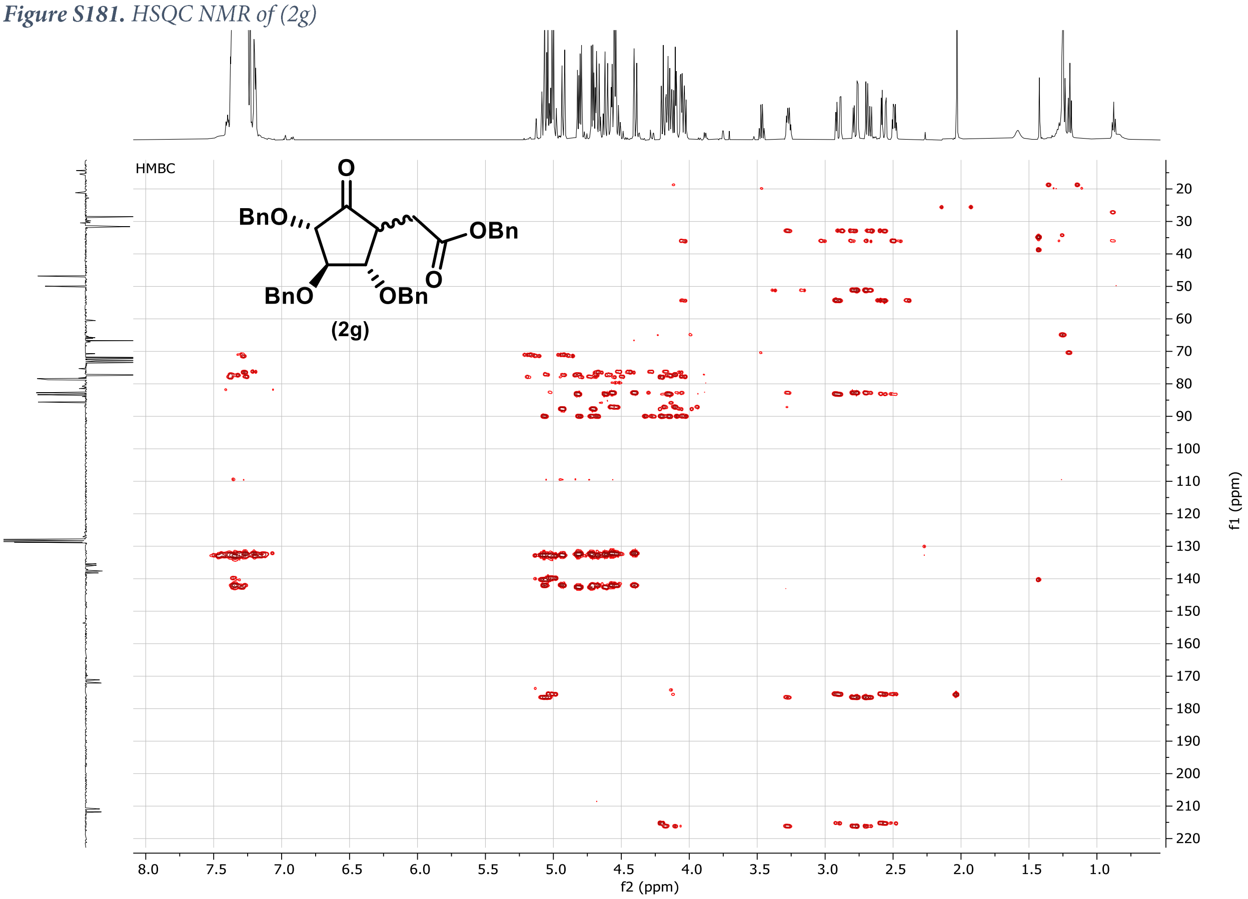

Figure S182. HMBC NMR of (2g)

D.5.1.6. Benzyl [(3R,4S)-3,4-bis(benzyloxy)-5-oxocyclopent-1-en-1-yl]acetate (3g)

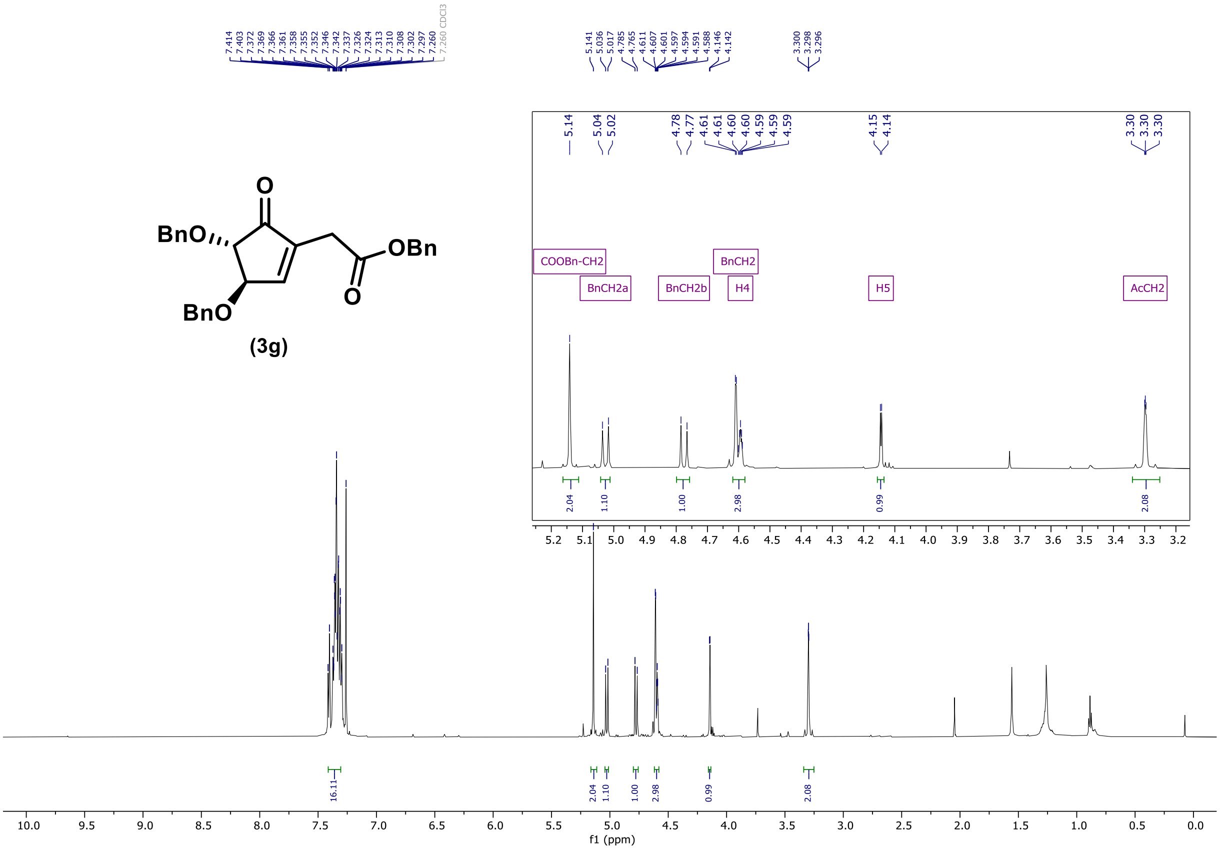

Figure S183. 600 MHz ^{1}H-NMR of (3g)

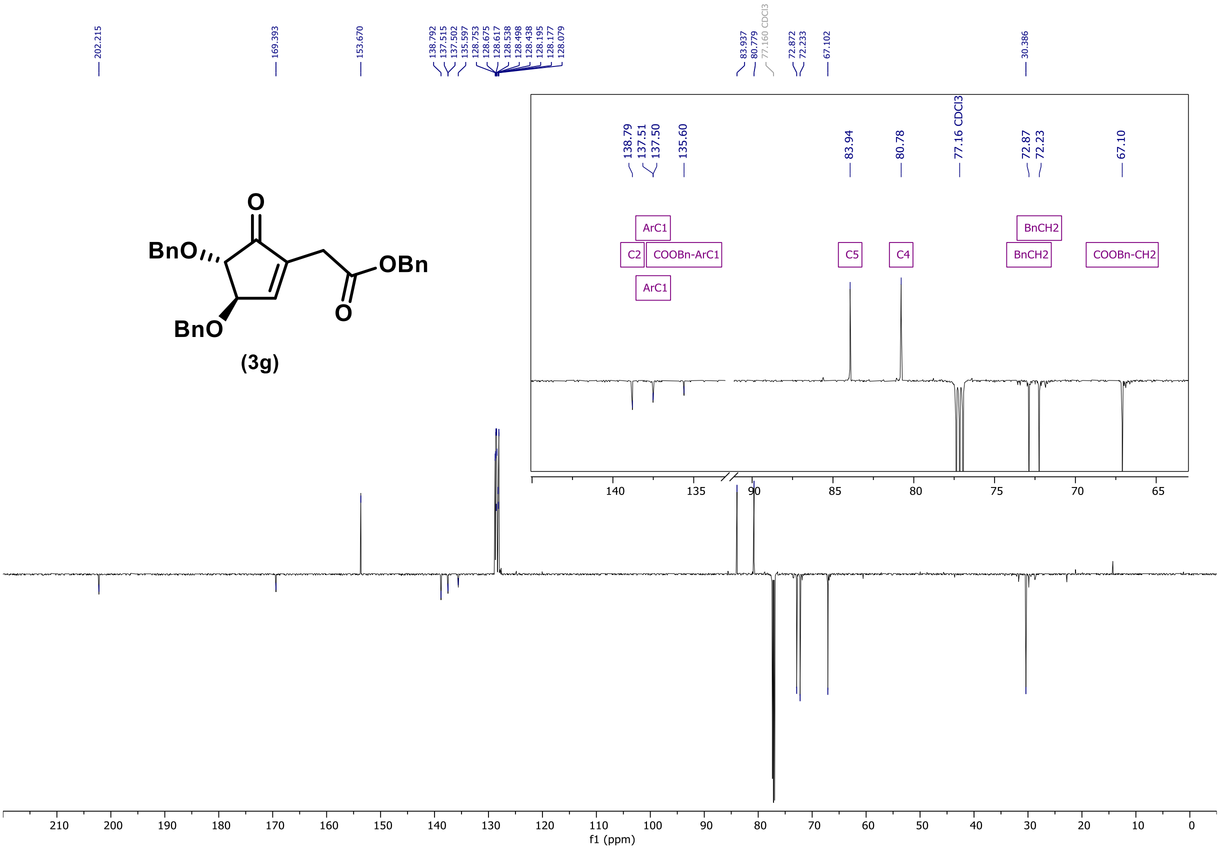

Figure S184. 151 MHz 13C{1H}-NMR of (3g)

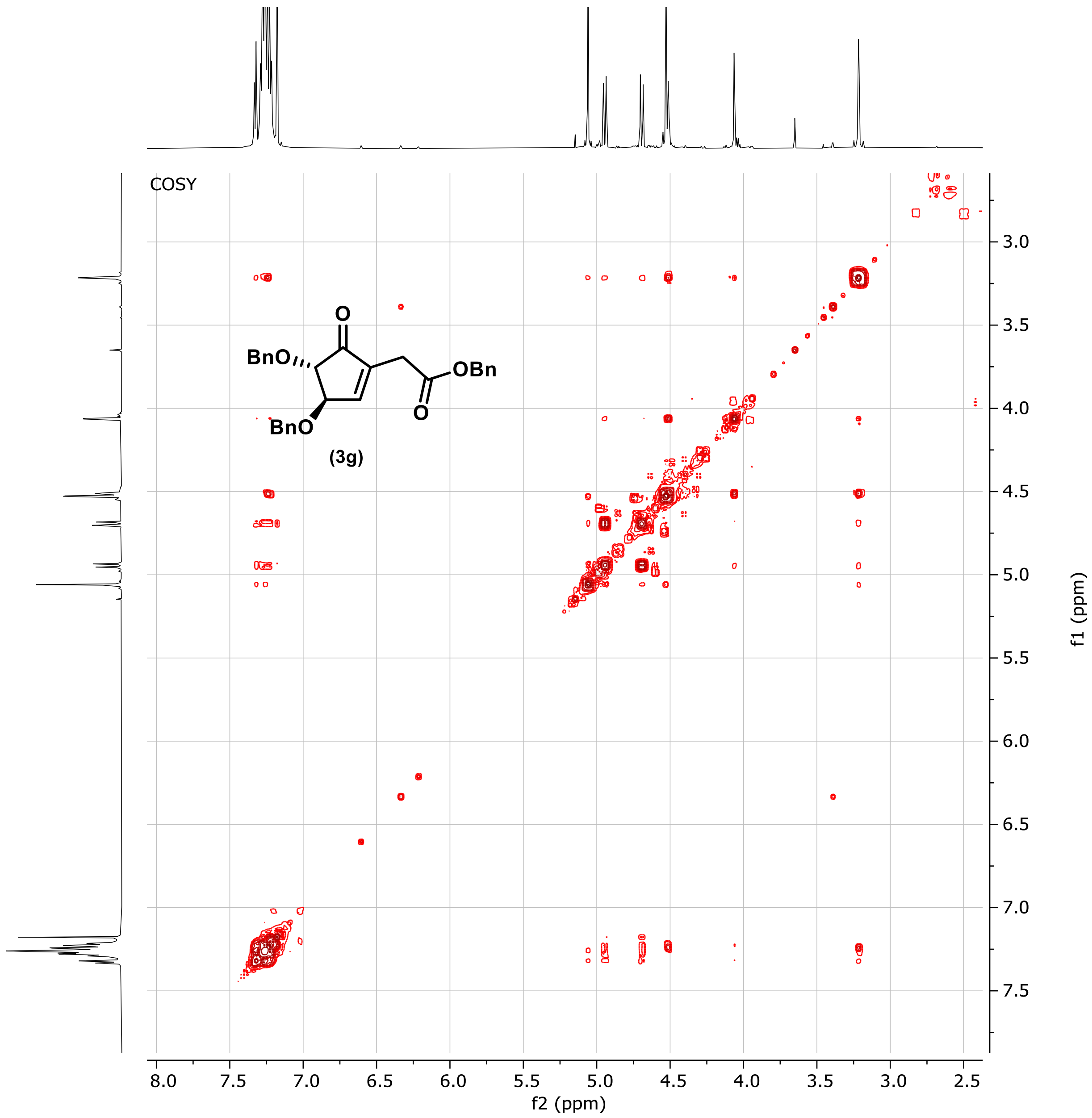

__Figure S185.__ COSY NMR of (3g)

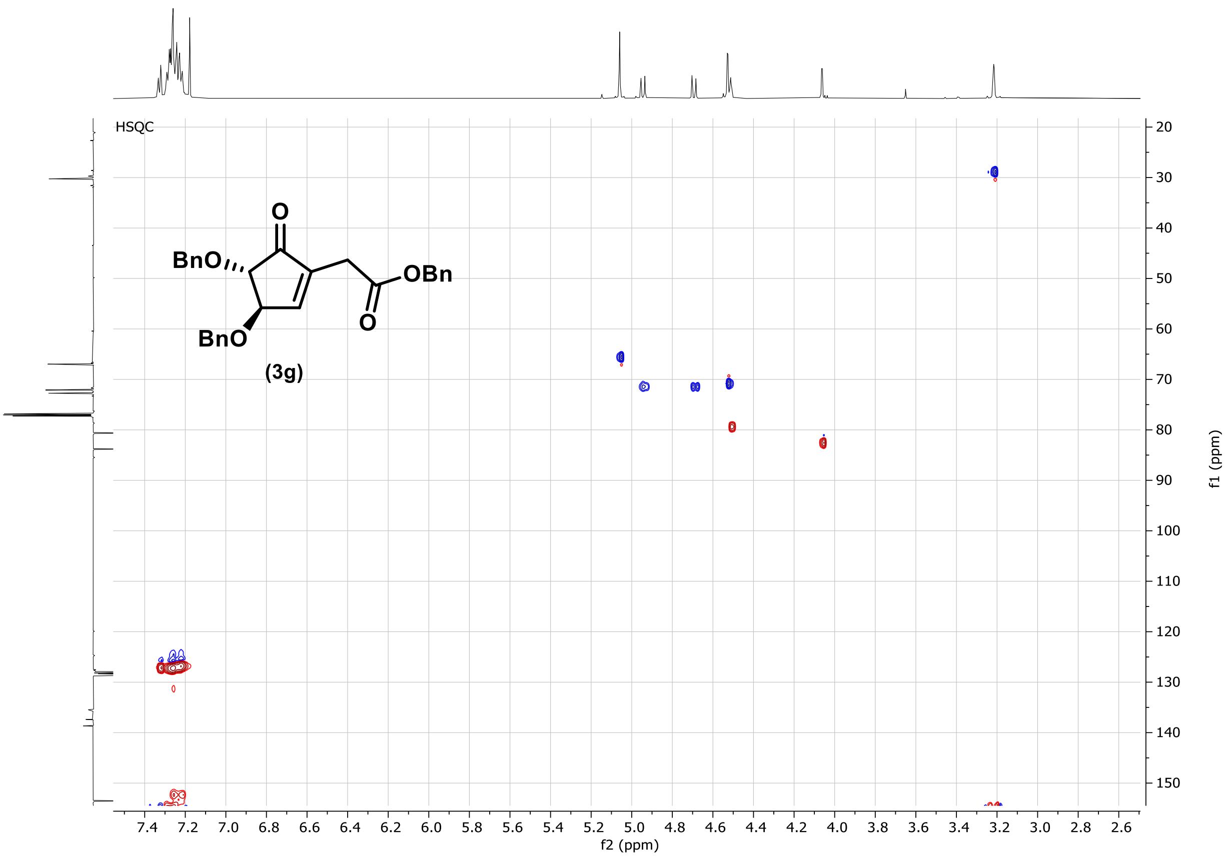

Figure S186. HSQC NMR of (3g)

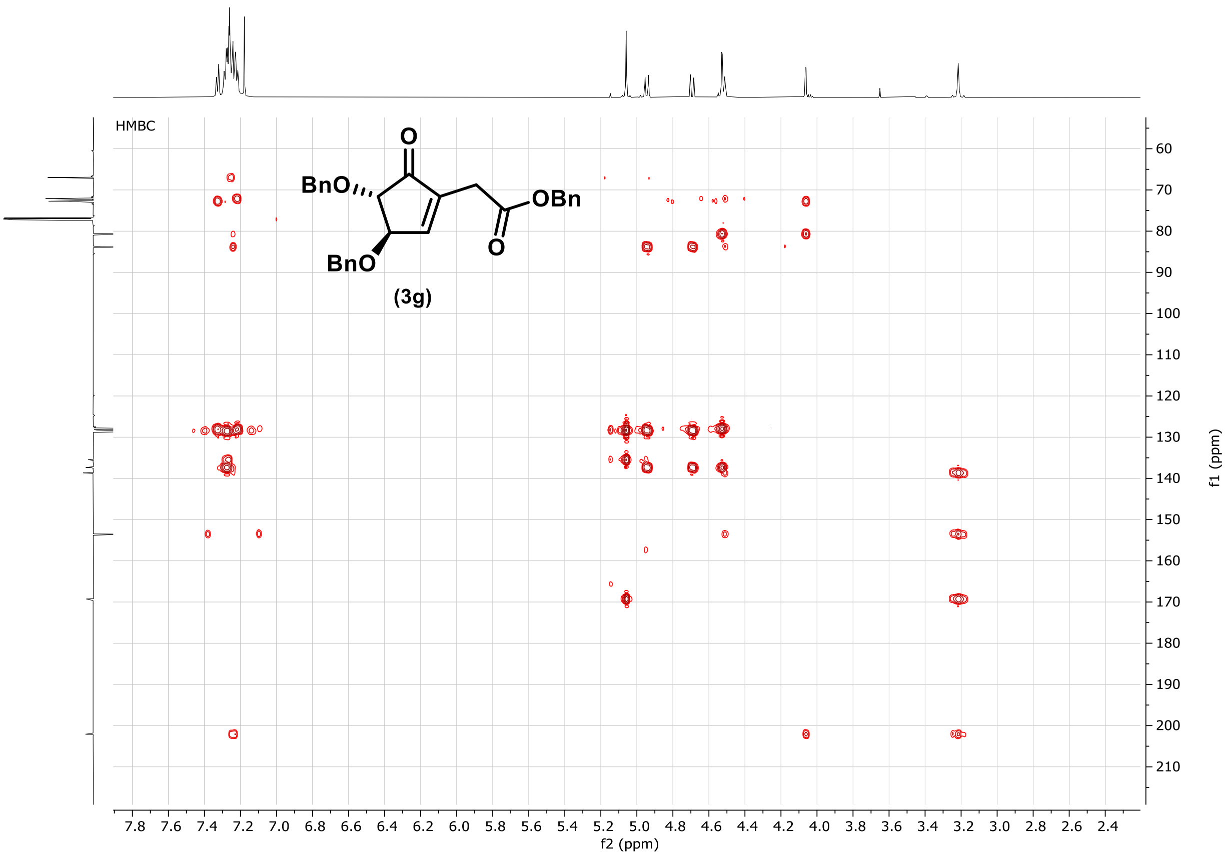

Figure S187. HMBC NMR of (3g)

D.5.1.7. Tert. Butyl [(3S,4R,5S)-3,4,5-tris(benzyloxy)-2-oxocyclopentyl]acetate (2h)

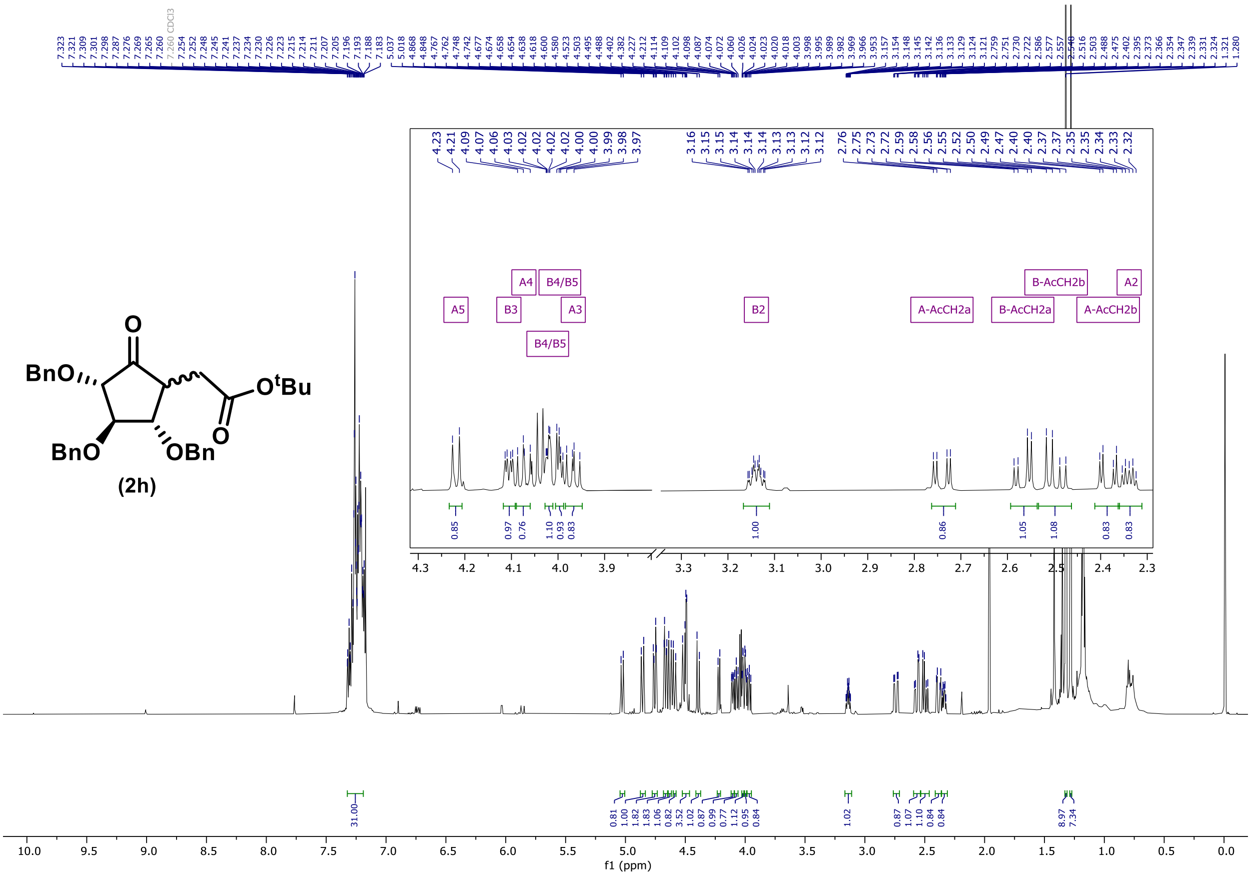

Figure S188. *600 MHz ^{1}H-NMR of (2h)*

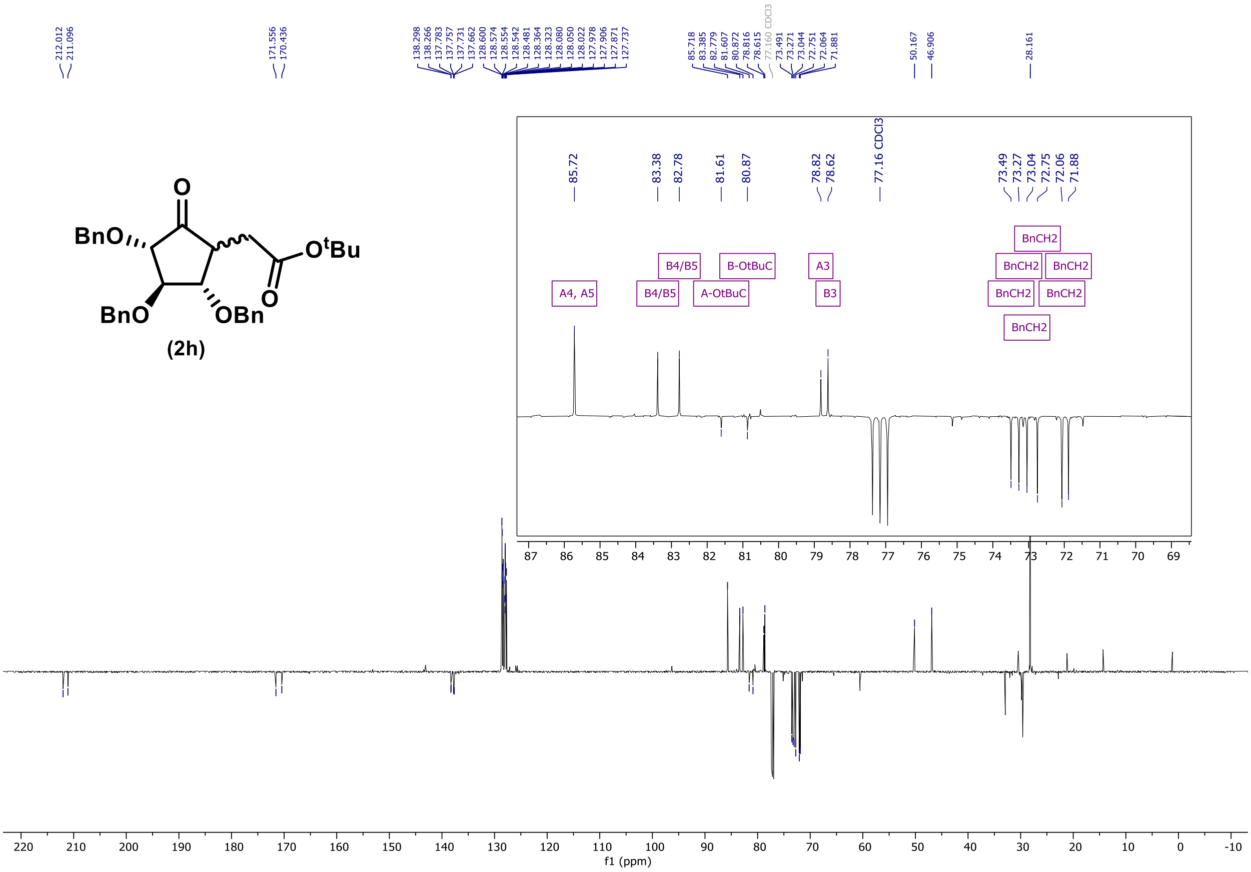

Figure S189. *151 MHz ^{13}C{^{1}H}-NMR of (2h)*

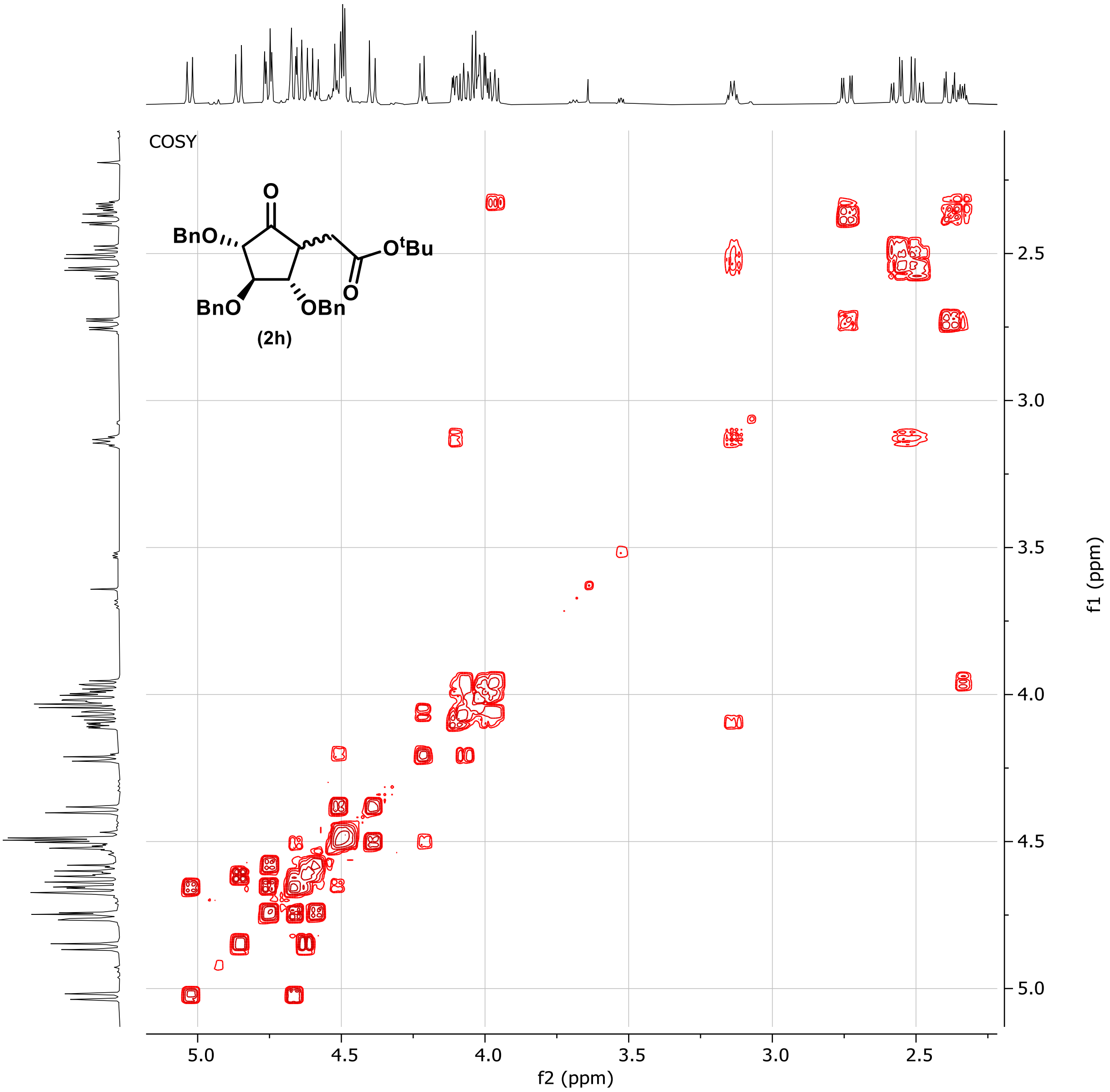

Figure S190. COSY NMR of (2h)

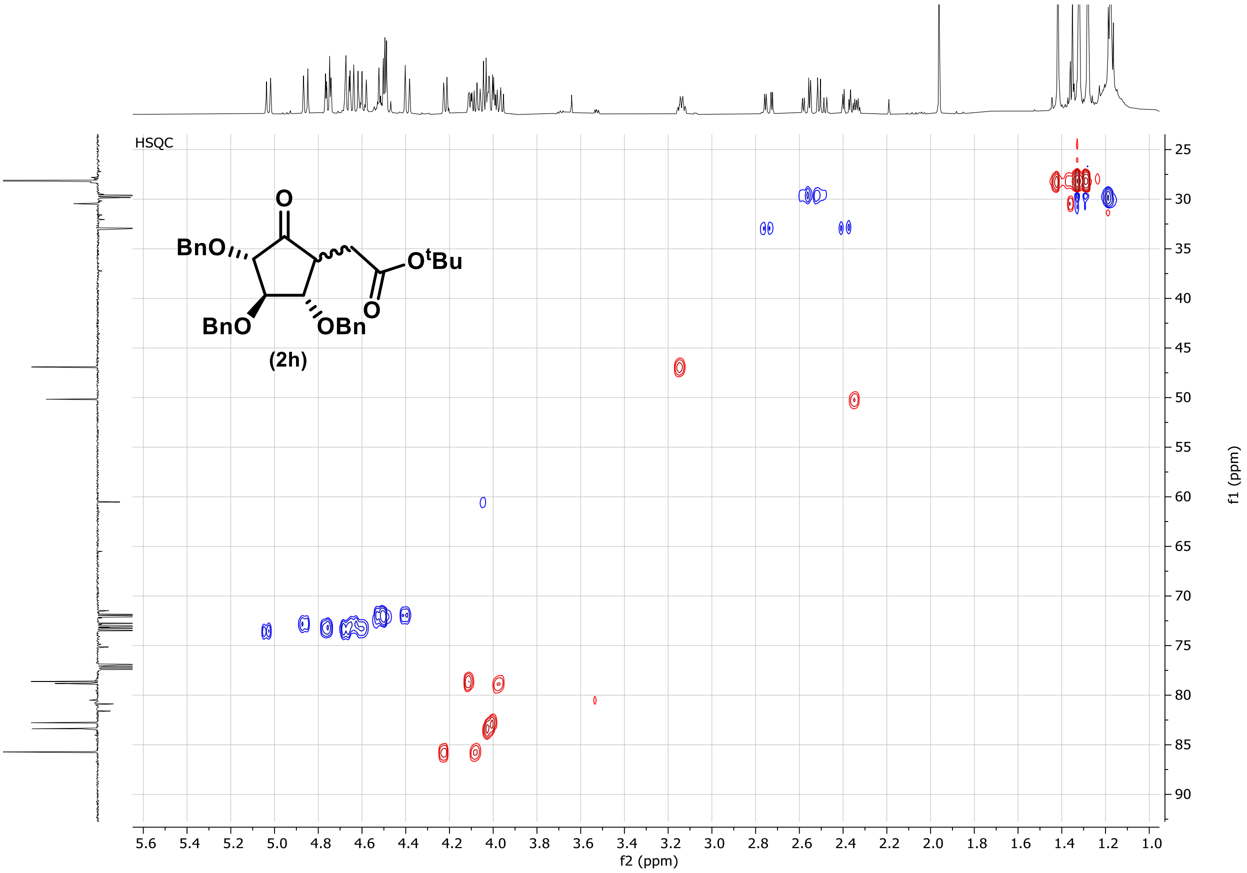

Figure S191. HSQC NMR of (2h)

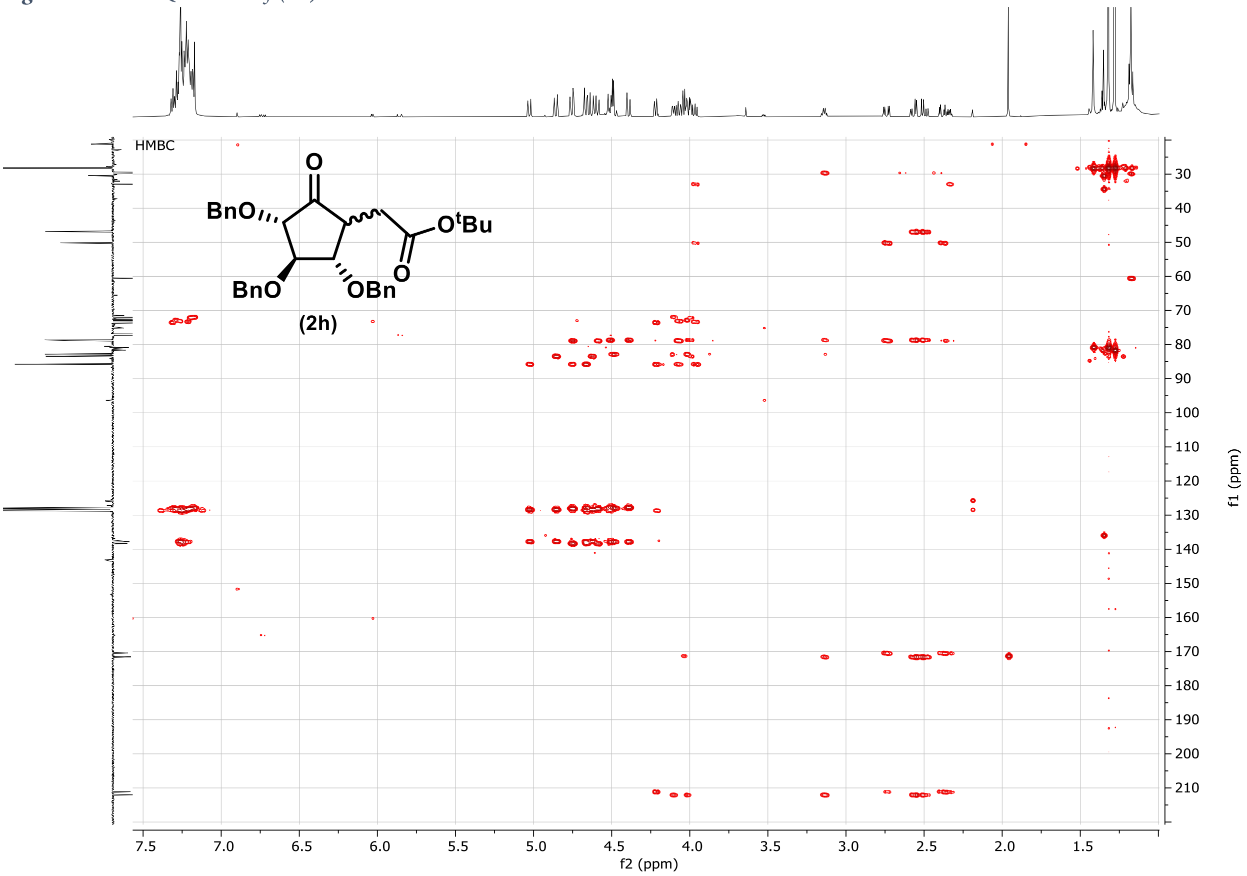

Figure S192. HMBC NMR of (2h)

D.5.1.8. [(3R,4S)-3,4-bis(benzyloxy)-5-oxocyclopent-1-en-1-yl]acetonitrile (3i)

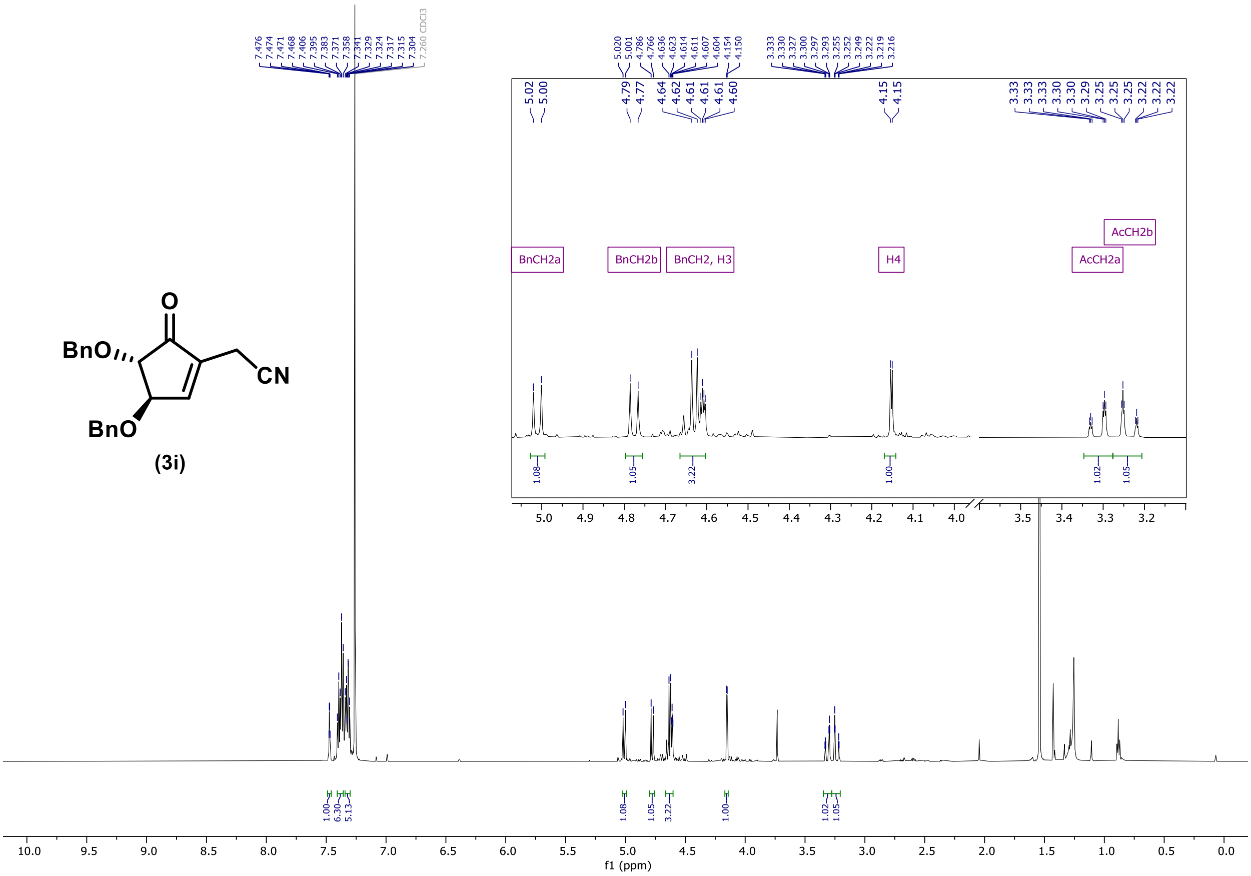

Figure S193. 600 MHz ^{1}H-NMR of (3i)

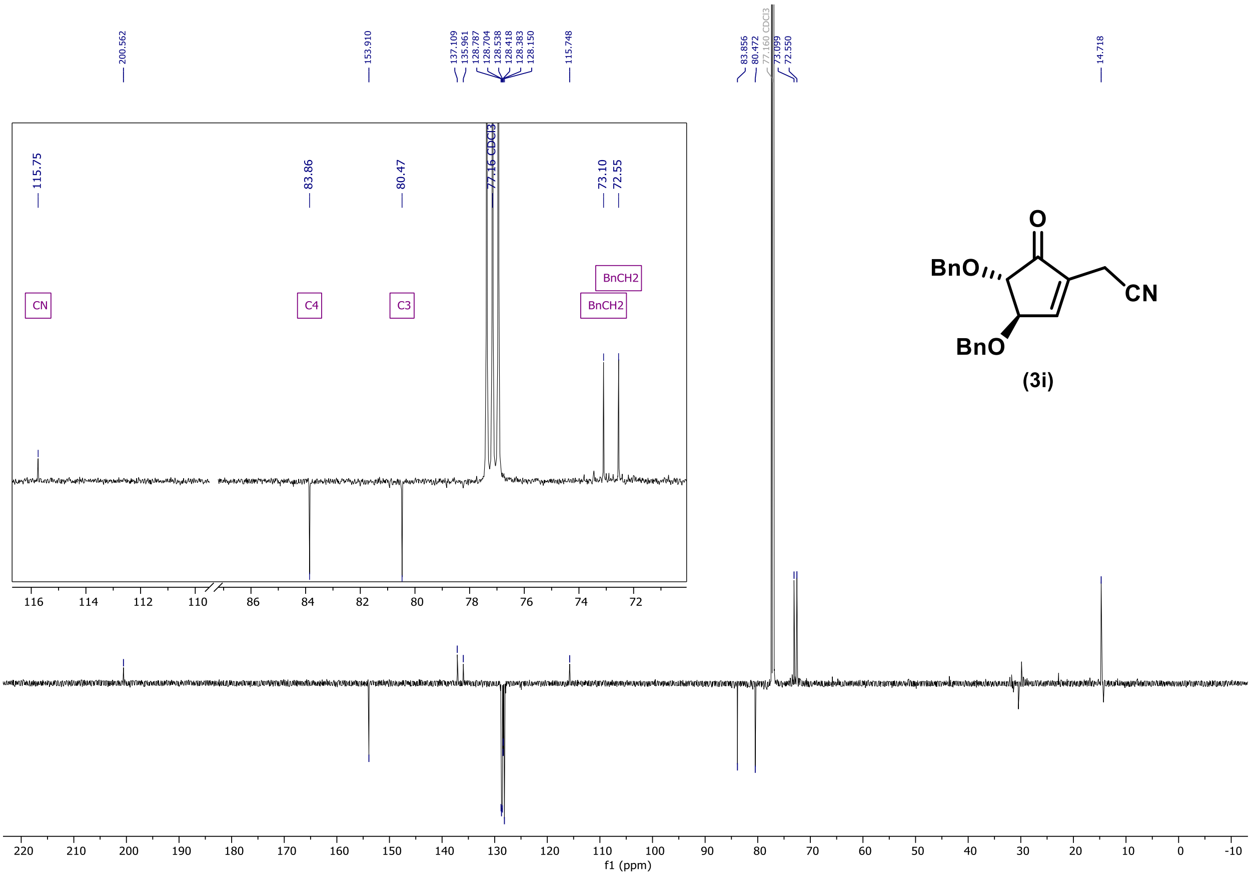

Figure S194. 151 MHz $^{13}C\{^{1}H\}$-NMR of (3i)

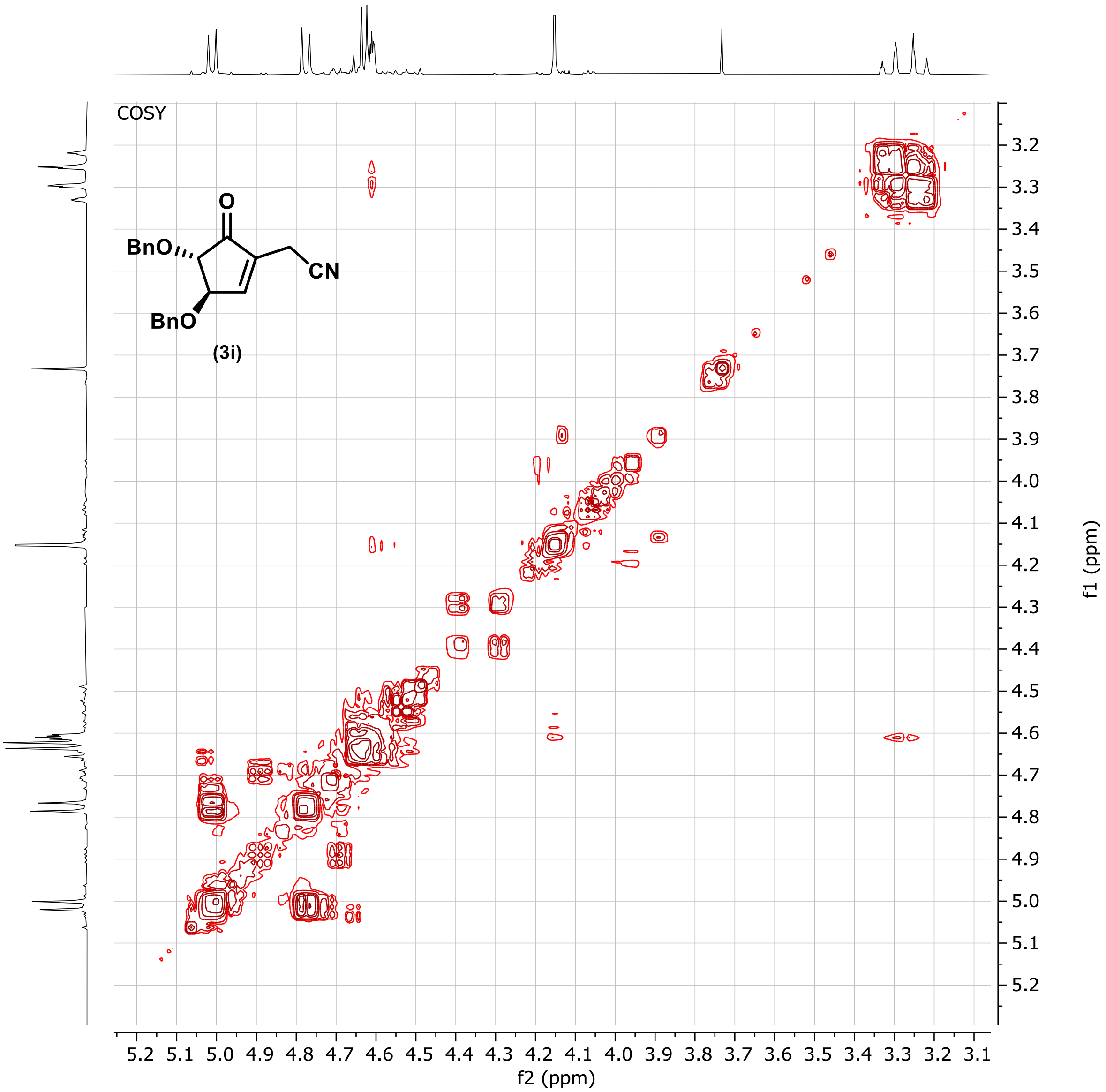

Figure S195. *COSY NMR of (3i)*

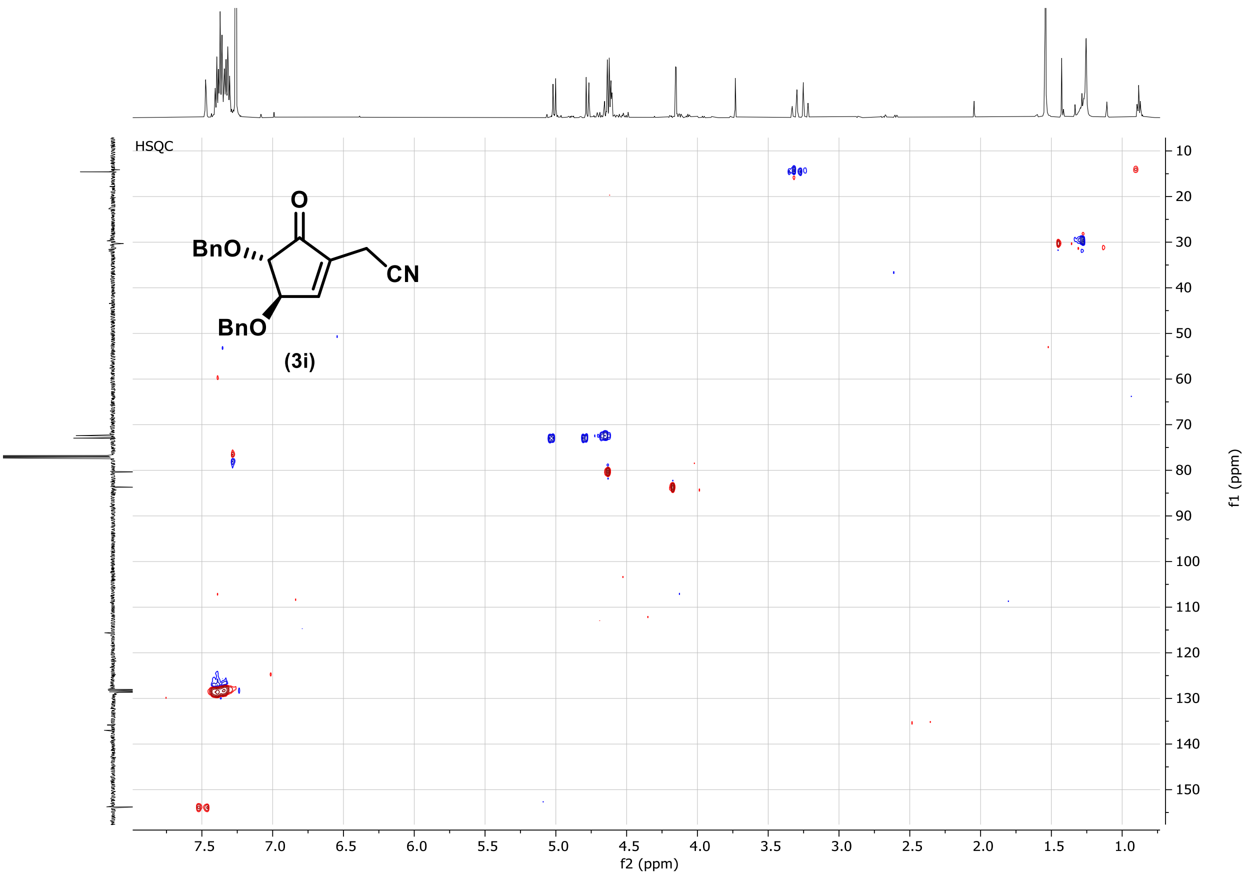

Figure S196. *HSQC NMR of (3i)*

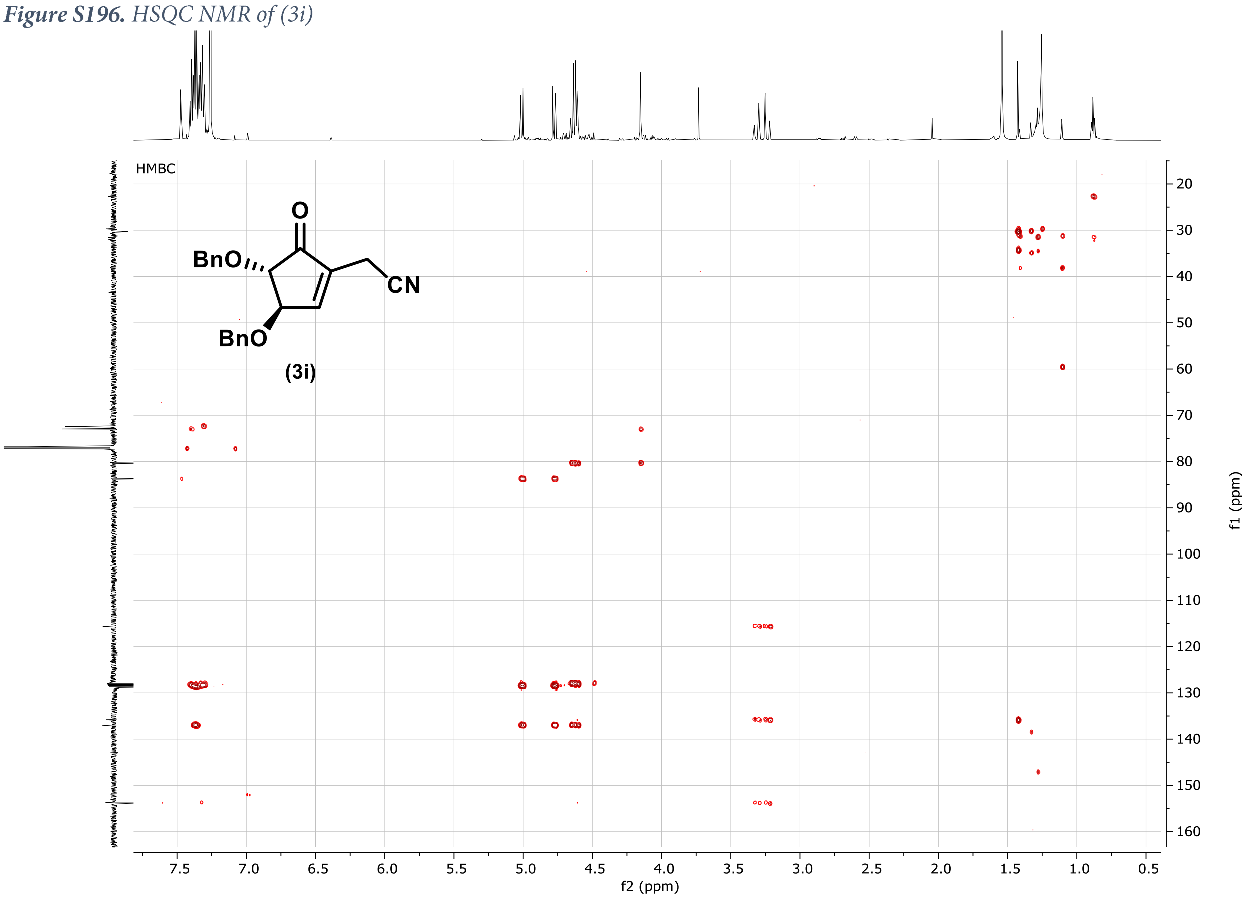

Figure S197. *HMBC NMR of (3i)*

D.5.1.9. Methyl [(1S,3S,4R,5S)-2-oxo-3,4,5-tris(trimethylsiloxy)cyclopentyl]acetate (2e)

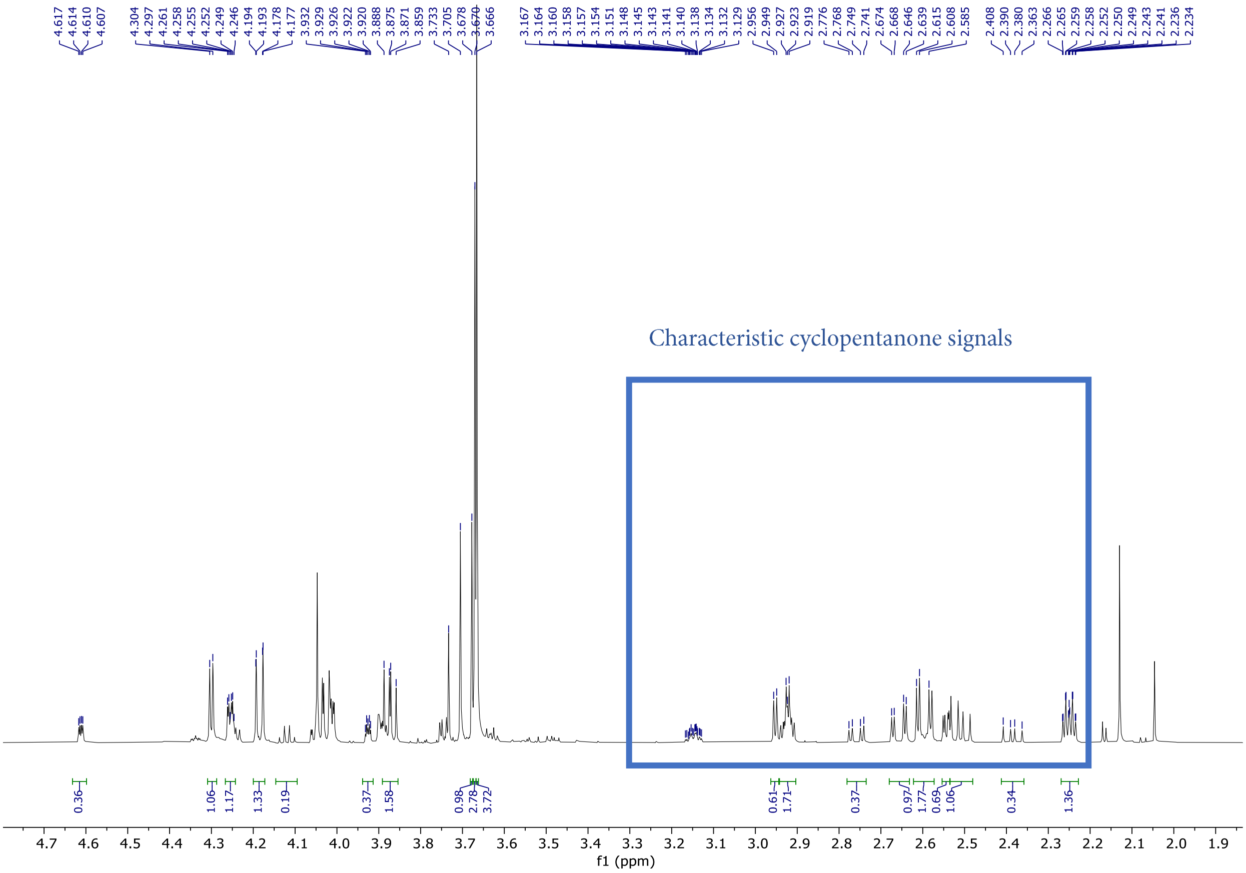

Figure S198. *151 MHz 1H-NMR of (2e)*

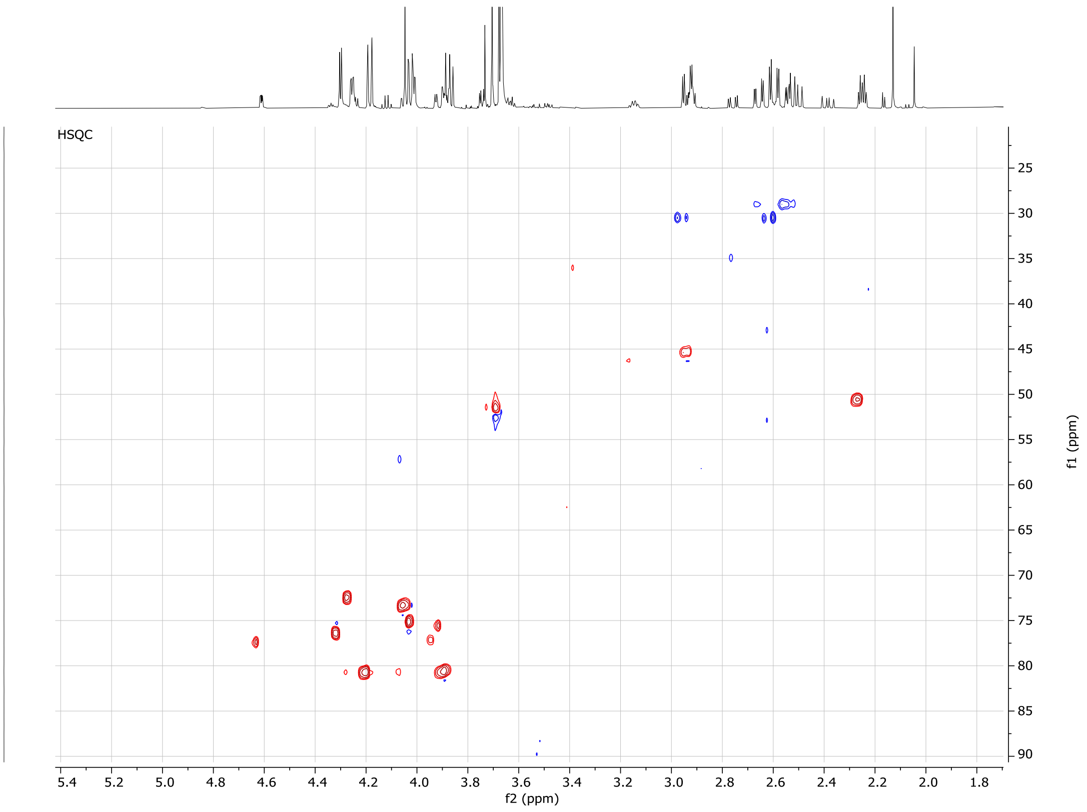

Figure S199. *HSQC of (2e)*

D.5.2. Other Stereoconfigurations

D.5.2.1. Methyl [(3S,4R,5R)-3,4,5-tris(benzyloxy)-2-oxocyclopentyl]acetate (Lyxo) *(2b)*

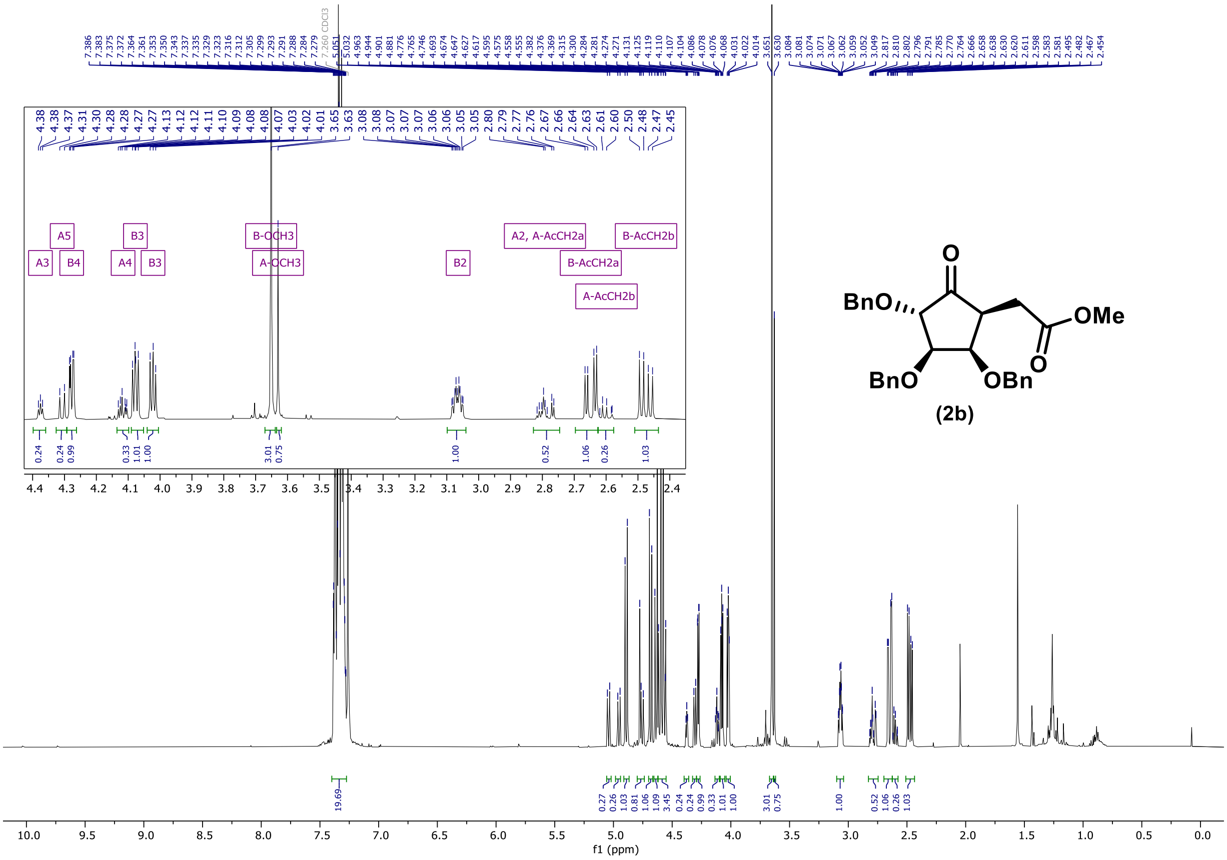

Figure S200. *600 MHz ^{1}H-NMR of (2l)*

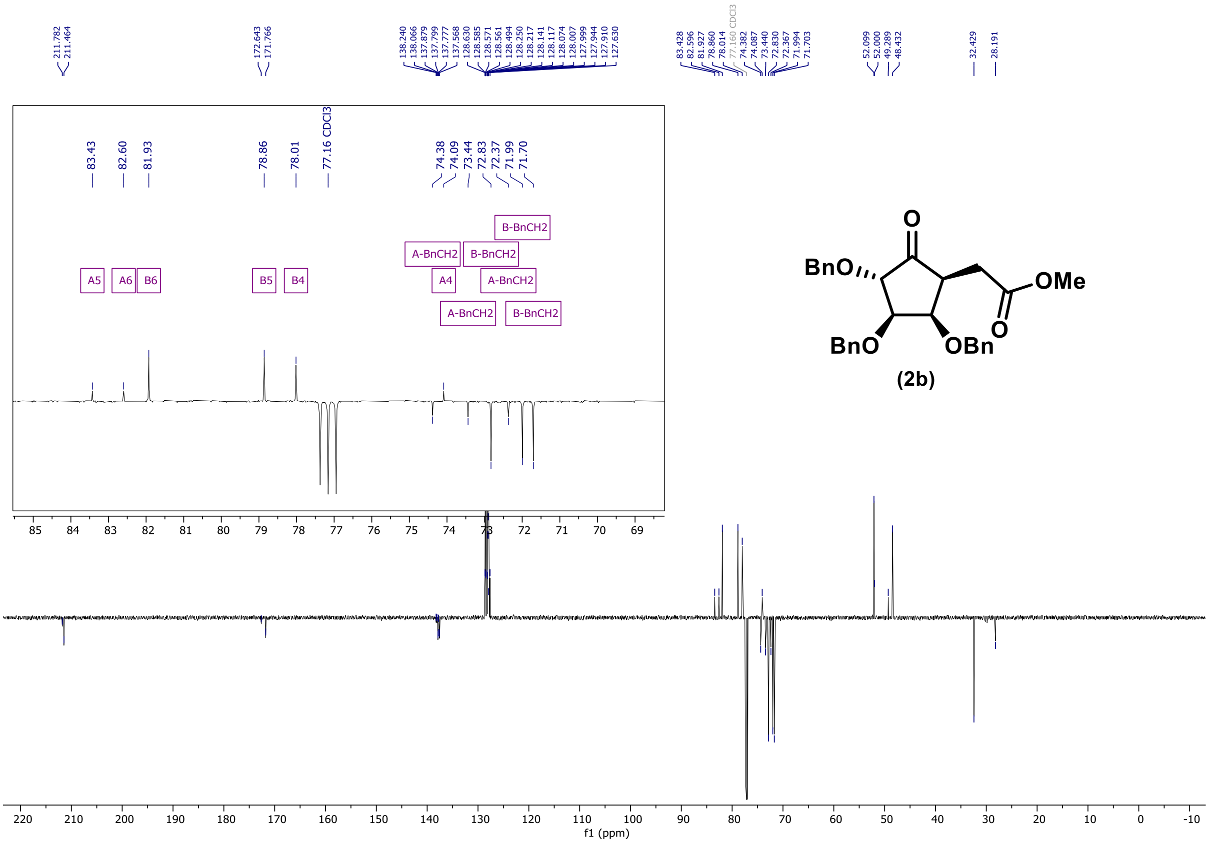

Figure S201. *151 MHz ^{13}C{^{1}H}-NMR of (2b)*

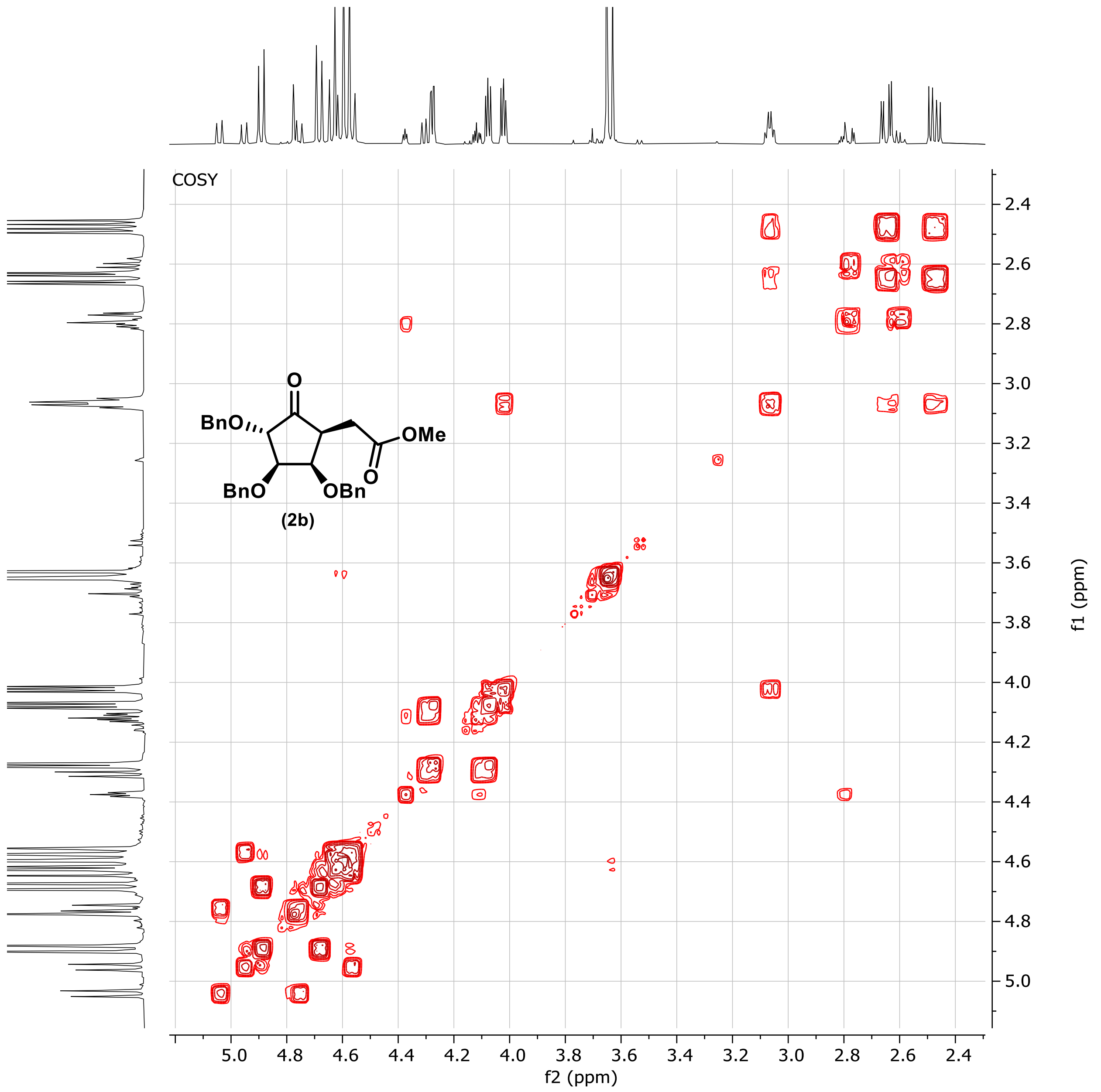

Figure S202. *COSY NMR of (2b)*

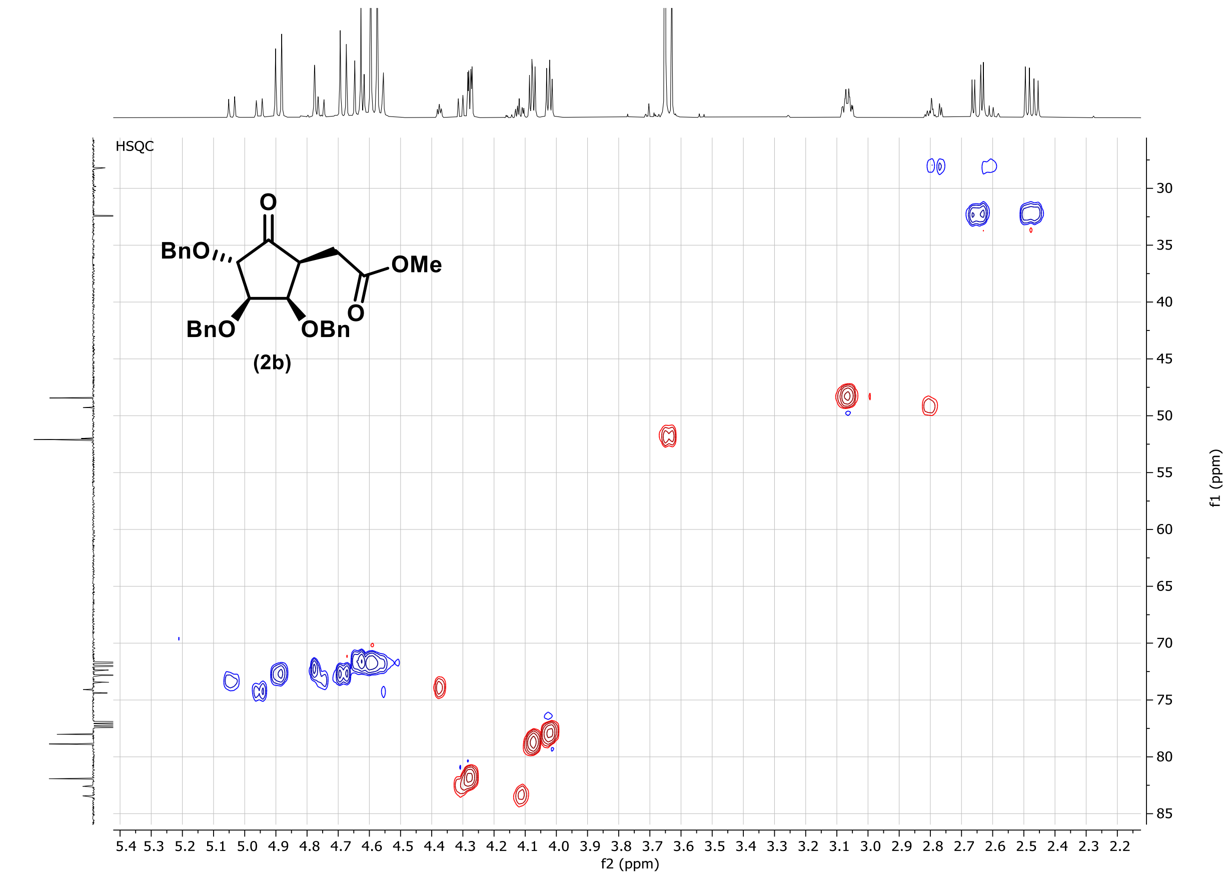

Figure S203. HSQC NMR of (2b)

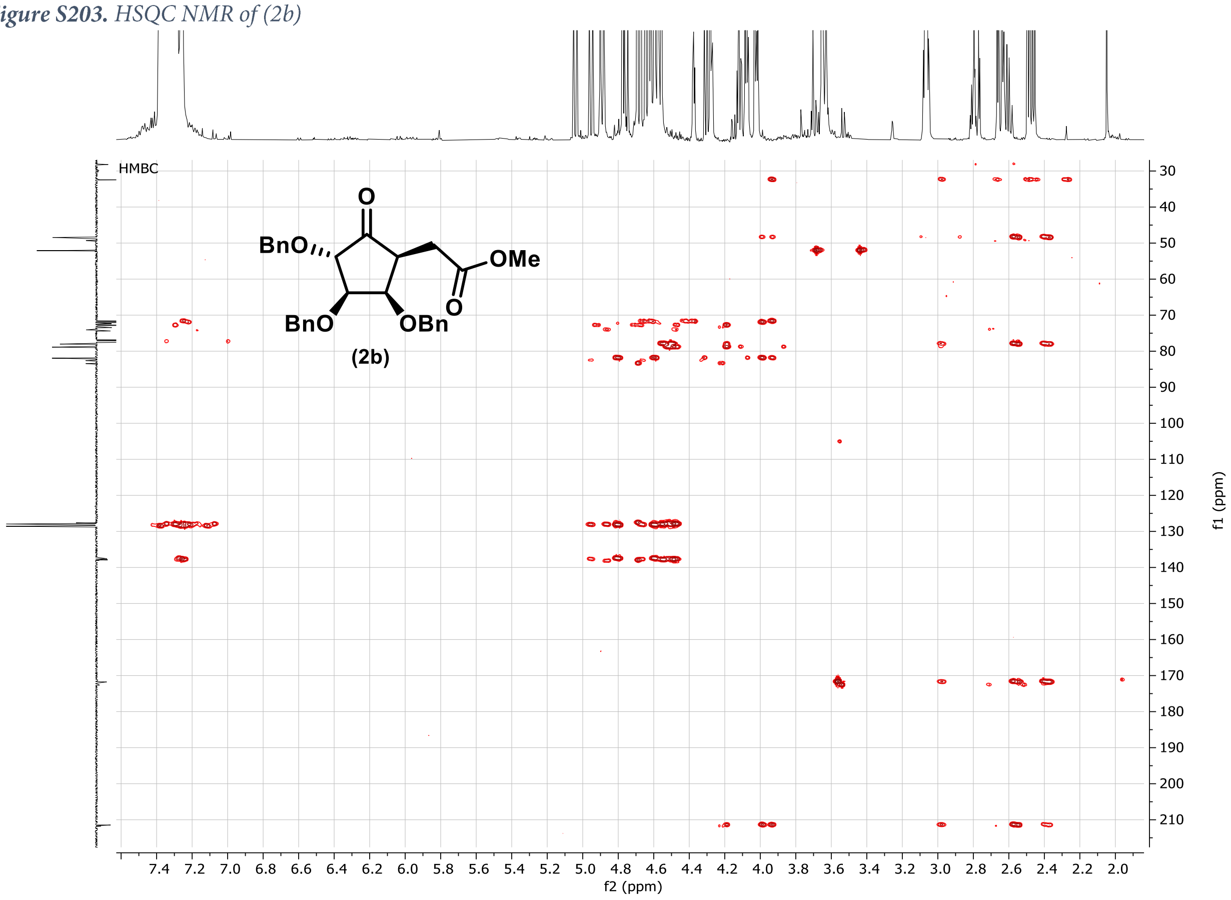

Figure S204. HMBC NMR of (2b)

D.5.2.2. Methyl [(1S,3S,4S,5S)-3,4,5-tris(benzyloxy)-2-oxocyclopentyl]acetate (ribo) *(2c)*

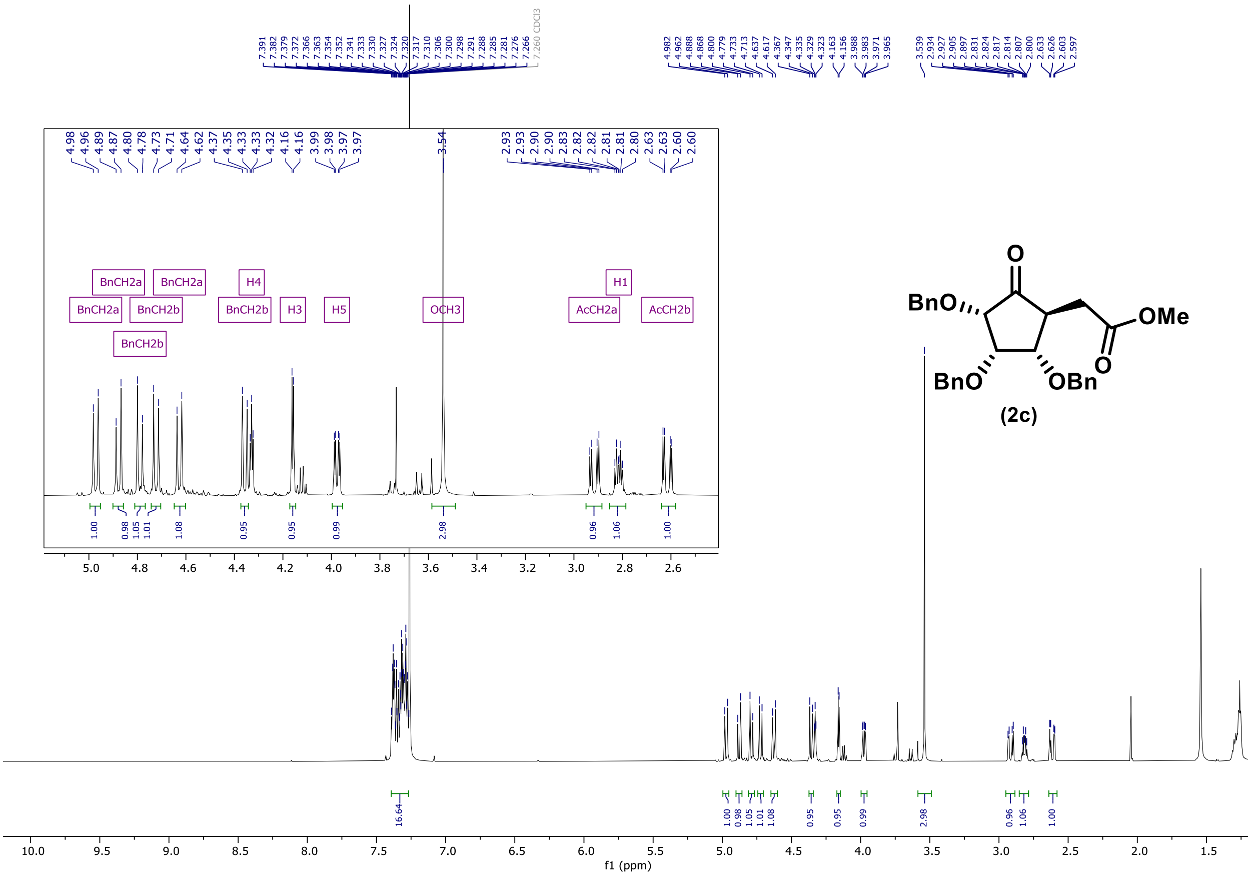

Figure S205. *600 MHz ¹H-NMR of (2c)*

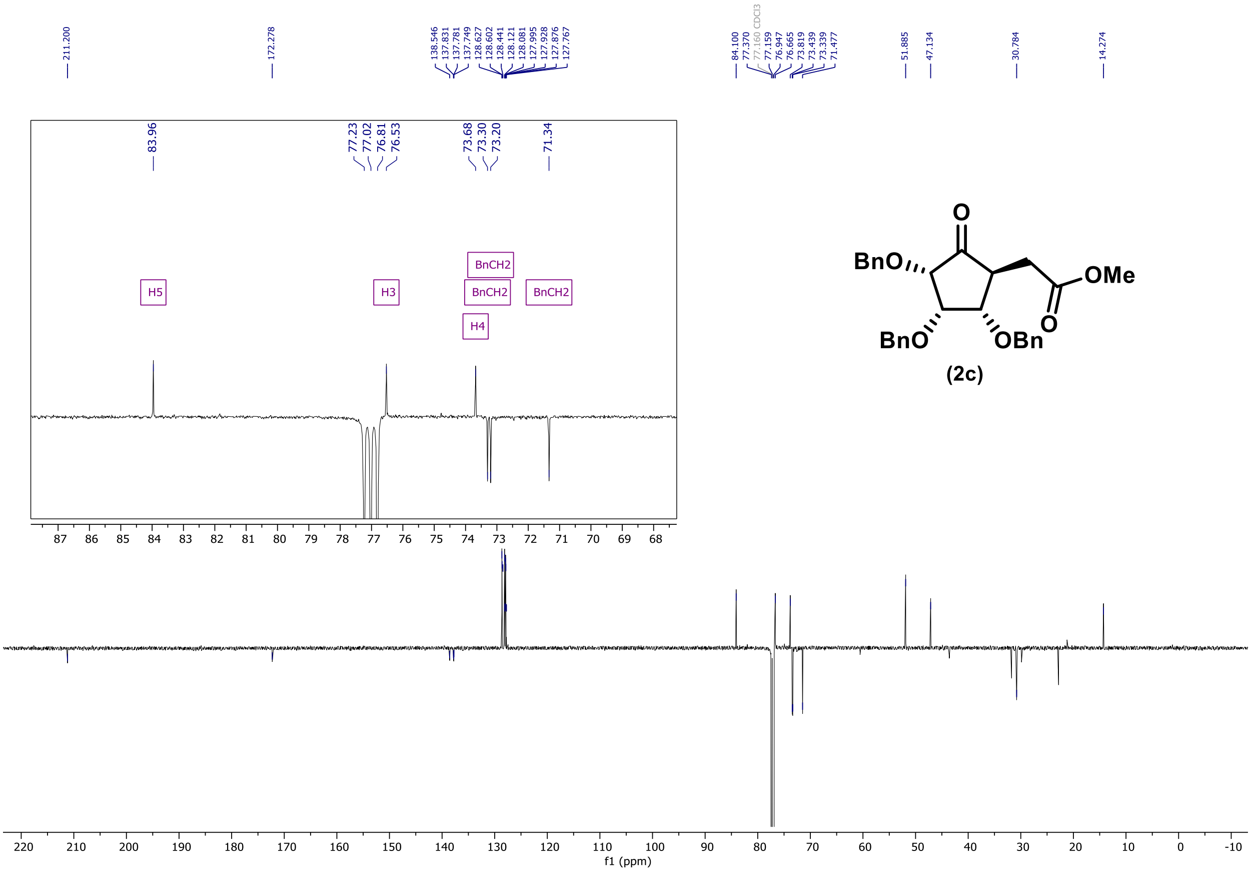

Figure S206. 151 MHz ¹³C{¹H}-NMR of (2c)

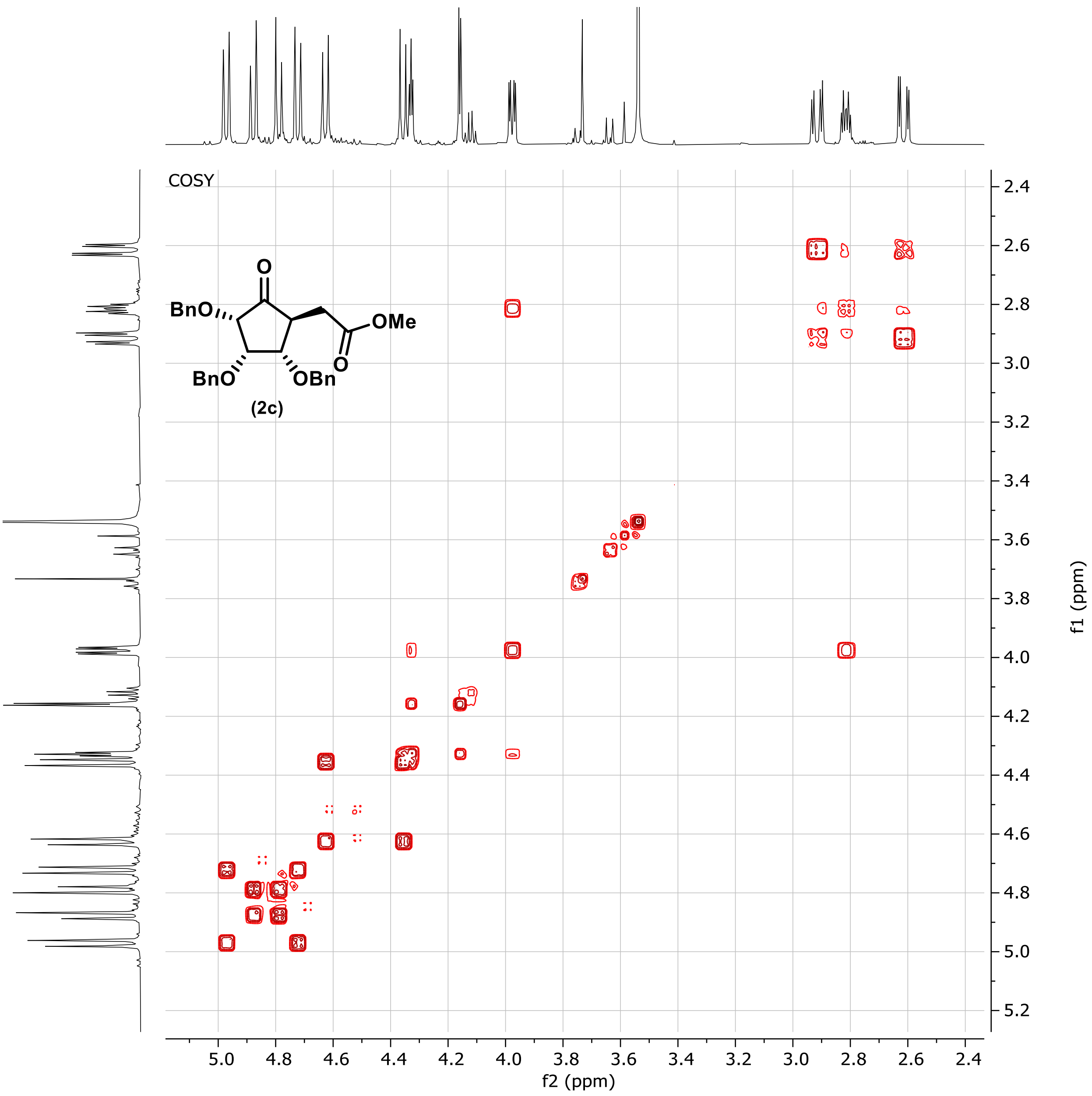

Figure S207. *COSY NMR of (2c)*

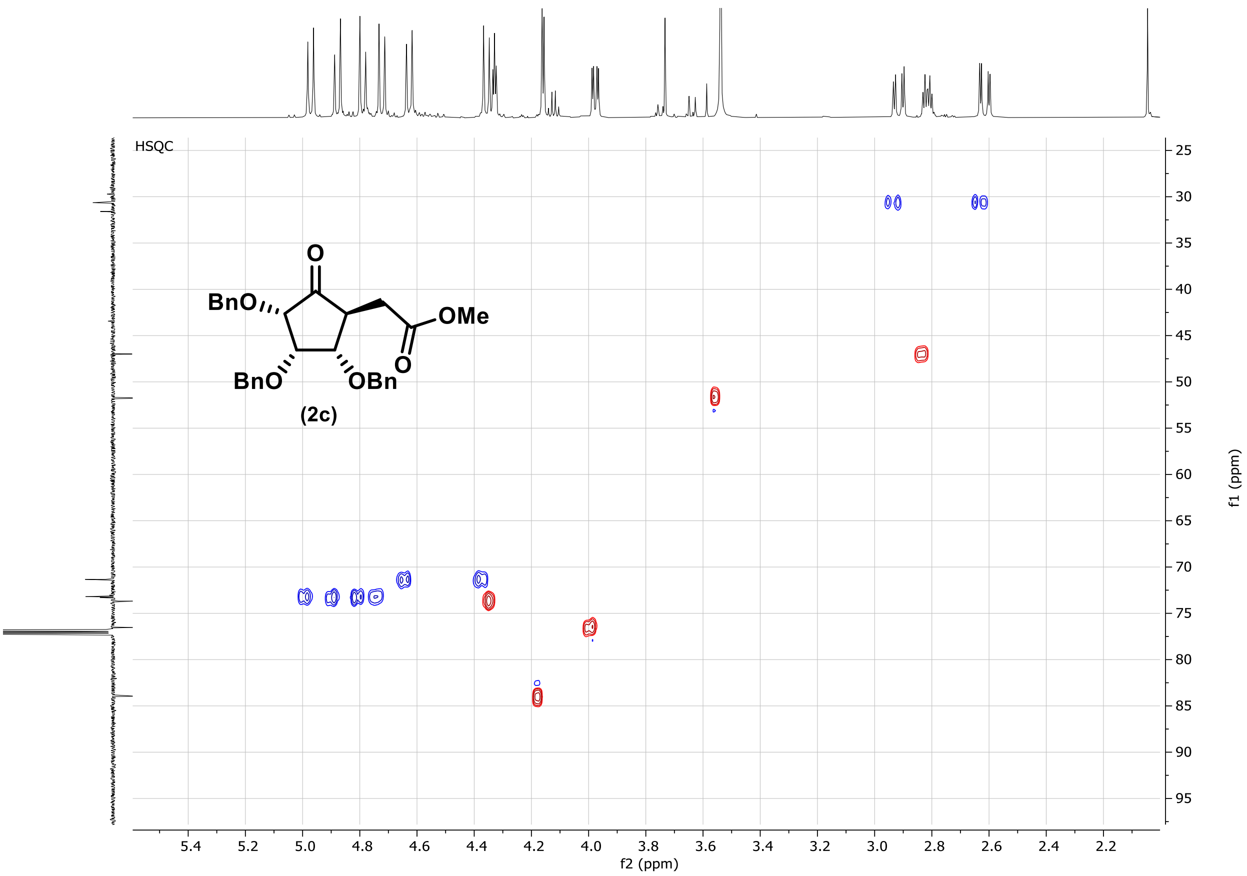

Figure S208. HSQC NMR of (2c)

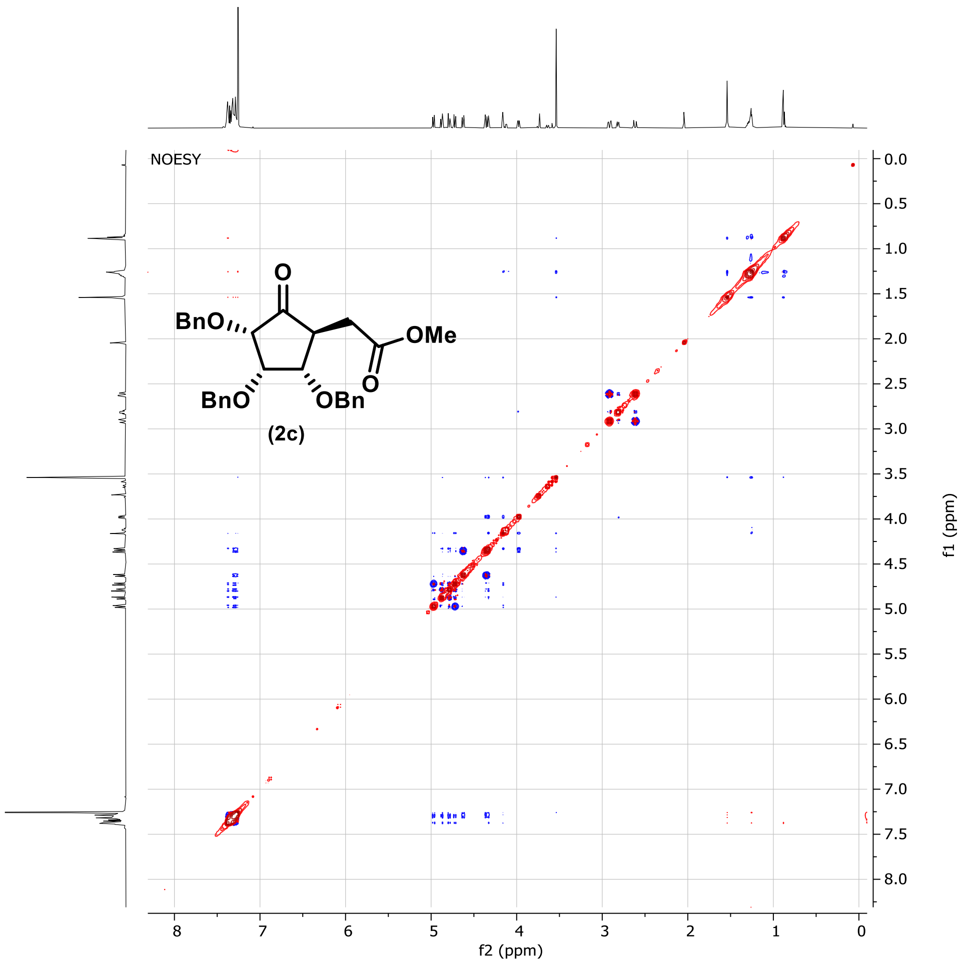

Figure S209. NOESY NMR of (2c)

D.5.2.3. *Methyl [(3S,4S,5R)-3,4,5-tris(benzyloxy)-2-oxocyclopentyl]acetate (Arabino) (2d)*

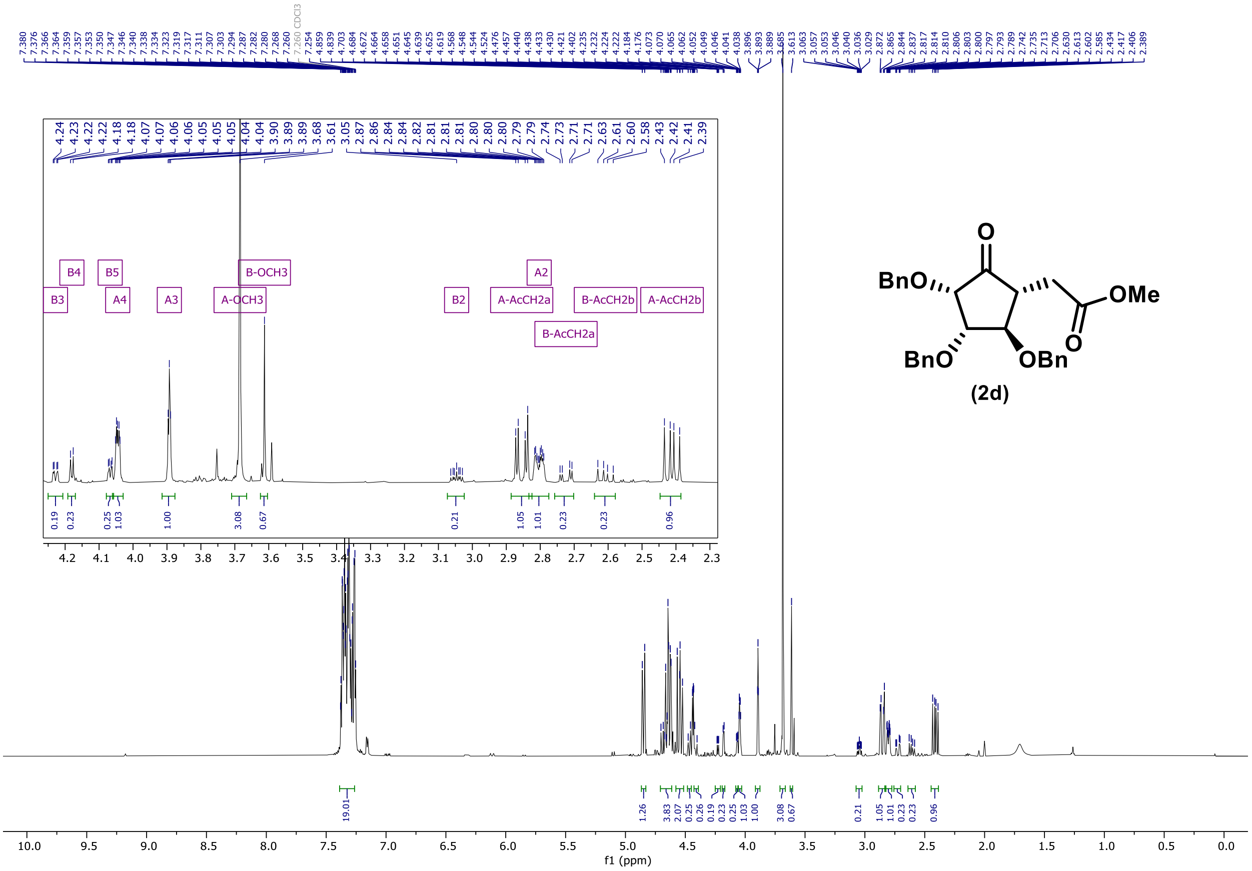

Figure S210. *600 MHz ^{1}H-NMR of (2d) as a mixture of syn/anti isomers ~ 1:5*

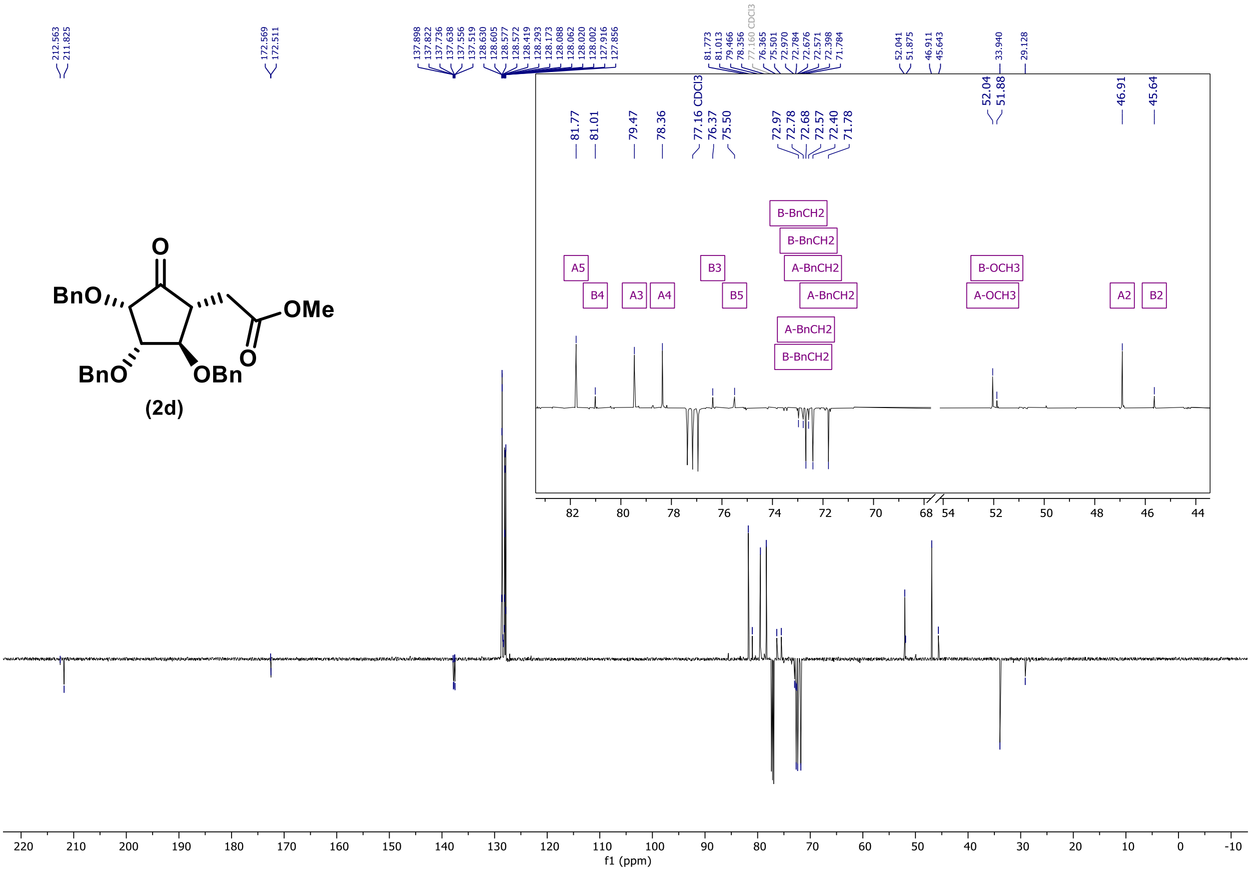

Figure S211. *151 MHz $^{13}C\{^{1}H\}$-NMR of (2d) as a mixture of syn/anti isomers ~ 1:5*

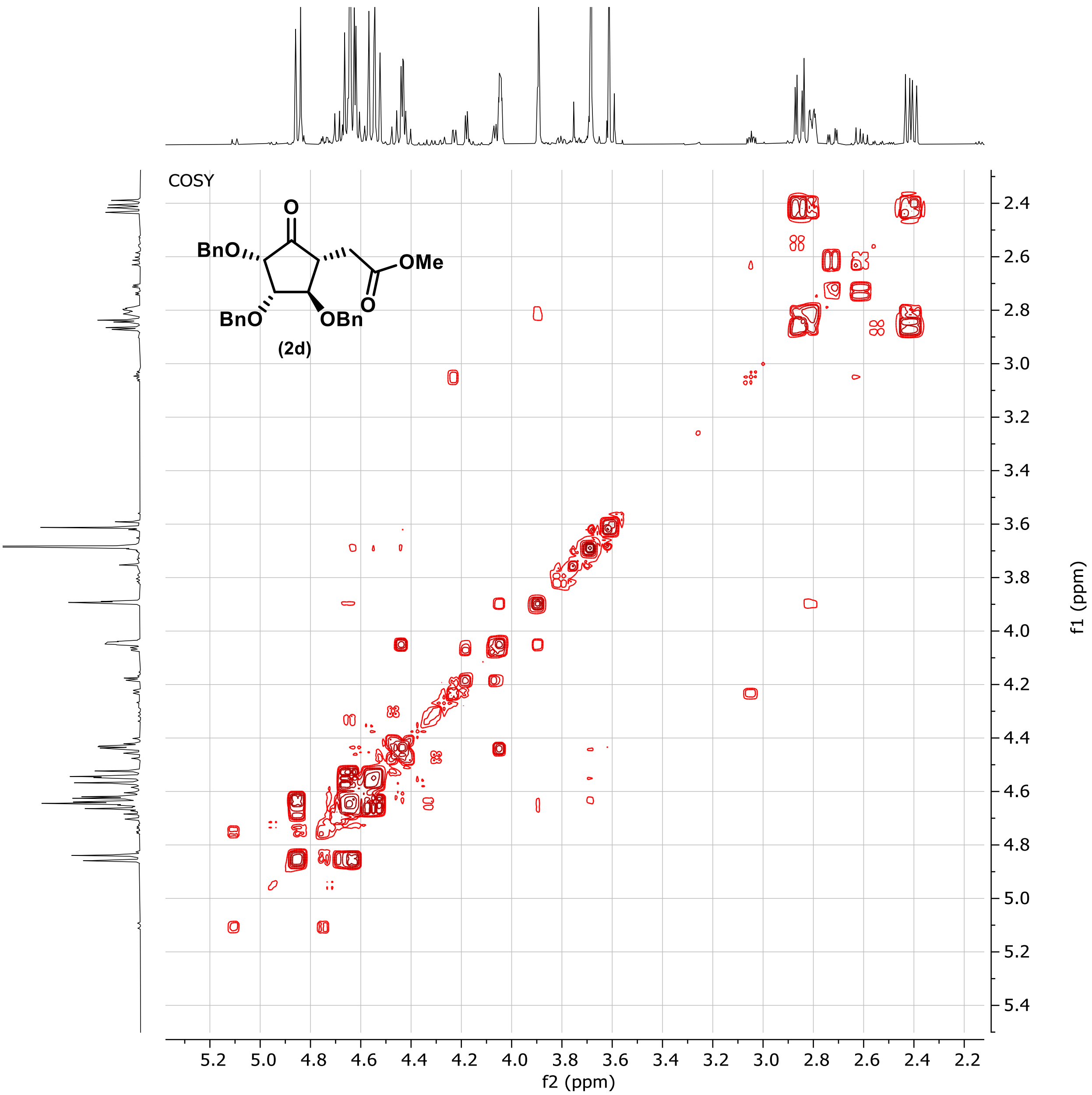

Figure S212. COSY NMR of (2d)

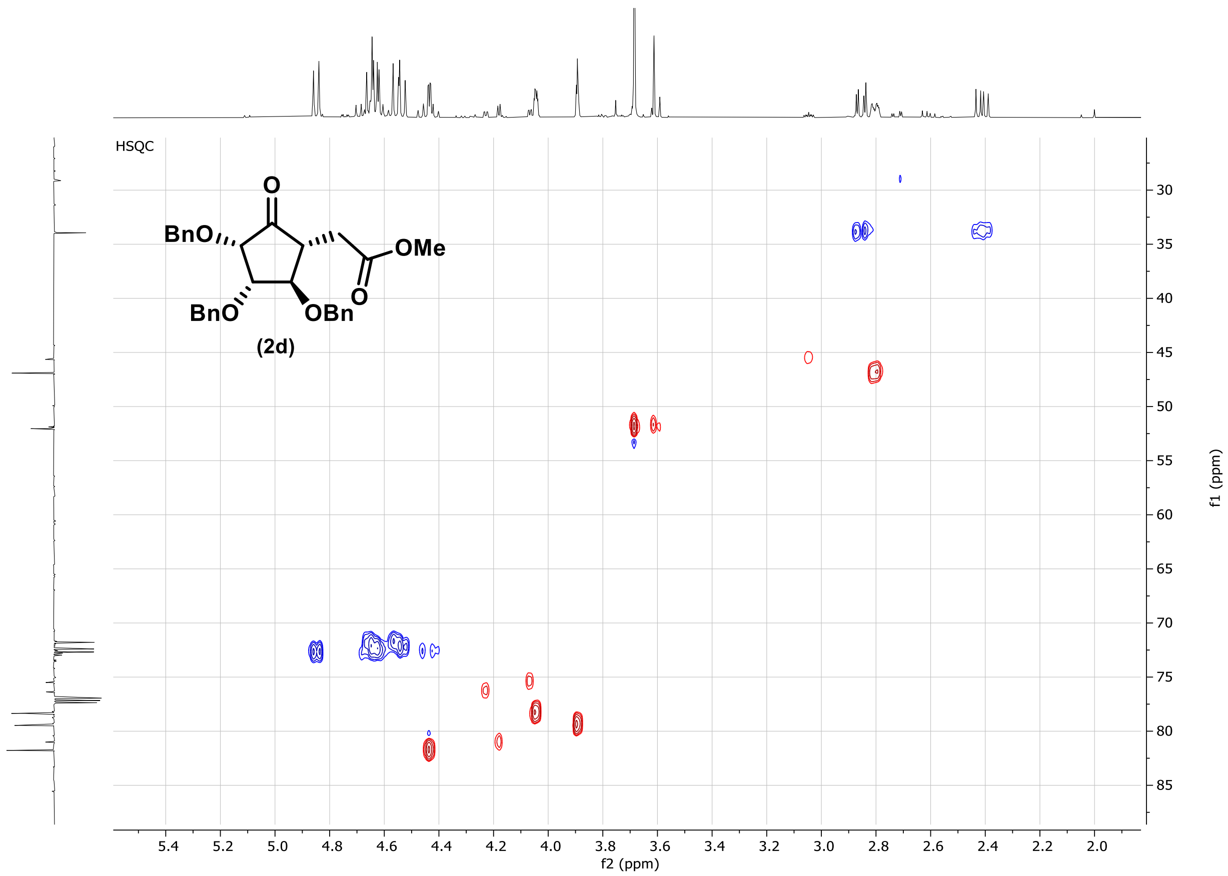

Figure S213. HSQC NMR of (2d)

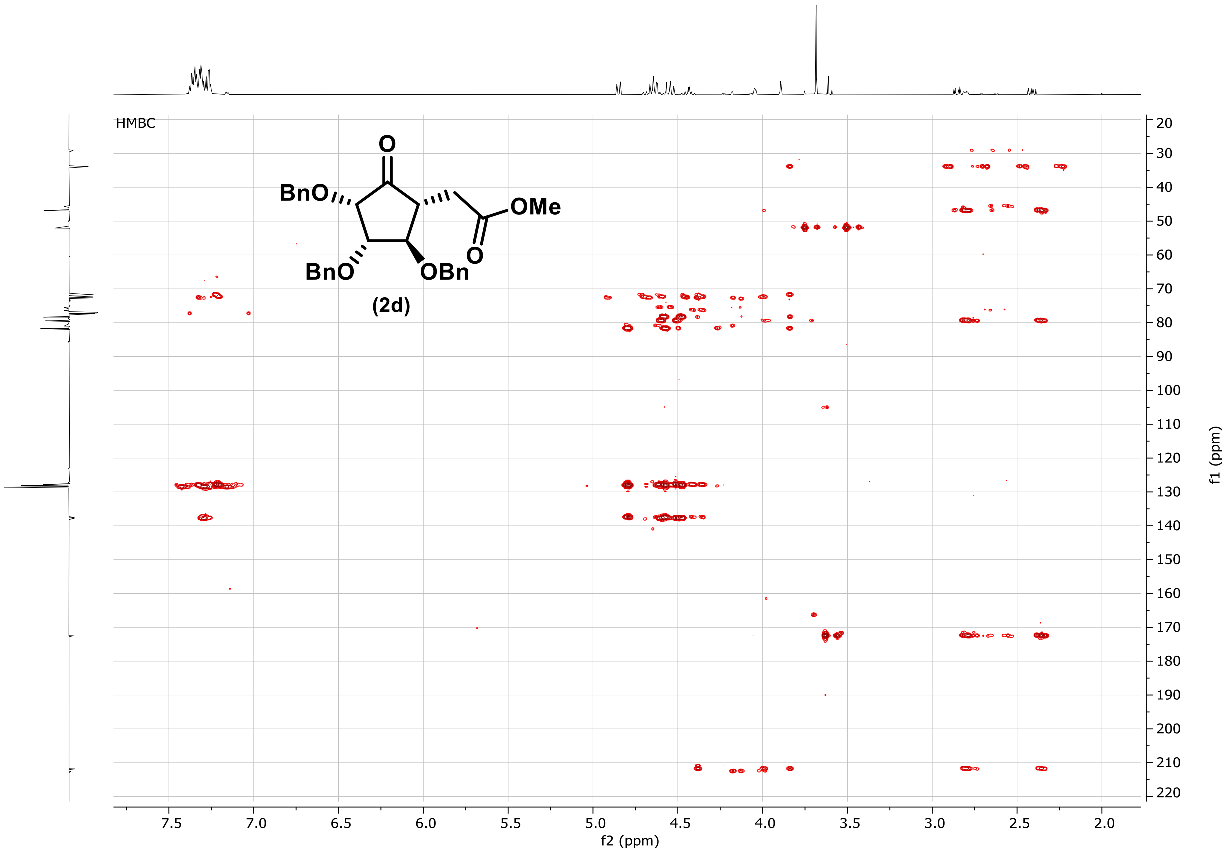

Figure S214. HMBC NMR of (2d)

D.5.2.4. Methyl [(3S,4S)-3,4-bis(benzyloxy)-5-oxocyclopent-1-en-1-yl]acetate (Ribo/Arabino) (3c)

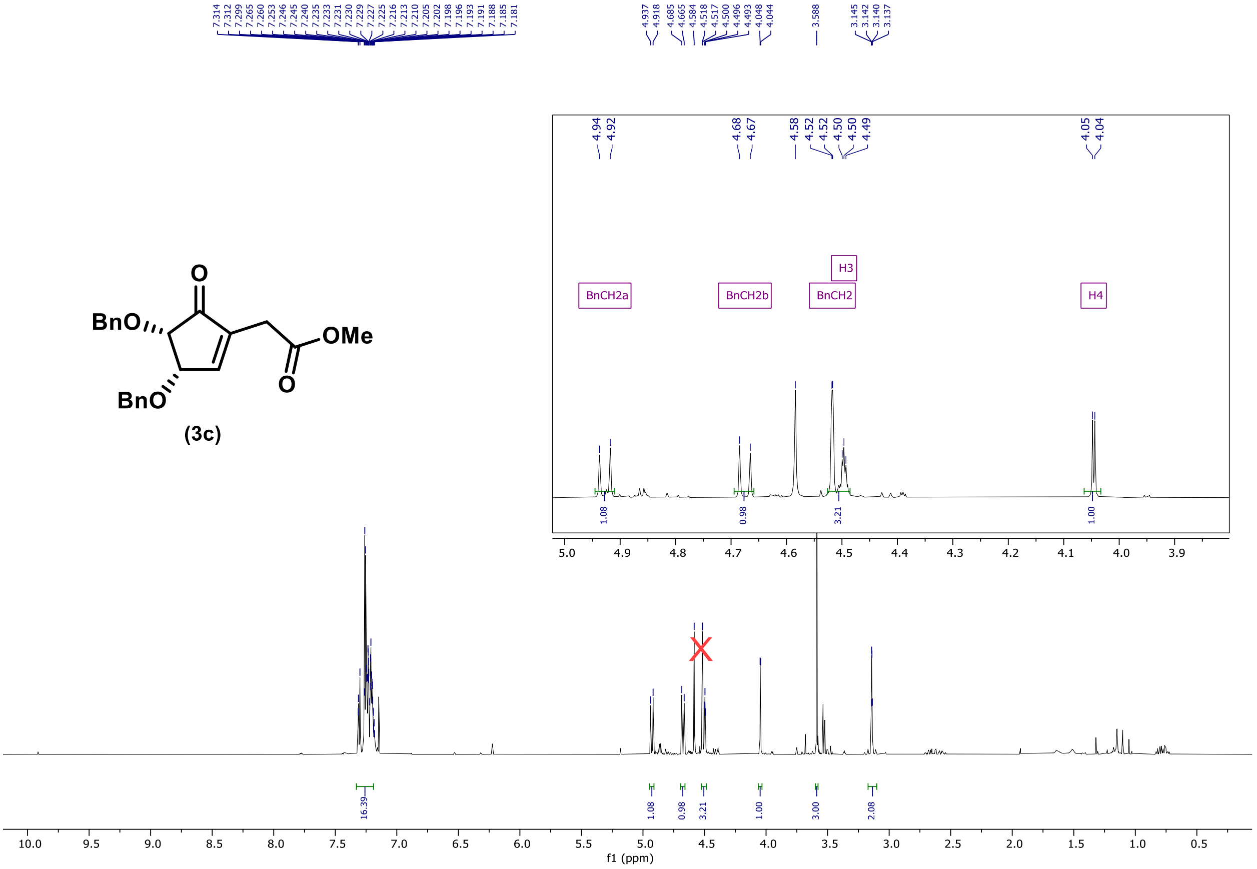

Figure S215. *600 MHz ^{1}H-NMR of (3c)*

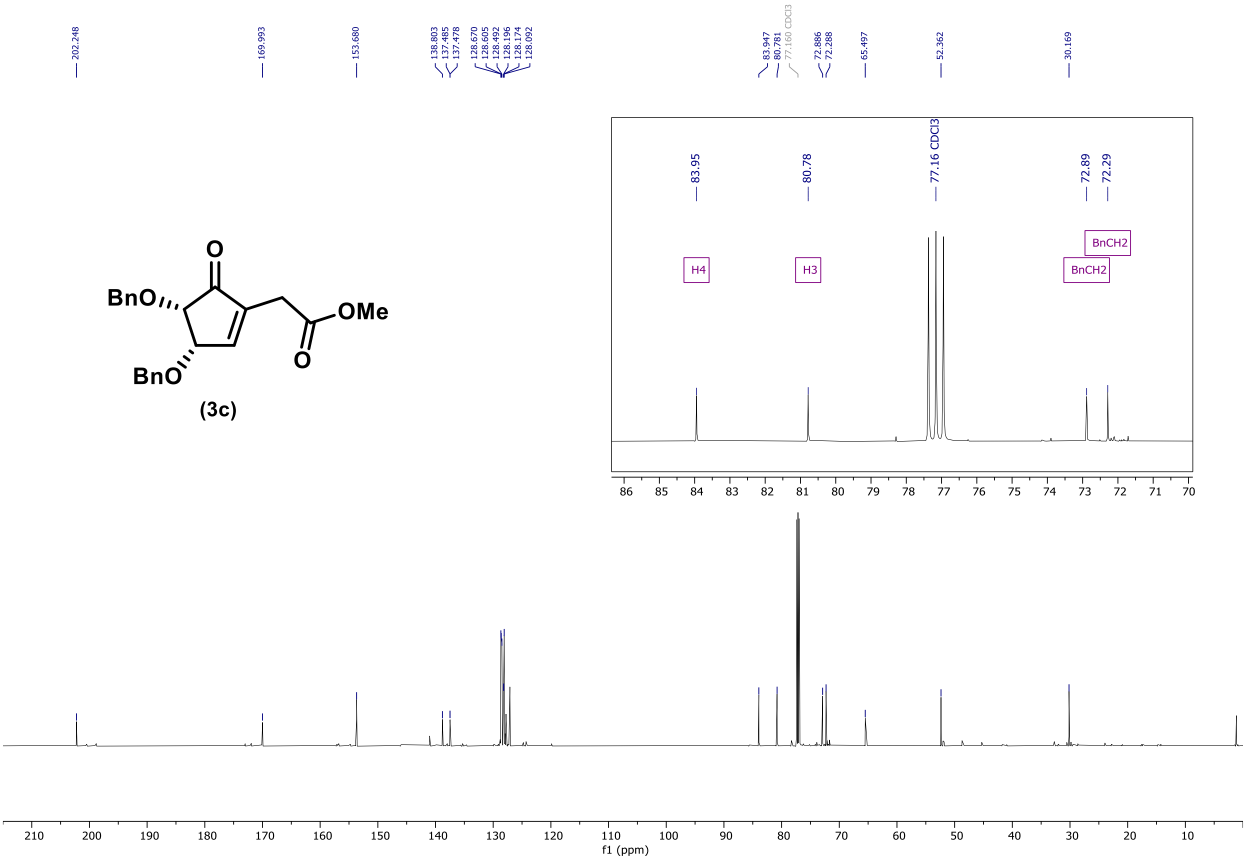

Figure S216. *151 MHz ^{13}C{^{1}H}-NMR of (3c)*

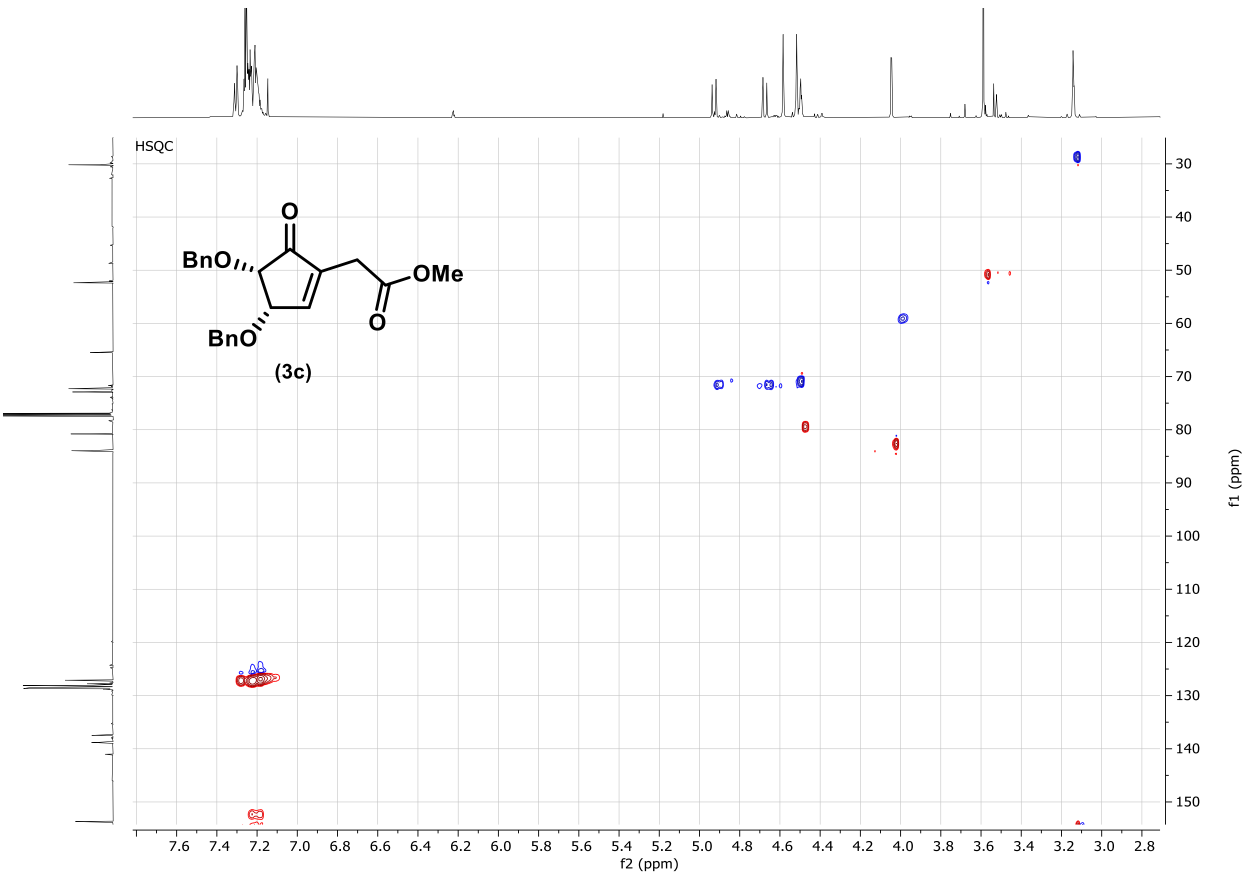

Figure S217. HSQC NMR of (3c)

E. LC-MS based Condition Screening

Reaction optimisation was done with xylose derived materials: aldehyde (1a) as pure E compound, carbocycles (2a) and eliminated product (3a).

E.1. Calibration

The materials that were used to create calibration standards, were first quantified via NMR using standard grade dimethylsulfone as internal standard. Spectra were recorded with a calibrated 90 ° pulse and 15 s relaxation delay on a 600 Mhz spectrometer. Spectra were processed with a 128K zero-filling and without the use of a window-function.
As solvent for all standard solutions LC-MS grade Acetonitrile with 2 mM addition of caffeine (as internal standard) was used.
For the aldehyde solution, 51.2 mg of material were dissolved in acetonitrile, resulting in a concentration of 10.78 mM. First, 1.00 mL of this solution were diluted with 1.00 mL of acetonitrile, giving a ~5.4 mM solution, and then 800 µL of the stock solution were diluted with 1.20 mL of acetonitrile, giving a ~4.3 mM solution. Those two solutions were each diluted 4 times 1:1, to give the standard concentrations indicated in *Table 1*.
For the stock solution of carbocyles and elimination product, 50 mg of a reaction mixture containing both diastereomeres and the eliminated product, was worked up, by extraction with 1M HCl solution, and evaporation. The resulting oil was quantified via NMR and then directly used to create calibration standards. 50.2 mg of material were dissolved in 10 mL of LC-MS grade acetonitrile. Resulting in the following concentrations: (Carbocycle major isomer: 5.61 mM, minor isomer: 2.32 mM, elimination product: 0.42 mM). 800 µL of this solution were diluted with 200 µL acetonitrile, to give another "stock solution". Both solutions were 3 times diluted 1 + 1 with acetonitrile to give standard concentrations indicated in *Table 1*
Samples were measured via LC-MS (C8 column, and quantified via PDA detector at 254 nm).

Table 1. Calibration overview for method optimisation. Each compound was calibrated using a 10 point calibration sequence. The resulting standard concentrations (after NMR quantification and dilution) are depicted in lines 2-11. Below linear regression parameters are indicated.

**Standard**	**Aldehyde (1a)** (mM)	**Carbocycle 1 (2x-anti)** (mM)	**Carbocycle 2 (2x-syn)** (mM)	**Elimination product (3)** (mM)
Std1	5.395	5.68	2.319	0.420
Std2	4.316	4.54	1.85	0.34
Std3	2.697	2.84	1.16	0.21
Std4	2.158	2.27	0.93	0.17
Std5	1.349	1.42	0.58	0.11
Std6	1.079	1.14	0.46	0.08
Std7	0.674	0.71	0.29	0.05
Std8	0.539	0.57	0.23	0.04
Std9	0.270	0.36	0.14	0.03
Blank	0	0	0	0
k	11629	7931.9	8063.7	13614
d	275.3	136.8	55.85	208.7
R^2	**0.9999**	**0.9999**	**0.99996**	**0.9976**

E.2. Screenings

E.2.1. Catalyst screening

For the catalyst screening, stock solutions of xylose-derived aldehyde (1a) (13.3 mg/mL), of Et_3N (8.5 mg/mL) in dichloroethane (DCE) were prepared. Further stock solutions of all catalysts in DMSO

were prepared according to *Table 2*. Each reaction was performed in a closed 8 mL screw-cap vial equipped with a magnetic stirring bar and a septum. First, 150 µL DCE were added, followed by aldehyde (1a) solution (750 µL) and catalyst solution (50 µL). The vial was placed into a metal heating block, and was pre-heated to 40 °C (internal temperature). The reactions were started by adding base solution (50 µL).

Table 2. *Stock solutions to perform catalyst screening. For each reaction, 750 µL aldehyde (1a), 50 µL of base solution, 50 µL respective catalyst and 150 µL DCE were added.*

Substance	Structure	Solvent	Concentrations (mM)	Concentrations (mg/mL)	Volume added (µL)
(1a)	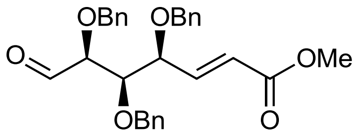	DCE	28.0	13.3	750
Et_3N		DCE	84.3	8.5	50
DBU		DCE	84.3	12.8	50
(A) C_6F_5	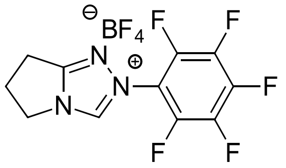	DMSO	105	38.2	50
(B) pTol	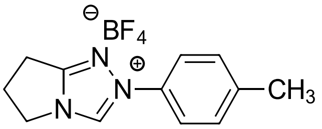	DMSO	105	30.2	50
(C) Mes	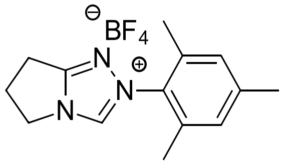	DMSO	105	33.2	50
TriPhen (D)	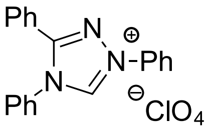	DMSO	105	41.9	50
Thiazol (E)	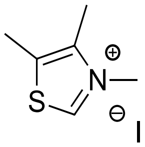	DMSO	105	26.9	50

After 30 min of stirring at 40 °C, approximately 300 µL of the reaction solution were taken out of the reaction vial via syringe into an Eppendorf vial, that was cooled via ice-bath to quench the reaction. Then 200 µL of the cooled reaction mixture were filled to 1 mL with HPLC grade acetonitrile (containing 2mM caffeine as internal standard).

Table 3. *Results of catalyst screenings. Yields are referenced to the entry without catalyst and base*

Cat.	Base	Conc. (mM) / Yield (%) (1a)	(2x – anti)	(2x – syn)	(3)	Conversion (%)
None	None	4.319 / 100	0 / 0	0 / 0	0 / 0	0
None	Et_3N	4.312 / 100	0 / 0	0 / 0	0 / 0	0
C_6F_5	Et_3N	0.097 / 2.2	2.152 / 49.6	1.59 / 36.8	0.041 / 0.9	87
Mes	Et_3N	3.137 / 72.6	0.38 / 1.5	0 / 0	0.021 / 0.5	2
Mes	DBU	0.872 / 20.2	0.263 / 6.1	0 / 0	0 / 0	6
*p*Tol	Et_3N	3.123 / 72.3	0.347 / 0.7	0 / 0	0 / 0	1
TriPhen	Et_3N	3.952 / 91.5	0.408 / 0.2	0 / 0	0 / 0	0
Thiazol	Et_3N	4.309 / 99.8	0.523 / 2	0 / 0	0 / 0	2

Thiazol	DBU	3.758 / 87.1	0.143 / 3.3	0 / 0	0 /0	3

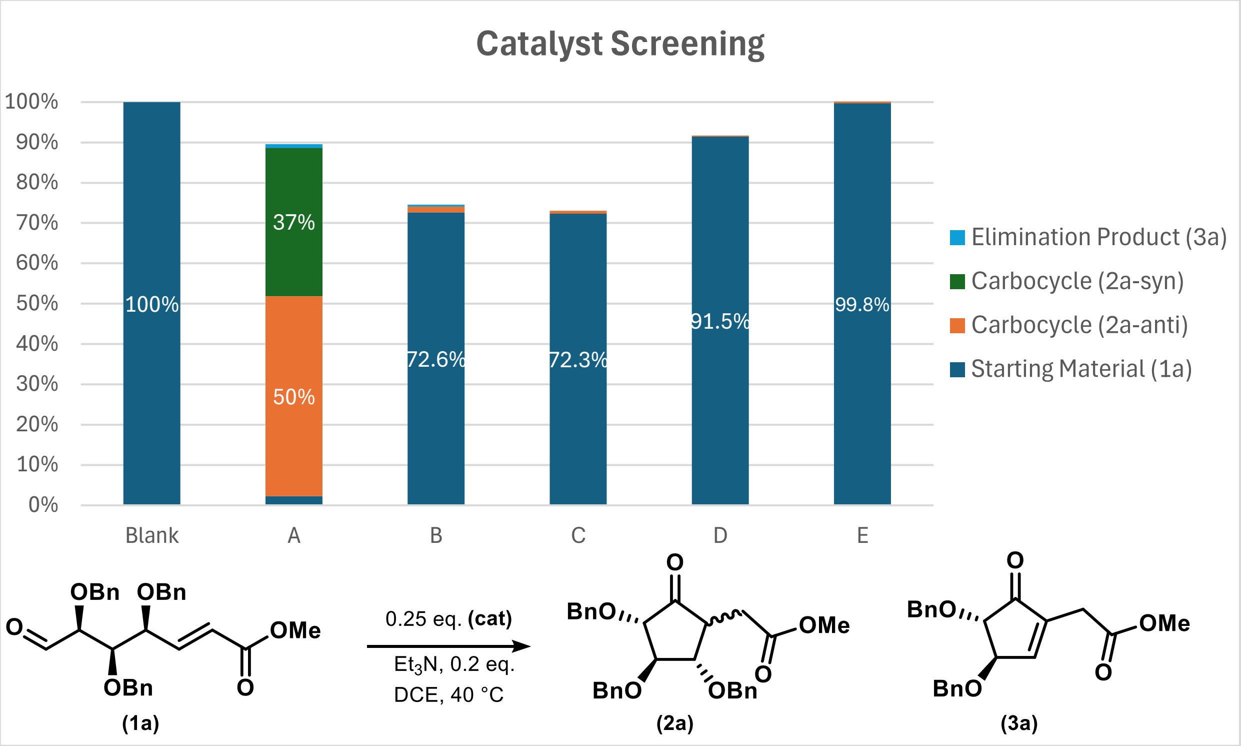

E.2.2. Other Screenings

All other screenings were performed with the same procedure as described above (*E.2.1*)
For the screening of solvents, stock solution of DBU was prepared in DMSO.

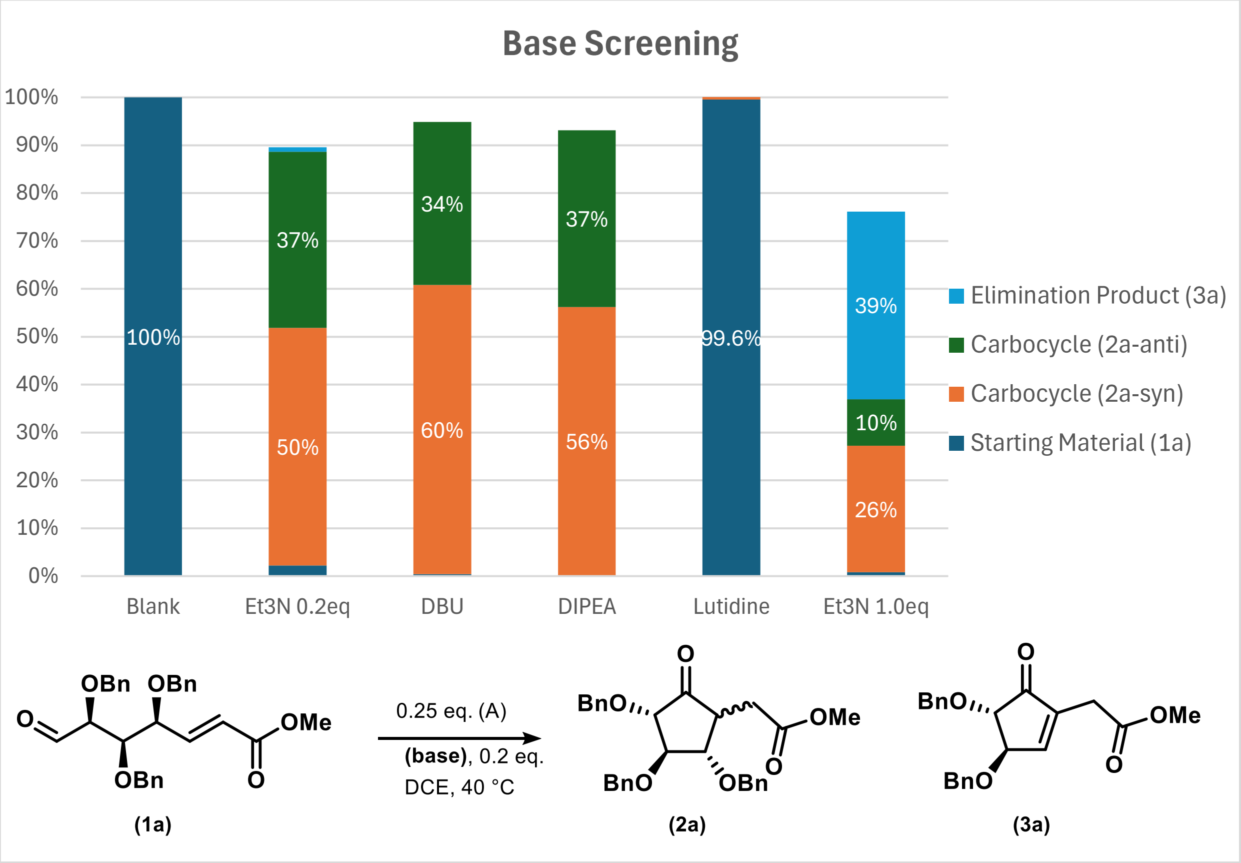

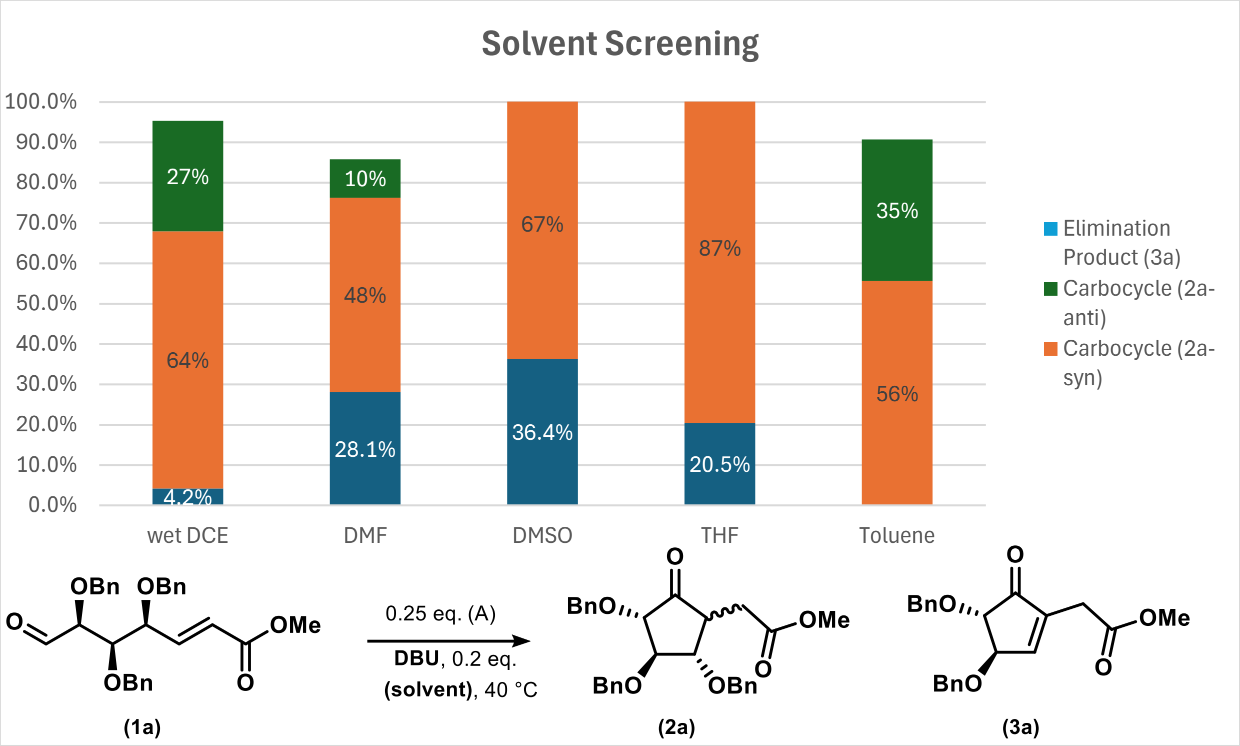

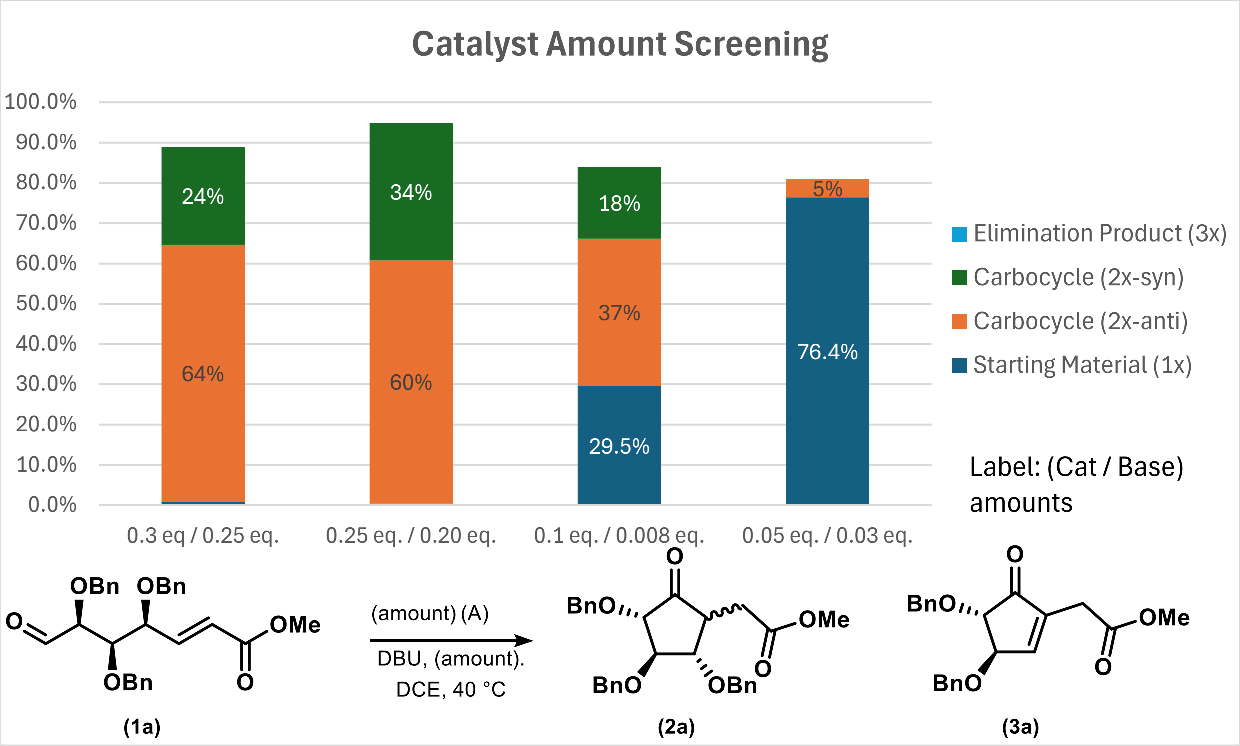

All optimisation experiments were performed within maximum 48 hours after calibration.

E.3. Influence of E/Z on diastereoselectivity and equilibration of diastereomeres

To test if the E/Z configuration of the starting aldehyde (1) has an influence on the reaction outcome, we performed a control experiment, with pure E-compound (1a) (same as the calibrated HPLC-UV screening), and compared the reaction outcome to a carbocyclisation reaction where the carbocyclisation was initiated from a 50/50 mixture of E/Z isomers. We found no significant change in the ratio of obtained isomers between the experiments (E/Z-Mixture gave 1:0.31, while pure E gave 1:0.34).

We then tested stability of the isomers under acidic conditions, by extracting a solution of the isomers obtained from the experiment stemming from pure E-compound, shaked in a separating funnel against 1N HCl for 10 min, this led to an equilibration of the isomers at a 1:1 ratio. Thus proving, that equilibration of isomers is a factor for the xylo-configured products (2a)

Reaction outcomes are visualized in *Figure S218.*

E.4. Influence of E/Z on diastereoselectivity and equilibration of diastereomeres

To test if the E/Z configuration of the starting aldehyde (1) has an influence on the reaction outcome, we performed a control experiment, with pure E-compound (1a) (same as the calibrated HPLC-UV screening), and compared the reaction outcome to a carbocyclisation reaction where the carbocyclisation was initiated from a 50/50 mixture of E/Z isomers. We found no significant change in the ratio of obtained isomers between the experiments (E/Z-Mixture gave 1:0.31, while pure E gave 1:0.34).

We then tested stability of the isomers under acidic conditions, by extracting a solution of the isomers obtained from the experiment stemming from pure E-compound, shaked in a separating funnel against 1N HCl for 10 min, this led to an equilibration of the isomers at a 1:1 ratio. Thus proving, that equilibration of isomers is a factor for the xylo-configured products (2a)

Reaction outcomes are visualized in *Figure S218.*

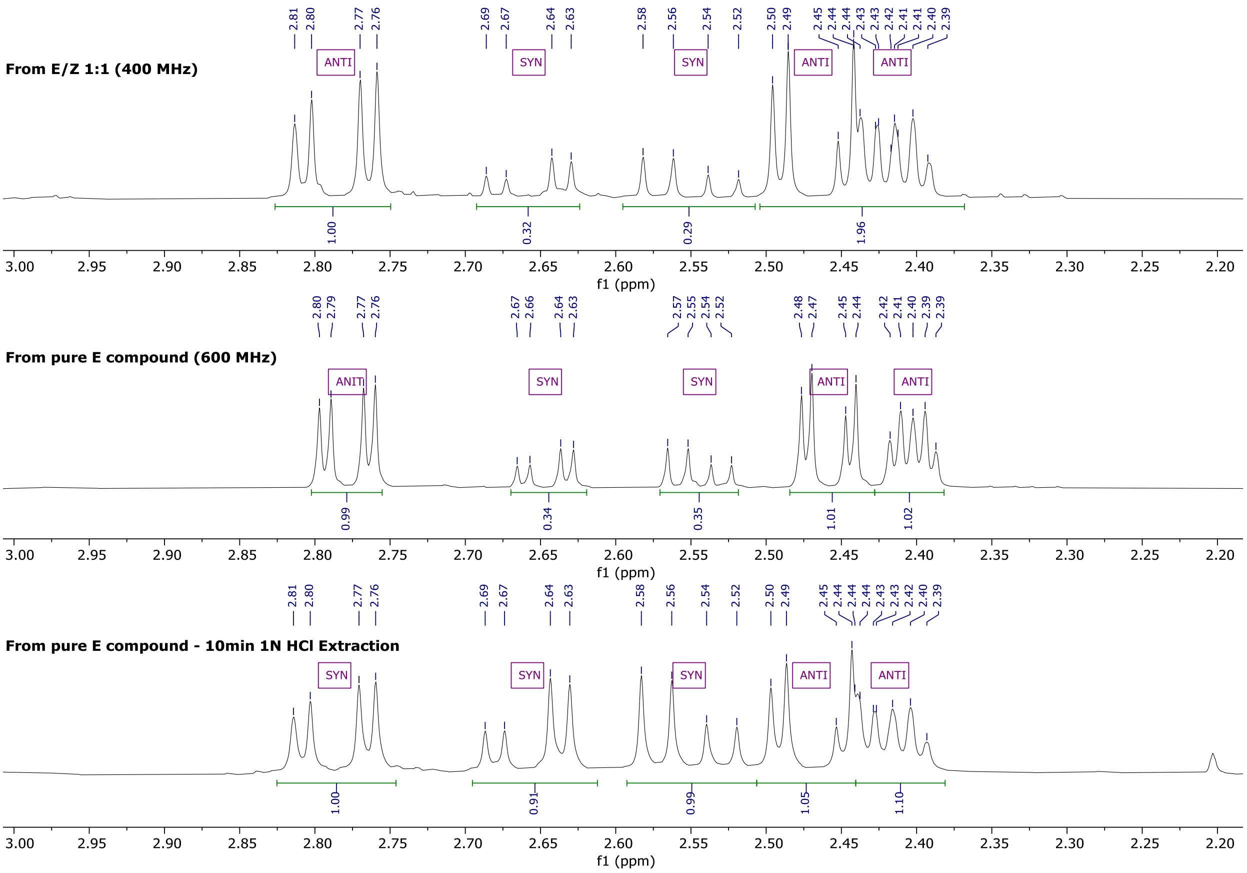

Figure S218. *NMR of control experiment, to test influence of E/Z configuration of the starting material on the diastereoselectivity of the reaction. Ratio between anti/syn compounds were similar for pure E compound (middle spectrum) and E/Z 1:1 mixture (top spectrum). We further tested equilibration behaviour of the xylo configured isomers (2a-syn) and (2a-anti) by treatment with 1N HCl for 10 min and saw equilibration of the isomers at 50/50.*

F. References

(1) Kerr, M. S.; Read de Alaniz, J.; Rovis, T. *The Journal of Organic Chemistry* **2005**, *70*, 5725.

(2) Langdon, S. M.; Gravel, M. *Organic Letters* **2022**, *24*, 7168.

(3) Choi, S.-h.; Mansoorabadi, S. O.; Liu, Y.-n.; Chien, T.-C.; Liu, H.-w. *Journal of the American Chemical Society* **2012**, *134*, 13946.

(4) Kireev, A. S.; Breithaupt, A. T.; Collins, W.; Nadein, O. N.; Kornienko, A. *The Journal of Organic Chemistry* **2005**, *70*, 742.

(5) Bennett, J. J.; Murphy, P. V. *Organic Process Research & Development* **2024**, *28*, 1848.

(6) Banachowicz, P.; Mlynarski, J.; Buda, S. *The Journal of Organic Chemistry* **2018**, *83*, 11269.

(7) Dolhem, F.; Smiljanic, N.; Lièvre, C.; Demailly, G. *Tetrahedron* **2006**, *62*, 7756.

(8) Lucero, C. G.; Woerpel, K. A. *The Journal of Organic Chemistry* **2006**, *71*, 2641.

(9) Biduś, N.; Banachowicz, P.; Buda, S. *Tetrahedron* **2020**, *76*, 131397.

(10) Mohal, N.; Vasella, A. *Helvetica Chimica Acta* **2005**, *88*, 100.

(11) Cachatra, V.; Martins, A.; Oliveira, M. C.; Oliveira, M. C.; Gano, L.; Paulo, A.; López, Ó.; Fernández-Bolaños, J. G.; Contino, M.; Colabufo, N. A.; Evans, D.; Man, T.; Rauter, A. P. *Organic & Biomolecular Chemistry* **2025**, *23*, 3845.
